# Transient-rare Bacterial Taxa Are Assembled Neutrally across Temporal Scales

**DOI:** 10.1264/jsme2.ME20110

**Published:** 2021-02-10

**Authors:** Sang-Hoon Lee, Taek-Seung Kim, Hee-Deung Park

**Affiliations:** 1 School of Civil, Environmental and Architectural Engineering, Korea University, Seoul, South Korea, 02841

**Keywords:** activated sludge, community assembly, neutral assembly, rare taxa

## Abstract

Despite the importance of microbial communities in ecosystem functions, the mechanisms underlying the assembly of rare taxa over time are poorly understood. It remains largely unknown whether rare taxa exhibit similar assembly processes to common taxa in local communities. We herein retrieved the 16S rRNA sequences of bacteria collected bimonthly for 2 years from the Pohang wastewater treatment plant. The transient-rare taxa showed different abundance distributions from the common taxa. Transient-rare taxon assemblages also exhibited higher temporal variations than common taxon assemblages, suggesting the distinct ecological patterns of the two assemblages. A multivariate analysis revealed that environmental parameters accounted for 25.3 and 61.6% of temporal variations in the transient-rare and common taxon assemblages, respectively. The fitting of all observed taxa to a neutral community model revealed that 96.4% of the transient-rare taxa (relative abundance, 71.4%) and 73.3% of the common taxa (relative abundance, 45.6%) followed the model, suggesting that stochastic mechanisms were more important than deterministic ones in the assembly of the transient-rare taxa. Collectively, the present results indicate that the transient-rare bacterial taxa at the Pohang wastewater treatment plant differed from the common taxa in ecological patterns, suggesting that dispersal is a key process in their assembly.

Local microbial communities influence ecosystem functions in their habitats. For example, local microbial communities are involved in plant growth in the soil (Nannipieri *et al.*, 2003), wastewater treatment in bioreactors (Briones and Raskin, 2003), and obesity in humans (Turnbaugh *et al.*, 2006). Microbial communities are typically characterized by species diversity (*e.g.*, richness or phylogenetic diversity) and relative abundance (community structure); however, these measures vary across spatiotemporal scales (Torsvik *et al.*, 2002). Despite the speculated significance of microbial community diversity in ecosystem functions (*e.g.*, see [Nemergut *et al.*, 2014]), the factors that drive and maintain the diversity dynamics of a community remain unclear. To better understand the mechanisms shaping local microbial communities, the ecological processes affecting the dynamic patterns of microbial diversity need to be evaluated.

Local microbes exhibit similar community structure patterns to larger organisms, namely, few taxa comprise a major part of the structure, while most are numerically rare. Therefore, the local community structure is strongly typified by rare microorganisms at both the micro- (Sogin *et al.*, 2006; Fuhrman, 2009) and macroecological scales (Grady *et al.*, 2019; Shade and Stopnisek, 2019; Morella *et al.*, 2020). Although rare species are often presumed to be inactive in local ecosystems, they contribute to ecosystem functions when environmental conditions become favorable for their growth (Shade and Gilbert, 2015). Nevertheless, the mechanisms underlying the assembly of rare taxa and their ecological functions within local habitats remain largely unknown.

There are two main perspectives explaining species assembly in local communities across spatiotemporal scales: the niche and neutral theories. According to the niche theory, competition for resources among species and their different abilities to utilize resources (*i.e.*, niche differentiation) (Hubbell, 2001; Nemergut *et al.*, 2013) shape the structure of local communities. According to the neutral theory, species at the same trophic level are equivalent, and the community structure is shaped by stochastic mechanisms involving immigration, emigration, birth, death, speciation, and ecological drift (Hubbell, 2001; Nemergut *et al.*, 2013). These two contrasting views are unlikely to be mutually exclusive; the processes underpinning the two theories function synergistically to shape the local community structure (Ofiţeru *et al.*, 2010; Ayarza and Erijman, 2011). Nevertheless, an outstanding question is how niche and neutral processes differently contribute to the dynamics of both rare and common taxa. The assembly of taxa with similar abundance (*e.g.*, rare *versus* common) or spatiotemporal distribution patterns (*e.g.*, persistent *versus* transient) may be driven by similar processes. Therefore, our overarching goal was to partition the contributions of niche and neutral processes to diverse community components, with a focus on processes governing the patterns of rarity.

Activated sludge bioreactors (Tchobanoglous and Burton, 1991) are the central units of biological wastewater treatment plants (WWTPs). In these bioreactors, naturally occurring microorganisms (mostly bacteria; >95%) are harnessed to degrade organic matter, oxidize inorganic nitrogen, and inactivate pathogens (Lee *et al.*, 2015). Microorganisms continuously enter and leave the bioreactor (<30 days of residence), in which they grow, die, and evolve. In this regard, each bioreactor may be viewed as an island at which ecological processes occur (Daims *et al.*, 2006). Therefore, a bioreactor may serve as a platform for testing community assembly theories at a tractable temporal scale relevant to microbial growth and community turnover. We herein used an activated sludge bioreactor to evaluate our hypothesis that niche and neutral processes contribute differently to the local assembly of rare and common taxa. To test this hypothesis, we used a 2-year time series of microbial community sequencing from the Pohang WWTP in South Korea. We focused our analysis on a comparison of the temporal dynamics of rare and common taxa and examined the relative contributions of niche and neutral processes to community assembly.

## Materials and Methods

### Sampling site, sample collection, and operational data

The Pohang WWTP (36.00°N, 129.35°E) treats approximately 160,000 m^3^ of domestic wastewater daily through a conventional activated sludge process (Rittmann and McCarty, 2001) with a low nutrient removal efficiency. Between May 2005 and April 2007, 12 samples were collected bimonthly at the same time of day from the bottom of the aeration tank of the treatment plant using a bucket grab sampler. Samples were immediately stored in an icebox and transferred to the laboratory for metacommunity DNA extraction. Plant operational data, including those related to biochemical oxygen demand, total nitrogen, total phosphorus, and mixed liquor, were collected. Suspended solids were analyzed using the standard method for the examination of water and wastewater (APHA *et al.*, 1989), and the data obtained are shown in Table S1. The flow rate, temperature, and dissolved oxygen level were monitored using installed online instruments.

### DNA extraction, PCR, and pyrosequencing

DNA from each sludge sample was extracted using the automated nucleic acid extractor (Obata *et al.*, 2001) Magtration System 6GC (PSS) following the manufacturer’s protocol. Nucleic acid purity was assessed based on absorbance at 260 nm and concentration (ng L^–1^) quantified using ND-1000 (NanoDrop Technologies). The universal primers, 27F (5′-AGAGTTTGATCMTGGCTCAG-3′) (Lane, 1991) and 518R (5′-ATTACCGCGGCTGCTGG-3′) (Muyzer *et al.*, 1993), were used to amplify the bacterial 16S rRNA gene from each sample. Purified PCR amplicons from each sample were subjected to barcoded 454 pyrosequencing (Genome Sequencer FLX Titanium Series; 454 Life Science) at Macrogen (Seoul) following the manufacturer’s protocol.

### Sequence analysis

Low-quality (<Q20) reads were removed from raw 16S rRNA sequences. Adaptor sequences were trimmed using the custom Perl script trimBarcode.pl (Macrogen). In addition, short reads (<300‍ ‍bp) and potential chimeric sequences were removed using Mothur (chimera.uchime) (Schloss *et al.*, 2009). After quality filtering, the phylogenetic affiliations of the sequence reads were assigned using the RDP Classifier (Wang *et al.*, 2007) for the taxonomic database (version 11.5). Operational taxonomic units (OTUs) were selected based on 97% sequence identity using Mothur (RDP Aligner, distance matrix, and UCLUST). Among all sequence reads, 2,988 (35,856 sequences and 4,999 OTUs from 12 samples) were randomly selected to reduce the error associated with the OTU richness estimation (Roesch *et al.*, 2007). Singleton OTUs (OTUs with only one sequence) were excluded from further statistical analyses (calculation of bacterial richness and generation of OTU rank abundance curves).

### Species (OTU) abundance distribution analysis

To evaluate how OTU abundance distribution patterns fit the theoretical models (*e.g.*, neutral and/or niche models), histograms of power-of-2 abundance classes (octave classes) of OTUs included in the rare-transient and common cohorts were plotted and then fit to the abundance models. Log-normal and log-series algorithms (Krebs, 1989) were applied to fit the model curves to each dataset, and the chi-squared test was used to evaluate the significance of each dataset. All statistical analyses were performed using PAST (version 2.12; http://folk.uio.no/ohammer/past/), and plots were generated using Sigmaplot (version 10.0; Jandel Scientific). A stacked bar plot of the relative distribution of transient-rare and other taxa at the phylum and proteobacterial class levels in the 12 activated sludge samples was generated using SigmaPlot 10.0.

### Non-metric multidimensional analysis (NMDS)

Temporal variations in transient-rare and other bacterial taxa were analyzed using NMDS ordination in PRIMER 6 (version 6.1.13; Primer-E) based on weighted UniFrac distances (Lozupone and Knight, 2005). A weighted UniFrac distance matrix for each group was generated for NMDS ordination by running the beta_diversity.py command on the equal-subsampled OTU table in QIIME (version 1.9.1, [Caporaso *et al.*, 2010]). The dimensionality of the ordination was selected by comparing the final stress values in R.

### Multivariate analyses

To assess the relative importance of environmental parameters in explaining patterns across the 12 activated sludge samples, a simplified redundancy analysis (RDA) and canonical correspondence analysis (CCA) were performed as described previously (ter Braak and Verdonschot, 1995; Van der Gucht *et al.*, 2007) using Canoco 4.5 (ter Braak and Smilauer, 2002). RDA and CCA were used for the common and transient-rare taxa, respectively, based on the results of a detrended correspondence analysis in Canoco 4.5. In both analyses, the total variation in the bacterial community matrix under unique environmental components with the corresponding *P* values was decomposed. The significance of each operational factor was evaluated with a Monte Carlo permutation test (999 permutations under the null hypothesis) using the forward selection method (ter Braak and Verdonschot, 1995). The explanatory environmental (E) variable was employed to measure the degree of variation (computed as the percentage of total variation for axes 1 and 2) using the multivariate extension of the linear regression with the corresponding R^2^ values. Unexplained variation was calculated as (1–[E]). To test our hypothesis by assessing differences in the community structure between the transient-rare and common taxa, a permutational multivariate analysis of variance (PERMANOVA) was performed based on weighted UniFrac distances. Additionally, to test the null hypothesis (no difference in dispersion between groups), a permutational multivariate dispersion (PERMDISP) test was conducted. PERMANOVA and the PERMDISP test were performed using the “adonis” and “betadisper” functions of the vegan package in R, respectively.

### Sloan and beta-abundance null model fitting

To evaluate the contribution of the neutral processes to community assembly, the OTU occurrence frequency and abundance data were fit to the Sloan neutral community model (Sloan *et al.*, 2006). This model was developed to predict the relationships between the occurrence frequencies and mean relative abundances of taxa sampled from an activated bioreactor at a given time point. The goodness-of-fit of the observed dataset to the model was calculated using the following equation: model fit=1–SS_err_/SS_total_ (generalized R^2^; [Östman *et al.*, 2010]), where SS_err_ is the sum of squares of residuals and SS_total_ is the total sum of squares. Moreover, to establish whether incorporating drift and dispersal limitations improves the model fit, fitness was compared between the neutral model and a binomial distribution model beyond the randomly sampled source metacommunity (Sloan *et al.*, 2006). All plots and statistical analyses were performed with the minpack.lm package in R (R Core Team, 2015) (Elzhov *et al.*, 2013) using the script provided by Burns *et al.* (2015). Moreover, 95% confidence intervals around model predictions were calculated using the HMisc package (Wilson score interval) in R (Brown *et al.*, 2001; Harrell Jr. and Dupont, 2006). The Student’s *t*-test was used to compare the taxonomic compositions of assemblages using R.

Beta-abundance null model fitting was performed based on both Bray–Curtis and weighted UniFrac distances for each transient-rare and common taxon OTU matrix using custom R scripts provided by Tucker *et al.* (Tucker *et al.*, 2016). Abundance null deviation values closer to zero indicate neutral communities in which species are ecologically equivalent to one another (Tucker *et al.*, 2016; Lee *et al.*, 2017).

### Dynamic simulation of the community assembly dataset

To confirm the effects of definition changes on the OTU classification at the detection limit using a dynamic simulation, 60 different dataset combinations (12 different relative abundances and 5 different occurrence frequencies) of various relative abundances and the occurrence frequencies of transient-rare and common taxa were used. The species abundance histograms of both transient-rare and common taxa were plotted from each dataset. Log-series and log-normal regression curves were then fit, and the goodness-of-fit (chi-squared test) of each regression was tested using both observed and expected regression values (Table S6). The closed box in Table S6 represents the dataset with the same transient-rare and common taxa.

### Accession numbers

The 16S rRNA gene sequences obtained in the present study are deposited in GenBank under the accession numbers HQ462572 to HQ524318.

## Results

### Definition of rare taxa

We performed 16S rRNA pyrosequencing to assess microbial community diversity. After removing singleton sequence reads (reads with a single sequence occurring only once in the dataset), 2,544 unique OTUs were retrieved at a 3% identity cut-off from 35,856 sequence reads of 12 samples collected bimonthly over 2 years (Table S2). Ranked taxon abundance distribution (Fig. S1) showed few abundant (*i.e.*, common) taxa and many rare taxa, similar to the commonly reported distributions of microorganisms in the soil, sediment, freshwater, and seawater (Nemergut *et al.*, 2011; Kim *et al.*, 2013; Mariadassou *et al.*, 2015; Roguet *et al.*, 2015). Rare taxa were defined as OTUs comprising less than 1% of all sequence reads (<334 reads), and were further classified as transient- (<4 occurrences [33%]) and persistent-rare taxa (>8 occurrences [66%]). Transient-rare taxa constituted 74.7% of the observed OTUs and 28.7% of the total sequence reads, but were numerically less important than the common taxa (Table S4). Persistent-rare taxa constituted 1.5% of the observed OTUs and 6.2% of the total sequence reads. Thereafter, analyses were focused on comparisons between the transient-rare and common taxa.

### Ecological patterns of transient-rare taxa

**Fig. 1.**
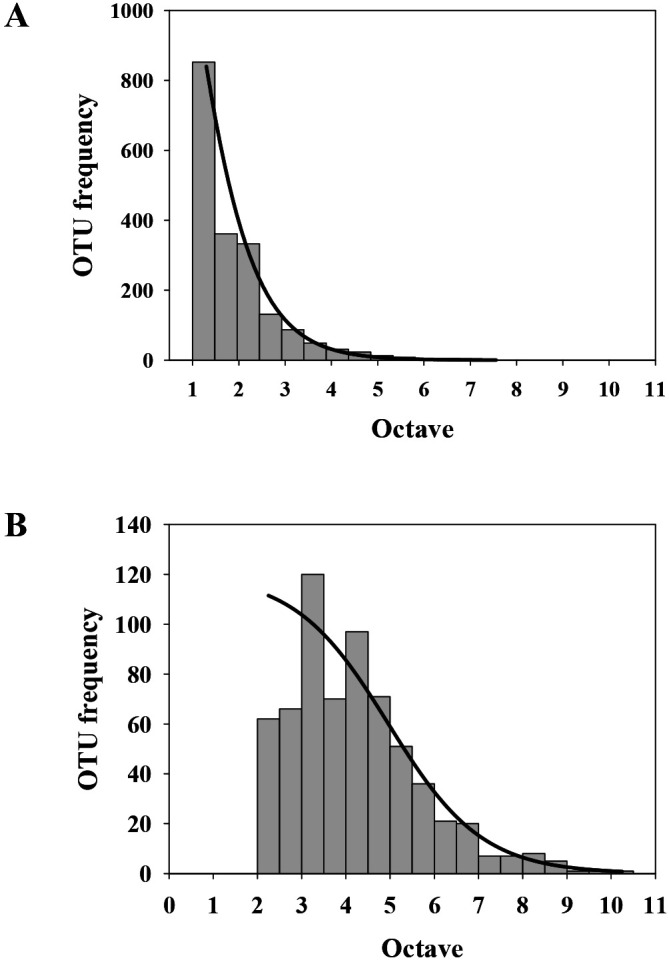
Operational taxonomic unit abundance distribution of (A) transient-rare and (B) common taxa. The fitted lines predicting the frequency and abundance of each taxon were obtained based on log-normal and log-series models. The octaves represent power-of-2 abundance classes.

OTU abundance distributions were analyzed based on the logarithmically binned abundance of the transient-rare and common taxa (Fig. 1). In the bioreactor, the two groups of taxon assemblages demonstrated very different patterns. The transient-rare taxa followed a log-series distribution (*χ^2^*=29.14, *P*<0.0001) (Fig. 1A), whereas the common taxa followed a near log-normal distribution (*χ^2^*=843.6, *P*<0.0001) (Fig. 1B). These results remained unchanged even when the definition of the transient-rare taxa was changed (relative abundance of 0.05–4.00% and occurrence frequency of 2–6; Table S6). Since species abundance distribution is affected by metacommunity diversity and the immigration rate (Brown and Kodric-Brown, 1977; Shmida and Wilson, 1985) and the taxon groups defined in this study were assembled from the same regional species pool, these two groups of taxa were expected to show similar abundance distributions. Accordingly, differences in their abundance distributions may be indicative of different contributions of niche and neutral processes in the bioreactor.

### Temporal variations in transient-rare taxon assembly

**Fig. 2.**
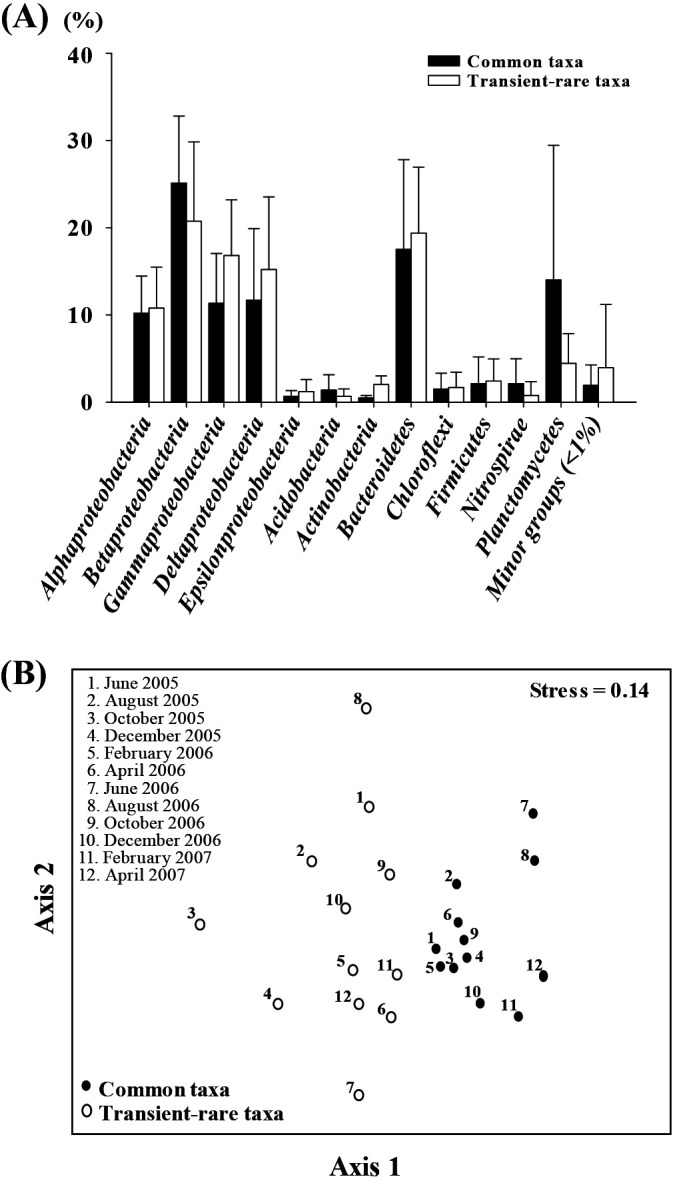
Taxonomic affiliations of transient-rare taxa and their temporal variability. (A) Classification of the transient-rare and common taxa according to the phylum and proteobacterial class levels. The error bars indicate the SD of 12 samples. (B) Non-metric multidimensional analysis ordination based on the weighted UniFrac distances for the assemblages of the transient-rare and common taxa.

We assessed the community composition of the transient-rare and common taxa by taxonomically classifying the observed OTUs at the phylum and proteobacterial class levels (Fig. 2A). Overall, the taxonomic compositions of the two assemblages were very similar (*t*-test, *P*=1.000). *Betaproteobacteria* and *Bacteroidetes* were the two major groups, followed by *Gammaproteobacteria*, *Alphaproteobacteria*, and *Deltaproteobacteria*. Nevertheless, a few groups (*e.g.*, *Epsilonproteobacteria*, *Nitrospirae*, and *Actinobacteria*) showed significant differences between the transient-rare and common taxa (*P*<0.05). The composition of the two cohorts was evidently different at the family level. A Venn diagram analysis demonstrated that specific families belonged to each cohort. Therefore, among 162 families, 60 and 11 families were only observed in the transient-rare and common cohorts, respectively (Fig. S2). Furthermore, the temporal community dynamics of the transient-rare and common taxa were compared across the samples. The relative abundance of the transient-rare taxa ranged between 18.4 and 40.9% and that of the common taxa ranged between 58.5 and 85.4%; however, the overall community composition of the two cohorts remained relatively stable over time (Fig. S3). The transient-rare taxa were dominated by *Betaproteobacteria* (average and standard deviation: 6.0±3.0%) and *Gammaproteobacteria* (average and standard deviation: 5.0±2.5%), while the common taxa were dominated by *Betaproteobacteria* (average and standard deviation: 17.7±5.2%) and *Bacteroidetes* (average and standard deviation: 12.3±6.6%). However, the relative abundance of the transient-rare and common taxa did not significantly differ across the samples over time (*P*>0.05). Moreover, temporal variations were investigated using NMDS ordination based on weighted UniFrac distances (Lozupone and Knight, 2005) between samples (Fig. 2B), which demonstrated that the phylogenetic distances between assemblages of the transient-rare taxa (average, 0.366 in 12 samples) were greater than those between assemblages of the common taxa (average, 0.275 in 12 samples). This result indicates a higher temporal variation in the transient-rare taxa than in the common taxa with respect to the phylogenetic distance (PERMDISP, *F*=8.011 and *P*=0.013). In addition, distinct clusters of the assemblages of the transient-rare and common taxa demonstrated that the two assemblages were phylogenetically distinct (PERMANOVA, *F*=8.011 and *P*<0.001) at the OTU level (97% sequence identity), which was not evident at the phylum and proteobacterial class levels (Fig. 2A). PERMANOVA and the PERMDISP test for the two cohorts based on unweighted UniFrac distances also supported our inference.

### Deterministic and stochastic processes contributing to the community assembly

The effects of explanatory variables related to the bioreactor (*i.e.*, environmental and operational parameters) on temporal variations in the two assemblages were assessed by quantifying their explanatory power using multivariate analyses (CCA for the transient-rare taxa and RDA for the common taxa) (Kelley, 1940). Twelve deterministic factors (temperature, dissolved oxygen, pH, hydraulic retention time, solid retention time, and mixed liquor suspended solids in the bioreactor and the biochemical oxygen demand, total nitrogen, and total phosphorus of the influent and effluent; Table S3) were tested, and the significance of differences in community assembly and environmental factors was estimated (Table S3). Collectively, these parameters may explain 23.5 and 63.4% of the variations in the transient-rare and common taxa, respectively. The significance of the results of RDA and CCA was also calculated for both the transient-rare and common taxa (Table S3). While the scores for both axes were significant for the transient-rare taxa (both *P*<0.01), the score was significant for axis 1 alone for the common taxa (for axes 1 and 2, *P*=0.04 and 0.382, respectively).

**Fig. 3.**
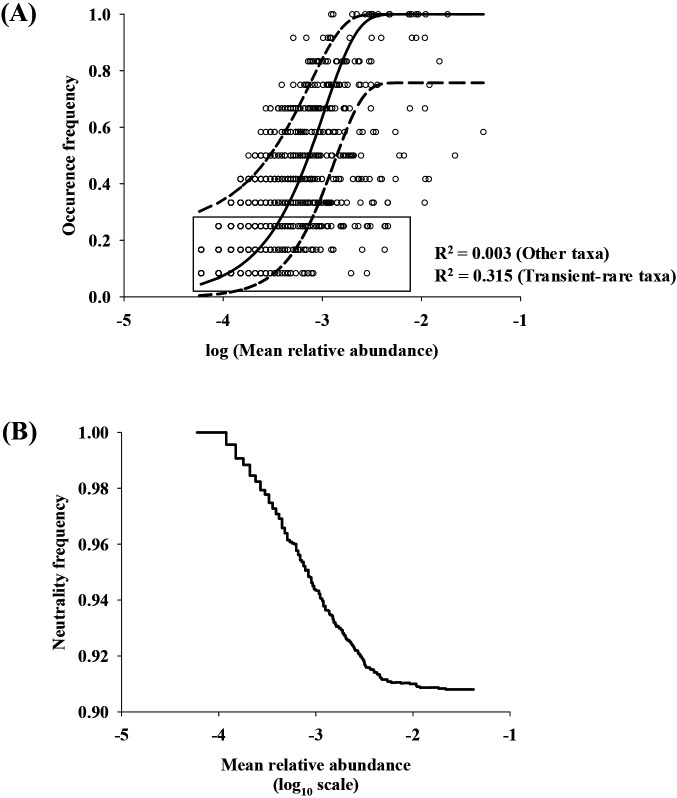
Evaluation of neutral community assembly. (A) Fitting of the operational taxonomic units (OTUs) observed in the bioreactor to the Sloan neutral community model. The solid line indicates the best fitting line, and dashed lines represent the 95% confidence intervals of the model. The transient-rare taxa are indicated in the inserted box. (B) Neutrality fraction of the observed OTUs as a function of mean relative abundance.

Furthermore, the contribution of neutral processes to the assembly of the transient-rare taxa was tested by fitting the OTU occurrence frequency and abundance data to the Sloan neutral community model (Sloan *et al.*, 2006). This model was developed to explain the occurrence and abundance patterns of prokaryotic communities based on dispersal and ecological drift and successfully described the neutral assembly of bacterial communities in activated sludge bioreactors (Ofiţeru *et al.*, 2010), lakes (Roguet *et al.*, 2015), human lungs (Venkataraman *et al.*, 2015), trees (Woodcock *et al.*, 2007), and zebrafish guts (Burns *et al.*, 2015). Fig. 3A shows the goodness-of-fit scores of the neutral model to all OTUs observed in the present study (solid line) with 95% confidence intervals of the prediction (dashed lines). The proportion of taxa (inserted box in Fig. 3A) within 95% confidence intervals was 96.4% (relative abundance, 71.4%) for the transient-rare taxa and 73.3% (relative abundance, 45.6%) for the common taxa, and both assemblages followed the neutral community model (Table S4). The neutrality fraction (defined as the fraction of OTUs within 95% confidence intervals of the neutral model) was inversely proportional to the mean relative abundance (Fig. 3B), and this result remained unchanged even when the definition of the transient-rare taxa was changed (relative abundance of 0.05–4.00% and occurrence frequency of 2–6; Table S6). Therefore, when taxa are relatively rare, neutral processes are more important for explaining their patterns.

**Fig. 4.**
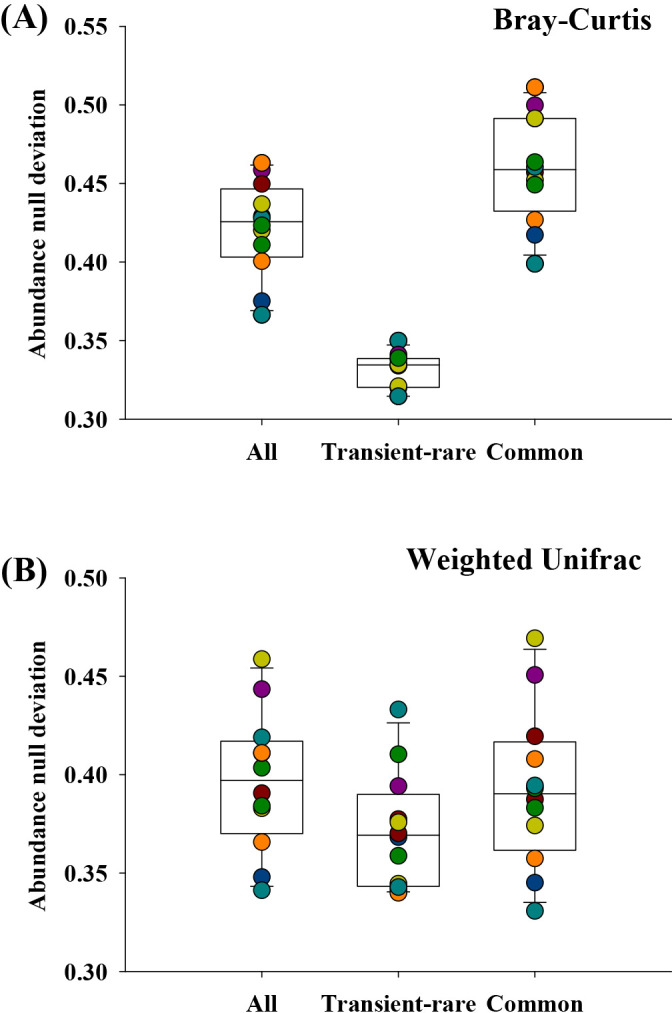
Relative changes in niche and neutral processes assessed based on deviations from abundance-weighted beta-null models. Abundance null deviations of taxon assembly based on (A) Bray–Curtis and (B) weighted UniFrac distances. Colored circles indicate individual samples. In both Bray–Curtis and weighted UniFrac resemblances, the transient-rare and common taxa showed distinct null deviations (*P*<0.05).

Additionally, to understand the deviation of the observed differences in beta-diversity from null expectations, abundance-based beta-null approaches were used to distinguish between the niche and neutral processes, as described by Tucker *et al.* (Tucker *et al.*, 2016) and Lee *et al.* (Lee *et al.*, 2017). In this comparative approach, deviations to and from a permuted null expectation (neutral) were used to interpret the relative contributions of the neutral and niche processes, respectively. Transient-rare communities deviated from the null expectation, with the transient-rare taxa occurring closer to the null expectation than the common taxa (Fig. 4). In addition, the common taxa showed a significantly higher beta-null deviation than the transient-rare taxa (*P*<0.05).

In the present study, the transient-rare taxa were defined as those with an occurrence frequency of <4 and a relative abundance of <1%. However, even minor changes in OTU abundance may change the classification of the transient-rare and common taxa. These noises are frequently observed in the OTUs around the detection limit. When both occurrence frequency and relative abundance conditions were applied together to construct the dataset, the relative abundance of the transient-rare taxa was <0.5% and the occurrence frequency ranged between <3 and <5. Slightly different combinations of the transient-rare and common taxa were observed in this dataset, with a relative abundance of <0.8% and an occurrence frequency ranging between <6 and <7.

In all tested dataset combinations, the transient-rare taxa mostly fit well to the log-series regression curves (red area in Table S6A), except in 10 combinations (blue area in Table S6A). In contrast, the common taxa fit better to the log-normal regression curves than to the log-series regression curves (blue area in Table S6B). The highest log-normal goodness-of-fit score for the common taxa was observed in a dataset with an occurrence frequency of >6 and a relative abundance ranging between >0.075 and 0.8% (*χ^2^*=10.8); however, a relatively higher log-series goodness-of-fit score was obtained for the transient-rare taxa in the same dataset (*χ^2^*=603.8). The lowest goodness-of-fit score (*χ^2^*=89.4) for the transient-rare taxa was obtained in a dataset with an occurrence frequency of >4 and relative abundance of >1%; however, the log-normal goodness-of-fit score for the common taxa (*χ^2^*=32.8) was slightly higher than the lowest value for the transient-rare taxa (*χ^2^*=10.8). Therefore, based on the best results of the chi-squared test on various combinations of the community dataset (occurrence frequency of 1–12 and relative abundance of 0.05–4.00%), we defined the transient-rare taxa as those with an occurrence frequency of <4 and relative abundance of <1%. Collectively, these results suggest that the species abundance distributions of the transient-rare and common taxa did not shift due to sampling artifacts.

## Discussion

Recent advances in high-throughput sequencing technologies, together with increased computational performance, have enabled us to uncover the members of rare biospheres in diverse ecosystems (Sogin *et al.*, 2006; Pedrós-Alió, 2012). Although this has further opened avenues for characterizing and understanding the microbial rare biosphere, how and why numerous species are rare remain unclear (Pedrós-Alió, 2012). Accordingly, the present study demonstrated the contributions of the transient-rare taxa to local community dynamics (in an activated sludge bioreactor). Environmental conditions and operational parameters minimally accounted for the variations observed in the transient-rare taxa in the bioreactor (25.2%), implying that abiotic deterministic factors did not play a prominent role in driving the assembly of transient-rare taxa. The remaining high proportion of the unexplained variation (76.5%) in the transient-rare taxa may be explained by unmeasured deterministic factors, microbial interactions (*e.g.*, viral infection), and neutral factors. However, neutral processes may explain most of the variations in the transient-rare taxa, as evidenced by the Sloan neutral community model (Fig. 3A). Neutral processes describe the occurrence of the transient-rare taxa as the stochastic replacement of open sites (generated by random death or emigration) by other taxa in the inflow or within the bioreactor. These findings indicate that neutral processes are likely to be more important than deterministic ones for the assembly of transient-rare taxa in the bioreactor. Similar findings have been reported in several non-bacterial ecosystems. Based on a study of six rare and six abundant amoeba species in a soil ecosystem, Finlay *et al.* (Finlay *et al.*, 2001) demonstrated that rare species followed a Poisson distribution (*i.e.*, random distribution). In addition, Magurran and Henderson (Magurran and Henderson, 2003) examined an estuarine fish community using a 21-year dataset and demonstrated that the abundance of occasional species was low, and they followed a random distribution in a headland ecosystem. These findings, together with the present results, suggest that the neutral assembly of the transient-rare or occasional species may be a common phenomenon in various organisms and ecosystems.

**Fig. 5.**
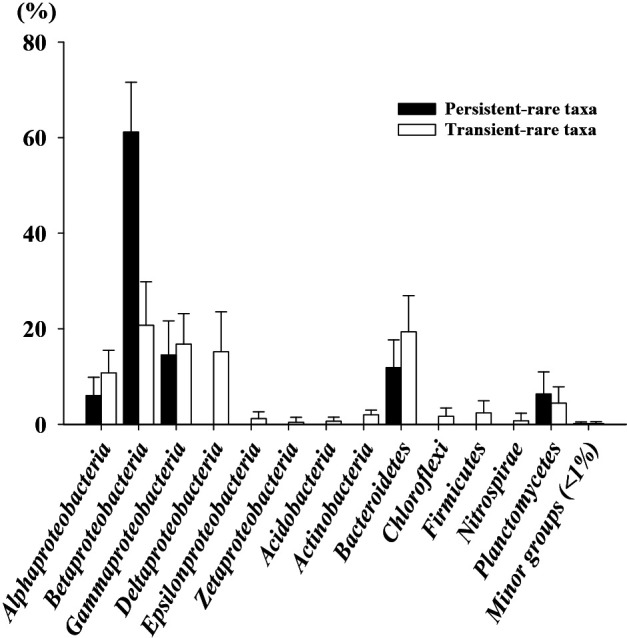
Classification of transient-rare (open bars) and persistent-rare (solid bars) taxa at phylum and proteobacterial class levels. The error bars indicate the SD of 12 samples.

Diverse bacterial species from the influent wastewater and atmosphere continuously arrive in activated sludge bioreactors (Lee *et al.*, 2015), which may affect the bacterial community compositions of these bioreactors. Some bacteria arriving in the bioreactors are selected by deterministic factors and, in turn, these proliferate in the bioreactors, whereas the other bacteria are not selected. The transient-rare taxa arrive in the bioreactors infrequently and at low numbers, but are not selected by deterministic factors. However, it remains unclear which factors (deterministic or neutral) drive the assembly of persistent-rare taxa. Such taxa may be assembled similar to the transient-rare taxa (*i.e.*, neutral assembly), and they may frequently immigrate to the bioreactor, but are not selected, suggesting that their standing populations are supported by continuous dispersal (*e.g.*, the mass effect [Leibold *et al.*, 2004]). Another possibility is that abiotic and/or biotic conditions maintain these taxa in the bioreactor, but grow markedly slower than the abundant taxa (Fuhrman, 2009). We also fit our cohort of persistent-rare taxa to the Sloan neutral community model (Fig. 3A and Table S8). Notably, their percentage was markedly lower than that of the transient-rare taxa (96.4%), suggesting that the second scenario explains the assembly of the persistent-rare taxa. Additionally, we performed beta-null deviation analyses and found that the transient-rare taxa occurred closer to the neutral expectations than the common taxa (Fig. 4). In addition, the common taxa showed significantly higher beta-null deviations than the transient-rare taxa (*P*<0.05). Furthermore, the phylogenetic composition of the transient- and persistent-rare taxa was distinct. The persistent-rare taxa included significantly higher proportions of *Betaproteobacteria* and *Planctomycetes*, but lower proportions of *Alphaproteobacteria*, *Gammaproteobacteria*, and *Bacteroidetes* than the transient-rare ones (Fig. 5). In our simulation test, the persistent-rare taxa with a relative abundance of >1% and an occurrence frequency of >10 showed a better goodness-of-fit for the log-normal distribution than the log-series distribution, indicating that the distribution of the persistent-rare taxa was affected by deterministic factors of the Pohang WWTP (Table S8). Overall, these results suggest that the two rare taxon assemblages differed in terms of their ecological roles in the bioreactor.

**Fig. 6.**
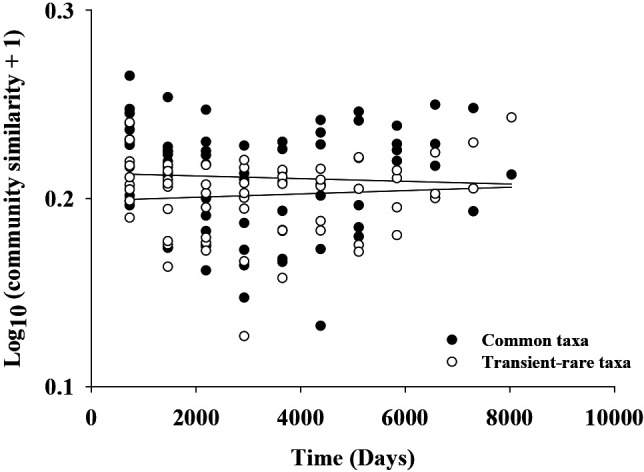
Time-decay relationships for transient-rare and common taxa. Data were fit to a model describing the relationship between decayed richness and time based on the calculation method described by Korhonen *et al.* (2010).

Difficulties are associated with predicting community assembly processes based on ecological patterns because a similar pattern may be generated by interactions among multiple processes (Hanson *et al.*, 2012) and different processes may predict similar patterns (Harpole, 2010). Nevertheless, community assembly processes may be projected in ecological patterns in a certain manner (Fargione *et al.*, 2003). In this regard, different ecological patterns observed between the transient-rare and common taxa (*e.g.*, taxa abundance distribution in Fig. 1) suggest that different community assembly processes acted on these two groups of assemblages. Similar to the present results, the transient-rare and common taxa showed a log-series and log-normal distribution, respectively, in other assemblages, including marine prokaryotes (Galand *et al.*, 2009), estuarine fish (Magurran and Henderson, 2003), and terrestrial insects (Southwood, 1996). However, the patterns of community similarity decay over time (“time-decay” [Korhonen *et al.*, 2010]) were not significant for both cohorts (Fig. 6).

In the present study, the transient-rare taxa were defined as those with an occurrence frequency of <4 and relative abundance of <1%. To confirm the effects of changes in this definition on the OTU classification at the detection limit, a dynamic simulation of 60 different dataset combinations was performed. The simulation results indicated that sampling artifacts were unlikely to shift the species abundance distributions of the transient-rare and common taxa (Table S6). In addition, we evaluated the neutrality of both transient-rare and common taxon assemblages in response to the dynamic OTU abundance and occurrence frequency based on the Sloan neutral community model fit (Table S7). The results obtained showed that even when relative abundance and occurrence frequency were changed, up to 90% OTUs belonging to the transient-rare taxa showed stronger neutrality than the common taxa. Specifically, the transient-rare taxa with an occurrence frequency of 2–5 showed significant neutrality in this analysis, indicating that the overarching patterns remain the same regardless of nuances in the thresholds applied.

Several explanations and conceptual models have been proposed for the roles of rare microbial taxa. The most common explanation is that rare microbial taxa do not actively grow in local communities (Pedrós-Alió, 2012), and, in turn, simply pass through the local systems (Shade *et al.*, 2014). Jones and Lennon (2010) investigated the activity of bacterial taxa (relative recovery of 16S rRNA relative to 16S rDNA) across abundance ranks in two lakes and observed that many rare bacterial taxa showed high relative recovery of 16S rRNA. Based on this observation, they claimed that rare bacterial taxa serve as a seedbank (Lennon and Jones, 2011) and respond to environmental changes. Furthermore, Shade *et al.* (2014) demonstrated that bacterial and archaeal rare taxa occasionally become abundant (conditionally rare taxa) in diverse ecosystems and then contribute to temporal changes in bacterial and archaeal diversity. This analysis included a distinction between taxa that were observed only once and those that were more persistent within a community. In this regard, although the transient-rare taxa defined in the present study had low abundance and were generally insensitive to the available resources in the bioreactor, they may become abundant in response to perturbation or changing conditions in the future. We observed a portion of the taxa within the present study that may be classified as exhibiting “conditional rarity” (2.0%), and all these conditionally rare taxa belonged to the persistent-rare cohort and not to the transient-rare cohort. They were occasionally rare, but remained prevalent during some periods. Notably, the neutral models often fit well to microbial communities (Sloan *et al.*, 2006; Woodcock *et al.*, 2007; Roguet *et al.*, 2015; Venkataraman *et al.*, 2015), which may have been due to the high proportion of transient-rare taxa in microbial communities because of high dispersal (Shade *et al.*, 2014). Nevertheless, the present study suggests that the transient-rare taxa may arguably not contribute to the key functional processes within the community in an activated sludge bioreactor.

It currently remains unclear whether the transient-rare taxa contribute to key functional processes within the bioreactor. However, previous studies suggested that these taxa are important for serving novel or redundant functions when the environment changes (Shade *et al.*, 2014). As a “microbial seedbank”, rare species are important because they contribute to the genetic diversity of the microbial community (Fuhrman, 2009) and potentially contribute to ecological functions if they exhibit blooming dynamics (Fuhrman, 2009; Shade *et al.*, 2014). Accordingly, rare taxa may play pivotal ecological roles in local communities when the environmental conditions in the bioreactor change. The present results demonstrating the importance of neutral processes in the assembly of the transient-rare taxa within a local community deepen our understanding of the various roles and dynamics of members of the microbial rare biosphere. In studies on communities, the transient-rare taxa impart significant noise due to their stochastic behavior instead of interactions with abiotic and biotic factors; however, they have been proposed to be important after awakening from dormancy (Jones and Lennon, 2010), and their neutrality is not considered to be permanent because of their existence at the limit of detection (Shade *et al.*, 2014; Shade and Gilbert, 2015). Therefore, if we remove these taxa from such studies, we may be able to further our understanding of the standing community and better explain deterministic drivers. Furthermore, the present results showing that the relative importance of diverse assembly processes is specific and that the composition of the transient- and persistent-rare taxa is distinct suggest that rare taxa with various occurrence patterns play ecological roles within their local communities (Shade and Gilbert, 2015).

The present results indicate that the community assembly of the transient-rare taxa is highly diverse and mostly affected by stochastic processes in the bioreactor. In contrast, the common taxa show relatively low diversity in an activated sludge bioreactor. Moreover, the common taxa may play important ecological roles in a WWTP because their distribution patterns are affected more by the operational conditions in an activated bioreactor (niche processes); therefore, the common taxa may be used as indicators of performance when evaluating bioreactor function.

In conclusion, microbial communities are dynamic in space and time, and their collective dynamics are underpinned by changes in discrete microbial populations, the relative contributions of which to the community fluctuate. The present study focused on a prominent subset of microbial populations within their community—the transient-rare taxa. Since microbial communities are species-rich, rare taxa often comprise the majority of the total observed taxa within these communities. We found that the neutral patterns of assembly best described the dynamics of these transient-rare taxa, supporting the notion that these taxa are not the contributing members of the community, but are rather driven by stochastic forces. Therefore, the dynamics of the transient-rare taxa may be explained by their patterns of dispersal in regional metacommunities.

## Citation

Lee, S.-H., Kim, T.-S., and Park, H.-D. (2021) Transient-rare Bacterial Taxa Are Assembled Neutrally across Temporal Scales. *Microbes Environ ***36**: ME20110.

https://doi.org/10.1264/jsme2.ME20110

## Supplementary Material

Supplementary Material
