# Supplementary material for: Transient-rare Bacterial Taxa Are Assembled Neutrally across Temporal Scales
Source: Microbes Environ. 2021 Feb 10;36(1):ME20110. doi: 10.1264/jsme2.ME20110 (PMC7966942; doi:10.1264/jsme2.ME20110)
Supplement: Supplementary file 1 — Supplementary Material [file 36_20110_s1.pdf]

**Supplementary material**

**Supplementary table legends**

**Table S1.** Operational and environmental parameters of the Pohang wastewater treatment plant.

**Table S2.** Number of raw and qualified sequences used in this study.

**Table S3**. Statistical significance of the unique environmental components in the redundancy analysis (RDA) and canonical correspondence analysis (CCA).

**Table S4**. Relative distribution of operational taxonomic units and their sequences belonging to each taxon assembly.

**Table S5.** Classification of operational taxonomic unit based on taxonomic affiliation.

**Table S6.** Goodness-of-fit test for the dynamically changed taxa.

**Table S7.** Dynamic neutrality test for the transient-rare and common taxa in various datasets.

**Table S8.** Goodness-of-fit test for the persistent-rare taxa.

**Supplementary figure legends**

**Fig. S1.** Relative operational taxonomic unit (OTU) abundance distribution based on 2,544 unique OTUs at a 3% identity cutoff from 33,401 sequence reads of 12 samples collected bimonthly over 2 years.

**Fig. S2.** Venn diagram of families found in (A) transient-rare, (B) both transient-rare and common, and (C) common taxa.

**Fig. S3.** Seasonal variation in bacterial phyla and proteobacterial classes belonging to (A) transient-rare and (B) common taxa from sludge samples collected bimonthly for 24 months from the Pohang wastewater treatment plant.

**Table S1**. Operational and environmental parameters of the Pohang WWTP

| Characteristic | Number of measurement | Range | Average | Standard deviation |
|---|---|---|---|---|
| Bioreactor temperature ($^{o}$C) | 68 | 8.7 ~ 27.2 | 18.6 | 5.6 |
| Dissolved oxygen (mg/L) | 68 | 0.4 ~ 2.0 | 0.8 | 0.3 |
| pH | 68 | 6.3 ~ 7.6 | 7.0 | 0.3 |
| Hydraulic retention time (HRT) (hr) | 704 | 5.5 ~ 29.9 | 6.2 | 1.0 |
| Solids retention time (SRT) (day) | 67 | 3.9 ~ 22.7 | 8.9 | 3.7 |
| Mixed liquor suspended soilds (MLSS) (mg/L) | 68 | 1,700 ~ 3,560 | 2,640 | 400 |
| Influent $BOD_5$ (mg/L) | 211 | 17.2 ~ 125.2 | 70.2 | 19.9 |
| Effluent $BOD_5$ (mg/L) | 730 | 1.0 ~ 19.0 | 7.5 | 5.0 |
| Influent total nitrogen (mg-N/L) | 212 | 5.6 ~ 43.9 | 19.2 | 5.4 |
| Effluent total nitrogen (mg-N/L) | 730 | 4.4 ~ 19.8 | 11.6 | 2.2 |
| Influent total phosphorus (mg-N/L) | 212 | 0.6 ~ 6.3 | 2.3 | 0.9 |
| Effluent total phosphorus (mg-N/L) | 729 | 0.1 ~ 2.3 | 1.0 | 0.4 |

**Table S2.** Number of raw and qualitied sequences used in this study.

| Sample ID | # of raw sequences | # of chimeric sequences | # of trimmed sequences | # of selected sequences |
|---|---|---|---|---|
| Total | 65,640 | 4,093 | 61,547 | 35,856 |
| Jun.05 | 5,708 | 318 | 5,390 | 2,988 |
| Aug.05 | 6,678 | 502 | 6,176 | 2,988 |
| Oct.05 | 5,031 | 490 | 4,541 | 2,988 |
| Dec.05 | 4,613 | 239 | 4,374 | 2,988 |
| Feb.06 | 3,117 | 129 | 2,988 | 2,988 |
| Apr.06 | 4,856 | 156 | 4,700 | 2,988 |
| Jun.06 | 6,689 | 288 | 6,401 | 2,988 |
| Aug.06 | 6,893 | 446 | 6,447 | 2,988 |
| Oct.06 | 6,596 | 385 | 6,211 | 2,988 |
| Dec.06 | 7,684 | 299 | 7,385 | 2,988 |
| Feb.07 | 3,621 | 495 | 3,126 | 2,988 |
| Apr.07 | 4,154 | 346 | 3,808 | 2,988 |

**Table S3**. Statistical significance of unique environmental components in RDA and CCA analyses.

| Variable | Transient-rare taxa | | Common taxa | |
|---|---|---|---|---|
| | P-value | F-value | P-value | F-value |
| Temperature | **0.001** | **1.190** | **0.043** | **1.330** |
| Solids retention time | 0.482 | 1.070 | 0.352 | 1.020 |
| Biochemical oxygen demand_influent | 0.341 | 0.990 | **0.001** | **1.150** |
| pH | 0.257 | 1.120 | 0.069 | 1.210 |
| Total nitrogen_Influent | 1.000 | 0.000 | 0.315 | 1.480 |
| Total nitrogen_Effluent | 0.086 | 1.070 | 0.344 | 0.970 |
| Mixed liquor suspended solids | 0.552 | 0.960 | **0.044** | **2.070** |
| Biochemical oxygen demand_Effluent | **0.001** | **1.200** | 0.443 | 0.710 |
| Dissolved oxygen | 0.395 | 1.020 | 0.743 | 0.400 |
| Hydraulic retention time | 0.805 | 0.890 | 0.889 | 0.290 |
| Total phosphorus_Influent | 0.220 | 0.930 | 1.000 | 0.000 |
| Total phosphorus_Effluent | 1.000 | 0.000 | 1.000 | 0.000 |

Bolded marks were statistically significant values ($P < 0.05$).

**Table S4**. Relative distribution of OTUs and their sequences belonging to each taxa assembly

| Assembly | Total | | | | Inner ± 95% | | | | Outer ± 95% | | | |
|---|---|---|---|---|---|---|---|---|---|---|---|---|
| | No. of OTUs | % | No. of sequences | % | No. of OTUs | % | No. of sequences | % | No. of OTUs | % | No. of sequences | % |
| Total | 2,544 | 100.0 | 33,401 | 100.0 | 2,304 | 90.6 | 6,838 | 20.5 | 240 | 9.4 | 2,736 | 8.2 |
| Transient-rare taxa | 1,900 | 74.7 | 9,574 | 28.7 | 1,832 | 72.0 | 6,838 | 20.5 | 68 | 2.7 | 2,736 | 8.2 |
| Common taxa | 644 | 25.3 | 23,827 | 71.3 | 472 | 18.6 | 10,855 | 32.5 | 172 | 6.8 | 12,972 | 38.8 |

**Table S5.** Classification of OTUs based on taxonomic affiliation.

| OTUs | Sampling time | | | | | | | | | | | | Taxonomic affiliation | | | | Group |
|---|---|---|---|---|---|---|---|---|---|---|---|---|---|---|---|---|---|
| | Jun.05 | Aug.05 | Oct.05 | Dec.05 | Feb.06 | Apr.06 | Jun.06 | Aug.06 | Oct.06 | Dec.06 | Feb.07 | Apr.07 | Phylum | Class | Family | Order | |
| OTU 1 | 0.06 | 0.00 | 0.00 | 6.72 | 36.41 | 10.03 | 16.30 | 0.00 | 0.00 | 0.00 | 0.11 | 0.05 | *Planctomycetes* | *Planctomycetia* | *Planctomycetales* | *Planctomycetaceae* | Common taxa |
| OTU 2 | 0.00 | 0.00 | 0.00 | 0.32 | 0.69 | 4.86 | 25.70 | 1.04 | 0.19 | 0.00 | 0.00 | 0.00 | *Planctomycetes* | *Planctomycetia* | *Planctomycetales* | *Planctomycetaceae* | Common taxa |
| OTU 5 | 0.00 | 0.00 | 0.19 | 0.82 | 0.32 | 2.76 | 6.37 | 6.55 | 1.18 | 0.12 | 0.00 | 0.05 | *Nitrospirae* | *Nitrospira* | *Nitrospirales* | *Nitrospiraceae* | Common taxa |
| OTU 6 | 0.00 | 0.00 | 0.00 | 0.00 | 0.00 | 0.00 | 0.00 | 0.32 | 0.80 | 4.57 | 5.78 | 7.69 | *Firmicutes* | *Bacilli* | *Bacillales* | *Bacillaceae 1* | Common taxa |
| OTU 7 | 0.62 | 0.63 | 5.51 | 1.23 | 1.01 | 0.56 | 0.70 | 0.41 | 1.04 | 2.99 | 0.98 | 1.76 | *Proteobacteria* | *Alphaproteobacteria* | *Rhodobacterales* | *Rhodobacteraceae* | Common taxa |
| OTU 8 | 0.00 | 0.06 | 0.52 | 15.83 | 0.16 | 0.05 | 0.00 | 0.00 | 0.00 | 0.00 | 0.00 | 0.00 | *Bacteroidetes* | *Flavobacteriia* | *Flavobacteriales* | *Flavobacteriaceae* | Common taxa |
| OTU 9 | 0.00 | 0.00 | 0.38 | 1.95 | 1.06 | 1.02 | 8.83 | 2.35 | 0.71 | 0.12 | 0.00 | 0.00 | *Proteobacteria* | *Gammaproteobacteria* | *Oceanospirillales* | *Oceanospirillaceae* | Common taxa |
| OTU 11 | 9.98 | 10.16 | 0.95 | 0.00 | 0.00 | 0.00 | 0.00 | 0.00 | 0.00 | 0.00 | 0.00 | 0.10 | *Proteobacteria* | *Gammaproteobacteria* | *Chromatiales* | *Chromatiaceae* | Common taxa |
| OTU 12 | 0.43 | 1.55 | 2.47 | 0.73 | 0.42 | 0.61 | 0.88 | 1.76 | 1.28 | 1.37 | 1.53 | 1.96 | *Proteobacteria* | *Betaproteobacteria* | *Rhodocyclales* | *Rhodocyclaceae* | Common taxa |
| OTU 14 | 0.50 | 1.89 | 1.81 | 0.86 | 0.32 | 0.26 | 0.97 | 1.54 | 1.32 | 1.82 | 1.04 | 1.91 | *Proteobacteria* | *Betaproteobacteria* | *Rhodocyclales* | *Rhodocyclaceae* | Common taxa |
| OTU 16 | 0.81 | 1.66 | 1.85 | 1.09 | 0.26 | 0.61 | 0.79 | 1.94 | 1.09 | 0.97 | 0.71 | 1.06 | *Proteobacteria* | *Betaproteobacteria* | *Rhodocyclales* | *Rhodocyclaceae* | Common taxa |
| OTU 17 | 0.00 | 0.00 | 0.33 | 6.99 | 0.74 | 2.92 | 0.22 | 0.09 | 0.61 | 0.12 | 0.00 | 0.00 | *Bacteroidetes* | *Sphingobacteriia* | *Sphingobacteriales* | *Saprospiraceae* | Common taxa |
| OTU 18 | 2.54 | 0.29 | 0.19 | 1.63 | 0.37 | 0.05 | 0.35 | 0.90 | 1.14 | 2.02 | 2.02 | 0.90 | *Proteobacteria* | *Gammaproteobacteria* | *Chromatiales* | *Chromatiaceae* | Common taxa |
| OTU 19 | 0.74 | 0.17 | 2.85 | 1.13 | 0.79 | 0.05 | 0.35 | 0.36 | 0.57 | 0.85 | 2.45 | 1.96 | *Proteobacteria* | *Alphaproteobacteria* | *Rhodobacterales* | *Rhodobacteraceae* | Common taxa |
| OTU 20 | 0.19 | 0.00 | 0.00 | 0.00 | 0.00 | 0.05 | 0.00 | 0.00 | 0.14 | 0.08 | 11.51 | 0.10 | *Proteobacteria* | *Deltaproteobacteria* | *Myxococcales* | *Nannocystineae* | Common taxa |
| OTU 21 | 0.62 | 0.00 | 0.00 | 0.00 | 0.00 | 0.00 | 0.00 | 0.09 | 2.98 | 3.68 | 0.76 | 1.01 | *Acidobacteria* | *Acidobacteria_Gp4* | *Gp4* | | Common taxa |
| OTU 22 | 0.00 | 1.26 | 0.00 | 0.00 | 0.00 | 0.00 | 0.04 | 6.91 | 0.28 | 0.00 | 0.00 | 0.05 | *Proteobacteria* | *Deltaproteobacteria* | *Myxococcales* | *Nannocystineae* | Common taxa |
| OTU 23 | 8.98 | 0.46 | 0.14 | 0.00 | 0.16 | 0.10 | 0.00 | 0.00 | 0.00 | 0.81 | 0.00 | 0.05 | *Bacteroidetes* | *Sphingobacteriia* | *Sphingobacteriales* | *Saprospiraceae* | Common taxa |
| OTU 24 | 0.19 | 1.32 | 1.33 | 0.54 | 0.11 | 0.10 | 0.57 | 1.13 | 0.76 | 0.73 | 0.33 | 0.60 | *Proteobacteria* | *Betaproteobacteria* | *Rhodocyclales* | *Rhodocyclaceae* | Common taxa |
| OTU 25 | 0.19 | 0.23 | 0.14 | 0.95 | 0.42 | 0.56 | 0.26 | 0.14 | 0.14 | 0.77 | 1.96 | 2.01 | *Proteobacteria* | *Betaproteobacteria* | *Rhodocyclales* | *Rhodocyclaceae* | Common taxa |
| OTU 26 | 0.00 | 0.00 | 0.00 | 0.00 | 0.00 | 0.00 | 0.00 | 0.00 | 0.09 | 0.16 | 7.75 | 0.25 | *Proteobacteria* | *Deltaproteobacteria* | *Myxococcales* | *Nannocystineae* | Common taxa |
| OTU 27 | 2.60 | 0.29 | 0.00 | 0.00 | 0.00 | 0.00 | 0.00 | 0.00 | 0.00 | 0.00 | 0.00 | 5.33 | *Proteobacteria* | *Betaproteobacteria* | *Burkholderiales* | *Comamonadaceae* | Common taxa |
| OTU 28 | 0.00 | 0.00 | 0.00 | 0.91 | 5.09 | 1.18 | 0.57 | 0.00 | 0.00 | 0.00 | 0.00 | 0.00 | *Bacteroidetes* | *Flavobacteriia* | *Flavobacteriales* | *Flavobacteriaceae* | Common taxa |
| OTU 29 | 0.00 | 0.00 | 0.05 | 6.40 | 0.11 | 0.00 | 0.00 | 0.00 | 0.00 | 0.00 | 0.00 | 0.00 | *Bacteroidetes* | *Sphingobacteriia* | *Sphingobacteriales* | *Saprospiraceae* | Common taxa |
| OTU 30 | 0.00 | 0.86 | 0.00 | 0.00 | 0.00 | 0.00 | 0.00 | 5.74 | 0.00 | 0.00 | 0.00 | 0.00 | *Proteobacteria* | *Deltaproteobacteria* | *Myxococcales* | *Nannocystineae* | Common taxa |
| OTU 31 | 0.00 | 0.00 | 0.00 | 0.00 | 0.53 | 6.04 | 0.62 | 0.00 | 0.00 | 0.00 | 0.00 | 0.00 | *Proteobacteria* | *Deltaproteobacteria* | *Myxococcales* | *Sorangiineae* | Common taxa |
| OTU 32 | 0.00 | 0.17 | 2.76 | 0.00 | 0.00 | 0.00 | 0.00 | 0.18 | 1.61 | 1.37 | 0.00 | 0.00 | *Proteobacteria* | *Alphaproteobacteria* | *Sphingomonadales* | *Sphingomonadaceae* | Common taxa |
| OTU 34 | 0.00 | 0.00 | 5.46 | 0.09 | 0.00 | 0.00 | 0.22 | 0.23 | 0.05 | 0.00 | 0.00 | 0.00 | *Proteobacteria* | *Gammaproteobacteria* | *Gammaproteobacteria_incertae_sedis* | *Thiohalophilus* | Common taxa |
| OTU 35 | 0.06 | 1.15 | 0.62 | 0.27 | 0.05 | 0.41 | 0.13 | 0.54 | 0.71 | 0.49 | 0.71 | 0.90 | *Proteobacteria* | *Betaproteobacteria* | *Rhodocyclales* | | Common taxa |
| OTU 37 | 0.00 | 0.00 | 0.05 | 2.40 | 0.64 | 0.82 | 0.04 | 0.00 | 0.00 | 1.01 | 0.44 | 0.00 | *Bacteroidetes* | *Sphingobacteriia* | *Sphingobacteriales* | *Saprospiraceae* | Common taxa |
| OTU 38 | 1.80 | 0.00 | 0.00 | 0.00 | 0.00 | 0.00 | 0.00 | 0.00 | 0.00 | 2.34 | 1.15 | 0.10 | *Bacteroidetes* | *Flavobacteriia* | *Flavobacteriales* | *Cryomorphaceae* | Common taxa |
| OTU 39 | 0.00 | 0.00 | 0.10 | 0.41 | 0.48 | 3.99 | 0.22 | 0.18 | 0.09 | 0.00 | 0.00 | 0.00 | *Proteobacteria* | *Deltaproteobacteria* | *Myxococcales* | *Cystobacterineae* | Common taxa |
| OTU 40 | 0.31 | 0.63 | 0.86 | 0.32 | 0.21 | 0.15 | 0.35 | 0.68 | 0.19 | 0.73 | 0.33 | 0.40 | *Proteobacteria* | *Betaproteobacteria* | *Rhodocyclales* | | Common taxa |
| OTU 41 | 0.25 | 0.57 | 1.00 | 0.50 | 0.21 | 0.31 | 0.13 | 0.99 | 0.43 | 0.24 | 0.11 | 0.45 | *Proteobacteria* | *Betaproteobacteria* | *Rhodocyclales* | | Common taxa |
| OTU 42 | 0.00 | 0.75 | 0.05 | 0.00 | 0.00 | 0.00 | 0.00 | 4.02 | 0.14 | 0.00 | 0.00 | 0.00 | *Proteobacteria* | *Deltaproteobacteria* | *Myxococcales* | *Nannocystineae* | Common taxa |
| OTU 43 | 0.12 | 0.57 | 0.76 | 0.50 | 0.21 | 0.15 | 0.40 | 0.68 | 0.47 | 0.40 | 0.16 | 0.65 | *Proteobacteria* | *Betaproteobacteria* | *Rhodocyclales* | | Common taxa |
| OTU 46 | 0.12 | 0.11 | 0.05 | 0.36 | 0.42 | 0.36 | 0.26 | 0.32 | 0.28 | 0.24 | 1.15 | 1.51 | *Proteobacteria* | *Betaproteobacteria* | *Rhodocyclales* | *Rhodocyclaceae* | Common taxa |
| OTU 47 | 0.06 | 1.89 | 0.67 | 0.00 | 0.00 | 0.10 | 0.00 | 0.00 | 1.75 | 0.49 | 0.05 | 0.15 | *Proteobacteria* | *Gammaproteobacteria* | *Xanthomonadales* | *Xanthomonadaceae* | Common taxa |
| OTU 50 | 0.00 | 0.00 | 0.00 | 0.05 | 0.11 | 0.36 | 0.00 | 3.70 | 0.38 | 0.00 | 0.00 | 0.00 | *Bacteroidetes* | *Sphingobacteriia* | *Sphingobacteriales* | *Saprospiraceae* | Common taxa |
| OTU 51 | 0.12 | 1.72 | 0.05 | 0.09 | 0.53 | 0.51 | 0.09 | 0.05 | 0.99 | 0.24 | 0.27 | 0.45 | *Bacteroidetes* | *Cytophagia* | *Cytophagales* | *Cytophagaceae* | Common taxa |
| OTU 52 | 0.19 | 0.40 | 0.71 | 0.32 | 0.37 | 0.20 | 0.26 | 0.72 | 0.28 | 0.44 | 0.38 | 0.50 | *Proteobacteria* | *Betaproteobacteria* | *Rhodocyclales* | *Rhodocyclaceae* | Common taxa |
| OTU 57 | 0.00 | 0.23 | 0.62 | 2.13 | 0.00 | 0.00 | 0.00 | 0.68 | 0.57 | 0.00 | 0.00 | 0.00 | *Proteobacteria* | *Alphaproteobacteria* | *Rhodobacterales* | *Rhodobacteraceae* | Common taxa |
| OTU 59 | 0.12 | 0.75 | 1.33 | 0.14 | 0.21 | 0.05 | 0.22 | 0.32 | 0.90 | 0.04 | 0.27 | 0.10 | *Proteobacteria* | *Betaproteobacteria* | *Rhodocyclales* | *Rhodocyclaceae* | Common taxa |
| OTU 60 | 0.99 | 0.00 | 0.00 | 0.00 | 0.42 | 0.77 | 0.00 | 0.00 | 0.57 | 0.85 | 0.38 | 0.30 | *Bacteroidetes* | *Cytophagia* | *Cytophagales* | *Cytophagaceae* | Common taxa |
| OTU 62 | 1.12 | 0.00 | 0.05 | 0.00 | 0.00 | 0.00 | 0.00 | 0.00 | 0.00 | 0.49 | 0.65 | 2.01 | *Bacteroidetes* | *Flavobacteriia* | *Flavobacteriales* | *Flavobacteriaceae* | Common taxa |
| OTU 63 | 0.00 | 2.81 | 0.29 | 0.00 | 0.00 | 0.00 | 0.00 | 0.00 | 0.05 | 0.93 | 0.05 | 0.10 | *Bacteroidetes* | *Cytophagia* | *Cytophagales* | *Flammeovirgaceae* | Common taxa |
| OTU 65 | 0.00 | 0.00 | 0.00 | 0.95 | 0.85 | 1.99 | 0.22 | 0.00 | 0.00 | 0.00 | 0.00 | 0.00 | *Bacteroidetes* | *Cytophagia* | *Cytophagales* | *Flammeovirgaceae* | Common taxa |
| OTU 67 | 0.00 | 0.00 | 0.00 | 0.05 | 0.05 | 1.23 | 0.26 | 0.77 | 0.95 | 0.40 | 0.00 | 0.00 | *Lentisphaerae* | *Lentisphaeria* | *Victivallales* | *Victivallaceae* | Common taxa |
| OTU 69 | 0.00 | 0.29 | 0.14 | 0.00 | 0.00 | 0.00 | 0.00 | 1.99 | 1.04 | 0.16 | 0.00 | 0.00 | *Chloroflexi* | *Anaerolineae* | *Anaerolineales* | *Anaerolineaceae* | Common taxa |
| OTU 72 | 0.00 | 0.34 | 0.10 | 0.00 | 0.00 | 0.00 | 0.00 | 2.08 | 0.95 | 0.08 | 0.00 | 0.00 | *Chloroflexi* | *Anaerolineae* | *Anaerolineales* | *Anaerolineaceae* | Common taxa |
| OTU 73 | 0.19 | 0.34 | 0.38 | 0.32 | 0.11 | 0.05 | 0.31 | 0.45 | 0.47 | 0.28 | 0.16 | 0.50 | *Proteobacteria* | *Betaproteobacteria* | *Rhodocyclales* | *Rhodocyclaceae* | Common taxa |
| OTU 76 | 0.00 | 0.17 | 0.00 | 0.00 | 0.05 | 0.00 | 0.00 | 0.05 | 2.13 | 0.69 | 0.00 | 0.10 | *Bacteroidetes* | *Sphingobacteriia* | *Sphingobacteriales* | *Chitinophagaceae* | Common taxa |
| OTU 77 | 0.12 | 0.06 | 0.00 | 0.00 | 0.00 | 0.10 | 0.00 | 0.00 | 0.09 | 0.00 | 2.89 | 0.45 | *Proteobacteria* | *Deltaproteobacteria* | *Myxococcales* | *Sorangiineae* | Common taxa |
| OTU 78 | 0.00 | 0.00 | 0.19 | 1.23 | 0.42 | 1.13 | 0.31 | 0.00 | 0.00 | 0.00 | 0.00 | 0.00 | *Proteobacteria* | *Deltaproteobacteria* | *Desulfobacterales* | *Desulfobacteraceae* | Common taxa |
| OTU 79 | 0.00 | 0.06 | 0.29 | 0.00 | 0.00 | 0.00 | 0.00 | 0.41 | 1.80 | 0.44 | 0.00 | 0.10 | *Acidobacteria* | *Acidobacteria_Gp4* | *Gp4* | | Common taxa |
| OTU 80 | 0.87 | 0.06 | 0.19 | 0.09 | 0.11 | 0.31 | 0.04 | 0.14 | 0.28 | 0.08 | 0.60 | 0.75 | *Proteobacteria* | *Betaproteobacteria* | *Rhodocyclales* | *Rhodocyclaceae* | Common taxa |
| OTU 81 | 0.00 | 0.06 | 0.10 | 0.27 | 0.42 | 1.07 | 1.19 | 0.00 | 0.00 | 0.00 | 0.00 | 0.00 | *Proteobacteria* | *Betaproteobacteria* | *Rhodocyclales* | *Rhodocyclaceae* | Common taxa |
| OTU 83 | 0.19 | 0.17 | 0.00 | 0.00 | 0.05 | 0.00 | 0.00 | 0.00 | 0.61 | 1.70 | 0.16 | 0.00 | *Proteobacteria* | *Betaproteobacteria* | *Burkholderiales* | *Comamonadaceae* | Common taxa |
| OTU 85 | 0.00 | 0.17 | 2.09 | 0.00 | 0.05 | 0.00 | 0.00 | 0.00 | 0.43 | 0.08 | 0.00 | 0.20 | *Nitrospirae* | *Nitrospira* | *Nitrospirales* | *Nitrospiraceae* | Common taxa |
| OTU 87 | 0.31 | 0.06 | 0.00 | 0.00 | 0.05 | 0.05 | 0.00 | 0.00 | 0.71 | 1.54 | 0.00 | 0.00 | *Proteobacteria* | *Gammaproteobacteria* | *Xanthomonadales* | *Xanthomonadaceae* | Common taxa |
| OTU 89 | 0.00 | 0.00 | 1.09 | 0.00 | 0.64 | 0.20 | 0.00 | 0.90 | 0.00 | 0.00 | 0.00 | 0.00 | *Proteobacteria* | *Deltaproteobacteria* | *Myxococcales* | *Nannocystineae* | Common taxa |
| OTU 91 | 0.00 | 0.00 | 0.14 | 0.82 | 1.17 | 0.51 | 0.18 | 0.05 | 0.00 | 0.00 | 0.00 | 0.00 | *Proteobacteria* | *Betaproteobacteria* | *Burkholderiales* | *Comamonadaceae* | Common taxa |
| OTU 97 | 0.00 | 0.40 | 0.62 | 0.14 | 0.00 | 0.00 | 0.00 | 0.14 | 0.47 | 0.77 | 0.00 | 0.00 | *Bacteroidetes* | *Sphingobacteriia* | *Sphingobacteriales* | *Chitinophagaceae* | Common taxa |
| OTU 102 | 0.00 | 0.00 | 0.05 | 0.91 | 0.58 | 0.51 | 0.48 | 0.05 | 0.00 | 0.00 | 0.00 | 0.00 | *Planctomycetes* | *Phycisphaerae* | *Phycisphaerales* | *Phycisphaeraceae* | Common taxa |
| OTU 104 | 0.06 | 2.12 | 0.00 | 0.00 | 0.00 | 0.00 | 0.00 | 0.00 | 0.05 | 0.53 | 0.00 | 0.00 | *Bacteroidetes* | *Cytophagia* | *Cytophagales* | *Flammeovirgaceae* | Common taxa |
| OTU 106 | 0.25 | 0.00 | 0.00 | 0.00 | 0.00 | 0.00 | 0.00 | 0.00 | 0.00 | 0.81 | 0.65 | 0.80 | *Bacteroidetes* | *Flavobacteriia* | *Flavobacteriales* | *Flavobacteriaceae* | Common taxa |
| OTU 107 | 0.00 | 0.00 | 0.14 | 0.27 | 0.00 | 0.20 | 0.62 | 0.95 | 0.14 | 0.00 | 0.00 | 0.00 | *Nitrospirae* | *Nitrospira* | *Nitrospirales* | *Nitrospiraceae* | Common taxa |
| OTU 108 | 0.00 | 0.00 | 1.52 | 0.54 | 0.11 | 0.00 | 0.18 | 0.05 | 0.00 | 0.00 | 0.00 | 0.00 | *Planctomycetes* | *Phycisphaerae* | *Phycisphaerales* | *Phycisphaeraceae* | Common taxa |
| OTU 111 | 0.00 | 0.52 | 0.90 | 0.09 | 0.11 | 0.41 | 0.44 | 0.00 | 0.00 | 0.00 | 0.00 | 0.00 | *Proteobacteria* | *Deltaproteobacteria* | *Myxococcales* | *Nannocystineae* | Common taxa |
| OTU 114 | 0.00 | 0.00 | 0.00 | 0.05 | 0.00 | 0.00 | 0.00 | 0.00 | 0.80 | 1.01 | 0.11 | 0.20 | *Bacteroidetes* | *Cytophagia* | *Cytophagales* | *Cytophagaceae* | Common taxa |
| OTU 116 | 0.00 | 0.00 | 0.24 | 0.00 | 0.48 | 0.05 | 1.41 | 0.00 | 0.00 | 0.00 | 0.00 | 0.00 | *Proteobacteria* | *Gammaproteobacteria* | *Chromatiales* | *Ectothiorhodospiraceae* | Common taxa |
| OTU 117 | 0.00 | 0.00 | 0.00 | 0.14 | 0.90 | 0.66 | 0.62 | 0.00 | 0.00 | 0.00 | 0.00 | 0.00 | *Bacteroidetes* | *Flavobacteriia* | *Flavobacteriales* | *Flavobacteriaceae* | Common taxa |
| OTU 120 | 0.06 | 0.00 | 0.00 | 0.00 | 0.00 | 0.00 | 0.00 | 0.00 | 0.19 | 0.00 | 2.18 | 0.10 | *Proteobacteria* | *Deltaproteobacteria* | *Myxococcales* | *Sorangiineae* | Common taxa |
| OTU 122 | 0.43 | 1.26 | 0.00 | 0.00 | 0.00 | 0.00 | 0.00 | 0.00 | 0.00 | 0.12 | 0.11 | 0.60 | *Bacteroidetes* | *Flavobacteriia* | *Flavobacteriales* | *Flavobacteriaceae* | Common taxa |
| OTU 126 | 0.93 | 0.06 | 0.10 | 0.09 | 0.11 | 0.10 | 0.04 | 0.05 | 0.28 | 0.16 | 0.05 | 0.30 | *Proteobacteria* | *Betaproteobacteria* | *Rhodocyclales* | *Rhodocyclaceae* | Common taxa |
| OTU 129 | 0.00 | 0.06 | 0.52 | 0.00 | 0.00 | 0.00 | 0.00 | 0.54 | 0.57 | 0.28 | 0.00 | 0.00 | *Proteobacteria* | *Betaproteobacteria* | *Rhodocyclales* | *Rhodocyclaceae* | Common taxa |
| OTU 133 | 0.00 | 0.00 | 0.00 | 0.09 | 1.06 | 0.51 | 0.40 | 0.00 | 0.00 | 0.00 | 0.00 | 0.00 | *Bacteroidetes* | *Flavobacteriia* | *Flavobacteriales* | *Flavobacteriaceae* | Common taxa |
| OTU 143 | 0.00 | 0.00 | 0.00 | 0.05 | 0.00 | 0.05 | 0.00 | 0.00 | 0.00 | 1.50 | 0.00 | 0.05 | *Firmicutes* | *Clostridia* | *Clostridiales* | *Clostridiales_Incertae Sedis XII* | Common taxa |
| OTU 147 | 0.00 | 0.00 | 0.00 | 1.32 | 0.05 | 0.15 | 0.26 | 0.00 | 0.00 | 0.00 | 0.00 | 0.00 | *Proteobacteria* | *Deltaproteobacteria* | *Myxococcales* | *Nannocystineae* | Common taxa |
| OTU 149 | 0.06 | 0.17 | 0.29 | 0.05 | 0.11 | 0.10 | 0.00 | 0.14 | 0.24 | 0.24 | 0.22 | 0.25 | *Proteobacteria* | *Betaproteobacteria* | *Rhodocyclales* | *Rhodocyclaceae* | Common taxa |
| OTU 151 | 0.00 | 0.06 | 0.14 | 0.05 | 0.16 | 0.41 | 0.00 | 0.54 | 0.28 | 0.12 | 0.00 | 0.05 | *Lentisphaerae* | *Lentisphaeria* | *Lentisphaerales* | *Lentisphaeraceae* | Common taxa |
| OTU 152 | 0.74 | 1.21 | 0.00 | 0.00 | 0.00 | 0.00 | 0.00 | 0.00 | 0.00 | 0.04 | 0.00 | 0.20 | *Proteobacteria* | *Betaproteobacteria* | *Rhodocyclales* | *Rhodocyclaceae* | Common taxa |
| OTU 154 | 0.19 | 0.06 | 0.00 | 0.00 | 0.00 | 0.00 | 0.00 | 0.00 | 0.47 | 0.97 | 0.00 | 0.00 | *Proteobacteria* | *Gammaproteobacteria* | *Xanthomonadales* | *Xanthomonadaceae* | Common taxa |
| OTU 157 | 0.00 | 0.00 | 0.14 | 0.00 | 0.05 | 0.46 | 1.05 | 0.00 | 0.00 | 0.00 | 0.00 | 0.00 | *Proteobacteria* | *Deltaproteobacteria* | *Myxococcales* | *Nannocystineae* | Common taxa |
| OTU 158 | 0.06 | 0.00 | 0.00 | 0.00 | 0.69 | 0.00 | 0.00 | 0.00 | 0.28 | 0.00 | 0.93 | 0.00 | *Proteobacteria* | *Alphaproteobacteria* | *Rickettsiales* | *Rickettsiaceae* | Common taxa |
| OTU 165 | 0.12 | 0.52 | 0.00 | 0.00 | 0.00 | 0.00 | 0.00 | 0.00 | 0.00 | 0.00 | 0.05 | 1.21 | *Proteobacteria* | *Betaproteobacteria* | *Rhodocyclales* | *Rhodocyclaceae* | Common taxa |
| OTU 172 | 0.19 | 0.17 | 0.29 | 0.09 | 0.05 | 0.10 | 0.00 | 0.05 | 0.28 | 0.28 | 0.05 | 0.10 | *Proteobacteria* | *Betaproteobacteria* | *Burkholderiales* | *Comamonadaceae* | Common taxa |
| OTU 173 | 0.00 | 0.06 | 0.00 | 0.00 | 0.00 | 0.00 | 0.00 | 0.14 | 1.32 | 0.04 | 0.00 | 0.00 | *Bacteroidetes* | *Sphingobacteriia* | *Sphingobacteriales* | *Saprospiraceae* | Common taxa |
| OTU 177 | 0.00 | 0.00 | 0.00 | 0.09 | 0.53 | 0.26 | 0.00 | 0.00 | 0.00 | 0.00 | 0.87 | 0.00 | *Firmicutes* | *Bacilli* | *Lactobacillales* | *Carnobacteriaceae* | Common taxa |
| OTU 186 | 0.43 | 0.06 | 0.19 | 0.09 | 0.11 | 0.15 | 0.00 | 0.05 | 0.19 | 0.16 | 0.00 | 0.15 | *Proteobacteria* | *Betaproteobacteria* | *Burkholderiales* | *Burkholderiales_incertae_sedis* | Common taxa |
| OTU 188 | 0.00 | 0.00 | 0.00 | 0.45 | 0.00 | 0.05 | 0.09 | 0.81 | 0.00 | 0.00 | 0.00 | 0.00 | *Bacteroidetes* | *Flavobacteriia* | *Flavobacteriales* | *Flavobacteriaceae* | Common taxa |
| OTU 189 | 0.00 | 0.00 | 0.00 | 0.27 | 1.22 | 0.00 | 0.04 | 0.00 | 0.00 | 0.04 | 0.00 | 0.00 | *Proteobacteria* | *Alphaproteobacteria* | *Rhodobacterales* | *Rhodobacteraceae* | Common taxa |
| OTU 196 | 0.25 | 0.06 | 0.00 | 0.09 | 0.11 | 0.05 | 0.00 | 0.05 | 0.05 | 0.08 | 0.16 | 0.65 | *Proteobacteria* | *Betaproteobacteria* | *Rhodocyclales* | *Rhodocyclaceae* | Common taxa |
| OTU 200 | 0.12 | 0.34 | 0.00 | 0.09 | 0.00 | 0.10 | 0.22 | 0.09 | 0.05 | 0.16 | 0.05 | 0.20 | *Proteobacteria* | *Betaproteobacteria* | *Rhodocyclales* | *Rhodocyclaceae* | Common taxa |
| OTU 219 | 0.31 | 0.06 | 0.05 | 0.09 | 0.00 | 0.15 | 0.04 | 0.05 | 0.52 | 0.00 | 0.05 | 0.05 | *Proteobacteria* | *Betaproteobacteria* | *Rhodocyclales* | *Rhodocyclaceae* | Common taxa |
| OTU 234 | 0.25 | 0.06 | 0.05 | 0.05 | 0.00 | 0.05 | 0.00 | 0.14 | 0.28 | 0.16 | 0.11 | 0.15 | *Proteobacteria* | *Gammaproteobacteria* | *Pseudomonadales* | *Moraxellaceae* | Common taxa |
| OTU 240 | 0.25 | 0.23 | 0.05 | 0.00 | 0.11 | 0.05 | 0.04 | 0.18 | 0.09 | 0.08 | 0.00 | 0.20 | *Proteobacteria* | *Betaproteobacteria* | *Rhodocyclales* | *Rhodocyclaceae* | Common taxa |
| OTU 250 | 0.00 | 0.17 | 0.24 | 0.14 | 0.11 | 0.15 | 0.04 | 0.05 | 0.14 | 0.04 | 0.00 | 0.10 | *Proteobacteria* | *Alphaproteobacteria* | *Sphingomonadales* | *Erythrobacteraceae* | Common taxa |
| OTU 251 | 0.00 | 0.06 | 0.14 | 0.09 | 0.32 | 0.20 | 0.18 | 0.09 | 0.00 | 0.04 | 0.00 | 0.05 | *Planctomycetes* | *Planctomycetia* | *Planctomycetales* | *Planctomycetaceae* | Common taxa |
| OTU 257 | 0.00 | 0.06 | 0.19 | 0.05 | 0.16 | 0.00 | 0.00 | 0.05 | 0.05 | 0.28 | 0.22 | 0.10 | *Proteobacteria* | *Betaproteobacteria* | *Burkholderiales* | *Burkholderiales_incertae_sedis* | Common taxa |
| OTU 265 | 0.06 | 0.17 | 0.14 | 0.00 | 0.16 | 0.15 | 0.00 | 0.09 | 0.09 | 0.16 | 0.00 | 0.10 | *Proteobacteria* | *Betaproteobacteria* | *Burkholderiales* | *Comamonadaceae* | Common taxa |
| OTU 269 | 0.06 | 0.06 | 0.05 | 0.05 | 0.11 | 0.20 | 0.00 | 0.09 | 0.05 | 0.16 | 0.16 | 0.15 | *Proteobacteria* | *Betaproteobacteria* | *Rhodocyclales* | *Rhodocyclaceae* | Common taxa |
| OTU 273 | 0.00 | 0.06 | 0.05 | 0.05 | 0.11 | 0.26 | 0.09 | 0.18 | 0.09 | 0.20 | 0.00 | 0.00 | *Planctomycetes* | *Planctomycetia* | *Planctomycetales* | *Planctomycetaceae* | Common taxa |
| OTU 288 | 0.06 | 0.00 | 0.14 | 0.14 | 0.16 | 0.26 | 0.09 | 0.00 | 0.05 | 0.04 | 0.00 | 0.15 | *Proteobacteria* | *Betaproteobacteria* | *Burkholderiales* | *Comamonadaceae* | Common taxa |
| OTU 317 | 0.00 | 0.11 | 0.00 | 0.09 | 0.05 | 0.20 | 0.04 | 0.00 | 0.24 | 0.12 | 0.05 | 0.05 | *Proteobacteria* | *Betaproteobacteria* | *Rhodocyclales* | *Rhodocyclaceae* | Common taxa |
| OTU 345 | 0.12 | 0.17 | 0.05 | 0.00 | 0.00 | 0.00 | 0.09 | 0.09 | 0.19 | 0.12 | 0.00 | 0.05 | *Proteobacteria* | *Alphaproteobacteria* | *Rhodobacterales* | *Rhodobacteraceae* | Common taxa |
| OTU 347 | 0.25 | 0.11 | 0.10 | 0.05 | 0.00 | 0.00 | 0.09 | 0.18 | 0.09 | 0.04 | 0.00 | 0.00 | *Bacteroidetes* | *Sphingobacteriia* | *Sphingobacteriales* | *Saprospiraceae* | Common taxa |
| OTU 351 | 0.50 | 0.06 | 0.00 | 0.00 | 0.05 | 0.05 | 0.04 | 0.09 | 0.14 | 0.04 | 0.00 | 0.00 | *Proteobacteria* | *Gammaproteobacteria* | *Chromatiales* | *Chromatiaceae* | Common taxa |
| OTU 361 | 0.25 | 0.29 | 0.14 | 0.05 | 0.00 | 0.00 | 0.00 | 0.00 | 0.05 | 0.04 | 0.05 | 0.05 | *Proteobacteria* | *Gammaproteobacteria* | *Gammaproteobacteria_incertae_sedis* | *Simiduia* | Common taxa |
| OTU 367 | 0.06 | 0.11 | 0.24 | 0.05 | 0.05 | 0.00 | 0.00 | 0.05 | 0.00 | 0.16 | 0.05 | 0.05 | *Proteobacteria* | *Alphaproteobacteria* | *Sphingomonadales* | *Sphingomonadaceae* | Common taxa |
| OTU 369 | 0.19 | 0.00 | 0.14 | 0.00 | 0.05 | 0.00 | 0.04 | 0.00 | 0.05 | 0.16 | 0.16 | 0.05 | *Proteobacteria* | *Gammaproteobacteria* | *Pseudomonadales* | *Moraxellaceae* | Common taxa |
| OTU 370 | 0.06 | 0.06 | 0.14 | 0.05 | 0.11 | 0.00 | 0.04 | 0.05 | 0.05 | 0.04 | 0.22 | 0.05 | *Proteobacteria* | *Betaproteobacteria* | *Rhodocyclales* | *Rhodocyclaceae* | Common taxa |
| OTU 379 | 0.25 | 0.00 | 0.00 | 0.05 | 0.11 | 0.05 | 0.00 | 0.05 | 0.00 | 0.08 | 0.22 | 0.10 | *Proteobacteria* | *Betaproteobacteria* | *Rhodocyclales* | *Rhodocyclaceae* | Common taxa |
| OTU 392 | 0.00 | 0.11 | 0.05 | 0.05 | 0.05 | 0.00 | 0.00 | 0.14 | 0.14 | 0.00 | 0.05 | 0.20 | *Proteobacteria* | *Betaproteobacteria* | *Rhodocyclales* | *Rhodocyclaceae* | Common taxa |
| OTU 398 | 0.00 | 0.40 | 0.10 | 0.05 | 0.05 | 0.00 | 0.00 | 0.05 | 0.09 | 0.04 | 0.00 | 0.05 | *Planctomycetes* | *Planctomycetia* | *Planctomycetales* | *Planctomycetaceae* | Common taxa |
| OTU 401 | 0.12 | 0.06 | 0.05 | 0.00 | 0.00 | 0.05 | 0.00 | 0.00 | 0.05 | 0.04 | 0.16 | 0.30 | *Proteobacteria* | *Betaproteobacteria* | *Rhodocyclales* | *Rhodocyclaceae* | Common taxa |
| OTU 410 | 0.00 | 0.06 | 0.00 | 0.14 | 0.05 | 0.05 | 0.04 | 0.05 | 0.00 | 0.00 | 0.22 | 0.15 | *Proteobacteria* | *Betaproteobacteria* | *Rhodocyclales* | *Rhodocyclaceae* | Common taxa |
| OTU 411 | 0.06 | 0.23 | 0.05 | 0.05 | 0.00 | 0.05 | 0.04 | 0.00 | 0.14 | 0.00 | 0.00 | 0.15 | *Proteobacteria* | *Betaproteobacteria* | *Rhodocyclales* | *Rhodocyclaceae* | Common taxa |
| OTU 429 | 0.06 | 0.17 | 0.05 | 0.00 | 0.05 | 0.00 | 0.00 | 0.09 | 0.09 | 0.04 | 0.00 | 0.15 | *Proteobacteria* | *Betaproteobacteria* | *Rhodocyclales* | *Rhodocyclaceae* | Common taxa |
| OTU 431 | 0.25 | 0.11 | 0.00 | 0.00 | 0.00 | 0.05 | 0.04 | 0.05 | 0.09 | 0.04 | 0.00 | 0.10 | *Proteobacteria* | *Betaproteobacteria* | *Rhodocyclales* | *Rhodocyclaceae* | Common taxa |
| OTU 434 | 0.00 | 0.06 | 0.19 | 0.00 | 0.11 | 0.05 | 0.00 | 0.05 | 0.00 | 0.08 | 0.00 | 0.15 | *Proteobacteria* | *Deltaproteobacteria* | *Myxococcales* | *Cystobacterineae* | Common taxa |
| OTU 445 | 0.19 | 0.29 | 0.05 | 0.00 | 0.00 | 0.05 | 0.00 | 0.09 | 0.05 | 0.04 | 0.00 | 0.00 | *Proteobacteria* | *Alphaproteobacteria* | *Sphingomonadales* | *Sphingomonadaceae* | Common taxa |
| OTU 455 | 0.06 | 0.00 | 0.05 | 0.05 | 0.11 | 0.00 | 0.22 | 0.00 | 0.05 | 0.04 | 0.00 | 0.05 | *Proteobacteria* | *Alphaproteobacteria* | *Caulobacterales* | *Caulobacteraceae* | Common taxa |
| OTU 457 | 0.31 | 0.06 | 0.00 | 0.00 | 0.05 | 0.05 | 0.00 | 0.00 | 0.05 | 0.04 | 0.05 | 0.10 | *Proteobacteria* | *Betaproteobacteria* | *Burkholderiales* | *Alcaligenaceae* | Common taxa |
| OTU 462 | 0.06 | 0.11 | 0.05 | 0.05 | 0.05 | 0.00 | 0.09 | 0.09 | 0.00 | 0.04 | 0.11 | 0.00 | *Proteobacteria* | *Betaproteobacteria* | *Rhodocyclales* | *Rhodocyclaceae* | Common taxa |
| OTU 463 | 0.06 | 0.00 | 0.05 | 0.00 | 0.00 | 0.00 | 0.09 | 0.14 | 0.14 | 0.04 | 0.00 | 0.10 | *Proteobacteria* | *Alphaproteobacteria* | *Caulobacterales* | *Hyphomonadaceae* | Common taxa |
| OTU 488 | 0.06 | 0.00 | 0.05 | 0.18 | 0.05 | 0.00 | 0.00 | 0.00 | 0.09 | 0.04 | 0.05 | 0.05 | *Proteobacteria* | *Zetaproteobacteria* | *Mariprofundales* | *Mariprofundaceae* | Common taxa |
| OTU 507 | 0.00 | 0.11 | 0.05 | 0.00 | 0.00 | 0.00 | 0.04 | 0.05 | 0.14 | 0.08 | 0.05 | 0.05 | *Proteobacteria* | *Betaproteobacteria* | *Rhodocyclales* | *Rhodocyclaceae* | Common taxa |
| OTU 518 | 0.06 | 0.00 | 0.05 | 0.00 | 0.11 | 0.05 | 0.00 | 0.05 | 0.09 | 0.08 | 0.00 | 0.10 | *Proteobacteria* | *Alphaproteobacteria* | *Sphingomonadales* | *Sphingomonadaceae* | Common taxa |
| OTU 519 | 0.06 | 0.11 | 0.14 | 0.00 | 0.00 | 0.05 | 0.00 | 0.05 | 0.05 | 0.04 | 0.00 | 0.10 | *Proteobacteria* | *Alphaproteobacteria* | *Rhizobiales* | *Hyphomicrobiaceae* | Common taxa |
| OTU 521 | 0.00 | 0.06 | 0.05 | 0.14 | 0.00 | 0.15 | 0.00 | 0.05 | 0.00 | 0.04 | 0.05 | 0.05 | *Proteobacteria* | *Betaproteobacteria* | *Burkholderiales* | *Comamonadaceae* | Common taxa |
| OTU 540 | 0.00 | 0.17 | 0.05 | 0.14 | 0.05 | 0.00 | 0.04 | 0.05 | 0.00 | 0.00 | 0.00 | 0.05 | *Planctomycetes* | *Phycisphaerae* | *Phycisphaerales* | *Phycisphaeraceae* | Common taxa |
| OTU 554 | 0.12 | 0.06 | 0.00 | 0.09 | 0.00 | 0.00 | 0.00 | 0.09 | 0.05 | 0.04 | 0.00 | 0.10 | *Proteobacteria* | *Betaproteobacteria* | *Rhodocyclales* | *Rhodocyclaceae* | Common taxa |
| OTU 562 | 0.06 | 0.06 | 0.10 | 0.00 | 0.11 | 0.00 | 0.00 | 0.05 | 0.09 | 0.00 | 0.05 | 0.00 | *Proteobacteria* | *Betaproteobacteria* | *Rhodocyclales* | *Rhodocyclaceae* | Common taxa |
| OTU 565 | 0.00 | 0.00 | 0.05 | 0.00 | 0.05 | 0.05 | 0.13 | 0.09 | 0.05 | 0.04 | 0.00 | 0.00 | *Proteobacteria* | *Alphaproteobacteria* | *Rhodospirillales* | *Rhodospirillaceae* | Common taxa |
| OTU 573 | 0.00 | 0.00 | 0.05 | 0.00 | 0.05 | 0.05 | 0.00 | 0.09 | 0.09 | 0.08 | 0.00 | 0.05 | *Bacteroidetes* | *Sphingobacteriia* | *Sphingobacteriales* | *Chitinophagaceae* | Common taxa |
| OTU 582 | 0.00 | 0.06 | 0.05 | 0.00 | 0.11 | 0.00 | 0.09 | 0.05 | 0.05 | 0.04 | 0.00 | 0.05 | *Proteobacteria* | *Betaproteobacteria* | *Burkholderiales* | *Burkholderiales_incertae_sedis* | Common taxa |

| | | | | | | | | | | | | | | | | | |
|---|---|---|---|---|---|---|---|---|---|---|---|---|---|---|---|---|---|
| OTU 590 | 0.06 | 0.11 | 0.05 | 0.00 | 0.05 | 0.00 | 0.04 | 0.05 | 0.09 | 0.04 | 0.00 | 0.00 | Proteobacteria | Betaproteobacteria | Rhodocyclales | Rhodocyclaceae | Common taxa |
| OTU 593 | 0.00 | 0.11 | 0.05 | 0.14 | 0.00 | 0.05 | 0.04 | 0.00 | 0.05 | 0.04 | 0.00 | 0.00 | Proteobacteria | Alphaproteobacteria | Rhizobiales | Hyphomicrobiaceae | Common taxa |
| OTU 610 | 0.06 | 0.11 | 0.00 | 0.00 | 0.00 | 0.05 | 0.00 | 0.09 | 0.05 | 0.04 | 0.00 | 0.10 | Firmicutes | Clostridia | Clostridiales | Ruminococcaceae | Common taxa |
| OTU 623 | 0.00 | 0.00 | 0.00 | 0.05 | 0.00 | 0.05 | 0.18 | 0.05 | 0.05 | 0.04 | 0.00 | 0.00 | Proteobacteria | Betaproteobacteria | Burkholderiales | Burkholderiaceae | Common taxa |
| OTU 628 | 0.06 | 0.06 | 0.05 | 0.00 | 0.05 | 0.00 | 0.00 | 0.05 | 0.05 | 0.08 | 0.00 | 0.05 | Proteobacteria | Betaproteobacteria | Rhodocyclales | Rhodocyclaceae | Common taxa |
| OTU 633 | 0.00 | 0.06 | 0.00 | 0.05 | 0.00 | 0.00 | 0.04 | 0.00 | 0.05 | 0.04 | 0.11 | 0.10 | Proteobacteria | Alphaproteobacteria | Sphingomonadales | Sphingomonadaceae | Common taxa |
| OTU 645 | 0.06 | 0.00 | 0.00 | 0.09 | 0.00 | 0.00 | 0.00 | 0.05 | 0.05 | 0.08 | 0.11 | 0.00 | Proteobacteria | Gammaproteobacteria | Pseudomonadales | Moraxellaceae | Common taxa |
| OTU 653 | 0.00 | 0.06 | 0.05 | 0.00 | 0.00 | 0.00 | 0.04 | 0.00 | 0.05 | 0.08 | 0.00 | 0.15 | Ignavibacteriae | Ignavibacteria | Ignavibacteriales | Ignavibacteriaceae | Common taxa |
| OTU 682 | 0.06 | 0.06 | 0.00 | 0.00 | 0.00 | 0.00 | 0.04 | 0.09 | 0.09 | 0.00 | 0.00 | 0.05 | Proteobacteria | Betaproteobacteria | Rhodocyclales | Rhodocyclaceae | Common taxa |
| OTU 690 | 0.00 | 0.06 | 0.00 | 0.00 | 0.00 | 0.10 | 0.00 | 0.05 | 0.05 | 0.04 | 0.00 | 0.10 | Bacteroidetes | Flavobacteriia | Flavobacteriales | Cryomorphaceae | Common taxa |
| OTU 695 | 0.06 | 0.06 | 0.10 | 0.00 | 0.00 | 0.05 | 0.00 | 0.05 | 0.09 | 0.00 | 0.00 | 0.00 | Actinobacteria | Actinobacteria | Acidimicrobidae | Acidimicrobiales | Common taxa |
| OTU 704 | 0.06 | 0.06 | 0.00 | 0.09 | 0.00 | 0.00 | 0.00 | 0.05 | 0.09 | 0.04 | 0.00 | 0.00 | Proteobacteria | Deltaproteobacteria | Myxococcales | Sorangiineae | Common taxa |
| OTU 706 | 0.06 | 0.11 | 0.05 | 0.00 | 0.00 | 0.00 | 0.04 | 0.09 | 0.05 | 0.00 | 0.00 | 0.00 | Proteobacteria | Betaproteobacteria | Rhodocyclales | Rhodocyclaceae | Common taxa |
| OTU 708 | 0.00 | 0.06 | 0.05 | 0.00 | 0.00 | 0.00 | 0.00 | 0.09 | 0.05 | 0.04 | 0.05 | 0.05 | Proteobacteria | Betaproteobacteria | Rhodocyclales | Rhodocyclaceae | Common taxa |
| OTU 717 | 0.12 | 0.06 | 0.10 | 0.00 | 0.00 | 0.05 | 0.00 | 0.00 | 0.05 | 0.00 | 0.00 | 0.05 | Thermotogae | Thermotogae | Thermotogales | Thermotogaceae | Common taxa |
| OTU 731 | 0.00 | 0.06 | 0.14 | 0.00 | 0.00 | 0.00 | 0.04 | 0.05 | 0.05 | 0.04 | 0.00 | 0.00 | Proteobacteria | Betaproteobacteria | Rhodocyclales | Rhodocyclaceae | Common taxa |
| OTU 748 | 0.00 | 0.06 | 0.05 | 0.00 | 0.05 | 0.05 | 0.09 | 0.05 | 0.00 | 0.00 | 0.00 | 0.00 | Planctomycetes | Planctomycetia | Planctomycetales | Planctomycetaceae | Common taxa |
| OTU 767 | 0.00 | 0.06 | 0.05 | 0.05 | 0.00 | 0.00 | 0.09 | 0.00 | 0.00 | 0.04 | 0.00 | 0.05 | Proteobacteria | Betaproteobacteria | Burkholderiales | Burkholderiales_incertae_sedis | Common taxa |
| OTU 801 | 0.00 | 0.06 | 0.00 | 0.05 | 0.00 | 0.00 | 0.00 | 0.05 | 0.09 | 0.04 | 0.00 | 0.05 | Proteobacteria | Betaproteobacteria | Rhodocyclales | Rhodocyclaceae | Common taxa |
| OTU 804 | 0.06 | 0.00 | 0.00 | 0.00 | 0.05 | 0.05 | 0.09 | 0.05 | 0.00 | 0.04 | 0.00 | 0.00 | Proteobacteria | Betaproteobacteria | Rhodocyclales | Rhodocyclaceae | Common taxa |
| OTU 813 | 0.06 | 0.06 | 0.10 | 0.00 | 0.00 | 0.00 | 0.00 | 0.05 | 0.00 | 0.04 | 0.05 | 0.00 | Proteobacteria | Alphaproteobacteria | Rhodobacterales | Rhodobacteraceae | Common taxa |
| OTU 835 | 0.00 | 0.00 | 0.00 | 0.00 | 0.05 | 0.10 | 0.00 | 0.05 | 0.00 | 0.04 | 0.00 | 0.05 | Planctomycetes | Planctomycetia | Planctomycetales | Planctomycetaceae | Common taxa |
| OTU 845 | 0.06 | 0.06 | 0.00 | 0.00 | 0.00 | 0.00 | 0.00 | 0.09 | 0.00 | 0.00 | 0.05 | 0.05 | Proteobacteria | Betaproteobacteria | Rhodocyclales | Rhodocyclaceae | Common taxa |
| OTU 846 | 0.06 | 0.06 | 0.00 | 0.00 | 0.00 | 0.00 | 0.00 | 0.05 | 0.05 | 0.00 | 0.11 | 0.00 | Proteobacteria | Betaproteobacteria | Rhodocyclales | Rhodocyclaceae | Common taxa |
| OTU 864 | 0.06 | 0.00 | 0.05 | 0.05 | 0.00 | 0.00 | 0.00 | 0.00 | 0.05 | 0.00 | 0.11 | 0.00 | Proteobacteria | Alphaproteobacteria | Rhodobacterales | Rhodobacteraceae | Common taxa |
| OTU 889 | 0.00 | 0.00 | 0.10 | 0.05 | 0.05 | 0.05 | 0.00 | 0.00 | 0.00 | 0.00 | 0.00 | 0.05 | Proteobacteria | Betaproteobacteria | Rhodocyclales | Rhodocyclaceae | Common taxa |
| OTU 894 | 0.00 | 0.11 | 0.00 | 0.05 | 0.00 | 0.00 | 0.00 | 0.00 | 0.05 | 0.00 | 0.05 | 0.05 | Proteobacteria | Gammaproteobacteria | Chromatiales | Chromatiaceae | Common taxa |
| OTU 899 | 0.00 | 0.00 | 0.05 | 0.00 | 0.00 | 0.05 | 0.00 | 0.05 | 0.00 | 0.04 | 0.05 | 0.05 | Proteobacteria | Betaproteobacteria | Rhodocyclales | Rhodocyclaceae | Common taxa |
| OTU 914 | 0.12 | 0.06 | 0.00 | 0.00 | 0.00 | 0.00 | 0.00 | 0.00 | 0.00 | 0.04 | 0.05 | 0.05 | Proteobacteria | Betaproteobacteria | Burkholderiales | Comamonadaceae | Common taxa |
| OTU 925 | 0.00 | 0.06 | 0.10 | 0.00 | 0.00 | 0.00 | 0.00 | 0.05 | 0.05 | 0.00 | 0.00 | 0.05 | Proteobacteria | Betaproteobacteria | Rhodocyclales | Rhodocyclaceae | Common taxa |
| OTU 960 | 0.06 | 0.00 | 0.00 | 0.00 | 0.05 | 0.05 | 0.00 | 0.00 | 0.00 | 0.00 | 0.05 | 0.05 | Bacteroidetes | Sphingobacteriia | Sphingobacteriales | Saprospiraceae | Common taxa |
| OTU 970 | 0.06 | 0.00 | 0.00 | 0.00 | 0.05 | 0.00 | 0.00 | 0.05 | 0.00 | 0.04 | 0.05 | 0.00 | Proteobacteria | Alphaproteobacteria | Sphingomonadales | Sphingomonadaceae | Common taxa |
| OTU 974 | 0.00 | 0.00 | 0.00 | 0.05 | 0.05 | 0.05 | 0.00 | 0.05 | 0.00 | 0.04 | 0.00 | 0.00 | Firmicutes | Erysipelotrichia | Erysipelotrichales | Erysipelotrichaceae | Common taxa |
| OTU 1058 | 0.06 | 0.00 | 0.00 | 0.00 | 0.05 | 0.00 | 0.00 | 0.05 | 0.00 | 0.04 | 0.00 | 0.05 | Proteobacteria | Betaproteobacteria | Rhodocyclales | Rhodocyclaceae | Common taxa |
| OTU 1075 | 0.00 | 0.06 | 0.05 | 0.00 | 0.00 | 0.00 | 0.04 | 0.05 | 0.00 | 0.00 | 0.00 | 0.05 | Proteobacteria | Betaproteobacteria | Burkholderiales | Burkholderiales_incertae_sedis | Common taxa |
| OTU 1077 | 0.06 | 0.00 | 0.00 | 0.05 | 0.00 | 0.00 | 0.04 | 0.05 | 0.05 | 0.00 | 0.00 | 0.00 | Proteobacteria | Deltaproteobacteria | Myxococcales | Sorangiineae | Common taxa |
| OTU 1078 | 0.00 | 0.06 | 0.05 | 0.00 | 0.00 | 0.05 | 0.00 | 0.00 | 0.00 | 0.04 | 0.00 | 0.05 | Proteobacteria | Gammaproteobacteria | Methylococcales | Methylococcaceae | Common taxa |
| OTU 3 | 0.68 | 2.70 | 4.18 | 1.59 | 1.27 | 1.48 | 1.71 | 3.84 | 1.99 | 2.71 | 3.33 | 4.22 | Proteobacteria | Betaproteobacteria | Rhodocyclales | Rhodocyclaceae | Common taxa |
| OTU 4 | 12.02 | 0.86 | 0.00 | 0.18 | 0.79 | 3.22 | 0.70 | 0.05 | 4.40 | 4.00 | 0.00 | 0.35 | Bacteroidetes | Sphingobacteriia | Sphingobacteriales | Cyclobacteriaceae | Common taxa |
| OTU 10 | 0.00 | 2.01 | 0.24 | 1.27 | 0.90 | 4.25 | 1.98 | 2.62 | 2.13 | 1.33 | 0.05 | 0.55 | Planctomycetes | Planctomycetia | Planctomycetales | Planctomycetaceae | Common taxa |
| OTU 13 | 0.93 | 2.12 | 0.57 | 0.36 | 0.00 | 0.05 | 0.31 | 1.13 | 1.04 | 4.85 | 1.20 | 1.31 | Proteobacteria | Deltaproteobacteria | Myxococcales | Sorangiineae | Common taxa |
| OTU 15 | 0.62 | 1.26 | 0.29 | 2.45 | 0.48 | 0.00 | 0.26 | 0.23 | 0.80 | 0.40 | 1.64 | 4.92 | Proteobacteria | Deltaproteobacteria | Myxococcales | Nannocystineae | Common taxa |
| OTU 33 | 0.00 | 0.75 | 2.14 | 1.32 | 0.21 | 0.20 | 0.04 | 0.32 | 0.52 | 0.57 | 0.05 | 0.10 | Bacteroidetes | Flavobacteriia | Flavobacteriales | Cryomorphaceae | Common taxa |
| OTU 36 | 0.00 | 0.17 | 0.24 | 1.27 | 0.42 | 1.38 | 0.04 | 1.90 | 0.14 | 0.12 | 0.00 | 0.00 | Bacteroidetes | Sphingobacteriia | Sphingobacteriales | Saprospiraceae | Common taxa |
| OTU 44 | 0.00 | 0.57 | 0.90 | 0.27 | 0.42 | 0.77 | 0.22 | 0.32 | 0.57 | 0.44 | 0.33 | 0.30 | Proteobacteria | Betaproteobacteria | Burkholderiales | Comamonadaceae | Common taxa |
| OTU 45 | 0.00 | 0.06 | 2.04 | 0.41 | 0.58 | 1.02 | 0.53 | 0.23 | 0.05 | 0.04 | 0.00 | 0.05 | Bacteroidetes | Bacteroidetes_incertae_sedis | Ohtaekwangia | | Common taxa |
| OTU 48 | 0.62 | 0.00 | 0.00 | 0.32 | 2.33 | 1.28 | 0.09 | 0.14 | 0.09 | 0.00 | 0.33 | 0.20 | Proteobacteria | Alphaproteobacteria | Rhodobacterales | Rhodobacteraceae | Common taxa |
| OTU 53 | 0.06 | 0.17 | 1.62 | 1.04 | 0.21 | 0.61 | 0.44 | 0.18 | 0.00 | 0.16 | 0.00 | 0.20 | Proteobacteria | Gammaproteobacteria | Alteromonadales | Alteromonadaceae | Common taxa |
| OTU 58 | 0.12 | 0.06 | 0.00 | 0.18 | 0.00 | 0.26 | 0.00 | 0.14 | 0.38 | 0.97 | 1.20 | 1.11 | Proteobacteria | Alphaproteobacteria | Sphingomonadales | Sphingomonadaceae | Common taxa |
| OTU 64 | 0.00 | 0.00 | 0.19 | 0.64 | 0.74 | 0.46 | 0.26 | 0.86 | 0.33 | 0.16 | 0.00 | 0.20 | Planctomycetes | Planctomycetia | Planctomycetales | Planctomycetaceae | Common taxa |
| OTU 66 | 0.19 | 1.72 | 0.52 | 0.09 | 0.05 | 0.00 | 0.00 | 0.09 | 1.04 | 0.20 | 0.05 | 0.10 | Proteobacteria | Gammaproteobacteria | Xanthomonadales | Xanthomonadaceae | Common taxa |
| OTU 68 | 0.50 | 0.63 | 0.05 | 0.18 | 0.21 | 0.87 | 0.00 | 1.13 | 0.24 | 0.12 | 0.00 | 0.00 | Proteobacteria | Deltaproteobacteria | Myxococcales | Sorangiineae | Common taxa |
| OTU 70 | 0.00 | 0.17 | 1.14 | 0.00 | 0.16 | 0.10 | 0.35 | 0.27 | 0.90 | 0.12 | 0.44 | 0.05 | Proteobacteria | Betaproteobacteria | Rhodocyclales | Rhodocyclaceae | Common taxa |
| OTU 74 | 0.37 | 0.00 | 0.05 | 0.05 | 0.05 | 0.15 | 0.04 | 0.00 | 0.05 | 1.94 | 0.38 | 0.10 | Proteobacteria | Gammaproteobacteria | Xanthomonadales | Xanthomonadaceae | Common taxa |
| OTU 84 | 0.50 | 1.78 | 0.52 | 0.05 | 0.05 | 0.36 | 0.13 | 0.00 | 0.00 | 0.00 | 0.00 | 0.05 | Planctomycetes | Planctomycetia | Candidatus Brocadiales | Candidatus Brocadiaceae | Common taxa |
| OTU 86 | 0.00 | 0.17 | 0.29 | 0.14 | 0.53 | 0.72 | 0.26 | 0.09 | 0.28 | 0.36 | 0.11 | 0.10 | Proteobacteria | Betaproteobacteria | Burkholderiales | Comamonadaceae | Common taxa |
| OTU 88 | 0.12 | 0.00 | 0.00 | 0.14 | 1.48 | 0.00 | 0.04 | 0.05 | 0.14 | 0.32 | 0.55 | 0.20 | Proteobacteria | Betaproteobacteria | Burkholderiales | Comamonadaceae | Common taxa |
| OTU 90 | 0.25 | 0.06 | 0.10 | 0.00 | 0.00 | 0.10 | 0.04 | 0.05 | 0.38 | 0.85 | 0.76 | 0.25 | Proteobacteria | Alphaproteobacteria | Rhodobacterales | Rhodobacteraceae | Common taxa |
| OTU 92 | 0.00 | 0.06 | 0.00 | 0.36 | 0.53 | 0.15 | 0.04 | 0.00 | 0.00 | 0.08 | 1.09 | 0.60 | Proteobacteria | Betaproteobacteria | Rhodocyclales | Rhodocyclaceae | Common taxa |
| OTU 93 | 0.06 | 0.00 | 0.00 | 0.14 | 0.16 | 0.15 | 0.04 | 0.09 | 0.05 | 0.24 | 1.31 | 0.65 | Proteobacteria | Betaproteobacteria | Rhodocyclales | Rhodocyclaceae | Common taxa |
| OTU 94 | 0.37 | 0.06 | 0.00 | 0.00 | 0.16 | 0.00 | 0.00 | 0.05 | 0.61 | 1.21 | 0.05 | 0.05 | Proteobacteria | Gammaproteobacteria | Xanthomonadales | Xanthomonadaceae | Common taxa |
| OTU 95 | 0.37 | 0.23 | 0.24 | 0.00 | 0.00 | 0.00 | 0.18 | 0.41 | 0.61 | 0.04 | 0.16 | 0.55 | Proteobacteria | Deltaproteobacteria | Myxococcales | Nannocystineae | Common taxa |
| OTU 96 | 0.00 | 0.29 | 1.28 | 0.23 | 0.00 | 0.00 | 0.35 | 0.27 | 0.09 | 0.12 | 0.00 | 0.00 | Proteobacteria | Betaproteobacteria | Nitrosomonadales | Nitrosomonadaceae | Common taxa |
| OTU 98 | 0.19 | 0.57 | 0.10 | 0.00 | 0.05 | 0.05 | 0.00 | 0.14 | 0.80 | 0.49 | 0.05 | 0.25 | Proteobacteria | Alphaproteobacteria | Sphingomonadales | Sphingomonadaceae | Common taxa |
| OTU 99 | 0.06 | 0.00 | 0.38 | 1.00 | 0.00 | 0.51 | 0.00 | 0.05 | 0.14 | 0.24 | 0.22 | 0.00 | Proteobacteria | Epsilonproteobacteria | Campylobacterales | Campylobacteraceae | Common taxa |
| OTU 103 | 0.00 | 0.75 | 0.05 | 0.05 | 0.05 | 0.20 | 0.09 | 0.63 | 0.47 | 0.20 | 0.00 | 0.10 | Planctomycetes | Planctomycetia | Planctomycetales | Planctomycetaceae | Common taxa |
| OTU 110 | 0.25 | 0.17 | 0.00 | 0.00 | 0.21 | 0.15 | 0.04 | 0.00 | 0.71 | 0.36 | 0.55 | 0.05 | Bacteroidetes | Sphingobacteriia | Sphingobacteriales | Saprospiraceae | Common taxa |
| OTU 112 | 0.43 | 0.40 | 0.00 | 0.18 | 0.00 | 0.41 | 0.04 | 0.63 | 0.38 | 0.00 | 0.00 | 0.00 | Proteobacteria | Deltaproteobacteria | Myxococcales | Sorangiineae | Common taxa |
| OTU 113 | 0.06 | 1.09 | 0.33 | 0.05 | 0.05 | 0.00 | 0.04 | 0.00 | 0.66 | 0.16 | 0.00 | 0.05 | Proteobacteria | Gammaproteobacteria | Xanthomonadales | Xanthomonadaceae | Common taxa |
| OTU 115 | 0.06 | 0.17 | 1.05 | 0.00 | 0.05 | 0.05 | 0.04 | 0.00 | 0.28 | 0.36 | 0.11 | 0.05 | Proteobacteria | Betaproteobacteria | Rhodocyclales | Rhodocyclaceae | Common taxa |
| OTU 118 | 0.12 | 0.40 | 0.19 | 0.00 | 0.05 | 0.00 | 0.18 | 0.09 | 0.14 | 0.40 | 0.00 | 0.70 | Bacteroidetes | Sphingobacteriia | Sphingobacteriales | Chitinophagaceae | Common taxa |
| OTU 119 | 0.12 | 0.00 | 0.05 | 0.09 | 0.05 | 0.15 | 0.00 | 0.00 | 0.43 | 0.49 | 0.11 | 0.75 | Proteobacteria | Alphaproteobacteria | Rhizobiales | Hyphomicrobiaceae | Common taxa |
| OTU 121 | 0.06 | 0.17 | 0.52 | 0.05 | 0.00 | 0.00 | 0.13 | 0.41 | 0.28 | 0.20 | 0.05 | 0.30 | Proteobacteria | Betaproteobacteria | Rhodocyclales | Rhodocyclaceae | Common taxa |
| OTU 123 | 0.00 | 0.06 | 0.05 | 0.05 | 0.37 | 0.92 | 0.35 | 0.09 | 0.00 | 0.00 | 0.16 | 0.25 | Planctomycetes | Planctomycetia | Planctomycetales | Planctomycetaceae | Common taxa |
| OTU 124 | 0.00 | 1.15 | 0.05 | 0.00 | 0.00 | 0.00 | 0.04 | 0.09 | 0.80 | 0.12 | 0.00 | 0.05 | Proteobacteria | Gammaproteobacteria | Xanthomonadales | Xanthomonadaceae | Common taxa |
| OTU 125 | 0.12 | 0.98 | 0.24 | 0.05 | 0.00 | 0.00 | 0.00 | 0.00 | 0.47 | 0.12 | 0.11 | 0.25 | Proteobacteria | Gammaproteobacteria | Xanthomonadales | Xanthomonadaceae | Common taxa |
| OTU 127 | 0.00 | 0.40 | 0.43 | 0.23 | 0.00 | 0.00 | 0.00 | 0.41 | 0.43 | 0.08 | 0.00 | 0.10 | Bacteroidetes | Cytophagia | Cytophagales | Flammeovirgaceae | Common taxa |
| OTU 131 | 0.87 | 0.00 | 0.00 | 0.00 | 0.00 | 0.00 | 0.00 | 0.05 | 0.66 | 0.24 | 0.05 | 0.30 | Proteobacteria | Zetaproteobacteria | Mariprofundales | Mariprofundaceae | Common taxa |
| OTU 132 | 0.06 | 0.00 | 0.05 | 0.00 | 0.74 | 0.10 | 0.04 | 0.00 | 0.24 | 0.20 | 0.71 | 0.00 | Proteobacteria | Betaproteobacteria | Burkholderiales | Comamonadaceae | Common taxa |
| OTU 134 | 0.06 | 0.06 | 0.62 | 0.14 | 0.00 | 0.00 | 0.13 | 0.27 | 0.24 | 0.32 | 0.00 | 0.05 | Proteobacteria | Alphaproteobacteria | Sphingomonadales | Erythrobacteraceae | Common taxa |
| OTU 137 | 0.37 | 0.06 | 0.14 | 0.09 | 0.00 | 0.05 | 0.04 | 0.05 | 0.43 | 0.53 | 0.05 | 0.15 | Bacteroidetes | Flavobacteriia | Flavobacteriales | Flavobacteriaceae | Common taxa |
| OTU 138 | 0.19 | 0.06 | 0.33 | 0.05 | 0.05 | 0.10 | 0.13 | 0.36 | 0.19 | 0.20 | 0.16 | 0.15 | Proteobacteria | Betaproteobacteria | Rhodocyclales | Rhodocyclaceae | Common taxa |
| OTU 139 | 0.00 | 0.29 | 0.00 | 0.00 | 0.05 | 0.97 | 0.13 | 0.54 | 0.05 | 0.00 | 0.00 | 0.00 | Proteobacteria | Betaproteobacteria | Nitrosomonadales | Nitrosomonadaceae | Common taxa |
| OTU 140 | 0.19 | 0.00 | 0.14 | 0.00 | 0.00 | 0.20 | 0.09 | 0.14 | 0.19 | 0.49 | 0.11 | 0.40 | Proteobacteria | Alphaproteobacteria | Rhodobacterales | Rhodobacteraceae | Common taxa |
| OTU 141 | 0.00 | 0.23 | 0.33 | 0.14 | 0.11 | 0.10 | 0.04 | 0.27 | 0.19 | 0.16 | 0.11 | 0.25 | Proteobacteria | Betaproteobacteria | Rhodocyclales | Rhodocyclaceae | Common taxa |
| OTU 142 | 0.00 | 0.11 | 0.71 | 0.09 | 0.05 | 0.00 | 0.40 | 0.05 | 0.38 | 0.08 | 0.00 | 0.00 | Planctomycetes | Phycisphaerae | Phycisphaerales | Phycisphaeraceae | Common taxa |
| OTU 144 | 1.55 | 0.11 | 0.19 | 0.00 | 0.00 | 0.00 | 0.09 | 0.05 | 0.09 | 0.00 | 0.00 | 0.15 | Proteobacteria | Betaproteobacteria | Burkholderiales | Comamonadaceae | Common taxa |
| OTU 145 | 0.00 | 0.06 | 0.86 | 0.09 | 0.00 | 0.05 | 0.04 | 0.09 | 0.33 | 0.12 | 0.22 | 0.00 | Proteobacteria | Betaproteobacteria | Rhodocyclales | Rhodocyclaceae | Common taxa |
| OTU 146 | 0.06 | 0.29 | 0.19 | 0.23 | 0.00 | 0.05 | 0.00 | 0.14 | 0.47 | 0.24 | 0.00 | 0.20 | Proteobacteria | Alphaproteobacteria | Rhizobiales | Bradyrhizobiaceae | Common taxa |
| OTU 148 | 0.62 | 0.11 | 0.10 | 0.27 | 0.00 | 0.00 | 0.00 | 0.05 | 0.14 | 0.32 | 0.11 | 0.25 | Proteobacteria | Gammaproteobacteria | Pseudomonadales | Moraxellaceae | Common taxa |
| OTU 150 | 0.25 | 0.29 | 0.00 | 0.00 | 0.00 | 0.00 | 0.18 | 0.00 | 0.05 | 0.20 | 0.33 | 0.65 | Planctomycetes | Planctomycetia | Candidatus Brocadiales | Candidatus Brocadiaceae | Common taxa |
| OTU 153 | 0.06 | 0.06 | 0.00 | 0.00 | 0.00 | 0.05 | 0.00 | 0.00 | 0.14 | 0.69 | 0.38 | 0.40 | Proteobacteria | Alphaproteobacteria | Sphingomonadales | Sphingomonadaceae | Common taxa |
| OTU 155 | 0.19 | 0.40 | 0.00 | 0.09 | 0.11 | 0.61 | 0.04 | 0.14 | 0.14 | 0.08 | 0.00 | 0.15 | Planctomycetes | Planctomycetia | Planctomycetales | Planctomycetaceae | Common taxa |
| OTU 159 | 0.31 | 0.06 | 0.00 | 0.00 | 0.21 | 0.61 | 0.40 | 0.00 | 0.00 | 0.08 | 0.16 | 0.05 | Proteobacteria | Betaproteobacteria | Burkholderiales | Burkholderiales_incertae_sedis | Common taxa |
| OTU 160 | 0.25 | 0.00 | 0.00 | 0.00 | 0.16 | 0.00 | 0.04 | 0.00 | 0.00 | 0.49 | 0.87 | 0.05 | Proteobacteria | Gammaproteobacteria | Xanthomonadales | Xanthomonadaceae | Common taxa |
| OTU 161 | 0.25 | 0.06 | 0.14 | 0.00 | 0.00 | 0.00 | 0.04 | 0.09 | 0.24 | 0.12 | 0.55 | 0.40 | Proteobacteria | Betaproteobacteria | Rhodocyclales | Rhodocyclaceae | Common taxa |
| OTU 163 | 0.00 | 0.06 | 0.14 | 0.00 | 0.16 | 0.00 | 0.18 | 0.45 | 0.24 | 0.32 | 0.11 | 0.00 | Lentisphaerae | Lentisphaeria | Lentisphaerales | Lentisphaeraceae | Common taxa |
| OTU 164 | 0.00 | 0.80 | 0.33 | 0.05 | 0.21 | 0.05 | 0.00 | 0.00 | 0.09 | 0.16 | 0.00 | 0.15 | Planctomycetes | Planctomycetia | Planctomycetales | Planctomycetaceae | Common taxa |
| OTU 166 | 0.31 | 0.00 | 0.10 | 0.23 | 0.00 | 0.20 | 0.04 | 0.05 | 0.47 | 0.04 | 0.16 | 0.20 | Proteobacteria | Gammaproteobacteria | Pseudomonadales | Moraxellaceae | Common taxa |
| OTU 167 | 0.00 | 0.17 | 0.00 | 0.09 | 0.05 | 0.10 | 0.00 | 0.14 | 0.90 | 0.04 | 0.27 | 0.00 | Proteobacteria | Alphaproteobacteria | Rhodobacterales | Rhodobacteraceae | Common taxa |
| OTU 168 | 0.12 | 0.29 | 0.05 | 0.05 | 0.21 | 0.26 | 0.04 | 0.00 | 0.14 | 0.04 | 0.00 | 0.65 | Planctomycetes | Planctomycetia | Planctomycetales | Planctomycetaceae | Common taxa |
| OTU 169 | 0.00 | 0.06 | 0.10 | 0.18 | 0.00 | 0.36 | 0.00 | 0.32 | 0.33 | 0.12 | 0.05 | 0.10 | Planctomycetes | Planctomycetia | Planctomycetales | Planctomycetaceae | Common taxa |
| OTU 170 | 0.50 | 0.29 | 0.00 | 0.00 | 0.00 | 0.00 | 0.09 | 0.27 | 0.09 | 0.20 | 0.00 | 0.30 | Proteobacteria | Deltaproteobacteria | Myxococcales | Nannocystineae | Common taxa |
| OTU 171 | 0.37 | 0.34 | 0.14 | 0.00 | 0.00 | 0.00 | 0.09 | 0.14 | 0.24 | 0.08 | 0.11 | 0.25 | Proteobacteria | Betaproteobacteria | Rhodocyclales | Rhodocyclaceae | Common taxa |
| OTU 175 | 0.81 | 0.57 | 0.05 | 0.00 | 0.00 | 0.00 | 0.00 | 0.00 | 0.05 | 0.00 | 0.00 | 0.40 | Chloroflexi | Anaerolineae | Anaerolineales | Anaerolineaceae | Common taxa |
| OTU 176 | 0.12 | 0.06 | 0.19 | 0.09 | 0.00 | 0.26 | 0.13 | 0.14 | 0.05 | 0.36 | 0.00 | 0.15 | Proteobacteria | Betaproteobacteria | Burkholderiales | Burkholderiales_incertae_sedis | Common taxa |
| OTU 178 | 0.06 | 0.11 | 0.00 | 0.00 | 0.00 | 0.00 | 0.00 | 0.00 | 0.19 | 0.61 | 0.00 | 0.55 | Proteobacteria | Alphaproteobacteria | Rhizobiales | Beijerinckiaceae | Common taxa |
| OTU 180 | 0.06 | 0.57 | 0.19 | 0.05 | 0.00 | 0.00 | 0.00 | 0.00 | 0.43 | 0.16 | 0.00 | 0.15 | Bacteroidetes | Sphingobacteriia | Sphingobacteriales | Saprospiraceae | Common taxa |
| OTU 181 | 0.00 | 0.00 | 0.14 | 0.09 | 0.21 | 0.05 | 0.00 | 0.18 | 0.19 | 0.44 | 0.16 | 0.00 | Proteobacteria | Alphaproteobacteria | Rhodobacterales | Rhodobacteraceae | Common taxa |
| OTU 182 | 0.00 | 0.11 | 0.00 | 0.05 | 0.00 | 0.00 | 0.00 | 0.09 | 0.00 | 0.08 | 0.71 | 0.60 | Proteobacteria | Gammaproteobacteria | Chromatiales | Ectothiorhodospiraceae | Common taxa |
| OTU 187 | 0.00 | 0.00 | 0.24 | 0.64 | 0.05 | 0.36 | 0.09 | 0.05 | 0.00 | 0.04 | 0.00 | 0.00 | Proteobacteria | Alphaproteobacteria | Sphingomonadales | Erythrobacteraceae | Common taxa |
| OTU 190 | 0.93 | 0.06 | 0.05 | 0.00 | 0.05 | 0.05 | 0.04 | 0.00 | 0.00 | 0.00 | 0.11 | 0.45 | Verrucomicrobia | Opitutae | Opitutales | Opitutaceae | Common taxa |
| OTU 191 | 0.68 | 0.00 | 0.05 | 0.05 | 0.00 | 0.05 | 0.00 | 0.00 | 0.00 | 0.36 | 0.00 | 0.40 | Proteobacteria | Alphaproteobacteria | Rhizobiales | Methylocystaceae | Common taxa |
| OTU 193 | 0.00 | 0.00 | 0.00 | 0.00 | 0.00 | 0.10 | 0.09 | 0.23 | 0.99 | 0.00 | 0.00 | 0.00 | Chloroflexi | Thermomicrobia | Thermomicrobiales | Thermomicrobiaceae | Common taxa |
| OTU 194 | 0.19 | 0.00 | 0.00 | 0.05 | 0.48 | 0.31 | 0.09 | 0.00 | 0.05 | 0.04 | 0.27 | 0.10 | Proteobacteria | Betaproteobacteria | Rhodocyclales | Rhodocyclaceae | Common taxa |
| OTU 195 | 0.00 | 0.00 | 0.00 | 0.00 | 0.00 | 0.46 | 0.13 | 0.77 | 0.00 | 0.04 | 0.00 | 0.00 | Acidobacteria | Acidobacteria_Gp4 | Gp4 | | Common taxa |
| OTU 197 | 0.00 | 0.00 | 0.19 | 0.27 | 0.37 | 0.36 | 0.26 | 0.00 | 0.00 | 0.00 | 0.00 | 0.00 | Proteobacteria | Deltaproteobacteria | Myxococcales | Sorangiineae | Common taxa |
| OTU 198 | 0.12 | 0.11 | 0.00 | 0.00 | 0.00 | 0.00 | 0.09 | 0.27 | 0.05 | 0.20 | 0.49 | 0.15 | Proteobacteria | Deltaproteobacteria | Myxococcales | Sorangiineae | Common taxa |
| OTU 199 | 0.00 | 0.06 | 0.19 | 0.00 | 0.00 | 0.10 | 0.00 | 0.86 | 0.14 | 0.04 | 0.00 | 0.00 | Planctomycetes | Planctomycetia | Planctomycetales | Planctomycetaceae | Common taxa |
| OTU 202 | 0.19 | 0.69 | 0.00 | 0.00 | 0.00 | 0.15 | 0.09 | 0.00 | 0.00 | 0.04 | 0.00 | 0.40 | Proteobacteria | Gammaproteobacteria | Chromatiales | Ectothiorhodospiraceae | Common taxa |
| OTU 204 | 0.25 | 0.00 | 0.33 | 0.18 | 0.05 | 0.26 | 0.00 | 0.23 | 0.09 | 0.00 | 0.00 | 0.05 | Proteobacteria | Epsilonproteobacteria | Campylobacterales | Campylobacteraceae | Common taxa |
| OTU 206 | 0.12 | 0.00 | 0.00 | 0.00 | 0.00 | 0.05 | 0.00 | 0.00 | 0.05 | 0.53 | 0.44 | 0.20 | Bacteroidetes | Sphingobacteriia | Sphingobacteriales | Saprospiraceae | Common taxa |
| OTU 209 | 0.00 | 0.06 | 0.00 | 0.18 | 0.05 | 0.05 | 0.09 | 0.09 | 0.80 | 0.00 | 0.00 | 0.00 | Proteobacteria | Gammaproteobacteria | Chromatiales | Halothiobacillaceae | Common taxa |
| OTU 210 | 0.00 | 0.11 | 0.86 | 0.00 | 0.05 | 0.00 | 0.00 | 0.27 | 0.05 | 0.00 | 0.00 | 0.00 | Proteobacteria | Deltaproteobacteria | Myxococcales | Nannocystineae | Common taxa |
| OTU 211 | 0.00 | 0.00 | 0.00 | 0.41 | 0.48 | 0.41 | 0.09 | 0.00 | 0.00 | 0.00 | 0.00 | 0.00 | Proteobacteria | Deltaproteobacteria | Myxococcales | Cystobacterineae | Common taxa |
| OTU 212 | 0.68 | 0.17 | 0.00 | 0.05 | 0.11 | 0.00 | 0.00 | 0.05 | 0.09 | 0.08 | 0.05 | 0.25 | Proteobacteria | Betaproteobacteria | Rhodocyclales | Rhodocyclaceae | Common taxa |
| OTU 213 | 0.06 | 0.00 | 0.00 | 0.05 | 0.05 | 0.00 | 0.13 | 0.00 | 0.19 | 0.49 | 0.27 | 0.05 | Proteobacteria | Deltaproteobacteria | Myxococcales | Nannocystineae | Common taxa |
| OTU 214 | 0.06 | 0.00 | 0.05 | 0.05 | 0.00 | 0.00 | 0.00 | 0.05 | 0.09 | 0.36 | 0.33 | 0.35 | Proteobacteria | Alphaproteobacteria | Rhodobacterales | Rhodobacteraceae | Common taxa |
| OTU 215 | 0.00 | 0.00 | 0.00 | 0.00 | 0.00 | 0.00 | 0.00 | 0.05 | 0.19 | 0.89 | 0.00 | 0.05 | Bacteroidetes | Sphingobacteriia | Sphingobacteriales | Saprospiraceae | Common taxa |
| OTU 216 | 0.06 | 0.00 | 0.00 | 0.00 | 0.00 | 0.00 | 0.00 | 0.00 | 0.00 | 0.16 | 0.33 | 0.85 | Bacteroidetes | Flavobacteriia | Flavobacteriales | Flavobacteriaceae | Common taxa |
| OTU 217 | 0.06 | 0.23 | 0.00 | 0.00 | 0.00 | 0.00 | 0.00 | 0.23 | 0.61 | 0.16 | 0.00 | 0.00 | Bacteroidetes | Sphingobacteriia | Sphingobacteriales | Chitinophagaceae | Common taxa |
| OTU 218 | 0.25 | 0.00 | 0.00 | 0.00 | 0.00 | 0.00 | 0.00 | 0.05 | 0.05 | 0.04 | 0.33 | 0.70 | Bacteroidetes | Sphingobacteriia | Sphingobacteriales | Chitinophagaceae | Common taxa |
| OTU 222 | 0.00 | 0.06 | 0.76 | 0.00 | 0.00 | 0.00 | 0.31 | 0.05 | 0.05 | 0.04 | 0.00 | 0.00 | Bacteroidetes | Flavobacteriia | Flavobacteriales | Cryomorphaceae | Common taxa |
| OTU 223 | 0.00 | 0.00 | 0.19 | 0.45 | 0.16 | 0.15 | 0.13 | 0.05 | 0.05 | 0.00 | 0.00 | 0.10 | Proteobacteria | Gammaproteobacteria | Alteromonadales | Alteromonadaceae | Common taxa |
| OTU 224 | 0.00 | 0.29 | 0.00 | 0.00 | 0.00 | 0.00 | 0.00 | 0.00 | 0.05 | 0.00 | 0.11 | 0.95 | Proteobacteria | Betaproteobacteria | Rhodocyclales | Rhodocyclaceae | Common taxa |
| OTU 226 | 0.31 | 0.00 | 0.00 | 0.00 | 0.00 | 0.00 | 0.00 | 0.00 | 0.43 | 0.16 | 0.38 | 0.05 | Proteobacteria | Deltaproteobacteria | Myxococcales | Sorangiineae | Common taxa |
| OTU 227 | 0.00 | 0.00 | 0.00 | 0.00 | 0.00 | 0.05 | 0.00 | 0.00 | 0.05 | 0.04 | 1.15 | 0.10 | Proteobacteria | Betaproteobacteria | Burkholderiales | Comamonadaceae | Common taxa |
| OTU 228 | 0.00 | 0.00 | 0.10 | 0.05 | 0.11 | 0.10 | 0.00 | 0.27 | 0.43 | 0.16 | 0.00 | 0.00 | candidate division WPS-1 | WPS-1_genera_incertae_sedis | | | Common taxa |
| OTU 229 | 0.00 | 0.40 | 0.05 | 0.05 | 0.00 | 0.00 | 0.18 | 0.00 | 0.05 | 0.12 | 0.16 | 0.30 | Proteobacteria | Gammaproteobacteria | Gammaproteobacteria_incertae_sedis | Congregibacter | Common taxa |
| OTU 230 | 0.00 | 0.06 | 0.14 | 0.00 | 0.00 | 0.00 | 0.00 | 0.09 | 0.24 | 0.36 | 0.11 | 0.20 | Proteobacteria | Betaproteobacteria | Rhodocyclales | Rhodocyclaceae | Common taxa |
| OTU 231 | 0.00 | 0.00 | 0.00 | 0.18 | 0.48 | 0.15 | 0.35 | 0.09 | 0.00 | 0.00 | 0.00 | 0.00 | Bacteroidetes | Flavobacteriia | Flavobacteriales | Flavobacteriaceae | Common taxa |

| | | | | | | | | | | | | | | | | | |
|---|---|---|---|---|---|---|---|---|---|---|---|---|---|---|---|---|---|
| OTU 233 | 0.06 | 0.00 | 0.14 | 0.00 | 0.00 | 0.05 | 0.04 | 0.50 | 0.19 | 0.04 | 0.16 | 0.05 | *Chloroflexi* | *Anaerolineae* | *Anaerolineales* | *Anaerolineaceae* | Common taxa |
| OTU 237 | 0.00 | 0.34 | 0.24 | 0.05 | 0.05 | 0.00 | 0.09 | 0.18 | 0.19 | 0.00 | 0.11 | 0.00 | *Proteobacteria* | *Betaproteobacteria* | *Rhodocyclales* | *Rhodocyclaceae* | Common taxa |
| OTU 238 | 0.00 | 0.29 | 0.52 | 0.09 | 0.00 | 0.00 | 0.00 | 0.14 | 0.19 | 0.00 | 0.00 | 0.00 | *Verrucomicrobia* | *Opitutae* | *Opitutales* | *Opitutaceae* | Common taxa |
| OTU 239 | 0.12 | 0.00 | 0.00 | 0.00 | 0.00 | 0.00 | 0.09 | 0.00 | 0.09 | 0.24 | 0.16 | 0.50 | *Proteobacteria* | *Alphaproteobacteria* | *Sphingomonadales* | *Sphingomonadaceae* | Common taxa |
| OTU 241 | 0.12 | 0.17 | 0.00 | 0.05 | 0.00 | 0.20 | 0.00 | 0.32 | 0.19 | 0.04 | 0.00 | 0.15 | *Proteobacteria* | *Deltaproteobacteria* | *Myxococcales* | *Sorangiineae* | Common taxa |
| OTU 242 | 0.12 | 1.03 | 0.00 | 0.05 | 0.00 | 0.10 | 0.00 | 0.00 | 0.00 | 0.00 | 0.05 | 0.05 | *Proteobacteria* | *Betaproteobacteria* | *Rhodocyclales* | *Rhodocyclaceae* | Common taxa |
| OTU 243 | 0.00 | 1.09 | 0.10 | 0.00 | 0.00 | 0.10 | 0.00 | 0.05 | 0.00 | 0.00 | 0.05 | 0.00 | *Proteobacteria* | *Betaproteobacteria* | *Burkholderiales* | *Comamonadaceae* | Common taxa |
| OTU 244 | 0.00 | 0.00 | 0.05 | 0.23 | 0.48 | 0.41 | 0.04 | 0.00 | 0.05 | 0.00 | 0.00 | 0.00 | *Proteobacteria* | *Betaproteobacteria* | *Burkholderiales* | *Comamonadaceae* | Common taxa |
| OTU 245 | 0.31 | 0.00 | 0.00 | 0.00 | 0.00 | 0.00 | 0.00 | 0.14 | 0.43 | 0.16 | 0.16 | 0.05 | *Proteobacteria* | *Deltaproteobacteria* | *Myxococcales* | *Nannocystineae* | Common taxa |
| OTU 246 | 0.00 | 0.06 | 0.05 | 0.05 | 1.11 | 0.05 | 0.00 | 0.00 | 0.00 | 0.00 | 0.00 | 0.00 | *Proteobacteria* | *Deltaproteobacteria* | *Desulfobacterales* | *Desulfobulbaceae* | Common taxa |
| OTU 248 | 0.19 | 0.00 | 0.00 | 0.00 | 0.00 | 0.00 | 0.00 | 0.00 | 0.00 | 0.08 | 0.16 | 0.85 | *Proteobacteria* | *Gammaproteobacteria* | *Xanthomonadales* | *Xanthomonadaceae* | Common taxa |
| OTU 249 | 0.25 | 0.00 | 0.10 | 0.00 | 0.05 | 0.20 | 0.00 | 0.14 | 0.19 | 0.00 | 0.05 | 0.25 | *Bacteroidetes* | *Bacteroidia* | *Bacteroidales* | *Porphyromonadaceae* | Common taxa |
| OTU 252 | 0.00 | 0.98 | 0.05 | 0.00 | 0.05 | 0.00 | 0.00 | 0.00 | 0.09 | 0.08 | 0.00 | 0.05 | *Planctomycetes* | *Planctomycetia* | *Planctomycetales* | *Planctomycetaceae* | Common taxa |
| OTU 254 | 0.19 | 0.00 | 0.00 | 0.14 | 0.21 | 0.36 | 0.04 | 0.00 | 0.00 | 0.12 | 0.11 | 0.05 | *Proteobacteria* | *Betaproteobacteria* | *Rhodocyclales* | *Rhodocyclaceae* | Common taxa |
| OTU 255 | 0.06 | 0.46 | 0.00 | 0.00 | 0.00 | 0.00 | 0.00 | 0.05 | 0.14 | 0.28 | 0.22 | 0.00 | *Parcubacteria* | *Parcubacteria_genera_incertae_sedis* | | | Common taxa |
| OTU 258 | 0.50 | 0.00 | 0.00 | 0.00 | 0.05 | 0.05 | 0.00 | 0.00 | 0.00 | 0.04 | 0.49 | 0.20 | *Proteobacteria* | *Gammaproteobacteria* | *Xanthomonadales* | *Xanthomonadaceae* | Common taxa |
| OTU 259 | 0.00 | 0.00 | 0.00 | 0.45 | 0.53 | 0.10 | 0.00 | 0.09 | 0.00 | 0.00 | 0.00 | 0.00 | *Proteobacteria* | *Betaproteobacteria* | *Rhodocyclales* | *Rhodocyclaceae* | Common taxa |
| OTU 260 | 0.00 | 0.00 | 0.00 | 0.09 | 0.11 | 0.10 | 0.31 | 0.41 | 0.09 | 0.00 | 0.00 | 0.00 | *Planctomycetes* | *Planctomycetia* | *Planctomycetales* | *Planctomycetaceae* | Common taxa |
| OTU 261 | 1.12 | 0.06 | 0.05 | 0.00 | 0.00 | 0.00 | 0.00 | 0.00 | 0.00 | 0.00 | 0.00 | 0.20 | *Bacteroidetes* | *Cytophagia* | *Cytophagales* | *Flammeovirgaceae* | Common taxa |
| OTU 263 | 0.19 | 0.00 | 0.29 | 0.05 | 0.00 | 0.00 | 0.00 | 0.00 | 0.00 | 0.04 | 0.11 | 0.55 | *Bacteroidetes* | *Sphingobacteriia* | *Sphingobacteriales* | *Chitinophagaceae* | Common taxa |
| OTU 264 | 0.87 | 0.29 | 0.05 | 0.00 | 0.05 | 0.00 | 0.00 | 0.00 | 0.00 | 0.00 | 0.00 | 0.10 | *Chloroflexi* | *Anaerolineae* | *Anaerolineales* | *Anaerolineaceae* | Common taxa |
| OTU 266 | 0.00 | 0.00 | 0.00 | 0.14 | 0.64 | 0.10 | 0.26 | 0.00 | 0.00 | 0.00 | 0.00 | 0.00 | *Bacteroidetes* | *Flavobacteriia* | *Flavobacteriales* | *Flavobacteriaceae* | Common taxa |
| OTU 267 | 0.00 | 0.29 | 0.19 | 0.14 | 0.00 | 0.00 | 0.00 | 0.27 | 0.24 | 0.00 | 0.00 | 0.00 | *Bacteroidetes* | *Sphingobacteriia* | *Sphingobacteriales* | *Saprospiraceae* | Common taxa |
| OTU 268 | 0.00 | 0.23 | 0.57 | 0.05 | 0.05 | 0.00 | 0.00 | 0.00 | 0.09 | 0.00 | 0.11 | 0.05 | *Proteobacteria* | *Betaproteobacteria* | *Rhodocyclales* | *Rhodocyclaceae* | Common taxa |
| OTU 272 | 0.00 | 0.00 | 0.05 | 0.23 | 0.21 | 0.51 | 0.13 | 0.00 | 0.00 | 0.00 | 0.00 | 0.00 | *Proteobacteria* | *Alphaproteobacteria* | *Rhodospirillales* | *Rhodospirillaceae* | Common taxa |
| OTU 275 | 0.12 | 0.00 | 0.00 | 0.00 | 0.00 | 0.00 | 0.00 | 0.00 | 0.00 | 0.04 | 0.98 | 0.10 | *Proteobacteria* | *Deltaproteobacteria* | *Myxococcales* | *Nannocystineae* | Common taxa |
| OTU 276 | 0.00 | 0.06 | 0.05 | 0.00 | 0.00 | 0.00 | 0.00 | 0.09 | 0.24 | 0.40 | 0.05 | 0.10 | *Proteobacteria* | *Alphaproteobacteria* | *Rhodobacterales* | *Rhodobacteraceae* | Common taxa |
| OTU 279 | 0.12 | 0.00 | 0.00 | 0.00 | 0.00 | 0.05 | 0.04 | 0.00 | 0.09 | 0.12 | 0.71 | 0.00 | *Proteobacteria* | *Betaproteobacteria* | *Burkholderiales* | *Comamonadaceae* | Common taxa |
| OTU 280 | 0.06 | 0.06 | 0.05 | 0.00 | 0.05 | 0.00 | 0.00 | 0.00 | 0.66 | 0.08 | 0.05 | 0.05 | *Bacteroidetes* | *Sphingobacteriia* | *Sphingobacteriales* | *Saprospiraceae* | Common taxa |
| OTU 281 | 0.06 | 0.52 | 0.14 | 0.00 | 0.00 | 0.00 | 0.26 | 0.05 | 0.05 | 0.00 | 0.00 | 0.05 | *Proteobacteria* | *Gammaproteobacteria* | *Chromatiales* | *Ectothiorhodospiraceae* | Common taxa |
| OTU 283 | 0.00 | 0.06 | 0.05 | 0.05 | 0.00 | 0.05 | 0.00 | 0.00 | 0.09 | 0.36 | 0.22 | 0.15 | *Proteobacteria* | *Alphaproteobacteria* | *Rhodobacterales* | *Rhodobacteraceae* | Common taxa |
| OTU 284 | 0.00 | 0.00 | 0.14 | 0.41 | 0.05 | 0.20 | 0.00 | 0.00 | 0.14 | 0.08 | 0.00 | 0.00 | *Bacteroidetes* | *Flavobacteriia* | *Flavobacteriales* | *Flavobacteriaceae* | Common taxa |
| OTU 286 | 0.00 | 0.00 | 0.19 | 0.14 | 0.00 | 0.05 | 0.00 | 0.18 | 0.09 | 0.12 | 0.11 | 0.15 | *Proteobacteria* | *Epsilonproteobacteria* | *Campylobacterales* | *Campylobacteraceae* | Common taxa |
| OTU 287 | 0.00 | 0.06 | 0.62 | 0.14 | 0.00 | 0.00 | 0.04 | 0.18 | 0.00 | 0.00 | 0.00 | 0.00 | *Proteobacteria* | *Betaproteobacteria* | *Nitrosomonadales* | *Nitrosomonadaceae* | Common taxa |
| OTU 291 | 0.00 | 0.00 | 0.00 | 0.00 | 0.00 | 0.05 | 0.04 | 0.00 | 0.00 | 0.04 | 1.04 | 0.00 | *Proteobacteria* | *Deltaproteobacteria* | *Myxococcales* | *Nannocystineae* | Common taxa |
| OTU 293 | 0.00 | 0.00 | 0.00 | 0.05 | 0.16 | 0.26 | 0.09 | 0.00 | 0.09 | 0.08 | 0.00 | 0.30 | *Proteobacteria* | *Alphaproteobacteria* | *Sphingomonadales* | *Sphingomonadaceae* | Common taxa |
| OTU 294 | 0.00 | 0.69 | 0.05 | 0.00 | 0.00 | 0.00 | 0.00 | 0.00 | 0.24 | 0.08 | 0.05 | 0.00 | *Parcubacteria* | *Parcubacteria_genera_incertae_sedis* | | | Common taxa |
| OTU 296 | 0.00 | 0.00 | 0.00 | 0.27 | 0.11 | 0.31 | 0.18 | 0.00 | 0.09 | 0.00 | 0.00 | 0.05 | *Proteobacteria* | *Alphaproteobacteria* | *Rhizobiales* | *Hyphomicrobiaceae* | Common taxa |
| OTU 297 | 1.05 | 0.06 | 0.00 | 0.00 | 0.00 | 0.05 | 0.00 | 0.00 | 0.00 | 0.08 | 0.00 | 0.00 | *Bacteroidetes* | *Sphingobacteriia* | *Sphingobacteriales* | *Saprospiraceae* | Common taxa |
| OTU 298 | 0.12 | 0.06 | 0.00 | 0.18 | 0.00 | 0.10 | 0.00 | 0.09 | 0.00 | 0.00 | 0.05 | 0.45 | *Proteobacteria* | *Alphaproteobacteria* | *Rhodospirillales* | *Acetobacteraceae* | Common taxa |
| OTU 299 | 0.81 | 0.11 | 0.00 | 0.00 | 0.05 | 0.00 | 0.00 | 0.00 | 0.00 | 0.12 | 0.05 | 0.05 | *Proteobacteria* | *Alphaproteobacteria* | *Caulobacterales* | *Caulobacteraceae* | Common taxa |
| OTU 301 | 0.00 | 0.00 | 0.24 | 0.00 | 0.00 | 0.00 | 0.00 | 0.27 | 0.33 | 0.12 | 0.00 | 0.00 | *Proteobacteria* | *Betaproteobacteria* | *Rhodocyclales* | *Rhodocyclaceae* | Common taxa |
| OTU 303 | 0.12 | 0.17 | 0.05 | 0.05 | 0.05 | 0.51 | 0.00 | 0.05 | 0.05 | 0.00 | 0.00 | 0.00 | *Chloroflexi* | *Thermomicrobia* | *Sphaerobacteridae* | *Sphaerobacterales* | Common taxa |
| OTU 304 | 0.00 | 0.00 | 0.00 | 0.00 | 0.00 | 0.05 | 0.26 | 0.27 | 0.28 | 0.00 | 0.05 | 0.00 | *Proteobacteria* | *Betaproteobacteria* | *Burkholderiales* | *Burkholderiaceae* | Common taxa |
| OTU 305 | 0.06 | 0.17 | 0.38 | 0.00 | 0.00 | 0.00 | 0.00 | 0.00 | 0.09 | 0.08 | 0.05 | 0.15 | *Proteobacteria* | *Betaproteobacteria* | *Rhodocyclales* | *Rhodocyclaceae* | Common taxa |
| OTU 306 | 0.00 | 0.11 | 0.05 | 0.00 | 0.00 | 0.00 | 0.09 | 0.09 | 0.33 | 0.00 | 0.00 | 0.30 | *Planctomycetes* | *Planctomycetia* | *Planctomycetales* | *Planctomycetaceae* | Common taxa |
| OTU 307 | 0.06 | 0.23 | 0.00 | 0.00 | 0.00 | 0.00 | 0.00 | 0.23 | 0.09 | 0.08 | 0.00 | 0.30 | *Acidobacteria* | *Acidobacteria_Gp6* | *Gp6* | | Common taxa |
| OTU 308 | 0.19 | 0.06 | 0.00 | 0.14 | 0.05 | 0.10 | 0.00 | 0.00 | 0.00 | 0.00 | 0.38 | 0.15 | *Proteobacteria* | *Gammaproteobacteria* | *Xanthomonadales* | *Xanthomonadaceae* | Common taxa |
| OTU 309 | 0.00 | 0.06 | 0.48 | 0.05 | 0.00 | 0.00 | 0.04 | 0.09 | 0.00 | 0.08 | 0.11 | 0.05 | *Proteobacteria* | *Alphaproteobacteria* | *Rhodobacterales* | *Rhodobacteraceae* | Common taxa |
| OTU 310 | 0.00 | 0.00 | 0.33 | 0.14 | 0.05 | 0.10 | 0.00 | 0.32 | 0.00 | 0.00 | 0.00 | 0.00 | *Proteobacteria* | *Deltaproteobacteria* | *Myxococcales* | *Nannocystineae* | Common taxa |
| OTU 311 | 0.00 | 0.34 | 0.38 | 0.09 | 0.00 | 0.05 | 0.00 | 0.05 | 0.05 | 0.04 | 0.00 | 0.00 | *Planctomycetes* | *Planctomycetia* | *Planctomycetales* | *Planctomycetaceae* | Common taxa |
| OTU 312 | 0.25 | 0.29 | 0.00 | 0.00 | 0.00 | 0.00 | 0.00 | 0.00 | 0.09 | 0.08 | 0.11 | 0.25 | *Planctomycetes* | *Phycisphaerae* | *Phycisphaerales* | *Phycisphaeraceae* | Common taxa |
| OTU 313 | 0.00 | 0.00 | 0.05 | 0.23 | 0.00 | 0.26 | 0.00 | 0.09 | 0.00 | 0.08 | 0.16 | 0.10 | *Proteobacteria* | *Gammaproteobacteria* | *Aeromonadales* | *Aeromonadaceae* | Common taxa |
| OTU 314 | 0.00 | 0.17 | 0.38 | 0.14 | 0.00 | 0.00 | 0.18 | 0.00 | 0.00 | 0.08 | 0.00 | 0.00 | *Proteobacteria* | *Betaproteobacteria* | *Nitrosomonadales* | *Nitrosomonadaceae* | Common taxa |
| OTU 315 | 0.12 | 0.00 | 0.24 | 0.00 | 0.00 | 0.00 | 0.00 | 0.09 | 0.33 | 0.04 | 0.05 | 0.10 | *Planctomycetes* | *Planctomycetia* | *Planctomycetales* | *Planctomycetaceae* | Common taxa |
| OTU 316 | 0.06 | 0.29 | 0.00 | 0.00 | 0.00 | 0.00 | 0.00 | 0.00 | 0.14 | 0.40 | 0.05 | 0.00 | *Parcubacteria* | *Parcubacteria_genera_incertae_sedis* | | | Common taxa |
| OTU 318 | 0.50 | 0.00 | 0.00 | 0.27 | 0.05 | 0.00 | 0.00 | 0.00 | 0.00 | 0.04 | 0.22 | 0.00 | *Proteobacteria* | *Alphaproteobacteria* | *Rhodobacterales* | *Rhodobacteraceae* | Common taxa |
| OTU 320 | 0.00 | 0.17 | 0.57 | 0.00 | 0.00 | 0.20 | 0.00 | 0.00 | 0.00 | 0.00 | 0.00 | 0.05 | *Proteobacteria* | *Gammaproteobacteria* | *Oceanospirillales* | *Hahellaceae* | Common taxa |
| OTU 321 | 0.00 | 0.23 | 0.38 | 0.00 | 0.00 | 0.00 | 0.04 | 0.09 | 0.19 | 0.04 | 0.00 | 0.00 | *Proteobacteria* | *Alphaproteobacteria* | *Rhizobiales* | *Methylocystaceae* | Common taxa |
| OTU 322 | 0.00 | 0.00 | 0.52 | 0.05 | 0.00 | 0.00 | 0.00 | 0.09 | 0.24 | 0.04 | 0.00 | 0.00 | *Proteobacteria* | *Gammaproteobacteria* | *Chromatiales* | *Ectothiorhodospiraceae* | Common taxa |
| OTU 323 | 0.00 | 0.00 | 0.10 | 0.05 | 0.16 | 0.05 | 0.00 | 0.00 | 0.05 | 0.28 | 0.22 | 0.05 | *Proteobacteria* | *Betaproteobacteria* | *Burkholderiales* | *Burkholderiales_incertae_sedis* | Common taxa |
| OTU 325 | 0.00 | 0.00 | 0.33 | 0.18 | 0.00 | 0.10 | 0.04 | 0.05 | 0.09 | 0.08 | 0.00 | 0.00 | *Proteobacteria* | *Gammaproteobacteria* | *Oceanospirillales* | *Hahellaceae* | Common taxa |
| OTU 326 | 0.00 | 0.40 | 0.24 | 0.00 | 0.00 | 0.15 | 0.00 | 0.05 | 0.05 | 0.08 | 0.00 | 0.00 | *Bacteroidetes* | *Cytophagia* | *Cytophagales* | *Flammeovirgaceae* | Common taxa |
| OTU 328 | 0.00 | 0.06 | 0.00 | 0.41 | 0.11 | 0.20 | 0.00 | 0.00 | 0.00 | 0.04 | 0.05 | 0.05 | *Proteobacteria* | *Betaproteobacteria* | *Burkholderiales* | *Burkholderiales_incertae_sedis* | Common taxa |
| OTU 329 | 0.43 | 0.40 | 0.00 | 0.00 | 0.00 | 0.00 | 0.04 | 0.00 | 0.00 | 0.00 | 0.00 | 0.20 | *Proteobacteria* | *Betaproteobacteria* | *Rhodocyclales* | *Rhodocyclaceae* | Common taxa |
| OTU 330 | 0.31 | 0.00 | 0.10 | 0.14 | 0.00 | 0.05 | 0.00 | 0.00 | 0.14 | 0.08 | 0.05 | 0.10 | *Proteobacteria* | *Gammaproteobacteria* | *Pseudomonadales* | *Moraxellaceae* | Common taxa |
| OTU 331 | 0.06 | 0.17 | 0.24 | 0.00 | 0.05 | 0.00 | 0.04 | 0.18 | 0.19 | 0.00 | 0.00 | 0.00 | *Actinobacteria* | *Actinobacteria* | *Acidimicrobidae* | *Acidimicrobiales* | Common taxa |
| OTU 332 | 0.00 | 0.06 | 0.05 | 0.18 | 0.05 | 0.10 | 0.00 | 0.05 | 0.00 | 0.00 | 0.38 | 0.10 | *Proteobacteria* | *Betaproteobacteria* | *Rhodocyclales* | *Rhodocyclaceae* | Common taxa |
| OTU 333 | 0.12 | 0.29 | 0.29 | 0.00 | 0.00 | 0.00 | 0.04 | 0.14 | 0.00 | 0.00 | 0.05 | 0.05 | *Proteobacteria* | *Alphaproteobacteria* | *Rhodospirillales* | *Acetobacteraceae* | Common taxa |
| OTU 334 | 0.06 | 0.00 | 0.00 | 0.09 | 0.11 | 0.10 | 0.00 | 0.00 | 0.19 | 0.00 | 0.33 | 0.10 | *Proteobacteria* | *Betaproteobacteria* | *Rhodocyclales* | *Rhodocyclaceae* | Common taxa |
| OTU 335 | 0.00 | 0.06 | 0.52 | 0.18 | 0.00 | 0.00 | 0.09 | 0.05 | 0.00 | 0.00 | 0.00 | 0.00 | *Bacteroidetes* | *Bacteroidia* | *Bacteroidales* | *Marinilabiliaceae* | Common taxa |
| OTU 338 | 0.12 | 0.00 | 0.00 | 0.05 | 0.05 | 0.15 | 0.00 | 0.00 | 0.09 | 0.04 | 0.11 | 0.35 | *Proteobacteria* | *Alphaproteobacteria* | *Sphingomonadales* | *Sphingomonadaceae* | Common taxa |
| OTU 339 | 0.43 | 0.46 | 0.05 | 0.00 | 0.00 | 0.00 | 0.00 | 0.00 | 0.00 | 0.00 | 0.05 | 0.05 | *Proteobacteria* | *Deltaproteobacteria* | *Myxococcales* | *Cystobacterineae* | Common taxa |
| OTU 340 | 0.19 | 0.11 | 0.00 | 0.00 | 0.00 | 0.05 | 0.09 | 0.27 | 0.00 | 0.00 | 0.16 | 0.05 | *Proteobacteria* | *Gammaproteobacteria* | *Chromatiales* | *Chromatiaceae* | Common taxa |
| OTU 341 | 0.25 | 0.29 | 0.00 | 0.00 | 0.00 | 0.00 | 0.00 | 0.00 | 0.14 | 0.16 | 0.11 | 0.00 | *Planctomycetes* | *Planctomycetia* | *Planctomycetales* | *Planctomycetaceae* | Common taxa |
| OTU 342 | 0.00 | 0.00 | 0.10 | 0.00 | 0.16 | 0.41 | 0.04 | 0.00 | 0.09 | 0.04 | 0.05 | 0.00 | *Proteobacteria* | *Gammaproteobacteria* | *Alteromonadales* | *Alteromonadaceae* | Common taxa |
| OTU 343 | 0.06 | 0.23 | 0.05 | 0.00 | 0.00 | 0.00 | 0.00 | 0.45 | 0.05 | 0.00 | 0.00 | 0.05 | *Chloroflexi* | *Anaerolineae* | *Anaerolineales* | *Anaerolineaceae* | Common taxa |
| OTU 344 | 0.00 | 0.57 | 0.24 | 0.00 | 0.00 | 0.00 | 0.04 | 0.09 | 0.00 | 0.00 | 0.00 | 0.00 | *Proteobacteria* | *Deltaproteobacteria* | *Myxococcales* | *Nannocystineae* | Common taxa |
| OTU 349 | 0.06 | 0.17 | 0.14 | 0.23 | 0.00 | 0.00 | 0.00 | 0.00 | 0.00 | 0.04 | 0.00 | 0.25 | *Proteobacteria* | *Betaproteobacteria* | *Rhodocyclales* | *Rhodocyclaceae* | Common taxa |
| OTU 350 | 0.00 | 0.00 | 0.00 | 0.05 | 0.05 | 0.36 | 0.18 | 0.23 | 0.00 | 0.00 | 0.00 | 0.00 | *Nitrospirae* | *Nitrospira* | *Nitrospirales* | *Nitrospiraceae* | Common taxa |
| OTU 352 | 0.00 | 0.00 | 0.38 | 0.05 | 0.32 | 0.05 | 0.00 | 0.05 | 0.00 | 0.00 | 0.00 | 0.05 | *Proteobacteria* | *Betaproteobacteria* | *Burkholderiales* | *Comamonadaceae* | Common taxa |
| OTU 354 | 0.00 | 0.00 | 0.43 | 0.14 | 0.00 | 0.20 | 0.00 | 0.00 | 0.05 | 0.04 | 0.00 | 0.00 | *Proteobacteria* | *Epsilonproteobacteria* | *Campylobacterales* | *Helicobacteraceae* | Common taxa |
| OTU 355 | 0.12 | 0.00 | 0.00 | 0.05 | 0.05 | 0.10 | 0.00 | 0.00 | 0.09 | 0.00 | 0.16 | 0.35 | *Proteobacteria* | *Alphaproteobacteria* | *Rhodobacterales* | *Rhodobacteraceae* | Common taxa |
| OTU 356 | 0.12 | 0.34 | 0.00 | 0.00 | 0.00 | 0.00 | 0.09 | 0.05 | 0.19 | 0.08 | 0.00 | 0.00 | *Acidobacteria* | *Acidobacteria_Gp6* | *Gp6* | | Common taxa |
| OTU 357 | 0.31 | 0.00 | 0.00 | 0.00 | 0.00 | 0.00 | 0.09 | 0.00 | 0.14 | 0.04 | 0.05 | 0.25 | *Proteobacteria* | *Betaproteobacteria* | *Rhodocyclales* | *Rhodocyclaceae* | Common taxa |
| OTU 358 | 0.12 | 0.00 | 0.00 | 0.00 | 0.00 | 0.00 | 0.04 | 0.00 | 0.09 | 0.32 | 0.11 | 0.10 | *Proteobacteria* | *Betaproteobacteria* | *Burkholderiales* | *Burkholderiales_incertae_sedis* | Common taxa |
| OTU 359 | 0.00 | 0.46 | 0.00 | 0.00 | 0.11 | 0.10 | 0.00 | 0.14 | 0.05 | 0.04 | 0.00 | 0.00 | *Proteobacteria* | *Betaproteobacteria* | *Rhodocyclales* | *Rhodocyclaceae* | Common taxa |
| OTU 362 | 0.12 | 0.17 | 0.14 | 0.00 | 0.00 | 0.00 | 0.00 | 0.00 | 0.24 | 0.16 | 0.00 | 0.00 | *Planctomycetes* | *Planctomycetia* | *Planctomycetales* | *Planctomycetaceae* | Common taxa |
| OTU 364 | 0.00 | 0.00 | 0.05 | 0.45 | 0.00 | 0.05 | 0.00 | 0.00 | 0.00 | 0.16 | 0.05 | 0.00 | *Proteobacteria* | *Epsilonproteobacteria* | *Campylobacterales* | *Campylobacteraceae* | Common taxa |
| OTU 371 | 0.00 | 0.11 | 0.05 | 0.00 | 0.00 | 0.00 | 0.00 | 0.00 | 0.33 | 0.24 | 0.00 | 0.05 | *Planctomycetes* | *Planctomycetia* | *Planctomycetales* | *Planctomycetaceae* | Common taxa |
| OTU 372 | 0.00 | 0.00 | 0.00 | 0.14 | 0.00 | 0.41 | 0.09 | 0.05 | 0.00 | 0.00 | 0.05 | 0.10 | *Proteobacteria* | *Deltaproteobacteria* | *Myxococcales* | *Sorangiineae* | Common taxa |
| OTU 373 | 0.06 | 0.23 | 0.00 | 0.00 | 0.00 | 0.15 | 0.00 | 0.32 | 0.09 | 0.00 | 0.00 | 0.00 | *Proteobacteria* | *Deltaproteobacteria* | *Myxococcales* | *Sorangiineae* | Common taxa |
| OTU 374 | 0.00 | 0.00 | 0.00 | 0.27 | 0.26 | 0.20 | 0.09 | 0.00 | 0.00 | 0.00 | 0.00 | 0.00 | *Proteobacteria* | *Gammaproteobacteria* | *Gammaproteobacteria_incertae_sedis* | *Porticoccus* | Common taxa |
| OTU 377 | 0.43 | 0.00 | 0.00 | 0.00 | 0.11 | 0.00 | 0.00 | 0.00 | 0.09 | 0.20 | 0.00 | 0.05 | *Bacteroidetes* | *Sphingobacteriia* | *Sphingobacteriales* | *Saprospiraceae* | Common taxa |
| OTU 380 | 0.00 | 0.00 | 0.29 | 0.00 | 0.00 | 0.00 | 0.04 | 0.00 | 0.00 | 0.00 | 0.38 | 0.15 | *Proteobacteria* | *Alphaproteobacteria* | *Rhodobacterales* | *Rhodobacteraceae* | Common taxa |
| OTU 381 | 0.00 | 0.06 | 0.33 | 0.00 | 0.05 | 0.15 | 0.09 | 0.09 | 0.00 | 0.00 | 0.00 | 0.00 | *Proteobacteria* | *Betaproteobacteria* | *Nitrosomonadales* | *Nitrosomonadaceae* | Common taxa |
| OTU 382 | 0.00 | 0.11 | 0.24 | 0.05 | 0.00 | 0.00 | 0.26 | 0.09 | 0.00 | 0.00 | 0.00 | 0.00 | *Proteobacteria* | *Deltaproteobacteria* | *Desulfobacterales* | *Desulfobacteraceae* | Common taxa |
| OTU 384 | 0.06 | 0.17 | 0.05 | 0.00 | 0.00 | 0.00 | 0.00 | 0.00 | 0.09 | 0.32 | 0.00 | 0.05 | *Bacteroidetes* | *Cytophagia* | *Cytophagales* | *Cytophagaceae* | Common taxa |
| OTU 387 | 0.00 | 0.00 | 0.00 | 0.00 | 0.00 | 0.15 | 0.09 | 0.00 | 0.00 | 0.00 | 0.05 | 0.50 | *Proteobacteria* | *Alphaproteobacteria* | *Sphingomonadales* | *Sphingomonadaceae* | Common taxa |
| OTU 388 | 0.62 | 0.11 | 0.00 | 0.00 | 0.05 | 0.00 | 0.00 | 0.00 | 0.09 | 0.04 | 0.00 | 0.00 | *Proteobacteria* | *Betaproteobacteria* | *Procabacteriales* | *Procabacteriaceae* | Common taxa |
| OTU 391 | 0.06 | 0.00 | 0.00 | 0.00 | 0.00 | 0.10 | 0.00 | 0.00 | 0.09 | 0.08 | 0.00 | 0.45 | *Bacteroidetes* | *Sphingobacteriia* | *Sphingobacteriales* | *Sphingobacteriaceae* | Common taxa |
| OTU 393 | 0.00 | 0.00 | 0.05 | 0.00 | 0.05 | 0.00 | 0.00 | 0.14 | 0.28 | 0.16 | 0.05 | 0.00 | *Proteobacteria* | *Alphaproteobacteria* | *Rhizobiales* | *Hyphomicrobiaceae* | Common taxa |
| OTU 394 | 0.00 | 0.00 | 0.00 | 0.05 | 0.48 | 0.20 | 0.04 | 0.00 | 0.00 | 0.00 | 0.00 | 0.05 | *Actinobacteria* | *Actinobacteria* | *Actinobacteridae* | *Actinomycetales* | Common taxa |
| OTU 395 | 0.00 | 0.00 | 0.05 | 0.05 | 0.00 | 0.00 | 0.13 | 0.18 | 0.14 | 0.16 | 0.00 | 0.00 | *Proteobacteria* | *Gammaproteobacteria* | *Gammaproteobacteria_incertae_sedis* | *Sedimenticola* | Common taxa |
| OTU 396 | 0.06 | 0.00 | 0.10 | 0.00 | 0.00 | 0.00 | 0.00 | 0.00 | 0.28 | 0.16 | 0.16 | 0.00 | *Proteobacteria* | *Betaproteobacteria* | *Rhodocyclales* | *Rhodocyclaceae* | Common taxa |
| OTU 397 | 0.19 | 0.11 | 0.05 | 0.00 | 0.00 | 0.00 | 0.09 | 0.00 | 0.09 | 0.00 | 0.16 | 0.15 | *Proteobacteria* | *Betaproteobacteria* | *Nitrosomonadales* | *Nitrosomonadaceae* | Common taxa |
| OTU 399 | 0.37 | 0.11 | 0.05 | 0.00 | 0.05 | 0.00 | 0.00 | 0.09 | 0.00 | 0.04 | 0.00 | 0.15 | *Proteobacteria* | *Gammaproteobacteria* | *Pseudomonadales* | *Pseudomonadaceae* | Common taxa |
| OTU 400 | 0.06 | 0.06 | 0.29 | 0.23 | 0.00 | 0.00 | 0.00 | 0.09 | 0.00 | 0.04 | 0.00 | 0.00 | *Proteobacteria* | *Gammaproteobacteria* | *Pseudomonadales* | *Pseudomonadaceae* | Common taxa |
| OTU 404 | 0.06 | 0.11 | 0.14 | 0.00 | 0.00 | 0.00 | 0.00 | 0.09 | 0.00 | 0.00 | 0.38 | 0.05 | *Proteobacteria* | *Alphaproteobacteria* | *Rhodobacterales* | *Rhodobacteraceae* | Common taxa |
| OTU 406 | 0.06 | 0.29 | 0.14 | 0.00 | 0.00 | 0.00 | 0.00 | 0.00 | 0.24 | 0.08 | 0.00 | 0.00 | *Proteobacteria* | *Gammaproteobacteria* | *Xanthomonadales* | *Xanthomonadaceae* | Common taxa |
| OTU 407 | 0.00 | 0.00 | 0.00 | 0.00 | 0.53 | 0.00 | 0.00 | 0.00 | 0.00 | 0.04 | 0.11 | 0.10 | *Proteobacteria* | *Betaproteobacteria* | *Burkholderiales* | *Comamonadaceae* | Common taxa |
| OTU 408 | 0.19 | 0.06 | 0.00 | 0.00 | 0.00 | 0.00 | 0.00 | 0.00 | 0.24 | 0.08 | 0.00 | 0.20 | *Acidobacteria* | *Holophagae* | *Holophagales* | *Holophagaceae* | Common taxa |
| OTU 409 | 0.00 | 0.11 | 0.24 | 0.00 | 0.05 | 0.05 | 0.00 | 0.14 | 0.05 | 0.08 | 0.00 | 0.00 | *Bacteroidetes* | *Bacteroidia* | *Bacteroidales* | *Marinilabiaceae* | Common taxa |
| OTU 412 | 0.00 | 0.06 | 0.24 | 0.09 | 0.16 | 0.00 | 0.00 | 0.05 | 0.05 | 0.08 | 0.00 | 0.00 | *Proteobacteria* | *Betaproteobacteria* | *Burkholderiales* | *Comamonadaceae* | Common taxa |
| OTU 414 | 0.12 | 0.00 | 0.00 | 0.14 | 0.00 | 0.05 | 0.04 | 0.00 | 0.00 | 0.00 | 0.00 | 0.40 | *Proteobacteria* | *Betaproteobacteria* | *Burkholderiales* | *Burkholderiales_incertae_sedis* | Common taxa |
| OTU 415 | 0.00 | 0.00 | 0.00 | 0.18 | 0.11 | 0.31 | 0.00 | 0.14 | 0.00 | 0.00 | 0.00 | 0.00 | *Proteobacteria* | *Alphaproteobacteria* | *Rhizobiales* | *Phyllobacteriaceae* | Common taxa |
| OTU 422 | 0.12 | 0.00 | 0.00 | 0.00 | 0.00 | 0.00 | 0.00 | 0.00 | 0.00 | 0.12 | 0.38 | 0.15 | *Bacteroidetes* | *Sphingobacteriia* | *Sphingobacteriales* | *Saprospiraceae* | Common taxa |
| OTU 423 | 0.06 | 0.00 | 0.00 | 0.00 | 0.00 | 0.00 | 0.00 | 0.00 | 0.05 | 0.20 | 0.16 | 0.25 | *Bacteroidetes* | *Sphingobacteriia* | *Sphingobacteriales* | *Chitinophagaceae* | Common taxa |
| OTU 424 | 0.00 | 0.06 | 0.48 | 0.09 | 0.00 | 0.00 | 0.04 | 0.00 | 0.00 | 0.00 | 0.00 | 0.00 | *Proteobacteria* | *Zetaproteobacteria* | *Mariprofundales* | *Mariprofundaceae* | Common taxa |
| OTU 425 | 0.00 | 0.00 | 0.00 | 0.05 | 0.42 | 0.10 | 0.13 | 0.00 | 0.00 | 0.00 | 0.00 | 0.00 | *Bacteroidetes* | *Flavobacteriia* | *Flavobacteriales* | *Flavobacteriaceae* | Common taxa |
| OTU 426 | 0.00 | 0.00 | 0.14 | 0.00 | 0.00 | 0.00 | 0.04 | 0.14 | 0.05 | 0.16 | 0.00 | 0.10 | *Planctomycetes* | *Planctomycetia* | *Planctomycetales* | *Planctomycetaceae* | Common taxa |
| OTU 433 | 0.00 | 0.00 | 0.05 | 0.09 | 0.26 | 0.20 | 0.04 | 0.00 | 0.00 | 0.00 | 0.05 | 0.00 | *Proteobacteria* | *Alphaproteobacteria* | *Rhizobiales* | *Hyphomicrobiaceae* | Common taxa |
| OTU 436 | 0.00 | 0.23 | 0.14 | 0.14 | 0.00 | 0.00 | 0.09 | 0.05 | 0.05 | 0.00 | 0.00 | 0.00 | *Proteobacteria* | *Deltaproteobacteria* | *Myxococcales* | *Sorangiineae* | Common taxa |
| OTU 437 | 0.25 | 0.00 | 0.10 | 0.05 | 0.00 | 0.00 | 0.00 | 0.00 | 0.09 | 0.08 | 0.00 | 0.15 | *Proteobacteria* | *Gammaproteobacteria* | *Pseudomonadales* | *Moraxellaceae* | Common taxa |
| OTU 438 | 0.00 | 0.00 | 0.00 | 0.05 | 0.37 | 0.20 | 0.09 | 0.00 | 0.00 | 0.00 | 0.00 | 0.00 | *Proteobacteria* | *Alphaproteobacteria* | *Sphingomonadales* | *Erythrobacteraceae* | Common taxa |
| OTU 439 | 0.19 | 0.00 | 0.00 | 0.18 | 0.00 | 0.00 | 0.04 | 0.00 | 0.00 | 0.12 | 0.05 | 0.10 | *Proteobacteria* | *Gammaproteobacteria* | *Pseudomonadales* | *Moraxellaceae* | Common taxa |
| OTU 440 | 0.00 | 0.00 | 0.00 | 0.05 | 0.11 | 0.15 | 0.00 | 0.00 | 0.05 | 0.16 | 0.00 | 0.15 | *Proteobacteria* | *Alphaproteobacteria* | *Caulobacterales* | *Caulobacteraceae* | Common taxa |
| OTU 441 | 0.00 | 0.06 | 0.05 | 0.00 | 0.00 | 0.10 | 0.44 | 0.00 | 0.00 | 0.00 | 0.00 | 0.00 | *Proteobacteria* | *Deltaproteobacteria* | *Myxococcales* | *Nannocystineae* | Common taxa |
| OTU 442 | 0.06 | 0.00 | 0.00 | 0.00 | 0.48 | 0.00 | 0.00 | 0.00 | 0.00 | 0.00 | 0.11 | 0.10 | *Proteobacteria* | *Alphaproteobacteria* | *Rhodobacterales* | *Rhodobacteraceae* | Common taxa |
| OTU 446 | 0.62 | 0.00 | 0.00 | 0.00 | 0.00 | 0.05 | 0.00 | 0.09 | 0.05 | 0.00 | 0.00 | 0.00 | *Actinobacteria* | *Actinobacteria* | *Actinobacteridae* | *Actinomycetales* | Common taxa |
| OTU 447 | 0.25 | 0.00 | 0.00 | 0.09 | 0.00 | 0.00 | 0.00 | 0.00 | 0.00 | 0.00 | 0.05 | 0.35 | *Planctomycetes* | *Planctomycetia* | *Planctomycetales* | *Planctomycetaceae* | Common taxa |
| OTU 448 | 0.00 | 0.06 | 0.05 | 0.00 | 0.05 | 0.41 | 0.04 | 0.09 | 0.00 | 0.00 | 0.00 | 0.00 | *Proteobacteria* | *Alphaproteobacteria* | *Rhizobiales* | *Phyllobacteriaceae* | Common taxa |
| OTU 449 | 0.12 | 0.11 | 0.29 | 0.00 | 0.00 | 0.00 | 0.00 | 0.00 | 0.05 | 0.00 | 0.11 | 0.05 | *Proteobacteria* | *Betaproteobacteria* | *Rhodocyclales* | *Rhodocyclaceae* | Common taxa |
| OTU 453 | 0.25 | 0.00 | 0.19 | 0.00 | 0.00 | 0.00 | 0.04 | 0.14 | 0.09 | 0.00 | 0.00 | 0.00 | *Proteobacteria* | *Alphaproteobacteria* | *Rhizobiales* | *Rhizobiales_incertae_sedis* | Common taxa |
| OTU 456 | 0.00 | 0.00 | 0.00 | 0.00 | 0.05 | 0.51 | 0.04 | 0.00 | 0.00 | 0.00 | 0.05 | 0.00 | *Proteobacteria* | *Gammaproteobacteria* | *Oceanospirillales* | *Halomonadaceae* | Common taxa |
| OTU 458 | 0.00 | 0.00 | 0.00 | 0.05 | 0.21 | 0.26 | 0.00 | 0.00 | 0.00 | 0.00 | 0.11 | 0.05 | *Proteobacteria* | *Alphaproteobacteria* | *Rhodobacterales* | *Rhodobacteraceae* | Common taxa |
| OTU 461 | 0.00 | 0.00 | 0.00 | 0.18 | 0.11 | 0.00 | 0.00 | 0.05 | 0.14 | 0.04 | 0.00 | 0.10 | *Bacteroidetes* | *Cytophagia* | *Cytophagales* | *Flammeovirgaceae* | Common taxa |
| OTU 464 | 0.00 | 0.00 | 0.00 | 0.05 | 0.00 | 0.00 | 0.31 | 0.05 | 0.00 | 0.00 | 0.16 | 0.05 | *Proteobacteria* | *Betaproteobacteria* | *Rhodocyclales* | *Rhodocyclaceae* | Common taxa |
| OTU 465 | 0.00 | 0.00 | 0.05 | 0.36 | 0.05 | 0.05 | 0.09 | 0.00 | 0.00 | 0.00 | 0.00 | 0.00 | *Proteobacteria* | *Alphaproteobacteria* | *Eilatimonas* | | Common taxa |
| OTU 468 | 0.06 | 0.00 | 0.00 | 0.00 | 0.05 | 0.00 | 0.00 | 0.05 | 0.24 | 0.00 | 0.11 | 0.15 | *Proteobacteria* | *Alphaproteobacteria* | *Sphingomonadales* | *Sphingomonadaceae* | Common taxa |
| OTU 470 | 0.19 | 0.00 | 0.00 | 0.00 | 0.26 | 0.10 | 0.00 | 0.00 | 0.00 | 0.04 | 0.05 | 0.05 | *Proteobacteria* | *Alphaproteobacteria* | *Sphingomonadales* | *Sphingomonadaceae* | Common taxa |
| OTU 471 | 0.00 | 0.00 | 0.00 | 0.18 | 0.11 | 0.00 | 0.00 | 0.00 | 0.00 | 0.16 | 0.00 | 0.15 | *Bacteroidetes* | *Flavobacteriia* | *Flavobacteriales* | *Flavobacteriaceae* | Common taxa |

| | | | | | | | | | | | | | | | | | |
|---|---|---|---|---|---|---|---|---|---|---|---|---|---|---|---|---|---|
| OTU 475 | 0.00 | 0.06 | 0.33 | 0.05 | 0.00 | 0.00 | 0.00 | 0.00 | 0.09 | 0.08 | 0.00 | 0.00 | *Proteobacteria* | *Betaproteobacteria* | *Rhodocyclales* | *Rhodocyclaceae* | Common taxa |
| OTU 477 | 0.00 | 0.00 | 0.24 | 0.09 | 0.11 | 0.00 | 0.00 | 0.05 | 0.09 | 0.00 | 0.05 | 0.00 | *Proteobacteria* | *Betaproteobacteria* | *Burkholderiales* | *Comamonadaceae* | Common taxa |
| OTU 478 | 0.00 | 0.00 | 0.29 | 0.14 | 0.00 | 0.05 | 0.04 | 0.00 | 0.00 | 0.08 | 0.00 | 0.00 | *Proteobacteria* | *Deltaproteobacteria* | *Myxococcales* | *Sorangiineae* | Common taxa |
| OTU 482 | 0.06 | 0.06 | 0.00 | 0.00 | 0.00 | 0.00 | 0.00 | 0.23 | 0.19 | 0.04 | 0.00 | 0.00 | *Bacteroidetes* | *Sphingobacteriia* | *Sphingobacteriales* | *Saprospiraceae* | Common taxa |
| OTU 484 | 0.12 | 0.00 | 0.00 | 0.00 | 0.11 | 0.00 | 0.00 | 0.00 | 0.14 | 0.20 | 0.00 | 0.00 | *Proteobacteria* | *Gammaproteobacteria* | *Legionellales* | *Legionellaceae* | Common taxa |
| OTU 485 | 0.00 | 0.06 | 0.24 | 0.00 | 0.11 | 0.00 | 0.00 | 0.00 | 0.05 | 0.12 | 0.00 | 0.00 | *Proteobacteria* | *Betaproteobacteria* | *Burkholderiales* | *Comamonadaceae* | Common taxa |
| OTU 487 | 0.00 | 0.00 | 0.00 | 0.00 | 0.16 | 0.10 | 0.00 | 0.00 | 0.00 | 0.04 | 0.22 | 0.10 | *Proteobacteria* | *Betaproteobacteria* | *Rhodocyclales* | *Rhodocyclaceae* | Common taxa |
| OTU 489 | 0.00 | 0.06 | 0.00 | 0.05 | 0.11 | 0.00 | 0.09 | 0.27 | 0.00 | 0.00 | 0.00 | 0.00 | *Bacteroidetes* | *Sphingobacteriia* | *Sphingobacteriales* | *Saprospiraceae* | Common taxa |
| OTU 490 | 0.00 | 0.06 | 0.05 | 0.00 | 0.00 | 0.00 | 0.00 | 0.41 | 0.05 | 0.00 | 0.00 | 0.00 | *Chloroflexi* | *Anaerolineae* | *Anaerolineales* | *Anaerolineaceae* | Common taxa |
| OTU 491 | 0.06 | 0.00 | 0.00 | 0.00 | 0.00 | 0.15 | 0.04 | 0.00 | 0.19 | 0.04 | 0.00 | 0.10 | *Proteobacteria* | *Alphaproteobacteria* | *Sphingomonadales* | *Sphingomonadaceae* | Common taxa |
| OTU 494 | 0.19 | 0.00 | 0.00 | 0.00 | 0.11 | 0.00 | 0.00 | 0.00 | 0.05 | 0.04 | 0.16 | 0.10 | *Bacteroidetes* | *Sphingobacteriia* | *Sphingobacteriales* | *Chitinophagaceae* | Common taxa |
| OTU 495 | 0.00 | 0.17 | 0.00 | 0.00 | 0.00 | 0.00 | 0.00 | 0.00 | 0.05 | 0.00 | 0.33 | 0.10 | *Proteobacteria* | *Betaproteobacteria* | *Gallionellales* | *Gallionellaceae* | Common taxa |
| OTU 496 | 0.37 | 0.00 | 0.00 | 0.00 | 0.00 | 0.05 | 0.00 | 0.00 | 0.19 | 0.04 | 0.00 | 0.00 | *Bacteroidetes* | *Sphingobacteriia* | *Sphingobacteriales* | *Cyclobacteriaceae* | Common taxa |
| OTU 497 | 0.06 | 0.00 | 0.00 | 0.00 | 0.00 | 0.00 | 0.00 | 0.00 | 0.00 | 0.04 | 0.49 | 0.05 | *Verrucomicrobia* | *Opitutae* | *Opitutales* | *Opitutaceae* | Common taxa |
| OTU 499 | 0.06 | 0.00 | 0.00 | 0.00 | 0.00 | 0.00 | 0.00 | 0.00 | 0.00 | 0.28 | 0.11 | 0.10 | *Proteobacteria* | *Deltaproteobacteria* | *Myxococcales* | *Nannocystineae* | Common taxa |
| OTU 501 | 0.00 | 0.11 | 0.14 | 0.00 | 0.00 | 0.00 | 0.00 | 0.00 | 0.24 | 0.08 | 0.00 | 0.00 | *Actinobacteria* | *Actinobacteria* | *Rubrobacteridae* | *Gaiellales* | Common taxa |
| OTU 502 | 0.00 | 0.00 | 0.05 | 0.00 | 0.11 | 0.00 | 0.04 | 0.23 | 0.14 | 0.00 | 0.00 | 0.00 | *Proteobacteria* | *Betaproteobacteria* | *Burkholderiales* | *Burkholderiales_incertae_sedis* | Common taxa |
| OTU 503 | 0.00 | 0.00 | 0.10 | 0.05 | 0.00 | 0.05 | 0.00 | 0.00 | 0.14 | 0.20 | 0.00 | 0.00 | *Proteobacteria* | *Alphaproteobacteria* | *Kiloniellales* | *Kiloniellaceae* | Common taxa |
| OTU 506 | 0.00 | 0.00 | 0.00 | 0.05 | 0.05 | 0.15 | 0.00 | 0.09 | 0.00 | 0.00 | 0.27 | 0.00 | *Proteobacteria* | *Gammaproteobacteria* | *Aeromonadales* | *Aeromonadaceae* | Common taxa |
| OTU 509 | 0.31 | 0.00 | 0.05 | 0.00 | 0.00 | 0.00 | 0.00 | 0.00 | 0.19 | 0.04 | 0.00 | 0.05 | *Proteobacteria* | *Betaproteobacteria* | *Rhodocyclales* | *Rhodocyclaceae* | Common taxa |
| OTU 512 | 0.00 | 0.06 | 0.00 | 0.00 | 0.05 | 0.00 | 0.04 | 0.23 | 0.00 | 0.04 | 0.00 | 0.15 | *Proteobacteria* | *Betaproteobacteria* | *Burkholderiales* | *Comamonadaceae* | Common taxa |
| OTU 514 | 0.19 | 0.00 | 0.00 | 0.00 | 0.00 | 0.00 | 0.00 | 0.05 | 0.00 | 0.00 | 0.22 | 0.20 | *Proteobacteria* | *Betaproteobacteria* | *Rhodocyclales* | *Rhodocyclaceae* | Common taxa |
| OTU 517 | 0.06 | 0.00 | 0.00 | 0.00 | 0.05 | 0.00 | 0.00 | 0.00 | 0.00 | 0.24 | 0.22 | 0.00 | *Proteobacteria* | *Betaproteobacteria* | *Burkholderiales* | *Comamonadaceae* | Common taxa |
| OTU 522 | 0.00 | 0.00 | 0.33 | 0.00 | 0.00 | 0.00 | 0.04 | 0.05 | 0.05 | 0.04 | 0.00 | 0.00 | *Planctomycetes* | *Planctomycetia* | *Planctomycetales* | *Planctomycetaceae* | Common taxa |
| OTU 523 | 0.00 | 0.00 | 0.10 | 0.18 | 0.00 | 0.00 | 0.00 | 0.14 | 0.09 | 0.00 | 0.00 | 0.00 | *Proteobacteria* | *Alphaproteobacteria* | *Rhodobacterales* | *Rhodobacteraceae* | Common taxa |
| OTU 524 | 0.00 | 0.06 | 0.05 | 0.05 | 0.16 | 0.20 | 0.00 | 0.00 | 0.00 | 0.00 | 0.00 | 0.05 | *Proteobacteria* | *Deltaproteobacteria* | *Desulfuromonadales* | *Geobacteraceae* | Common taxa |
| OTU 525 | 0.00 | 0.00 | 0.00 | 0.23 | 0.05 | 0.00 | 0.00 | 0.00 | 0.09 | 0.00 | 0.11 | 0.05 | *Proteobacteria* | *Betaproteobacteria* | *Rhodocyclales* | *Rhodocyclaceae* | Common taxa |
| OTU 527 | 0.00 | 0.00 | 0.14 | 0.00 | 0.00 | 0.00 | 0.18 | 0.05 | 0.14 | 0.00 | 0.00 | 0.00 | *Proteobacteria* | *Alphaproteobacteria* | *Rhodospirillales* | *Rhodospirillaceae* | Common taxa |
| OTU 528 | 0.25 | 0.23 | 0.00 | 0.00 | 0.00 | 0.05 | 0.00 | 0.00 | 0.05 | 0.00 | 0.00 | 0.05 | *Bacteroidetes* | *Sphingobacteriia* | *Sphingobacteriales* | *Saprospiraceae* | Common taxa |
| OTU 532 | 0.12 | 0.00 | 0.05 | 0.00 | 0.37 | 0.00 | 0.00 | 0.00 | 0.00 | 0.04 | 0.00 | 0.00 | *Proteobacteria* | *Betaproteobacteria* | *Rhodocyclales* | *Rhodocyclaceae* | Common taxa |
| OTU 533 | 0.06 | 0.00 | 0.00 | 0.05 | 0.05 | 0.05 | 0.00 | 0.00 | 0.00 | 0.00 | 0.27 | 0.10 | *Proteobacteria* | *Betaproteobacteria* | *Burkholderiales* | *Comamonadaceae* | Common taxa |
| OTU 534 | 0.12 | 0.06 | 0.00 | 0.00 | 0.00 | 0.05 | 0.00 | 0.05 | 0.00 | 0.08 | 0.22 | 0.00 | *Proteobacteria* | *Betaproteobacteria* | *Rhodocyclales* | *Rhodocyclaceae* | Common taxa |
| OTU 535 | 0.00 | 0.00 | 0.00 | 0.00 | 0.11 | 0.00 | 0.00 | 0.14 | 0.05 | 0.08 | 0.00 | 0.15 | *Proteobacteria* | *Gammaproteobacteria* | *Pseudomonadales* | *Pseudomonadaceae* | Common taxa |
| OTU 536 | 0.00 | 0.46 | 0.05 | 0.00 | 0.00 | 0.00 | 0.00 | 0.05 | 0.05 | 0.00 | 0.00 | 0.00 | *Proteobacteria* | *Gammaproteobacteria* | *Xanthomonadales* | *Xanthomonadaceae* | Common taxa |
| OTU 537 | 0.06 | 0.34 | 0.10 | 0.00 | 0.00 | 0.00 | 0.00 | 0.00 | 0.00 | 0.08 | 0.00 | 0.00 | *Bacteroidetes* | *Flavobacteriia* | *Flavobacteriales* | *Flavobacteriaceae* | Common taxa |
| OTU 538 | 0.00 | 0.17 | 0.00 | 0.09 | 0.00 | 0.10 | 0.04 | 0.14 | 0.00 | 0.00 | 0.00 | 0.00 | *Proteobacteria* | *Deltaproteobacteria* | *Myxococcales* | *Sorangiineae* | Common taxa |
| OTU 539 | 0.00 | 0.00 | 0.05 | 0.14 | 0.00 | 0.05 | 0.00 | 0.00 | 0.09 | 0.12 | 0.00 | 0.05 | *Proteobacteria* | *Gammaproteobacteria* | *Pseudomonadales* | *Moraxellaceae* | Common taxa |
| OTU 541 | 0.00 | 0.00 | 0.00 | 0.05 | 0.00 | 0.00 | 0.00 | 0.27 | 0.00 | 0.08 | 0.11 | 0.00 | *Proteobacteria* | *Betaproteobacteria* | *Burkholderiales* | *Comamonadaceae* | Common taxa |
| OTU 542 | 0.25 | 0.00 | 0.00 | 0.00 | 0.26 | 0.00 | 0.00 | 0.00 | 0.00 | 0.04 | 0.00 | 0.05 | *Aquificae* | *Aquificae* | *Aquificales* | *Hydrogenothermaceae* | Common taxa |
| OTU 544 | 0.00 | 0.00 | 0.10 | 0.00 | 0.00 | 0.00 | 0.09 | 0.14 | 0.00 | 0.08 | 0.05 | 0.05 | *Proteobacteria* | *Gammaproteobacteria* | *Aeromonadales* | *Aeromonadaceae* | Common taxa |
| OTU 545 | 0.12 | 0.00 | 0.05 | 0.23 | 0.00 | 0.00 | 0.00 | 0.00 | 0.05 | 0.08 | 0.00 | 0.00 | *Proteobacteria* | *Gammaproteobacteria* | *Pseudomonadales* | *Pseudomonadaceae* | Common taxa |
| OTU 547 | 0.00 | 0.00 | 0.05 | 0.05 | 0.21 | 0.05 | 0.00 | 0.18 | 0.00 | 0.00 | 0.00 | 0.00 | *Planctomycetes* | *Planctomycetia* | *Planctomycetales* | *Planctomycetaceae* | Common taxa |
| OTU 549 | 0.06 | 0.17 | 0.10 | 0.05 | 0.00 | 0.00 | 0.00 | 0.00 | 0.00 | 0.04 | 0.16 | 0.00 | *Proteobacteria* | *Betaproteobacteria* | *Rhodocyclales* | *Rhodocyclaceae* | Common taxa |
| OTU 550 | 0.06 | 0.00 | 0.10 | 0.00 | 0.00 | 0.10 | 0.00 | 0.00 | 0.14 | 0.08 | 0.00 | 0.05 | *Proteobacteria* | *Alphaproteobacteria* | *Rhizobiales* | *Methylobacteriaceae* | Common taxa |
| OTU 551 | 0.12 | 0.00 | 0.00 | 0.00 | 0.00 | 0.00 | 0.00 | 0.00 | 0.00 | 0.04 | 0.11 | 0.30 | *Proteobacteria* | *Gammaproteobacteria* | *Xanthomonadales* | *Xanthomonadaceae* | Common taxa |
| OTU 555 | 0.06 | 0.06 | 0.00 | 0.05 | 0.00 | 0.00 | 0.00 | 0.00 | 0.05 | 0.00 | 0.38 | 0.00 | *Proteobacteria* | *Betaproteobacteria* | *Burkholderiales* | *Comamonadaceae* | Common taxa |
| OTU 558 | 0.00 | 0.00 | 0.00 | 0.00 | 0.11 | 0.05 | 0.00 | 0.00 | 0.05 | 0.12 | 0.00 | 0.20 | *Bacteroidetes* | *Flavobacteriia* | *Flavobacteriales* | *Flavobacteriaceae* | Common taxa |
| OTU 559 | 0.00 | 0.00 | 0.00 | 0.14 | 0.21 | 0.00 | 0.09 | 0.00 | 0.00 | 0.08 | 0.00 | 0.00 | *Bacteroidetes* | *Cytophagia* | *Cytophagales* | *Flammeovirgaceae* | Common taxa |
| OTU 560 | 0.25 | 0.06 | 0.00 | 0.00 | 0.00 | 0.05 | 0.00 | 0.00 | 0.14 | 0.04 | 0.00 | 0.00 | *Proteobacteria* | *Alphaproteobacteria* | *Rhizobiales* | *Methylocystaceae* | Common taxa |
| OTU 563 | 0.00 | 0.00 | 0.19 | 0.00 | 0.05 | 0.05 | 0.13 | 0.00 | 0.05 | 0.00 | 0.00 | 0.00 | *Proteobacteria* | *Betaproteobacteria* | *Rhodocyclales* | *Rhodocyclaceae* | Common taxa |
| OTU 564 | 0.12 | 0.06 | 0.29 | 0.00 | 0.00 | 0.00 | 0.00 | 0.00 | 0.05 | 0.00 | 0.00 | 0.00 | *Proteobacteria* | *Gammaproteobacteria* | *Chromatiales* | *Ectothiorhodospiraceae* | Common taxa |
| OTU 566 | 0.00 | 0.11 | 0.00 | 0.00 | 0.00 | 0.00 | 0.09 | 0.00 | 0.09 | 0.08 | 0.00 | 0.10 | *Proteobacteria* | *Betaproteobacteria* | *Rhodocyclales* | *Rhodocyclaceae* | Common taxa |
| OTU 569 | 0.00 | 0.06 | 0.33 | 0.00 | 0.00 | 0.00 | 0.04 | 0.00 | 0.05 | 0.00 | 0.00 | 0.00 | *Proteobacteria* | *Betaproteobacteria* | *Rhodocyclales* | *Rhodocyclaceae* | Common taxa |
| OTU 571 | 0.00 | 0.06 | 0.05 | 0.14 | 0.05 | 0.15 | 0.00 | 0.00 | 0.00 | 0.00 | 0.00 | 0.05 | *Planctomycetes* | *Planctomycetia* | *Planctomycetales* | *Planctomycetaceae* | Common taxa |
| OTU 572 | 0.00 | 0.00 | 0.00 | 0.00 | 0.00 | 0.00 | 0.04 | 0.23 | 0.05 | 0.04 | 0.00 | 0.10 | *Proteobacteria* | *Gammaproteobacteria* | *Pseudomonadales* | *Pseudomonadaceae* | Common taxa |
| OTU 576 | 0.00 | 0.00 | 0.05 | 0.09 | 0.05 | 0.00 | 0.18 | 0.05 | 0.05 | 0.00 | 0.00 | 0.00 | *Proteobacteria* | *Gammaproteobacteria* | *Thiotrichales* | *Thiotrichaceae* | Common taxa |
| OTU 577 | 0.25 | 0.06 | 0.00 | 0.00 | 0.00 | 0.00 | 0.09 | 0.00 | 0.14 | 0.00 | 0.00 | 0.00 | *Proteobacteria* | *Betaproteobacteria* | *Rhodocyclales* | *Rhodocyclaceae* | Common taxa |
| OTU 578 | 0.00 | 0.00 | 0.00 | 0.05 | 0.05 | 0.05 | 0.31 | 0.00 | 0.00 | 0.00 | 0.00 | 0.00 | *Latescibacteria* | *Latescibacteria_genera_incertae_sedis* | | | Common taxa |
| OTU 579 | 0.12 | 0.00 | 0.00 | 0.00 | 0.05 | 0.00 | 0.00 | 0.00 | 0.05 | 0.04 | 0.27 | 0.00 | *Proteobacteria* | *Gammaproteobacteria* | *Xanthomonadales* | *Xanthomonadaceae* | Common taxa |
| OTU 580 | 0.00 | 0.00 | 0.00 | 0.00 | 0.00 | 0.00 | 0.04 | 0.05 | 0.05 | 0.00 | 0.27 | 0.10 | *Proteobacteria* | *Betaproteobacteria* | *Nitrosomonadales* | *Nitrosomonadaceae* | Common taxa |
| OTU 583 | 0.19 | 0.23 | 0.05 | 0.00 | 0.00 | 0.00 | 0.00 | 0.00 | 0.00 | 0.00 | 0.00 | 0.10 | *Chloroflexi* | *Anaerolineae* | *Anaerolineales* | *Anaerolineaceae* | Common taxa |
| OTU 584 | 0.06 | 0.00 | 0.00 | 0.00 | 0.00 | 0.00 | 0.00 | 0.00 | 0.00 | 0.04 | 0.11 | 0.30 | *Planctomycetes* | *Planctomycetia* | *Planctomycetales* | *Planctomycetaceae* | Common taxa |
| OTU 585 | 0.12 | 0.11 | 0.14 | 0.00 | 0.00 | 0.00 | 0.00 | 0.09 | 0.00 | 0.00 | 0.00 | 0.05 | *Ignavibacteriae* | *Ignavibacteria* | *Ignavibacteriales* | *Ignavibacteriaceae* | Common taxa |
| OTU 587 | 0.00 | 0.00 | 0.05 | 0.09 | 0.11 | 0.15 | 0.04 | 0.00 | 0.05 | 0.00 | 0.00 | 0.00 | *Proteobacteria* | *Deltaproteobacteria* | *Myxococcales* | *Cystobacterineae* | Common taxa |
| OTU 588 | 0.06 | 0.17 | 0.14 | 0.00 | 0.00 | 0.05 | 0.04 | 0.00 | 0.00 | 0.00 | 0.00 | 0.05 | *Proteobacteria* | *Gammaproteobacteria* | *Gammaproteobacteria_incertae_sedis* | *Thiohalomonas* | Common taxa |
| OTU 591 | 0.00 | 0.00 | 0.00 | 0.18 | 0.11 | 0.10 | 0.00 | 0.00 | 0.00 | 0.04 | 0.00 | 0.05 | *Proteobacteria* | *Betaproteobacteria* | *Burkholderiales* | *Burkholderiales_incertae_sedis* | Common taxa |
| OTU 594 | 0.00 | 0.00 | 0.05 | 0.00 | 0.00 | 0.00 | 0.09 | 0.23 | 0.05 | 0.00 | 0.05 | 0.00 | *Proteobacteria* | *Betaproteobacteria* | *Rhodocyclales* | *Rhodocyclaceae* | Common taxa |
| OTU 596 | 0.37 | 0.06 | 0.05 | 0.00 | 0.00 | 0.00 | 0.00 | 0.05 | 0.00 | 0.00 | 0.00 | 0.05 | *Proteobacteria* | *Betaproteobacteria* | *Burkholderiales* | *Burkholderiales_incertae_sedis* | Common taxa |
| OTU 597 | 0.06 | 0.00 | 0.00 | 0.05 | 0.00 | 0.26 | 0.00 | 0.00 | 0.00 | 0.04 | 0.11 | 0.00 | *Bacteroidetes* | *Flavobacteriia* | *Flavobacteriales* | *Cryomorphaceae* | Common taxa |
| OTU 598 | 0.00 | 0.00 | 0.05 | 0.05 | 0.00 | 0.15 | 0.00 | 0.00 | 0.24 | 0.00 | 0.00 | 0.00 | *Bacteroidetes* | *Cytophagia* | *Cytophagales* | *Cytophagaceae* | Common taxa |
| OTU 600 | 0.00 | 0.06 | 0.14 | 0.00 | 0.00 | 0.00 | 0.00 | 0.23 | 0.05 | 0.00 | 0.00 | 0.00 | *Proteobacteria* | *Deltaproteobacteria* | *Myxococcales* | *Sorangiineae* | Common taxa |
| OTU 609 | 0.06 | 0.06 | 0.00 | 0.00 | 0.00 | 0.00 | 0.00 | 0.00 | 0.00 | 0.04 | 0.05 | 0.30 | *Bacteroidetes* | *Bacteroidetes_incertae_sedis* | *Ohtaekwangia* | | Common taxa |
| OTU 612 | 0.06 | 0.00 | 0.29 | 0.00 | 0.00 | 0.00 | 0.00 | 0.05 | 0.05 | 0.00 | 0.00 | 0.05 | *Proteobacteria* | *Alphaproteobacteria* | *Rhodospirillales* | *Acetobacteraceae* | Common taxa |
| OTU 613 | 0.00 | 0.00 | 0.00 | 0.00 | 0.00 | 0.00 | 0.00 | 0.05 | 0.00 | 0.04 | 0.38 | 0.05 | *Proteobacteria* | *Betaproteobacteria* | *Burkholderiales* | *Comamonadaceae* | Common taxa |
| OTU 614 | 0.00 | 0.00 | 0.00 | 0.05 | 0.16 | 0.15 | 0.00 | 0.00 | 0.05 | 0.00 | 0.00 | 0.05 | *Proteobacteria* | *Deltaproteobacteria* | *Myxococcales* | *Sorangiineae* | Common taxa |
| OTU 619 | 0.00 | 0.00 | 0.00 | 0.00 | 0.11 | 0.00 | 0.00 | 0.05 | 0.09 | 0.00 | 0.11 | 0.10 | *Proteobacteria* | *Betaproteobacteria* | *Rhodocyclales* | *Rhodocyclaceae* | Common taxa |
| OTU 620 | 0.37 | 0.00 | 0.00 | 0.00 | 0.05 | 0.00 | 0.00 | 0.00 | 0.05 | 0.00 | 0.00 | 0.05 | *Proteobacteria* | *Deltaproteobacteria* | *Myxococcales* | *Sorangiineae* | Common taxa |
| OTU 624 | 0.19 | 0.00 | 0.00 | 0.00 | 0.00 | 0.00 | 0.00 | 0.00 | 0.14 | 0.08 | 0.05 | 0.00 | *Proteobacteria* | *Betaproteobacteria* | *Rhodocyclales* | *Rhodocyclaceae* | Common taxa |
| OTU 629 | 0.00 | 0.00 | 0.00 | 0.00 | 0.05 | 0.00 | 0.04 | 0.00 | 0.24 | 0.08 | 0.00 | 0.00 | *Proteobacteria* | *Deltaproteobacteria* | *Bdellovibrionales* | *Bdellovibrionaceae* | Common taxa |
| OTU 632 | 0.25 | 0.00 | 0.00 | 0.00 | 0.00 | 0.05 | 0.00 | 0.00 | 0.09 | 0.08 | 0.00 | 0.00 | *Bacteroidetes* | *Sphingobacteriia* | *Sphingobacteriales* | *Cyclobacteriaceae* | Common taxa |
| OTU 634 | 0.00 | 0.00 | 0.05 | 0.00 | 0.11 | 0.10 | 0.13 | 0.00 | 0.05 | 0.00 | 0.00 | 0.00 | *Proteobacteria* | *Alphaproteobacteria* | *Alphaproteobacteria_incertae_sedis* | *Geminicoccus* | Common taxa |
| OTU 636 | 0.06 | 0.06 | 0.00 | 0.00 | 0.00 | 0.00 | 0.00 | 0.23 | 0.00 | 0.08 | 0.00 | 0.00 | *Proteobacteria* | *Betaproteobacteria* | *Rhodocyclales* | *Rhodocyclaceae* | Common taxa |
| OTU 637 | 0.00 | 0.00 | 0.00 | 0.00 | 0.00 | 0.00 | 0.00 | 0.09 | 0.19 | 0.04 | 0.05 | 0.05 | *Chloroflexi* | *Anaerolineae* | *Anaerolineales* | *Anaerolineaceae* | Common taxa |
| OTU 638 | 0.00 | 0.06 | 0.00 | 0.00 | 0.00 | 0.15 | 0.00 | 0.00 | 0.09 | 0.00 | 0.05 | 0.10 | *Verrucomicrobia* | *Opitutae* | *Opitutales* | *Opitutaceae* | Common taxa |
| OTU 640 | 0.00 | 0.00 | 0.00 | 0.05 | 0.11 | 0.00 | 0.09 | 0.05 | 0.14 | 0.00 | 0.00 | 0.00 | *Proteobacteria* | *Betaproteobacteria* | *Burkholderiales* | *Burkholderiaceae* | Common taxa |
| OTU 641 | 0.00 | 0.00 | 0.05 | 0.14 | 0.16 | 0.05 | 0.00 | 0.00 | 0.00 | 0.00 | 0.00 | 0.05 | *Actinobacteria* | *Actinobacteria* | *Actinobacteridae* | *Actinomycetales* | Common taxa |
| OTU 643 | 0.06 | 0.00 | 0.14 | 0.00 | 0.00 | 0.00 | 0.00 | 0.05 | 0.09 | 0.00 | 0.00 | 0.10 | *Proteobacteria* | *Betaproteobacteria* | *Rhodocyclales* | *Rhodocyclaceae* | Common taxa |
| OTU 644 | 0.00 | 0.00 | 0.00 | 0.00 | 0.00 | 0.00 | 0.00 | 0.00 | 0.09 | 0.12 | 0.16 | 0.05 | *Proteobacteria* | *Betaproteobacteria* | *Rhodocyclales* | *Rhodocyclaceae* | Common taxa |
| OTU 654 | 0.00 | 0.00 | 0.00 | 0.00 | 0.00 | 0.15 | 0.00 | 0.00 | 0.05 | 0.16 | 0.00 | 0.05 | *Proteobacteria* | *Deltaproteobacteria* | *Myxococcales* | *Cystobacterineae* | Common taxa |
| OTU 655 | 0.12 | 0.00 | 0.00 | 0.00 | 0.00 | 0.05 | 0.00 | 0.00 | 0.00 | 0.00 | 0.05 | 0.25 | *Bacteroidetes* | *Bacteroidia* | *Bacteroidales* | *Marinilabiliaceae* | Common taxa |
| OTU 656 | 0.06 | 0.00 | 0.00 | 0.00 | 0.00 | 0.00 | 0.00 | 0.00 | 0.00 | 0.04 | 0.05 | 0.30 | *Verrucomicrobia* | *Opitutae* | *Opitutales* | *Opitutaceae* | Common taxa |
| OTU 658 | 0.00 | 0.29 | 0.05 | 0.00 | 0.00 | 0.00 | 0.00 | 0.05 | 0.00 | 0.08 | 0.00 | 0.00 | *Proteobacteria* | *Alphaproteobacteria* | *Rhizobiales* | *Methylocystaceae* | Common taxa |
| OTU 664 | 0.06 | 0.00 | 0.00 | 0.00 | 0.00 | 0.05 | 0.00 | 0.00 | 0.00 | 0.04 | 0.05 | 0.25 | *Proteobacteria* | *Betaproteobacteria* | *Burkholderiales* | *Burkholderiales_incertae_sedis* | Common taxa |
| OTU 665 | 0.06 | 0.06 | 0.10 | 0.00 | 0.00 | 0.20 | 0.00 | 0.00 | 0.00 | 0.00 | 0.05 | 0.00 | *Proteobacteria* | *Alphaproteobacteria* | *Rhodobacterales* | *Rhodobacteraceae* | Common taxa |
| OTU 668 | 0.00 | 0.00 | 0.00 | 0.00 | 0.00 | 0.00 | 0.04 | 0.09 | 0.09 | 0.12 | 0.00 | 0.00 | *Proteobacteria* | *Alphaproteobacteria* | *Sphingomonadales* | *Sphingomonadaceae* | Common taxa |
| OTU 669 | 0.00 | 0.06 | 0.19 | 0.05 | 0.00 | 0.00 | 0.00 | 0.00 | 0.05 | 0.00 | 0.00 | 0.05 | *Bacteroidetes* | *Bacteroidetes_incertae_sedis* | *Ohtaekwangia* | | Common taxa |
| OTU 670 | 0.06 | 0.06 | 0.00 | 0.00 | 0.00 | 0.20 | 0.04 | 0.00 | 0.00 | 0.00 | 0.00 | 0.05 | *Firmicutes* | *Clostridia* | *Clostridiales* | *Ruminococcaceae* | Common taxa |
| OTU 671 | 0.00 | 0.00 | 0.14 | 0.00 | 0.11 | 0.05 | 0.04 | 0.05 | 0.00 | 0.00 | 0.00 | 0.00 | *Bacteroidetes* | *Bacteroidetes_incertae_sedis* | *Ohtaekwangia* | | Common taxa |
| OTU 672 | 0.00 | 0.00 | 0.00 | 0.00 | 0.00 | 0.05 | 0.09 | 0.18 | 0.05 | 0.00 | 0.00 | 0.00 | *Nitrospirae* | *Nitrospira* | *Nitrospirales* | *Nitrospiraceae* | Common taxa |
| OTU 676 | 0.00 | 0.17 | 0.00 | 0.00 | 0.00 | 0.00 | 0.00 | 0.05 | 0.09 | 0.04 | 0.00 | 0.05 | *Actinobacteria* | *Actinobacteria* | *Acidimicrobidae* | *Acidimicrobiales* | Common taxa |
| OTU 677 | 0.06 | 0.11 | 0.00 | 0.00 | 0.00 | 0.00 | 0.00 | 0.00 | 0.19 | 0.04 | 0.00 | 0.00 | *Proteobacteria* | *Deltaproteobacteria* | *Myxococcales* | *Nannocystineae* | Common taxa |
| OTU 679 | 0.00 | 0.00 | 0.00 | 0.00 | 0.00 | 0.20 | 0.00 | 0.05 | 0.05 | 0.08 | 0.00 | 0.00 | *Proteobacteria* | *Betaproteobacteria* | *Neisseriales* | *Neisseriaceae* | Common taxa |
| OTU 680 | 0.00 | 0.00 | 0.00 | 0.00 | 0.00 | 0.05 | 0.00 | 0.14 | 0.09 | 0.08 | 0.00 | 0.00 | *Proteobacteria* | *Betaproteobacteria* | *Burkholderiales* | *Burkholderiaceae* | Common taxa |
| OTU 684 | 0.06 | 0.00 | 0.00 | 0.00 | 0.00 | 0.05 | 0.13 | 0.00 | 0.05 | 0.08 | 0.00 | 0.00 | *Proteobacteria* | *Betaproteobacteria* | *Rhodocyclales* | *Rhodocyclaceae* | Common taxa |
| OTU 688 | 0.00 | 0.00 | 0.00 | 0.00 | 0.16 | 0.10 | 0.00 | 0.00 | 0.00 | 0.00 | 0.05 | 0.10 | *Firmicutes* | *Clostridia* | *Clostridiales* | *Clostridiaceae 1* | Common taxa |
| OTU 691 | 0.00 | 0.06 | 0.00 | 0.00 | 0.00 | 0.00 | 0.09 | 0.00 | 0.05 | 0.00 | 0.22 | 0.00 | *Proteobacteria* | *Deltaproteobacteria* | *Myxococcales* | *Sorangiineae* | Common taxa |
| OTU 693 | 0.00 | 0.00 | 0.05 | 0.00 | 0.00 | 0.00 | 0.00 | 0.05 | 0.09 | 0.00 | 0.22 | 0.00 | *Proteobacteria* | *Betaproteobacteria* | *Rhodocyclales* | *Rhodocyclaceae* | Common taxa |
| OTU 697 | 0.00 | 0.00 | 0.24 | 0.00 | 0.00 | 0.00 | 0.04 | 0.05 | 0.00 | 0.04 | 0.00 | 0.00 | *Proteobacteria* | *Betaproteobacteria* | *Nitrosomonadales* | *Nitrosomonadaceae* | Common taxa |
| OTU 702 | 0.00 | 0.00 | 0.05 | 0.00 | 0.00 | 0.05 | 0.00 | 0.23 | 0.05 | 0.00 | 0.00 | 0.00 | *Proteobacteria* | *Epsilonproteobacteria* | *Campylobacterales* | *Campylobacteraceae* | Common taxa |
| OTU 703 | 0.00 | 0.17 | 0.10 | 0.05 | 0.00 | 0.00 | 0.00 | 0.00 | 0.05 | 0.00 | 0.00 | 0.05 | *Proteobacteria* | *Alphaproteobacteria* | *Rhodobacterales* | *Rhodobacteraceae* | Common taxa |
| OTU 707 | 0.00 | 0.00 | 0.00 | 0.00 | 0.00 | 0.00 | 0.04 | 0.09 | 0.09 | 0.04 | 0.00 | 0.10 | *Proteobacteria* | *Betaproteobacteria* | *Rhodocyclales* | *Rhodocyclaceae* | Common taxa |
| OTU 709 | 0.00 | 0.00 | 0.00 | 0.00 | 0.21 | 0.05 | 0.00 | 0.00 | 0.00 | 0.00 | 0.05 | 0.10 | *Firmicutes* | *Clostridia* | *Clostridiales* | *Ruminococcaceae* | Common taxa |
| OTU 718 | 0.12 | 0.00 | 0.00 | 0.09 | 0.00 | 0.00 | 0.00 | 0.00 | 0.00 | 0.04 | 0.05 | 0.10 | *Proteobacteria* | *Betaproteobacteria* | *Rhodocyclales* | *Rhodocyclaceae* | Common taxa |
| OTU 720 | 0.06 | 0.17 | 0.05 | 0.00 | 0.00 | 0.00 | 0.00 | 0.05 | 0.09 | 0.00 | 0.00 | 0.00 | *Proteobacteria* | *Betaproteobacteria* | *Rhodocyclales* | *Rhodocyclaceae* | Common taxa |
| OTU 724 | 0.00 | 0.00 | 0.00 | 0.05 | 0.00 | 0.00 | 0.00 | 0.00 | 0.09 | 0.12 | 0.00 | 0.10 | *Proteobacteria* | *Deltaproteobacteria* | *Desulfobacterales* | *Desulfobulbaceae* | Common taxa |
| OTU 725 | 0.06 | 0.06 | 0.00 | 0.09 | 0.00 | 0.00 | 0.00 | 0.00 | 0.09 | 0.00 | 0.00 | 0.10 | *Proteobacteria* | *Gammaproteobacteria* | *Pseudomonadales* | *Moraxellaceae* | Common taxa |
| OTU 727 | 0.12 | 0.00 | 0.00 | 0.00 | 0.00 | 0.00 | 0.00 | 0.00 | 0.00 | 0.04 | 0.11 | 0.15 | *Proteobacteria* | *Betaproteobacteria* | *Rhodocyclales* | *Rhodocyclaceae* | Common taxa |
| OTU 728 | 0.25 | 0.00 | 0.00 | 0.00 | 0.00 | 0.10 | 0.00 | 0.00 | 0.00 | 0.00 | 0.05 | 0.05 | *Proteobacteria* | *Alphaproteobacteria* | *Sphingomonadales* | *Sphingomonadaceae* | Common taxa |
| OTU 729 | 0.00 | 0.11 | 0.00 | 0.00 | 0.00 | 0.15 | 0.00 | 0.05 | 0.09 | 0.00 | 0.00 | 0.00 | *Proteobacteria* | *Alphaproteobacteria* | *Caulobacterales* | *Hyphomonadaceae* | Common taxa |
| OTU 734 | 0.06 | 0.00 | 0.00 | 0.00 | 0.00 | 0.15 | 0.00 | 0.05 | 0.09 | 0.04 | 0.00 | 0.00 | *Proteobacteria* | *Epsilonproteobacteria* | *Campylobacterales* | *Campylobacteraceae* | Common taxa |
| OTU 737 | 0.00 | 0.00 | 0.00 | 0.18 | 0.00 | 0.05 | 0.00 | 0.09 | 0.00 | 0.04 | 0.00 | 0.00 | *Planctomycetes* | *Phycisphaerae* | *Phycisphaerales* | *Phycisphaeraceae* | Common taxa |
| OTU 749 | 0.00 | 0.00 | 0.14 | 0.00 | 0.05 | 0.10 | 0.04 | 0.00 | 0.00 | 0.00 | 0.00 | 0.00 | *Proteobacteria* | *Gammaproteobacteria* | *Alteromonadales* | *Alteromonadaceae* | Common taxa |
| OTU 753 | 0.00 | 0.00 | 0.00 | 0.00 | 0.00 | 0.00 | 0.09 | 0.14 | 0.05 | 0.04 | 0.00 | 0.00 | *Nitrospirae* | *Nitrospira* | *Nitrospirales* | *Nitrospiraceae* | Common taxa |
| OTU 757 | 0.00 | 0.06 | 0.14 | 0.00 | 0.05 | 0.00 | 0.00 | 0.05 | 0.00 | 0.04 | 0.00 | 0.00 | *Planctomycetes* | *Planctomycetia* | *Planctomycetales* | *Planctomycetaceae* | Common taxa |
| OTU 758 | 0.00 | 0.06 | 0.00 | 0.00 | 0.00 | 0.00 | 0.00 | 0.09 | 0.09 | 0.04 | 0.00 | 0.05 | *Proteobacteria* | *Gammaproteobacteria* | *Thiotrichales* | *Thiotrichaceae* | Common taxa |
| OTU 760 | 0.00 | 0.00 | 0.05 | 0.05 | 0.00 | 0.00 | 0.13 | 0.00 | 0.00 | 0.00 | 0.00 | 0.10 | *Proteobacteria* | *Alphaproteobacteria* | *Sphingomonadales* | *Erythrobacteraceae* | Common taxa |
| OTU 764 | 0.00 | 0.06 | 0.19 | 0.00 | 0.00 | 0.00 | 0.00 | 0.05 | 0.05 | 0.00 | 0.00 | 0.00 | *Proteobacteria* | *Betaproteobacteria* | *Rhodocyclales* | *Rhodocyclaceae* | Common taxa |
| OTU 765 | 0.00 | 0.06 | 0.00 | 0.00 | 0.00 | 0.00 | 0.00 | 0.05 | 0.09 | 0.00 | 0.16 | 0.00 | *Proteobacteria* | *Betaproteobacteria* | *Rhodocyclales* | *Rhodocyclaceae* | Common taxa |
| OTU 768 | 0.00 | 0.00 | 0.00 | 0.00 | 0.05 | 0.00 | 0.00 | 0.05 | 0.14 | 0.00 | 0.00 | 0.10 | *Chloroflexi* | *Anaerolineae* | *Anaerolineales* | *Anaerolineaceae* | Common taxa |
| OTU 771 | 0.12 | 0.11 | 0.05 | 0.00 | 0.00 | 0.05 | 0.00 | 0.00 | 0.00 | 0.00 | 0.05 | 0.00 | *Actinobacteria* | *Actinobacteria* | *Acidimicrobidae* | *Acidimicrobiales* | Common taxa |
| OTU 772 | 0.19 | 0.00 | 0.00 | 0.00 | 0.00 | 0.00 | 0.00 | 0.00 | 0.05 | 0.08 | 0.05 | 0.00 | *Proteobacteria* | *Deltaproteobacteria* | *Myxococcales* | *Sorangiineae* | Common taxa |
| OTU 773 | 0.00 | 0.00 | 0.00 | 0.05 | 0.00 | 0.15 | 0.00 | 0.00 | 0.00 | 0.08 | 0.05 | 0.00 | *Fusobacteria* | *Fusobacteriia* | *Fusobacteriales* | *Fusobacteriaceae* | Common taxa |
| OTU 774 | 0.00 | 0.06 | 0.00 | 0.00 | 0.05 | 0.00 | 0.00 | 0.00 | 0.00 | 0.00 | 0.22 | 0.05 | *Proteobacteria* | *Betaproteobacteria* | *Burkholderiales* | *Burkholderiales_incertae_sedis* | Common taxa |
| OTU 779 | 0.06 | 0.00 | 0.00 | 0.00 | 0.00 | 0.00 | 0.00 | 0.09 | 0.05 | 0.04 | 0.00 | 0.10 | *Planctomycetes* | *Planctomycetia* | *Planctomycetales* | *Planctomycetaceae* | Common taxa |
| OTU 780 | 0.06 | 0.00 | 0.00 | 0.00 | 0.00 | 0.00 | 0.00 | 0.00 | 0.05 | 0.08 | 0.00 | 0.15 | *Proteobacteria* | *Alphaproteobacteria* | *Caulobacterales* | *Caulobacteraceae* | Common taxa |
| OTU 784 | 0.00 | 0.06 | 0.00 | 0.00 | 0.00 | 0.10 | 0.00 | 0.09 | 0.05 | 0.00 | 0.00 | 0.05 | *Proteobacteria* | *Gammaproteobacteria* | *Aeromonadales* | *Aeromonadaceae* | Common taxa |
| OTU 786 | 0.06 | 0.06 | 0.00 | 0.00 | 0.05 | 0.00 | 0.00 | 0.00 | 0.00 | 0.00 | 0.00 | 0.20 | *Proteobacteria* | *Betaproteobacteria* | *Neisseriales* | *Neisseriaceae* | Common taxa |
| OTU 787 | 0.00 | 0.00 | 0.00 | 0.00 | 0.05 | 0.00 | 0.04 | 0.09 | 0.09 | 0.04 | 0.00 | 0.00 | *Proteobacteria* | *Betaproteobacteria* | *Rhodocyclales* | *Rhodocyclaceae* | Common taxa |
| OTU 788 | 0.00 | 0.00 | 0.00 | 0.05 | 0.11 | 0.00 | 0.00 | 0.00 | 0.00 | 0.00 | 0.11 | 0.10 | *Proteobacteria* | *Betaproteobacteria* | *Rhodocyclales* | *Rhodocyclaceae* | Common taxa |
| OTU 789 | 0.00 | 0.11 | 0.05 | 0.09 | 0.00 | 0.00 | 0.00 | 0.05 | 0.00 | 0.00 | 0.00 | 0.05 | *Proteobacteria* | *Betaproteobacteria* | *Rhodocyclales* | *Rhodocyclaceae* | Common taxa |
| OTU 791 | 0.12 | 0.00 | 0.00 | 0.00 | 0.00 | 0.10 | 0.04 | 0.00 | 0.00 | 0.08 | 0.00 | 0.00 | *Proteobacteria* | *Alphaproteobacteria* | *Rhizobiales* | *Hyphomicrobiaceae* | Common taxa |
| OTU 792 | 0.06 | 0.00 | 0.00 | 0.00 | 0.05 | 0.15 | 0.00 | 0.05 | 0.00 | 0.04 | 0.00 | 0.00 | *Proteobacteria* | *Alphaproteobacteria* | *Rhizobiales* | *Beijerinckiaceae* | Common taxa |

| | | | | | | | | | | | | | | | | | |
|---|---|---|---|---|---|---|---|---|---|---|---|---|---|---|---|---|---|
| OTU 794 | 0.00 | 0.00 | 0.05 | 0.09 | 0.05 | 0.10 | 0.00 | 0.05 | 0.00 | 0.00 | 0.00 | 0.00 | *Bacteroidetes* | *Sphingobacteriia* | *Sphingobacteriales* | *Saprospiraceae* | Common taxa |
| OTU 795 | 0.06 | 0.00 | 0.10 | 0.00 | 0.00 | 0.10 | 0.00 | 0.00 | 0.00 | 0.00 | 0.00 | 0.10 | *Proteobacteria* | *Gammaproteobacteria* | *Thiotrichales* | *Thiotrichaceae* | Common taxa |
| OTU 802 | 0.00 | 0.00 | 0.14 | 0.05 | 0.00 | 0.05 | 0.00 | 0.00 | 0.05 | 0.04 | 0.00 | 0.00 | *Bacteroidetes* | *Cytophagia* | *Cytophagales* | *Cytophagaceae* | Common taxa |
| OTU 803 | 0.00 | 0.00 | 0.00 | 0.05 | 0.00 | 0.00 | 0.00 | 0.14 | 0.09 | 0.04 | 0.00 | 0.00 | *Proteobacteria* | *Betaproteobacteria* | *Burkholderiales* | *Comamonadaceae* | Common taxa |
| OTU 809 | 0.06 | 0.00 | 0.00 | 0.00 | 0.05 | 0.00 | 0.00 | 0.00 | 0.00 | 0.04 | 0.00 | 0.20 | *Spirochaetes* | *Spirochaetia* | *Spirochaetales* | *Spirochaetaceae* | Common taxa |
| OTU 818 | 0.00 | 0.00 | 0.00 | 0.00 | 0.00 | 0.00 | 0.00 | 0.00 | 0.09 | 0.04 | 0.11 | 0.10 | *Bacteroidetes* | *Sphingobacteriia* | *Sphingobacteriales* | *Cyclobacteriaceae* | Common taxa |
| OTU 825 | 0.00 | 0.00 | 0.00 | 0.09 | 0.16 | 0.05 | 0.04 | 0.00 | 0.00 | 0.00 | 0.00 | 0.00 | *Bacteroidetes* | *Flavobacteriia* | *Flavobacteriales* | *Flavobacteriaceae* | Common taxa |
| OTU 826 | 0.06 | 0.00 | 0.05 | 0.00 | 0.00 | 0.10 | 0.00 | 0.00 | 0.00 | 0.12 | 0.00 | 0.00 | *Proteobacteria* | *Gammaproteobacteria* | *Methylococcales* | *Methylococcaceae* | Common taxa |
| OTU 828 | 0.00 | 0.00 | 0.00 | 0.00 | 0.00 | 0.00 | 0.00 | 0.05 | 0.09 | 0.04 | 0.00 | 0.10 | *Firmicutes* | *Clostridia* | *Clostridiales* | *Clostridiales_Incertae Sedis XII* | Common taxa |
| OTU 833 | 0.12 | 0.00 | 0.00 | 0.00 | 0.05 | 0.00 | 0.04 | 0.00 | 0.09 | 0.00 | 0.00 | 0.00 | *Bacteroidetes* | *Sphingobacteriia* | *Sphingobacteriales* | *Cyclobacteriaceae* | Common taxa |
| OTU 834 | 0.00 | 0.11 | 0.05 | 0.00 | 0.00 | 0.00 | 0.09 | 0.00 | 0.00 | 0.00 | 0.00 | 0.05 | *Planctomycetes* | *Planctomycetia* | *Planctomycetales* | *Planctomycetaceae* | Common taxa |
| OTU 842 | 0.00 | 0.00 | 0.00 | 0.05 | 0.00 | 0.05 | 0.13 | 0.05 | 0.00 | 0.00 | 0.00 | 0.00 | *Proteobacteria* | *Gammaproteobacteria* | *Thiotrichales* | *Thiotrichaceae* | Common taxa |
| OTU 849 | 0.00 | 0.00 | 0.00 | 0.14 | 0.05 | 0.05 | 0.04 | 0.00 | 0.00 | 0.00 | 0.00 | 0.00 | *Bacteroidetes* | *Bacteroidia* | *Bacteroidales* | *Marinilabiliaceae* | Common taxa |
| OTU 854 | 0.00 | 0.00 | 0.00 | 0.00 | 0.00 | 0.00 | 0.00 | 0.09 | 0.09 | 0.04 | 0.00 | 0.05 | *Acidobacteria* | *Acidobacteria_Gp4* | *Gp4* | | Common taxa |
| OTU 856 | 0.00 | 0.11 | 0.00 | 0.00 | 0.00 | 0.00 | 0.04 | 0.00 | 0.05 | 0.00 | 0.11 | 0.00 | *Proteobacteria* | *Gammaproteobacteria* | *Pseudomonadales* | *Moraxellaceae* | Common taxa |
| OTU 860 | 0.12 | 0.00 | 0.00 | 0.00 | 0.00 | 0.00 | 0.00 | 0.00 | 0.05 | 0.08 | 0.05 | 0.00 | *Proteobacteria* | *Gammaproteobacteria* | *Xanthomonadales* | *Xanthomonadaceae* | Common taxa |
| OTU 862 | 0.00 | 0.00 | 0.00 | 0.09 | 0.11 | 0.00 | 0.00 | 0.00 | 0.00 | 0.04 | 0.00 | 0.05 | *Bacteroidetes* | *Cytophagia* | *Cytophagales* | *Cytophagaceae* | Common taxa |
| OTU 867 | 0.00 | 0.00 | 0.00 | 0.05 | 0.05 | 0.00 | 0.00 | 0.00 | 0.00 | 0.00 | 0.11 | 0.10 | *Bacteroidetes* | *Flavobacteriia* | *Flavobacteriales* | *Flavobacteriaceae* | Common taxa |
| OTU 868 | 0.00 | 0.11 | 0.05 | 0.09 | 0.00 | 0.00 | 0.00 | 0.00 | 0.05 | 0.00 | 0.00 | 0.00 | *Bacteroidetes* | *Sphingobacteriia* | *Sphingobacteriales* | *Saprospiraceae* | Common taxa |
| OTU 875 | 0.00 | 0.00 | 0.05 | 0.05 | 0.00 | 0.00 | 0.04 | 0.14 | 0.00 | 0.00 | 0.00 | 0.00 | *Proteobacteria* | *Gammaproteobacteria* | *Xanthomonadales* | *Sinobacteraceae* | Common taxa |
| OTU 878 | 0.06 | 0.00 | 0.00 | 0.00 | 0.00 | 0.00 | 0.04 | 0.00 | 0.00 | 0.04 | 0.16 | 0.00 | *Proteobacteria* | *Gammaproteobacteria* | *Xanthomonadales* | *Xanthomonadaceae* | Common taxa |
| OTU 884 | 0.06 | 0.11 | 0.05 | 0.00 | 0.00 | 0.00 | 0.00 | 0.00 | 0.00 | 0.00 | 0.11 | 0.00 | *Proteobacteria* | *Betaproteobacteria* | *Rhodocyclales* | *Rhodocyclaceae* | Common taxa |
| OTU 885 | 0.00 | 0.00 | 0.00 | 0.00 | 0.00 | 0.05 | 0.00 | 0.05 | 0.14 | 0.04 | 0.00 | 0.00 | *Proteobacteria* | *Deltaproteobacteria* | *Myxococcales* | *Sorangiineae* | Common taxa |
| OTU 897 | 0.00 | 0.00 | 0.00 | 0.00 | 0.00 | 0.10 | 0.00 | 0.09 | 0.05 | 0.04 | 0.00 | 0.00 | *Lentisphaerae* | *Lentisphaeria* | *Lentisphaerales* | *Lentisphaeraceae* | Common taxa |
| OTU 915 | 0.12 | 0.00 | 0.00 | 0.00 | 0.00 | 0.10 | 0.00 | 0.00 | 0.05 | 0.00 | 0.05 | 0.00 | *Proteobacteria* | *Betaproteobacteria* | *Burkholderiales* | *Comamonadaceae* | Common taxa |
| OTU 916 | 0.00 | 0.00 | 0.14 | 0.00 | 0.05 | 0.05 | 0.00 | 0.00 | 0.00 | 0.04 | 0.00 | 0.00 | *Proteobacteria* | *Alphaproteobacteria* | *Sneathiellales* | *Sneathiellaceae* | Common taxa |
| OTU 917 | 0.00 | 0.00 | 0.00 | 0.00 | 0.00 | 0.05 | 0.00 | 0.00 | 0.05 | 0.04 | 0.00 | 0.15 | *Planctomycetes* | *Planctomycetia* | *Candidatus Brocadiales* | *Candidatus Brocadiaceae* | Common taxa |
| OTU 918 | 0.00 | 0.00 | 0.00 | 0.00 | 0.00 | 0.05 | 0.00 | 0.00 | 0.00 | 0.04 | 0.11 | 0.10 | *Proteobacteria* | *Alphaproteobacteria* | *Eilatimonas* | | Common taxa |
| OTU 922 | 0.00 | 0.00 | 0.05 | 0.00 | 0.00 | 0.10 | 0.09 | 0.05 | 0.00 | 0.00 | 0.00 | 0.00 | *Planctomycetes* | *Planctomycetia* | *Planctomycetales* | *Planctomycetaceae* | Common taxa |
| OTU 929 | 0.00 | 0.00 | 0.00 | 0.00 | 0.05 | 0.05 | 0.00 | 0.00 | 0.00 | 0.08 | 0.00 | 0.10 | *Bacteroidetes* | *Bacteroidia* | *Bacteroidales* | *Porphyromonadaceae* | Common taxa |
| OTU 936 | 0.00 | 0.06 | 0.05 | 0.00 | 0.00 | 0.00 | 0.00 | 0.00 | 0.05 | 0.00 | 0.00 | 0.15 | *Actinobacteria* | *Actinobacteria* | *Acidimicrobidae* | *Acidimicrobiales* | Common taxa |
| OTU 944 | 0.00 | 0.00 | 0.00 | 0.00 | 0.05 | 0.00 | 0.04 | 0.09 | 0.00 | 0.00 | 0.00 | 0.05 | *Bacteroidetes* | *Flavobacteriia* | *Flavobacteriales* | *Flavobacteriaceae* | Common taxa |
| OTU 945 | 0.00 | 0.00 | 0.00 | 0.05 | 0.00 | 0.00 | 0.00 | 0.00 | 0.05 | 0.08 | 0.05 | 0.00 | *Bacteroidetes* | *Cytophagia* | *Cytophagales* | *Cytophagaceae* | Common taxa |
| OTU 947 | 0.00 | 0.00 | 0.00 | 0.00 | 0.05 | 0.00 | 0.00 | 0.00 | 0.05 | 0.00 | 0.11 | 0.05 | *Actinobacteria* | *Actinobacteria* | *Acidimicrobidae* | *Acidimicrobiales* | Common taxa |
| OTU 948 | 0.00 | 0.00 | 0.00 | 0.05 | 0.11 | 0.00 | 0.04 | 0.05 | 0.00 | 0.00 | 0.00 | 0.00 | *Firmicutes* | *Clostridia* | *Clostridiales* | *Ruminococcaceae* | Common taxa |
| OTU 954 | 0.00 | 0.00 | 0.00 | 0.00 | 0.00 | 0.00 | 0.00 | 0.05 | 0.00 | 0.04 | 0.05 | 0.10 | *Firmicutes* | *Bacilli* | *Bacillales* | *Bacillaceae 1* | Common taxa |
| OTU 975 | 0.00 | 0.00 | 0.00 | 0.05 | 0.00 | 0.00 | 0.04 | 0.00 | 0.09 | 0.00 | 0.05 | 0.00 | *Proteobacteria* | *Gammaproteobacteria* | *Xanthomonadales* | *Xanthomonadaceae* | Common taxa |
| OTU 979 | 0.00 | 0.00 | 0.00 | 0.05 | 0.00 | 0.00 | 0.00 | 0.09 | 0.05 | 0.04 | 0.00 | 0.00 | *Bacteroidetes* | *Sphingobacteriia* | *Sphingobacteriales* | *Saprospiraceae* | Common taxa |
| OTU 986 | 0.00 | 0.06 | 0.10 | 0.00 | 0.00 | 0.05 | 0.00 | 0.00 | 0.00 | 0.04 | 0.00 | 0.00 | *Actinobacteria* | *Actinobacteria* | *Actinobacteridae* | *Actinomycetales* | Common taxa |
| OTU 988 | 0.06 | 0.00 | 0.00 | 0.00 | 0.00 | 0.00 | 0.00 | 0.00 | 0.05 | 0.04 | 0.11 | 0.00 | *Bacteroidetes* | *Flavobacteriia* | *Flavobacteriales* | *Flavobacteriaceae* | Common taxa |
| OTU 1001 | 0.06 | 0.00 | 0.00 | 0.00 | 0.05 | 0.00 | 0.00 | 0.00 | 0.00 | 0.00 | 0.11 | 0.05 | *Proteobacteria* | *Betaproteobacteria* | *Rhodocyclales* | *Rhodocyclaceae* | Common taxa |
| OTU 1002 | 0.00 | 0.00 | 0.00 | 0.09 | 0.00 | 0.05 | 0.00 | 0.00 | 0.05 | 0.00 | 0.05 | 0.00 | *Proteobacteria* | *Betaproteobacteria* | *Burkholderiales* | *Burkholderiales_incertae_sedis* | Common taxa |
| OTU 1006 | 0.00 | 0.00 | 0.10 | 0.00 | 0.00 | 0.05 | 0.00 | 0.05 | 0.00 | 0.04 | 0.00 | 0.00 | *Proteobacteria* | *Epsilonproteobacteria* | *Campylobacterales* | *Campylobacteraceae* | Common taxa |
| OTU 1022 | 0.12 | 0.00 | 0.00 | 0.05 | 0.05 | 0.00 | 0.00 | 0.00 | 0.00 | 0.04 | 0.00 | 0.00 | *Proteobacteria* | *Deltaproteobacteria* | *Myxococcales* | *Cystobacterineae* | Common taxa |
| OTU 1025 | 0.00 | 0.00 | 0.10 | 0.05 | 0.00 | 0.05 | 0.00 | 0.05 | 0.00 | 0.00 | 0.00 | 0.00 | *Nitrospirae* | *Nitrospira* | *Nitrospirales* | *Nitrospiraceae* | Common taxa |
| OTU 1026 | 0.00 | 0.00 | 0.10 | 0.00 | 0.00 | 0.00 | 0.00 | 0.00 | 0.00 | 0.04 | 0.05 | 0.05 | *Proteobacteria* | *Alphaproteobacteria* | *Rhodobacterales* | *Rhodobacteraceae* | Common taxa |
| OTU 1036 | 0.12 | 0.00 | 0.00 | 0.00 | 0.00 | 0.05 | 0.00 | 0.00 | 0.05 | 0.04 | 0.00 | 0.00 | *Bacteroidetes* | *Sphingobacteriia* | *Sphingobacteriales* | *Cyclobacteriaceae* | Common taxa |
| OTU 1041 | 0.12 | 0.06 | 0.05 | 0.00 | 0.00 | 0.00 | 0.00 | 0.00 | 0.00 | 0.00 | 0.05 | 0.00 | *Proteobacteria* | *Betaproteobacteria* | *Rhodocyclales* | *Rhodocyclaceae* | Common taxa |
| OTU 1042 | 0.00 | 0.00 | 0.00 | 0.00 | 0.00 | 0.05 | 0.00 | 0.00 | 0.00 | 0.04 | 0.11 | 0.05 | *Proteobacteria* | *Alphaproteobacteria* | *Caulobacterales* | *Caulobacteraceae* | Common taxa |
| OTU 1048 | 0.00 | 0.06 | 0.05 | 0.00 | 0.00 | 0.00 | 0.00 | 0.09 | 0.05 | 0.00 | 0.00 | 0.00 | *Chloroflexi* | *Anaerolineae* | *Anaerolineales* | *Anaerolineaceae* | Common taxa |
| OTU 1052 | 0.06 | 0.00 | 0.05 | 0.00 | 0.00 | 0.00 | 0.00 | 0.00 | 0.05 | 0.00 | 0.00 | 0.10 | *Proteobacteria* | *Alphaproteobacteria* | *Rhizobiales* | *Hyphomicrobiaceae* | Common taxa |
| OTU 1056 | 0.06 | 0.11 | 0.00 | 0.05 | 0.00 | 0.00 | 0.00 | 0.00 | 0.05 | 0.00 | 0.00 | 0.00 | *Chloroflexi* | *Anaerolineae* | *Anaerolineales* | *Anaerolineaceae* | Common taxa |
| OTU 1065 | 0.06 | 0.00 | 0.05 | 0.05 | 0.11 | 0.00 | 0.00 | 0.00 | 0.00 | 0.00 | 0.00 | 0.00 | *Proteobacteria* | *Alphaproteobacteria* | *Rhizobiales* | *Rhizobiaceae* | Common taxa |
| OTU 1071 | 0.00 | 0.00 | 0.10 | 0.05 | 0.05 | 0.00 | 0.00 | 0.00 | 0.00 | 0.00 | 0.05 | 0.00 | *Proteobacteria* | *Epsilonproteobacteria* | *Campylobacterales* | *Campylobacteraceae* | Common taxa |
| OTU 1074 | 0.00 | 0.00 | 0.00 | 0.00 | 0.00 | 0.05 | 0.00 | 0.05 | 0.05 | 0.08 | 0.00 | 0.00 | *Proteobacteria* | *Alphaproteobacteria* | *Sneathiellales* | *Sneathiellaceae* | Common taxa |
| OTU 1082 | 0.00 | 0.00 | 0.00 | 0.00 | 0.00 | 0.10 | 0.04 | 0.00 | 0.05 | 0.00 | 0.05 | 0.00 | *Chloroflexi* | *Anaerolineae* | *Anaerolineales* | *Anaerolineaceae* | Common taxa |
| OTU 1089 | 0.00 | 0.00 | 0.05 | 0.00 | 0.05 | 0.00 | 0.00 | 0.00 | 0.05 | 0.08 | 0.00 | 0.00 | *Proteobacteria* | *Alphaproteobacteria* | *Rhodospirillales* | *Acetobacteraceae* | Common taxa |
| OTU 1090 | 0.00 | 0.00 | 0.10 | 0.05 | 0.00 | 0.00 | 0.00 | 0.00 | 0.00 | 0.00 | 0.05 | 0.05 | *Bacteroidetes* | *Sphingobacteriia* | *Sphingobacteriales* | *Sphingobacteriaceae* | Common taxa |
| OTU 1093 | 0.00 | 0.06 | 0.00 | 0.00 | 0.00 | 0.05 | 0.00 | 0.00 | 0.05 | 0.00 | 0.00 | 0.05 | *Proteobacteria* | *Betaproteobacteria* | *Rhodocyclales* | *Rhodocyclaceae* | Common taxa |
| OTU 1097 | 0.00 | 0.00 | 0.05 | 0.00 | 0.00 | 0.05 | 0.04 | 0.00 | 0.00 | 0.00 | 0.05 | 0.00 | *Proteobacteria* | *Betaproteobacteria* | *Rhodocyclales* | *Rhodocyclaceae* | Common taxa |
| OTU 1105 | 0.00 | 0.00 | 0.00 | 0.05 | 0.05 | 0.00 | 0.00 | 0.00 | 0.05 | 0.00 | 0.00 | 0.05 | *Bacteroidetes* | *Sphingobacteriia* | *Sphingobacteriales* | *Saprospiraceae* | Common taxa |
| OTU 1111 | 0.06 | 0.06 | 0.00 | 0.00 | 0.00 | 0.00 | 0.00 | 0.05 | 0.00 | 0.00 | 0.05 | 0.00 | *Proteobacteria* | *Betaproteobacteria* | *Rhodocyclales* | *Rhodocyclaceae* | Common taxa |
| OTU 1123 | 0.00 | 0.06 | 0.00 | 0.00 | 0.00 | 0.00 | 0.04 | 0.00 | 0.00 | 0.00 | 0.05 | 0.05 | *Proteobacteria* | *Betaproteobacteria* | *Rhodocyclales* | *Rhodocyclaceae* | Common taxa |
| OTU 1126 | 0.00 | 0.06 | 0.00 | 0.00 | 0.00 | 0.00 | 0.00 | 0.05 | 0.05 | 0.00 | 0.00 | 0.05 | *Proteobacteria* | *Deltaproteobacteria* | *Myxococcales* | *Sorangiineae* | Common taxa |
| OTU 1137 | 0.00 | 0.06 | 0.00 | 0.05 | 0.00 | 0.00 | 0.00 | 0.05 | 0.05 | 0.00 | 0.00 | 0.00 | *Chloroflexi* | *Caldilineae* | *Caldilineales* | *Caldilineaceae* | Common taxa |
| OTU 1149 | 0.06 | 0.00 | 0.00 | 0.00 | 0.00 | 0.05 | 0.00 | 0.00 | 0.05 | 0.04 | 0.00 | 0.00 | *Proteobacteria* | *Alphaproteobacteria* | *Rhizobiales* | *Methylobacteriaceae* | Common taxa |
| OTU 1162 | 0.00 | 0.06 | 0.05 | 0.05 | 0.00 | 0.00 | 0.00 | 0.00 | 0.05 | 0.00 | 0.00 | 0.00 | *Bacteroidetes* | *Sphingobacteriia* | *Sphingobacteriales* | *Chitinophagaceae* | Common taxa |
| OTU 1168 | 0.00 | 0.06 | 0.05 | 0.00 | 0.05 | 0.00 | 0.04 | 0.00 | 0.00 | 0.00 | 0.00 | 0.00 | *Proteobacteria* | *Deltaproteobacteria* | *Desulfarculales* | *Desulfarculaceae* | Common taxa |
| OTU 1173 | 0.00 | 0.00 | 0.05 | 0.05 | 0.00 | 0.00 | 0.00 | 0.05 | 0.05 | 0.00 | 0.00 | 0.00 | *Proteobacteria* | *Betaproteobacteria* | *Rhodocyclales* | *Rhodocyclaceae* | Common taxa |
| OTU 1180 | 0.00 | 0.00 | 0.00 | 0.00 | 0.05 | 0.00 | 0.00 | 0.05 | 0.00 | 0.04 | 0.00 | 0.05 | *Proteobacteria* | *Betaproteobacteria* | *Rhodocyclales* | *Rhodocyclaceae* | Common taxa |
| OTU 1201 | 0.00 | 0.06 | 0.00 | 0.00 | 0.00 | 0.05 | 0.00 | 0.05 | 0.05 | 0.00 | 0.00 | 0.00 | *Proteobacteria* | *Alphaproteobacteria* | *Rhizobiales* | *Beijerinckiaceae* | Common taxa |
| OTU 1202 | 0.00 | 0.06 | 0.00 | 0.00 | 0.00 | 0.00 | 0.04 | 0.05 | 0.00 | 0.00 | 0.05 | 0.00 | *Proteobacteria* | *Betaproteobacteria* | *Rhodocyclales* | *Rhodocyclaceae* | Common taxa |
| OTU 1204 | 0.06 | 0.00 | 0.00 | 0.00 | 0.00 | 0.05 | 0.00 | 0.00 | 0.00 | 0.00 | 0.05 | 0.05 | *Proteobacteria* | *Deltaproteobacteria* | *Myxococcales* | *Sorangiineae* | Common taxa |
| OTU 1212 | 0.06 | 0.06 | 0.00 | 0.00 | 0.00 | 0.00 | 0.00 | 0.00 | 0.05 | 0.00 | 0.05 | 0.00 | *Proteobacteria* | *Betaproteobacteria* | *Rhodocyclales* | *Rhodocyclaceae* | Common taxa |
| OTU 1222 | 0.06 | 0.00 | 0.05 | 0.05 | 0.00 | 0.00 | 0.00 | 0.00 | 0.05 | 0.00 | 0.00 | 0.00 | *Proteobacteria* | *Betaproteobacteria* | *Burkholderiales* | *Comamonadaceae* | Common taxa |
| OTU 1245 | 0.06 | 0.00 | 0.00 | 0.00 | 0.00 | 0.05 | 0.00 | 0.00 | 0.05 | 0.04 | 0.00 | 0.00 | *Proteobacteria* | *Gammaproteobacteria* | *Methylococcales* | *Methylococcaceae* | Common taxa |
| OTU 1248 | 0.00 | 0.00 | 0.05 | 0.05 | 0.05 | 0.00 | 0.04 | 0.00 | 0.00 | 0.00 | 0.00 | 0.00 | *Bacteroidetes* | *Bacteroidia* | *Bacteroidales* | *Marinilabiliaceae* | Common taxa |
| OTU 1262 | 0.00 | 0.00 | 0.00 | 0.00 | 0.00 | 0.05 | 0.04 | 0.05 | 0.05 | 0.00 | 0.00 | 0.00 | *Proteobacteria* | *Alphaproteobacteria* | *Rhodobacterales* | *Rhodobacteraceae* | Common taxa |
| OTU 1266 | 0.06 | 0.00 | 0.00 | 0.00 | 0.00 | 0.00 | 0.00 | 0.00 | 0.00 | 0.04 | 0.05 | 0.05 | *Proteobacteria* | *Betaproteobacteria* | *Rhodocyclales* | *Rhodocyclaceae* | Common taxa |
| OTU 1268 | 0.00 | 0.00 | 0.05 | 0.00 | 0.05 | 0.05 | 0.00 | 0.00 | 0.00 | 0.04 | 0.00 | 0.00 | *Proteobacteria* | *Gammaproteobacteria* | *Enterobacteriales* | *Enterobacteriaceae* | Common taxa |
| OTU 1271 | 0.00 | 0.00 | 0.00 | 0.00 | 0.00 | 0.00 | 0.04 | 0.05 | 0.00 | 0.04 | 0.05 | 0.00 | *Proteobacteria* | *Betaproteobacteria* | *Rhodocyclales* | *Rhodocyclaceae* | Common taxa |
| OTU 1272 | 0.00 | 0.00 | 0.00 | 0.05 | 0.00 | 0.00 | 0.04 | 0.00 | 0.00 | 0.00 | 0.05 | 0.05 | *Proteobacteria* | *Deltaproteobacteria* | *Myxococcales* | *Nannocystineae* | Common taxa |
| OTU 1284 | 0.00 | 0.00 | 0.05 | 0.05 | 0.00 | 0.00 | 0.04 | 0.00 | 0.00 | 0.00 | 0.00 | 0.05 | *Proteobacteria* | *Deltaproteobacteria* | *Myxococcales* | *Nannocystineae* | Common taxa |
| OTU 1289 | 0.00 | 0.00 | 0.00 | 0.00 | 0.00 | 0.05 | 0.00 | 0.05 | 0.00 | 0.00 | 0.05 | 0.05 | *Proteobacteria* | *Betaproteobacteria* | *Rhodocyclales* | *Rhodocyclaceae* | Common taxa |
| OTU 1320 | 0.00 | 0.00 | 0.00 | 0.00 | 0.00 | 0.05 | 0.04 | 0.05 | 0.05 | 0.00 | 0.00 | 0.00 | *Actinobacteria* | *Actinobacteria* | *Actinobacteridae* | *Actinomycetales* | Common taxa |
| OTU 1324 | 0.00 | 0.00 | 0.00 | 0.00 | 0.05 | 0.05 | 0.00 | 0.05 | 0.00 | 0.04 | 0.00 | 0.00 | *Proteobacteria* | *Gammaproteobacteria* | *Gammaproteobacteria_incertae_sedis* | *Thioprofundum* | Common taxa |
| OTU 49 | 0.00 | 0.00 | 0.00 | 0.00 | 0.09 | 0.97 | 0.08 | 0.00 | 0.00 | 0.00 | 0.00 | 0.00 | *Proteobacteria* | *Betaproteobacteria* | *Rhodocyclales* | *Rhodocyclaceae* | Transient-rare taxa |
| OTU 54 | 0.00 | 0.00 | 0.00 | 0.01 | 0.97 | 0.09 | 0.00 | 0.00 | 0.00 | 0.00 | 0.00 | 0.00 | *Proteobacteria* | *Deltaproteobacteria* | *Myxococcales* | *Sorangiineae* | Transient-rare taxa |
| OTU 55 | 0.00 | 0.00 | 0.00 | 0.00 | 1.05 | 0.00 | 0.00 | 0.00 | 0.00 | 0.00 | 0.00 | 0.00 | *Bacteroidetes* | *Sphingobacteriia* | *Sphingobacteriales* | *Saprospiraceae* | Transient-rare taxa |
| OTU 56 | 0.00 | 0.00 | 0.00 | 0.98 | 0.00 | 0.03 | 0.03 | 0.00 | 0.00 | 0.00 | 0.00 | 0.00 | *Proteobacteria* | *Gammaproteobacteria* | *Gammaproteobacteria_incertae_sedis* | *Methylohalomonas* | Transient-rare taxa |
| OTU 61 | 0.00 | 0.00 | 0.00 | 0.00 | 0.86 | 0.08 | 0.00 | 0.00 | 0.00 | 0.00 | 0.00 | 0.00 | *Proteobacteria* | *Gammaproteobacteria* | *Alteromonadales* | *Alteromonadaceae* | Transient-rare taxa |
| OTU 71 | 0.33 | 0.43 | 0.00 | 0.00 | 0.00 | 0.00 | 0.00 | 0.00 | 0.00 | 0.00 | 0.00 | 0.09 | *Proteobacteria* | *Betaproteobacteria* | *Rhodocyclales* | *Rhodocyclaceae* | Transient-rare taxa |
| OTU 75 | 0.00 | 0.00 | 0.00 | 0.00 | 0.09 | 0.64 | 0.06 | 0.00 | 0.00 | 0.00 | 0.00 | 0.00 | *Proteobacteria* | *Betaproteobacteria* | *Rhodocyclales* | *Rhodocyclaceae* | Transient-rare taxa |
| OTU 82 | 0.00 | 0.00 | 0.00 | 0.00 | 0.72 | 0.00 | 0.00 | 0.00 | 0.00 | 0.00 | 0.00 | 0.00 | *Proteobacteria* | *Deltaproteobacteria* | *Desulfovibrionales* | *Desulfovibrionaceae* | Transient-rare taxa |
| OTU 100 | 0.18 | 0.02 | 0.00 | 0.00 | 0.00 | 0.00 | 0.00 | 0.00 | 0.00 | 0.00 | 0.00 | 0.41 | *Proteobacteria* | *Betaproteobacteria* | *Rhodocyclales* | *Rhodocyclaceae* | Transient-rare taxa |
| OTU 101 | 0.00 | 0.00 | 0.00 | 0.03 | 0.00 | 0.23 | 0.33 | 0.00 | 0.00 | 0.00 | 0.00 | 0.00 | *Proteobacteria* | *Alphaproteobacteria* | *Sphingomonadales* | *Erythrobacteraceae* | Transient-rare taxa |
| OTU 105 | 0.28 | 0.17 | 0.00 | 0.00 | 0.00 | 0.00 | 0.00 | 0.00 | 0.00 | 0.00 | 0.00 | 0.13 | *Proteobacteria* | *Betaproteobacteria* | *Rhodocyclales* | *Rhodocyclaceae* | Transient-rare taxa |
| OTU 109 | 0.19 | 0.28 | 0.00 | 0.00 | 0.00 | 0.00 | 0.00 | 0.00 | 0.00 | 0.00 | 0.00 | 0.10 | *Proteobacteria* | *Betaproteobacteria* | *Rhodocyclales* | *Rhodocyclaceae* | Transient-rare taxa |
| OTU 128 | 0.00 | 0.00 | 0.00 | 0.00 | 0.00 | 0.00 | 0.00 | 0.39 | 0.00 | 0.09 | 0.00 | 0.00 | *Proteobacteria* | *Deltaproteobacteria* | *Myxococcales* | *Nannocystineae* | Transient-rare taxa |
| OTU 130 | 0.00 | 0.00 | 0.00 | 0.44 | 0.00 | 0.03 | 0.00 | 0.00 | 0.00 | 0.00 | 0.00 | 0.00 | *Bacteroidetes* | *Flavobacteriia* | *Flavobacteriales* | *Cryomorphaceae* | Transient-rare taxa |
| OTU 135 | 0.00 | 0.00 | 0.00 | 0.00 | 0.00 | 0.00 | 0.00 | 0.00 | 0.00 | 0.00 | 0.43 | 0.02 | *Proteobacteria* | *Betaproteobacteria* | *Burkholderiales* | *Comamonadaceae* | Transient-rare taxa |
| OTU 136 | 0.06 | 0.00 | 0.00 | 0.00 | 0.00 | 0.00 | 0.00 | 0.00 | 0.00 | 0.00 | 0.10 | 0.30 | *Proteobacteria* | *Gammaproteobacteria* | *Xanthomonadales* | *Xanthomonadaceae* | Transient-rare taxa |
| OTU 156 | 0.28 | 0.01 | 0.00 | 0.00 | 0.00 | 0.00 | 0.00 | 0.00 | 0.00 | 0.00 | 0.00 | 0.13 | *Proteobacteria* | *Alphaproteobacteria* | *Rhodospirillales* | *Elioraea* | Transient-rare taxa |
| OTU 162 | 0.01 | 0.00 | 0.00 | 0.00 | 0.00 | 0.00 | 0.00 | 0.00 | 0.00 | 0.08 | 0.00 | 0.32 | *Proteobacteria* | *Zetaproteobacteria* | *Mariprofundales* | *Mariprofundaceae* | Transient-rare taxa |
| OTU 174 | 0.00 | 0.01 | 0.27 | 0.09 | 0.00 | 0.00 | 0.00 | 0.00 | 0.00 | 0.00 | 0.00 | 0.00 | *Ignavibacteriae* | *Ignavibacteria* | *Ignavibacteriales* | *Ignavibacteriaceae* | Transient-rare taxa |
| OTU 179 | 0.00 | 0.00 | 0.00 | 0.00 | 0.19 | 0.16 | 0.01 | 0.00 | 0.00 | 0.00 | 0.00 | 0.00 | *Proteobacteria* | *Gammaproteobacteria* | *Thiotrichales* | *Thiotrichaceae* | Transient-rare taxa |
| OTU 183 | 0.00 | 0.00 | 0.00 | 0.32 | 0.00 | 0.00 | 0.00 | 0.00 | 0.00 | 0.03 | 0.00 | 0.00 | *Bacteroidetes* | *Flavobacteriia* | *Flavobacteriales* | *Flavobacteriaceae* | Transient-rare taxa |
| OTU 184 | 0.00 | 0.00 | 0.00 | 0.01 | 0.00 | 0.34 | 0.00 | 0.00 | 0.00 | 0.00 | 0.00 | 0.00 | *Bacteroidetes* | *Sphingobacteriia* | *Sphingobacteriales* | *Saprospiraceae* | Transient-rare taxa |
| OTU 185 | 0.00 | 0.00 | 0.00 | 0.00 | 0.01 | 0.30 | 0.03 | 0.00 | 0.00 | 0.00 | 0.00 | 0.00 | *Proteobacteria* | *Betaproteobacteria* | *Rhodocyclales* | *Rhodocyclaceae* | Transient-rare taxa |
| OTU 192 | 0.01 | 0.00 | 0.00 | 0.00 | 0.00 | 0.00 | 0.00 | 0.00 | 0.00 | 0.31 | 0.02 | 0.00 | *Bacteroidetes* | *Flavobacteriia* | *Flavobacteriales* | *Flavobacteriaceae* | Transient-rare taxa |
| OTU 201 | 0.00 | 0.00 | 0.00 | 0.00 | 0.00 | 0.00 | 0.00 | 0.00 | 0.01 | 0.00 | 0.30 | 0.01 | *Proteobacteria* | *Deltaproteobacteria* | *Myxococcales* | *Sorangiineae* | Transient-rare taxa |
| OTU 203 | 0.00 | 0.00 | 0.00 | 0.00 | 0.00 | 0.03 | 0.00 | 0.28 | 0.00 | 0.01 | 0.00 | 0.00 | *Bacteroidetes* | *Sphingobacteriia* | *Sphingobacteriales* | *Saprospiraceae* | Transient-rare taxa |
| OTU 205 | 0.00 | 0.00 | 0.17 | 0.12 | 0.00 | 0.00 | 0.03 | 0.00 | 0.00 | 0.00 | 0.00 | 0.00 | *Bacteroidetes* | *Flavobacteriia* | *Flavobacteriales* | *Flavobacteriaceae* | Transient-rare taxa |
| OTU 207 | 0.00 | 0.00 | 0.00 | 0.00 | 0.00 | 0.00 | 0.00 | 0.00 | 0.00 | 0.02 | 0.14 | 0.16 | *Proteobacteria* | *Deltaproteobacteria* | *Myxococcales* | *Nannocystineae* | Transient-rare taxa |
| OTU 208 | 0.08 | 0.00 | 0.00 | 0.00 | 0.00 | 0.00 | 0.00 | 0.00 | 0.00 | 0.00 | 0.00 | 0.24 | *Bacteroidetes* | *Sphingobacteriia* | *Sphingobacteriales* | *Saprospiraceae* | Transient-rare taxa |
| OTU 220 | 0.00 | 0.30 | 0.00 | 0.00 | 0.00 | 0.00 | 0.00 | 0.00 | 0.00 | 0.00 | 0.00 | 0.00 | *Bacteroidetes* | *Sphingobacteriia* | *Sphingobacteriales* | *Saprospiraceae* | Transient-rare taxa |
| OTU 221 | 0.00 | 0.00 | 0.00 | 0.00 | 0.19 | 0.07 | 0.04 | 0.00 | 0.00 | 0.00 | 0.00 | 0.00 | *Proteobacteria* | *Alphaproteobacteria* | *Rhodobacterales* | *Rhodobacteraceae* | Transient-rare taxa |
| OTU 225 | 0.28 | 0.00 | 0.00 | 0.00 | 0.00 | 0.00 | 0.00 | 0.00 | 0.00 | 0.00 | 0.00 | 0.02 | *Proteobacteria* | *Deltaproteobacteria* | *Myxococcales* | *Sorangiineae* | Transient-rare taxa |
| OTU 232 | 0.00 | 0.00 | 0.00 | 0.00 | 0.00 | 0.26 | 0.03 | 0.00 | 0.00 | 0.00 | 0.00 | 0.00 | *Proteobacteria* | *Deltaproteobacteria* | *Myxococcales* | *Nannocystineae* | Transient-rare taxa |
| OTU 235 | 0.00 | 0.00 | 0.00 | 0.00 | 0.00 | 0.28 | 0.01 | 0.00 | 0.00 | 0.00 | 0.00 | 0.00 | *Proteobacteria* | *Deltaproteobacteria* | *Myxococcales* | *Cystobacterineae* | Transient-rare taxa |
| OTU 236 | 0.00 | 0.00 | 0.29 | 0.00 | 0.00 | 0.00 | 0.00 | 0.00 | 0.00 | 0.00 | 0.00 | 0.00 | *Proteobacteria* | *Gammaproteobacteria* | *Gammaproteobacteria_incertae_sedis* | *Porticoccus* | Transient-rare taxa |
| OTU 247 | 0.24 | 0.00 | 0.00 | 0.00 | 0.00 | 0.00 | 0.00 | 0.01 | 0.02 | 0.00 | 0.00 | 0.00 | *Proteobacteria* | *Gammaproteobacteria* | *Thiotrichales* | *Thiotrichaceae* | Transient-rare taxa |
| OTU 253 | 0.00 | 0.00 | 0.00 | 0.00 | 0.27 | 0.00 | 0.00 | 0.00 | 0.00 | 0.00 | 0.00 | 0.00 | *Proteobacteria* | *Gammaproteobacteria* | *Gammaproteobacteria_incertae_sedis* | *Sedimenticola* | Transient-rare taxa |
| OTU 262 | 0.27 | 0.00 | 0.00 | 0.00 | 0.00 | 0.00 | 0.00 | 0.00 | 0.00 | 0.00 | 0.00 | 0.00 | *Proteobacteria* | *Deltaproteobacteria* | *Bdellovibrionales* | *Bacteriovoracaceae* | Transient-rare taxa |
| OTU 274 | 0.00 | 0.00 | 0.00 | 0.22 | 0.00 | 0.00 | 0.03 | 0.00 | 0.00 | 0.00 | 0.00 | 0.00 | *Proteobacteria* | *Alphaproteobacteria* | *Rhizobiales* | *Rhodobiaceae* | Transient-rare taxa |
| OTU 277 | 0.00 | 0.00 | 0.24 | 0.00 | 0.00 | 0.00 | 0.00 | 0.00 | 0.00 | 0.00 | 0.00 | 0.00 | *Bacteroidetes* | *Bacteroidia* | *Bacteroidales* | *Marinilabiliaceae* | Transient-rare taxa |
| OTU 278 | 0.00 | 0.00 | 0.22 | 0.00 | 0.00 | 0.00 | 0.02 | 0.00 | 0.00 | 0.00 | 0.00 | 0.00 | *Proteobacteria* | *Gammaproteobacteria* | *Oceanospirillales* | *Hahellaceae* | Transient-rare taxa |
| OTU 289 | 0.00 | 0.00 | 0.00 | 0.23 | 0.00 | 0.01 | 0.00 | 0.00 | 0.00 | 0.00 | 0.00 | 0.00 | *Proteobacteria* | *Gammaproteobacteria* | *Gammaproteobacteria_incertae_sedis* | *Porticoccus* | Transient-rare taxa |
| OTU 290 | 0.00 | 0.00 | 0.00 | 0.00 | 0.00 | 0.24 | 0.00 | 0.00 | 0.00 | 0.00 | 0.00 | 0.00 | *Firmicutes* | *Bacilli* | *Lactobacillales* | *Carnobacteriaceae* | Transient-rare taxa |
| OTU 292 | 0.00 | 0.00 | 0.23 | 0.00 | 0.00 | 0.00 | 0.00 | 0.00 | 0.00 | 0.01 | 0.00 | 0.00 | *Proteobacteria* | *Betaproteobacteria* | *Hydrogenophilales* | *Hydrogenophilaceae* | Transient-rare taxa |
| OTU 302 | 0.00 | 0.00 | 0.00 | 0.00 | 0.00 | 0.00 | 0.00 | 0.00 | 0.00 | 0.00 | 0.00 | 0.23 | *Proteobacteria* | *Deltaproteobacteria* | *Myxococcales* | *Sorangiineae* | Transient-rare taxa |
| OTU 319 | 0.00 | 0.00 | 0.00 | 0.00 | 0.07 | 0.16 | 0.00 | 0.00 | 0.00 | 0.00 | 0.00 | 0.00 | *Proteobacteria* | *Alphaproteobacteria* | *Rhodobacterales* | *Rhodobacteraceae* | Transient-rare taxa |
| OTU 336 | 0.00 | 0.00 | 0.00 | 0.00 | 0.01 | 0.20 | 0.00 | 0.00 | 0.00 | 0.00 | 0.00 | 0.00 | *Proteobacteria* | *Deltaproteobacteria* | *Desulfobacterales* | *Desulfobacteraceae* | Transient-rare taxa |
| OTU 337 | 0.00 | 0.00 | 0.00 | 0.00 | 0.13 | 0.08 | 0.00 | 0.00 | 0.00 | 0.00 | 0.00 | 0.00 | *Proteobacteria* | *Alphaproteobacteria* | *Rhodobacterales* | *Rhodobacteraceae* | Transient-rare taxa |
| OTU 346 | 0.10 | 0.10 | 0.00 | 0.00 | 0.00 | 0.00 | 0.00 | 0.00 | 0.00 | 0.00 | 0.00 | 0.00 | *Proteobacteria* | *Gammaproteobacteria* | *Chromatiales* | *Chromatiaceae* | Transient-rare taxa |
| OTU 348 | 0.00 | 0.00 | 0.00 | 0.00 | 0.09 | 0.11 | 0.00 | 0.00 | 0.00 | 0.00 | 0.00 | 0.00 | *Proteobacteria* | *Alphaproteobacteria* | *Rhodobacterales* | *Rhodobacteraceae* | Transient-rare taxa |
| OTU 353 | 0.20 | 0.00 | 0.00 | 0.00 | 0.00 | 0.00 | 0.00 | 0.00 | 0.00 | 0.00 | 0.00 | 0.00 | *Proteobacteria* | *Deltaproteobacteria* | *Myxococcales* | *Nannocystineae* | Transient-rare taxa |
| OTU 383 | 0.00 | 0.00 | 0.00 | 0.00 | 0.00 | 0.00 | 0.18 | 0.00 | 0.00 | 0.00 | 0.00 | 0.00 | *Proteobacteria* | *Alphaproteobacteria* | *Rhodospirillales* | *Rhodospirillaceae* | Transient-rare taxa |
| OTU 385 | 0.00 | 0.00 | 0.00 | 0.00 | 0.00 | 0.00 | 0.18 | 0.00 | 0.00 | 0.00 | 0.00 | 0.00 | *Proteobacteria* | *Gammaproteobacteria* | *Chromatiales* | *Chromatiaceae* | Transient-rare taxa |
| OTU 390 | 0.00 | 0.00 | 0.00 | 0.00 | 0.00 | 0.00 | 0.00 | 0.00 | 0.00 | 0.00 | 0.18 | 0.00 | *Bacteroidetes* | *Cytophagia* | *Cytophagales* | *Cytophagaceae* | Transient-rare taxa |
| OTU 402 | 0.18 | 0.00 | 0.00 | 0.00 | 0.00 | 0.00 | 0.00 | 0.00 | 0.00 | 0.00 | 0.00 | 0.00 | *Proteobacteria* | *Betaproteobacteria* | *Rhodocyclales* | *Rhodocyclaceae* | Transient-rare taxa |

| | | | | | | | | | | | | | | | | | |
|---|---|---|---|---|---|---|---|---|---|---|---|---|---|---|---|---|---|
| OTU 405 | 0.00 | 0.00 | 0.18 | 0.00 | 0.00 | 0.00 | 0.00 | 0.00 | 0.00 | 0.00 | 0.00 | 0.00 | *Proteobacteria* | *Alphaproteobacteria* | *Rhodobacterales* | *Rhodobacteraceae* | Transient-rare taxa |
| OTU 419 | 0.00 | 0.00 | 0.17 | 0.00 | 0.00 | 0.00 | 0.00 | 0.00 | 0.00 | 0.00 | 0.00 | 0.00 | *Proteobacteria* | *Betaproteobacteria* | *Hydrogenophilales* | *Hydrogenophilaceae* | Transient-rare taxa |
| OTU 420 | 0.00 | 0.00 | 0.00 | 0.00 | 0.00 | 0.00 | 0.00 | 0.00 | 0.00 | 0.00 | 0.00 | 0.17 | *Proteobacteria* | *Betaproteobacteria* | *Rhodocyclales* | *Rhodocyclaceae* | Transient-rare taxa |
| OTU 444 | 0.00 | 0.16 | 0.00 | 0.00 | 0.00 | 0.00 | 0.00 | 0.00 | 0.00 | 0.00 | 0.00 | 0.00 | *Planctomycetes* | *Planctomycetia* | *Planctomycetales* | *Planctomycetaceae* | Transient-rare taxa |
| OTU 479 | 0.00 | 0.00 | 0.14 | 0.00 | 0.00 | 0.00 | 0.00 | 0.00 | 0.00 | 0.00 | 0.00 | 0.00 | *Proteobacteria* | *Betaproteobacteria* | *Hydrogenophilales* | *Hydrogenophilaceae* | Transient-rare taxa |
| OTU 492 | 0.00 | 0.00 | 0.00 | 0.00 | 0.00 | 0.00 | 0.00 | 0.00 | 0.00 | 0.00 | 0.13 | 0.00 | *Proteobacteria* | *Deltaproteobacteria* | *Myxococcales* | *Sorangiineae* | Transient-rare taxa |
| OTU 520 | 0.00 | 0.00 | 0.00 | 0.00 | 0.00 | 0.00 | 0.00 | 0.00 | 0.00 | 0.00 | 0.13 | 0.00 | *Bacteroidetes* | *Sphingobacteriia* | *Sphingobacteriales* | *Chitinophagaceae* | Transient-rare taxa |
| OTU 546 | 0.00 | 0.00 | 0.00 | 0.12 | 0.00 | 0.00 | 0.00 | 0.00 | 0.00 | 0.00 | 0.00 | 0.00 | *Proteobacteria* | *Alphaproteobacteria* | *Sphingomonadales* | *Sphingomonadaceae* | Transient-rare taxa |
| OTU 548 | 0.00 | 0.00 | 0.00 | 0.00 | 0.00 | 0.12 | 0.00 | 0.00 | 0.00 | 0.00 | 0.00 | 0.00 | *Proteobacteria* | *Alphaproteobacteria* | *Rhodospirillales* | *Rhodospirillaceae* | Transient-rare taxa |
| OTU 256 | 0.00 | 0.16 | 0.00 | 0.00 | 0.00 | 0.00 | 0.00 | 0.02 | 0.09 | 0.00 | 0.00 | 0.00 | *Proteobacteria* | *Deltaproteobacteria* | *Myxococcales* | *Sorangiineae* | Transient-rare taxa |
| OTU 270 | 0.07 | 0.00 | 0.00 | 0.00 | 0.00 | 0.00 | 0.00 | 0.00 | 0.00 | 0.00 | 0.17 | 0.02 | *Proteobacteria* | *Gammaproteobacteria* | *Oceanospirillales* | *Halomonadaceae* | Transient-rare taxa |
| OTU 271 | 0.07 | 0.03 | 0.00 | 0.00 | 0.00 | 0.00 | 0.00 | 0.00 | 0.00 | 0.00 | 0.00 | 0.16 | *Proteobacteria* | *Betaproteobacteria* | *Rhodocyclales* | *Rhodocyclaceae* | Transient-rare taxa |
| OTU 282 | 0.04 | 0.00 | 0.00 | 0.00 | 0.00 | 0.00 | 0.00 | 0.00 | 0.00 | 0.00 | 0.16 | 0.04 | *Proteobacteria* | *Gammaproteobacteria* | *Pseudomonadales* | *Moraxellaceae* | Transient-rare taxa |
| OTU 285 | 0.02 | 0.00 | 0.00 | 0.00 | 0.00 | 0.00 | 0.00 | 0.00 | 0.07 | 0.16 | 0.00 | 0.00 | *Proteobacteria* | *Gammaproteobacteria* | *Xanthomonadales* | *Xanthomonadaceae* | Transient-rare taxa |
| OTU 295 | 0.08 | 0.00 | 0.00 | 0.00 | 0.00 | 0.00 | 0.00 | 0.00 | 0.00 | 0.00 | 0.12 | 0.03 | *Proteobacteria* | *Deltaproteobacteria* | *Myxococcales* | *Nannocystineae* | Transient-rare taxa |
| OTU 300 | 0.00 | 0.00 | 0.00 | 0.00 | 0.02 | 0.19 | 0.02 | 0.00 | 0.00 | 0.00 | 0.00 | 0.00 | *Proteobacteria* | *Alphaproteobacteria* | *Sphingomonadales* | *Sphingomonadaceae* | Transient-rare taxa |
| OTU 324 | 0.00 | 0.00 | 0.00 | 0.00 | 0.06 | 0.14 | 0.02 | 0.00 | 0.00 | 0.00 | 0.00 | 0.00 | *Proteobacteria* | *Betaproteobacteria* | *Burkholderiales* | *Burkholderiales_incertae_sedis* | Transient-rare taxa |
| OTU 327 | 0.00 | 0.00 | 0.00 | 0.00 | 0.01 | 0.00 | 0.00 | 0.00 | 0.00 | 0.00 | 0.19 | 0.01 | *Proteobacteria* | *Deltaproteobacteria* | *Myxococcales* | *Sorangiineae* | Transient-rare taxa |
| OTU 360 | 0.01 | 0.00 | 0.00 | 0.00 | 0.00 | 0.00 | 0.00 | 0.00 | 0.00 | 0.00 | 0.14 | 0.03 | *Bacteroidetes* | *Flavobacteriia* | *Flavobacteriales* | *Flavobacteriaceae* | Transient-rare taxa |
| OTU 363 | 0.00 | 0.00 | 0.04 | 0.00 | 0.00 | 0.00 | 0.00 | 0.11 | 0.03 | 0.00 | 0.00 | 0.00 | *Actinobacteria* | *Actinobacteria* | *Acidimicrobidae* | *Acidimicrobiales* | Transient-rare taxa |
| OTU 365 | 0.00 | 0.00 | 0.00 | 0.00 | 0.03 | 0.16 | 0.00 | 0.00 | 0.00 | 0.00 | 0.00 | 0.00 | *Planctomycetes* | *Planctomycetia* | *Planctomycetales* | *Planctomycetaceae* | Transient-rare taxa |
| OTU 366 | 0.00 | 0.00 | 0.08 | 0.00 | 0.00 | 0.00 | 0.00 | 0.09 | 0.02 | 0.00 | 0.00 | 0.00 | *Proteobacteria* | *Deltaproteobacteria* | *Myxococcales* | *Nannocystineae* | Transient-rare taxa |
| OTU 368 | 0.00 | 0.02 | 0.00 | 0.00 | 0.00 | 0.00 | 0.00 | 0.14 | 0.02 | 0.00 | 0.00 | 0.00 | *Proteobacteria* | *Deltaproteobacteria* | *Myxococcales* | *Nannocystineae* | Transient-rare taxa |
| OTU 375 | 0.17 | 0.00 | 0.00 | 0.00 | 0.00 | 0.00 | 0.00 | 0.00 | 0.00 | 0.00 | 0.00 | 0.02 | *Bacteroidetes* | *Sphingobacteriia* | *Sphingobacteriales* | *Saprospiraceae* | Transient-rare taxa |
| OTU 376 | 0.13 | 0.00 | 0.00 | 0.00 | 0.00 | 0.00 | 0.00 | 0.00 | 0.00 | 0.00 | 0.00 | 0.06 | *Proteobacteria* | *Gammaproteobacteria* | *Xanthomonadales* | *Xanthomonadaceae* | Transient-rare taxa |
| OTU 378 | 0.00 | 0.00 | 0.00 | 0.00 | 0.01 | 0.00 | 0.00 | 0.00 | 0.00 | 0.00 | 0.02 | 0.16 | *Proteobacteria* | *Alphaproteobacteria* | *Eilatimonas* | | Transient-rare taxa |
| OTU 386 | 0.03 | 0.00 | 0.00 | 0.00 | 0.00 | 0.00 | 0.00 | 0.00 | 0.00 | 0.00 | 0.07 | 0.08 | *Bacteroidetes* | *Sphingobacteriia* | *Sphingobacteriales* | *Sphingobacteriaceae* | Transient-rare taxa |
| OTU 389 | 0.07 | 0.00 | 0.00 | 0.00 | 0.00 | 0.00 | 0.00 | 0.00 | 0.00 | 0.00 | 0.08 | 0.03 | *Bacteroidetes* | *Flavobacteriia* | *Flavobacteriales* | *Flavobacteriaceae* | Transient-rare taxa |
| OTU 403 | 0.00 | 0.00 | 0.02 | 0.06 | 0.00 | 0.00 | 0.00 | 0.00 | 0.00 | 0.10 | 0.00 | 0.00 | *Proteobacteria* | *Epsilonproteobacteria* | *Campylobacterales* | *Helicobacteraceae* | Transient-rare taxa |
| OTU 413 | 0.06 | 0.10 | 0.01 | 0.00 | 0.00 | 0.00 | 0.00 | 0.00 | 0.00 | 0.00 | 0.00 | 0.00 | *Proteobacteria* | *Gammaproteobacteria* | *Chromatiales* | *Chromatiaceae* | Transient-rare taxa |
| OTU 416 | 0.00 | 0.00 | 0.00 | 0.01 | 0.00 | 0.16 | 0.00 | 0.00 | 0.00 | 0.00 | 0.00 | 0.00 | *Proteobacteria* | *Gammaproteobacteria* | *Pseudomonadales* | *Moraxellaceae* | Transient-rare taxa |
| OTU 417 | 0.00 | 0.00 | 0.00 | 0.00 | 0.00 | 0.00 | 0.00 | 0.00 | 0.00 | 0.00 | 0.10 | 0.07 | *Proteobacteria* | *Alphaproteobacteria* | *Rhizobiales* | *Phyllobacteriaceae* | Transient-rare taxa |
| OTU 418 | 0.08 | 0.00 | 0.00 | 0.00 | 0.00 | 0.00 | 0.00 | 0.00 | 0.00 | 0.00 | 0.08 | 0.01 | *Bacteroidetes* | *Sphingobacteriia* | *Sphingobacteriales* | *Sphingobacteriaceae* | Transient-rare taxa |
| OTU 421 | 0.00 | 0.00 | 0.00 | 0.00 | 0.00 | 0.00 | 0.00 | 0.00 | 0.00 | 0.00 | 0.12 | 0.04 | *Bacteroidetes* | *Flavobacteriia* | *Flavobacteriales* | *Flavobacteriaceae* | Transient-rare taxa |
| OTU 427 | 0.07 | 0.03 | 0.00 | 0.00 | 0.00 | 0.00 | 0.00 | 0.00 | 0.00 | 0.00 | 0.00 | 0.06 | *Proteobacteria* | *Betaproteobacteria* | *Rhodocyclales* | *Rhodocyclaceae* | Transient-rare taxa |
| OTU 428 | 0.00 | 0.14 | 0.01 | 0.00 | 0.00 | 0.00 | 0.00 | 0.00 | 0.00 | 0.00 | 0.00 | 0.00 | *Parcubacteria* | *Parcubacteria_genera_incertae_sedis* | | | Transient-rare taxa |
| OTU 430 | 0.00 | 0.08 | 0.07 | 0.00 | 0.00 | 0.00 | 0.00 | 0.00 | 0.00 | 0.01 | 0.00 | 0.00 | *Planctomycetes* | *Planctomycetia* | *Planctomycetales* | *Planctomycetaceae* | Transient-rare taxa |
| OTU 432 | 0.00 | 0.00 | 0.00 | 0.00 | 0.00 | 0.00 | 0.00 | 0.00 | 0.00 | 0.00 | 0.07 | 0.09 | *Bacteroidetes* | *Flavobacteriia* | *Flavobacteriales* | *Cryomorphaceae* | Transient-rare taxa |
| OTU 435 | 0.08 | 0.08 | 0.00 | 0.00 | 0.00 | 0.00 | 0.00 | 0.00 | 0.00 | 0.00 | 0.00 | 0.00 | *Proteobacteria* | *Gammaproteobacteria* | *Chromatiales* | *Chromatiaceae* | Transient-rare taxa |
| OTU 443 | 0.00 | 0.00 | 0.00 | 0.00 | 0.09 | 0.07 | 0.00 | 0.00 | 0.00 | 0.00 | 0.00 | 0.00 | *Proteobacteria* | *Deltaproteobacteria* | *Myxococcales* | *Sorangiineae* | Transient-rare taxa |
| OTU 450 | 0.00 | 0.00 | 0.00 | 0.00 | 0.00 | 0.00 | 0.00 | 0.00 | 0.00 | 0.01 | 0.00 | 0.14 | *Proteobacteria* | *Betaproteobacteria* | *Burkholderiales* | *Burkholderiales_incertae_sedis* | Transient-rare taxa |
| OTU 451 | 0.00 | 0.01 | 0.14 | 0.00 | 0.00 | 0.00 | 0.00 | 0.00 | 0.00 | 0.00 | 0.00 | 0.00 | *Planctomycetes* | *Planctomycetia* | *Planctomycetales* | *Planctomycetaceae* | Transient-rare taxa |
| OTU 452 | 0.00 | 0.01 | 0.14 | 0.00 | 0.00 | 0.00 | 0.00 | 0.00 | 0.00 | 0.00 | 0.00 | 0.00 | *Proteobacteria* | *Betaproteobacteria* | *Neisseriales* | *Neisseriaceae* | Transient-rare taxa |
| OTU 454 | 0.00 | 0.00 | 0.00 | 0.00 | 0.00 | 0.00 | 0.00 | 0.04 | 0.09 | 0.01 | 0.00 | 0.00 | *Proteobacteria* | *Deltaproteobacteria* | *Myxococcales* | *Nannocystineae* | Transient-rare taxa |
| OTU 459 | 0.00 | 0.00 | 0.00 | 0.00 | 0.02 | 0.12 | 0.00 | 0.00 | 0.00 | 0.00 | 0.00 | 0.00 | *Proteobacteria* | *Betaproteobacteria* | *Rhodocyclales* | *Rhodocyclaceae* | Transient-rare taxa |
| OTU 460 | 0.00 | 0.00 | 0.00 | 0.03 | 0.00 | 0.00 | 0.00 | 0.06 | 0.06 | 0.00 | 0.00 | 0.00 | *Proteobacteria* | *Alphaproteobacteria* | *Rickettsiales* | *Anaplasmataceae* | Transient-rare taxa |
| OTU 466 | 0.00 | 0.00 | 0.00 | 0.00 | 0.00 | 0.00 | 0.00 | 0.00 | 0.01 | 0.00 | 0.03 | 0.10 | *Bacteroidetes* | *Flavobacteriia* | *Flavobacteriales* | *Flavobacteriaceae* | Transient-rare taxa |
| OTU 467 | 0.00 | 0.06 | 0.00 | 0.06 | 0.00 | 0.00 | 0.00 | 0.03 | 0.00 | 0.00 | 0.00 | 0.00 | *Bacteroidetes* | *Flavobacteriia* | *Flavobacteriales* | *Cryomorphaceae* | Transient-rare taxa |
| OTU 469 | 0.00 | 0.00 | 0.02 | 0.11 | 0.00 | 0.01 | 0.00 | 0.00 | 0.00 | 0.00 | 0.00 | 0.00 | *Bacteroidetes* | *Flavobacteriia* | *Flavobacteriales* | *Flavobacteriaceae* | Transient-rare taxa |
| OTU 472 | 0.02 | 0.08 | 0.00 | 0.00 | 0.00 | 0.00 | 0.00 | 0.00 | 0.00 | 0.00 | 0.00 | 0.04 | *Proteobacteria* | *Betaproteobacteria* | *Rhodocyclales* | *Rhodocyclaceae* | Transient-rare taxa |
| OTU 473 | 0.00 | 0.00 | 0.13 | 0.00 | 0.00 | 0.00 | 0.01 | 0.00 | 0.00 | 0.00 | 0.00 | 0.00 | *Proteobacteria* | *Gammaproteobacteria* | *Gammaproteobacteria_incertae_sedis* | *Thiohalophilus* | Transient-rare taxa |
| OTU 474 | 0.04 | 0.00 | 0.00 | 0.00 | 0.00 | 0.00 | 0.00 | 0.00 | 0.00 | 0.00 | 0.00 | 0.10 | *Proteobacteria* | *Betaproteobacteria* | *Rhodocyclales* | *Rhodocyclaceae* | Transient-rare taxa |
| OTU 476 | 0.03 | 0.00 | 0.00 | 0.00 | 0.00 | 0.00 | 0.00 | 0.00 | 0.00 | 0.00 | 0.00 | 0.11 | *Proteobacteria* | *Deltaproteobacteria* | *Myxococcales* | *Sorangiineae* | Transient-rare taxa |
| OTU 480 | 0.01 | 0.00 | 0.12 | 0.00 | 0.00 | 0.01 | 0.00 | 0.00 | 0.00 | 0.00 | 0.00 | 0.00 | *Bacteroidetes* | *Flavobacteriia* | *Flavobacteriales* | *Cryomorphaceae* | Transient-rare taxa |
| OTU 481 | 0.00 | 0.00 | 0.00 | 0.00 | 0.00 | 0.00 | 0.00 | 0.04 | 0.07 | 0.02 | 0.00 | 0.00 | *Bacteroidetes* | *Sphingobacteriia* | *Sphingobacteriales* | *Chitinophagaceae* | Transient-rare taxa |
| OTU 483 | 0.00 | 0.00 | 0.12 | 0.00 | 0.00 | 0.00 | 0.01 | 0.00 | 0.00 | 0.00 | 0.00 | 0.00 | *Proteobacteria* | *Epsilonproteobacteria* | *Campylobacterales* | *Helicobacteraceae* | Transient-rare taxa |
| OTU 486 | 0.00 | 0.00 | 0.00 | 0.00 | 0.11 | 0.01 | 0.00 | 0.00 | 0.01 | 0.00 | 0.00 | 0.00 | *Proteobacteria* | *Betaproteobacteria* | *Rhodocyclales* | *Rhodocyclaceae* | Transient-rare taxa |
| OTU 493 | 0.06 | 0.00 | 0.00 | 0.00 | 0.00 | 0.00 | 0.00 | 0.00 | 0.00 | 0.00 | 0.07 | 0.01 | *Proteobacteria* | *Deltaproteobacteria* | *Myxococcales* | *Nannocystineae* | Transient-rare taxa |
| OTU 498 | 0.00 | 0.00 | 0.00 | 0.06 | 0.07 | 0.00 | 0.01 | 0.00 | 0.00 | 0.00 | 0.00 | 0.00 | *Proteobacteria* | *Betaproteobacteria* | *Burkholderiales* | *Comamonadaceae* | Transient-rare taxa |
| OTU 500 | 0.00 | 0.00 | 0.00 | 0.09 | 0.01 | 0.03 | 0.00 | 0.00 | 0.00 | 0.00 | 0.00 | 0.00 | *Proteobacteria* | *Gammaproteobacteria* | *Oceanospirillales* | *Oceanospirillaceae* | Transient-rare taxa |
| OTU 504 | 0.00 | 0.00 | 0.00 | 0.00 | 0.00 | 0.00 | 0.00 | 0.12 | 0.01 | 0.00 | 0.00 | 0.00 | *Proteobacteria* | *Deltaproteobacteria* | *Myxococcales* | *Nannocystineae* | Transient-rare taxa |
| OTU 505 | 0.00 | 0.00 | 0.00 | 0.12 | 0.00 | 0.00 | 0.01 | 0.00 | 0.00 | 0.00 | 0.00 | 0.00 | *Proteobacteria* | *Deltaproteobacteria* | *Desulfuromonadales* | *Geobacteraceae* | Transient-rare taxa |
| OTU 508 | 0.00 | 0.00 | 0.00 | 0.00 | 0.00 | 0.00 | 0.00 | 0.00 | 0.12 | 0.01 | 0.00 | 0.00 | *Bacteroidetes* | *Sphingobacteriia* | *Sphingobacteriales* | *Saprospiraceae* | Transient-rare taxa |
| OTU 510 | 0.08 | 0.06 | 0.00 | 0.00 | 0.00 | 0.00 | 0.00 | 0.00 | 0.00 | 0.00 | 0.00 | 0.00 | *Proteobacteria* | *Betaproteobacteria* | *Rhodocyclales* | *Rhodocyclaceae* | Transient-rare taxa |
| OTU 511 | 0.06 | 0.00 | 0.00 | 0.00 | 0.00 | 0.00 | 0.00 | 0.07 | 0.00 | 0.00 | 0.00 | 0.01 | *Proteobacteria* | *Betaproteobacteria* | *Burkholderiales* | *Comamonadaceae* | Transient-rare taxa |
| OTU 513 | 0.00 | 0.00 | 0.01 | 0.12 | 0.00 | 0.00 | 0.00 | 0.00 | 0.00 | 0.00 | 0.00 | 0.00 | *Bacteroidetes* | *Flavobacteriia* | *Flavobacteriales* | *Flavobacteriaceae* | Transient-rare taxa |
| OTU 515 | 0.02 | 0.00 | 0.00 | 0.00 | 0.00 | 0.00 | 0.00 | 0.00 | 0.00 | 0.00 | 0.07 | 0.04 | *Proteobacteria* | *Gammaproteobacteria* | *Oceanospirillales* | *Halomonadaceae* | Transient-rare taxa |
| OTU 516 | 0.02 | 0.00 | 0.00 | 0.00 | 0.00 | 0.00 | 0.00 | 0.00 | 0.00 | 0.00 | 0.00 | 0.11 | *Proteobacteria* | *Betaproteobacteria* | *Rhodocyclales* | *Rhodocyclaceae* | Transient-rare taxa |
| OTU 526 | 0.00 | 0.00 | 0.00 | 0.01 | 0.00 | 0.10 | 0.01 | 0.00 | 0.00 | 0.00 | 0.00 | 0.00 | *Proteobacteria* | *Gammaproteobacteria* | *Gammaproteobacteria_incertae_sedis* | *Cocleimonas* | Transient-rare taxa |
| OTU 529 | 0.00 | 0.00 | 0.00 | 0.00 | 0.07 | 0.04 | 0.00 | 0.00 | 0.00 | 0.00 | 0.01 | 0.00 | *Proteobacteria* | *Alphaproteobacteria* | *Rhodobacterales* | *Rhodobacteraceae* | Transient-rare taxa |
| OTU 530 | 0.00 | 0.10 | 0.00 | 0.00 | 0.00 | 0.00 | 0.00 | 0.00 | 0.01 | 0.01 | 0.00 | 0.00 | *Bacteroidetes* | *Cytophagia* | *Cytophagales* | *Flammeovirgaceae* | Transient-rare taxa |
| OTU 531 | 0.00 | 0.03 | 0.06 | 0.00 | 0.00 | 0.00 | 0.00 | 0.00 | 0.03 | 0.00 | 0.00 | 0.00 | *Planctomycetes* | *Planctomycetia* | *Planctomycetales* | *Planctomycetaceae* | Transient-rare taxa |
| OTU 543 | 0.00 | 0.00 | 0.00 | 0.02 | 0.10 | 0.00 | 0.00 | 0.00 | 0.00 | 0.00 | 0.00 | 0.00 | *Bacteroidetes* | *Flavobacteriia* | *Flavobacteriales* | *Cryomorphaceae* | Transient-rare taxa |
| OTU 552 | 0.00 | 0.00 | 0.00 | 0.04 | 0.04 | 0.03 | 0.00 | 0.00 | 0.00 | 0.00 | 0.00 | 0.00 | *Proteobacteria* | *Gammaproteobacteria* | *Chromatiales* | *Ectothiorhodospiraceae* | Transient-rare taxa |
| OTU 553 | 0.11 | 0.00 | 0.01 | 0.00 | 0.00 | 0.00 | 0.00 | 0.00 | 0.00 | 0.00 | 0.00 | 0.00 | *Proteobacteria* | *Deltaproteobacteria* | *Myxococcales* | *Nannocystineae* | Transient-rare taxa |
| OTU 556 | 0.01 | 0.00 | 0.00 | 0.00 | 0.00 | 0.00 | 0.00 | 0.00 | 0.03 | 0.08 | 0.00 | 0.00 | *Proteobacteria* | *Gammaproteobacteria* | *Xanthomonadales* | *Xanthomonadaceae* | Transient-rare taxa |
| OTU 557 | 0.03 | 0.00 | 0.00 | 0.00 | 0.01 | 0.00 | 0.00 | 0.00 | 0.00 | 0.08 | 0.00 | 0.00 | *Proteobacteria* | *Gammaproteobacteria* | *Xanthomonadales* | *Xanthomonadaceae* | Transient-rare taxa |
| OTU 561 | 0.00 | 0.00 | 0.00 | 0.00 | 0.00 | 0.00 | 0.00 | 0.00 | 0.03 | 0.00 | 0.08 | 0.00 | *Proteobacteria* | *Deltaproteobacteria* | *Myxococcales* | *Sorangiineae* | Transient-rare taxa |
| OTU 567 | 0.00 | 0.00 | 0.00 | 0.00 | 0.07 | 0.02 | 0.02 | 0.00 | 0.00 | 0.00 | 0.00 | 0.00 | *Proteobacteria* | *Alphaproteobacteria* | *Rhodobacterales* | *Rhodobacteraceae* | Transient-rare taxa |
| OTU 568 | 0.00 | 0.00 | 0.00 | 0.00 | 0.00 | 0.00 | 0.00 | 0.04 | 0.07 | 0.00 | 0.00 | 0.00 | *Planctomycetes* | *Planctomycetia* | *Planctomycetales* | *Planctomycetaceae* | Transient-rare taxa |
| OTU 570 | 0.00 | 0.03 | 0.00 | 0.00 | 0.00 | 0.00 | 0.01 | 0.00 | 0.00 | 0.07 | 0.00 | 0.00 | *Proteobacteria* | *Betaproteobacteria* | *Rhodocyclales* | *Rhodocyclaceae* | Transient-rare taxa |
| OTU 574 | 0.00 | 0.11 | 0.00 | 0.00 | 0.00 | 0.00 | 0.00 | 0.00 | 0.00 | 0.00 | 0.00 | 0.00 | *Proteobacteria* | *Gammaproteobacteria* | *Pseudomonadales* | *Moraxellaceae* | Transient-rare taxa |
| OTU 575 | 0.00 | 0.00 | 0.00 | 0.00 | 0.08 | 0.00 | 0.03 | 0.00 | 0.00 | 0.00 | 0.00 | 0.00 | *Bacteroidetes* | *Flavobacteriia* | *Flavobacteriales* | *Flavobacteriaceae* | Transient-rare taxa |
| OTU 581 | 0.04 | 0.07 | 0.00 | 0.00 | 0.00 | 0.00 | 0.00 | 0.00 | 0.00 | 0.00 | 0.00 | 0.00 | *Proteobacteria* | *Gammaproteobacteria* | *Chromatiales* | *Chromatiaceae* | Transient-rare taxa |
| OTU 586 | 0.00 | 0.02 | 0.00 | 0.00 | 0.00 | 0.00 | 0.00 | 0.00 | 0.04 | 0.04 | 0.00 | 0.00 | *Bacteroidetes* | *Sphingobacteriia* | *Sphingobacteriales* | *Saprospiraceae* | Transient-rare taxa |
| OTU 589 | 0.00 | 0.00 | 0.00 | 0.00 | 0.08 | 0.03 | 0.00 | 0.00 | 0.00 | 0.00 | 0.00 | 0.00 | *Bacteroidetes* | *Sphingobacteriia* | *Sphingobacteriales* | *Saprospiraceae* | Transient-rare taxa |
| OTU 592 | 0.01 | 0.00 | 0.00 | 0.00 | 0.00 | 0.00 | 0.00 | 0.00 | 0.02 | 0.00 | 0.08 | 0.00 | *Proteobacteria* | *Deltaproteobacteria* | *Myxococcales* | *Sorangiineae* | Transient-rare taxa |
| OTU 595 | 0.04 | 0.01 | 0.00 | 0.00 | 0.00 | 0.00 | 0.00 | 0.00 | 0.00 | 0.00 | 0.00 | 0.06 | *Proteobacteria* | *Betaproteobacteria* | *Burkholderiales* | *Burkholderiaceae* | Transient-rare taxa |
| OTU 599 | 0.03 | 0.08 | 0.00 | 0.00 | 0.00 | 0.00 | 0.00 | 0.00 | 0.00 | 0.00 | 0.00 | 0.00 | *Planctomycetes* | *Planctomycetia* | *Planctomycetales* | *Planctomycetaceae* | Transient-rare taxa |
| OTU 601 | 0.07 | 0.00 | 0.00 | 0.00 | 0.00 | 0.00 | 0.00 | 0.00 | 0.02 | 0.02 | 0.00 | 0.00 | *Proteobacteria* | *Betaproteobacteria* | *Rhodocyclales* | *Rhodocyclaceae* | Transient-rare taxa |
| OTU 602 | 0.03 | 0.00 | 0.00 | 0.00 | 0.00 | 0.00 | 0.00 | 0.00 | 0.00 | 0.00 | 0.00 | 0.08 | *Proteobacteria* | *Deltaproteobacteria* | *Myxococcales* | *Sorangiineae* | Transient-rare taxa |
| OTU 603 | 0.06 | 0.00 | 0.00 | 0.00 | 0.00 | 0.00 | 0.00 | 0.00 | 0.00 | 0.00 | 0.04 | 0.01 | *Proteobacteria* | *Deltaproteobacteria* | *Desulfuromonadales* | *Geobacteraceae* | Transient-rare taxa |
| OTU 604 | 0.09 | 0.00 | 0.00 | 0.01 | 0.00 | 0.00 | 0.00 | 0.00 | 0.00 | 0.00 | 0.01 | 0.00 | *Actinobacteria* | *Actinobacteria* | *Actinobacteridae* | *Actinomycetales* | Transient-rare taxa |
| OTU 605 | 0.03 | 0.00 | 0.00 | 0.00 | 0.00 | 0.00 | 0.00 | 0.00 | 0.00 | 0.00 | 0.00 | 0.08 | *Proteobacteria* | *Betaproteobacteria* | *Rhodocyclales* | *Rhodocyclaceae* | Transient-rare taxa |
| OTU 606 | 0.01 | 0.00 | 0.00 | 0.00 | 0.00 | 0.00 | 0.00 | 0.00 | 0.00 | 0.00 | 0.00 | 0.10 | *Bacteroidetes* | *Cytophagia* | *Cytophagales* | *Cytophagaceae* | Transient-rare taxa |
| OTU 607 | 0.00 | 0.00 | 0.00 | 0.00 | 0.00 | 0.00 | 0.00 | 0.00 | 0.00 | 0.00 | 0.00 | 0.11 | *Proteobacteria* | *Gammaproteobacteria* | *Chromatiales* | *Chromatiaceae* | Transient-rare taxa |
| OTU 608 | 0.03 | 0.00 | 0.00 | 0.00 | 0.00 | 0.00 | 0.00 | 0.00 | 0.00 | 0.00 | 0.00 | 0.08 | *Actinobacteria* | *Actinobacteria* | *Acidimicrobidae* | *Acidimicrobiales* | Transient-rare taxa |
| OTU 611 | 0.00 | 0.00 | 0.00 | 0.00 | 0.00 | 0.00 | 0.00 | 0.11 | 0.00 | 0.00 | 0.00 | 0.00 | *Proteobacteria* | *Deltaproteobacteria* | *Myxococcales* | *Nannocystineae* | Transient-rare taxa |
| OTU 615 | 0.00 | 0.00 | 0.01 | 0.00 | 0.00 | 0.00 | 0.00 | 0.03 | 0.06 | 0.00 | 0.00 | 0.00 | *Planctomycetes* | *Planctomycetia* | *Planctomycetales* | *Planctomycetaceae* | Transient-rare taxa |
| OTU 616 | 0.00 | 0.00 | 0.09 | 0.00 | 0.01 | 0.00 | 0.00 | 0.00 | 0.00 | 0.00 | 0.00 | 0.00 | *Proteobacteria* | *Gammaproteobacteria* | *Oceanospirillales* | *Oceanospirillaceae* | Transient-rare taxa |
| OTU 617 | 0.00 | 0.00 | 0.00 | 0.00 | 0.00 | 0.01 | 0.09 | 0.00 | 0.00 | 0.00 | 0.00 | 0.00 | *Planctomycetes* | *Planctomycetia* | *Planctomycetales* | *Planctomycetaceae* | Transient-rare taxa |
| OTU 618 | 0.01 | 0.00 | 0.00 | 0.00 | 0.09 | 0.00 | 0.00 | 0.00 | 0.00 | 0.00 | 0.00 | 0.00 | *Proteobacteria* | *Betaproteobacteria* | *Burkholderiales* | *Comamonadaceae* | Transient-rare taxa |
| OTU 621 | 0.00 | 0.09 | 0.00 | 0.00 | 0.00 | 0.00 | 0.00 | 0.00 | 0.00 | 0.01 | 0.00 | 0.00 | *Proteobacteria* | *Gammaproteobacteria* | *Chromatiales* | *Ectothiorhodospiraceae* | Transient-rare taxa |
| OTU 622 | 0.00 | 0.00 | 0.00 | 0.00 | 0.00 | 0.00 | 0.00 | 0.00 | 0.00 | 0.00 | 0.10 | 0.00 | *Proteobacteria* | *Deltaproteobacteria* | *Myxococcales* | *Sorangiineae* | Transient-rare taxa |
| OTU 625 | 0.00 | 0.00 | 0.00 | 0.00 | 0.00 | 0.00 | 0.00 | 0.00 | 0.07 | 0.00 | 0.03 | 0.00 | *Proteobacteria* | *Gammaproteobacteria* | *Chromatiales* | *Ectothiorhodospiraceae* | Transient-rare taxa |
| OTU 626 | 0.01 | 0.09 | 0.00 | 0.00 | 0.00 | 0.00 | 0.00 | 0.00 | 0.00 | 0.00 | 0.00 | 0.00 | *Proteobacteria* | *Betaproteobacteria* | *Rhodocyclales* | *Rhodocyclaceae* | Transient-rare taxa |
| OTU 627 | 0.00 | 0.00 | 0.00 | 0.00 | 0.06 | 0.04 | 0.00 | 0.00 | 0.00 | 0.00 | 0.00 | 0.00 | *Bacteroidetes* | *Sphingobacteriia* | *Sphingobacteriales* | *Saprospiraceae* | Transient-rare taxa |
| OTU 630 | 0.00 | 0.00 | 0.00 | 0.01 | 0.09 | 0.00 | 0.00 | 0.00 | 0.00 | 0.00 | 0.00 | 0.00 | *Bacteroidetes* | *Cytophagia* | *Cytophagales* | *Flammeovirgaceae* | Transient-rare taxa |
| OTU 631 | 0.02 | 0.06 | 0.00 | 0.00 | 0.00 | 0.00 | 0.00 | 0.00 | 0.00 | 0.00 | 0.00 | 0.02 | *Proteobacteria* | *Betaproteobacteria* | *Rhodocyclales* | *Rhodocyclaceae* | Transient-rare taxa |
| OTU 635 | 0.00 | 0.00 | 0.00 | 0.00 | 0.00 | 0.00 | 0.00 | 0.00 | 0.00 | 0.00 | 0.10 | 0.00 | *Proteobacteria* | *Deltaproteobacteria* | *Myxococcales* | *Nannocystineae* | Transient-rare taxa |
| OTU 639 | 0.00 | 0.00 | 0.00 | 0.00 | 0.00 | 0.00 | 0.00 | 0.00 | 0.02 | 0.01 | 0.00 | 0.07 | *Bacteroidetes* | *Cytophagia* | *Cytophagales* | *Flammeovirgaceae* | Transient-rare taxa |
| OTU 642 | 0.00 | 0.00 | 0.00 | 0.00 | 0.02 | 0.08 | 0.00 | 0.00 | 0.00 | 0.00 | 0.00 | 0.00 | *Proteobacteria* | *Gammaproteobacteria* | *Oceanospirillales* | *Halomonadaceae* | Transient-rare taxa |
| OTU 646 | 0.02 | 0.04 | 0.00 | 0.00 | 0.00 | 0.00 | 0.00 | 0.00 | 0.00 | 0.00 | 0.00 | 0.03 | *Planctomycetes* | *Planctomycetia* | *Planctomycetales* | *Planctomycetaceae* | Transient-rare taxa |
| OTU 647 | 0.00 | 0.00 | 0.00 | 0.00 | 0.00 | 0.08 | 0.02 | 0.00 | 0.00 | 0.00 | 0.00 | 0.00 | *Proteobacteria* | *Betaproteobacteria* | *Rhodocyclales* | *Rhodocyclaceae* | Transient-rare taxa |
| OTU 648 | 0.00 | 0.00 | 0.00 | 0.00 | 0.00 | 0.10 | 0.00 | 0.00 | 0.00 | 0.00 | 0.00 | 0.00 | *Bacteroidetes* | *Flavobacteriia* | *Flavobacteriales* | *Flavobacteriaceae* | Transient-rare taxa |
| OTU 649 | 0.08 | 0.00 | 0.00 | 0.00 | 0.00 | 0.00 | 0.00 | 0.02 | 0.00 | 0.00 | 0.00 | 0.00 | *Firmicutes* | *Bacilli* | *Bacillales* | *Bacillaceae 2* | Transient-rare taxa |
| OTU 650 | 0.00 | 0.00 | 0.01 | 0.00 | 0.00 | 0.00 | 0.00 | 0.08 | 0.01 | 0.00 | 0.00 | 0.00 | *Proteobacteria* | *Deltaproteobacteria* | *Myxococcales* | *Nannocystineae* | Transient-rare taxa |
| OTU 651 | 0.03 | 0.07 | 0.00 | 0.00 | 0.00 | 0.00 | 0.00 | 0.00 | 0.00 | 0.00 | 0.00 | 0.00 | *Bacteroidetes* | *Sphingobacteriia* | *Sphingobacteriales* | *Saprospiraceae* | Transient-rare taxa |
| OTU 652 | 0.10 | 0.00 | 0.00 | 0.00 | 0.00 | 0.00 | 0.00 | 0.00 | 0.00 | 0.00 | 0.00 | 0.00 | *Proteobacteria* | *Betaproteobacteria* | *Burkholderiales* | *Burkholderiales_incertae_sedis* | Transient-rare taxa |
| OTU 657 | 0.00 | 0.00 | 0.03 | 0.02 | 0.00 | 0.00 | 0.00 | 0.00 | 0.00 | 0.04 | 0.00 | 0.00 | *Proteobacteria* | *Deltaproteobacteria* | *Bdellovibrionales* | *Bacteriovoracaceae* | Transient-rare taxa |
| OTU 659 | 0.01 | 0.00 | 0.00 | 0.00 | 0.00 | 0.00 | 0.00 | 0.00 | 0.00 | 0.00 | 0.04 | 0.04 | *Proteobacteria* | *Gammaproteobacteria* | *Pseudomonadales* | *Pseudomonadaceae* | Transient-rare taxa |
| OTU 660 | 0.00 | 0.00 | 0.00 | 0.00 | 0.00 | 0.00 | 0.00 | 0.00 | 0.03 | 0.03 | 0.00 | 0.03 | *Proteobacteria* | *Deltaproteobacteria* | *Myxococcales* | *Cystobacterineae* | Transient-rare taxa |
| OTU 661 | 0.00 | 0.00 | 0.00 | 0.00 | 0.00 | 0.01 | 0.00 | 0.00 | 0.00 | 0.04 | 0.00 | 0.04 | *Proteobacteria* | *Betaproteobacteria* | *Rhodocyclales* | *Rhodocyclaceae* | Transient-rare taxa |
| OTU 662 | 0.09 | 0.00 | 0.00 | 0.00 | 0.00 | 0.00 | 0.00 | 0.00 | 0.00 | 0.00 | 0.00 | 0.01 | *Bacteroidetes* | *Cytophagia* | *Cytophagales* | *Cytophagaceae* | Transient-rare taxa |
| OTU 663 | 0.00 | 0.00 | 0.10 | 0.00 | 0.00 | 0.00 | 0.00 | 0.00 | 0.00 | 0.00 | 0.00 | 0.00 | *Proteobacteria* | *Gammaproteobacteria* | *Thiotrichales* | *Francisellaceae* | Transient-rare taxa |
| OTU 666 | 0.00 | 0.00 | 0.00 | 0.00 | 0.00 | 0.10 | 0.00 | 0.00 | 0.00 | 0.00 | 0.00 | 0.00 | *Proteobacteria* | *Deltaproteobacteria* | *Myxococcales* | *Nannocystineae* | Transient-rare taxa |
| OTU 667 | 0.00 | 0.00 | 0.00 | 0.00 | 0.00 | 0.10 | 0.00 | 0.00 | 0.00 | 0.00 | 0.00 | 0.00 | *Verrucomicrobia* | *Opitutae* | *Opitutales* | *Opitutaceae* | Transient-rare taxa |
| OTU 673 | 0.01 | 0.07 | 0.00 | 0.00 | 0.00 | 0.00 | 0.00 | 0.00 | 0.01 | 0.00 | 0.00 | 0.00 | *Proteobacteria* | *Betaproteobacteria* | *Neisseriales* | *Neisseriaceae* | Transient-rare taxa |
| OTU 674 | 0.00 | 0.00 | 0.00 | 0.00 | 0.00 | 0.00 | 0.03 | 0.06 | 0.00 | 0.00 | 0.00 | 0.00 | *Nitrospirae* | *Nitrospira* | *Nitrospirales* | *Nitrospiraceae* | Transient-rare taxa |
| OTU 675 | 0.00 | 0.00 | 0.00 | 0.00 | 0.09 | 0.00 | 0.00 | 0.00 | 0.00 | 0.00 | 0.00 | 0.00 | *Firmicutes* | *Clostridia* | *Clostridiales* | *Lachnospiraceae* | Transient-rare taxa |
| OTU 678 | 0.01 | 0.00 | 0.00 | 0.00 | 0.00 | 0.00 | 0.00 | 0.00 | 0.07 | 0.00 | 0.01 | 0.00 | *Proteobacteria* | *Gammaproteobacteria* | *Xanthomonadales* | *Xanthomonadaceae* | Transient-rare taxa |
| OTU 681 | 0.00 | 0.07 | 0.01 | 0.00 | 0.00 | 0.00 | 0.00 | 0.01 | 0.00 | 0.00 | 0.00 | 0.00 | *Proteobacteria* | *Gammaproteobacteria* | *Chromatiales* | *Ectothiorhodospiraceae* | Transient-rare taxa |
| OTU 683 | 0.00 | 0.00 | 0.00 | 0.00 | 0.00 | 0.04 | 0.00 | 0.00 | 0.00 | 0.02 | 0.02 | 0.00 | *Bacteroidetes* | *Sphingobacteriia* | *Sphingobacteriales* | *Saprospiraceae* | Transient-rare taxa |
| OTU 685 | 0.00 | 0.00 | 0.00 | 0.07 | 0.00 | 0.00 | 0.00 | 0.00 | 0.00 | 0.00 | 0.02 | 0.00 | *Proteobacteria* | *Gammaproteobacteria* | *Pseudomonadales* | *Moraxellaceae* | Transient-rare taxa |
| OTU 686 | 0.00 | 0.08 | 0.01 | 0.00 | 0.00 | 0.00 | 0.00 | 0.00 | 0.00 | 0.00 | 0.00 | 0.00 | *Proteobacteria* | *Gammaproteobacteria* | *Chromatiales* | *Ectothiorhodospiraceae* | Transient-rare taxa |
| OTU 687 | 0.00 | 0.00 | 0.00 | 0.00 | 0.08 | 0.01 | 0.00 | 0.00 | 0.00 | 0.00 | 0.00 | 0.00 | *Proteobacteria* | *Alphaproteobacteria* | *Rhodobacterales* | *Rhodobacteraceae* | Transient-rare taxa |
| OTU 689 | 0.00 | 0.00 | 0.00 | 0.00 | 0.07 | 0.00 | 0.00 | 0.00 | 0.00 | 0.02 | 0.00 | 0.00 | *Bacteroidetes* | *Bacteroidia* | *Bacteroidales* | *Prevotellaceae* | Transient-rare taxa |
| OTU 692 | 0.00 | 0.00 | 0.00 | 0.00 | 0.00 | 0.00 | 0.00 | 0.00 | 0.00 | 0.00 | 0.09 | 0.00 | *Proteobacteria* | *Deltaproteobacteria* | *Myxococcales* | *Nannocystineae* | Transient-rare taxa |
| OTU 694 | 0.00 | 0.00 | 0.00 | 0.00 | 0.08 | 0.00 | 0.00 | 0.00 | 0.00 | 0.00 | 0.01 | 0.00 | *Proteobacteria* | *Gammaproteobacteria* | *Pseudomonadales* | *Pseudomonadaceae* | Transient-rare taxa |
| OTU 696 | 0.00 | 0.00 | 0.00 | 0.00 | 0.00 | 0.00 | 0.00 | 0.04 | 0.04 | 0.00 | 0.00 | 0.00 | *Proteobacteria* | *Deltaproteobacteria* | *Myxococcales* | *Nannocystineae* | Transient-rare taxa |
| OTU 698 | 0.00 | 0.00 | 0.00 | 0.02 | 0.00 | 0.00 | 0.00 | 0.00 | 0.04 | 0.02 | 0.00 | 0.00 | *Bacteroidetes* | *Flavobacteriia* | *Flavobacteriales* | *Cryomorphaceae* | Transient-rare taxa |

| | | | | | | | | | | | | | | | | | |
|---|---|---|---|---|---|---|---|---|---|---|---|---|---|---|---|---|---|
| OTU 699 | 0.00 | 0.00 | 0.00 | 0.00 | 0.09 | 0.00 | 0.00 | 0.00 | 0.00 | 0.00 | 0.00 | 0.00 | Proteobacteria | Deltaproteobacteria | Myxococcales | Sorangiineae | Transient-rare taxa |
| OTU 700 | 0.02 | 0.07 | 0.00 | 0.00 | 0.00 | 0.00 | 0.00 | 0.00 | 0.00 | 0.00 | 0.00 | 0.00 | Proteobacteria | Gammaproteobacteria | Chromatiales | Chromatiaceae | Transient-rare taxa |
| OTU 701 | 0.00 | 0.01 | 0.00 | 0.08 | 0.00 | 0.00 | 0.00 | 0.00 | 0.00 | 0.00 | 0.00 | 0.00 | Bacteroidetes | Bacteroidetes_incertae_sedis | Ohtaekwangia | | Transient-rare taxa |
| OTU 705 | 0.00 | 0.00 | 0.00 | 0.00 | 0.07 | 0.02 | 0.00 | 0.00 | 0.00 | 0.00 | 0.00 | 0.00 | Proteobacteria | Alphaproteobacteria | Rhodobacterales | Rhodobacteraceae | Transient-rare taxa |
| OTU 710 | 0.00 | 0.00 | 0.00 | 0.08 | 0.01 | 0.00 | 0.00 | 0.00 | 0.00 | 0.00 | 0.00 | 0.00 | Proteobacteria | Deltaproteobacteria | Desulfobacterales | Desulfobulbaceae | Transient-rare taxa |
| OTU 711 | 0.00 | 0.00 | 0.01 | 0.00 | 0.00 | 0.07 | 0.00 | 0.00 | 0.01 | 0.00 | 0.00 | 0.00 | Proteobacteria | Gammaproteobacteria | Gammaproteobacteria_incertae_sedis | Thiohalobacter | Transient-rare taxa |
| OTU 712 | 0.08 | 0.00 | 0.00 | 0.00 | 0.00 | 0.01 | 0.00 | 0.00 | 0.00 | 0.00 | 0.00 | 0.00 | Actinobacteria | Actinobacteria | Actinobacteridae | Actinomycetales | Transient-rare taxa |
| OTU 713 | 0.00 | 0.00 | 0.00 | 0.00 | 0.03 | 0.06 | 0.00 | 0.00 | 0.00 | 0.00 | 0.00 | 0.00 | Proteobacteria | Alphaproteobacteria | Sphingomonadales | Erythrobacteraceae | Transient-rare taxa |
| OTU 714 | 0.00 | 0.00 | 0.00 | 0.09 | 0.00 | 0.00 | 0.00 | 0.00 | 0.00 | 0.00 | 0.00 | 0.00 | Bacteroidetes | Flavobacteriia | Flavobacteriales | Flavobacteriaceae | Transient-rare taxa |
| OTU 715 | 0.02 | 0.00 | 0.00 | 0.00 | 0.00 | 0.01 | 0.06 | 0.00 | 0.00 | 0.00 | 0.00 | 0.00 | Proteobacteria | Betaproteobacteria | Rhodocyclales | Rhodocyclaceae | Transient-rare taxa |
| OTU 716 | 0.08 | 0.00 | 0.00 | 0.00 | 0.00 | 0.00 | 0.01 | 0.00 | 0.00 | 0.00 | 0.00 | 0.00 | Proteobacteria | Betaproteobacteria | Burkholderiales | Comamonadaceae | Transient-rare taxa |
| OTU 719 | 0.02 | 0.00 | 0.07 | 0.00 | 0.00 | 0.00 | 0.00 | 0.00 | 0.00 | 0.00 | 0.00 | 0.00 | Chloroflexi | Ardenticatenia | Ardenticatenales | Ardenticatenaceae | Transient-rare taxa |
| OTU 721 | 0.00 | 0.00 | 0.09 | 0.00 | 0.00 | 0.00 | 0.00 | 0.00 | 0.00 | 0.00 | 0.00 | 0.00 | Proteobacteria | Betaproteobacteria | Burkholderiales | Comamonadaceae | Transient-rare taxa |
| OTU 722 | 0.02 | 0.00 | 0.00 | 0.00 | 0.00 | 0.00 | 0.00 | 0.00 | 0.00 | 0.00 | 0.00 | 0.07 | Planctomycetes | Planctomycetia | Planctomycetales | Planctomycetaceae | Transient-rare taxa |
| OTU 723 | 0.00 | 0.00 | 0.00 | 0.00 | 0.02 | 0.00 | 0.00 | 0.00 | 0.00 | 0.00 | 0.01 | 0.06 | Bacteroidetes | Flavobacteriia | Flavobacteriales | Flavobacteriaceae | Transient-rare taxa |
| OTU 726 | 0.00 | 0.00 | 0.00 | 0.00 | 0.00 | 0.00 | 0.00 | 0.01 | 0.00 | 0.08 | 0.00 | 0.00 | Proteobacteria | Betaproteobacteria | Nitrosomonadales | Nitrosomonadaceae | Transient-rare taxa |
| OTU 730 | 0.00 | 0.00 | 0.00 | 0.00 | 0.00 | 0.00 | 0.00 | 0.00 | 0.00 | 0.09 | 0.00 | 0.00 | Firmicutes | Clostridia | Clostridiales | Clostridiales_Incertae Sedis XII | Transient-rare taxa |
| OTU 732 | 0.00 | 0.00 | 0.09 | 0.00 | 0.00 | 0.00 | 0.00 | 0.00 | 0.00 | 0.00 | 0.00 | 0.00 | Proteobacteria | Gammaproteobacteria | Thiotrichales | Francisellaceae | Transient-rare taxa |
| OTU 733 | 0.00 | 0.00 | 0.09 | 0.00 | 0.00 | 0.00 | 0.00 | 0.00 | 0.00 | 0.00 | 0.00 | 0.00 | Candidatus Saccharibacteria | Saccharibacteria_genera_incertae_sedis | | | Transient-rare taxa |
| OTU 735 | 0.00 | 0.00 | 0.03 | 0.00 | 0.00 | 0.00 | 0.00 | 0.03 | 0.02 | 0.00 | 0.00 | 0.00 | Proteobacteria | Betaproteobacteria | Rhodocyclales | Rhodocyclaceae | Transient-rare taxa |
| OTU 736 | 0.00 | 0.00 | 0.00 | 0.08 | 0.01 | 0.00 | 0.00 | 0.00 | 0.00 | 0.00 | 0.00 | 0.00 | Proteobacteria | Gammaproteobacteria | Alteromonadales | Psychromonadaceae | Transient-rare taxa |
| OTU 738 | 0.00 | 0.00 | 0.00 | 0.00 | 0.02 | 0.02 | 0.00 | 0.00 | 0.00 | 0.00 | 0.00 | 0.04 | Proteobacteria | Betaproteobacteria | Burkholderiales | Comamonadaceae | Transient-rare taxa |
| OTU 739 | 0.00 | 0.00 | 0.03 | 0.06 | 0.00 | 0.00 | 0.00 | 0.00 | 0.00 | 0.00 | 0.00 | 0.00 | Bacteroidetes | Flavobacteriia | Flavobacteriales | Flavobacteriaceae | Transient-rare taxa |
| OTU 740 | 0.00 | 0.00 | 0.00 | 0.00 | 0.00 | 0.00 | 0.00 | 0.02 | 0.02 | 0.03 | 0.00 | 0.00 | Chloroflexi | Anaerolineae | Anaerolineales | Anaerolineaceae | Transient-rare taxa |
| OTU 741 | 0.00 | 0.04 | 0.00 | 0.00 | 0.00 | 0.00 | 0.00 | 0.00 | 0.03 | 0.00 | 0.00 | 0.00 | Proteobacteria | Gammaproteobacteria | Xanthomonadales | Xanthomonadaceae | Transient-rare taxa |
| OTU 742 | 0.00 | 0.00 | 0.06 | 0.00 | 0.00 | 0.00 | 0.00 | 0.02 | 0.00 | 0.00 | 0.00 | 0.00 | Bacteroidetes | Flavobacteriia | Flavobacteriales | Flavobacteriaceae | Transient-rare taxa |
| OTU 743 | 0.00 | 0.00 | 0.02 | 0.03 | 0.00 | 0.00 | 0.00 | 0.00 | 0.00 | 0.02 | 0.00 | 0.00 | Proteobacteria | Deltaproteobacteria | Desulfuromonadales | Geobacteraceae | Transient-rare taxa |
| OTU 744 | 0.00 | 0.00 | 0.00 | 0.00 | 0.00 | 0.00 | 0.08 | 0.00 | 0.00 | 0.00 | 0.00 | 0.00 | Planctomycetes | Planctomycetia | Planctomycetales | Planctomycetaceae | Transient-rare taxa |
| OTU 745 | 0.00 | 0.00 | 0.00 | 0.00 | 0.00 | 0.01 | 0.07 | 0.00 | 0.00 | 0.00 | 0.00 | 0.00 | Proteobacteria | Betaproteobacteria | Nitrosomonadales | Nitrosomonadaceae | Transient-rare taxa |
| OTU 746 | 0.00 | 0.00 | 0.04 | 0.02 | 0.00 | 0.00 | 0.01 | 0.00 | 0.00 | 0.00 | 0.00 | 0.00 | Bacteroidetes | Flavobacteriia | Flavobacteriales | Flavobacteriaceae | Transient-rare taxa |
| OTU 747 | 0.00 | 0.06 | 0.00 | 0.00 | 0.00 | 0.00 | 0.02 | 0.00 | 0.00 | 0.00 | 0.00 | 0.00 | Proteobacteria | Betaproteobacteria | Rhodocyclales | Rhodocyclaceae | Transient-rare taxa |
| OTU 750 | 0.01 | 0.04 | 0.00 | 0.00 | 0.00 | 0.00 | 0.00 | 0.02 | 0.00 | 0.00 | 0.00 | 0.00 | Proteobacteria | Betaproteobacteria | Rhodocyclales | Rhodocyclaceae | Transient-rare taxa |
| OTU 751 | 0.00 | 0.01 | 0.03 | 0.00 | 0.00 | 0.00 | 0.00 | 0.00 | 0.03 | 0.00 | 0.00 | 0.00 | Proteobacteria | Deltaproteobacteria | Myxococcales | Sorangiineae | Transient-rare taxa |
| OTU 752 | 0.00 | 0.00 | 0.01 | 0.04 | 0.00 | 0.00 | 0.02 | 0.00 | 0.00 | 0.00 | 0.00 | 0.00 | Bacteroidetes | Sphingobacteriia | Sphingobacteriales | Saprospiraceae | Transient-rare taxa |
| OTU 754 | 0.00 | 0.00 | 0.00 | 0.00 | 0.02 | 0.00 | 0.06 | 0.00 | 0.00 | 0.00 | 0.00 | 0.00 | Bacteroidetes | Flavobacteriia | Flavobacteriales | Flavobacteriaceae | Transient-rare taxa |
| OTU 755 | 0.00 | 0.00 | 0.00 | 0.00 | 0.01 | 0.01 | 0.00 | 0.06 | 0.00 | 0.00 | 0.00 | 0.00 | Firmicutes | Clostridia | Clostridiales | Ruminococcaceae | Transient-rare taxa |
| OTU 756 | 0.00 | 0.04 | 0.00 | 0.00 | 0.00 | 0.00 | 0.00 | 0.01 | 0.02 | 0.00 | 0.00 | 0.00 | Proteobacteria | Deltaproteobacteria | Myxococcales | Sorangiineae | Transient-rare taxa |
| OTU 759 | 0.00 | 0.00 | 0.01 | 0.00 | 0.00 | 0.01 | 0.06 | 0.00 | 0.00 | 0.00 | 0.00 | 0.00 | Proteobacteria | Alphaproteobacteria | Alphaproteobacteria_incertae_sedis | Geminicoccus | Transient-rare taxa |
| OTU 761 | 0.00 | 0.01 | 0.00 | 0.00 | 0.00 | 0.00 | 0.07 | 0.00 | 0.00 | 0.00 | 0.00 | 0.00 | Proteobacteria | Deltaproteobacteria | Myxococcales | Nannocystineae | Transient-rare taxa |
| OTU 762 | 0.00 | 0.01 | 0.00 | 0.00 | 0.00 | 0.00 | 0.00 | 0.00 | 0.04 | 0.00 | 0.02 | 0.00 | Proteobacteria | Deltaproteobacteria | Myxococcales | Nannocystineae | Transient-rare taxa |
| OTU 763 | 0.00 | 0.07 | 0.00 | 0.00 | 0.00 | 0.00 | 0.00 | 0.00 | 0.01 | 0.00 | 0.00 | 0.00 | Proteobacteria | Deltaproteobacteria | Myxococcales | Sorangiineae | Transient-rare taxa |
| OTU 766 | 0.00 | 0.00 | 0.00 | 0.00 | 0.00 | 0.00 | 0.00 | 0.00 | 0.00 | 0.01 | 0.07 | 0.00 | Bacteroidetes | Sphingobacteriia | Sphingobacteriales | Chitinophagaceae | Transient-rare taxa |
| OTU 769 | 0.06 | 0.02 | 0.00 | 0.00 | 0.00 | 0.00 | 0.00 | 0.00 | 0.00 | 0.00 | 0.00 | 0.00 | Proteobacteria | Gammaproteobacteria | Chromatiales | Chromatiaceae | Transient-rare taxa |
| OTU 770 | 0.00 | 0.00 | 0.00 | 0.00 | 0.04 | 0.02 | 0.01 | 0.00 | 0.00 | 0.00 | 0.00 | 0.00 | Proteobacteria | Betaproteobacteria | Burkholderiales | Comamonadaceae | Transient-rare taxa |
| OTU 775 | 0.00 | 0.00 | 0.00 | 0.00 | 0.02 | 0.06 | 0.00 | 0.00 | 0.00 | 0.00 | 0.00 | 0.00 | Proteobacteria | Gammaproteobacteria | Oceanospirillales | Oceanospirillaceae | Transient-rare taxa |
| OTU 776 | 0.00 | 0.00 | 0.00 | 0.00 | 0.00 | 0.00 | 0.00 | 0.01 | 0.06 | 0.01 | 0.00 | 0.00 | Acidobacteria | Acidobacteria_Gp10 | Gp10 | | Transient-rare taxa |
| OTU 777 | 0.03 | 0.00 | 0.00 | 0.00 | 0.00 | 0.00 | 0.00 | 0.00 | 0.03 | 0.01 | 0.00 | 0.00 | Proteobacteria | Gammaproteobacteria | Xanthomonadales | Xanthomonadaceae | Transient-rare taxa |
| OTU 778 | 0.00 | 0.01 | 0.00 | 0.00 | 0.00 | 0.00 | 0.00 | 0.07 | 0.00 | 0.00 | 0.00 | 0.00 | Proteobacteria | Deltaproteobacteria | Myxococcales | Nannocystineae | Transient-rare taxa |
| OTU 781 | 0.00 | 0.00 | 0.01 | 0.07 | 0.00 | 0.00 | 0.00 | 0.00 | 0.00 | 0.00 | 0.00 | 0.00 | Bacteroidetes | Flavobacteriia | Flavobacteriales | Cryomorphaceae | Transient-rare taxa |
| OTU 782 | 0.02 | 0.00 | 0.00 | 0.04 | 0.00 | 0.00 | 0.00 | 0.00 | 0.00 | 0.00 | 0.00 | 0.01 | Bacteroidetes | Flavobacteriia | Flavobacteriales | Flavobacteriaceae | Transient-rare taxa |
| OTU 783 | 0.00 | 0.00 | 0.00 | 0.08 | 0.00 | 0.00 | 0.00 | 0.00 | 0.00 | 0.00 | 0.00 | 0.00 | Proteobacteria | Deltaproteobacteria | Desulfuromonadales | Desulfuromonadaceae | Transient-rare taxa |
| OTU 785 | 0.00 | 0.00 | 0.00 | 0.02 | 0.04 | 0.01 | 0.00 | 0.00 | 0.00 | 0.00 | 0.00 | 0.00 | Bacteroidetes | Flavobacteriia | Flavobacteriales | Flavobacteriaceae | Transient-rare taxa |
| OTU 790 | 0.00 | 0.00 | 0.04 | 0.00 | 0.02 | 0.00 | 0.00 | 0.00 | 0.00 | 0.00 | 0.00 | 0.01 | Proteobacteria | Betaproteobacteria | Burkholderiales | Burkholderiales_incertae_sedis | Transient-rare taxa |
| OTU 793 | 0.00 | 0.00 | 0.00 | 0.00 | 0.07 | 0.01 | 0.00 | 0.00 | 0.00 | 0.00 | 0.00 | 0.00 | Bacteroidetes | Flavobacteriia | Flavobacteriales | Flavobacteriaceae | Transient-rare taxa |
| OTU 796 | 0.07 | 0.00 | 0.00 | 0.00 | 0.00 | 0.00 | 0.00 | 0.00 | 0.00 | 0.01 | 0.00 | 0.00 | Proteobacteria | Deltaproteobacteria | Myxococcales | Cystobacterineae | Transient-rare taxa |
| OTU 797 | 0.00 | 0.00 | 0.00 | 0.00 | 0.01 | 0.00 | 0.00 | 0.00 | 0.00 | 0.04 | 0.00 | 0.02 | Bacteroidetes | Cytophagia | Cytophagales | Cytophagaceae | Transient-rare taxa |
| OTU 798 | 0.00 | 0.00 | 0.00 | 0.00 | 0.00 | 0.01 | 0.00 | 0.06 | 0.01 | 0.00 | 0.00 | 0.00 | Bacteroidetes | Sphingobacteriia | Sphingobacteriales | Saprospiraceae | Transient-rare taxa |
| OTU 799 | 0.08 | 0.00 | 0.00 | 0.00 | 0.00 | 0.00 | 0.00 | 0.00 | 0.00 | 0.00 | 0.00 | 0.00 | Proteobacteria | Gammaproteobacteria | Chromatiales | Chromatiaceae | Transient-rare taxa |
| OTU 800 | 0.00 | 0.02 | 0.00 | 0.00 | 0.00 | 0.00 | 0.00 | 0.04 | 0.00 | 0.00 | 0.00 | 0.01 | Chloroflexi | Anaerolineae | Anaerolineales | Anaerolineaceae | Transient-rare taxa |
| OTU 805 | 0.01 | 0.00 | 0.00 | 0.00 | 0.00 | 0.00 | 0.00 | 0.00 | 0.00 | 0.00 | 0.00 | 0.07 | Proteobacteria | Deltaproteobacteria | Myxococcales | Sorangiineae | Transient-rare taxa |
| OTU 806 | 0.00 | 0.02 | 0.00 | 0.00 | 0.00 | 0.00 | 0.00 | 0.00 | 0.00 | 0.00 | 0.00 | 0.06 | Proteobacteria | Betaproteobacteria | Rhodocyclales | Rhodocyclaceae | Transient-rare taxa |
| OTU 807 | 0.00 | 0.00 | 0.00 | 0.00 | 0.00 | 0.00 | 0.00 | 0.00 | 0.00 | 0.00 | 0.00 | 0.08 | Bacteroidetes | Bacteroidetes_incertae_sedis | Ohtaekwangia | | Transient-rare taxa |
| OTU 808 | 0.00 | 0.00 | 0.00 | 0.00 | 0.01 | 0.00 | 0.00 | 0.00 | 0.00 | 0.07 | 0.00 | 0.00 | Firmicutes | Clostridia | Clostridiales | Clostridiales_Incertae Sedis XIII | Transient-rare taxa |
| OTU 810 | 0.00 | 0.00 | 0.00 | 0.00 | 0.00 | 0.00 | 0.00 | 0.00 | 0.02 | 0.06 | 0.00 | 0.00 | Proteobacteria | Betaproteobacteria | Rhodocyclales | Rhodocyclaceae | Transient-rare taxa |
| OTU 811 | 0.00 | 0.00 | 0.00 | 0.00 | 0.00 | 0.00 | 0.00 | 0.00 | 0.02 | 0.06 | 0.00 | 0.00 | Marinimicrobia | Marinimicrobia_genera_incertae_sedis | | | Transient-rare taxa |
| OTU 812 | 0.01 | 0.00 | 0.00 | 0.00 | 0.00 | 0.00 | 0.00 | 0.00 | 0.00 | 0.06 | 0.00 | 0.01 | Proteobacteria | Betaproteobacteria | Rhodocyclales | Rhodocyclaceae | Transient-rare taxa |
| OTU 814 | 0.01 | 0.00 | 0.00 | 0.00 | 0.00 | 0.00 | 0.00 | 0.00 | 0.00 | 0.00 | 0.00 | 0.07 | Proteobacteria | Betaproteobacteria | Rhodocyclales | Rhodocyclaceae | Transient-rare taxa |
| OTU 815 | 0.00 | 0.00 | 0.06 | 0.02 | 0.00 | 0.00 | 0.00 | 0.00 | 0.00 | 0.00 | 0.00 | 0.00 | Planctomycetes | Planctomycetia | Planctomycetales | Planctomycetaceae | Transient-rare taxa |
| OTU 816 | 0.00 | 0.00 | 0.00 | 0.00 | 0.00 | 0.00 | 0.01 | 0.06 | 0.00 | 0.00 | 0.01 | 0.00 | Planctomycetes | Planctomycetia | Planctomycetales | Planctomycetaceae | Transient-rare taxa |
| OTU 817 | 0.00 | 0.00 | 0.00 | 0.00 | 0.00 | 0.00 | 0.00 | 0.00 | 0.03 | 0.02 | 0.00 | 0.02 | Planctomycetes | Planctomycetia | Planctomycetales | Planctomycetaceae | Transient-rare taxa |
| OTU 819 | 0.00 | 0.00 | 0.00 | 0.00 | 0.00 | 0.00 | 0.00 | 0.00 | 0.00 | 0.00 | 0.08 | 0.00 | Proteobacteria | Betaproteobacteria | Rhodocyclales | Rhodocyclaceae | Transient-rare taxa |
| OTU 820 | 0.00 | 0.00 | 0.00 | 0.00 | 0.00 | 0.00 | 0.00 | 0.00 | 0.00 | 0.00 | 0.08 | 0.00 | Proteobacteria | Deltaproteobacteria | Myxococcales | Nannocystineae | Transient-rare taxa |
| OTU 821 | 0.00 | 0.00 | 0.00 | 0.00 | 0.00 | 0.00 | 0.00 | 0.00 | 0.00 | 0.00 | 0.08 | 0.00 | Proteobacteria | Deltaproteobacteria | Myxococcales | Nannocystineae | Transient-rare taxa |
| OTU 822 | 0.00 | 0.00 | 0.00 | 0.00 | 0.07 | 0.00 | 0.00 | 0.00 | 0.00 | 0.00 | 0.01 | 0.00 | Proteobacteria | Betaproteobacteria | Burkholderiales | Comamonadaceae | Transient-rare taxa |
| OTU 823 | 0.01 | 0.00 | 0.00 | 0.00 | 0.00 | 0.00 | 0.00 | 0.00 | 0.00 | 0.00 | 0.07 | 0.00 | Proteobacteria | Deltaproteobacteria | Myxococcales | Nannocystineae | Transient-rare taxa |
| OTU 824 | 0.00 | 0.00 | 0.00 | 0.00 | 0.00 | 0.01 | 0.00 | 0.00 | 0.00 | 0.00 | 0.02 | 0.04 | Bacteroidetes | Flavobacteriia | Flavobacteriales | Flavobacteriaceae | Transient-rare taxa |
| OTU 827 | 0.00 | 0.00 | 0.00 | 0.00 | 0.02 | 0.06 | 0.00 | 0.00 | 0.00 | 0.00 | 0.00 | 0.00 | Proteobacteria | Alphaproteobacteria | Rhizobiales | Phyllobacteriaceae | Transient-rare taxa |
| OTU 829 | 0.01 | 0.00 | 0.00 | 0.00 | 0.00 | 0.00 | 0.00 | 0.00 | 0.02 | 0.00 | 0.00 | 0.03 | Proteobacteria | Alphaproteobacteria | Rhodobacterales | Rhodobacteraceae | Transient-rare taxa |
| OTU 830 | 0.00 | 0.01 | 0.06 | 0.00 | 0.00 | 0.00 | 0.00 | 0.00 | 0.00 | 0.00 | 0.00 | 0.00 | Proteobacteria | Deltaproteobacteria | Bdellovibrionales | Bacteriovoracaceae | Transient-rare taxa |
| OTU 831 | 0.00 | 0.01 | 0.06 | 0.00 | 0.00 | 0.00 | 0.00 | 0.00 | 0.00 | 0.00 | 0.00 | 0.00 | Proteobacteria | Betaproteobacteria | Hydrogenophilales | Hydrogenophilaceae | Transient-rare taxa |
| OTU 832 | 0.00 | 0.00 | 0.07 | 0.00 | 0.00 | 0.00 | 0.00 | 0.00 | 0.00 | 0.00 | 0.00 | 0.00 | Proteobacteria | Gammaproteobacteria | Chromatiales | Ectothiorhodospiraceae | Transient-rare taxa |
| OTU 836 | 0.00 | 0.00 | 0.00 | 0.00 | 0.06 | 0.00 | 0.01 | 0.00 | 0.00 | 0.00 | 0.00 | 0.00 | Bacteroidetes | Flavobacteriia | Flavobacteriales | Flavobacteriaceae | Transient-rare taxa |
| OTU 837 | 0.00 | 0.00 | 0.00 | 0.00 | 0.00 | 0.00 | 0.00 | 0.01 | 0.06 | 0.00 | 0.00 | 0.00 | Proteobacteria | Deltaproteobacteria | Myxococcales | Nannocystineae | Transient-rare taxa |
| OTU 838 | 0.00 | 0.00 | 0.00 | 0.00 | 0.00 | 0.00 | 0.00 | 0.01 | 0.04 | 0.00 | 0.00 | 0.01 | Proteobacteria | Betaproteobacteria | Nitrosomonadales | Nitrosomonadaceae | Transient-rare taxa |
| OTU 839 | 0.00 | 0.01 | 0.00 | 0.00 | 0.00 | 0.00 | 0.00 | 0.01 | 0.04 | 0.00 | 0.00 | 0.00 | Planctomycetes | Planctomycetia | Planctomycetales | Planctomycetaceae | Transient-rare taxa |
| OTU 840 | 0.00 | 0.07 | 0.00 | 0.00 | 0.00 | 0.00 | 0.00 | 0.00 | 0.00 | 0.00 | 0.00 | 0.00 | Planctomycetes | Planctomycetia | Planctomycetales | Planctomycetaceae | Transient-rare taxa |
| OTU 841 | 0.00 | 0.06 | 0.00 | 0.00 | 0.00 | 0.00 | 0.00 | 0.00 | 0.00 | 0.01 | 0.00 | 0.00 | Planctomycetes | Planctomycetia | Planctomycetales | Planctomycetaceae | Transient-rare taxa |
| OTU 843 | 0.00 | 0.00 | 0.00 | 0.00 | 0.00 | 0.00 | 0.00 | 0.00 | 0.06 | 0.01 | 0.00 | 0.00 | Planctomycetes | Planctomycetia | Planctomycetales | Planctomycetaceae | Transient-rare taxa |
| OTU 844 | 0.01 | 0.00 | 0.00 | 0.00 | 0.00 | 0.00 | 0.00 | 0.00 | 0.01 | 0.00 | 0.00 | 0.04 | Proteobacteria | Betaproteobacteria | Rhodocyclales | Rhodocyclaceae | Transient-rare taxa |
| OTU 847 | 0.00 | 0.07 | 0.00 | 0.00 | 0.00 | 0.00 | 0.00 | 0.00 | 0.00 | 0.00 | 0.00 | 0.00 | Proteobacteria | Betaproteobacteria | Nitrosomonadales | Nitrosomonadaceae | Transient-rare taxa |
| OTU 848 | 0.00 | 0.00 | 0.00 | 0.00 | 0.00 | 0.00 | 0.07 | 0.00 | 0.00 | 0.00 | 0.00 | 0.00 | Bacteroidetes | Sphingobacteriia | Sphingobacteriales | Saprospiraceae | Transient-rare taxa |
| OTU 850 | 0.00 | 0.00 | 0.00 | 0.00 | 0.00 | 0.00 | 0.00 | 0.00 | 0.04 | 0.01 | 0.00 | 0.01 | Verrucomicrobia | Subdivision3 | Subdivision3_genera_incertae_sedis | | Transient-rare taxa |
| OTU 851 | 0.00 | 0.00 | 0.00 | 0.00 | 0.00 | 0.00 | 0.00 | 0.02 | 0.04 | 0.00 | 0.00 | 0.00 | Proteobacteria | Deltaproteobacteria | Myxococcales | Cystobacterineae | Transient-rare taxa |
| OTU 852 | 0.00 | 0.00 | 0.01 | 0.00 | 0.00 | 0.01 | 0.00 | 0.00 | 0.00 | 0.00 | 0.04 | 0.00 | Thermotogae | Thermotogae | Thermotogales | Thermotogaceae | Transient-rare taxa |
| OTU 853 | 0.01 | 0.00 | 0.00 | 0.00 | 0.04 | 0.00 | 0.00 | 0.01 | 0.00 | 0.00 | 0.00 | 0.00 | Proteobacteria | Gammaproteobacteria | Xanthomonadales | Xanthomonadaceae | Transient-rare taxa |
| OTU 855 | 0.00 | 0.03 | 0.02 | 0.00 | 0.00 | 0.00 | 0.00 | 0.00 | 0.00 | 0.00 | 0.01 | 0.00 | Proteobacteria | Betaproteobacteria | Rhodocyclales | Rhodocyclaceae | Transient-rare taxa |
| OTU 857 | 0.00 | 0.01 | 0.00 | 0.00 | 0.00 | 0.00 | 0.00 | 0.00 | 0.06 | 0.00 | 0.00 | 0.00 | Proteobacteria | Gammaproteobacteria | Xanthomonadales | Xanthomonadaceae | Transient-rare taxa |
| OTU 858 | 0.00 | 0.06 | 0.01 | 0.00 | 0.00 | 0.00 | 0.00 | 0.00 | 0.00 | 0.00 | 0.00 | 0.00 | Planctomycetes | Planctomycetia | Candidatus Brocadiales | Candidatus Brocadiaceae | Transient-rare taxa |
| OTU 859 | 0.00 | 0.00 | 0.00 | 0.00 | 0.00 | 0.00 | 0.00 | 0.01 | 0.02 | 0.03 | 0.00 | 0.00 | Acidobacteria | Acidobacteria_Gp4 | Gp4 | | Transient-rare taxa |
| OTU 861 | 0.00 | 0.00 | 0.04 | 0.00 | 0.02 | 0.00 | 0.00 | 0.00 | 0.00 | 0.00 | 0.00 | 0.00 | Proteobacteria | Deltaproteobacteria | Myxococcales | Nannocystineae | Transient-rare taxa |
| OTU 863 | 0.00 | 0.00 | 0.00 | 0.00 | 0.01 | 0.06 | 0.00 | 0.00 | 0.00 | 0.00 | 0.00 | 0.00 | Actinobacteria | Actinobacteria | Actinobacteridae | Actinomycetales | Transient-rare taxa |
| OTU 865 | 0.00 | 0.00 | 0.00 | 0.00 | 0.06 | 0.01 | 0.00 | 0.00 | 0.00 | 0.00 | 0.00 | 0.00 | Planctomycetes | Planctomycetia | Candidatus Brocadiales | Candidatus Brocadiaceae | Transient-rare taxa |
| OTU 866 | 0.00 | 0.00 | 0.00 | 0.00 | 0.02 | 0.04 | 0.00 | 0.00 | 0.00 | 0.00 | 0.00 | 0.00 | Planctomycetes | Planctomycetia | Planctomycetales | Planctomycetaceae | Transient-rare taxa |
| OTU 869 | 0.00 | 0.00 | 0.00 | 0.00 | 0.00 | 0.00 | 0.04 | 0.00 | 0.01 | 0.01 | 0.00 | 0.00 | Proteobacteria | Alphaproteobacteria | Rhodospirillales | Rhodospirillaceae | Transient-rare taxa |
| OTU 870 | 0.00 | 0.04 | 0.00 | 0.00 | 0.00 | 0.00 | 0.00 | 0.00 | 0.01 | 0.00 | 0.00 | 0.01 | Chloroflexi | Caldilineae | Caldilineales | Caldilineaceae | Transient-rare taxa |
| OTU 871 | 0.00 | 0.06 | 0.00 | 0.00 | 0.00 | 0.00 | 0.00 | 0.00 | 0.00 | 0.01 | 0.00 | 0.00 | Candidatus Saccharibacteria | Saccharibacteria_genera_incertae_sedis | | | Transient-rare taxa |
| OTU 872 | 0.00 | 0.04 | 0.02 | 0.00 | 0.00 | 0.00 | 0.00 | 0.00 | 0.00 | 0.00 | 0.00 | 0.00 | Proteobacteria | Betaproteobacteria | Rhodocyclales | Rhodocyclaceae | Transient-rare taxa |
| OTU 873 | 0.00 | 0.00 | 0.00 | 0.07 | 0.00 | 0.00 | 0.00 | 0.00 | 0.00 | 0.00 | 0.00 | 0.00 | Proteobacteria | Epsilonproteobacteria | Campylobacterales | Campylobacteraceae | Transient-rare taxa |
| OTU 874 | 0.03 | 0.00 | 0.00 | 0.00 | 0.02 | 0.01 | 0.00 | 0.00 | 0.00 | 0.00 | 0.00 | 0.00 | Ignavibacteriae | Ignavibacteria | Ignavibacteriales | Ignavibacteriaceae | Transient-rare taxa |
| OTU 876 | 0.00 | 0.02 | 0.03 | 0.00 | 0.00 | 0.00 | 0.00 | 0.00 | 0.00 | 0.00 | 0.01 | 0.00 | Proteobacteria | Alphaproteobacteria | Rhodobacterales | Rhodobacteraceae | Transient-rare taxa |
| OTU 877 | 0.00 | 0.00 | 0.00 | 0.00 | 0.00 | 0.00 | 0.02 | 0.03 | 0.00 | 0.00 | 0.00 | 0.01 | Nitrospirae | Nitrospira | Nitrospirales | Nitrospiraceae | Transient-rare taxa |
| OTU 879 | 0.00 | 0.00 | 0.00 | 0.00 | 0.03 | 0.00 | 0.00 | 0.00 | 0.01 | 0.02 | 0.00 | 0.00 | Proteobacteria | Gammaproteobacteria | Pseudomonadales | Pseudomonadaceae | Transient-rare taxa |
| OTU 880 | 0.00 | 0.06 | 0.00 | 0.00 | 0.00 | 0.00 | 0.00 | 0.00 | 0.01 | 0.00 | 0.00 | 0.00 | Planctomycetes | Planctomycetia | Planctomycetales | Planctomycetaceae | Transient-rare taxa |
| OTU 881 | 0.00 | 0.00 | 0.01 | 0.04 | 0.00 | 0.00 | 0.00 | 0.00 | 0.00 | 0.00 | 0.01 | 0.00 | Proteobacteria | Gammaproteobacteria | Pseudomonadales | Moraxellaceae | Transient-rare taxa |
| OTU 882 | 0.00 | 0.00 | 0.00 | 0.07 | 0.00 | 0.00 | 0.00 | 0.00 | 0.00 | 0.00 | 0.00 | 0.00 | Bacteroidetes | Flavobacteriia | Flavobacteriales | Cryomorphaceae | Transient-rare taxa |
| OTU 883 | 0.00 | 0.00 | 0.00 | 0.02 | 0.04 | 0.00 | 0.00 | 0.00 | 0.00 | 0.00 | 0.00 | 0.00 | Spirochaetes | Spirochaetia | Spirochaetales | Spirochaetaceae | Transient-rare taxa |
| OTU 886 | 0.01 | 0.03 | 0.00 | 0.00 | 0.00 | 0.00 | 0.00 | 0.02 | 0.00 | 0.00 | 0.00 | 0.00 | Proteobacteria | Gammaproteobacteria | Xanthomonadales | Xanthomonadaceae | Transient-rare taxa |
| OTU 887 | 0.00 | 0.00 | 0.00 | 0.04 | 0.02 | 0.00 | 0.00 | 0.00 | 0.00 | 0.00 | 0.00 | 0.00 | Proteobacteria | Alphaproteobacteria | Rhodobacterales | Rhodobacteraceae | Transient-rare taxa |
| OTU 888 | 0.01 | 0.03 | 0.00 | 0.00 | 0.00 | 0.00 | 0.00 | 0.00 | 0.00 | 0.00 | 0.00 | 0.02 | Proteobacteria | Betaproteobacteria | Hydrogenophilales | Hydrogenophilaceae | Transient-rare taxa |
| OTU 890 | 0.00 | 0.00 | 0.00 | 0.00 | 0.02 | 0.04 | 0.00 | 0.00 | 0.00 | 0.00 | 0.00 | 0.00 | Lentisphaerae | Lentisphaeria | Victivallales | Victivallaceae | Transient-rare taxa |
| OTU 891 | 0.00 | 0.00 | 0.00 | 0.00 | 0.00 | 0.01 | 0.06 | 0.00 | 0.00 | 0.00 | 0.00 | 0.00 | Proteobacteria | Gammaproteobacteria | Thiotrichales | Thiotrichaceae | Transient-rare taxa |
| OTU 892 | 0.00 | 0.00 | 0.00 | 0.00 | 0.06 | 0.01 | 0.00 | 0.00 | 0.00 | 0.00 | 0.00 | 0.00 | Proteobacteria | Deltaproteobacteria | Myxococcales | Nannocystineae | Transient-rare taxa |
| OTU 893 | 0.04 | 0.00 | 0.00 | 0.00 | 0.01 | 0.00 | 0.00 | 0.00 | 0.00 | 0.00 | 0.00 | 0.01 | Proteobacteria | Betaproteobacteria | Rhodocyclales | Rhodocyclaceae | Transient-rare taxa |
| OTU 895 | 0.00 | 0.00 | 0.00 | 0.03 | 0.01 | 0.02 | 0.00 | 0.00 | 0.00 | 0.00 | 0.00 | 0.00 | Bacteroidetes | Flavobacteriia | Flavobacteriales | Cryomorphaceae | Transient-rare taxa |
| OTU 896 | 0.00 | 0.00 | 0.00 | 0.00 | 0.03 | 0.00 | 0.00 | 0.00 | 0.00 | 0.00 | 0.02 | 0.01 | Bacteroidetes | Flavobacteriia | Flavobacteriales | Flavobacteriaceae | Transient-rare taxa |
| OTU 898 | 0.00 | 0.00 | 0.00 | 0.00 | 0.00 | 0.00 | 0.00 | 0.00 | 0.00 | 0.00 | 0.07 | 0.00 | Proteobacteria | Deltaproteobacteria | Myxococcales | Sorangiineae | Transient-rare taxa |
| OTU 900 | 0.00 | 0.00 | 0.00 | 0.00 | 0.02 | 0.00 | 0.00 | 0.00 | 0.00 | 0.00 | 0.04 | 0.00 | Proteobacteria | Gammaproteobacteria | Xanthomonadales | Xanthomonadaceae | Transient-rare taxa |
| OTU 901 | 0.07 | 0.00 | 0.00 | 0.00 | 0.00 | 0.00 | 0.00 | 0.00 | 0.00 | 0.00 | 0.00 | 0.00 | Proteobacteria | Deltaproteobacteria | Myxococcales | Sorangiineae | Transient-rare taxa |
| OTU 902 | 0.00 | 0.00 | 0.00 | 0.04 | 0.00 | 0.02 | 0.00 | 0.00 | 0.00 | 0.00 | 0.00 | 0.00 | Proteobacteria | Gammaproteobacteria | Pseudomonadales | Pseudomonadaceae | Transient-rare taxa |
| OTU 903 | 0.07 | 0.00 | 0.00 | 0.00 | 0.00 | 0.00 | 0.00 | 0.00 | 0.00 | 0.00 | 0.00 | 0.00 | Bacteroidetes | Sphingobacteriia | Sphingobacteriales | Saprospiraceae | Transient-rare taxa |
| OTU 904 | 0.00 | 0.03 | 0.02 | 0.00 | 0.00 | 0.00 | 0.00 | 0.01 | 0.00 | 0.00 | 0.00 | 0.00 | Proteobacteria | Gammaproteobacteria | Xanthomonadales | Xanthomonadaceae | Transient-rare taxa |
| OTU 905 | 0.00 | 0.00 | 0.00 | 0.00 | 0.00 | 0.00 | 0.00 | 0.07 | 0.00 | 0.00 | 0.00 | 0.00 | Proteobacteria | Deltaproteobacteria | Myxococcales | Nannocystineae | Transient-rare taxa |
| OTU 906 | 0.00 | 0.00 | 0.00 | 0.00 | 0.00 | 0.00 | 0.03 | 0.02 | 0.01 | 0.00 | 0.00 | 0.00 | Nitrospirae | Nitrospira | Nitrospirales | Nitrospiraceae | Transient-rare taxa |
| OTU 907 | 0.00 | 0.00 | 0.00 | 0.00 | 0.00 | 0.00 | 0.00 | 0.02 | 0.00 | 0.02 | 0.00 | 0.02 | Proteobacteria | Gammaproteobacteria | Oceanospirillales | Hahellaceae | Transient-rare taxa |
| OTU 908 | 0.01 | 0.00 | 0.00 | 0.00 | 0.00 | 0.00 | 0.00 | 0.00 | 0.01 | 0.00 | 0.04 | 0.00 | Proteobacteria | Gammaproteobacteria | Xanthomonadales | Xanthomonadaceae | Transient-rare taxa |
| OTU 909 | 0.00 | 0.00 | 0.00 | 0.00 | 0.00 | 0.00 | 0.00 | 0.00 | 0.00 | 0.03 | 0.03 | 0.00 | Proteobacteria | Deltaproteobacteria | Myxococcales | Sorangiineae | Transient-rare taxa |
| OTU 910 | 0.07 | 0.00 | 0.00 | 0.00 | 0.00 | 0.00 | 0.00 | 0.00 | 0.00 | 0.00 | 0.00 | 0.00 | Proteobacteria | Alphaproteobacteria | Sphingomonadales | Sphingomonadaceae | Transient-rare taxa |
| OTU 911 | 0.00 | 0.00 | 0.00 | 0.00 | 0.00 | 0.00 | 0.00 | 0.00 | 0.03 | 0.01 | 0.00 | 0.02 | Proteobacteria | Gammaproteobacteria | Pseudomonadales | Pseudomonadaceae | Transient-rare taxa |
| OTU 912 | 0.00 | 0.00 | 0.02 | 0.00 | 0.00 | 0.00 | 0.00 | 0.00 | 0.04 | 0.00 | 0.00 | 0.00 | Proteobacteria | Epsilonproteobacteria | Nautiliales | Nautiliaceae | Transient-rare taxa |
| OTU 913 | 0.00 | 0.00 | 0.00 | 0.00 | 0.00 | 0.00 | 0.00 | 0.00 | 0.00 | 0.00 | 0.00 | 0.07 | Proteobacteria | Alphaproteobacteria | Rhizobiales | Hyphomicrobiaceae | Transient-rare taxa |
| OTU 919 | 0.00 | 0.00 | 0.03 | 0.00 | 0.00 | 0.00 | 0.00 | 0.00 | 0.02 | 0.00 | 0.00 | 0.01 | Proteobacteria | Gammaproteobacteria | Pseudomonadales | Pseudomonadaceae | Transient-rare taxa |
| OTU 920 | 0.01 | 0.00 | 0.00 | 0.00 | 0.00 | 0.00 | 0.00 | 0.00 | 0.00 | 0.00 | 0.00 | 0.06 | Proteobacteria | Betaproteobacteria | Rhodocyclales | Rhodocyclaceae | Transient-rare taxa |

| | | | | | | | | | | | | | | | | | |
|---|---|---|---|---|---|---|---|---|---|---|---|---|---|---|---|---|---|
| OTU 921 | 0.00 | 0.00 | 0.00 | 0.00 | 0.00 | 0.00 | 0.00 | 0.01 | 0.03 | 0.02 | 0.00 | 0.00 | *Bacteroidetes* | *Sphingobacteriia* | *Sphingobacteriales* | *Chitinophagaceae* | Transient-rare taxa |
| OTU 923 | 0.04 | 0.00 | 0.00 | 0.00 | 0.00 | 0.01 | 0.00 | 0.00 | 0.00 | 0.00 | 0.01 | 0.00 | *Proteobacteria* | *Betaproteobacteria* | *Rhodocyclales* | *Rhodocyclaceae* | Transient-rare taxa |
| OTU 924 | 0.00 | 0.00 | 0.02 | 0.00 | 0.00 | 0.00 | 0.00 | 0.00 | 0.01 | 0.03 | 0.00 | 0.00 | *Firmicutes* | *Clostridia* | *Clostridiales* | *Clostridiales_Incertae Sedis XII* | Transient-rare taxa |
| OTU 926 | 0.00 | 0.00 | 0.00 | 0.00 | 0.00 | 0.00 | 0.00 | 0.00 | 0.00 | 0.03 | 0.03 | 0.00 | *Proteobacteria* | *Betaproteobacteria* | *Ferrovales* | *Ferrovaceae* | Transient-rare taxa |
| OTU 927 | 0.01 | 0.00 | 0.00 | 0.00 | 0.00 | 0.00 | 0.00 | 0.00 | 0.00 | 0.00 | 0.00 | 0.06 | *Proteobacteria* | *Betaproteobacteria* | *Rhodocyclales* | *Rhodocyclaceae* | Transient-rare taxa |
| OTU 928 | 0.04 | 0.00 | 0.00 | 0.00 | 0.00 | 0.00 | 0.00 | 0.00 | 0.00 | 0.00 | 0.00 | 0.02 | *Proteobacteria* | *Gammaproteobacteria* | *Xanthomonadales* | *Xanthomonadaceae* | Transient-rare taxa |
| OTU 930 | 0.00 | 0.00 | 0.00 | 0.00 | 0.00 | 0.00 | 0.00 | 0.00 | 0.00 | 0.00 | 0.06 | 0.01 | *Proteobacteria* | *Gammaproteobacteria* | *Pseudomonadales* | *Pseudomonadaceae* | Transient-rare taxa |
| OTU 931 | 0.00 | 0.00 | 0.00 | 0.00 | 0.00 | 0.00 | 0.00 | 0.00 | 0.00 | 0.07 | 0.00 | 0.00 | *Firmicutes* | *Clostridia* | *Clostridiales* | *Clostridiales_Incertae Sedis XII* | Transient-rare taxa |
| OTU 932 | 0.00 | 0.00 | 0.00 | 0.00 | 0.00 | 0.00 | 0.00 | 0.00 | 0.00 | 0.00 | 0.00 | 0.07 | *Proteobacteria* | *Alphaproteobacteria* | *Rhodobacterales* | *Rhodobacteraceae* | Transient-rare taxa |
| OTU 933 | 0.00 | 0.00 | 0.00 | 0.06 | 0.01 | 0.00 | 0.00 | 0.00 | 0.00 | 0.00 | 0.00 | 0.00 | *Proteobacteria* | *Gammaproteobacteria* | *Oceanospirillales* | *Oleiphilaceae* | Transient-rare taxa |
| OTU 934 | 0.00 | 0.00 | 0.00 | 0.00 | 0.00 | 0.00 | 0.00 | 0.00 | 0.00 | 0.03 | 0.00 | 0.03 | *Bacteroidetes* | *Sphingobacteriia* | *Sphingobacteriales* | *Sphingobacteriaceae* | Transient-rare taxa |
| OTU 935 | 0.00 | 0.00 | 0.00 | 0.00 | 0.00 | 0.00 | 0.00 | 0.01 | 0.00 | 0.00 | 0.00 | 0.06 | *Proteobacteria* | *Deltaproteobacteria* | *Myxococcales* | *Sorangiineae* | Transient-rare taxa |
| OTU 937 | 0.00 | 0.01 | 0.00 | 0.00 | 0.00 | 0.00 | 0.00 | 0.00 | 0.04 | 0.00 | 0.00 | 0.00 | *Bacteroidetes* | *Cytophagia* | *Cytophagales* | *Cytophagaceae* | Transient-rare taxa |
| OTU 938 | 0.00 | 0.00 | 0.00 | 0.00 | 0.00 | 0.00 | 0.04 | 0.00 | 0.01 | 0.00 | 0.00 | 0.00 | *candidate division WPS-1* | *WPS-1_genera_incertae_sedis* | | | Transient-rare taxa |
| OTU 939 | 0.00 | 0.00 | 0.00 | 0.00 | 0.00 | 0.00 | 0.03 | 0.01 | 0.01 | 0.00 | 0.00 | 0.00 | *Planctomycetes* | *Phycisphaerae* | *Phycisphaerales* | *Phycisphaeraceae* | Transient-rare taxa |
| OTU 940 | 0.00 | 0.00 | 0.02 | 0.00 | 0.00 | 0.00 | 0.03 | 0.00 | 0.00 | 0.00 | 0.00 | 0.00 | *Proteobacteria* | *Deltaproteobacteria* | *Myxococcales* | *Nannocystineae* | Transient-rare taxa |
| OTU 941 | 0.00 | 0.00 | 0.00 | 0.01 | 0.01 | 0.00 | 0.03 | 0.00 | 0.00 | 0.00 | 0.00 | 0.00 | *Bacteroidetes* | *Flavobacteriia* | *Flavobacteriales* | *Flavobacteriaceae* | Transient-rare taxa |
| OTU 942 | 0.00 | 0.00 | 0.00 | 0.00 | 0.01 | 0.00 | 0.00 | 0.00 | 0.04 | 0.00 | 0.00 | 0.00 | *Proteobacteria* | *Betaproteobacteria* | *Rhodocyclales* | *Rhodocyclaceae* | Transient-rare taxa |
| OTU 943 | 0.00 | 0.01 | 0.00 | 0.00 | 0.00 | 0.00 | 0.00 | 0.00 | 0.00 | 0.00 | 0.00 | 0.04 | *Proteobacteria* | *Betaproteobacteria* | *Burkholderiales* | *Burkholderiales_incertae_sedis* | Transient-rare taxa |
| OTU 946 | 0.01 | 0.04 | 0.00 | 0.00 | 0.00 | 0.00 | 0.00 | 0.00 | 0.00 | 0.00 | 0.00 | 0.00 | *Proteobacteria* | *Gammaproteobacteria* | *Chromatiales* | *Chromatiaceae* | Transient-rare taxa |
| OTU 949 | 0.03 | 0.02 | 0.00 | 0.00 | 0.00 | 0.00 | 0.00 | 0.00 | 0.00 | 0.00 | 0.00 | 0.00 | *Proteobacteria* | *Betaproteobacteria* | *Burkholderiales* | *Comamonadaceae* | Transient-rare taxa |
| OTU 950 | 0.00 | 0.06 | 0.00 | 0.00 | 0.00 | 0.00 | 0.00 | 0.00 | 0.00 | 0.00 | 0.00 | 0.00 | *Proteobacteria* | *Betaproteobacteria* | *Rhodocyclales* | *Rhodocyclaceae* | Transient-rare taxa |
| OTU 951 | 0.00 | 0.06 | 0.00 | 0.00 | 0.00 | 0.00 | 0.00 | 0.00 | 0.00 | 0.00 | 0.00 | 0.00 | *Proteobacteria* | *Gammaproteobacteria* | *Chromatiales* | *Ectothiorhodospiraceae* | Transient-rare taxa |
| OTU 952 | 0.02 | 0.00 | 0.00 | 0.00 | 0.01 | 0.00 | 0.02 | 0.00 | 0.00 | 0.00 | 0.00 | 0.00 | *Proteobacteria* | *Betaproteobacteria* | *Rhodocyclales* | *Rhodocyclaceae* | Transient-rare taxa |
| OTU 953 | 0.00 | 0.00 | 0.00 | 0.00 | 0.00 | 0.00 | 0.00 | 0.00 | 0.04 | 0.00 | 0.00 | 0.01 | *Proteobacteria* | *Deltaproteobacteria* | *Myxococcales* | *Nannocystineae* | Transient-rare taxa |
| OTU 955 | 0.00 | 0.00 | 0.00 | 0.00 | 0.00 | 0.00 | 0.00 | 0.00 | 0.00 | 0.00 | 0.06 | 0.00 | *Proteobacteria* | *Betaproteobacteria* | *Burkholderiales* | *Comamonadaceae* | Transient-rare taxa |
| OTU 956 | 0.00 | 0.00 | 0.00 | 0.00 | 0.00 | 0.00 | 0.00 | 0.00 | 0.00 | 0.01 | 0.04 | 0.00 | *Proteobacteria* | *Betaproteobacteria* | *Burkholderiales* | *Burkholderiales_incertae_sedis* | Transient-rare taxa |
| OTU 957 | 0.01 | 0.02 | 0.00 | 0.00 | 0.00 | 0.00 | 0.00 | 0.00 | 0.02 | 0.00 | 0.00 | 0.00 | *Proteobacteria* | *Alphaproteobacteria* | *Sphingomonadales* | *Sphingomonadaceae* | Transient-rare taxa |
| OTU 958 | 0.01 | 0.00 | 0.00 | 0.00 | 0.00 | 0.00 | 0.00 | 0.00 | 0.04 | 0.00 | 0.00 | 0.00 | *Proteobacteria* | *Alphaproteobacteria* | *Sphingomonadales* | *Sphingomonadaceae* | Transient-rare taxa |
| OTU 959 | 0.00 | 0.04 | 0.00 | 0.00 | 0.00 | 0.00 | 0.00 | 0.00 | 0.01 | 0.00 | 0.00 | 0.00 | *Proteobacteria* | *Alphaproteobacteria* | *Sphingomonadales* | *Sphingomonadaceae* | Transient-rare taxa |
| OTU 961 | 0.00 | 0.01 | 0.00 | 0.03 | 0.00 | 0.00 | 0.00 | 0.00 | 0.00 | 0.00 | 0.01 | 0.00 | *Proteobacteria* | *Betaproteobacteria* | *Burkholderiales* | *Burkholderiales_incertae_sedis* | Transient-rare taxa |
| OTU 962 | 0.00 | 0.00 | 0.00 | 0.01 | 0.00 | 0.00 | 0.02 | 0.02 | 0.00 | 0.00 | 0.00 | 0.00 | *Nitrospirae* | *Nitrospira* | *Nitrospirales* | *Nitrospiraceae* | Transient-rare taxa |
| OTU 963 | 0.01 | 0.03 | 0.00 | 0.00 | 0.00 | 0.00 | 0.00 | 0.00 | 0.01 | 0.00 | 0.00 | 0.00 | *Planctomycetes* | *Planctomycetia* | *Planctomycetales* | *Planctomycetaceae* | Transient-rare taxa |
| OTU 964 | 0.01 | 0.04 | 0.00 | 0.00 | 0.00 | 0.00 | 0.00 | 0.00 | 0.00 | 0.00 | 0.00 | 0.00 | *Proteobacteria* | *Gammaproteobacteria* | *Chromatiales* | *Chromatiaceae* | Transient-rare taxa |
| OTU 965 | 0.00 | 0.01 | 0.02 | 0.00 | 0.00 | 0.00 | 0.00 | 0.00 | 0.00 | 0.00 | 0.00 | 0.02 | *Bacteroidetes* | *Bacteroidetes_incertae_sedis* | *Ohtaekwangia* | | Transient-rare taxa |
| OTU 966 | 0.00 | 0.02 | 0.00 | 0.00 | 0.00 | 0.00 | 0.00 | 0.00 | 0.00 | 0.01 | 0.02 | 0.00 | *Bacteroidetes* | *Cytophagia* | *Cytophagales* | *Flammeovirgaceae* | Transient-rare taxa |
| OTU 967 | 0.00 | 0.00 | 0.00 | 0.00 | 0.00 | 0.00 | 0.06 | 0.00 | 0.00 | 0.00 | 0.00 | 0.00 | *Armatimonadetes* | *Fimbriimonadia* | *Fimbriimonadales* | *Fimbriimonadaceae* | Transient-rare taxa |
| OTU 968 | 0.00 | 0.02 | 0.00 | 0.00 | 0.00 | 0.00 | 0.00 | 0.00 | 0.03 | 0.00 | 0.00 | 0.00 | *Bacteroidetes* | *Sphingobacteriia* | *Sphingobacteriales* | *Chitinophagaceae* | Transient-rare taxa |
| OTU 969 | 0.00 | 0.01 | 0.00 | 0.00 | 0.00 | 0.00 | 0.00 | 0.00 | 0.04 | 0.00 | 0.00 | 0.00 | *Proteobacteria* | *Alphaproteobacteria* | *Rhizobiales* | *Hyphomicrobiaceae* | Transient-rare taxa |
| OTU 971 | 0.00 | 0.00 | 0.00 | 0.00 | 0.00 | 0.01 | 0.00 | 0.04 | 0.00 | 0.00 | 0.00 | 0.00 | *Proteobacteria* | *Deltaproteobacteria* | *Myxococcales* | *Sorangiineae* | Transient-rare taxa |
| OTU 972 | 0.00 | 0.02 | 0.00 | 0.00 | 0.00 | 0.00 | 0.00 | 0.03 | 0.00 | 0.00 | 0.00 | 0.00 | *Chloroflexi* | *Ardenticatenia* | *Ardenticatenales* | *Ardenticatenaceae* | Transient-rare taxa |
| OTU 973 | 0.00 | 0.00 | 0.00 | 0.06 | 0.00 | 0.00 | 0.00 | 0.00 | 0.00 | 0.00 | 0.00 | 0.00 | *Bacteroidetes* | *Bacteroidetes_incertae_sedis* | *Marinifilum* | | Transient-rare taxa |
| OTU 976 | 0.00 | 0.00 | 0.01 | 0.00 | 0.00 | 0.00 | 0.00 | 0.00 | 0.03 | 0.01 | 0.00 | 0.00 | *Proteobacteria* | *Alphaproteobacteria* | *Rhodobacterales* | *Rhodobacteraceae* | Transient-rare taxa |
| OTU 977 | 0.01 | 0.00 | 0.00 | 0.00 | 0.00 | 0.00 | 0.02 | 0.00 | 0.02 | 0.00 | 0.00 | 0.00 | *Proteobacteria* | *Gammaproteobacteria* | *Oceanospirillales* | *Oceanospirillaceae* | Transient-rare taxa |
| OTU 978 | 0.00 | 0.02 | 0.00 | 0.00 | 0.00 | 0.00 | 0.00 | 0.02 | 0.01 | 0.00 | 0.00 | 0.00 | *Bacteroidetes* | *Sphingobacteriia* | *Sphingobacteriales* | *Saprospiraceae* | Transient-rare taxa |
| OTU 980 | 0.00 | 0.02 | 0.00 | 0.00 | 0.00 | 0.00 | 0.03 | 0.00 | 0.00 | 0.00 | 0.00 | 0.00 | *Proteobacteria* | *Betaproteobacteria* | *Rhodocyclales* | *Rhodocyclaceae* | Transient-rare taxa |
| OTU 981 | 0.03 | 0.02 | 0.00 | 0.00 | 0.00 | 0.00 | 0.00 | 0.00 | 0.00 | 0.00 | 0.00 | 0.00 | *Proteobacteria* | *Gammaproteobacteria* | *Chromatiales* | *Chromatiaceae* | Transient-rare taxa |
| OTU 982 | 0.00 | 0.02 | 0.02 | 0.01 | 0.00 | 0.00 | 0.00 | 0.00 | 0.00 | 0.00 | 0.00 | 0.00 | *Proteobacteria* | *Betaproteobacteria* | *Rhodocyclales* | *Rhodocyclaceae* | Transient-rare taxa |
| OTU 983 | 0.00 | 0.02 | 0.01 | 0.00 | 0.00 | 0.00 | 0.00 | 0.02 | 0.00 | 0.00 | 0.00 | 0.00 | *Proteobacteria* | *Deltaproteobacteria* | *Myxococcales* | *Cystobacterineae* | Transient-rare taxa |
| OTU 984 | 0.00 | 0.00 | 0.00 | 0.00 | 0.00 | 0.03 | 0.00 | 0.01 | 0.01 | 0.00 | 0.00 | 0.00 | *Planctomycetes* | *Planctomycetia* | *Planctomycetales* | *Planctomycetaceae* | Transient-rare taxa |
| OTU 985 | 0.01 | 0.04 | 0.00 | 0.00 | 0.00 | 0.00 | 0.00 | 0.00 | 0.00 | 0.00 | 0.00 | 0.00 | *Proteobacteria* | *Deltaproteobacteria* | *Myxococcales* | *Sorangiineae* | Transient-rare taxa |
| OTU 987 | 0.00 | 0.03 | 0.00 | 0.00 | 0.00 | 0.00 | 0.01 | 0.00 | 0.00 | 0.00 | 0.00 | 0.01 | *Proteobacteria* | *Deltaproteobacteria* | *Myxococcales* | *Nannocystineae* | Transient-rare taxa |
| OTU 989 | 0.00 | 0.02 | 0.00 | 0.00 | 0.00 | 0.01 | 0.00 | 0.00 | 0.02 | 0.00 | 0.00 | 0.00 | *Planctomycetes* | *Planctomycetia* | *Planctomycetales* | *Planctomycetaceae* | Transient-rare taxa |
| OTU 990 | 0.00 | 0.06 | 0.00 | 0.00 | 0.00 | 0.00 | 0.00 | 0.00 | 0.00 | 0.00 | 0.00 | 0.00 | *Proteobacteria* | *Betaproteobacteria* | *Rhodocyclales* | *Rhodocyclaceae* | Transient-rare taxa |
| OTU 991 | 0.00 | 0.00 | 0.00 | 0.01 | 0.00 | 0.02 | 0.00 | 0.02 | 0.00 | 0.00 | 0.00 | 0.00 | *Proteobacteria* | *Alphaproteobacteria* | *Rhizobiales* | *Bradyrhizobiaceae* | Transient-rare taxa |
| OTU 992 | 0.00 | 0.00 | 0.00 | 0.06 | 0.00 | 0.00 | 0.00 | 0.00 | 0.00 | 0.00 | 0.00 | 0.00 | *Proteobacteria* | *Alphaproteobacteria* | *Rhizobiales* | *Brucellaceae* | Transient-rare taxa |
| OTU 993 | 0.00 | 0.00 | 0.00 | 0.00 | 0.04 | 0.01 | 0.00 | 0.00 | 0.00 | 0.00 | 0.00 | 0.00 | *Bacteroidetes* | *Flavobacteriia* | *Flavobacteriales* | *Flavobacteriaceae* | Transient-rare taxa |
| OTU 994 | 0.00 | 0.00 | 0.00 | 0.00 | 0.00 | 0.02 | 0.02 | 0.01 | 0.00 | 0.00 | 0.00 | 0.00 | *Proteobacteria* | *Betaproteobacteria* | *Burkholderiales* | *Burkholderiales_incertae_sedis* | Transient-rare taxa |
| OTU 995 | 0.00 | 0.00 | 0.00 | 0.02 | 0.01 | 0.00 | 0.02 | 0.00 | 0.00 | 0.00 | 0.00 | 0.00 | *Proteobacteria* | *Alphaproteobacteria* | *Sneathiellales* | *Sneathiellaceae* | Transient-rare taxa |
| OTU 996 | 0.00 | 0.00 | 0.00 | 0.00 | 0.01 | 0.00 | 0.02 | 0.00 | 0.00 | 0.00 | 0.00 | 0.02 | *Proteobacteria* | *Alphaproteobacteria* | *Rhizobiales* | *Bradyrhizobiaceae* | Transient-rare taxa |
| OTU 997 | 0.00 | 0.00 | 0.00 | 0.00 | 0.00 | 0.04 | 0.01 | 0.00 | 0.00 | 0.00 | 0.00 | 0.00 | *Proteobacteria* | *Betaproteobacteria* | *Burkholderiales* | *Burkholderiales_incertae_sedis* | Transient-rare taxa |
| OTU 998 | 0.00 | 0.00 | 0.00 | 0.00 | 0.00 | 0.00 | 0.00 | 0.04 | 0.00 | 0.00 | 0.00 | 0.01 | *Planctomycetes* | *Planctomycetia* | *Planctomycetales* | *Planctomycetaceae* | Transient-rare taxa |
| OTU 999 | 0.00 | 0.06 | 0.00 | 0.00 | 0.00 | 0.00 | 0.00 | 0.00 | 0.00 | 0.00 | 0.00 | 0.00 | *Proteobacteria* | *Alphaproteobacteria* | *Rhodospirillales* | *Acetobacteraceae* | Transient-rare taxa |
| OTU 1000 | 0.00 | 0.00 | 0.00 | 0.00 | 0.00 | 0.00 | 0.00 | 0.00 | 0.01 | 0.01 | 0.03 | 0.00 | *Proteobacteria* | *Betaproteobacteria* | *Rhodocyclales* | *Rhodocyclaceae* | Transient-rare taxa |
| OTU 1003 | 0.00 | 0.01 | 0.00 | 0.00 | 0.00 | 0.04 | 0.00 | 0.00 | 0.00 | 0.00 | 0.00 | 0.00 | *Proteobacteria* | *Betaproteobacteria* | *Nitrosomonadales* | *Nitrosomonadaceae* | Transient-rare taxa |
| OTU 1004 | 0.00 | 0.00 | 0.00 | 0.00 | 0.00 | 0.00 | 0.00 | 0.06 | 0.00 | 0.00 | 0.00 | 0.00 | *Planctomycetes* | *Planctomycetia* | *Planctomycetales* | *Planctomycetaceae* | Transient-rare taxa |
| OTU 1005 | 0.00 | 0.00 | 0.00 | 0.00 | 0.00 | 0.00 | 0.03 | 0.02 | 0.00 | 0.00 | 0.00 | 0.00 | *Nitrospirae* | *Nitrospira* | *Nitrospirales* | *Nitrospiraceae* | Transient-rare taxa |
| OTU 1007 | 0.00 | 0.00 | 0.00 | 0.00 | 0.00 | 0.00 | 0.00 | 0.00 | 0.00 | 0.00 | 0.01 | 0.04 | *Candidatus Saccharibacteria* | *Saccharibacteria_genera_incertae_sedis* | | | Transient-rare taxa |
| OTU 1008 | 0.06 | 0.00 | 0.00 | 0.00 | 0.00 | 0.00 | 0.00 | 0.00 | 0.00 | 0.00 | 0.00 | 0.00 | *Proteobacteria* | *Gammaproteobacteria* | *Chromatiales* | *Chromatiaceae* | Transient-rare taxa |
| OTU 1009 | 0.06 | 0.00 | 0.00 | 0.00 | 0.00 | 0.00 | 0.00 | 0.00 | 0.00 | 0.00 | 0.00 | 0.00 | *Proteobacteria* | *Alphaproteobacteria* | *Rhodospirillales* | *Rhodospirillaceae* | Transient-rare taxa |
| OTU 1010 | 0.00 | 0.00 | 0.00 | 0.00 | 0.00 | 0.00 | 0.00 | 0.06 | 0.00 | 0.00 | 0.00 | 0.00 | *Bacteroidetes* | *Sphingobacteriia* | *Sphingobacteriales* | *Saprospiraceae* | Transient-rare taxa |
| OTU 1011 | 0.02 | 0.00 | 0.00 | 0.00 | 0.00 | 0.00 | 0.00 | 0.00 | 0.00 | 0.00 | 0.00 | 0.03 | *Proteobacteria* | *Deltaproteobacteria* | *Myxococcales* | *Sorangiineae* | Transient-rare taxa |
| OTU 1012 | 0.00 | 0.00 | 0.00 | 0.00 | 0.00 | 0.00 | 0.00 | 0.06 | 0.00 | 0.00 | 0.00 | 0.00 | *Proteobacteria* | *Deltaproteobacteria* | *Myxococcales* | *Sorangiineae* | Transient-rare taxa |
| OTU 1013 | 0.00 | 0.00 | 0.00 | 0.00 | 0.00 | 0.00 | 0.00 | 0.06 | 0.00 | 0.00 | 0.00 | 0.00 | *Proteobacteria* | *Deltaproteobacteria* | *Myxococcales* | *Nannocystineae* | Transient-rare taxa |
| OTU 1014 | 0.03 | 0.00 | 0.00 | 0.01 | 0.00 | 0.01 | 0.00 | 0.00 | 0.00 | 0.00 | 0.00 | 0.00 | *Proteobacteria* | *Gammaproteobacteria* | *Oceanospirillales* | *Hahellaceae* | Transient-rare taxa |
| OTU 1015 | 0.00 | 0.00 | 0.00 | 0.03 | 0.00 | 0.02 | 0.00 | 0.00 | 0.00 | 0.00 | 0.00 | 0.00 | *Bacteroidetes* | *Flavobacteriia* | *Flavobacteriales* | *Flavobacteriaceae* | Transient-rare taxa |
| OTU 1016 | 0.00 | 0.00 | 0.00 | 0.01 | 0.00 | 0.03 | 0.00 | 0.00 | 0.00 | 0.01 | 0.00 | 0.00 | *Firmicutes* | *Clostridia* | *Clostridiales* | *Peptostreptococcaceae* | Transient-rare taxa |
| OTU 1017 | 0.00 | 0.00 | 0.00 | 0.06 | 0.00 | 0.00 | 0.00 | 0.00 | 0.00 | 0.00 | 0.00 | 0.00 | *Bacteroidetes* | *Flavobacteriia* | *Flavobacteriales* | *Flavobacteriaceae* | Transient-rare taxa |
| OTU 1018 | 0.04 | 0.00 | 0.00 | 0.00 | 0.00 | 0.00 | 0.00 | 0.00 | 0.00 | 0.01 | 0.00 | 0.00 | *Proteobacteria* | *Betaproteobacteria* | *Rhodocyclales* | *Rhodocyclaceae* | Transient-rare taxa |
| OTU 1019 | 0.00 | 0.00 | 0.02 | 0.00 | 0.00 | 0.01 | 0.02 | 0.00 | 0.00 | 0.00 | 0.00 | 0.00 | *Proteobacteria* | *Gammaproteobacteria* | *Alteromonadales* | *Alteromonadaceae* | Transient-rare taxa |
| OTU 1020 | 0.04 | 0.00 | 0.01 | 0.00 | 0.00 | 0.00 | 0.00 | 0.00 | 0.00 | 0.00 | 0.00 | 0.00 | *Bacteroidetes* | *Sphingobacteriia* | *Sphingobacteriales* | *Saprospiraceae* | Transient-rare taxa |
| OTU 1021 | 0.03 | 0.02 | 0.00 | 0.00 | 0.00 | 0.00 | 0.00 | 0.00 | 0.00 | 0.00 | 0.00 | 0.00 | *Proteobacteria* | *Betaproteobacteria* | *Burkholderiales* | *Burkholderiales_incertae_sedis* | Transient-rare taxa |
| OTU 1023 | 0.06 | 0.00 | 0.00 | 0.00 | 0.00 | 0.00 | 0.00 | 0.00 | 0.00 | 0.00 | 0.00 | 0.00 | *Proteobacteria* | *Alphaproteobacteria* | *Rhizobiales* | *Brucellaceae* | Transient-rare taxa |
| OTU 1024 | 0.00 | 0.04 | 0.00 | 0.00 | 0.00 | 0.00 | 0.00 | 0.00 | 0.01 | 0.00 | 0.00 | 0.00 | *Proteobacteria* | *Deltaproteobacteria* | *Myxococcales* | *Sorangiineae* | Transient-rare taxa |
| OTU 1027 | 0.06 | 0.00 | 0.00 | 0.00 | 0.00 | 0.00 | 0.00 | 0.00 | 0.00 | 0.00 | 0.00 | 0.00 | *Proteobacteria* | *Deltaproteobacteria* | *Myxococcales* | *Nannocystineae* | Transient-rare taxa |
| OTU 1028 | 0.00 | 0.00 | 0.00 | 0.03 | 0.02 | 0.00 | 0.00 | 0.00 | 0.00 | 0.00 | 0.00 | 0.00 | *Proteobacteria* | *Alphaproteobacteria* | *Rhodobacterales* | *Rhodobacteraceae* | Transient-rare taxa |
| OTU 1029 | 0.00 | 0.03 | 0.00 | 0.00 | 0.00 | 0.00 | 0.00 | 0.01 | 0.00 | 0.00 | 0.00 | 0.01 | *Proteobacteria* | *Alphaproteobacteria* | *Rhizobiales* | *Methylobacteriaceae* | Transient-rare taxa |
| OTU 1030 | 0.06 | 0.00 | 0.00 | 0.00 | 0.00 | 0.00 | 0.00 | 0.00 | 0.00 | 0.00 | 0.00 | 0.00 | *Bacteroidetes* | *Sphingobacteriia* | *Sphingobacteriales* | *Saprospiraceae* | Transient-rare taxa |
| OTU 1031 | 0.00 | 0.00 | 0.00 | 0.00 | 0.00 | 0.00 | 0.00 | 0.00 | 0.00 | 0.00 | 0.00 | 0.06 | *Proteobacteria* | *Betaproteobacteria* | *Burkholderiales* | *Oxalobacteraceae* | Transient-rare taxa |
| OTU 1032 | 0.01 | 0.00 | 0.00 | 0.00 | 0.00 | 0.00 | 0.00 | 0.00 | 0.00 | 0.01 | 0.00 | 0.03 | *Bacteroidetes* | *Sphingobacteriia* | *Sphingobacteriales* | *Sphingobacteriaceae* | Transient-rare taxa |
| OTU 1033 | 0.04 | 0.00 | 0.00 | 0.00 | 0.00 | 0.00 | 0.00 | 0.00 | 0.00 | 0.00 | 0.00 | 0.01 | *Proteobacteria* | *Betaproteobacteria* | *Rhodocyclales* | *Rhodocyclaceae* | Transient-rare taxa |
| OTU 1034 | 0.04 | 0.01 | 0.00 | 0.00 | 0.00 | 0.00 | 0.00 | 0.00 | 0.00 | 0.00 | 0.00 | 0.00 | *Proteobacteria* | *Alphaproteobacteria* | *Sphingomonadales* | *Erythrobacteraceae* | Transient-rare taxa |
| OTU 1035 | 0.01 | 0.00 | 0.00 | 0.00 | 0.00 | 0.00 | 0.00 | 0.00 | 0.00 | 0.00 | 0.00 | 0.04 | *Proteobacteria* | *Deltaproteobacteria* | *Myxococcales* | *Nannocystineae* | Transient-rare taxa |
| OTU 1037 | 0.01 | 0.00 | 0.00 | 0.00 | 0.00 | 0.00 | 0.00 | 0.00 | 0.00 | 0.00 | 0.02 | 0.02 | *Proteobacteria* | *Betaproteobacteria* | *Burkholderiales* | *Comamonadaceae* | Transient-rare taxa |
| OTU 1038 | 0.00 | 0.00 | 0.04 | 0.00 | 0.00 | 0.00 | 0.00 | 0.00 | 0.01 | 0.00 | 0.00 | 0.00 | *Proteobacteria* | *Gammaproteobacteria* | *Thiotrichales* | *Thiotrichaceae* | Transient-rare taxa |
| OTU 1039 | 0.00 | 0.00 | 0.00 | 0.00 | 0.00 | 0.00 | 0.00 | 0.00 | 0.00 | 0.00 | 0.01 | 0.04 | *Gemmatimonadetes* | *Gemmatimonadetes* | *Gemmatimonadales* | *Gemmatimonadaceae* | Transient-rare taxa |
| OTU 1040 | 0.00 | 0.00 | 0.04 | 0.00 | 0.00 | 0.00 | 0.00 | 0.00 | 0.00 | 0.00 | 0.00 | 0.01 | *Proteobacteria* | *Deltaproteobacteria* | *Myxococcales* | *Sorangiineae* | Transient-rare taxa |
| OTU 1043 | 0.00 | 0.00 | 0.00 | 0.00 | 0.00 | 0.00 | 0.00 | 0.00 | 0.00 | 0.00 | 0.02 | 0.03 | *Proteobacteria* | *Alphaproteobacteria* | *Rhodobacterales* | *Rhodobacteraceae* | Transient-rare taxa |
| OTU 1044 | 0.00 | 0.02 | 0.00 | 0.00 | 0.00 | 0.00 | 0.00 | 0.00 | 0.00 | 0.00 | 0.00 | 0.03 | *Proteobacteria* | *Betaproteobacteria* | *Rhodocyclales* | *Rhodocyclaceae* | Transient-rare taxa |
| OTU 1045 | 0.00 | 0.00 | 0.00 | 0.00 | 0.00 | 0.00 | 0.00 | 0.06 | 0.00 | 0.00 | 0.00 | 0.00 | *Bacteroidetes* | *Sphingobacteriia* | *Sphingobacteriales* | *Saprospiraceae* | Transient-rare taxa |
| OTU 1046 | 0.00 | 0.00 | 0.00 | 0.00 | 0.00 | 0.00 | 0.00 | 0.00 | 0.00 | 0.01 | 0.00 | 0.04 | *Proteobacteria* | *Deltaproteobacteria* | *Myxococcales* | *Nannocystineae* | Transient-rare taxa |
| OTU 1047 | 0.00 | 0.00 | 0.00 | 0.00 | 0.00 | 0.00 | 0.00 | 0.00 | 0.00 | 0.01 | 0.00 | 0.04 | *Bacteroidetes* | *Sphingobacteriia* | *Sphingobacteriales* | *Sphingobacteriaceae* | Transient-rare taxa |
| OTU 1049 | 0.00 | 0.00 | 0.01 | 0.00 | 0.00 | 0.00 | 0.00 | 0.00 | 0.00 | 0.00 | 0.04 | 0.00 | *Proteobacteria* | *Alphaproteobacteria* | *Rhodobacterales* | *Rhodobacteraceae* | Transient-rare taxa |
| OTU 1050 | 0.00 | 0.00 | 0.03 | 0.00 | 0.00 | 0.00 | 0.00 | 0.00 | 0.00 | 0.01 | 0.01 | 0.00 | *Bacteroidetes* | *Sphingobacteriia* | *Sphingobacteriales* | *Chitinophagaceae* | Transient-rare taxa |
| OTU 1051 | 0.00 | 0.00 | 0.00 | 0.01 | 0.01 | 0.00 | 0.00 | 0.00 | 0.00 | 0.03 | 0.00 | 0.00 | *Firmicutes* | *Clostridia* | *Clostridiales* | *Clostridiales_Incertae Sedis XII* | Transient-rare taxa |
| OTU 1053 | 0.00 | 0.00 | 0.00 | 0.00 | 0.00 | 0.01 | 0.00 | 0.00 | 0.00 | 0.01 | 0.00 | 0.03 | *Proteobacteria* | *Deltaproteobacteria* | *Myxococcales* | *Sorangiineae* | Transient-rare taxa |
| OTU 1054 | 0.00 | 0.00 | 0.00 | 0.00 | 0.00 | 0.01 | 0.00 | 0.00 | 0.00 | 0.02 | 0.00 | 0.02 | *Proteobacteria* | *Gammaproteobacteria* | *Pseudomonadales* | *Pseudomonadaceae* | Transient-rare taxa |
| OTU 1055 | 0.04 | 0.00 | 0.00 | 0.00 | 0.00 | 0.00 | 0.00 | 0.00 | 0.00 | 0.01 | 0.00 | 0.00 | *Chloroflexi* | *Thermomicrobia* | *Sphaerobacteridae* | *Sphaerobacterales* | Transient-rare taxa |
| OTU 1057 | 0.02 | 0.00 | 0.00 | 0.00 | 0.00 | 0.00 | 0.00 | 0.00 | 0.01 | 0.00 | 0.00 | 0.02 | *Bacteroidetes* | *Sphingobacteriia* | *Sphingobacteriales* | *Chitinophagaceae* | Transient-rare taxa |
| OTU 1059 | 0.00 | 0.00 | 0.04 | 0.01 | 0.00 | 0.00 | 0.00 | 0.00 | 0.00 | 0.00 | 0.00 | 0.00 | *Bacteroidetes* | *Bacteroidia* | *Bacteroidales* | *Marinilabiaceae* | Transient-rare taxa |
| OTU 1060 | 0.00 | 0.00 | 0.00 | 0.00 | 0.00 | 0.00 | 0.00 | 0.00 | 0.00 | 0.04 | 0.01 | 0.00 | *Bacteroidetes* | *Sphingobacteriia* | *Sphingobacteriales* | *Chitinophagaceae* | Transient-rare taxa |
| OTU 1061 | 0.00 | 0.00 | 0.00 | 0.00 | 0.00 | 0.00 | 0.00 | 0.00 | 0.01 | 0.01 | 0.03 | 0.00 | *Proteobacteria* | *Deltaproteobacteria* | *Myxococcales* | *Sorangiineae* | Transient-rare taxa |
| OTU 1062 | 0.00 | 0.00 | 0.00 | 0.00 | 0.00 | 0.03 | 0.01 | 0.01 | 0.00 | 0.00 | 0.00 | 0.00 | *Proteobacteria* | *Deltaproteobacteria* | *Desulfuromonadales* | *Geobacteraceae* | Transient-rare taxa |
| OTU 1063 | 0.00 | 0.02 | 0.00 | 0.00 | 0.00 | 0.00 | 0.00 | 0.03 | 0.00 | 0.00 | 0.00 | 0.00 | *Proteobacteria* | *Deltaproteobacteria* | *Myxococcales* | *Nannocystineae* | Transient-rare taxa |
| OTU 1064 | 0.00 | 0.00 | 0.00 | 0.00 | 0.00 | 0.00 | 0.00 | 0.00 | 0.00 | 0.06 | 0.00 | 0.00 | *Proteobacteria* | *Alphaproteobacteria* | *Rhodospirillales* | *Rhodospirillaceae* | Transient-rare taxa |
| OTU 1066 | 0.00 | 0.00 | 0.00 | 0.00 | 0.00 | 0.00 | 0.00 | 0.04 | 0.01 | 0.00 | 0.00 | 0.00 | *Proteobacteria* | *Gammaproteobacteria* | *Oceanospirillales* | *Hahellaceae* | Transient-rare taxa |
| OTU 1067 | 0.01 | 0.00 | 0.00 | 0.00 | 0.00 | 0.03 | 0.00 | 0.00 | 0.00 | 0.01 | 0.00 | 0.00 | *Bacteroidetes* | *Cytophagia* | *Cytophagales* | *Cytophagaceae* | Transient-rare taxa |
| OTU 1068 | 0.00 | 0.00 | 0.00 | 0.00 | 0.06 | 0.00 | 0.00 | 0.00 | 0.00 | 0.00 | 0.00 | 0.00 | *Proteobacteria* | *Gammaproteobacteria* | *Alteromonadales* | *Alteromonadaceae* | Transient-rare taxa |
| OTU 1069 | 0.00 | 0.00 | 0.00 | 0.00 | 0.01 | 0.03 | 0.00 | 0.00 | 0.00 | 0.01 | 0.00 | 0.00 | *Chloroflexi* | *Caldilineae* | *Caldilineales* | *Caldilineaceae* | Transient-rare taxa |
| OTU 1070 | 0.01 | 0.00 | 0.03 | 0.00 | 0.00 | 0.00 | 0.00 | 0.00 | 0.00 | 0.00 | 0.00 | 0.01 | *Spirochaetes* | *Spirochaetia* | *Spirochaetales* | *Leptospiraceae* | Transient-rare taxa |
| OTU 1072 | 0.01 | 0.00 | 0.00 | 0.00 | 0.00 | 0.00 | 0.00 | 0.00 | 0.00 | 0.00 | 0.04 | 0.00 | *Proteobacteria* | *Gammaproteobacteria* | *Oceanospirillales* | *Halomonadaceae* | Transient-rare taxa |
| OTU 1073 | 0.00 | 0.00 | 0.00 | 0.00 | 0.00 | 0.00 | 0.00 | 0.00 | 0.00 | 0.00 | 0.06 | 0.00 | *Proteobacteria* | *Deltaproteobacteria* | *Bdellovibrionales* | *Bacteriovoracaceae* | Transient-rare taxa |
| OTU 1076 | 0.00 | 0.00 | 0.00 | 0.00 | 0.01 | 0.00 | 0.00 | 0.00 | 0.00 | 0.02 | 0.00 | 0.02 | *Bacteroidetes* | *Bacteroidia* | *Bacteroidales* | *Porphyromonadaceae* | Transient-rare taxa |
| OTU 1079 | 0.02 | 0.00 | 0.00 | 0.00 | 0.02 | 0.00 | 0.00 | 0.00 | 0.00 | 0.00 | 0.00 | 0.01 | *Proteobacteria* | *Alphaproteobacteria* | *Caulobacterales* | *Caulobacteraceae* | Transient-rare taxa |
| OTU 1080 | 0.00 | 0.00 | 0.00 | 0.00 | 0.00 | 0.03 | 0.02 | 0.00 | 0.00 | 0.00 | 0.00 | 0.00 | *candidate division WPS-1* | *WPS-1_genera_incertae_sedis* | | | Transient-rare taxa |
| OTU 1081 | 0.00 | 0.00 | 0.00 | 0.01 | 0.00 | 0.00 | 0.00 | 0.00 | 0.00 | 0.00 | 0.02 | 0.02 | *Bacteroidetes* | *Flavobacteriia* | *Flavobacteriales* | *Cryomorphaceae* | Transient-rare taxa |
| OTU 1083 | 0.00 | 0.00 | 0.00 | 0.00 | 0.00 | 0.00 | 0.00 | 0.00 | 0.00 | 0.00 | 0.01 | 0.04 | *Bacteroidetes* | *Cytophagia* | *Cytophagales* | *Flammeovirgaceae* | Transient-rare taxa |
| OTU 1084 | 0.00 | 0.00 | 0.00 | 0.00 | 0.06 | 0.00 | 0.00 | 0.00 | 0.00 | 0.00 | 0.00 | 0.00 | *Bacteroidetes* | *Flavobacteriia* | *Flavobacteriales* | *Cryomorphaceae* | Transient-rare taxa |
| OTU 1085 | 0.00 | 0.00 | 0.00 | 0.03 | 0.00 | 0.02 | 0.00 | 0.00 | 0.00 | 0.00 | 0.00 | 0.00 | *Bacteroidetes* | *Sphingobacteriia* | *Sphingobacteriales* | *Chitinophagaceae* | Transient-rare taxa |
| OTU 1086 | 0.00 | 0.00 | 0.00 | 0.00 | 0.03 | 0.02 | 0.00 | 0.00 | 0.00 | 0.00 | 0.00 | 0.00 | *Bacteroidetes* | *Sphingobacteriia* | *Sphingobacteriales* | *Saprospiraceae* | Transient-rare taxa |
| OTU 1087 | 0.00 | 0.02 | 0.03 | 0.00 | 0.00 | 0.00 | 0.00 | 0.00 | 0.00 | 0.00 | 0.00 | 0.00 | *Bacteroidetes* | *Sphingobacteriia* | *Sphingobacteriales* | *Saprospiraceae* | Transient-rare taxa |
| OTU 1088 | 0.00 | 0.00 | 0.06 | 0.00 | 0.00 | 0.00 | 0.00 | 0.00 | 0.00 | 0.00 | 0.00 | 0.00 | *Bacteroidetes* | *Sphingobacteriia* | *Sphingobacteriales* | *Chitinophagaceae* | Transient-rare taxa |
| OTU 1091 | 0.00 | 0.00 | 0.00 | 0.00 | 0.00 | 0.04 | 0.00 | 0.01 | 0.00 | 0.00 | 0.00 | 0.00 | *Proteobacteria* | *Gammaproteobacteria* | *Methylococcales* | *Methylococcaceae* | Transient-rare taxa |
| OTU 1092 | 0.00 | 0.00 | 0.00 | 0.00 | 0.00 | 0.00 | 0.00 | 0.00 | 0.00 | 0.00 | 0.00 | 0.06 | *Proteobacteria* | *Alphaproteobacteria* | *Kiloniellales* | *Kiloniellaceae* | Transient-rare taxa |
| OTU 1094 | 0.00 | 0.00 | 0.00 | 0.00 | 0.00 | 0.00 | 0.01 | 0.01 | 0.02 | 0.00 | 0.00 | 0.00 | *Proteobacteria* | *Betaproteobacteria* | *Burkholderiales* | *Burkholderiaceae* | Transient-rare taxa |
| OTU 1095 | 0.00 | 0.00 | 0.03 | 0.00 | 0.00 | 0.00 | 0.00 | 0.01 | 0.00 | 0.00 | 0.00 | 0.00 | *Proteobacteria* | *Alphaproteobacteria* | *Rhizobiales* | *Methylocystaceae* | Transient-rare taxa |
| OTU 1096 | 0.00 | 0.00 | 0.00 | 0.00 | 0.00 | 0.01 | 0.01 | 0.00 | 0.00 | 0.02 | 0.00 | 0.00 | *Actinobacteria* | *Actinobacteria* | *Actinobacteridae* | *Actinomycetales* | Transient-rare taxa |
| OTU 1098 | 0.00 | 0.02 | 0.02 | 0.00 | 0.00 | 0.00 | 0.00 | 0.00 | 0.00 | 0.00 | 0.00 | 0.00 | *Chloroflexi* | *Ardenticatenia* | *Ardenticatenales* | *Ardenticatenaceae* | Transient-rare taxa |
| OTU 1099 | 0.00 | 0.00 | 0.00 | 0.01 | 0.03 | 0.00 | 0.00 | 0.00 | 0.00 | 0.00 | 0.00 | 0.00 | *Proteobacteria* | *Betaproteobacteria* | *Rhodocyclales* | *Rhodocyclaceae* | Transient-rare taxa |
| OTU 1100 | 0.00 | 0.02 | 0.01 | 0.00 | 0.00 | 0.00 | 0.00 | 0.00 | 0.01 | 0.00 | 0.00 | 0.00 | *Proteobacteria* | *Gammaproteobacteria* | *Xanthomonadales* | *Xanthomonadaceae* | Transient-rare taxa |
| OTU 1101 | 0.00 | 0.00 | 0.00 | 0.00 | 0.00 | 0.00 | 0.00 | 0.01 | 0.03 | 0.00 | 0.00 | 0.00 | *Planctomycetes* | *Planctomycetia* | *Planctomycetales* | *Planctomycetaceae* | Transient-rare taxa |
| OTU 1102 | 0.00 | 0.00 | 0.00 | 0.01 | 0.00 | 0.00 | 0.01 | 0.00 | 0.02 | 0.00 | 0.00 | 0.00 | *Proteobacteria* | *Deltaproteobacteria* | *Desulfobacterales* | *Desulfobacteraceae* | Transient-rare taxa |

| | | | | | | | | | | | | | | | | |
|---|---|---|---|---|---|---|---|---|---|---|---|---|---|---|---|---|---|
| OTU 1103 | 0.00 | 0.00 | 0.00 | 0.00 | 0.00 | 0.00 | 0.03 | 0.00 | 0.01 | 0.00 | 0.00 | 0.00 | *Proteobacteria* | *Deltaproteobacteria* | *Myxococcales* | *Nannocystineae* | Transient-rare taxa |
| OTU 1104 | 0.00 | 0.01 | 0.00 | 0.00 | 0.00 | 0.00 | 0.00 | 0.00 | 0.03 | 0.00 | 0.00 | 0.00 | *Proteobacteria* | *Betaproteobacteria* | *Rhodocyclales* | *Rhodocyclaceae* | Transient-rare taxa |
| OTU 1106 | 0.01 | 0.02 | 0.00 | 0.00 | 0.00 | 0.00 | 0.00 | 0.00 | 0.00 | 0.00 | 0.00 | 0.01 | *Chloroflexi* | *Anaerolineae* | *Anaerolineales* | *Anaerolineaceae* | Transient-rare taxa |
| OTU 1107 | 0.00 | 0.00 | 0.00 | 0.00 | 0.01 | 0.00 | 0.02 | 0.01 | 0.00 | 0.00 | 0.00 | 0.00 | *Proteobacteria* | *Gammaproteobacteria* | *Oceanospirillales* | *Oceanospirillaceae* | Transient-rare taxa |
| OTU 1108 | 0.00 | 0.00 | 0.00 | 0.00 | 0.00 | 0.00 | 0.00 | 0.02 | 0.02 | 0.00 | 0.00 | 0.00 | *Proteobacteria* | *Deltaproteobacteria* | *Myxococcales* | *Sorangiineae* | Transient-rare taxa |
| OTU 1109 | 0.00 | 0.00 | 0.00 | 0.00 | 0.00 | 0.00 | 0.00 | 0.03 | 0.01 | 0.00 | 0.00 | 0.00 | *Chloroflexi* | *Anaerolineae* | *Anaerolineales* | *Anaerolineaceae* | Transient-rare taxa |
| OTU 1110 | 0.01 | 0.03 | 0.00 | 0.00 | 0.00 | 0.00 | 0.00 | 0.00 | 0.00 | 0.00 | 0.00 | 0.00 | *Proteobacteria* | *Betaproteobacteria* | *Rhodocyclales* | *Rhodocyclaceae* | Transient-rare taxa |
| OTU 1112 | 0.01 | 0.03 | 0.00 | 0.00 | 0.00 | 0.00 | 0.00 | 0.00 | 0.00 | 0.00 | 0.00 | 0.00 | *Proteobacteria* | *Betaproteobacteria* | *Neisseriales* | *Neisseriaceae* | Transient-rare taxa |
| OTU 1113 | 0.00 | 0.01 | 0.00 | 0.00 | 0.00 | 0.00 | 0.00 | 0.00 | 0.03 | 0.00 | 0.00 | 0.00 | *Bacteroidetes* | *Sphingobacteriia* | *Sphingobacteriales* | *Saprospiraceae* | Transient-rare taxa |
| OTU 1114 | 0.00 | 0.02 | 0.01 | 0.00 | 0.01 | 0.00 | 0.00 | 0.00 | 0.00 | 0.00 | 0.00 | 0.00 | *Proteobacteria* | *Betaproteobacteria* | *Burkholderiales* | *Comamonadaceae* | Transient-rare taxa |
| OTU 1115 | 0.01 | 0.02 | 0.00 | 0.00 | 0.00 | 0.00 | 0.00 | 0.00 | 0.01 | 0.00 | 0.00 | 0.00 | *Proteobacteria* | *Gammaproteobacteria* | *Chromatiales* | *Ectothiorhodospiraceae* | Transient-rare taxa |
| OTU 1116 | 0.00 | 0.00 | 0.00 | 0.00 | 0.00 | 0.01 | 0.02 | 0.00 | 0.01 | 0.00 | 0.00 | 0.00 | *Proteobacteria* | *Gammaproteobacteria* | *Thiotrichales* | *Thiotrichaceae* | Transient-rare taxa |
| OTU 1117 | 0.00 | 0.00 | 0.00 | 0.00 | 0.00 | 0.00 | 0.00 | 0.00 | 0.02 | 0.01 | 0.01 | 0.00 | *Bacteroidetes* | *Sphingobacteriia* | *Sphingobacteriales* | *Sphingobacteriaceae* | Transient-rare taxa |
| OTU 1118 | 0.00 | 0.00 | 0.00 | 0.00 | 0.00 | 0.00 | 0.00 | 0.00 | 0.00 | 0.00 | 0.04 | 0.00 | *Proteobacteria* | *Deltaproteobacteria* | *Myxococcales* | *Nannocystineae* | Transient-rare taxa |
| OTU 1119 | 0.00 | 0.00 | 0.00 | 0.00 | 0.00 | 0.00 | 0.00 | 0.01 | 0.00 | 0.00 | 0.03 | 0.00 | *Proteobacteria* | *Betaproteobacteria* | *Nitrosomonadales* | *Nitrosomonadaceae* | Transient-rare taxa |
| OTU 1120 | 0.00 | 0.00 | 0.00 | 0.03 | 0.00 | 0.00 | 0.01 | 0.00 | 0.00 | 0.00 | 0.00 | 0.00 | *Proteobacteria* | *Gammaproteobacteria* | *Alteromonadales* | *Alteromonadaceae* | Transient-rare taxa |
| OTU 1121 | 0.00 | 0.00 | 0.00 | 0.00 | 0.00 | 0.00 | 0.00 | 0.00 | 0.01 | 0.00 | 0.03 | 0.00 | *Proteobacteria* | *Deltaproteobacteria* | *Myxococcales* | *Nannocystineae* | Transient-rare taxa |
| OTU 1122 | 0.00 | 0.00 | 0.00 | 0.00 | 0.00 | 0.01 | 0.00 | 0.01 | 0.00 | 0.00 | 0.02 | 0.00 | *Proteobacteria* | *Alphaproteobacteria* | *Sphingomonadales* | *Sphingomonadaceae* | Transient-rare taxa |
| OTU 1124 | 0.00 | 0.04 | 0.00 | 0.00 | 0.00 | 0.00 | 0.00 | 0.00 | 0.00 | 0.00 | 0.00 | 0.00 | *Proteobacteria* | *Deltaproteobacteria* | *Myxococcales* | *Nannocystineae* | Transient-rare taxa |
| OTU 1125 | 0.01 | 0.00 | 0.00 | 0.00 | 0.03 | 0.00 | 0.00 | 0.00 | 0.00 | 0.00 | 0.00 | 0.00 | *Proteobacteria* | *Betaproteobacteria* | *Rhodocyclales* | *Rhodocyclaceae* | Transient-rare taxa |
| OTU 1127 | 0.01 | 0.02 | 0.00 | 0.00 | 0.00 | 0.00 | 0.00 | 0.01 | 0.00 | 0.00 | 0.00 | 0.00 | *Bacteroidetes* | *Sphingobacteriia* | *Sphingobacteriales* | *Chitinophagaceae* | Transient-rare taxa |
| OTU 1128 | 0.00 | 0.02 | 0.00 | 0.01 | 0.00 | 0.00 | 0.00 | 0.00 | 0.00 | 0.00 | 0.01 | 0.00 | *Proteobacteria* | *Betaproteobacteria* | *Rhodocyclales* | *Rhodocyclaceae* | Transient-rare taxa |
| OTU 1129 | 0.00 | 0.00 | 0.00 | 0.00 | 0.04 | 0.00 | 0.00 | 0.00 | 0.00 | 0.00 | 0.00 | 0.00 | *Bacteroidetes* | *Flavobacteriia* | *Flavobacteriales* | *Flavobacteriaceae* | Transient-rare taxa |
| OTU 1130 | 0.00 | 0.00 | 0.00 | 0.00 | 0.00 | 0.00 | 0.00 | 0.00 | 0.02 | 0.00 | 0.00 | 0.02 | *Proteobacteria* | *Alphaproteobacteria* | *Rhodobacterales* | *Rhodobacteraceae* | Transient-rare taxa |
| OTU 1131 | 0.00 | 0.02 | 0.00 | 0.00 | 0.00 | 0.00 | 0.00 | 0.00 | 0.01 | 0.00 | 0.01 | 0.00 | *Proteobacteria* | *Betaproteobacteria* | *Rhodocyclales* | *Rhodocyclaceae* | Transient-rare taxa |
| OTU 1132 | 0.00 | 0.04 | 0.00 | 0.00 | 0.00 | 0.00 | 0.00 | 0.00 | 0.00 | 0.00 | 0.00 | 0.00 | *Bacteroidetes* | *Cytophagia* | *Cytophagales* | *Flammeovirgaceae* | Transient-rare taxa |
| OTU 1133 | 0.00 | 0.03 | 0.00 | 0.00 | 0.00 | 0.00 | 0.00 | 0.00 | 0.01 | 0.00 | 0.00 | 0.00 | *Bacteroidetes* | *Sphingobacteriia* | *Sphingobacteriales* | *Saprospiraceae* | Transient-rare taxa |
| OTU 1134 | 0.00 | 0.00 | 0.00 | 0.00 | 0.04 | 0.00 | 0.00 | 0.00 | 0.00 | 0.00 | 0.00 | 0.00 | *Bacteroidetes* | *Flavobacteriia* | *Flavobacteriales* | *Cryomorphaceae* | Transient-rare taxa |
| OTU 1135 | 0.00 | 0.03 | 0.00 | 0.00 | 0.00 | 0.00 | 0.01 | 0.00 | 0.00 | 0.00 | 0.00 | 0.00 | *Proteobacteria* | *Gammaproteobacteria* | *Pseudomonadales* | *Pseudomonadaceae* | Transient-rare taxa |
| OTU 1136 | 0.00 | 0.03 | 0.00 | 0.00 | 0.00 | 0.00 | 0.00 | 0.00 | 0.01 | 0.00 | 0.00 | 0.00 | *Proteobacteria* | *Gammaproteobacteria* | *Xanthomonadales* | *Xanthomonadaceae* | Transient-rare taxa |
| OTU 1138 | 0.00 | 0.00 | 0.00 | 0.00 | 0.01 | 0.01 | 0.02 | 0.00 | 0.00 | 0.00 | 0.00 | 0.00 | *Bacteroidetes* | *Flavobacteriia* | *Flavobacteriales* | *Flavobacteriaceae* | Transient-rare taxa |
| OTU 1139 | 0.00 | 0.00 | 0.02 | 0.00 | 0.00 | 0.00 | 0.01 | 0.01 | 0.00 | 0.00 | 0.00 | 0.00 | *Proteobacteria* | *Gammaproteobacteria* | *Zhongshania* | | Transient-rare taxa |
| OTU 1140 | 0.00 | 0.00 | 0.00 | 0.00 | 0.00 | 0.00 | 0.00 | 0.00 | 0.00 | 0.02 | 0.02 | 0.00 | *Proteobacteria* | *Betaproteobacteria* | *Burkholderiales* | *Comamonadaceae* | Transient-rare taxa |
| OTU 1141 | 0.00 | 0.00 | 0.00 | 0.00 | 0.00 | 0.00 | 0.04 | 0.00 | 0.00 | 0.00 | 0.00 | 0.00 | *Proteobacteria* | *Betaproteobacteria* | *Rhodocyclales* | *Rhodocyclaceae* | Transient-rare taxa |
| OTU 1142 | 0.01 | 0.02 | 0.00 | 0.00 | 0.00 | 0.00 | 0.00 | 0.00 | 0.00 | 0.00 | 0.01 | 0.00 | *Proteobacteria* | *Alphaproteobacteria* | *Rhodospirillales* | *Acetobacteraceae* | Transient-rare taxa |
| OTU 1143 | 0.00 | 0.00 | 0.01 | 0.00 | 0.00 | 0.00 | 0.00 | 0.02 | 0.00 | 0.00 | 0.01 | 0.00 | *Proteobacteria* | *Betaproteobacteria* | *Rhodocyclales* | *Rhodocyclaceae* | Transient-rare taxa |
| OTU 1144 | 0.00 | 0.00 | 0.00 | 0.00 | 0.03 | 0.00 | 0.01 | 0.00 | 0.00 | 0.00 | 0.00 | 0.00 | *Bacteroidetes* | *Flavobacteriia* | *Flavobacteriales* | *Flavobacteriaceae* | Transient-rare taxa |
| OTU 1145 | 0.01 | 0.00 | 0.00 | 0.00 | 0.00 | 0.00 | 0.00 | 0.00 | 0.00 | 0.00 | 0.03 | 0.00 | *Proteobacteria* | *Deltaproteobacteria* | *Myxococcales* | *Nannocystineae* | Transient-rare taxa |
| OTU 1146 | 0.00 | 0.00 | 0.00 | 0.00 | 0.01 | 0.02 | 0.00 | 0.00 | 0.00 | 0.01 | 0.00 | 0.00 | *Firmicutes* | *Erysipelotrichia* | *Erysipelotrichales* | *Erysipelotrichaceae* | Transient-rare taxa |
| OTU 1147 | 0.00 | 0.00 | 0.00 | 0.00 | 0.02 | 0.00 | 0.00 | 0.02 | 0.00 | 0.00 | 0.00 | 0.00 | *Bacteroidetes* | *Sphingobacteriia* | *Sphingobacteriales* | *Saprospiraceae* | Transient-rare taxa |
| OTU 1148 | 0.02 | 0.01 | 0.01 | 0.00 | 0.00 | 0.00 | 0.00 | 0.00 | 0.00 | 0.00 | 0.00 | 0.00 | *Proteobacteria* | *Betaproteobacteria* | *Burkholderiales* | *Comamonadaceae* | Transient-rare taxa |
| OTU 1150 | 0.00 | 0.00 | 0.00 | 0.00 | 0.02 | 0.00 | 0.00 | 0.00 | 0.00 | 0.00 | 0.00 | 0.02 | *Bacteroidetes* | *Flavobacteriia* | *Flavobacteriales* | *Cryomorphaceae* | Transient-rare taxa |
| OTU 1151 | 0.00 | 0.00 | 0.00 | 0.00 | 0.02 | 0.01 | 0.01 | 0.00 | 0.00 | 0.00 | 0.00 | 0.00 | *Bacteroidetes* | *Flavobacteriia* | *Flavobacteriales* | *Flavobacteriaceae* | Transient-rare taxa |
| OTU 1152 | 0.00 | 0.03 | 0.01 | 0.00 | 0.00 | 0.00 | 0.00 | 0.00 | 0.00 | 0.00 | 0.00 | 0.00 | *Proteobacteria* | *Gammaproteobacteria* | *Chromatiales* | *Chromatiaceae* | Transient-rare taxa |
| OTU 1153 | 0.00 | 0.00 | 0.00 | 0.00 | 0.00 | 0.00 | 0.00 | 0.03 | 0.01 | 0.00 | 0.00 | 0.00 | *Bacteroidetes* | *Cytophagia* | *Cytophagales* | *Flammeovirgaceae* | Transient-rare taxa |
| OTU 1154 | 0.00 | 0.01 | 0.00 | 0.00 | 0.00 | 0.02 | 0.00 | 0.00 | 0.00 | 0.00 | 0.01 | 0.00 | *Actinobacteria* | *Actinobacteria* | *Coriobacteridae* | *Coriobacteriales* | Transient-rare taxa |
| OTU 1155 | 0.00 | 0.01 | 0.00 | 0.00 | 0.00 | 0.00 | 0.02 | 0.01 | 0.00 | 0.00 | 0.00 | 0.00 | *Proteobacteria* | *Betaproteobacteria* | *Burkholderiales* | *Burkholderiales_incertae_sedis* | Transient-rare taxa |
| OTU 1156 | 0.01 | 0.00 | 0.00 | 0.00 | 0.00 | 0.00 | 0.03 | 0.00 | 0.00 | 0.00 | 0.00 | 0.00 | *Proteobacteria* | *Gammaproteobacteria* | *Chromatiales* | *Chromatiaceae* | Transient-rare taxa |
| OTU 1157 | 0.02 | 0.00 | 0.00 | 0.00 | 0.00 | 0.00 | 0.00 | 0.00 | 0.00 | 0.01 | 0.01 | 0.00 | *Proteobacteria* | *Betaproteobacteria* | *Rhodocyclales* | *Rhodocyclaceae* | Transient-rare taxa |
| OTU 1158 | 0.00 | 0.00 | 0.00 | 0.00 | 0.00 | 0.01 | 0.00 | 0.02 | 0.00 | 0.00 | 0.01 | 0.00 | *Proteobacteria* | *Alphaproteobacteria* | *Rhodobacterales* | *Rhodobacteraceae* | Transient-rare taxa |
| OTU 1159 | 0.00 | 0.00 | 0.00 | 0.00 | 0.00 | 0.00 | 0.00 | 0.00 | 0.00 | 0.01 | 0.03 | 0.00 | *Bacteroidetes* | *Cytophagia* | *Cytophagales* | *Cytophagaceae* | Transient-rare taxa |
| OTU 1160 | 0.00 | 0.01 | 0.00 | 0.00 | 0.00 | 0.00 | 0.01 | 0.02 | 0.00 | 0.00 | 0.00 | 0.00 | *Proteobacteria* | *Deltaproteobacteria* | *Myxococcales* | *Sorangiineae* | Transient-rare taxa |
| OTU 1161 | 0.00 | 0.00 | 0.00 | 0.00 | 0.00 | 0.00 | 0.04 | 0.00 | 0.00 | 0.00 | 0.00 | 0.00 | *Proteobacteria* | *Alphaproteobacteria* | *Sphingomonadales* | *Sphingomonadaceae* | Transient-rare taxa |
| OTU 1163 | 0.00 | 0.00 | 0.00 | 0.00 | 0.00 | 0.00 | 0.00 | 0.00 | 0.02 | 0.00 | 0.02 | 0.00 | *Planctomycetes* | *Phycisphaerae* | *Phycisphaerales* | *Phycisphaeraceae* | Transient-rare taxa |
| OTU 1164 | 0.01 | 0.03 | 0.00 | 0.00 | 0.00 | 0.00 | 0.00 | 0.00 | 0.00 | 0.00 | 0.00 | 0.00 | *Proteobacteria* | *Gammaproteobacteria* | *Chromatiales* | *Chromatiaceae* | Transient-rare taxa |
| OTU 1165 | 0.00 | 0.00 | 0.00 | 0.01 | 0.03 | 0.00 | 0.00 | 0.00 | 0.00 | 0.00 | 0.00 | 0.00 | *Proteobacteria* | *Betaproteobacteria* | *Burkholderiales* | *Comamonadaceae* | Transient-rare taxa |
| OTU 1166 | 0.00 | 0.00 | 0.00 | 0.00 | 0.00 | 0.01 | 0.03 | 0.00 | 0.00 | 0.00 | 0.00 | 0.00 | *Proteobacteria* | *Alphaproteobacteria* | *Sphingomonadales* | *Erythrobacteraceae* | Transient-rare taxa |
| OTU 1167 | 0.00 | 0.00 | 0.00 | 0.00 | 0.00 | 0.00 | 0.00 | 0.03 | 0.01 | 0.00 | 0.00 | 0.00 | *Proteobacteria* | *Betaproteobacteria* | *Rhodocyclales* | *Rhodocyclaceae* | Transient-rare taxa |
| OTU 1169 | 0.00 | 0.00 | 0.00 | 0.01 | 0.00 | 0.01 | 0.00 | 0.00 | 0.02 | 0.00 | 0.00 | 0.00 | *Proteobacteria* | *Betaproteobacteria* | *Rhodocyclales* | *Rhodocyclaceae* | Transient-rare taxa |
| OTU 1170 | 0.01 | 0.00 | 0.00 | 0.00 | 0.00 | 0.00 | 0.01 | 0.00 | 0.02 | 0.00 | 0.00 | 0.00 | *Proteobacteria* | *Gammaproteobacteria* | *Xanthomonadales* | *Xanthomonadaceae* | Transient-rare taxa |
| OTU 1171 | 0.00 | 0.00 | 0.00 | 0.03 | 0.00 | 0.00 | 0.01 | 0.00 | 0.00 | 0.00 | 0.00 | 0.00 | *Bacteroidetes* | *Flavobacteriia* | *Flavobacteriales* | *Cryomorphaceae* | Transient-rare taxa |
| OTU 1172 | 0.00 | 0.04 | 0.00 | 0.00 | 0.00 | 0.00 | 0.00 | 0.00 | 0.00 | 0.00 | 0.00 | 0.00 | *Proteobacteria* | *Gammaproteobacteria* | *Xanthomonadales* | *Xanthomonadaceae* | Transient-rare taxa |
| OTU 1174 | 0.00 | 0.00 | 0.00 | 0.00 | 0.00 | 0.00 | 0.00 | 0.04 | 0.00 | 0.00 | 0.00 | 0.00 | *Proteobacteria* | *Deltaproteobacteria* | *Myxococcales* | *Nannocystineae* | Transient-rare taxa |
| OTU 1175 | 0.01 | 0.00 | 0.00 | 0.00 | 0.00 | 0.00 | 0.00 | 0.03 | 0.00 | 0.00 | 0.00 | 0.00 | *Proteobacteria* | *Betaproteobacteria* | *Neisseriales* | *Neisseriaceae* | Transient-rare taxa |
| OTU 1176 | 0.00 | 0.00 | 0.00 | 0.00 | 0.01 | 0.01 | 0.00 | 0.00 | 0.00 | 0.00 | 0.00 | 0.02 | *Proteobacteria* | *Alphaproteobacteria* | *Caulobacterales* | *Caulobacteraceae* | Transient-rare taxa |
| OTU 1177 | 0.00 | 0.00 | 0.00 | 0.02 | 0.02 | 0.00 | 0.00 | 0.00 | 0.00 | 0.00 | 0.00 | 0.00 | *Bacteroidetes* | *Flavobacteriia* | *Flavobacteriales* | *Flavobacteriaceae* | Transient-rare taxa |
| OTU 1178 | 0.00 | 0.00 | 0.00 | 0.00 | 0.02 | 0.00 | 0.00 | 0.02 | 0.00 | 0.00 | 0.00 | 0.00 | *Bacteroidetes* | *Bacteroidia* | *Bacteroidales* | *Porphyromonadaceae* | Transient-rare taxa |
| OTU 1179 | 0.00 | 0.02 | 0.00 | 0.00 | 0.00 | 0.00 | 0.01 | 0.01 | 0.00 | 0.00 | 0.00 | 0.00 | *Gemmatimonadetes* | *Gemmatimonadetes* | *Gemmatimonadales* | *Gemmatimonadaceae* | Transient-rare taxa |
| OTU 1181 | 0.00 | 0.00 | 0.02 | 0.01 | 0.00 | 0.00 | 0.00 | 0.01 | 0.00 | 0.00 | 0.00 | 0.00 | *Actinobacteria* | *Actinobacteria* | *Acidimicrobidae* | *Acidimicrobiales* | Transient-rare taxa |
| OTU 1182 | 0.00 | 0.04 | 0.00 | 0.00 | 0.00 | 0.00 | 0.00 | 0.00 | 0.00 | 0.00 | 0.00 | 0.00 | *Bacteroidetes* | *Cytophagia* | *Cytophagales* | *Flammeovirgaceae* | Transient-rare taxa |
| OTU 1183 | 0.00 | 0.00 | 0.02 | 0.00 | 0.01 | 0.00 | 0.01 | 0.00 | 0.00 | 0.00 | 0.00 | 0.00 | *Proteobacteria* | *Gammaproteobacteria* | *Alteromonadales* | *Alteromonadaceae* | Transient-rare taxa |
| OTU 1184 | 0.01 | 0.00 | 0.00 | 0.00 | 0.00 | 0.00 | 0.00 | 0.00 | 0.02 | 0.00 | 0.00 | 0.01 | *Proteobacteria* | *Alphaproteobacteria* | *Sphingomonadales* | *Sphingomonadaceae* | Transient-rare taxa |
| OTU 1185 | 0.02 | 0.00 | 0.00 | 0.00 | 0.00 | 0.00 | 0.01 | 0.00 | 0.01 | 0.00 | 0.00 | 0.00 | *Proteobacteria* | *Betaproteobacteria* | *Rhodocyclales* | *Rhodocyclaceae* | Transient-rare taxa |
| OTU 1186 | 0.00 | 0.00 | 0.00 | 0.00 | 0.00 | 0.00 | 0.00 | 0.00 | 0.03 | 0.01 | 0.00 | 0.00 | *Gemmatimonadetes* | *Gemmatimonadetes* | *Gemmatimonadales* | *Gemmatimonadaceae* | Transient-rare taxa |
| OTU 1187 | 0.03 | 0.01 | 0.00 | 0.00 | 0.00 | 0.00 | 0.00 | 0.00 | 0.00 | 0.00 | 0.00 | 0.00 | *Bacteroidetes* | *Sphingobacteriia* | *Sphingobacteriales* | *Cyclobacteriaceae* | Transient-rare taxa |
| OTU 1188 | 0.01 | 0.00 | 0.00 | 0.00 | 0.00 | 0.00 | 0.00 | 0.00 | 0.03 | 0.00 | 0.00 | 0.00 | *Proteobacteria* | *Alphaproteobacteria* | *Rhizobiales* | *Brucellaceae* | Transient-rare taxa |
| OTU 1189 | 0.01 | 0.00 | 0.00 | 0.00 | 0.00 | 0.00 | 0.00 | 0.00 | 0.02 | 0.01 | 0.00 | 0.00 | *Proteobacteria* | *Gammaproteobacteria* | *Xanthomonadales* | *Xanthomonadaceae* | Transient-rare taxa |
| OTU 1190 | 0.00 | 0.01 | 0.00 | 0.03 | 0.00 | 0.00 | 0.00 | 0.00 | 0.00 | 0.00 | 0.00 | 0.00 | *Bacteroidetes* | *Flavobacteriia* | *Flavobacteriales* | *Cryomorphaceae* | Transient-rare taxa |
| OTU 1191 | 0.03 | 0.01 | 0.00 | 0.00 | 0.00 | 0.00 | 0.00 | 0.00 | 0.00 | 0.00 | 0.00 | 0.00 | *Proteobacteria* | *Gammaproteobacteria* | *Chromatiales* | *Chromatiaceae* | Transient-rare taxa |
| OTU 1192 | 0.00 | 0.04 | 0.00 | 0.00 | 0.00 | 0.00 | 0.00 | 0.00 | 0.00 | 0.00 | 0.00 | 0.00 | *Planctomycetes* | *Planctomycetia* | *Planctomycetales* | *Planctomycetaceae* | Transient-rare taxa |
| OTU 1193 | 0.00 | 0.00 | 0.00 | 0.03 | 0.01 | 0.00 | 0.00 | 0.00 | 0.00 | 0.00 | 0.00 | 0.00 | *Bacteroidetes* | *Flavobacteriia* | *Flavobacteriales* | *Flavobacteriaceae* | Transient-rare taxa |
| OTU 1194 | 0.00 | 0.00 | 0.01 | 0.00 | 0.00 | 0.02 | 0.01 | 0.00 | 0.00 | 0.00 | 0.00 | 0.00 | *Proteobacteria* | *Alphaproteobacteria* | *Rhodospirillales* | *Rhodospirillaceae* | Transient-rare taxa |
| OTU 1195 | 0.00 | 0.01 | 0.00 | 0.00 | 0.00 | 0.02 | 0.01 | 0.00 | 0.00 | 0.00 | 0.00 | 0.00 | *Planctomycetes* | *Planctomycetia* | *Planctomycetales* | *Planctomycetaceae* | Transient-rare taxa |
| OTU 1196 | 0.00 | 0.00 | 0.00 | 0.00 | 0.02 | 0.00 | 0.02 | 0.00 | 0.00 | 0.00 | 0.00 | 0.00 | *Proteobacteria* | *Gammaproteobacteria* | *Chromatiales* | *Ectothiorhodospiraceae* | Transient-rare taxa |
| OTU 1197 | 0.00 | 0.00 | 0.02 | 0.00 | 0.01 | 0.01 | 0.00 | 0.00 | 0.00 | 0.00 | 0.00 | 0.00 | *Proteobacteria* | *Gammaproteobacteria* | *Alteromonadales* | *Alteromonadaceae* | Transient-rare taxa |
| OTU 1198 | 0.00 | 0.00 | 0.00 | 0.00 | 0.03 | 0.00 | 0.00 | 0.00 | 0.00 | 0.01 | 0.00 | 0.00 | *Bacteroidetes* | *Bacteroidia* | *Bacteroidales* | *Sunxiuqinia* | Transient-rare taxa |
| OTU 1199 | 0.00 | 0.00 | 0.00 | 0.00 | 0.01 | 0.02 | 0.01 | 0.00 | 0.00 | 0.00 | 0.00 | 0.00 | *Proteobacteria* | *Betaproteobacteria* | *Burkholderiales* | *Burkholderiales_incertae_sedis* | Transient-rare taxa |
| OTU 1200 | 0.00 | 0.00 | 0.00 | 0.00 | 0.04 | 0.00 | 0.00 | 0.00 | 0.00 | 0.00 | 0.00 | 0.00 | *Bacteroidetes* | *Sphingobacteriia* | *Sphingobacteriales* | *Saprospiraceae* | Transient-rare taxa |
| OTU 1203 | 0.02 | 0.02 | 0.00 | 0.00 | 0.00 | 0.00 | 0.00 | 0.00 | 0.00 | 0.00 | 0.00 | 0.00 | *Proteobacteria* | *Gammaproteobacteria* | *Chromatiales* | *Ectothiorhodospiraceae* | Transient-rare taxa |
| OTU 1205 | 0.00 | 0.00 | 0.02 | 0.00 | 0.00 | 0.01 | 0.00 | 0.01 | 0.00 | 0.00 | 0.00 | 0.00 | *Proteobacteria* | *Alphaproteobacteria* | *Sphingomonadales* | *Erythrobacteraceae* | Transient-rare taxa |
| OTU 1206 | 0.00 | 0.00 | 0.00 | 0.00 | 0.00 | 0.04 | 0.00 | 0.00 | 0.00 | 0.00 | 0.00 | 0.00 | *Actinobacteria* | *Actinobacteria* | *Actinobacteridae* | *Actinomycetales* | Transient-rare taxa |
| OTU 1207 | 0.00 | 0.00 | 0.00 | 0.00 | 0.00 | 0.04 | 0.00 | 0.00 | 0.00 | 0.00 | 0.00 | 0.00 | *Proteobacteria* | *Gammaproteobacteria* | *Oceanospirillales* | *Halomonadaceae* | Transient-rare taxa |
| OTU 1208 | 0.01 | 0.03 | 0.00 | 0.00 | 0.00 | 0.00 | 0.00 | 0.00 | 0.00 | 0.00 | 0.00 | 0.00 | *Proteobacteria* | *Betaproteobacteria* | *Neisseriales* | *Neisseriaceae* | Transient-rare taxa |
| OTU 1209 | 0.02 | 0.01 | 0.00 | 0.00 | 0.00 | 0.00 | 0.00 | 0.00 | 0.00 | 0.00 | 0.00 | 0.01 | *Proteobacteria* | *Betaproteobacteria* | *Rhodocyclales* | *Rhodocyclaceae* | Transient-rare taxa |
| OTU 1210 | 0.02 | 0.00 | 0.00 | 0.00 | 0.00 | 0.00 | 0.01 | 0.00 | 0.01 | 0.00 | 0.00 | 0.00 | *Bacteroidetes* | *Bacteroidia* | *Bacteroidales* | *Marinilabiliaceae* | Transient-rare taxa |
| OTU 1211 | 0.00 | 0.00 | 0.00 | 0.00 | 0.00 | 0.01 | 0.01 | 0.00 | 0.02 | 0.00 | 0.00 | 0.00 | *Proteobacteria* | *Betaproteobacteria* | *Rhodocyclales* | *Rhodocyclaceae* | Transient-rare taxa |
| OTU 1213 | 0.00 | 0.02 | 0.01 | 0.00 | 0.00 | 0.00 | 0.00 | 0.00 | 0.00 | 0.00 | 0.01 | 0.00 | *Proteobacteria* | *Betaproteobacteria* | *Rhodocyclales* | *Rhodocyclaceae* | Transient-rare taxa |
| OTU 1214 | 0.03 | 0.01 | 0.00 | 0.00 | 0.00 | 0.00 | 0.00 | 0.00 | 0.00 | 0.00 | 0.00 | 0.00 | *Proteobacteria* | *Gammaproteobacteria* | *Chromatiales* | *Chromatiaceae* | Transient-rare taxa |
| OTU 1215 | 0.00 | 0.01 | 0.00 | 0.00 | 0.00 | 0.00 | 0.00 | 0.00 | 0.02 | 0.01 | 0.00 | 0.00 | *Planctomycetes* | *Planctomycetia* | *Planctomycetales* | *Planctomycetaceae* | Transient-rare taxa |
| OTU 1216 | 0.00 | 0.01 | 0.02 | 0.00 | 0.00 | 0.00 | 0.00 | 0.01 | 0.00 | 0.00 | 0.00 | 0.00 | *Planctomycetes* | *Planctomycetia* | *Planctomycetales* | *Planctomycetaceae* | Transient-rare taxa |
| OTU 1217 | 0.00 | 0.00 | 0.00 | 0.00 | 0.00 | 0.00 | 0.00 | 0.00 | 0.00 | 0.00 | 0.04 | 0.00 | *Proteobacteria* | *Alphaproteobacteria* | *Sphingomonadales* | *Sphingomonadaceae* | Transient-rare taxa |
| OTU 1218 | 0.00 | 0.00 | 0.00 | 0.00 | 0.00 | 0.00 | 0.00 | 0.00 | 0.00 | 0.00 | 0.04 | 0.00 | *Chlorobi* | *Chlorobia* | *Chlorobiales* | *Chlorobiaceae* | Transient-rare taxa |
| OTU 1219 | 0.00 | 0.00 | 0.01 | 0.00 | 0.00 | 0.00 | 0.00 | 0.00 | 0.01 | 0.00 | 0.02 | 0.00 | *Actinobacteria* | *Actinobacteria* | *Acidimicrobidae* | *Acidimicrobiales* | Transient-rare taxa |
| OTU 1220 | 0.03 | 0.00 | 0.00 | 0.01 | 0.00 | 0.00 | 0.00 | 0.00 | 0.00 | 0.00 | 0.00 | 0.00 | *Bacteroidetes* | *Sphingobacteriia* | *Sphingobacteriales* | *Saprospiraceae* | Transient-rare taxa |
| OTU 1221 | 0.04 | 0.00 | 0.00 | 0.00 | 0.00 | 0.00 | 0.00 | 0.00 | 0.00 | 0.00 | 0.00 | 0.00 | *Bacteroidetes* | *Sphingobacteriia* | *Sphingobacteriales* | *Saprospiraceae* | Transient-rare taxa |
| OTU 1223 | 0.02 | 0.00 | 0.00 | 0.00 | 0.00 | 0.00 | 0.00 | 0.00 | 0.00 | 0.02 | 0.00 | 0.00 | *Thermodesulfobacteria* | *Thermodesulfobacteria* | *Thermodesulfobacteriales* | *Thermodesulfobacteriaceae* | Transient-rare taxa |
| OTU 1224 | 0.01 | 0.00 | 0.00 | 0.00 | 0.00 | 0.00 | 0.00 | 0.00 | 0.00 | 0.00 | 0.01 | 0.02 | *Gemmatimonadetes* | *Gemmatimonadetes* | *Gemmatimonadales* | *Gemmatimonadaceae* | Transient-rare taxa |
| OTU 1225 | 0.00 | 0.00 | 0.03 | 0.01 | 0.00 | 0.00 | 0.00 | 0.00 | 0.00 | 0.00 | 0.00 | 0.00 | *Bacteroidetes* | *Flavobacteriia* | *Flavobacteriales* | *Flavobacteriaceae* | Transient-rare taxa |
| OTU 1226 | 0.03 | 0.01 | 0.00 | 0.00 | 0.00 | 0.00 | 0.00 | 0.00 | 0.00 | 0.00 | 0.00 | 0.00 | *Bacteroidetes* | *Sphingobacteriia* | *Sphingobacteriales* | *Saprospiraceae* | Transient-rare taxa |
| OTU 1227 | 0.02 | 0.00 | 0.00 | 0.00 | 0.01 | 0.01 | 0.00 | 0.00 | 0.00 | 0.00 | 0.00 | 0.00 | *Proteobacteria* | *Betaproteobacteria* | *Burkholderiales* | *Burkholderiaceae* | Transient-rare taxa |
| OTU 1228 | 0.04 | 0.00 | 0.00 | 0.00 | 0.00 | 0.00 | 0.00 | 0.00 | 0.00 | 0.00 | 0.00 | 0.00 | *Bacteroidetes* | *Sphingobacteriia* | *Sphingobacteriales* | *Chitinophagaceae* | Transient-rare taxa |
| OTU 1229 | 0.03 | 0.00 | 0.00 | 0.00 | 0.00 | 0.00 | 0.00 | 0.00 | 0.00 | 0.01 | 0.00 | 0.00 | *Proteobacteria* | *Betaproteobacteria* | *Rhodocyclales* | *Rhodocyclaceae* | Transient-rare taxa |
| OTU 1230 | 0.03 | 0.00 | 0.00 | 0.00 | 0.00 | 0.00 | 0.00 | 0.00 | 0.00 | 0.00 | 0.01 | 0.00 | *Proteobacteria* | *Gammaproteobacteria* | *Methylococcales* | *Methylococcaceae* | Transient-rare taxa |
| OTU 1231 | 0.00 | 0.00 | 0.00 | 0.04 | 0.00 | 0.00 | 0.00 | 0.00 | 0.00 | 0.00 | 0.00 | 0.00 | *Proteobacteria* | *Gammaproteobacteria* | *Thiotrichales* | *Francisellaceae* | Transient-rare taxa |
| OTU 1232 | 0.00 | 0.00 | 0.01 | 0.00 | 0.00 | 0.00 | 0.02 | 0.00 | 0.00 | 0.00 | 0.00 | 0.01 | *Proteobacteria* | *Alphaproteobacteria* | *Rhizobiales* | *Hyphomicrobiaceae* | Transient-rare taxa |
| OTU 1233 | 0.00 | 0.02 | 0.01 | 0.00 | 0.00 | 0.00 | 0.00 | 0.01 | 0.00 | 0.00 | 0.00 | 0.00 | *Planctomycetes* | *Planctomycetia* | *Planctomycetales* | *Planctomycetaceae* | Transient-rare taxa |
| OTU 1234 | 0.00 | 0.02 | 0.00 | 0.00 | 0.00 | 0.00 | 0.00 | 0.01 | 0.00 | 0.00 | 0.00 | 0.01 | *Proteobacteria* | *Betaproteobacteria* | *Rhodocyclales* | *Rhodocyclaceae* | Transient-rare taxa |
| OTU 1235 | 0.00 | 0.01 | 0.00 | 0.00 | 0.00 | 0.00 | 0.00 | 0.00 | 0.00 | 0.02 | 0.01 | 0.00 | *Bacteroidetes* | *Sphingobacteriia* | *Sphingobacteriales* | *Sphingobacteriaceae* | Transient-rare taxa |
| OTU 1236 | 0.00 | 0.00 | 0.01 | 0.00 | 0.00 | 0.00 | 0.00 | 0.00 | 0.00 | 0.02 | 0.00 | 0.01 | *Bacteroidetes* | *Flavobacteriia* | *Flavobacteriales* | *Cryomorphaceae* | Transient-rare taxa |
| OTU 1237 | 0.00 | 0.00 | 0.00 | 0.04 | 0.00 | 0.00 | 0.00 | 0.00 | 0.00 | 0.00 | 0.00 | 0.00 | *Bacteroidetes* | *Cytophagia* | *Cytophagales* | *Cytophagaceae* | Transient-rare taxa |
| OTU 1238 | 0.00 | 0.00 | 0.00 | 0.00 | 0.00 | 0.00 | 0.00 | 0.00 | 0.00 | 0.00 | 0.00 | 0.04 | *Proteobacteria* | *Deltaproteobacteria* | *Myxococcales* | *Nannocystineae* | Transient-rare taxa |
| OTU 1239 | 0.00 | 0.00 | 0.01 | 0.00 | 0.00 | 0.02 | 0.00 | 0.00 | 0.00 | 0.00 | 0.00 | 0.01 | *Proteobacteria* | *Betaproteobacteria* | *Rhodocyclales* | *Rhodocyclaceae* | Transient-rare taxa |
| OTU 1240 | 0.00 | 0.00 | 0.01 | 0.00 | 0.00 | 0.02 | 0.01 | 0.00 | 0.00 | 0.00 | 0.00 | 0.00 | *Proteobacteria* | *Betaproteobacteria* | *Rhodocyclales* | *Rhodocyclaceae* | Transient-rare taxa |
| OTU 1241 | 0.03 | 0.00 | 0.00 | 0.00 | 0.00 | 0.00 | 0.00 | 0.00 | 0.00 | 0.01 | 0.00 | 0.00 | *Bacteroidetes* | *Flavobacteriia* | *Flavobacteriales* | *Cryomorphaceae* | Transient-rare taxa |
| OTU 1242 | 0.02 | 0.00 | 0.00 | 0.00 | 0.00 | 0.02 | 0.00 | 0.00 | 0.00 | 0.00 | 0.00 | 0.00 | *Proteobacteria* | *Gammaproteobacteria* | *Oceanospirillales* | *Hahellaceae* | Transient-rare taxa |
| OTU 1243 | 0.00 | 0.01 | 0.00 | 0.00 | 0.00 | 0.00 | 0.00 | 0.01 | 0.00 | 0.02 | 0.00 | 0.00 | *Planctomycetes* | *Planctomycetia* | *Planctomycetales* | *Planctomycetaceae* | Transient-rare taxa |
| OTU 1244 | 0.03 | 0.00 | 0.00 | 0.00 | 0.00 | 0.00 | 0.01 | 0.00 | 0.00 | 0.00 | 0.00 | 0.00 | *Acidobacteria* | *Acidobacteria_Gp1* | *Acidobacterium* | | Transient-rare taxa |
| OTU 1246 | 0.02 | 0.00 | 0.00 | 0.00 | 0.00 | 0.00 | 0.00 | 0.00 | 0.00 | 0.00 | 0.00 | 0.02 | *Bacteroidetes* | *Flavobacteriia* | *Flavobacteriales* | *Flavobacteriaceae* | Transient-rare taxa |
| OTU 1247 | 0.01 | 0.00 | 0.00 | 0.00 | 0.00 | 0.00 | 0.00 | 0.00 | 0.00 | 0.00 | 0.00 | 0.03 | *Bacteroidetes* | *Sphingobacteriia* | *Sphingobacteriales* | *Saprospiraceae* | Transient-rare taxa |
| OTU 1249 | 0.01 | 0.03 | 0.00 | 0.00 | 0.00 | 0.00 | 0.00 | 0.00 | 0.00 | 0.00 | 0.00 | 0.00 | *Proteobacteria* | *Betaproteobacteria* | *Burkholderiales* | *Comamonadaceae* | Transient-rare taxa |
| OTU 1250 | 0.01 | 0.03 | 0.00 | 0.00 | 0.00 | 0.00 | 0.00 | 0.00 | 0.00 | 0.00 | 0.00 | 0.00 | *Proteobacteria* | *Gammaproteobacteria* | *Chromatiales* | *Chromatiaceae* | Transient-rare taxa |
| OTU 1251 | 0.01 | 0.00 | 0.01 | 0.00 | 0.00 | 0.00 | 0.00 | 0.00 | 0.00 | 0.00 | 0.00 | 0.02 | *Actinobacteria* | *Actinobacteria* | *Actinobacteridae* | *Actinomycetales* | Transient-rare taxa |
| OTU 1252 | 0.00 | 0.00 | 0.00 | 0.00 | 0.00 | 0.00 | 0.00 | 0.00 | 0.00 | 0.00 | 0.02 | 0.02 | *Firmicutes* | *Bacilli* | *Bacillales* | *Bacillaceae 1* | Transient-rare taxa |
| OTU 1253 | 0.02 | 0.00 | 0.00 | 0.01 | 0.00 | 0.00 | 0.00 | 0.00 | 0.00 | 0.01 | 0.00 | 0.00 | *Proteobacteria* | *Gammaproteobacteria* | *Pseudomonadales* | *Moraxellaceae* | Transient-rare taxa |
| OTU 1254 | 0.00 | 0.00 | 0.00 | 0.00 | 0.00 | 0.00 | 0.00 | 0.00 | 0.04 | 0.00 | 0.00 | 0.00 | *Firmicutes* | *Clostridia* | *Clostridiales* | *Ruminococcaceae* | Transient-rare taxa |
| OTU 1255 | 0.00 | 0.00 | 0.00 | 0.00 | 0.00 | 0.01 | 0.00 | 0.00 | 0.02 | 0.00 | 0.01 | 0.00 | *Proteobacteria* | *Gammaproteobacteria* | *Xanthomonadales* | *Xanthomonadaceae* | Transient-rare taxa |
| OTU 1256 | 0.00 | 0.00 | 0.01 | 0.00 | 0.00 | 0.00 | 0.00 | 0.00 | 0.00 | 0.01 | 0.00 | 0.02 | *Spirochaetes* | *Spirochaetia* | *Spirochaetales* | *Spirochaetaceae* | Transient-rare taxa |
| OTU 1257 | 0.00 | 0.00 | 0.00 | 0.00 | 0.00 | 0.00 | 0.02 | 0.02 | 0.00 | 0.00 | 0.00 | 0.00 | *Gemmatimonadetes* | *Gemmatimonadetes* | *Gemmatimonadales* | *Gemmatimonadaceae* | Transient-rare taxa |
| OTU 1258 | 0.03 | 0.00 | 0.00 | 0.00 | 0.00 | 0.00 | 0.00 | 0.00 | 0.00 | 0.00 | 0.00 | 0.01 | *Proteobacteria* | *Betaproteobacteria* | *Burkholderiales* | *Comamonadaceae* | Transient-rare taxa |
| OTU 1259 | 0.01 | 0.00 | 0.00 | 0.00 | 0.00 | 0.00 | 0.00 | 0.00 | 0.01 | 0.02 | 0.00 | 0.00 | *Bacteroidetes* | *Flavobacteriia* | *Flavobacteriales* | *Cryomorphaceae* | Transient-rare taxa |
| OTU 1260 | 0.00 | 0.00 | 0.00 | 0.00 | 0.00 | 0.00 | 0.00 | 0.01 | 0.00 | 0.02 | 0.00 | 0.01 | *Bacteroidetes* | *Bacteroidia* | *Bacteroidales* | *Porphyromonadaceae* | Transient-rare taxa |
| OTU 1261 | 0.02 | 0.00 | 0.00 | 0.00 | 0.00 | 0.00 | 0.00 | 0.00 | 0.00 | 0.02 | 0.00 | 0.00 | *Proteobacteria* | *Gammaproteobacteria* | *Gammaproteobacteria_incertae_sedis* | *Umboniibacter* | Transient-rare taxa |

| | | | | | | | | | | | | | | | | | |
|---|---|---|---|---|---|---|---|---|---|---|---|---|---|---|---|---|---|
| OTU 1263 | 0.00 | 0.00 | 0.00 | 0.00 | 0.00 | 0.00 | 0.00 | 0.00 | 0.00 | 0.04 | 0.00 | 0.00 | Bacteroidetes | Sphingobacteriia | Sphingobacteriales | Sphingobacteriaceae | Transient-rare taxa |
| OTU 1264 | 0.00 | 0.00 | 0.00 | 0.00 | 0.00 | 0.00 | 0.00 | 0.00 | 0.00 | 0.00 | 0.00 | 0.04 | Proteobacteria | Betaproteobacteria | Rhodocyclales | Rhodocyclaceae | Transient-rare taxa |
| OTU 1265 | 0.02 | 0.00 | 0.00 | 0.00 | 0.00 | 0.00 | 0.00 | 0.00 | 0.00 | 0.00 | 0.00 | 0.02 | Proteobacteria | Alphaproteobacteria | Rhodobacterales | Rhodobacteraceae | Transient-rare taxa |
| OTU 1267 | 0.00 | 0.00 | 0.00 | 0.00 | 0.00 | 0.00 | 0.00 | 0.00 | 0.04 | 0.00 | 0.00 | 0.00 | Bacteroidetes | Sphingobacteriia | Sphingobacteriales | Saprospiraceae | Transient-rare taxa |
| OTU 1269 | 0.00 | 0.00 | 0.04 | 0.00 | 0.00 | 0.00 | 0.00 | 0.00 | 0.00 | 0.00 | 0.00 | 0.00 | Chloroflexi | Caldilineae | Caldilineales | Caldilineaceae | Transient-rare taxa |
| OTU 1270 | 0.00 | 0.00 | 0.00 | 0.00 | 0.00 | 0.02 | 0.00 | 0.00 | 0.00 | 0.00 | 0.01 | 0.01 | Actinobacteria | Actinobacteria | Actinobacteridae | Actinomycetales | Transient-rare taxa |
| OTU 1273 | 0.00 | 0.00 | 0.00 | 0.00 | 0.00 | 0.00 | 0.00 | 0.00 | 0.00 | 0.00 | 0.00 | 0.04 | Spirochaetes | Spirochaetia | Spirochaetales | Spirochaetaceae | Transient-rare taxa |
| OTU 1274 | 0.00 | 0.00 | 0.01 | 0.00 | 0.00 | 0.00 | 0.00 | 0.00 | 0.01 | 0.00 | 0.02 | 0.00 | Proteobacteria | Betaproteobacteria | Rhodocyclales | Rhodocyclaceae | Transient-rare taxa |
| OTU 1275 | 0.00 | 0.00 | 0.03 | 0.00 | 0.00 | 0.00 | 0.00 | 0.00 | 0.01 | 0.00 | 0.00 | 0.00 | Proteobacteria | Deltaproteobacteria | Myxococcales | Sorangiineae | Transient-rare taxa |
| OTU 1276 | 0.00 | 0.01 | 0.02 | 0.00 | 0.00 | 0.00 | 0.00 | 0.00 | 0.01 | 0.00 | 0.00 | 0.00 | Proteobacteria | Betaproteobacteria | Ferrovales | Ferrovaceae | Transient-rare taxa |
| OTU 1277 | 0.00 | 0.00 | 0.02 | 0.00 | 0.00 | 0.00 | 0.00 | 0.00 | 0.01 | 0.01 | 0.00 | 0.00 | Proteobacteria | Alphaproteobacteria | Sphingomonadales | Sphingomonadaceae | Transient-rare taxa |
| OTU 1278 | 0.00 | 0.00 | 0.01 | 0.00 | 0.00 | 0.00 | 0.00 | 0.00 | 0.01 | 0.00 | 0.02 | 0.00 | Proteobacteria | Betaproteobacteria | Rhodocyclales | Rhodocyclaceae | Transient-rare taxa |
| OTU 1279 | 0.00 | 0.00 | 0.00 | 0.00 | 0.00 | 0.00 | 0.00 | 0.00 | 0.01 | 0.03 | 0.00 | 0.00 | Actinobacteria | Actinobacteria | Acidimicrobidae | Acidimicrobiales | Transient-rare taxa |
| OTU 1280 | 0.00 | 0.00 | 0.04 | 0.00 | 0.00 | 0.00 | 0.00 | 0.00 | 0.00 | 0.00 | 0.00 | 0.00 | Proteobacteria | Betaproteobacteria | Hydrogenophilales | Hydrogenophilaceae | Transient-rare taxa |
| OTU 1281 | 0.00 | 0.00 | 0.00 | 0.00 | 0.00 | 0.00 | 0.00 | 0.00 | 0.01 | 0.02 | 0.01 | 0.00 | Proteobacteria | Betaproteobacteria | Rhodocyclales | Rhodocyclaceae | Transient-rare taxa |
| OTU 1282 | 0.00 | 0.00 | 0.02 | 0.01 | 0.00 | 0.00 | 0.00 | 0.01 | 0.00 | 0.00 | 0.00 | 0.00 | Proteobacteria | Betaproteobacteria | Ferrovales | Ferrovaceae | Transient-rare taxa |
| OTU 1283 | 0.00 | 0.00 | 0.02 | 0.02 | 0.00 | 0.00 | 0.00 | 0.00 | 0.00 | 0.00 | 0.00 | 0.00 | Proteobacteria | Gammaproteobacteria | Gammaproteobacteria_incertae_sedis | Simiduia | Transient-rare taxa |
| OTU 1285 | 0.00 | 0.00 | 0.00 | 0.00 | 0.00 | 0.00 | 0.00 | 0.01 | 0.00 | 0.00 | 0.01 | 0.02 | Proteobacteria | Gammaproteobacteria | Xanthomonadales | Xanthomonadaceae | Transient-rare taxa |
| OTU 1286 | 0.01 | 0.00 | 0.00 | 0.00 | 0.00 | 0.00 | 0.00 | 0.00 | 0.00 | 0.00 | 0.02 | 0.01 | Bacteroidetes | Sphingobacteriia | Sphingobacteriales | Chitinophagaceae | Transient-rare taxa |
| OTU 1287 | 0.00 | 0.00 | 0.00 | 0.00 | 0.01 | 0.01 | 0.00 | 0.00 | 0.00 | 0.00 | 0.02 | 0.00 | Proteobacteria | Betaproteobacteria | Burkholderiales | Comamonadaceae | Transient-rare taxa |
| OTU 1288 | 0.00 | 0.00 | 0.00 | 0.00 | 0.00 | 0.00 | 0.00 | 0.00 | 0.00 | 0.00 | 0.03 | 0.01 | Proteobacteria | Gammaproteobacteria | Xanthomonadales | Xanthomonadaceae | Transient-rare taxa |
| OTU 1290 | 0.00 | 0.00 | 0.00 | 0.00 | 0.00 | 0.00 | 0.00 | 0.00 | 0.00 | 0.00 | 0.04 | 0.00 | Proteobacteria | Deltaproteobacteria | Myxococcales | Nannocystineae | Transient-rare taxa |
| OTU 1291 | 0.00 | 0.00 | 0.00 | 0.00 | 0.00 | 0.00 | 0.00 | 0.00 | 0.00 | 0.00 | 0.02 | 0.02 | Bacteroidetes | Sphingobacteriia | Sphingobacteriales | Chitinophagaceae | Transient-rare taxa |
| OTU 1292 | 0.00 | 0.00 | 0.00 | 0.01 | 0.00 | 0.02 | 0.00 | 0.00 | 0.00 | 0.00 | 0.01 | 0.00 | Proteobacteria | Betaproteobacteria | Burkholderiales | Burkholderiales_incertae_sedis | Transient-rare taxa |
| OTU 1293 | 0.00 | 0.00 | 0.01 | 0.00 | 0.00 | 0.00 | 0.00 | 0.00 | 0.00 | 0.00 | 0.03 | 0.00 | Proteobacteria | Alphaproteobacteria | Rhodobacterales | Rhodobacteraceae | Transient-rare taxa |
| OTU 1294 | 0.01 | 0.00 | 0.00 | 0.00 | 0.00 | 0.00 | 0.00 | 0.00 | 0.00 | 0.00 | 0.03 | 0.00 | Proteobacteria | Deltaproteobacteria | Myxococcales | Nannocystineae | Transient-rare taxa |
| OTU 1295 | 0.01 | 0.00 | 0.00 | 0.00 | 0.01 | 0.02 | 0.00 | 0.00 | 0.00 | 0.00 | 0.00 | 0.00 | Proteobacteria | Alphaproteobacteria | Rhizobiales | Hyphomicrobiaceae | Transient-rare taxa |
| OTU 1296 | 0.00 | 0.00 | 0.01 | 0.02 | 0.00 | 0.00 | 0.00 | 0.01 | 0.00 | 0.00 | 0.00 | 0.00 | Actinobacteria | Actinobacteria | Actinobacteridae | Actinomycetales | Transient-rare taxa |
| OTU 1297 | 0.01 | 0.00 | 0.00 | 0.00 | 0.00 | 0.00 | 0.00 | 0.00 | 0.00 | 0.00 | 0.03 | 0.00 | Proteobacteria | Gammaproteobacteria | Pseudomonadales | Moraxellaceae | Transient-rare taxa |
| OTU 1298 | 0.00 | 0.00 | 0.01 | 0.00 | 0.00 | 0.00 | 0.00 | 0.03 | 0.00 | 0.00 | 0.00 | 0.00 | Proteobacteria | Deltaproteobacteria | Myxococcales | Nannocystineae | Transient-rare taxa |
| OTU 1299 | 0.00 | 0.00 | 0.00 | 0.00 | 0.00 | 0.00 | 0.00 | 0.00 | 0.00 | 0.00 | 0.02 | 0.02 | Proteobacteria | Alphaproteobacteria | Rhodospirillales | Rhodospirillaceae | Transient-rare taxa |
| OTU 1300 | 0.00 | 0.00 | 0.00 | 0.00 | 0.03 | 0.00 | 0.00 | 0.00 | 0.00 | 0.00 | 0.01 | 0.00 | Proteobacteria | Betaproteobacteria | Burkholderiales | Burkholderiaceae | Transient-rare taxa |
| OTU 1301 | 0.00 | 0.00 | 0.00 | 0.00 | 0.00 | 0.00 | 0.00 | 0.00 | 0.00 | 0.00 | 0.04 | 0.00 | Proteobacteria | Alphaproteobacteria | Sphingomonadales | Erythrobacteraceae | Transient-rare taxa |
| OTU 1302 | 0.00 | 0.00 | 0.00 | 0.00 | 0.00 | 0.00 | 0.00 | 0.00 | 0.00 | 0.00 | 0.04 | 0.00 | Proteobacteria | Betaproteobacteria | Rhodocyclales | Rhodocyclaceae | Transient-rare taxa |
| OTU 1303 | 0.00 | 0.00 | 0.00 | 0.00 | 0.00 | 0.00 | 0.00 | 0.00 | 0.00 | 0.00 | 0.04 | 0.00 | Proteobacteria | Deltaproteobacteria | Myxococcales | Nannocystineae | Transient-rare taxa |
| OTU 1304 | 0.00 | 0.00 | 0.00 | 0.00 | 0.00 | 0.00 | 0.00 | 0.00 | 0.00 | 0.00 | 0.04 | 0.00 | Proteobacteria | Alphaproteobacteria | Rhizobiales | Rhodobiaceae | Transient-rare taxa |
| OTU 1305 | 0.00 | 0.00 | 0.00 | 0.00 | 0.00 | 0.00 | 0.00 | 0.00 | 0.00 | 0.00 | 0.04 | 0.00 | Proteobacteria | Betaproteobacteria | Burkholderiales | Burkholderiales_incertae_sedis | Transient-rare taxa |
| OTU 1306 | 0.00 | 0.00 | 0.00 | 0.00 | 0.00 | 0.00 | 0.00 | 0.00 | 0.00 | 0.04 | 0.00 | 0.00 | Marinimicrobia | Marinimicrobia_genera_incertae_sedis | | | Transient-rare taxa |
| OTU 1307 | 0.00 | 0.00 | 0.00 | 0.01 | 0.00 | 0.00 | 0.00 | 0.00 | 0.02 | 0.01 | 0.00 | 0.00 | Proteobacteria | Alphaproteobacteria | Sphingomonadales | Erythrobacteraceae | Transient-rare taxa |
| OTU 1308 | 0.00 | 0.00 | 0.00 | 0.00 | 0.00 | 0.00 | 0.00 | 0.00 | 0.00 | 0.00 | 0.03 | 0.01 | Proteobacteria | Gammaproteobacteria | Oceanospirillales | Halomonadaceae | Transient-rare taxa |
| OTU 1309 | 0.00 | 0.00 | 0.00 | 0.00 | 0.00 | 0.00 | 0.00 | 0.00 | 0.02 | 0.00 | 0.00 | 0.02 | Acidobacteria | Acidobacteria_Gp17 | Gp17 | | Transient-rare taxa |
| OTU 1310 | 0.00 | 0.04 | 0.00 | 0.00 | 0.00 | 0.00 | 0.00 | 0.00 | 0.00 | 0.00 | 0.00 | 0.00 | Bacteroidetes | Sphingobacteriia | Sphingobacteriales | Saprospiraceae | Transient-rare taxa |
| OTU 1311 | 0.00 | 0.03 | 0.00 | 0.00 | 0.00 | 0.00 | 0.00 | 0.00 | 0.01 | 0.00 | 0.00 | 0.00 | Proteobacteria | Gammaproteobacteria | Xanthomonadales | Xanthomonadaceae | Transient-rare taxa |
| OTU 1312 | 0.00 | 0.01 | 0.00 | 0.00 | 0.00 | 0.00 | 0.00 | 0.00 | 0.00 | 0.00 | 0.02 | 0.01 | Proteobacteria | Betaproteobacteria | Nitrosomonadales | Nitrosomonadaceae | Transient-rare taxa |
| OTU 1313 | 0.02 | 0.00 | 0.00 | 0.00 | 0.00 | 0.00 | 0.00 | 0.00 | 0.00 | 0.01 | 0.00 | 0.01 | Bacteroidetes | Cytophagia | Cytophagales | Cytophagaceae | Transient-rare taxa |
| OTU 1314 | 0.00 | 0.00 | 0.00 | 0.00 | 0.00 | 0.00 | 0.02 | 0.02 | 0.00 | 0.00 | 0.00 | 0.00 | Planctomycetes | Planctomycetia | Planctomycetales | Planctomycetaceae | Transient-rare taxa |
| OTU 1315 | 0.00 | 0.00 | 0.00 | 0.00 | 0.01 | 0.00 | 0.00 | 0.01 | 0.02 | 0.00 | 0.00 | 0.00 | Proteobacteria | Gammaproteobacteria | Chromatiales | Ectothiorhodospiraceae | Transient-rare taxa |
| OTU 1316 | 0.00 | 0.00 | 0.00 | 0.00 | 0.00 | 0.00 | 0.00 | 0.04 | 0.00 | 0.00 | 0.00 | 0.00 | Proteobacteria | Betaproteobacteria | Ferrovales | Ferrovaceae | Transient-rare taxa |
| OTU 1317 | 0.00 | 0.00 | 0.00 | 0.03 | 0.00 | 0.01 | 0.00 | 0.00 | 0.00 | 0.00 | 0.00 | 0.00 | Firmicutes | Erysipelotrichia | Erysipelotrichales | Erysipelotrichaceae | Transient-rare taxa |
| OTU 1318 | 0.00 | 0.00 | 0.00 | 0.00 | 0.00 | 0.01 | 0.03 | 0.00 | 0.00 | 0.00 | 0.00 | 0.00 | Proteobacteria | Alphaproteobacteria | Rhodobacterales | Rhodobacteraceae | Transient-rare taxa |
| OTU 1319 | 0.00 | 0.00 | 0.00 | 0.00 | 0.00 | 0.00 | 0.00 | 0.00 | 0.00 | 0.00 | 0.00 | 0.04 | Proteobacteria | Alphaproteobacteria | Sphingomonadales | Sphingomonadaceae | Transient-rare taxa |
| OTU 1321 | 0.00 | 0.00 | 0.00 | 0.00 | 0.01 | 0.00 | 0.00 | 0.00 | 0.00 | 0.00 | 0.00 | 0.03 | Proteobacteria | Alphaproteobacteria | Rickettsiales | Anaplasmataceae | Transient-rare taxa |
| OTU 1322 | 0.00 | 0.00 | 0.00 | 0.00 | 0.00 | 0.04 | 0.00 | 0.00 | 0.00 | 0.00 | 0.00 | 0.00 | Proteobacteria | Alphaproteobacteria | Rhizobiales | Rhizobiales_incertae_sedis | Transient-rare taxa |
| OTU 1323 | 0.00 | 0.00 | 0.00 | 0.00 | 0.04 | 0.00 | 0.00 | 0.00 | 0.00 | 0.00 | 0.00 | 0.00 | Proteobacteria | Alphaproteobacteria | Rhizobiales | Hyphomicrobiaceae | Transient-rare taxa |
| OTU 1325 | 0.00 | 0.00 | 0.04 | 0.00 | 0.00 | 0.00 | 0.00 | 0.00 | 0.00 | 0.00 | 0.00 | 0.00 | Bacteroidetes | Bacteroidia | Bacteroidales | Marinilabiliaceae | Transient-rare taxa |
| OTU 1326 | 0.00 | 0.00 | 0.00 | 0.00 | 0.00 | 0.04 | 0.00 | 0.00 | 0.00 | 0.00 | 0.00 | 0.00 | Proteobacteria | Alphaproteobacteria | Rhodobacterales | Rhodobacteraceae | Transient-rare taxa |
| OTU 1327 | 0.03 | 0.00 | 0.00 | 0.00 | 0.00 | 0.00 | 0.00 | 0.00 | 0.00 | 0.00 | 0.00 | 0.01 | Bacteroidetes | Sphingobacteriia | Sphingobacteriales | Cyclobacteriaceae | Transient-rare taxa |
| OTU 1328 | 0.00 | 0.00 | 0.04 | 0.00 | 0.00 | 0.00 | 0.00 | 0.00 | 0.00 | 0.00 | 0.00 | 0.00 | Proteobacteria | Gammaproteobacteria | Gammaproteobacteria_incertae_sedis | Thiohalophilus | Transient-rare taxa |
| OTU 1329 | 0.00 | 0.00 | 0.00 | 0.00 | 0.00 | 0.04 | 0.00 | 0.00 | 0.00 | 0.00 | 0.00 | 0.00 | Proteobacteria | Betaproteobacteria | Rhodocyclales | Rhodocyclaceae | Transient-rare taxa |
| OTU 1330 | 0.00 | 0.00 | 0.03 | 0.00 | 0.00 | 0.00 | 0.00 | 0.01 | 0.00 | 0.00 | 0.00 | 0.00 | Actinobacteria | Actinobacteria | Actinobacteridae | Actinomycetales | Transient-rare taxa |
| OTU 1331 | 0.00 | 0.00 | 0.00 | 0.00 | 0.00 | 0.02 | 0.00 | 0.01 | 0.00 | 0.00 | 0.00 | 0.01 | Proteobacteria | Alphaproteobacteria | Sphingomonadales | Sphingomonadaceae | Transient-rare taxa |
| OTU 1332 | 0.00 | 0.00 | 0.00 | 0.00 | 0.00 | 0.00 | 0.01 | 0.00 | 0.02 | 0.00 | 0.00 | 0.00 | Bacteroidetes | Bacteroidia | Bacteroidales | Marinilabiliaceae | Transient-rare taxa |
| OTU 1333 | 0.00 | 0.00 | 0.02 | 0.00 | 0.00 | 0.00 | 0.01 | 0.00 | 0.00 | 0.00 | 0.00 | 0.00 | Proteobacteria | Betaproteobacteria | Nitrosomonadales | Nitrosomonadaceae | Transient-rare taxa |
| OTU 1334 | 0.00 | 0.00 | 0.00 | 0.00 | 0.00 | 0.00 | 0.02 | 0.01 | 0.00 | 0.00 | 0.00 | 0.00 | Proteobacteria | Gammaproteobacteria | Oceanospirillales | Oceanospirillaceae | Transient-rare taxa |
| OTU 1335 | 0.00 | 0.00 | 0.00 | 0.01 | 0.01 | 0.01 | 0.00 | 0.00 | 0.00 | 0.00 | 0.00 | 0.00 | Proteobacteria | Betaproteobacteria | Rhodocyclales | Rhodocyclaceae | Transient-rare taxa |
| OTU 1336 | 0.00 | 0.00 | 0.00 | 0.00 | 0.03 | 0.00 | 0.00 | 0.00 | 0.00 | 0.00 | 0.00 | 0.00 | Proteobacteria | Gammaproteobacteria | Alteromonadales | Alteromonadaceae | Transient-rare taxa |
| OTU 1337 | 0.01 | 0.00 | 0.00 | 0.00 | 0.01 | 0.00 | 0.00 | 0.00 | 0.00 | 0.00 | 0.01 | 0.00 | Planctomycetes | Planctomycetia | Planctomycetales | Planctomycetaceae | Transient-rare taxa |
| OTU 1338 | 0.00 | 0.01 | 0.00 | 0.00 | 0.00 | 0.00 | 0.00 | 0.00 | 0.02 | 0.00 | 0.00 | 0.00 | Proteobacteria | Alphaproteobacteria | Sphingomonadales | Sphingomonadaceae | Transient-rare taxa |
| OTU 1339 | 0.00 | 0.01 | 0.00 | 0.00 | 0.00 | 0.00 | 0.02 | 0.00 | 0.00 | 0.00 | 0.00 | 0.00 | Proteobacteria | Betaproteobacteria | Rhodocyclales | Rhodocyclaceae | Transient-rare taxa |
| OTU 1340 | 0.00 | 0.00 | 0.00 | 0.00 | 0.00 | 0.00 | 0.03 | 0.00 | 0.00 | 0.00 | 0.00 | 0.00 | Proteobacteria | Gammaproteobacteria | Thiotrichales | Thiotrichaceae | Transient-rare taxa |
| OTU 1341 | 0.00 | 0.03 | 0.00 | 0.00 | 0.00 | 0.00 | 0.00 | 0.00 | 0.00 | 0.00 | 0.00 | 0.00 | Proteobacteria | Gammaproteobacteria | Xanthomonadales | Xanthomonadaceae | Transient-rare taxa |
| OTU 1342 | 0.00 | 0.00 | 0.01 | 0.00 | 0.00 | 0.01 | 0.00 | 0.00 | 0.01 | 0.00 | 0.00 | 0.00 | Firmicutes | Clostridia | Clostridiales | Clostridiaceae 1 | Transient-rare taxa |
| OTU 1343 | 0.00 | 0.00 | 0.00 | 0.00 | 0.00 | 0.00 | 0.00 | 0.03 | 0.00 | 0.00 | 0.00 | 0.00 | Chloroflexi | Anaerolineae | Anaerolineales | Anaerolineaceae | Transient-rare taxa |
| OTU 1344 | 0.00 | 0.00 | 0.00 | 0.00 | 0.00 | 0.00 | 0.00 | 0.03 | 0.00 | 0.00 | 0.00 | 0.00 | Planctomycetes | Planctomycetia | Planctomycetales | Planctomycetaceae | Transient-rare taxa |
| OTU 1345 | 0.00 | 0.00 | 0.00 | 0.00 | 0.01 | 0.00 | 0.01 | 0.00 | 0.01 | 0.00 | 0.00 | 0.00 | Proteobacteria | Betaproteobacteria | Burkholderiales | Burkholderiaceae | Transient-rare taxa |
| OTU 1346 | 0.00 | 0.02 | 0.00 | 0.00 | 0.00 | 0.00 | 0.00 | 0.00 | 0.01 | 0.00 | 0.00 | 0.00 | Bacteroidetes | Sphingobacteriia | Sphingobacteriales | Saprospiraceae | Transient-rare taxa |
| OTU 1347 | 0.00 | 0.01 | 0.02 | 0.00 | 0.00 | 0.00 | 0.00 | 0.00 | 0.00 | 0.00 | 0.00 | 0.00 | Proteobacteria | Gammaproteobacteria | Oceanospirillales | Hahellaceae | Transient-rare taxa |
| OTU 1348 | 0.00 | 0.00 | 0.00 | 0.00 | 0.00 | 0.01 | 0.00 | 0.00 | 0.00 | 0.00 | 0.01 | 0.01 | Proteobacteria | Alphaproteobacteria | Rhizobiales | Bradyrhizobiaceae | Transient-rare taxa |
| OTU 1349 | 0.00 | 0.00 | 0.00 | 0.00 | 0.00 | 0.02 | 0.01 | 0.00 | 0.00 | 0.00 | 0.00 | 0.00 | Proteobacteria | Deltaproteobacteria | Myxococcales | Nannocystineae | Transient-rare taxa |
| OTU 1350 | 0.00 | 0.00 | 0.00 | 0.00 | 0.00 | 0.00 | 0.00 | 0.00 | 0.00 | 0.00 | 0.03 | 0.00 | Proteobacteria | Deltaproteobacteria | Myxococcales | Nannocystineae | Transient-rare taxa |
| OTU 1351 | 0.00 | 0.00 | 0.00 | 0.00 | 0.00 | 0.00 | 0.00 | 0.00 | 0.03 | 0.00 | 0.00 | 0.00 | candidate division WPS-1 | WPS-1_genera_incertae_sedis | | | Transient-rare taxa |
| OTU 1352 | 0.00 | 0.00 | 0.01 | 0.00 | 0.00 | 0.00 | 0.00 | 0.00 | 0.01 | 0.00 | 0.01 | 0.00 | Proteobacteria | Betaproteobacteria | Rhodocyclales | Rhodocyclaceae | Transient-rare taxa |
| OTU 1353 | 0.00 | 0.00 | 0.01 | 0.00 | 0.00 | 0.01 | 0.00 | 0.00 | 0.00 | 0.00 | 0.01 | 0.00 | Proteobacteria | Betaproteobacteria | Burkholderiales | Burkholderiales_incertae_sedis | Transient-rare taxa |
| OTU 1354 | 0.00 | 0.00 | 0.00 | 0.00 | 0.02 | 0.00 | 0.01 | 0.00 | 0.00 | 0.00 | 0.00 | 0.00 | Proteobacteria | Alphaproteobacteria | Rhodobacterales | Rhodobacteraceae | Transient-rare taxa |
| OTU 1355 | 0.00 | 0.00 | 0.00 | 0.01 | 0.02 | 0.00 | 0.00 | 0.00 | 0.00 | 0.00 | 0.00 | 0.00 | Bacteroidetes | Bacteroidia | Bacteroidales | Bacteroidaceae | Transient-rare taxa |
| OTU 1356 | 0.00 | 0.00 | 0.00 | 0.00 | 0.00 | 0.00 | 0.00 | 0.01 | 0.01 | 0.00 | 0.01 | 0.00 | Proteobacteria | Alphaproteobacteria | Rhodobacterales | Rhodobacteraceae | Transient-rare taxa |
| OTU 1357 | 0.00 | 0.02 | 0.00 | 0.00 | 0.00 | 0.00 | 0.00 | 0.00 | 0.01 | 0.00 | 0.00 | 0.00 | Synergistetes | Synergistia | Synergistales | Synergistaceae | Transient-rare taxa |
| OTU 1358 | 0.00 | 0.00 | 0.00 | 0.00 | 0.02 | 0.01 | 0.00 | 0.00 | 0.00 | 0.00 | 0.00 | 0.00 | Bacteroidetes | Flavobacteriia | Flavobacteriales | Flavobacteriaceae | Transient-rare taxa |
| OTU 1359 | 0.00 | 0.00 | 0.01 | 0.00 | 0.01 | 0.00 | 0.01 | 0.00 | 0.00 | 0.00 | 0.00 | 0.00 | Proteobacteria | Gammaproteobacteria | Chromatiales | Ectothiorhodospiraceae | Transient-rare taxa |
| OTU 1360 | 0.00 | 0.01 | 0.00 | 0.00 | 0.00 | 0.00 | 0.00 | 0.00 | 0.01 | 0.00 | 0.01 | 0.00 | Proteobacteria | Alphaproteobacteria | Rhizobiales | Rhodobiaceae | Transient-rare taxa |
| OTU 1361 | 0.00 | 0.03 | 0.00 | 0.00 | 0.00 | 0.00 | 0.00 | 0.00 | 0.00 | 0.00 | 0.00 | 0.00 | Proteobacteria | Betaproteobacteria | Burkholderiales | Oxalobacteraceae | Transient-rare taxa |
| OTU 1362 | 0.00 | 0.00 | 0.00 | 0.00 | 0.00 | 0.00 | 0.03 | 0.00 | 0.00 | 0.00 | 0.00 | 0.00 | Proteobacteria | Betaproteobacteria | Burkholderiales | Burkholderiaceae | Transient-rare taxa |
| OTU 1363 | 0.00 | 0.00 | 0.01 | 0.00 | 0.00 | 0.00 | 0.02 | 0.00 | 0.00 | 0.00 | 0.00 | 0.00 | Proteobacteria | Betaproteobacteria | Rhodocyclales | Rhodocyclaceae | Transient-rare taxa |
| OTU 1364 | 0.00 | 0.00 | 0.00 | 0.00 | 0.00 | 0.00 | 0.00 | 0.00 | 0.00 | 0.00 | 0.03 | 0.00 | Proteobacteria | Deltaproteobacteria | Myxococcales | Nannocystineae | Transient-rare taxa |
| OTU 1365 | 0.00 | 0.00 | 0.00 | 0.00 | 0.00 | 0.00 | 0.00 | 0.00 | 0.02 | 0.00 | 0.01 | 0.00 | Bacteroidetes | Sphingobacteriia | Sphingobacteriales | Saprospiraceae | Transient-rare taxa |
| OTU 1366 | 0.00 | 0.00 | 0.00 | 0.00 | 0.00 | 0.00 | 0.00 | 0.00 | 0.00 | 0.00 | 0.02 | 0.01 | Proteobacteria | Betaproteobacteria | Rhodocyclales | Rhodocyclaceae | Transient-rare taxa |
| OTU 1367 | 0.00 | 0.01 | 0.00 | 0.00 | 0.00 | 0.00 | 0.00 | 0.00 | 0.00 | 0.00 | 0.02 | 0.00 | Proteobacteria | Alphaproteobacteria | Rhodobacterales | Rhodobacteraceae | Transient-rare taxa |
| OTU 1368 | 0.00 | 0.00 | 0.00 | 0.00 | 0.00 | 0.00 | 0.00 | 0.00 | 0.00 | 0.00 | 0.03 | 0.00 | Proteobacteria | Deltaproteobacteria | Myxococcales | Nannocystineae | Transient-rare taxa |
| OTU 1369 | 0.00 | 0.00 | 0.00 | 0.00 | 0.00 | 0.00 | 0.00 | 0.00 | 0.00 | 0.00 | 0.01 | 0.02 | SR1 | SR1_genera_incertae_sedis | | | Transient-rare taxa |
| OTU 1370 | 0.01 | 0.00 | 0.00 | 0.00 | 0.00 | 0.00 | 0.00 | 0.00 | 0.00 | 0.00 | 0.01 | 0.01 | Bacteroidetes | Sphingobacteriia | Sphingobacteriales | Chitinophagaceae | Transient-rare taxa |
| OTU 1371 | 0.00 | 0.00 | 0.00 | 0.00 | 0.00 | 0.01 | 0.00 | 0.00 | 0.02 | 0.00 | 0.00 | 0.00 | Bacteroidetes | Bacteroidia | Bacteroidales | Bacteroidaceae | Transient-rare taxa |
| OTU 1372 | 0.00 | 0.00 | 0.01 | 0.00 | 0.00 | 0.01 | 0.00 | 0.00 | 0.01 | 0.00 | 0.00 | 0.00 | Bacteroidetes | Sphingobacteriia | Sphingobacteriales | Saprospiraceae | Transient-rare taxa |
| OTU 1373 | 0.00 | 0.00 | 0.00 | 0.00 | 0.01 | 0.02 | 0.00 | 0.00 | 0.00 | 0.00 | 0.00 | 0.00 | Fusobacteria | Fusobacteriia | Fusobacteriales | Fusobacteriaceae | Transient-rare taxa |
| OTU 1374 | 0.00 | 0.00 | 0.00 | 0.00 | 0.01 | 0.00 | 0.00 | 0.00 | 0.00 | 0.00 | 0.02 | 0.00 | Bacteroidetes | Flavobacteriia | Flavobacteriales | Flavobacteriaceae | Transient-rare taxa |
| OTU 1375 | 0.00 | 0.00 | 0.01 | 0.00 | 0.00 | 0.00 | 0.00 | 0.00 | 0.02 | 0.00 | 0.00 | 0.00 | Proteobacteria | Betaproteobacteria | Hydrogenophilales | Hydrogenophilaceae | Transient-rare taxa |
| OTU 1376 | 0.01 | 0.02 | 0.00 | 0.00 | 0.00 | 0.00 | 0.00 | 0.00 | 0.00 | 0.00 | 0.00 | 0.00 | Proteobacteria | Betaproteobacteria | Rhodocyclales | Rhodocyclaceae | Transient-rare taxa |
| OTU 1377 | 0.00 | 0.03 | 0.00 | 0.00 | 0.00 | 0.00 | 0.00 | 0.00 | 0.00 | 0.00 | 0.00 | 0.00 | Proteobacteria | Deltaproteobacteria | Myxococcales | Cystobacterineae | Transient-rare taxa |
| OTU 1378 | 0.00 | 0.02 | 0.00 | 0.00 | 0.00 | 0.00 | 0.00 | 0.01 | 0.00 | 0.00 | 0.00 | 0.00 | Proteobacteria | Betaproteobacteria | Burkholderiales | Burkholderiales_incertae_sedis | Transient-rare taxa |
| OTU 1379 | 0.00 | 0.01 | 0.00 | 0.00 | 0.00 | 0.00 | 0.00 | 0.01 | 0.00 | 0.00 | 0.01 | 0.00 | Proteobacteria | Deltaproteobacteria | Myxococcales | Sorangiineae | Transient-rare taxa |
| OTU 1380 | 0.01 | 0.01 | 0.00 | 0.00 | 0.00 | 0.00 | 0.00 | 0.00 | 0.01 | 0.00 | 0.00 | 0.00 | Proteobacteria | Alphaproteobacteria | Rhodobacterales | Rhodobacteraceae | Transient-rare taxa |
| OTU 1381 | 0.00 | 0.00 | 0.00 | 0.00 | 0.00 | 0.00 | 0.01 | 0.00 | 0.00 | 0.00 | 0.01 | 0.01 | Proteobacteria | Betaproteobacteria | Rhodocyclales | Rhodocyclaceae | Transient-rare taxa |
| OTU 1382 | 0.00 | 0.00 | 0.00 | 0.00 | 0.00 | 0.00 | 0.00 | 0.00 | 0.00 | 0.00 | 0.03 | 0.00 | Proteobacteria | Betaproteobacteria | Rhodocyclales | Rhodocyclaceae | Transient-rare taxa |
| OTU 1383 | 0.00 | 0.00 | 0.00 | 0.00 | 0.00 | 0.00 | 0.03 | 0.00 | 0.00 | 0.00 | 0.00 | 0.00 | Verrucomicrobia | Opitutae | Puniceicoccales | Puniceicoccaceae | Transient-rare taxa |
| OTU 1384 | 0.00 | 0.00 | 0.00 | 0.00 | 0.00 | 0.00 | 0.03 | 0.00 | 0.00 | 0.00 | 0.00 | 0.00 | Proteobacteria | Betaproteobacteria | Rhodocyclales | Rhodocyclaceae | Transient-rare taxa |
| OTU 1385 | 0.00 | 0.00 | 0.00 | 0.00 | 0.00 | 0.00 | 0.00 | 0.00 | 0.00 | 0.00 | 0.02 | 0.01 | Proteobacteria | Gammaproteobacteria | Pseudomonadales | Moraxellaceae | Transient-rare taxa |
| OTU 1386 | 0.00 | 0.00 | 0.00 | 0.00 | 0.00 | 0.00 | 0.03 | 0.00 | 0.00 | 0.00 | 0.00 | 0.00 | Proteobacteria | Alphaproteobacteria | Sphingomonadales | Sphingomonadaceae | Transient-rare taxa |
| OTU 1387 | 0.00 | 0.00 | 0.00 | 0.00 | 0.00 | 0.00 | 0.02 | 0.01 | 0.00 | 0.00 | 0.00 | 0.00 | Planctomycetes | Planctomycetia | Planctomycetales | Planctomycetaceae | Transient-rare taxa |
| OTU 1388 | 0.00 | 0.00 | 0.00 | 0.00 | 0.00 | 0.00 | 0.00 | 0.00 | 0.00 | 0.00 | 0.03 | 0.00 | Proteobacteria | Betaproteobacteria | Burkholderiales | Comamonadaceae | Transient-rare taxa |
| OTU 1389 | 0.02 | 0.00 | 0.00 | 0.00 | 0.00 | 0.00 | 0.00 | 0.00 | 0.00 | 0.00 | 0.01 | 0.00 | Proteobacteria | Gammaproteobacteria | Xanthomonadales | Xanthomonadaceae | Transient-rare taxa |
| OTU 1390 | 0.00 | 0.00 | 0.00 | 0.00 | 0.00 | 0.00 | 0.00 | 0.00 | 0.00 | 0.00 | 0.02 | 0.01 | Planctomycetes | Planctomycetia | Planctomycetales | Planctomycetaceae | Transient-rare taxa |
| OTU 1391 | 0.00 | 0.00 | 0.00 | 0.00 | 0.00 | 0.00 | 0.01 | 0.02 | 0.00 | 0.00 | 0.00 | 0.00 | Actinobacteria | Actinobacteria | Acidimicrobidae | Acidimicrobiales | Transient-rare taxa |
| OTU 1392 | 0.01 | 0.01 | 0.01 | 0.00 | 0.00 | 0.00 | 0.00 | 0.00 | 0.00 | 0.00 | 0.00 | 0.00 | Proteobacteria | Alphaproteobacteria | Rhodobacterales | Rhodobacteraceae | Transient-rare taxa |
| OTU 1393 | 0.00 | 0.02 | 0.00 | 0.00 | 0.00 | 0.00 | 0.00 | 0.01 | 0.00 | 0.00 | 0.00 | 0.00 | Proteobacteria | Deltaproteobacteria | Myxococcales | Nannocystineae | Transient-rare taxa |
| OTU 1394 | 0.02 | 0.01 | 0.00 | 0.00 | 0.00 | 0.00 | 0.00 | 0.00 | 0.00 | 0.00 | 0.00 | 0.00 | Proteobacteria | Betaproteobacteria | Burkholderiales | Comamonadaceae | Transient-rare taxa |
| OTU 1395 | 0.00 | 0.00 | 0.00 | 0.00 | 0.00 | 0.00 | 0.00 | 0.03 | 0.00 | 0.00 | 0.00 | 0.00 | Proteobacteria | Deltaproteobacteria | Myxococcales | Sorangiineae | Transient-rare taxa |
| OTU 1396 | 0.00 | 0.00 | 0.00 | 0.00 | 0.00 | 0.00 | 0.01 | 0.02 | 0.00 | 0.00 | 0.00 | 0.00 | Nitrospirae | Nitrospira | Nitrospirales | Nitrospiraceae | Transient-rare taxa |
| OTU 1397 | 0.01 | 0.01 | 0.00 | 0.00 | 0.00 | 0.00 | 0.00 | 0.00 | 0.01 | 0.00 | 0.00 | 0.00 | Chloroflexi | Anaerolineae | Anaerolineales | Anaerolineaceae | Transient-rare taxa |
| OTU 1398 | 0.00 | 0.00 | 0.00 | 0.00 | 0.00 | 0.00 | 0.01 | 0.00 | 0.02 | 0.00 | 0.00 | 0.00 | Proteobacteria | Betaproteobacteria | Rhodocyclales | Rhodocyclaceae | Transient-rare taxa |
| OTU 1399 | 0.00 | 0.00 | 0.00 | 0.01 | 0.00 | 0.02 | 0.00 | 0.00 | 0.00 | 0.00 | 0.00 | 0.00 | Proteobacteria | Deltaproteobacteria | Desulfobacterales | Desulfobacteraceae | Transient-rare taxa |
| OTU 1400 | 0.00 | 0.02 | 0.00 | 0.01 | 0.00 | 0.00 | 0.00 | 0.00 | 0.00 | 0.00 | 0.00 | 0.00 | Latescibacteria | Latescibacteria_genera_incertae_sedis | | | Transient-rare taxa |
| OTU 1401 | 0.00 | 0.00 | 0.00 | 0.01 | 0.01 | 0.00 | 0.01 | 0.00 | 0.00 | 0.00 | 0.00 | 0.00 | Bacteroidetes | Flavobacteriia | Flavobacteriales | Flavobacteriaceae | Transient-rare taxa |
| OTU 1402 | 0.00 | 0.03 | 0.00 | 0.00 | 0.00 | 0.00 | 0.00 | 0.00 | 0.00 | 0.00 | 0.00 | 0.00 | Proteobacteria | Betaproteobacteria | Rhodocyclales | Rhodocyclaceae | Transient-rare taxa |
| OTU 1403 | 0.00 | 0.00 | 0.00 | 0.01 | 0.00 | 0.02 | 0.00 | 0.00 | 0.00 | 0.00 | 0.00 | 0.00 | Proteobacteria | Betaproteobacteria | Burkholderiales | Burkholderiales_incertae_sedis | Transient-rare taxa |
| OTU 1404 | 0.00 | 0.00 | 0.00 | 0.03 | 0.00 | 0.00 | 0.00 | 0.00 | 0.00 | 0.00 | 0.00 | 0.00 | Proteobacteria | Gammaproteobacteria | Gammaproteobacteria_incertae_sedis | Methylohalomonas | Transient-rare taxa |
| OTU 1405 | 0.01 | 0.02 | 0.00 | 0.00 | 0.00 | 0.00 | 0.00 | 0.00 | 0.00 | 0.00 | 0.00 | 0.00 | Proteobacteria | Gammaproteobacteria | Chromatiales | Chromatiaceae | Transient-rare taxa |
| OTU 1406 | 0.00 | 0.00 | 0.00 | 0.00 | 0.00 | 0.00 | 0.00 | 0.01 | 0.02 | 0.00 | 0.00 | 0.00 | Planctomycetes | Planctomycetia | Planctomycetales | Planctomycetaceae | Transient-rare taxa |
| OTU 1407 | 0.00 | 0.00 | 0.00 | 0.02 | 0.00 | 0.00 | 0.00 | 0.00 | 0.00 | 0.01 | 0.00 | 0.00 | Proteobacteria | Gammaproteobacteria | Oceanospirillales | Oceanospirillaceae | Transient-rare taxa |
| OTU 1408 | 0.00 | 0.00 | 0.00 | 0.02 | 0.00 | 0.00 | 0.00 | 0.00 | 0.00 | 0.00 | 0.00 | 0.01 | Actinobacteria | Actinobacteria | Actinobacteridae | Actinomycetales | Transient-rare taxa |
| OTU 1409 | 0.00 | 0.03 | 0.00 | 0.00 | 0.00 | 0.00 | 0.00 | 0.00 | 0.00 | 0.00 | 0.00 | 0.00 | Bacteroidetes | Sphingobacteriia | Sphingobacteriales | Sphingobacteriaceae | Transient-rare taxa |
| OTU 1410 | 0.00 | 0.03 | 0.00 | 0.00 | 0.00 | 0.00 | 0.00 | 0.00 | 0.00 | 0.00 | 0.00 | 0.00 | Bacteroidetes | Cytophagia | Cytophagales | Flammeovirgaceae | Transient-rare taxa |
| OTU 1411 | 0.00 | 0.01 | 0.00 | 0.00 | 0.00 | 0.01 | 0.00 | 0.00 | 0.00 | 0.00 | 0.00 | 0.01 | Proteobacteria | Betaproteobacteria | Rhodocyclales | Rhodocyclaceae | Transient-rare taxa |
| OTU 1412 | 0.00 | 0.02 | 0.00 | 0.00 | 0.00 | 0.00 | 0.00 | 0.01 | 0.00 | 0.00 | 0.00 | 0.00 | Proteobacteria | Betaproteobacteria | Rhodocyclales | Rhodocyclaceae | Transient-rare taxa |

| | | | | | | | | | | | | | | | | |
|---|---|---|---|---|---|---|---|---|---|---|---|---|---|---|---|---|
| OTU 1413 | 0.00 | 0.00 | 0.00 | 0.00 | 0.03 | 0.00 | 0.00 | 0.00 | 0.00 | 0.00 | 0.00 | 0.00 | Proteobacteria | Deltaproteobacteria | Desulfovibrionales | Desulfovibrionaceae | Transient-rare taxa |
| OTU 1414 | 0.00 | 0.01 | 0.00 | 0.00 | 0.00 | 0.00 | 0.00 | 0.02 | 0.00 | 0.00 | 0.00 | 0.00 | Proteobacteria | Betaproteobacteria | Burkholderiales | Comamonadaceae | Transient-rare taxa |
| OTU 1415 | 0.01 | 0.00 | 0.00 | 0.00 | 0.01 | 0.00 | 0.00 | 0.00 | 0.00 | 0.00 | 0.01 | 0.00 | Proteobacteria | Betaproteobacteria | Rhodocyclales | Rhodocyclaceae | Transient-rare taxa |
| OTU 1416 | 0.00 | 0.00 | 0.00 | 0.01 | 0.01 | 0.01 | 0.00 | 0.00 | 0.00 | 0.00 | 0.00 | 0.00 | Proteobacteria | Gammaproteobacteria | Oceanospirillales | Oceanospirillaceae | Transient-rare taxa |
| OTU 1417 | 0.00 | 0.00 | 0.00 | 0.00 | 0.00 | 0.00 | 0.03 | 0.00 | 0.00 | 0.00 | 0.00 | 0.00 | Proteobacteria | Gammaproteobacteria | Chromatiales | Ectothiorhodospiraceae | Transient-rare taxa |
| OTU 1418 | 0.00 | 0.00 | 0.00 | 0.00 | 0.00 | 0.00 | 0.03 | 0.00 | 0.00 | 0.00 | 0.00 | 0.00 | Proteobacteria | Betaproteobacteria | Ferrovales | Ferrovaceae | Transient-rare taxa |
| OTU 1419 | 0.00 | 0.00 | 0.00 | 0.00 | 0.00 | 0.00 | 0.00 | 0.03 | 0.00 | 0.00 | 0.00 | 0.00 | Proteobacteria | Deltaproteobacteria | Myxococcales | Nannocystineae | Transient-rare taxa |
| OTU 1420 | 0.00 | 0.00 | 0.00 | 0.00 | 0.00 | 0.00 | 0.00 | 0.01 | 0.02 | 0.00 | 0.00 | 0.00 | Proteobacteria | Gammaproteobacteria | Xanthomonadales | Xanthomonadaceae | Transient-rare taxa |
| OTU 1421 | 0.00 | 0.00 | 0.00 | 0.00 | 0.03 | 0.00 | 0.00 | 0.00 | 0.00 | 0.00 | 0.00 | 0.00 | Proteobacteria | Deltaproteobacteria | Myxococcales | Sorangiineae | Transient-rare taxa |
| OTU 1422 | 0.00 | 0.00 | 0.00 | 0.00 | 0.00 | 0.00 | 0.00 | 0.03 | 0.00 | 0.00 | 0.00 | 0.00 | Proteobacteria | Gammaproteobacteria | Oceanospirillales | Hahellaceae | Transient-rare taxa |
| OTU 1423 | 0.00 | 0.01 | 0.00 | 0.00 | 0.02 | 0.00 | 0.00 | 0.00 | 0.00 | 0.00 | 0.00 | 0.00 | Proteobacteria | Betaproteobacteria | Rhodocyclales | Rhodocyclaceae | Transient-rare taxa |
| OTU 1424 | 0.00 | 0.01 | 0.00 | 0.00 | 0.00 | 0.00 | 0.00 | 0.00 | 0.01 | 0.00 | 0.00 | 0.01 | Proteobacteria | Betaproteobacteria | Burkholderiales | Alcaligenaceae | Transient-rare taxa |
| OTU 1425 | 0.01 | 0.00 | 0.00 | 0.00 | 0.00 | 0.00 | 0.00 | 0.00 | 0.02 | 0.00 | 0.00 | 0.00 | Proteobacteria | Gammaproteobacteria | Xanthomonadales | Xanthomonadaceae | Transient-rare taxa |
| OTU 1426 | 0.00 | 0.02 | 0.00 | 0.00 | 0.00 | 0.00 | 0.00 | 0.00 | 0.01 | 0.00 | 0.00 | 0.00 | Proteobacteria | Alphaproteobacteria | Sphingomonadales | Sphingomonadaceae | Transient-rare taxa |
| OTU 1427 | 0.00 | 0.00 | 0.00 | 0.00 | 0.00 | 0.00 | 0.00 | 0.00 | 0.03 | 0.00 | 0.00 | 0.00 | Proteobacteria | Alphaproteobacteria | Rhodospirillales | Rhodospirillaceae | Transient-rare taxa |
| OTU 1428 | 0.00 | 0.03 | 0.00 | 0.00 | 0.00 | 0.00 | 0.00 | 0.00 | 0.00 | 0.00 | 0.00 | 0.00 | Proteobacteria | Alphaproteobacteria | Sphingomonadales | Sphingomonadaceae | Transient-rare taxa |
| OTU 1429 | 0.01 | 0.02 | 0.00 | 0.00 | 0.00 | 0.00 | 0.00 | 0.00 | 0.00 | 0.00 | 0.00 | 0.00 | Firmicutes | Clostridia | Clostridiales | Clostridiales_Incertae Sedis XI | Transient-rare taxa |
| OTU 1430 | 0.01 | 0.01 | 0.00 | 0.00 | 0.00 | 0.00 | 0.00 | 0.00 | 0.00 | 0.00 | 0.00 | 0.01 | Proteobacteria | Betaproteobacteria | Burkholderiales | Comamonadaceae | Transient-rare taxa |
| OTU 1431 | 0.01 | 0.02 | 0.00 | 0.00 | 0.00 | 0.00 | 0.00 | 0.00 | 0.00 | 0.00 | 0.00 | 0.00 | Proteobacteria | Gammaproteobacteria | Chromatiales | Chromatiaceae | Transient-rare taxa |
| OTU 1432 | 0.00 | 0.00 | 0.01 | 0.00 | 0.00 | 0.00 | 0.00 | 0.00 | 0.02 | 0.00 | 0.00 | 0.00 | Bacteroidetes | Flavobacteriia | Flavobacteriales | Flavobacteriaceae | Transient-rare taxa |
| OTU 1433 | 0.00 | 0.00 | 0.01 | 0.00 | 0.00 | 0.00 | 0.00 | 0.01 | 0.01 | 0.00 | 0.00 | 0.00 | Proteobacteria | Alphaproteobacteria | Rhodobacterales | Rhodobacteraceae | Transient-rare taxa |
| OTU 1434 | 0.00 | 0.00 | 0.00 | 0.02 | 0.01 | 0.00 | 0.00 | 0.00 | 0.00 | 0.00 | 0.00 | 0.00 | Bacteroidetes | Flavobacteriia | Flavobacteriales | Flavobacteriaceae | Transient-rare taxa |
| OTU 1435 | 0.00 | 0.00 | 0.00 | 0.03 | 0.00 | 0.00 | 0.00 | 0.00 | 0.00 | 0.00 | 0.00 | 0.00 | Proteobacteria | Epsilonproteobacteria | Campylobacterales | Campylobacteraceae | Transient-rare taxa |
| OTU 1436 | 0.00 | 0.02 | 0.00 | 0.00 | 0.00 | 0.01 | 0.00 | 0.00 | 0.00 | 0.00 | 0.00 | 0.00 | Proteobacteria | Betaproteobacteria | Burkholderiales | Burkholderiaceae | Transient-rare taxa |
| OTU 1437 | 0.00 | 0.01 | 0.00 | 0.00 | 0.00 | 0.00 | 0.00 | 0.00 | 0.01 | 0.00 | 0.01 | 0.00 | Proteobacteria | Betaproteobacteria | Burkholderiales | Comamonadaceae | Transient-rare taxa |
| OTU 1438 | 0.02 | 0.01 | 0.00 | 0.00 | 0.00 | 0.00 | 0.00 | 0.00 | 0.00 | 0.00 | 0.00 | 0.00 | Proteobacteria | Gammaproteobacteria | Pseudomonadales | Pseudomonadaceae | Transient-rare taxa |
| OTU 1439 | 0.00 | 0.00 | 0.00 | 0.00 | 0.00 | 0.00 | 0.01 | 0.01 | 0.01 | 0.00 | 0.00 | 0.00 | Proteobacteria | Betaproteobacteria | Rhodocyclales | Rhodocyclaceae | Transient-rare taxa |
| OTU 1440 | 0.00 | 0.00 | 0.00 | 0.00 | 0.00 | 0.00 | 0.00 | 0.03 | 0.00 | 0.00 | 0.00 | 0.00 | Actinobacteria | Actinobacteria | Acidimicrobidae | Acidimicrobiales | Transient-rare taxa |
| OTU 1441 | 0.00 | 0.00 | 0.00 | 0.00 | 0.03 | 0.00 | 0.00 | 0.00 | 0.00 | 0.00 | 0.00 | 0.00 | Proteobacteria | Epsilonproteobacteria | Campylobacterales | Campylobacteraceae | Transient-rare taxa |
| OTU 1442 | 0.00 | 0.00 | 0.00 | 0.00 | 0.02 | 0.00 | 0.01 | 0.00 | 0.00 | 0.00 | 0.00 | 0.00 | Proteobacteria | Alphaproteobacteria | Rhodospirillales | Rhodospirillaceae | Transient-rare taxa |
| OTU 1443 | 0.00 | 0.01 | 0.00 | 0.00 | 0.00 | 0.00 | 0.00 | 0.00 | 0.00 | 0.00 | 0.02 | 0.00 | Proteobacteria | Betaproteobacteria | Nitrosomonadales | Nitrosomonadaceae | Transient-rare taxa |
| OTU 1444 | 0.00 | 0.00 | 0.00 | 0.03 | 0.00 | 0.00 | 0.00 | 0.00 | 0.00 | 0.00 | 0.00 | 0.00 | Bacteroidetes | Flavobacteriia | Flavobacteriales | Flavobacteriaceae | Transient-rare taxa |
| OTU 1445 | 0.00 | 0.00 | 0.00 | 0.00 | 0.00 | 0.00 | 0.00 | 0.02 | 0.01 | 0.00 | 0.00 | 0.00 | Proteobacteria | Betaproteobacteria | Rhodocyclales | Rhodocyclaceae | Transient-rare taxa |
| OTU 1446 | 0.00 | 0.00 | 0.00 | 0.00 | 0.00 | 0.00 | 0.00 | 0.00 | 0.03 | 0.00 | 0.00 | 0.00 | Bacteroidetes | Sphingobacteriia | Sphingobacteriales | Chitinophagaceae | Transient-rare taxa |
| OTU 1447 | 0.00 | 0.00 | 0.00 | 0.03 | 0.00 | 0.00 | 0.00 | 0.00 | 0.00 | 0.00 | 0.00 | 0.00 | Proteobacteria | Betaproteobacteria | Burkholderiales | Oxalobacteraceae | Transient-rare taxa |
| OTU 1448 | 0.00 | 0.00 | 0.00 | 0.03 | 0.00 | 0.00 | 0.00 | 0.00 | 0.00 | 0.00 | 0.00 | 0.00 | Proteobacteria | Gammaproteobacteria | Xanthomonadales | Xanthomonadaceae | Transient-rare taxa |
| OTU 1449 | 0.00 | 0.00 | 0.01 | 0.00 | 0.00 | 0.00 | 0.00 | 0.01 | 0.01 | 0.00 | 0.00 | 0.00 | Planctomycetes | Planctomycetia | Planctomycetales | Planctomycetaceae | Transient-rare taxa |
| OTU 1450 | 0.00 | 0.00 | 0.00 | 0.00 | 0.00 | 0.01 | 0.00 | 0.00 | 0.01 | 0.01 | 0.00 | 0.00 | Proteobacteria | Betaproteobacteria | Nitrosomonadales | Nitrosomonadaceae | Transient-rare taxa |
| OTU 1451 | 0.00 | 0.01 | 0.01 | 0.00 | 0.00 | 0.00 | 0.00 | 0.00 | 0.00 | 0.00 | 0.00 | 0.01 | Proteobacteria | Betaproteobacteria | Rhodocyclales | Rhodocyclaceae | Transient-rare taxa |
| OTU 1452 | 0.00 | 0.03 | 0.00 | 0.00 | 0.00 | 0.00 | 0.00 | 0.00 | 0.00 | 0.00 | 0.00 | 0.00 | Proteobacteria | Alphaproteobacteria | Sphingomonadales | Sphingomonadaceae | Transient-rare taxa |
| OTU 1453 | 0.00 | 0.00 | 0.00 | 0.00 | 0.01 | 0.02 | 0.00 | 0.00 | 0.00 | 0.00 | 0.00 | 0.00 | Verrucomicrobia | Verrucomicrobiae | Verrucomicrobiales | Verrucomicrobiaceae | Transient-rare taxa |
| OTU 1454 | 0.01 | 0.01 | 0.00 | 0.00 | 0.00 | 0.00 | 0.00 | 0.00 | 0.00 | 0.01 | 0.00 | 0.00 | Proteobacteria | Betaproteobacteria | Rhodocyclales | Rhodocyclaceae | Transient-rare taxa |
| OTU 1455 | 0.00 | 0.00 | 0.00 | 0.01 | 0.00 | 0.02 | 0.00 | 0.00 | 0.00 | 0.00 | 0.00 | 0.00 | Bacteroidetes | Flavobacteriia | Flavobacteriales | Cryomorphaceae | Transient-rare taxa |
| OTU 1456 | 0.00 | 0.00 | 0.00 | 0.01 | 0.00 | 0.01 | 0.01 | 0.00 | 0.00 | 0.00 | 0.00 | 0.00 | Proteobacteria | Betaproteobacteria | Neisseriales | Neisseriaceae | Transient-rare taxa |
| OTU 1457 | 0.00 | 0.00 | 0.01 | 0.00 | 0.00 | 0.01 | 0.00 | 0.01 | 0.00 | 0.00 | 0.00 | 0.00 | Proteobacteria | Deltaproteobacteria | Myxococcales | Nannocystineae | Transient-rare taxa |
| OTU 1458 | 0.00 | 0.01 | 0.00 | 0.00 | 0.00 | 0.00 | 0.00 | 0.02 | 0.00 | 0.00 | 0.00 | 0.00 | Proteobacteria | Deltaproteobacteria | Myxococcales | Nannocystineae | Transient-rare taxa |
| OTU 1459 | 0.00 | 0.00 | 0.01 | 0.00 | 0.00 | 0.01 | 0.01 | 0.00 | 0.00 | 0.00 | 0.00 | 0.00 | Bacteroidetes | Bacteroidia | Bacteroidales | Sunxiuqinia | Transient-rare taxa |
| OTU 1460 | 0.00 | 0.00 | 0.00 | 0.01 | 0.00 | 0.02 | 0.00 | 0.00 | 0.00 | 0.00 | 0.00 | 0.00 | Proteobacteria | Epsilonproteobacteria | Nautiliales | Nautiliaceae | Transient-rare taxa |
| OTU 1461 | 0.00 | 0.00 | 0.00 | 0.00 | 0.00 | 0.03 | 0.00 | 0.00 | 0.00 | 0.00 | 0.00 | 0.00 | Proteobacteria | Betaproteobacteria | Burkholderiales | Alcaligenaceae | Transient-rare taxa |
| OTU 1462 | 0.02 | 0.00 | 0.00 | 0.00 | 0.00 | 0.01 | 0.00 | 0.00 | 0.00 | 0.00 | 0.00 | 0.00 | Aquificae | Aquificae | Aquificales | Aquificaceae | Transient-rare taxa |
| OTU 1463 | 0.00 | 0.00 | 0.00 | 0.00 | 0.00 | 0.00 | 0.01 | 0.00 | 0.01 | 0.00 | 0.01 | 0.00 | Bacteroidetes | Flavobacteriia | Flavobacteriales | Cryomorphaceae | Transient-rare taxa |
| OTU 1464 | 0.00 | 0.00 | 0.00 | 0.01 | 0.01 | 0.01 | 0.00 | 0.00 | 0.00 | 0.00 | 0.00 | 0.00 | Bacteroidetes | Flavobacteriia | Flavobacteriales | Cryomorphaceae | Transient-rare taxa |
| OTU 1465 | 0.00 | 0.01 | 0.00 | 0.01 | 0.00 | 0.01 | 0.00 | 0.00 | 0.00 | 0.00 | 0.00 | 0.00 | Proteobacteria | Gammaproteobacteria | Alteromonadales | Alteromonadaceae | Transient-rare taxa |
| OTU 1466 | 0.00 | 0.00 | 0.00 | 0.00 | 0.00 | 0.01 | 0.00 | 0.00 | 0.01 | 0.00 | 0.01 | 0.00 | Actinobacteria | Actinobacteria | Actinobacteridae | Actinomycetales | Transient-rare taxa |
| OTU 1467 | 0.02 | 0.00 | 0.00 | 0.00 | 0.00 | 0.00 | 0.00 | 0.00 | 0.01 | 0.00 | 0.00 | 0.00 | Proteobacteria | Betaproteobacteria | Rhodocyclales | Rhodocyclaceae | Transient-rare taxa |
| OTU 1468 | 0.00 | 0.00 | 0.00 | 0.03 | 0.00 | 0.00 | 0.00 | 0.00 | 0.00 | 0.00 | 0.00 | 0.00 | Proteobacteria | Alphaproteobacteria | Rhodobacterales | Rhodobacteraceae | Transient-rare taxa |
| OTU 1469 | 0.00 | 0.01 | 0.00 | 0.00 | 0.00 | 0.00 | 0.01 | 0.00 | 0.00 | 0.00 | 0.00 | 0.01 | Firmicutes | Clostridia | Clostridiales | Clostridiaceae 1 | Transient-rare taxa |
| OTU 1470 | 0.00 | 0.00 | 0.00 | 0.00 | 0.00 | 0.00 | 0.00 | 0.00 | 0.00 | 0.00 | 0.03 | 0.00 | Bacteroidetes | Flavobacteriia | Flavobacteriales | Flavobacteriaceae | Transient-rare taxa |
| OTU 1471 | 0.01 | 0.02 | 0.00 | 0.00 | 0.00 | 0.00 | 0.00 | 0.00 | 0.00 | 0.00 | 0.00 | 0.00 | Proteobacteria | Deltaproteobacteria | Myxococcales | Cystobacterineae | Transient-rare taxa |
| OTU 1472 | 0.00 | 0.02 | 0.00 | 0.00 | 0.00 | 0.00 | 0.00 | 0.00 | 0.01 | 0.00 | 0.00 | 0.00 | Proteobacteria | Gammaproteobacteria | Xanthomonadales | Xanthomonadaceae | Transient-rare taxa |
| OTU 1473 | 0.00 | 0.01 | 0.01 | 0.00 | 0.00 | 0.00 | 0.00 | 0.01 | 0.00 | 0.00 | 0.00 | 0.00 | Chloroflexi | Anaerolineae | Anaerolineales | Anaerolineaceae | Transient-rare taxa |
| OTU 1474 | 0.00 | 0.02 | 0.00 | 0.00 | 0.00 | 0.00 | 0.00 | 0.00 | 0.01 | 0.00 | 0.00 | 0.00 | Proteobacteria | Gammaproteobacteria | Xanthomonadales | Xanthomonadaceae | Transient-rare taxa |
| OTU 1475 | 0.02 | 0.00 | 0.00 | 0.00 | 0.00 | 0.00 | 0.00 | 0.00 | 0.00 | 0.00 | 0.01 | 0.00 | Proteobacteria | Betaproteobacteria | Rhodocyclales | Rhodocyclaceae | Transient-rare taxa |
| OTU 1476 | 0.00 | 0.02 | 0.00 | 0.00 | 0.00 | 0.01 | 0.00 | 0.00 | 0.00 | 0.00 | 0.00 | 0.00 | Planctomycetes | Planctomycetia | Planctomycetales | Planctomycetaceae | Transient-rare taxa |
| OTU 1477 | 0.00 | 0.01 | 0.00 | 0.00 | 0.00 | 0.00 | 0.00 | 0.02 | 0.00 | 0.00 | 0.00 | 0.00 | Proteobacteria | Deltaproteobacteria | Myxococcales | Nannocystineae | Transient-rare taxa |
| OTU 1478 | 0.01 | 0.00 | 0.00 | 0.00 | 0.00 | 0.00 | 0.00 | 0.00 | 0.00 | 0.01 | 0.01 | 0.00 | Verrucomicrobia | Opitutae | Opitutales | Opitutaceae | Transient-rare taxa |
| OTU 1479 | 0.00 | 0.00 | 0.00 | 0.00 | 0.00 | 0.00 | 0.03 | 0.00 | 0.00 | 0.00 | 0.00 | 0.00 | Proteobacteria | Gammaproteobacteria | Thiotrichales | Thiotrichaceae | Transient-rare taxa |
| OTU 1480 | 0.03 | 0.00 | 0.00 | 0.00 | 0.00 | 0.00 | 0.00 | 0.00 | 0.00 | 0.00 | 0.00 | 0.00 | Bacteroidetes | Bacteroidia | Bacteroidales | Marinilabiliaceae | Transient-rare taxa |
| OTU 1481 | 0.03 | 0.00 | 0.00 | 0.00 | 0.00 | 0.00 | 0.00 | 0.00 | 0.00 | 0.00 | 0.00 | 0.00 | Proteobacteria | Betaproteobacteria | Rhodocyclales | Rhodocyclaceae | Transient-rare taxa |
| OTU 1482 | 0.03 | 0.00 | 0.00 | 0.00 | 0.00 | 0.00 | 0.00 | 0.00 | 0.00 | 0.00 | 0.00 | 0.00 | Bacteroidetes | Sphingobacteriia | Sphingobacteriales | Chitinophagaceae | Transient-rare taxa |
| OTU 1483 | 0.02 | 0.00 | 0.00 | 0.00 | 0.00 | 0.00 | 0.00 | 0.00 | 0.00 | 0.00 | 0.00 | 0.01 | Bacteroidetes | Sphingobacteriia | Sphingobacteriales | Cyclobacteriaceae | Transient-rare taxa |
| OTU 1484 | 0.00 | 0.00 | 0.01 | 0.00 | 0.01 | 0.01 | 0.00 | 0.00 | 0.00 | 0.00 | 0.00 | 0.00 | Proteobacteria | Deltaproteobacteria | Desulfobacterales | Desulfobacteraceae | Transient-rare taxa |
| OTU 1485 | 0.00 | 0.00 | 0.01 | 0.00 | 0.00 | 0.00 | 0.00 | 0.01 | 0.01 | 0.00 | 0.00 | 0.00 | Proteobacteria | Betaproteobacteria | Nitrosomonadales | Nitrosomonadaceae | Transient-rare taxa |
| OTU 1486 | 0.00 | 0.00 | 0.00 | 0.00 | 0.00 | 0.00 | 0.00 | 0.03 | 0.00 | 0.00 | 0.00 | 0.00 | Chloroflexi | Anaerolineae | Anaerolineales | Anaerolineaceae | Transient-rare taxa |
| OTU 1487 | 0.00 | 0.00 | 0.01 | 0.01 | 0.00 | 0.00 | 0.00 | 0.01 | 0.00 | 0.00 | 0.00 | 0.00 | Bacteroidetes | Sphingobacteriia | Sphingobacteriales | Saprospiraceae | Transient-rare taxa |
| OTU 1488 | 0.00 | 0.01 | 0.00 | 0.01 | 0.00 | 0.00 | 0.00 | 0.01 | 0.00 | 0.00 | 0.00 | 0.00 | Proteobacteria | Betaproteobacteria | Rhodocyclales | Rhodocyclaceae | Transient-rare taxa |
| OTU 1489 | 0.00 | 0.00 | 0.00 | 0.00 | 0.02 | 0.01 | 0.00 | 0.00 | 0.00 | 0.00 | 0.00 | 0.00 | Proteobacteria | Gammaproteobacteria | Gammaproteobacteria_incertae_sedis | Sedimenticola | Transient-rare taxa |
| OTU 1490 | 0.00 | 0.00 | 0.00 | 0.00 | 0.00 | 0.00 | 0.00 | 0.03 | 0.00 | 0.00 | 0.00 | 0.00 | Proteobacteria | Deltaproteobacteria | Myxococcales | Sorangiineae | Transient-rare taxa |
| OTU 1491 | 0.02 | 0.00 | 0.00 | 0.00 | 0.01 | 0.00 | 0.00 | 0.00 | 0.00 | 0.00 | 0.00 | 0.00 | Actinobacteria | Actinobacteria | Acidimicrobidae | Acidimicrobiales | Transient-rare taxa |
| OTU 1492 | 0.02 | 0.00 | 0.00 | 0.00 | 0.00 | 0.00 | 0.00 | 0.00 | 0.00 | 0.00 | 0.00 | 0.01 | Proteobacteria | Betaproteobacteria | Rhodocyclales | Rhodocyclaceae | Transient-rare taxa |
| OTU 1493 | 0.00 | 0.00 | 0.00 | 0.03 | 0.00 | 0.00 | 0.00 | 0.00 | 0.00 | 0.00 | 0.00 | 0.00 | Bacteroidetes | Flavobacteriia | Flavobacteriales | Flavobacteriaceae | Transient-rare taxa |
| OTU 1494 | 0.03 | 0.00 | 0.00 | 0.00 | 0.00 | 0.00 | 0.00 | 0.00 | 0.00 | 0.00 | 0.00 | 0.00 | Proteobacteria | Gammaproteobacteria | Thiotrichales | Thiotrichaceae | Transient-rare taxa |
| OTU 1495 | 0.02 | 0.00 | 0.00 | 0.00 | 0.00 | 0.00 | 0.00 | 0.00 | 0.00 | 0.00 | 0.00 | 0.01 | Bacteroidetes | Flavobacteriia | Flavobacteriales | Cryomorphaceae | Transient-rare taxa |
| OTU 1496 | 0.02 | 0.01 | 0.00 | 0.00 | 0.00 | 0.00 | 0.00 | 0.00 | 0.00 | 0.00 | 0.00 | 0.00 | Bacteroidetes | Sphingobacteriia | Sphingobacteriales | Sphingobacteriaceae | Transient-rare taxa |
| OTU 1497 | 0.03 | 0.00 | 0.00 | 0.00 | 0.00 | 0.00 | 0.00 | 0.00 | 0.00 | 0.00 | 0.00 | 0.00 | Proteobacteria | Betaproteobacteria | Burkholderiales | Comamonadaceae | Transient-rare taxa |
| OTU 1498 | 0.00 | 0.00 | 0.00 | 0.00 | 0.00 | 0.00 | 0.01 | 0.01 | 0.01 | 0.00 | 0.00 | 0.00 | Proteobacteria | Betaproteobacteria | Burkholderiales | Burkholderiales_incertae_sedis | Transient-rare taxa |
| OTU 1499 | 0.02 | 0.01 | 0.00 | 0.00 | 0.00 | 0.00 | 0.00 | 0.00 | 0.00 | 0.00 | 0.00 | 0.00 | Proteobacteria | Gammaproteobacteria | Xanthomonadales | Xanthomonadaceae | Transient-rare taxa |
| OTU 1500 | 0.02 | 0.01 | 0.00 | 0.00 | 0.00 | 0.00 | 0.00 | 0.00 | 0.00 | 0.00 | 0.00 | 0.00 | Proteobacteria | Deltaproteobacteria | Myxococcales | Sorangiineae | Transient-rare taxa |
| OTU 1501 | 0.00 | 0.03 | 0.00 | 0.00 | 0.00 | 0.00 | 0.00 | 0.00 | 0.00 | 0.00 | 0.00 | 0.00 | Proteobacteria | Gammaproteobacteria | Xanthomonadales | Xanthomonadaceae | Transient-rare taxa |
| OTU 1502 | 0.00 | 0.00 | 0.00 | 0.00 | 0.00 | 0.00 | 0.00 | 0.01 | 0.02 | 0.00 | 0.00 | 0.00 | Chloroflexi | Caldilineae | Caldilineales | Caldilineaceae | Transient-rare taxa |
| OTU 1503 | 0.00 | 0.00 | 0.00 | 0.01 | 0.00 | 0.00 | 0.00 | 0.00 | 0.00 | 0.00 | 0.02 | 0.00 | Actinobacteria | Actinobacteria | Rubrobacteridae | Solirubrobacterales | Transient-rare taxa |
| OTU 1504 | 0.00 | 0.00 | 0.00 | 0.02 | 0.01 | 0.00 | 0.00 | 0.00 | 0.00 | 0.00 | 0.00 | 0.00 | Proteobacteria | Betaproteobacteria | Burkholderiales | Oxalobacteraceae | Transient-rare taxa |
| OTU 1505 | 0.01 | 0.02 | 0.00 | 0.00 | 0.00 | 0.00 | 0.00 | 0.00 | 0.00 | 0.00 | 0.00 | 0.00 | Proteobacteria | Alphaproteobacteria | Sphingomonadales | Sphingomonadaceae | Transient-rare taxa |
| OTU 1506 | 0.01 | 0.00 | 0.00 | 0.00 | 0.00 | 0.00 | 0.00 | 0.00 | 0.02 | 0.00 | 0.00 | 0.00 | Proteobacteria | Alphaproteobacteria | Rhodospirillales | Rhodospirillaceae | Transient-rare taxa |
| OTU 1507 | 0.01 | 0.00 | 0.00 | 0.00 | 0.00 | 0.02 | 0.00 | 0.00 | 0.00 | 0.00 | 0.00 | 0.00 | Actinobacteria | Actinobacteria | Actinobacteridae | Actinomycetales | Transient-rare taxa |
| OTU 1508 | 0.00 | 0.00 | 0.02 | 0.00 | 0.00 | 0.00 | 0.00 | 0.01 | 0.00 | 0.00 | 0.00 | 0.00 | Actinobacteria | Actinobacteria | Actinobacteridae | Actinomycetales | Transient-rare taxa |
| OTU 1509 | 0.00 | 0.02 | 0.00 | 0.00 | 0.00 | 0.00 | 0.00 | 0.00 | 0.00 | 0.00 | 0.00 | 0.01 | Proteobacteria | Betaproteobacteria | Ferrovales | Ferrovaceae | Transient-rare taxa |
| OTU 1510 | 0.00 | 0.00 | 0.02 | 0.00 | 0.00 | 0.00 | 0.00 | 0.00 | 0.00 | 0.01 | 0.00 | 0.00 | Proteobacteria | Deltaproteobacteria | Myxococcales | Sorangiineae | Transient-rare taxa |
| OTU 1511 | 0.02 | 0.00 | 0.00 | 0.00 | 0.00 | 0.00 | 0.00 | 0.00 | 0.00 | 0.01 | 0.00 | 0.00 | Proteobacteria | Betaproteobacteria | Burkholderiales | Comamonadaceae | Transient-rare taxa |
| OTU 1512 | 0.03 | 0.00 | 0.00 | 0.00 | 0.00 | 0.00 | 0.00 | 0.00 | 0.00 | 0.00 | 0.00 | 0.00 | Actinobacteria | Actinobacteria | Acidimicrobidae | Acidimicrobiales | Transient-rare taxa |
| OTU 1513 | 0.00 | 0.00 | 0.00 | 0.00 | 0.00 | 0.00 | 0.00 | 0.00 | 0.00 | 0.01 | 0.00 | 0.02 | Firmicutes | Negativicutes | Selenomonadales | Veillonellaceae | Transient-rare taxa |
| OTU 1514 | 0.02 | 0.00 | 0.00 | 0.00 | 0.00 | 0.00 | 0.00 | 0.00 | 0.00 | 0.00 | 0.00 | 0.01 | Proteobacteria | Betaproteobacteria | Rhodocyclales | Rhodocyclaceae | Transient-rare taxa |
| OTU 1515 | 0.00 | 0.00 | 0.00 | 0.00 | 0.00 | 0.00 | 0.00 | 0.00 | 0.00 | 0.00 | 0.00 | 0.03 | Proteobacteria | Betaproteobacteria | Hydrogenophilales | Hydrogenophilaceae | Transient-rare taxa |
| OTU 1516 | 0.02 | 0.00 | 0.00 | 0.00 | 0.00 | 0.00 | 0.00 | 0.00 | 0.00 | 0.01 | 0.00 | 0.00 | Bacteroidetes | Sphingobacteriia | Sphingobacteriales | Sphingobacteriaceae | Transient-rare taxa |
| OTU 1517 | 0.00 | 0.00 | 0.00 | 0.00 | 0.00 | 0.01 | 0.00 | 0.00 | 0.02 | 0.00 | 0.00 | 0.00 | Chloroflexi | Anaerolineae | Anaerolineales | Anaerolineaceae | Transient-rare taxa |
| OTU 1518 | 0.00 | 0.00 | 0.00 | 0.01 | 0.00 | 0.02 | 0.00 | 0.00 | 0.00 | 0.00 | 0.00 | 0.00 | Proteobacteria | Alphaproteobacteria | Rhizobiales | Rhizobiales_incertae_sedis | Transient-rare taxa |
| OTU 1519 | 0.01 | 0.00 | 0.00 | 0.00 | 0.00 | 0.00 | 0.00 | 0.00 | 0.00 | 0.00 | 0.02 | 0.00 | Proteobacteria | Deltaproteobacteria | Myxococcales | Sorangiineae | Transient-rare taxa |
| OTU 1520 | 0.01 | 0.00 | 0.00 | 0.00 | 0.00 | 0.00 | 0.00 | 0.00 | 0.00 | 0.01 | 0.01 | 0.00 | Proteobacteria | Gammaproteobacteria | Xanthomonadales | Xanthomonadaceae | Transient-rare taxa |
| OTU 1521 | 0.03 | 0.00 | 0.00 | 0.00 | 0.00 | 0.00 | 0.00 | 0.00 | 0.00 | 0.00 | 0.00 | 0.00 | Proteobacteria | Betaproteobacteria | Burkholderiales | Comamonadaceae | Transient-rare taxa |
| OTU 1522 | 0.00 | 0.00 | 0.00 | 0.00 | 0.00 | 0.01 | 0.00 | 0.00 | 0.01 | 0.01 | 0.00 | 0.00 | Acidobacteria | Acidobacteria_Gp4 | Gp4 | | Transient-rare taxa |
| OTU 1523 | 0.01 | 0.00 | 0.00 | 0.00 | 0.00 | 0.01 | 0.00 | 0.00 | 0.00 | 0.01 | 0.00 | 0.00 | Bacteroidetes | Bacteroidetes_incertae_sedis | Ohtaekwangia | | Transient-rare taxa |
| OTU 1524 | 0.00 | 0.00 | 0.00 | 0.00 | 0.00 | 0.02 | 0.00 | 0.00 | 0.00 | 0.00 | 0.00 | 0.01 | Proteobacteria | Gammaproteobacteria | Xanthomonadales | Xanthomonadaceae | Transient-rare taxa |
| OTU 1525 | 0.00 | 0.00 | 0.00 | 0.00 | 0.00 | 0.00 | 0.00 | 0.01 | 0.00 | 0.00 | 0.00 | 0.02 | Proteobacteria | Deltaproteobacteria | Desulfuromonadales | Geobacteraceae | Transient-rare taxa |
| OTU 1526 | 0.01 | 0.00 | 0.00 | 0.01 | 0.00 | 0.00 | 0.00 | 0.00 | 0.01 | 0.00 | 0.00 | 0.00 | Proteobacteria | Deltaproteobacteria | Myxococcales | Nannocystineae | Transient-rare taxa |
| OTU 1527 | 0.02 | 0.01 | 0.00 | 0.00 | 0.00 | 0.00 | 0.00 | 0.00 | 0.00 | 0.00 | 0.00 | 0.00 | Bacteroidetes | Flavobacteriia | Flavobacteriales | Flavobacteriaceae | Transient-rare taxa |
| OTU 1528 | 0.00 | 0.00 | 0.00 | 0.00 | 0.00 | 0.00 | 0.00 | 0.00 | 0.00 | 0.01 | 0.00 | 0.02 | Acidobacteria | Acidobacteria_Gp7 | Gp7 | | Transient-rare taxa |
| OTU 1529 | 0.01 | 0.00 | 0.00 | 0.00 | 0.00 | 0.00 | 0.00 | 0.00 | 0.00 | 0.00 | 0.00 | 0.02 | Proteobacteria | Alphaproteobacteria | Rhodobacterales | Rhodobacteraceae | Transient-rare taxa |
| OTU 1530 | 0.01 | 0.00 | 0.00 | 0.00 | 0.00 | 0.00 | 0.00 | 0.01 | 0.01 | 0.00 | 0.00 | 0.00 | Bacteroidetes | Sphingobacteriia | Sphingobacteriales | Chitinophagaceae | Transient-rare taxa |
| OTU 1531 | 0.00 | 0.00 | 0.00 | 0.00 | 0.00 | 0.00 | 0.00 | 0.00 | 0.01 | 0.02 | 0.00 | 0.00 | Proteobacteria | Deltaproteobacteria | Myxococcales | Nannocystineae | Transient-rare taxa |
| OTU 1532 | 0.00 | 0.00 | 0.00 | 0.00 | 0.00 | 0.00 | 0.00 | 0.00 | 0.00 | 0.03 | 0.00 | 0.00 | Bacteroidetes | Flavobacteriia | Flavobacteriales | Flavobacteriaceae | Transient-rare taxa |
| OTU 1533 | 0.00 | 0.00 | 0.02 | 0.00 | 0.00 | 0.00 | 0.00 | 0.00 | 0.01 | 0.00 | 0.00 | 0.00 | Proteobacteria | Deltaproteobacteria | Myxococcales | Sorangiineae | Transient-rare taxa |
| OTU 1534 | 0.03 | 0.00 | 0.00 | 0.00 | 0.00 | 0.00 | 0.00 | 0.00 | 0.00 | 0.00 | 0.00 | 0.00 | Proteobacteria | Deltaproteobacteria | Myxococcales | Sorangiineae | Transient-rare taxa |
| OTU 1535 | 0.02 | 0.00 | 0.01 | 0.00 | 0.00 | 0.00 | 0.00 | 0.00 | 0.00 | 0.00 | 0.00 | 0.00 | Proteobacteria | Gammaproteobacteria | Alteromonadales | Maricurvus | Transient-rare taxa |
| OTU 1536 | 0.00 | 0.00 | 0.02 | 0.00 | 0.00 | 0.00 | 0.00 | 0.00 | 0.01 | 0.00 | 0.00 | 0.00 | Proteobacteria | Alphaproteobacteria | Rhodobacterales | Rhodobacteraceae | Transient-rare taxa |
| OTU 1537 | 0.00 | 0.00 | 0.00 | 0.00 | 0.00 | 0.00 | 0.00 | 0.00 | 0.00 | 0.01 | 0.01 | 0.01 | Bacteroidetes | Flavobacteriia | Flavobacteriales | Flavobacteriaceae | Transient-rare taxa |
| OTU 1538 | 0.00 | 0.00 | 0.00 | 0.00 | 0.01 | 0.00 | 0.00 | 0.01 | 0.00 | 0.00 | 0.00 | 0.01 | Hydrogenedentes | Candidatus Hydrogenedens | | | Transient-rare taxa |
| OTU 1539 | 0.02 | 0.00 | 0.00 | 0.00 | 0.00 | 0.01 | 0.00 | 0.00 | 0.00 | 0.00 | 0.00 | 0.00 | Actinobacteria | Actinobacteria | Actinobacteridae | Actinomycetales | Transient-rare taxa |
| OTU 1540 | 0.01 | 0.02 | 0.00 | 0.00 | 0.00 | 0.00 | 0.00 | 0.00 | 0.00 | 0.00 | 0.00 | 0.00 | Proteobacteria | Deltaproteobacteria | Desulfuromonadales | Geobacteraceae | Transient-rare taxa |
| OTU 1541 | 0.00 | 0.00 | 0.00 | 0.00 | 0.00 | 0.00 | 0.00 | 0.00 | 0.01 | 0.02 | 0.00 | 0.00 | Proteobacteria | Epsilonproteobacteria | Campylobacterales | Helicobacteraceae | Transient-rare taxa |
| OTU 1542 | 0.00 | 0.00 | 0.00 | 0.00 | 0.00 | 0.01 | 0.00 | 0.01 | 0.01 | 0.00 | 0.00 | 0.00 | Bacteroidetes | Sphingobacteriia | Sphingobacteriales | Saprospiraceae | Transient-rare taxa |
| OTU 1543 | 0.00 | 0.00 | 0.00 | 0.00 | 0.01 | 0.00 | 0.00 | 0.01 | 0.01 | 0.00 | 0.00 | 0.00 | Proteobacteria | Betaproteobacteria | Rhodocyclales | Rhodocyclaceae | Transient-rare taxa |
| OTU 1544 | 0.00 | 0.00 | 0.00 | 0.00 | 0.00 | 0.00 | 0.00 | 0.00 | 0.00 | 0.02 | 0.00 | 0.01 | Verrucomicrobia | Opitutae | Opitutales | Opitutaceae | Transient-rare taxa |
| OTU 1545 | 0.01 | 0.00 | 0.00 | 0.00 | 0.00 | 0.00 | 0.00 | 0.00 | 0.00 | 0.00 | 0.02 | 0.00 | Proteobacteria | Alphaproteobacteria | Sphingomonadales | Sphingomonadaceae | Transient-rare taxa |
| OTU 1546 | 0.00 | 0.00 | 0.00 | 0.00 | 0.00 | 0.00 | 0.00 | 0.00 | 0.00 | 0.00 | 0.00 | 0.03 | Proteobacteria | Betaproteobacteria | Burkholderiales | Oxalobacteraceae | Transient-rare taxa |
| OTU 1547 | 0.01 | 0.00 | 0.00 | 0.00 | 0.00 | 0.00 | 0.00 | 0.00 | 0.00 | 0.01 | 0.00 | 0.01 | Bacteroidetes | Sphingobacteriia | Sphingobacteriales | Saprospiraceae | Transient-rare taxa |
| OTU 1548 | 0.00 | 0.00 | 0.00 | 0.00 | 0.00 | 0.00 | 0.00 | 0.00 | 0.00 | 0.00 | 0.01 | 0.02 | Planctomycetes | Planctomycetia | Planctomycetales | Planctomycetaceae | Transient-rare taxa |
| OTU 1549 | 0.00 | 0.00 | 0.00 | 0.00 | 0.01 | 0.00 | 0.00 | 0.01 | 0.00 | 0.00 | 0.00 | 0.01 | Proteobacteria | Deltaproteobacteria | Syntrophobacterales | Syntrophobacteraceae | Transient-rare taxa |
| OTU 1550 | 0.01 | 0.00 | 0.01 | 0.00 | 0.00 | 0.00 | 0.00 | 0.00 | 0.00 | 0.01 | 0.00 | 0.00 | Chloroflexi | Anaerolineae | Anaerolineales | Anaerolineaceae | Transient-rare taxa |
| OTU 1551 | 0.00 | 0.00 | 0.00 | 0.00 | 0.00 | 0.00 | 0.00 | 0.03 | 0.00 | 0.00 | 0.00 | 0.00 | Bacteroidetes | Sphingobacteriia | Sphingobacteriales | Saprospiraceae | Transient-rare taxa |
| OTU 1552 | 0.00 | 0.00 | 0.01 | 0.00 | 0.00 | 0.01 | 0.00 | 0.00 | 0.00 | 0.01 | 0.00 | 0.00 | Proteobacteria | Gammaproteobacteria | Legionellales | Legionellaceae | Transient-rare taxa |
| OTU 1553 | 0.00 | 0.00 | 0.01 | 0.00 | 0.00 | 0.00 | 0.00 | 0.02 | 0.00 | 0.00 | 0.00 | 0.00 | Proteobacteria | Epsilonproteobacteria | Campylobacterales | Campylobacteraceae | Transient-rare taxa |
| OTU 1554 | 0.00 | 0.00 | 0.00 | 0.00 | 0.00 | 0.00 | 0.00 | 0.00 | 0.00 | 0.00 | 0.02 | 0.01 | Firmicutes | Bacilli | Bacillales | Bacillaceae 1 | Transient-rare taxa |

| | | | | | | | | | | | | | | | | | |
|---|---|---|---|---|---|---|---|---|---|---|---|---|---|---|---|---|---|
| OTU 1555 | 0.00 | 0.00 | 0.00 | 0.00 | 0.00 | 0.00 | 0.02 | 0.01 | 0.00 | 0.00 | 0.00 | 0.00 | *Proteobacteria* | *Gammaproteobacteria* | *Methylococcales* | *Methylococcaceae* | Transient-rare taxa |
| OTU 1556 | 0.03 | 0.00 | 0.00 | 0.00 | 0.00 | 0.00 | 0.00 | 0.00 | 0.00 | 0.00 | 0.00 | 0.00 | *Proteobacteria* | *Gammaproteobacteria* | *Chromatiales* | *Ectothiorhodospiraceae* | Transient-rare taxa |
| OTU 1557 | 0.02 | 0.01 | 0.00 | 0.00 | 0.00 | 0.00 | 0.00 | 0.00 | 0.00 | 0.00 | 0.00 | 0.00 | *Proteobacteria* | *Betaproteobacteria* | *Rhodocyclales* | *Rhodocyclaceae* | Transient-rare taxa |
| OTU 1558 | 0.00 | 0.03 | 0.00 | 0.00 | 0.00 | 0.00 | 0.00 | 0.00 | 0.00 | 0.00 | 0.00 | 0.00 | *Proteobacteria* | *Betaproteobacteria* | *Rhodocyclales* | *Rhodocyclaceae* | Transient-rare taxa |
| OTU 1559 | 0.00 | 0.02 | 0.00 | 0.00 | 0.00 | 0.00 | 0.00 | 0.00 | 0.01 | 0.00 | 0.00 | 0.00 | *Proteobacteria* | *Alphaproteobacteria* | *Rhodobacterales* | *Rhodobacteraceae* | Transient-rare taxa |
| OTU 1560 | 0.00 | 0.03 | 0.00 | 0.00 | 0.00 | 0.00 | 0.00 | 0.00 | 0.00 | 0.00 | 0.00 | 0.00 | *Planctomycetes* | *Planctomycetia* | *Candidatus Brocadiales* | *Candidatus Brocadiaceae* | Transient-rare taxa |
| OTU 1561 | 0.00 | 0.01 | 0.00 | 0.01 | 0.00 | 0.00 | 0.00 | 0.00 | 0.01 | 0.00 | 0.00 | 0.00 | *Proteobacteria* | *Betaproteobacteria* | *Rhodocyclales* | *Rhodocyclaceae* | Transient-rare taxa |
| OTU 1562 | 0.00 | 0.00 | 0.00 | 0.00 | 0.00 | 0.00 | 0.00 | 0.00 | 0.03 | 0.00 | 0.00 | 0.00 | *Proteobacteria* | *Deltaproteobacteria* | *Desulfovibrionales* | *Desulfovibrionaceae* | Transient-rare taxa |
| OTU 1563 | 0.00 | 0.00 | 0.00 | 0.00 | 0.00 | 0.00 | 0.00 | 0.02 | 0.00 | 0.00 | 0.01 | 0.00 | *Proteobacteria* | *Gammaproteobacteria* | *Xanthomonadales* | *Xanthomonadaceae* | Transient-rare taxa |
| OTU 1564 | 0.01 | 0.00 | 0.00 | 0.00 | 0.00 | 0.00 | 0.00 | 0.00 | 0.00 | 0.00 | 0.02 | 0.00 | *Bacteroidetes* | *Sphingobacteriia* | *Sphingobacteriales* | *Sphingobacteriaceae* | Transient-rare taxa |
| OTU 1565 | 0.00 | 0.00 | 0.00 | 0.00 | 0.00 | 0.00 | 0.00 | 0.00 | 0.01 | 0.00 | 0.00 | 0.02 | *Proteobacteria* | *Alphaproteobacteria* | *Rhizobiales* | *Hyphomicrobiaceae* | Transient-rare taxa |
| OTU 1566 | 0.00 | 0.02 | 0.00 | 0.00 | 0.00 | 0.00 | 0.00 | 0.01 | 0.00 | 0.00 | 0.00 | 0.00 | *Proteobacteria* | *Alphaproteobacteria* | *Rhizobiales* | *Methylocystaceae* | Transient-rare taxa |
| OTU 1567 | 0.01 | 0.02 | 0.00 | 0.00 | 0.00 | 0.00 | 0.00 | 0.00 | 0.00 | 0.00 | 0.00 | 0.00 | *Proteobacteria* | *Betaproteobacteria* | *Rhodocyclales* | *Rhodocyclaceae* | Transient-rare taxa |
| OTU 1568 | 0.00 | 0.00 | 0.02 | 0.00 | 0.00 | 0.00 | 0.00 | 0.00 | 0.00 | 0.01 | 0.00 | 0.00 | *Bacteroidetes* | *Sphingobacteriia* | *Sphingobacteriales* | *Sphingobacteriaceae* | Transient-rare taxa |
| OTU 1569 | 0.00 | 0.00 | 0.01 | 0.00 | 0.00 | 0.00 | 0.00 | 0.00 | 0.01 | 0.00 | 0.01 | 0.00 | *Proteobacteria* | *Betaproteobacteria* | *Burkholderiales* | *Comamonadaceae* | Transient-rare taxa |
| OTU 1570 | 0.00 | 0.00 | 0.02 | 0.00 | 0.00 | 0.00 | 0.00 | 0.00 | 0.01 | 0.00 | 0.00 | 0.00 | *Actinobacteria* | *Actinobacteria* | *Actinobacteridae* | *Actinomycetales* | Transient-rare taxa |
| OTU 1571 | 0.01 | 0.00 | 0.00 | 0.00 | 0.00 | 0.00 | 0.00 | 0.00 | 0.02 | 0.00 | 0.00 | 0.00 | *Proteobacteria* | *Alphaproteobacteria* | *Rhodobacterales* | *Rhodobacteraceae* | Transient-rare taxa |
| OTU 1572 | 0.01 | 0.00 | 0.00 | 0.00 | 0.00 | 0.00 | 0.00 | 0.00 | 0.01 | 0.00 | 0.00 | 0.01 | *Proteobacteria* | *Deltaproteobacteria* | *Myxococcales* | *Sorangiineae* | Transient-rare taxa |
| OTU 1573 | 0.00 | 0.00 | 0.00 | 0.01 | 0.00 | 0.01 | 0.00 | 0.00 | 0.00 | 0.00 | 0.01 | 0.00 | *Proteobacteria* | *Gammaproteobacteria* | *Methylococcales* | *Methylococcaceae* | Transient-rare taxa |
| OTU 1574 | 0.00 | 0.01 | 0.02 | 0.00 | 0.00 | 0.00 | 0.00 | 0.00 | 0.00 | 0.00 | 0.00 | 0.00 | *Proteobacteria* | *Alphaproteobacteria* | *Rhizobiales* | *Rhodobiaceae* | Transient-rare taxa |
| OTU 1575 | 0.01 | 0.00 | 0.01 | 0.00 | 0.00 | 0.00 | 0.00 | 0.00 | 0.00 | 0.00 | 0.01 | 0.00 | *Proteobacteria* | *Epsilonproteobacteria* | *Campylobacterales* | *Campylobacteraceae* | Transient-rare taxa |
| OTU 1576 | 0.03 | 0.00 | 0.00 | 0.00 | 0.00 | 0.00 | 0.00 | 0.00 | 0.00 | 0.00 | 0.00 | 0.00 | *Bacteroidetes* | *Sphingobacteriia* | *Sphingobacteriales* | *Saprospiraceae* | Transient-rare taxa |
| OTU 1577 | 0.01 | 0.00 | 0.00 | 0.00 | 0.00 | 0.00 | 0.00 | 0.00 | 0.00 | 0.01 | 0.00 | 0.01 | *Proteobacteria* | *Alphaproteobacteria* | *Rhizobiales* | *Methylobacteriaceae* | Transient-rare taxa |
| OTU 1578 | 0.00 | 0.01 | 0.00 | 0.00 | 0.00 | 0.00 | 0.01 | 0.00 | 0.01 | 0.00 | 0.00 | 0.00 | *Proteobacteria* | *Betaproteobacteria* | *Rhodocyclales* | *Rhodocyclaceae* | Transient-rare taxa |
| OTU 1579 | 0.00 | 0.00 | 0.00 | 0.00 | 0.00 | 0.00 | 0.00 | 0.00 | 0.02 | 0.00 | 0.01 | 0.00 | *Acidobacteria* | *Acidobacteria_Gp4* | *Gp4* | | Transient-rare taxa |
| OTU 1580 | 0.00 | 0.00 | 0.00 | 0.00 | 0.00 | 0.00 | 0.00 | 0.00 | 0.00 | 0.00 | 0.00 | 0.03 | *Proteobacteria* | *Alphaproteobacteria* | *Alphaproteobacteria_incertae_sedis* | *Rhizomicrobium* | Transient-rare taxa |
| OTU 1581 | 0.00 | 0.00 | 0.03 | 0.00 | 0.00 | 0.00 | 0.00 | 0.00 | 0.00 | 0.00 | 0.00 | 0.00 | *Proteobacteria* | *Deltaproteobacteria* | *Myxococcales* | *Sorangiineae* | Transient-rare taxa |
| OTU 1582 | 0.00 | 0.00 | 0.01 | 0.00 | 0.00 | 0.00 | 0.00 | 0.01 | 0.01 | 0.00 | 0.00 | 0.00 | *Chloroflexi* | *Anaerolineae* | *Anaerolineales* | *Anaerolineaceae* | Transient-rare taxa |
| OTU 1583 | 0.01 | 0.00 | 0.00 | 0.00 | 0.00 | 0.00 | 0.00 | 0.00 | 0.00 | 0.00 | 0.00 | 0.02 | *Proteobacteria* | *Betaproteobacteria* | *Rhodocyclales* | *Rhodocyclaceae* | Transient-rare taxa |
| OTU 1584 | 0.03 | 0.00 | 0.00 | 0.00 | 0.00 | 0.00 | 0.00 | 0.00 | 0.00 | 0.00 | 0.00 | 0.00 | *Proteobacteria* | *Epsilonproteobacteria* | *Nautiliales* | *Nautiliaceae* | Transient-rare taxa |
| OTU 1585 | 0.01 | 0.00 | 0.00 | 0.00 | 0.00 | 0.00 | 0.00 | 0.00 | 0.00 | 0.00 | 0.02 | 0.00 | *Proteobacteria* | *Gammaproteobacteria* | *Gammaproteobacteria_incertae_sedis* | *Umboniibacter* | Transient-rare taxa |
| OTU 1586 | 0.02 | 0.01 | 0.00 | 0.00 | 0.00 | 0.00 | 0.00 | 0.00 | 0.00 | 0.00 | 0.00 | 0.00 | *Proteobacteria* | *Betaproteobacteria* | *Burkholderiales* | *Comamonadaceae* | Transient-rare taxa |
| OTU 1587 | 0.00 | 0.00 | 0.00 | 0.00 | 0.00 | 0.01 | 0.00 | 0.00 | 0.02 | 0.00 | 0.00 | 0.00 | *Proteobacteria* | *Betaproteobacteria* | *Rhodocyclales* | *Rhodocyclaceae* | Transient-rare taxa |
| OTU 1588 | 0.00 | 0.01 | 0.00 | 0.00 | 0.00 | 0.00 | 0.02 | 0.00 | 0.00 | 0.00 | 0.00 | 0.00 | *Proteobacteria* | *Betaproteobacteria* | *Burkholderiales* | *Burkholderiales_incertae_sedis* | Transient-rare taxa |
| OTU 1589 | 0.00 | 0.01 | 0.01 | 0.00 | 0.00 | 0.01 | 0.00 | 0.00 | 0.00 | 0.00 | 0.00 | 0.00 | *Proteobacteria* | *Gammaproteobacteria* | *Aeromonadales* | *Aeromonadaceae* | Transient-rare taxa |
| OTU 1590 | 0.00 | 0.01 | 0.00 | 0.00 | 0.00 | 0.00 | 0.00 | 0.01 | 0.00 | 0.00 | 0.01 | 0.00 | *Proteobacteria* | *Betaproteobacteria* | *Rhodocyclales* | *Rhodocyclaceae* | Transient-rare taxa |
| OTU 1591 | 0.00 | 0.01 | 0.00 | 0.00 | 0.00 | 0.00 | 0.00 | 0.00 | 0.00 | 0.00 | 0.02 | 0.00 | *Proteobacteria* | *Deltaproteobacteria* | *Myxococcales* | *Nannocystineae* | Transient-rare taxa |
| OTU 1592 | 0.00 | 0.00 | 0.00 | 0.00 | 0.00 | 0.00 | 0.00 | 0.00 | 0.00 | 0.01 | 0.00 | 0.02 | *Bacteroidetes* | *Sphingobacteriia* | *Sphingobacteriales* | *Chitinophagaceae* | Transient-rare taxa |
| OTU 1593 | 0.00 | 0.00 | 0.01 | 0.00 | 0.00 | 0.00 | 0.01 | 0.00 | 0.01 | 0.00 | 0.00 | 0.00 | *Proteobacteria* | *Gammaproteobacteria* | *Thiotrichales* | *Thiotrichaceae* | Transient-rare taxa |
| OTU 1594 | 0.01 | 0.00 | 0.01 | 0.00 | 0.00 | 0.00 | 0.00 | 0.00 | 0.00 | 0.00 | 0.00 | 0.01 | *Proteobacteria* | *Gammaproteobacteria* | *Xanthomonadales* | *Sinobacteraceae* | Transient-rare taxa |
| OTU 1595 | 0.00 | 0.00 | 0.00 | 0.00 | 0.00 | 0.00 | 0.00 | 0.00 | 0.00 | 0.00 | 0.03 | 0.00 | *Firmicutes* | *Bacilli* | *Lactobacillales* | *Carnobacteriaceae* | Transient-rare taxa |
| OTU 1596 | 0.00 | 0.00 | 0.00 | 0.00 | 0.00 | 0.00 | 0.00 | 0.00 | 0.00 | 0.03 | 0.00 | 0.00 | *Verrucomicrobia* | *Opitutae* | *Opitutales* | *Opitutaceae* | Transient-rare taxa |
| OTU 1597 | 0.00 | 0.00 | 0.00 | 0.00 | 0.00 | 0.00 | 0.01 | 0.00 | 0.00 | 0.00 | 0.02 | 0.00 | *Acidobacteria* | *Acidobacteria_Gp4* | *Gp4* | | Transient-rare taxa |
| OTU 1598 | 0.01 | 0.00 | 0.00 | 0.00 | 0.00 | 0.00 | 0.00 | 0.00 | 0.00 | 0.00 | 0.02 | 0.00 | *Proteobacteria* | *Betaproteobacteria* | *Burkholderiales* | *Comamonadaceae* | Transient-rare taxa |
| OTU 1599 | 0.00 | 0.00 | 0.00 | 0.00 | 0.02 | 0.01 | 0.00 | 0.00 | 0.00 | 0.00 | 0.00 | 0.00 | *Firmicutes* | *Clostridia* | *Clostridiales* | *Clostridiales_Incertae Sedis XIII* | Transient-rare taxa |
| OTU 1600 | 0.00 | 0.00 | 0.00 | 0.00 | 0.00 | 0.01 | 0.01 | 0.01 | 0.00 | 0.00 | 0.00 | 0.00 | *Nitrospirae* | *Nitrospira* | *Nitrospirales* | *Nitrospiraceae* | Transient-rare taxa |
| OTU 1601 | 0.00 | 0.00 | 0.00 | 0.00 | 0.00 | 0.00 | 0.00 | 0.03 | 0.00 | 0.00 | 0.00 | 0.00 | *Bacteroidetes* | *Sphingobacteriia* | *Sphingobacteriales* | *Saprospiraceae* | Transient-rare taxa |
| OTU 1602 | 0.01 | 0.00 | 0.00 | 0.00 | 0.00 | 0.00 | 0.00 | 0.00 | 0.00 | 0.00 | 0.02 | 0.00 | *Proteobacteria* | *Betaproteobacteria* | *Rhodocyclales* | *Rhodocyclaceae* | Transient-rare taxa |
| OTU 1603 | 0.00 | 0.00 | 0.00 | 0.01 | 0.01 | 0.00 | 0.00 | 0.00 | 0.00 | 0.00 | 0.00 | 0.01 | *Proteobacteria* | *Betaproteobacteria* | *Rhodocyclales* | *Rhodocyclaceae* | Transient-rare taxa |
| OTU 1604 | 0.00 | 0.00 | 0.00 | 0.00 | 0.00 | 0.00 | 0.00 | 0.00 | 0.00 | 0.00 | 0.03 | 0.00 | *Proteobacteria* | *Alphaproteobacteria* | *Sphingomonadales* | *Sphingomonadaceae* | Transient-rare taxa |
| OTU 1605 | 0.00 | 0.00 | 0.00 | 0.00 | 0.01 | 0.01 | 0.00 | 0.00 | 0.00 | 0.00 | 0.01 | 0.00 | *Proteobacteria* | *Betaproteobacteria* | *Burkholderiales* | *Comamonadaceae* | Transient-rare taxa |
| OTU 1606 | 0.00 | 0.00 | 0.00 | 0.00 | 0.00 | 0.00 | 0.00 | 0.03 | 0.00 | 0.00 | 0.00 | 0.00 | *Planctomycetes* | *Planctomycetia* | *Planctomycetales* | *Planctomycetaceae* | Transient-rare taxa |
| OTU 1607 | 0.00 | 0.00 | 0.00 | 0.00 | 0.00 | 0.00 | 0.00 | 0.00 | 0.00 | 0.00 | 0.03 | 0.00 | *Proteobacteria* | *Betaproteobacteria* | *Burkholderiales* | *Comamonadaceae* | Transient-rare taxa |
| OTU 1608 | 0.01 | 0.00 | 0.00 | 0.00 | 0.00 | 0.00 | 0.00 | 0.00 | 0.00 | 0.01 | 0.01 | 0.00 | *Proteobacteria* | *Deltaproteobacteria* | *Myxococcales* | *Nannocystineae* | Transient-rare taxa |
| OTU 1609 | 0.00 | 0.00 | 0.00 | 0.00 | 0.00 | 0.00 | 0.00 | 0.00 | 0.00 | 0.00 | 0.03 | 0.00 | *Proteobacteria* | *Alphaproteobacteria* | *Rhizobiales* | *Phyllobacteriaceae* | Transient-rare taxa |
| OTU 1610 | 0.01 | 0.00 | 0.00 | 0.00 | 0.00 | 0.00 | 0.00 | 0.00 | 0.00 | 0.00 | 0.02 | 0.00 | *Proteobacteria* | *Betaproteobacteria* | *Rhodocyclales* | *Rhodocyclaceae* | Transient-rare taxa |
| OTU 1611 | 0.00 | 0.00 | 0.00 | 0.00 | 0.00 | 0.00 | 0.00 | 0.00 | 0.00 | 0.01 | 0.02 | 0.00 | *Bacteroidetes* | *Sphingobacteriia* | *Sphingobacteriales* | *Chitinophagaceae* | Transient-rare taxa |
| OTU 1612 | 0.00 | 0.00 | 0.00 | 0.00 | 0.02 | 0.01 | 0.00 | 0.00 | 0.00 | 0.00 | 0.00 | 0.00 | *Bacteroidetes* | *Flavobacteriia* | *Flavobacteriales* | *Flavobacteriaceae* | Transient-rare taxa |
| OTU 1613 | 0.00 | 0.00 | 0.00 | 0.00 | 0.02 | 0.00 | 0.01 | 0.00 | 0.00 | 0.00 | 0.00 | 0.00 | *Bacteroidetes* | *Flavobacteriia* | *Flavobacteriales* | *Flavobacteriaceae* | Transient-rare taxa |
| OTU 1614 | 0.01 | 0.00 | 0.00 | 0.00 | 0.00 | 0.00 | 0.00 | 0.00 | 0.00 | 0.00 | 0.02 | 0.00 | *Proteobacteria* | *Betaproteobacteria* | *Burkholderiales* | *Comamonadaceae* | Transient-rare taxa |
| OTU 1615 | 0.00 | 0.00 | 0.00 | 0.02 | 0.00 | 0.00 | 0.00 | 0.00 | 0.00 | 0.00 | 0.01 | 0.00 | *Proteobacteria* | *Gammaproteobacteria* | *Aeromonadales* | *Aeromonadaceae* | Transient-rare taxa |
| OTU 1616 | 0.00 | 0.01 | 0.00 | 0.00 | 0.00 | 0.01 | 0.01 | 0.00 | 0.00 | 0.00 | 0.00 | 0.00 | *Planctomycetes* | *Planctomycetia* | *Planctomycetales* | *Planctomycetaceae* | Transient-rare taxa |
| OTU 1617 | 0.02 | 0.00 | 0.00 | 0.00 | 0.00 | 0.00 | 0.00 | 0.01 | 0.00 | 0.00 | 0.00 | 0.00 | *candidate division WPS-2* | *WPS-2_genera_incertae_sedis* | | | Transient-rare taxa |
| OTU 1618 | 0.02 | 0.00 | 0.00 | 0.00 | 0.00 | 0.00 | 0.00 | 0.00 | 0.00 | 0.00 | 0.00 | 0.01 | *Bacteroidetes* | *Bacteroidia* | *Bacteroidales* | *Marinilabiliaceae* | Transient-rare taxa |
| OTU 1619 | 0.01 | 0.00 | 0.00 | 0.00 | 0.00 | 0.00 | 0.00 | 0.00 | 0.00 | 0.02 | 0.00 | 0.00 | *Proteobacteria* | *Alphaproteobacteria* | *Rhodobacterales* | *Rhodobacteraceae* | Transient-rare taxa |
| OTU 1620 | 0.00 | 0.00 | 0.00 | 0.00 | 0.00 | 0.00 | 0.00 | 0.00 | 0.00 | 0.00 | 0.03 | 0.00 | *Bacteroidetes* | *Flavobacteriia* | *Flavobacteriales* | *Flavobacteriaceae* | Transient-rare taxa |
| OTU 1621 | 0.00 | 0.00 | 0.00 | 0.00 | 0.00 | 0.00 | 0.00 | 0.01 | 0.00 | 0.00 | 0.02 | 0.00 | *Proteobacteria* | *Deltaproteobacteria* | *Myxococcales* | *Sorangiineae* | Transient-rare taxa |
| OTU 1622 | 0.01 | 0.00 | 0.00 | 0.00 | 0.00 | 0.00 | 0.00 | 0.00 | 0.00 | 0.02 | 0.00 | 0.00 | *Proteobacteria* | *Alphaproteobacteria* | *Sphingomonadales* | *Erythrobacteraceae* | Transient-rare taxa |
| OTU 1623 | 0.01 | 0.00 | 0.00 | 0.00 | 0.00 | 0.00 | 0.00 | 0.01 | 0.01 | 0.00 | 0.00 | 0.00 | *Proteobacteria* | *Betaproteobacteria* | *Burkholderiales* | *Comamonadaceae* | Transient-rare taxa |
| OTU 1624 | 0.00 | 0.00 | 0.00 | 0.00 | 0.02 | 0.00 | 0.00 | 0.00 | 0.00 | 0.01 | 0.00 | 0.00 | *Cloacimonetes* | *Candidatus Cloacamonas* | | | Transient-rare taxa |
| OTU 1625 | 0.00 | 0.00 | 0.00 | 0.00 | 0.00 | 0.00 | 0.00 | 0.02 | 0.00 | 0.00 | 0.01 | 0.00 | *Proteobacteria* | *Alphaproteobacteria* | *Rhodobacterales* | *Rhodobacteraceae* | Transient-rare taxa |
| OTU 1626 | 0.01 | 0.00 | 0.01 | 0.00 | 0.00 | 0.00 | 0.00 | 0.00 | 0.00 | 0.00 | 0.01 | 0.00 | *Proteobacteria* | *Gammaproteobacteria* | *Xanthomonadales* | *Xanthomonadaceae* | Transient-rare taxa |
| OTU 1627 | 0.03 | 0.00 | 0.00 | 0.00 | 0.00 | 0.00 | 0.00 | 0.00 | 0.00 | 0.00 | 0.00 | 0.00 | *Proteobacteria* | *Betaproteobacteria* | *Burkholderiales* | *Comamonadaceae* | Transient-rare taxa |
| OTU 1628 | 0.00 | 0.00 | 0.01 | 0.00 | 0.01 | 0.00 | 0.00 | 0.00 | 0.00 | 0.01 | 0.00 | 0.00 | *Bacteroidetes* | *Cytophagia* | *Cytophagales* | *Cytophagaceae* | Transient-rare taxa |
| OTU 1629 | 0.00 | 0.00 | 0.00 | 0.02 | 0.01 | 0.00 | 0.00 | 0.00 | 0.00 | 0.00 | 0.00 | 0.00 | *Proteobacteria* | *Epsilonproteobacteria* | *Campylobacterales* | *Campylobacteraceae* | Transient-rare taxa |
| OTU 1630 | 0.00 | 0.00 | 0.00 | 0.00 | 0.00 | 0.00 | 0.00 | 0.00 | 0.00 | 0.00 | 0.03 | 0.00 | *Bacteroidetes* | *Flavobacteriia* | *Flavobacteriales* | *Flavobacteriaceae* | Transient-rare taxa |
| OTU 1631 | 0.00 | 0.00 | 0.00 | 0.00 | 0.00 | 0.00 | 0.00 | 0.00 | 0.01 | 0.01 | 0.00 | 0.01 | *Proteobacteria* | *Alphaproteobacteria* | *Rhizobiales* | *Methylobacteriaceae* | Transient-rare taxa |
| OTU 1632 | 0.00 | 0.00 | 0.00 | 0.00 | 0.01 | 0.00 | 0.00 | 0.00 | 0.02 | 0.00 | 0.00 | 0.00 | *Bacteroidetes* | *Cytophagia* | *Cytophagales* | *Cytophagaceae* | Transient-rare taxa |
| OTU 1633 | 0.00 | 0.00 | 0.00 | 0.00 | 0.00 | 0.00 | 0.00 | 0.00 | 0.00 | 0.00 | 0.01 | 0.02 | *Bacteroidetes* | *Sphingobacteriia* | *Sphingobacteriales* | *Chitinophagaceae* | Transient-rare taxa |
| OTU 1634 | 0.01 | 0.01 | 0.00 | 0.00 | 0.00 | 0.00 | 0.00 | 0.00 | 0.00 | 0.00 | 0.01 | 0.00 | *Proteobacteria* | *Betaproteobacteria* | *Burkholderiales* | *Comamonadaceae* | Transient-rare taxa |
| OTU 1635 | 0.00 | 0.00 | 0.00 | 0.00 | 0.00 | 0.00 | 0.00 | 0.00 | 0.00 | 0.03 | 0.00 | 0.00 | *Firmicutes* | *Clostridia* | *Clostridiales* | *Peptostreptococcaceae* | Transient-rare taxa |
| OTU 1636 | 0.00 | 0.00 | 0.00 | 0.03 | 0.00 | 0.00 | 0.00 | 0.00 | 0.00 | 0.00 | 0.00 | 0.00 | *Proteobacteria* | *Epsilonproteobacteria* | *Campylobacterales* | *Campylobacteraceae* | Transient-rare taxa |
| OTU 1637 | 0.00 | 0.00 | 0.00 | 0.03 | 0.00 | 0.00 | 0.00 | 0.00 | 0.00 | 0.00 | 0.00 | 0.00 | *Proteobacteria* | *Gammaproteobacteria* | *Pseudomonadales* | *Moraxellaceae* | Transient-rare taxa |
| OTU 1638 | 0.00 | 0.00 | 0.00 | 0.01 | 0.02 | 0.00 | 0.00 | 0.00 | 0.00 | 0.00 | 0.00 | 0.00 | *Firmicutes* | *Clostridia* | *Clostridiales* | *Lachnospiraceae* | Transient-rare taxa |
| OTU 1639 | 0.00 | 0.01 | 0.00 | 0.00 | 0.00 | 0.00 | 0.00 | 0.00 | 0.00 | 0.00 | 0.00 | 0.02 | *Proteobacteria* | *Betaproteobacteria* | *Rhodocyclales* | *Rhodocyclaceae* | Transient-rare taxa |
| OTU 1640 | 0.00 | 0.00 | 0.01 | 0.00 | 0.00 | 0.00 | 0.00 | 0.02 | 0.00 | 0.00 | 0.00 | 0.00 | *Proteobacteria* | *Deltaproteobacteria* | *Myxococcales* | *Nannocystineae* | Transient-rare taxa |
| OTU 1641 | 0.00 | 0.00 | 0.00 | 0.00 | 0.01 | 0.00 | 0.00 | 0.00 | 0.00 | 0.00 | 0.00 | 0.02 | *Proteobacteria* | *Betaproteobacteria* | *Burkholderiales* | *Burkholderiales_incertae_sedis* | Transient-rare taxa |
| OTU 1642 | 0.00 | 0.00 | 0.00 | 0.00 | 0.00 | 0.00 | 0.03 | 0.00 | 0.00 | 0.00 | 0.00 | 0.00 | *Proteobacteria* | *Gammaproteobacteria* | *Xanthomonadales* | *Xanthomonadaceae* | Transient-rare taxa |
| OTU 1643 | 0.00 | 0.00 | 0.00 | 0.00 | 0.00 | 0.00 | 0.03 | 0.00 | 0.00 | 0.00 | 0.00 | 0.00 | *Proteobacteria* | *Gammaproteobacteria* | *Candidatus Carsonella* | | Transient-rare taxa |
| OTU 1644 | 0.00 | 0.00 | 0.01 | 0.01 | 0.01 | 0.00 | 0.00 | 0.00 | 0.00 | 0.00 | 0.00 | 0.00 | *Proteobacteria* | *Deltaproteobacteria* | *Bdellovibrionales* | *Bdellovibrionaceae* | Transient-rare taxa |
| OTU 1645 | 0.00 | 0.00 | 0.00 | 0.00 | 0.00 | 0.00 | 0.00 | 0.00 | 0.00 | 0.01 | 0.01 | 0.01 | *Proteobacteria* | *Alphaproteobacteria* | *Sphingomonadales* | *Sphingomonadaceae* | Transient-rare taxa |
| OTU 1646 | 0.00 | 0.00 | 0.00 | 0.00 | 0.00 | 0.00 | 0.03 | 0.00 | 0.00 | 0.00 | 0.00 | 0.00 | *Bacteroidetes* | *Flavobacteriia* | *Flavobacteriales* | *Flavobacteriaceae* | Transient-rare taxa |
| OTU 1647 | 0.00 | 0.01 | 0.00 | 0.00 | 0.00 | 0.00 | 0.00 | 0.02 | 0.00 | 0.00 | 0.00 | 0.00 | *Proteobacteria* | *Gammaproteobacteria* | *Xanthomonadales* | *Xanthomonadaceae* | Transient-rare taxa |
| OTU 1648 | 0.00 | 0.00 | 0.00 | 0.02 | 0.01 | 0.00 | 0.00 | 0.00 | 0.00 | 0.00 | 0.00 | 0.00 | *Bacteroidetes* | *Sphingobacteriia* | *Sphingobacteriales* | *Saprospiraceae* | Transient-rare taxa |
| OTU 1649 | 0.01 | 0.00 | 0.00 | 0.01 | 0.00 | 0.00 | 0.00 | 0.00 | 0.00 | 0.00 | 0.00 | 0.01 | *Proteobacteria* | *Epsilonproteobacteria* | *Campylobacterales* | *Helicobacteraceae* | Transient-rare taxa |
| OTU 1650 | 0.00 | 0.01 | 0.00 | 0.00 | 0.00 | 0.00 | 0.00 | 0.00 | 0.00 | 0.00 | 0.01 | 0.01 | *Bacteroidetes* | *Sphingobacteriia* | *Sphingobacteriales* | *Saprospiraceae* | Transient-rare taxa |
| OTU 1651 | 0.00 | 0.00 | 0.00 | 0.02 | 0.00 | 0.01 | 0.00 | 0.00 | 0.00 | 0.00 | 0.00 | 0.00 | *Tenericutes* | *Mollicutes* | *Haloplasmatales* | *Haloplasmataceae* | Transient-rare taxa |
| OTU 1652 | 0.00 | 0.00 | 0.00 | 0.01 | 0.00 | 0.01 | 0.00 | 0.00 | 0.00 | 0.00 | 0.00 | 0.01 | *Chloroflexi* | *Anaerolineae* | *Anaerolineales* | *Anaerolineaceae* | Transient-rare taxa |
| OTU 1653 | 0.00 | 0.00 | 0.00 | 0.00 | 0.00 | 0.00 | 0.00 | 0.00 | 0.00 | 0.00 | 0.03 | 0.00 | *Proteobacteria* | *Betaproteobacteria* | *Rhodocyclales* | *Rhodocyclaceae* | Transient-rare taxa |
| OTU 1654 | 0.00 | 0.00 | 0.00 | 0.00 | 0.00 | 0.00 | 0.00 | 0.00 | 0.00 | 0.00 | 0.00 | 0.03 | *Firmicutes* | *Clostridia* | *Clostridiales* | *Clostridiales_Incertae Sedis XII* | Transient-rare taxa |
| OTU 1655 | 0.00 | 0.00 | 0.02 | 0.01 | 0.00 | 0.00 | 0.00 | 0.00 | 0.00 | 0.00 | 0.00 | 0.00 | *Bacteroidetes* | *Sphingobacteriia* | *Sphingobacteriales* | *Saprospiraceae* | Transient-rare taxa |
| OTU 1656 | 0.00 | 0.00 | 0.00 | 0.00 | 0.00 | 0.00 | 0.03 | 0.00 | 0.00 | 0.00 | 0.00 | 0.00 | *Proteobacteria* | *Gammaproteobacteria* | *Chromatiales* | *Ectothiorhodospiraceae* | Transient-rare taxa |
| OTU 1657 | 0.00 | 0.00 | 0.00 | 0.00 | 0.00 | 0.00 | 0.00 | 0.00 | 0.01 | 0.00 | 0.00 | 0.02 | *Proteobacteria* | *Betaproteobacteria* | *Rhodocyclales* | *Rhodocyclaceae* | Transient-rare taxa |
| OTU 1658 | 0.00 | 0.00 | 0.00 | 0.00 | 0.00 | 0.03 | 0.00 | 0.00 | 0.00 | 0.00 | 0.00 | 0.00 | *Proteobacteria* | *Betaproteobacteria* | *Burkholderiales* | *Alcaligenaceae* | Transient-rare taxa |
| OTU 1659 | 0.00 | 0.00 | 0.00 | 0.00 | 0.00 | 0.03 | 0.00 | 0.00 | 0.00 | 0.00 | 0.00 | 0.00 | *Actinobacteria* | *Actinobacteria* | *Acidimicrobidae* | *Acidimicrobiales* | Transient-rare taxa |
| OTU 1660 | 0.00 | 0.00 | 0.00 | 0.00 | 0.01 | 0.02 | 0.00 | 0.00 | 0.00 | 0.00 | 0.00 | 0.00 | *Chloroflexi* | *Anaerolineae* | *Anaerolineales* | *Anaerolineaceae* | Transient-rare taxa |
| OTU 1661 | 0.00 | 0.00 | 0.01 | 0.00 | 0.00 | 0.01 | 0.00 | 0.01 | 0.00 | 0.00 | 0.00 | 0.00 | *Bacteroidetes* | *Bacteroidia* | *Bacteroidales* | *Bacteroidaceae* | Transient-rare taxa |
| OTU 1662 | 0.00 | 0.00 | 0.03 | 0.00 | 0.00 | 0.00 | 0.00 | 0.00 | 0.00 | 0.00 | 0.00 | 0.00 | *Verrucomicrobia* | *Opitutae* | *Puniceicoccales* | *Puniceicoccaceae* | Transient-rare taxa |
| OTU 1663 | 0.00 | 0.00 | 0.00 | 0.00 | 0.03 | 0.00 | 0.00 | 0.00 | 0.00 | 0.00 | 0.00 | 0.00 | *Bacteroidetes* | *Bacteroidetes_incertae_sedis* | *Prolixibacter* | | Transient-rare taxa |
| OTU 1664 | 0.00 | 0.00 | 0.00 | 0.00 | 0.00 | 0.01 | 0.00 | 0.00 | 0.00 | 0.00 | 0.00 | 0.02 | *Bacteroidetes* | *Sphingobacteriia* | *Sphingobacteriales* | *Chitinophagaceae* | Transient-rare taxa |
| OTU 1665 | 0.00 | 0.00 | 0.00 | 0.01 | 0.00 | 0.02 | 0.00 | 0.00 | 0.00 | 0.00 | 0.00 | 0.00 | *Proteobacteria* | *Gammaproteobacteria* | *Gammaproteobacteria_incertae_sedis* | *Thiohalobacter* | Transient-rare taxa |
| OTU 1666 | 0.00 | 0.00 | 0.00 | 0.02 | 0.01 | 0.00 | 0.00 | 0.00 | 0.00 | 0.00 | 0.00 | 0.00 | *Proteobacteria* | *Betaproteobacteria* | *Burkholderiales* | *Comamonadaceae* | Transient-rare taxa |
| OTU 1667 | 0.00 | 0.00 | 0.00 | 0.01 | 0.00 | 0.00 | 0.02 | 0.00 | 0.00 | 0.00 | 0.00 | 0.00 | *Proteobacteria* | *Alphaproteobacteria* | *Rhizobiales* | *Hyphomicrobiaceae* | Transient-rare taxa |
| OTU 1668 | 0.00 | 0.00 | 0.00 | 0.00 | 0.00 | 0.00 | 0.00 | 0.00 | 0.00 | 0.00 | 0.00 | 0.03 | *Proteobacteria* | *Alphaproteobacteria* | *Rhodospirillales* | *Rhodospirillaceae* | Transient-rare taxa |
| OTU 1669 | 0.00 | 0.00 | 0.00 | 0.00 | 0.01 | 0.02 | 0.00 | 0.00 | 0.00 | 0.00 | 0.00 | 0.00 | *Bacteroidetes* | *Cytophagia* | *Cytophagales* | *Flammeovirgaceae* | Transient-rare taxa |
| OTU 1670 | 0.01 | 0.00 | 0.00 | 0.01 | 0.00 | 0.00 | 0.01 | 0.00 | 0.00 | 0.00 | 0.00 | 0.00 | *Bacteroidetes* | *Sphingobacteriia* | *Sphingobacteriales* | *Cyclobacteriaceae* | Transient-rare taxa |
| OTU 1671 | 0.00 | 0.00 | 0.03 | 0.00 | 0.00 | 0.00 | 0.00 | 0.00 | 0.00 | 0.00 | 0.00 | 0.00 | *Planctomycetes* | *Planctomycetia* | *Planctomycetales* | *Planctomycetaceae* | Transient-rare taxa |
| OTU 1672 | 0.00 | 0.00 | 0.00 | 0.00 | 0.01 | 0.00 | 0.00 | 0.00 | 0.00 | 0.00 | 0.00 | 0.02 | *Proteobacteria* | *Betaproteobacteria* | *Rhodocyclales* | *Rhodocyclaceae* | Transient-rare taxa |
| OTU 1673 | 0.00 | 0.00 | 0.00 | 0.01 | 0.00 | 0.02 | 0.00 | 0.00 | 0.00 | 0.00 | 0.00 | 0.00 | *Proteobacteria* | *Alphaproteobacteria* | *Rhodobacterales* | *Rhodobacteraceae* | Transient-rare taxa |
| OTU 1674 | 0.00 | 0.00 | 0.00 | 0.00 | 0.00 | 0.03 | 0.00 | 0.00 | 0.00 | 0.00 | 0.00 | 0.00 | *Planctomycetes* | *Planctomycetia* | *Planctomycetales* | *Planctomycetaceae* | Transient-rare taxa |
| OTU 1675 | 0.01 | 0.00 | 0.00 | 0.00 | 0.00 | 0.00 | 0.00 | 0.00 | 0.00 | 0.00 | 0.00 | 0.02 | *Proteobacteria* | *Betaproteobacteria* | *Burkholderiales* | *Burkholderiaceae* | Transient-rare taxa |
| OTU 1676 | 0.00 | 0.00 | 0.02 | 0.00 | 0.00 | 0.00 | 0.00 | 0.00 | 0.00 | 0.00 | 0.00 | 0.01 | *Synergistetes* | *Synergistia* | *Synergistales* | *Synergistaceae* | Transient-rare taxa |
| OTU 1677 | 0.00 | 0.00 | 0.00 | 0.00 | 0.00 | 0.03 | 0.00 | 0.00 | 0.00 | 0.00 | 0.00 | 0.00 | *Proteobacteria* | *Betaproteobacteria* | *Burkholderiales* | *Alcaligenaceae* | Transient-rare taxa |
| OTU 1678 | 0.00 | 0.00 | 0.03 | 0.00 | 0.00 | 0.00 | 0.00 | 0.00 | 0.00 | 0.00 | 0.00 | 0.00 | *Proteobacteria* | *Betaproteobacteria* | *Nitrosomonadales* | *Nitrosomonadaceae* | Transient-rare taxa |
| OTU 1679 | 0.00 | 0.00 | 0.03 | 0.00 | 0.00 | 0.00 | 0.00 | 0.00 | 0.00 | 0.00 | 0.00 | 0.00 | *Proteobacteria* | *Betaproteobacteria* | *Hydrogenophilales* | *Hydrogenophilaceae* | Transient-rare taxa |
| OTU 1680 | 0.00 | 0.00 | 0.02 | 0.00 | 0.00 | 0.00 | 0.00 | 0.00 | 0.01 | 0.00 | 0.00 | 0.00 | *Proteobacteria* | *Betaproteobacteria* | *Rhodocyclales* | *Rhodocyclaceae* | Transient-rare taxa |
| OTU 1681 | 0.00 | 0.00 | 0.01 | 0.00 | 0.00 | 0.00 | 0.00 | 0.00 | 0.01 | 0.01 | 0.00 | 0.00 | *Bacteroidetes* | *Sphingobacteriia* | *Sphingobacteriales* | *Chitinophagaceae* | Transient-rare taxa |
| OTU 1682 | 0.00 | 0.00 | 0.00 | 0.00 | 0.00 | 0.03 | 0.00 | 0.00 | 0.00 | 0.00 | 0.00 | 0.00 | *Proteobacteria* | *Alphaproteobacteria* | *Rhizobiales* | *Phyllobacteriaceae* | Transient-rare taxa |
| OTU 1683 | 0.00 | 0.00 | 0.02 | 0.00 | 0.00 | 0.00 | 0.00 | 0.01 | 0.00 | 0.00 | 0.00 | 0.00 | *Proteobacteria* | *Gammaproteobacteria* | *Pseudomonadales* | *Pseudomonadaceae* | Transient-rare taxa |
| OTU 1684 | 0.00 | 0.00 | 0.00 | 0.00 | 0.01 | 0.02 | 0.00 | 0.00 | 0.00 | 0.00 | 0.00 | 0.00 | *Bacteroidetes* | *Flavobacteriia* | *Flavobacteriales* | *Flavobacteriaceae* | Transient-rare taxa |
| OTU 1685 | 0.01 | 0.00 | 0.00 | 0.00 | 0.00 | 0.00 | 0.00 | 0.00 | 0.00 | 0.00 | 0.00 | 0.02 | *Proteobacteria* | *Alphaproteobacteria* | *Rhodobacterales* | *Rhodobacteraceae* | Transient-rare taxa |
| OTU 1686 | 0.01 | 0.00 | 0.00 | 0.00 | 0.00 | 0.00 | 0.00 | 0.01 | 0.00 | 0.00 | 0.00 | 0.01 | *Chloroflexi* | *Anaerolineae* | *Anaerolineales* | *Anaerolineaceae* | Transient-rare taxa |
| OTU 1687 | 0.00 | 0.00 | 0.01 | 0.00 | 0.00 | 0.00 | 0.00 | 0.00 | 0.00 | 0.00 | 0.00 | 0.02 | *Proteobacteria* | *Betaproteobacteria* | *Burkholderiales* | *Comamonadaceae* | Transient-rare taxa |
| OTU 1688 | 0.00 | 0.00 | 0.03 | 0.00 | 0.00 | 0.00 | 0.00 | 0.00 | 0.00 | 0.00 | 0.00 | 0.00 | *Marinimicrobia* | *Marinimicrobia_genera_incertae_sedis* | | | Transient-rare taxa |
| OTU 1689 | 0.00 | 0.00 | 0.03 | 0.00 | 0.00 | 0.00 | 0.00 | 0.00 | 0.00 | 0.00 | 0.00 | 0.00 | *Proteobacteria* | *Alphaproteobacteria* | *Rickettsiales* | *Rickettsiaceae* | Transient-rare taxa |
| OTU 1690 | 0.00 | 0.00 | 0.00 | 0.00 | 0.00 | 0.00 | 0.00 | 0.00 | 0.00 | 0.03 | 0.00 | 0.00 | *Bacteroidetes* | *Cytophagia* | *Cytophagales* | *Cytophagaceae* | Transient-rare taxa |
| OTU 1691 | 0.00 | 0.00 | 0.00 | 0.00 | 0.00 | 0.00 | 0.00 | 0.00 | 0.00 | 0.00 | 0.00 | 0.03 | *Proteobacteria* | *Alphaproteobacteria* | *Sphingomonadales* | *Sphingomonadaceae* | Transient-rare taxa |
| OTU 1692 | 0.00 | 0.00 | 0.00 | 0.00 | 0.00 | 0.00 | 0.00 | 0.00 | 0.00 | 0.03 | 0.00 | 0.00 | *Aquificae* | *Aquificae* | *Aquificales* | *Aquificaceae* | Transient-rare taxa |
| OTU 1693 | 0.01 | 0.00 | 0.00 | 0.00 | 0.00 | 0.00 | 0.00 | 0.00 | 0.01 | 0.00 | 0.00 | 0.00 | *Bacteroidetes* | *Sphingobacteriia* | *Sphingobacteriales* | *Chitinophagaceae* | Transient-rare taxa |
| OTU 1694 | 0.00 | 0.00 | 0.00 | 0.00 | 0.00 | 0.00 | 0.00 | 0.01 | 0.01 | 0.00 | 0.00 | 0.00 | *Bacteroidetes* | *Sphingobacteriia* | *Sphingobacteriales* | *Saprospiraceae* | Transient-rare taxa |
| OTU 1695 | 0.00 | 0.01 | 0.00 | 0.00 | 0.00 | 0.00 | 0.00 | 0.00 | 0.01 | 0.00 | 0.00 | 0.00 | *Proteobacteria* | *Alphaproteobacteria* | *Rhodobacterales* | *Rhodobacteraceae* | Transient-rare taxa |
| OTU 1696 | 0.00 | 0.00 | 0.00 | 0.00 | 0.00 | 0.00 | 0.00 | 0.00 | 0.02 | 0.00 | 0.00 | 0.00 | *Bacteroidetes* | *Flavobacteriia* | *Flavobacteriales* | *Flavobacteriaceae* | Transient-rare taxa |

| | | | | | | | | | | | | | | | | |
|---|---|---|---|---|---|---|---|---|---|---|---|---|---|---|---|---|
| OTU 1697 | 0.00 | 0.00 | 0.00 | 0.00 | 0.00 | 0.00 | 0.00 | 0.00 | 0.02 | 0.00 | 0.00 | 0.00 | Planctomycetes | Planctomycetia | Planctomycetales | Planctomycetaceae | Transient-rare taxa |
| OTU 1698 | 0.00 | 0.00 | 0.00 | 0.00 | 0.00 | 0.00 | 0.00 | 0.01 | 0.01 | 0.00 | 0.00 | 0.00 | Bacteroidetes | Sphingobacteriia | Sphingobacteriales | Sphingobacteriaceae | Transient-rare taxa |
| OTU 1699 | 0.00 | 0.00 | 0.01 | 0.00 | 0.00 | 0.00 | 0.01 | 0.00 | 0.00 | 0.00 | 0.00 | 0.00 | Bacteroidetes | Flavobacteriia | Flavobacteriales | Cryomorphaceae | Transient-rare taxa |
| OTU 1700 | 0.00 | 0.00 | 0.01 | 0.01 | 0.00 | 0.00 | 0.00 | 0.00 | 0.00 | 0.00 | 0.00 | 0.00 | Proteobacteria | Gammaproteobacteria | Alteromonadales | Maricurvus | Transient-rare taxa |
| OTU 1701 | 0.00 | 0.00 | 0.02 | 0.00 | 0.00 | 0.00 | 0.00 | 0.00 | 0.00 | 0.00 | 0.00 | 0.00 | Proteobacteria | Alphaproteobacteria | Rhodobacterales | Rhodobacteraceae | Transient-rare taxa |
| OTU 1702 | 0.00 | 0.00 | 0.02 | 0.00 | 0.00 | 0.00 | 0.00 | 0.00 | 0.00 | 0.00 | 0.00 | 0.00 | Proteobacteria | Gammaproteobacteria | Thiotrichales | Francisellaceae | Transient-rare taxa |
| OTU 1703 | 0.00 | 0.00 | 0.02 | 0.00 | 0.00 | 0.00 | 0.00 | 0.00 | 0.00 | 0.00 | 0.00 | 0.00 | Bacteroidetes | Cytophagia | Cytophagales | Cytophagaceae | Transient-rare taxa |
| OTU 1704 | 0.00 | 0.00 | 0.02 | 0.00 | 0.00 | 0.00 | 0.00 | 0.00 | 0.00 | 0.00 | 0.00 | 0.00 | Proteobacteria | Alphaproteobacteria | Rhodobacterales | Rhodobacteraceae | Transient-rare taxa |
| OTU 1705 | 0.00 | 0.00 | 0.02 | 0.00 | 0.00 | 0.00 | 0.00 | 0.00 | 0.00 | 0.00 | 0.00 | 0.00 | Proteobacteria | Alphaproteobacteria | Rickettsiales | Rickettsiaceae | Transient-rare taxa |
| OTU 1706 | 0.00 | 0.00 | 0.00 | 0.00 | 0.01 | 0.00 | 0.01 | 0.00 | 0.00 | 0.00 | 0.00 | 0.00 | Proteobacteria | Gammaproteobacteria | Alteromonadales | Alteromonadaceae | Transient-rare taxa |
| OTU 1707 | 0.00 | 0.00 | 0.00 | 0.00 | 0.00 | 0.00 | 0.02 | 0.00 | 0.00 | 0.00 | 0.00 | 0.00 | Proteobacteria | Betaproteobacteria | Burkholderiales | Burkholderiales_incertae_sedis | Transient-rare taxa |
| OTU 1708 | 0.00 | 0.00 | 0.00 | 0.00 | 0.00 | 0.00 | 0.02 | 0.00 | 0.00 | 0.00 | 0.00 | 0.00 | Nitrospirae | Nitrospira | Nitrospirales | Nitrospiraceae | Transient-rare taxa |
| OTU 1709 | 0.00 | 0.00 | 0.00 | 0.00 | 0.00 | 0.00 | 0.01 | 0.00 | 0.01 | 0.00 | 0.00 | 0.00 | Chloroflexi | Anaerolineae | Anaerolineales | Anaerolineaceae | Transient-rare taxa |
| OTU 1710 | 0.00 | 0.00 | 0.00 | 0.00 | 0.00 | 0.01 | 0.01 | 0.00 | 0.00 | 0.00 | 0.00 | 0.00 | Bacteroidetes | Flavobacteriia | Flavobacteriales | Cryomorphaceae | Transient-rare taxa |
| OTU 1711 | 0.00 | 0.00 | 0.00 | 0.00 | 0.00 | 0.00 | 0.02 | 0.00 | 0.00 | 0.00 | 0.00 | 0.00 | Bacteroidetes | Sphingobacteriia | Sphingobacteriales | Saprospiraceae | Transient-rare taxa |
| OTU 1712 | 0.00 | 0.00 | 0.00 | 0.00 | 0.00 | 0.00 | 0.02 | 0.00 | 0.00 | 0.00 | 0.00 | 0.00 | Proteobacteria | Deltaproteobacteria | Myxococcales | Sorangiineae | Transient-rare taxa |
| OTU 1713 | 0.00 | 0.00 | 0.00 | 0.00 | 0.02 | 0.00 | 0.00 | 0.00 | 0.00 | 0.00 | 0.00 | 0.00 | Proteobacteria | Deltaproteobacteria | Myxococcales | Sorangiineae | Transient-rare taxa |
| OTU 1714 | 0.00 | 0.00 | 0.00 | 0.00 | 0.00 | 0.00 | 0.00 | 0.00 | 0.01 | 0.00 | 0.01 | 0.00 | Parcubacteria | Parcubacteria_genera_incertae_sedis | | | Transient-rare taxa |
| OTU 1715 | 0.00 | 0.00 | 0.00 | 0.00 | 0.02 | 0.00 | 0.00 | 0.00 | 0.00 | 0.00 | 0.00 | 0.00 | Proteobacteria | Betaproteobacteria | Burkholderiales | Alcaligenaceae | Transient-rare taxa |
| OTU 1716 | 0.00 | 0.00 | 0.00 | 0.00 | 0.00 | 0.00 | 0.00 | 0.00 | 0.02 | 0.00 | 0.00 | 0.00 | Bacteroidetes | Sphingobacteriia | Sphingobacteriales | Saprospiraceae | Transient-rare taxa |
| OTU 1717 | 0.00 | 0.00 | 0.00 | 0.00 | 0.02 | 0.00 | 0.00 | 0.00 | 0.00 | 0.00 | 0.00 | 0.00 | Chloroflexi | Caldilineae | Caldilineales | Caldilineaceae | Transient-rare taxa |
| OTU 1718 | 0.00 | 0.02 | 0.00 | 0.00 | 0.00 | 0.00 | 0.00 | 0.00 | 0.00 | 0.00 | 0.00 | 0.00 | Proteobacteria | Deltaproteobacteria | Myxococcales | Cystobacterineae | Transient-rare taxa |
| OTU 1719 | 0.00 | 0.01 | 0.00 | 0.00 | 0.01 | 0.00 | 0.00 | 0.00 | 0.00 | 0.00 | 0.00 | 0.00 | Proteobacteria | Betaproteobacteria | Rhodocyclales | Rhodocyclaceae | Transient-rare taxa |
| OTU 1720 | 0.00 | 0.00 | 0.00 | 0.01 | 0.00 | 0.00 | 0.01 | 0.00 | 0.00 | 0.00 | 0.00 | 0.00 | Proteobacteria | Alphaproteobacteria | Rhizobiales | Methylocystaceae | Transient-rare taxa |
| OTU 1721 | 0.00 | 0.00 | 0.00 | 0.00 | 0.00 | 0.00 | 0.02 | 0.00 | 0.00 | 0.00 | 0.00 | 0.00 | Firmicutes | Clostridia | Clostridiales | Ruminococcaceae | Transient-rare taxa |
| OTU 1722 | 0.01 | 0.01 | 0.00 | 0.00 | 0.00 | 0.00 | 0.00 | 0.00 | 0.00 | 0.00 | 0.00 | 0.00 | Proteobacteria | Gammaproteobacteria | Chromatiales | Chromatiaceae | Transient-rare taxa |
| OTU 1723 | 0.00 | 0.00 | 0.00 | 0.00 | 0.01 | 0.00 | 0.00 | 0.00 | 0.00 | 0.00 | 0.01 | 0.00 | Proteobacteria | Betaproteobacteria | Rhodocyclales | Rhodocyclaceae | Transient-rare taxa |
| OTU 1724 | 0.00 | 0.00 | 0.00 | 0.00 | 0.02 | 0.00 | 0.00 | 0.00 | 0.00 | 0.00 | 0.00 | 0.00 | Proteobacteria | Deltaproteobacteria | Desulfobacterales | Desulfobacteraceae | Transient-rare taxa |
| OTU 1725 | 0.00 | 0.02 | 0.00 | 0.00 | 0.00 | 0.00 | 0.00 | 0.00 | 0.00 | 0.00 | 0.00 | 0.00 | Proteobacteria | Gammaproteobacteria | Chromatiales | Chromatiaceae | Transient-rare taxa |
| OTU 1726 | 0.00 | 0.00 | 0.00 | 0.00 | 0.02 | 0.00 | 0.00 | 0.00 | 0.00 | 0.00 | 0.00 | 0.00 | Bacteroidetes | Flavobacteriia | Flavobacteriales | Flavobacteriaceae | Transient-rare taxa |
| OTU 1727 | 0.00 | 0.00 | 0.00 | 0.00 | 0.02 | 0.00 | 0.00 | 0.00 | 0.00 | 0.00 | 0.00 | 0.00 | Bacteroidetes | Bacteroidia | Bacteroidales | Porphyromonadaceae | Transient-rare taxa |
| OTU 1728 | 0.00 | 0.00 | 0.00 | 0.00 | 0.00 | 0.00 | 0.01 | 0.00 | 0.01 | 0.00 | 0.00 | 0.00 | Deferribacteres | Deferribacteres | Deferribacterales | Deferribacterales_incertae_sedis | Transient-rare taxa |
| OTU 1729 | 0.00 | 0.01 | 0.00 | 0.00 | 0.00 | 0.00 | 0.00 | 0.01 | 0.00 | 0.00 | 0.00 | 0.00 | Bacteroidetes | Bacteroidetes_incertae_sedis | Ohtaekwangia | | Transient-rare taxa |
| OTU 1730 | 0.00 | 0.00 | 0.00 | 0.00 | 0.00 | 0.00 | 0.02 | 0.00 | 0.00 | 0.00 | 0.00 | 0.00 | Proteobacteria | Gammaproteobacteria | Legionellales | Coxiellaceae | Transient-rare taxa |
| OTU 1731 | 0.00 | 0.02 | 0.00 | 0.00 | 0.00 | 0.00 | 0.00 | 0.00 | 0.00 | 0.00 | 0.00 | 0.00 | Bacteroidetes | Sphingobacteriia | Sphingobacteriales | Saprospiraceae | Transient-rare taxa |
| OTU 1732 | 0.00 | 0.02 | 0.00 | 0.00 | 0.00 | 0.00 | 0.00 | 0.00 | 0.00 | 0.00 | 0.00 | 0.00 | Proteobacteria | Betaproteobacteria | Rhodocyclales | Rhodocyclaceae | Transient-rare taxa |
| OTU 1733 | 0.00 | 0.00 | 0.00 | 0.00 | 0.00 | 0.00 | 0.00 | 0.01 | 0.01 | 0.00 | 0.00 | 0.00 | Proteobacteria | Deltaproteobacteria | Desulfovibrionales | Desulfovibrionaceae | Transient-rare taxa |
| OTU 1734 | 0.00 | 0.00 | 0.00 | 0.00 | 0.00 | 0.00 | 0.01 | 0.00 | 0.01 | 0.00 | 0.00 | 0.00 | Proteobacteria | Alphaproteobacteria | Sphingomonadales | Erythrobacteraceae | Transient-rare taxa |
| OTU 1735 | 0.01 | 0.01 | 0.00 | 0.00 | 0.00 | 0.00 | 0.00 | 0.00 | 0.00 | 0.00 | 0.00 | 0.00 | Proteobacteria | Gammaproteobacteria | Chromatiales | Ectothiorhodospiraceae | Transient-rare taxa |
| OTU 1736 | 0.00 | 0.01 | 0.00 | 0.00 | 0.00 | 0.00 | 0.00 | 0.00 | 0.00 | 0.00 | 0.01 | 0.00 | Bacteroidetes | Cytophagia | Cytophagales | Cytophagaceae | Transient-rare taxa |
| OTU 1737 | 0.00 | 0.01 | 0.00 | 0.00 | 0.00 | 0.00 | 0.00 | 0.01 | 0.00 | 0.00 | 0.00 | 0.00 | Proteobacteria | Deltaproteobacteria | Myxococcales | Nannocystineae | Transient-rare taxa |
| OTU 1738 | 0.00 | 0.01 | 0.00 | 0.00 | 0.00 | 0.00 | 0.00 | 0.00 | 0.00 | 0.01 | 0.00 | 0.00 | Proteobacteria | Betaproteobacteria | Rhodocyclales | Rhodocyclaceae | Transient-rare taxa |
| OTU 1739 | 0.00 | 0.02 | 0.00 | 0.00 | 0.00 | 0.00 | 0.00 | 0.00 | 0.00 | 0.00 | 0.00 | 0.00 | Proteobacteria | Betaproteobacteria | Hydrogenophilales | Hydrogenophilaceae | Transient-rare taxa |
| OTU 1740 | 0.00 | 0.00 | 0.00 | 0.00 | 0.00 | 0.00 | 0.00 | 0.00 | 0.02 | 0.00 | 0.00 | 0.00 | Proteobacteria | Gammaproteobacteria | Pseudomonadales | Pseudomonadaceae | Transient-rare taxa |
| OTU 1741 | 0.00 | 0.00 | 0.00 | 0.00 | 0.00 | 0.00 | 0.01 | 0.00 | 0.01 | 0.00 | 0.00 | 0.00 | Planctomycetes | Planctomycetia | Planctomycetales | Planctomycetaceae | Transient-rare taxa |
| OTU 1742 | 0.00 | 0.00 | 0.00 | 0.00 | 0.00 | 0.00 | 0.00 | 0.01 | 0.01 | 0.00 | 0.00 | 0.00 | Proteobacteria | Deltaproteobacteria | Desulfobacterales | Desulfobacteraceae | Transient-rare taxa |
| OTU 1743 | 0.00 | 0.00 | 0.00 | 0.00 | 0.00 | 0.00 | 0.02 | 0.00 | 0.00 | 0.00 | 0.00 | 0.00 | Proteobacteria | Gammaproteobacteria | Xanthomonadales | Xanthomonadaceae | Transient-rare taxa |
| OTU 1744 | 0.00 | 0.00 | 0.01 | 0.00 | 0.00 | 0.00 | 0.00 | 0.01 | 0.00 | 0.00 | 0.00 | 0.00 | Chloroflexi | Caldilineae | Caldilineales | Caldilineaceae | Transient-rare taxa |
| OTU 1745 | 0.00 | 0.02 | 0.00 | 0.00 | 0.00 | 0.00 | 0.00 | 0.00 | 0.00 | 0.00 | 0.00 | 0.00 | Proteobacteria | Betaproteobacteria | Burkholderiales | Comamonadaceae | Transient-rare taxa |
| OTU 1746 | 0.00 | 0.01 | 0.01 | 0.00 | 0.00 | 0.00 | 0.00 | 0.00 | 0.00 | 0.00 | 0.00 | 0.00 | Proteobacteria | Gammaproteobacteria | Gammaproteobacteria_incertae_sedis | Porticoccus | Transient-rare taxa |
| OTU 1747 | 0.01 | 0.00 | 0.00 | 0.00 | 0.00 | 0.00 | 0.00 | 0.01 | 0.00 | 0.00 | 0.00 | 0.00 | Bacteroidetes | Flavobacteriia | Flavobacteriales | Flavobacteriaceae | Transient-rare taxa |
| OTU 1748 | 0.00 | 0.01 | 0.00 | 0.00 | 0.00 | 0.00 | 0.01 | 0.00 | 0.00 | 0.00 | 0.00 | 0.00 | Proteobacteria | Deltaproteobacteria | Myxococcales | Nannocystineae | Transient-rare taxa |
| OTU 1749 | 0.00 | 0.00 | 0.00 | 0.00 | 0.00 | 0.00 | 0.00 | 0.00 | 0.02 | 0.00 | 0.00 | 0.00 | Planctomycetes | Planctomycetia | Planctomycetales | Planctomycetaceae | Transient-rare taxa |
| OTU 1750 | 0.00 | 0.00 | 0.00 | 0.00 | 0.00 | 0.00 | 0.01 | 0.01 | 0.00 | 0.00 | 0.00 | 0.00 | Proteobacteria | Betaproteobacteria | Rhodocyclales | Rhodocyclaceae | Transient-rare taxa |
| OTU 1751 | 0.01 | 0.01 | 0.00 | 0.00 | 0.00 | 0.00 | 0.00 | 0.00 | 0.00 | 0.00 | 0.00 | 0.00 | Proteobacteria | Betaproteobacteria | Neisseriales | Neisseriaceae | Transient-rare taxa |
| OTU 1752 | 0.00 | 0.02 | 0.00 | 0.00 | 0.00 | 0.00 | 0.00 | 0.00 | 0.00 | 0.00 | 0.00 | 0.00 | Bacteroidetes | Flavobacteriia | Flavobacteriales | Flavobacteriaceae | Transient-rare taxa |
| OTU 1753 | 0.00 | 0.01 | 0.00 | 0.01 | 0.00 | 0.00 | 0.00 | 0.00 | 0.00 | 0.00 | 0.00 | 0.00 | Proteobacteria | Deltaproteobacteria | Desulfuromonadales | Geobacteraceae | Transient-rare taxa |
| OTU 1754 | 0.00 | 0.00 | 0.00 | 0.00 | 0.00 | 0.00 | 0.00 | 0.01 | 0.01 | 0.00 | 0.00 | 0.00 | Planctomycetes | Phycisphaerae | Phycisphaerales | Phycisphaeraceae | Transient-rare taxa |
| OTU 1755 | 0.00 | 0.01 | 0.00 | 0.00 | 0.00 | 0.00 | 0.01 | 0.00 | 0.00 | 0.00 | 0.00 | 0.00 | Proteobacteria | Deltaproteobacteria | Myxococcales | Nannocystineae | Transient-rare taxa |
| OTU 1756 | 0.01 | 0.01 | 0.00 | 0.00 | 0.00 | 0.00 | 0.00 | 0.00 | 0.00 | 0.00 | 0.00 | 0.00 | Proteobacteria | Gammaproteobacteria | Chromatiales | Chromatiaceae | Transient-rare taxa |
| OTU 1757 | 0.00 | 0.02 | 0.00 | 0.00 | 0.00 | 0.00 | 0.00 | 0.00 | 0.00 | 0.00 | 0.00 | 0.00 | Proteobacteria | Betaproteobacteria | Rhodocyclales | Rhodocyclaceae | Transient-rare taxa |
| OTU 1758 | 0.00 | 0.01 | 0.00 | 0.00 | 0.00 | 0.00 | 0.00 | 0.00 | 0.01 | 0.00 | 0.00 | 0.00 | Proteobacteria | Betaproteobacteria | Rhodocyclales | Rhodocyclaceae | Transient-rare taxa |
| OTU 1759 | 0.00 | 0.00 | 0.00 | 0.00 | 0.00 | 0.00 | 0.02 | 0.00 | 0.00 | 0.00 | 0.00 | 0.00 | Proteobacteria | Gammaproteobacteria | Alteromonadales | Alteromonadaceae | Transient-rare taxa |
| OTU 1760 | 0.00 | 0.00 | 0.00 | 0.00 | 0.00 | 0.00 | 0.00 | 0.01 | 0.01 | 0.00 | 0.00 | 0.00 | Proteobacteria | Betaproteobacteria | Rhodocyclales | Rhodocyclaceae | Transient-rare taxa |
| OTU 1761 | 0.00 | 0.02 | 0.00 | 0.00 | 0.00 | 0.00 | 0.00 | 0.00 | 0.00 | 0.00 | 0.00 | 0.00 | Acidobacteria | Acidobacteria_Gp3 | Gp3 | | Transient-rare taxa |
| OTU 1762 | 0.00 | 0.01 | 0.00 | 0.01 | 0.00 | 0.00 | 0.00 | 0.00 | 0.00 | 0.00 | 0.00 | 0.00 | Bacteroidetes | Sphingobacteriia | Sphingobacteriales | Saprospiraceae | Transient-rare taxa |
| OTU 1763 | 0.00 | 0.00 | 0.00 | 0.00 | 0.00 | 0.00 | 0.00 | 0.00 | 0.00 | 0.00 | 0.02 | 0.00 | Bacteroidetes | Sphingobacteriia | Sphingobacteriales | Chitinophagaceae | Transient-rare taxa |
| OTU 1764 | 0.00 | 0.00 | 0.00 | 0.00 | 0.00 | 0.00 | 0.00 | 0.00 | 0.02 | 0.00 | 0.00 | 0.00 | Proteobacteria | Gammaproteobacteria | Xanthomonadales | Xanthomonadaceae | Transient-rare taxa |
| OTU 1765 | 0.00 | 0.00 | 0.00 | 0.00 | 0.01 | 0.00 | 0.01 | 0.00 | 0.00 | 0.00 | 0.00 | 0.00 | Proteobacteria | Betaproteobacteria | Rhodocyclales | Rhodocyclaceae | Transient-rare taxa |
| OTU 1766 | 0.00 | 0.00 | 0.01 | 0.00 | 0.00 | 0.00 | 0.00 | 0.00 | 0.01 | 0.00 | 0.00 | 0.00 | Proteobacteria | Alphaproteobacteria | Sphingomonadales | Sphingomonadaceae | Transient-rare taxa |
| OTU 1767 | 0.00 | 0.00 | 0.00 | 0.00 | 0.00 | 0.00 | 0.01 | 0.00 | 0.00 | 0.00 | 0.01 | 0.00 | Actinobacteria | Actinobacteria | Actinobacteridae | Actinomycetales | Transient-rare taxa |
| OTU 1768 | 0.01 | 0.00 | 0.00 | 0.00 | 0.00 | 0.00 | 0.00 | 0.00 | 0.00 | 0.00 | 0.01 | 0.00 | Proteobacteria | Betaproteobacteria | Rhodocyclales | Rhodocyclaceae | Transient-rare taxa |
| OTU 1769 | 0.00 | 0.00 | 0.00 | 0.00 | 0.00 | 0.00 | 0.00 | 0.00 | 0.00 | 0.00 | 0.02 | 0.00 | Proteobacteria | Betaproteobacteria | Burkholderiales | Comamonadaceae | Transient-rare taxa |
| OTU 1770 | 0.00 | 0.00 | 0.00 | 0.01 | 0.00 | 0.00 | 0.00 | 0.00 | 0.00 | 0.00 | 0.01 | 0.00 | Proteobacteria | Gammaproteobacteria | Pseudomonadales | Moraxellaceae | Transient-rare taxa |
| OTU 1771 | 0.00 | 0.02 | 0.00 | 0.00 | 0.00 | 0.00 | 0.00 | 0.00 | 0.00 | 0.00 | 0.00 | 0.00 | Proteobacteria | Alphaproteobacteria | Sphingomonadales | Sphingomonadaceae | Transient-rare taxa |
| OTU 1772 | 0.00 | 0.01 | 0.01 | 0.00 | 0.00 | 0.00 | 0.00 | 0.00 | 0.00 | 0.00 | 0.00 | 0.00 | Nitrospirae | Nitrospira | Nitrospirales | Nitrospiraceae | Transient-rare taxa |
| OTU 1773 | 0.00 | 0.02 | 0.00 | 0.00 | 0.00 | 0.00 | 0.00 | 0.00 | 0.00 | 0.00 | 0.00 | 0.00 | Proteobacteria | Deltaproteobacteria | Myxococcales | Nannocystineae | Transient-rare taxa |
| OTU 1774 | 0.00 | 0.00 | 0.00 | 0.00 | 0.02 | 0.00 | 0.00 | 0.00 | 0.00 | 0.00 | 0.00 | 0.00 | Firmicutes | Clostridia | Clostridiales | Ruminococcaceae | Transient-rare taxa |
| OTU 1775 | 0.00 | 0.02 | 0.00 | 0.00 | 0.00 | 0.00 | 0.00 | 0.00 | 0.00 | 0.00 | 0.00 | 0.00 | Verrucomicrobia | Opitutae | Opitutales | Opitutaceae | Transient-rare taxa |
| OTU 1776 | 0.00 | 0.00 | 0.00 | 0.00 | 0.00 | 0.00 | 0.00 | 0.00 | 0.02 | 0.00 | 0.00 | 0.00 | Bacteroidetes | Sphingobacteriia | Sphingobacteriales | Saprospiraceae | Transient-rare taxa |
| OTU 1777 | 0.00 | 0.00 | 0.00 | 0.00 | 0.00 | 0.00 | 0.00 | 0.00 | 0.01 | 0.00 | 0.01 | 0.00 | Proteobacteria | Alphaproteobacteria | Rhodobacterales | Rhodobacteraceae | Transient-rare taxa |
| OTU 1778 | 0.00 | 0.01 | 0.00 | 0.00 | 0.00 | 0.00 | 0.00 | 0.01 | 0.00 | 0.00 | 0.00 | 0.00 | Proteobacteria | Betaproteobacteria | Burkholderiales | Comamonadaceae | Transient-rare taxa |
| OTU 1779 | 0.00 | 0.01 | 0.00 | 0.00 | 0.00 | 0.00 | 0.00 | 0.00 | 0.00 | 0.00 | 0.00 | 0.01 | Proteobacteria | Alphaproteobacteria | Rhodobacterales | Rhodobacteraceae | Transient-rare taxa |
| OTU 1780 | 0.01 | 0.01 | 0.00 | 0.00 | 0.00 | 0.00 | 0.00 | 0.00 | 0.00 | 0.00 | 0.00 | 0.00 | Proteobacteria | Gammaproteobacteria | Chromatiales | Chromatiaceae | Transient-rare taxa |
| OTU 1781 | 0.00 | 0.00 | 0.00 | 0.00 | 0.00 | 0.01 | 0.00 | 0.00 | 0.01 | 0.00 | 0.00 | 0.00 | Proteobacteria | Gammaproteobacteria | Xanthomonadales | Xanthomonadaceae | Transient-rare taxa |
| OTU 1782 | 0.00 | 0.00 | 0.00 | 0.00 | 0.00 | 0.00 | 0.00 | 0.00 | 0.01 | 0.00 | 0.00 | 0.01 | Firmicutes | Bacilli | Bacillales | Bacillaceae 2 | Transient-rare taxa |
| OTU 1783 | 0.00 | 0.00 | 0.00 | 0.00 | 0.00 | 0.00 | 0.00 | 0.00 | 0.01 | 0.00 | 0.00 | 0.01 | Spirochaetes | Spirochaetia | Spirochaetales | Spirochaetaceae | Transient-rare taxa |
| OTU 1784 | 0.00 | 0.02 | 0.00 | 0.00 | 0.00 | 0.00 | 0.00 | 0.00 | 0.00 | 0.00 | 0.00 | 0.00 | Proteobacteria | Deltaproteobacteria | Desulfobacterales | Desulfobacteraceae | Transient-rare taxa |
| OTU 1785 | 0.00 | 0.00 | 0.00 | 0.00 | 0.00 | 0.00 | 0.00 | 0.00 | 0.01 | 0.01 | 0.00 | 0.00 | Planctomycetes | Planctomycetia | Planctomycetales | Planctomycetaceae | Transient-rare taxa |
| OTU 1786 | 0.00 | 0.00 | 0.00 | 0.00 | 0.00 | 0.00 | 0.00 | 0.00 | 0.00 | 0.00 | 0.02 | 0.00 | Proteobacteria | Betaproteobacteria | Rhodocyclales | Rhodocyclaceae | Transient-rare taxa |
| OTU 1787 | 0.00 | 0.00 | 0.01 | 0.00 | 0.00 | 0.00 | 0.00 | 0.00 | 0.00 | 0.00 | 0.01 | 0.00 | Proteobacteria | Alphaproteobacteria | Rhizobiales | Brucellaceae | Transient-rare taxa |
| OTU 1788 | 0.00 | 0.00 | 0.01 | 0.00 | 0.00 | 0.00 | 0.01 | 0.00 | 0.00 | 0.00 | 0.00 | 0.00 | Proteobacteria | Gammaproteobacteria | Chromatiales | Ectothiorhodospiraceae | Transient-rare taxa |
| OTU 1789 | 0.00 | 0.00 | 0.00 | 0.00 | 0.00 | 0.00 | 0.00 | 0.00 | 0.00 | 0.01 | 0.01 | 0.00 | Bacteroidetes | Flavobacteriia | Flavobacteriales | Flavobacteriaceae | Transient-rare taxa |
| OTU 1790 | 0.00 | 0.00 | 0.00 | 0.00 | 0.01 | 0.00 | 0.00 | 0.00 | 0.00 | 0.00 | 0.01 | 0.00 | Proteobacteria | Betaproteobacteria | Rhodocyclales | Rhodocyclaceae | Transient-rare taxa |
| OTU 1791 | 0.00 | 0.00 | 0.00 | 0.00 | 0.00 | 0.00 | 0.00 | 0.00 | 0.00 | 0.00 | 0.02 | 0.00 | Bacteroidetes | Flavobacteriia | Flavobacteriales | Flavobacteriaceae | Transient-rare taxa |
| OTU 1792 | 0.00 | 0.00 | 0.00 | 0.00 | 0.00 | 0.00 | 0.00 | 0.01 | 0.00 | 0.00 | 0.01 | 0.00 | Proteobacteria | Betaproteobacteria | Rhodocyclales | Rhodocyclaceae | Transient-rare taxa |
| OTU 1793 | 0.00 | 0.00 | 0.00 | 0.00 | 0.00 | 0.00 | 0.00 | 0.00 | 0.00 | 0.00 | 0.02 | 0.00 | Bacteroidetes | Flavobacteriia | Flavobacteriales | Flavobacteriaceae | Transient-rare taxa |
| OTU 1794 | 0.00 | 0.00 | 0.00 | 0.00 | 0.00 | 0.00 | 0.00 | 0.00 | 0.00 | 0.00 | 0.02 | 0.00 | Proteobacteria | Deltaproteobacteria | Myxococcales | Nannocystineae | Transient-rare taxa |
| OTU 1795 | 0.00 | 0.00 | 0.00 | 0.00 | 0.00 | 0.00 | 0.00 | 0.00 | 0.00 | 0.00 | 0.02 | 0.00 | Proteobacteria | Betaproteobacteria | Rhodocyclales | Rhodocyclaceae | Transient-rare taxa |
| OTU 1796 | 0.00 | 0.00 | 0.00 | 0.00 | 0.00 | 0.00 | 0.00 | 0.00 | 0.00 | 0.00 | 0.02 | 0.00 | Bacteroidetes | Sphingobacteriia | Sphingobacteriales | Saprospiraceae | Transient-rare taxa |
| OTU 1797 | 0.00 | 0.00 | 0.00 | 0.01 | 0.00 | 0.00 | 0.01 | 0.00 | 0.00 | 0.00 | 0.00 | 0.00 | Proteobacteria | Deltaproteobacteria | Myxococcales | Cystobacterineae | Transient-rare taxa |
| OTU 1798 | 0.01 | 0.01 | 0.00 | 0.00 | 0.00 | 0.00 | 0.00 | 0.00 | 0.00 | 0.00 | 0.00 | 0.00 | Proteobacteria | Gammaproteobacteria | Xanthomonadales | Xanthomonadaceae | Transient-rare taxa |
| OTU 1799 | 0.00 | 0.00 | 0.00 | 0.00 | 0.02 | 0.00 | 0.00 | 0.00 | 0.00 | 0.00 | 0.00 | 0.00 | Bacteroidetes | Flavobacteriia | Flavobacteriales | Cryomorphaceae | Transient-rare taxa |
| OTU 1800 | 0.00 | 0.02 | 0.00 | 0.00 | 0.00 | 0.00 | 0.00 | 0.00 | 0.00 | 0.00 | 0.00 | 0.00 | Planctomycetes | Planctomycetia | Planctomycetales | Planctomycetaceae | Transient-rare taxa |
| OTU 1801 | 0.00 | 0.00 | 0.00 | 0.00 | 0.00 | 0.00 | 0.00 | 0.00 | 0.00 | 0.00 | 0.02 | 0.00 | Proteobacteria | Betaproteobacteria | Rhodocyclales | Rhodocyclaceae | Transient-rare taxa |
| OTU 1802 | 0.00 | 0.00 | 0.00 | 0.00 | 0.00 | 0.00 | 0.01 | 0.00 | 0.00 | 0.00 | 0.01 | 0.00 | Bacteroidetes | Flavobacteriia | Flavobacteriales | Flavobacteriaceae | Transient-rare taxa |
| OTU 1803 | 0.00 | 0.00 | 0.01 | 0.00 | 0.00 | 0.00 | 0.00 | 0.00 | 0.01 | 0.00 | 0.00 | 0.00 | Proteobacteria | Betaproteobacteria | Rhodocyclales | Rhodocyclaceae | Transient-rare taxa |
| OTU 1804 | 0.00 | 0.02 | 0.00 | 0.00 | 0.00 | 0.00 | 0.00 | 0.00 | 0.00 | 0.00 | 0.00 | 0.00 | Proteobacteria | Deltaproteobacteria | Desulfobacterales | Desulfobacteraceae | Transient-rare taxa |
| OTU 1805 | 0.00 | 0.00 | 0.00 | 0.00 | 0.00 | 0.00 | 0.00 | 0.00 | 0.01 | 0.00 | 0.00 | 0.01 | Bacteroidetes | Flavobacteriia | Flavobacteriales | Flavobacteriaceae | Transient-rare taxa |
| OTU 1806 | 0.00 | 0.00 | 0.00 | 0.00 | 0.00 | 0.00 | 0.00 | 0.00 | 0.01 | 0.01 | 0.00 | 0.00 | Bacteroidetes | Bacteroidia | Bacteroidales | Porphyromonadaceae | Transient-rare taxa |
| OTU 1807 | 0.00 | 0.01 | 0.01 | 0.00 | 0.00 | 0.00 | 0.00 | 0.00 | 0.00 | 0.00 | 0.00 | 0.00 | Proteobacteria | Betaproteobacteria | Hydrogenophilales | Hydrogenophilaceae | Transient-rare taxa |
| OTU 1808 | 0.00 | 0.00 | 0.00 | 0.00 | 0.00 | 0.00 | 0.00 | 0.01 | 0.01 | 0.00 | 0.00 | 0.00 | Proteobacteria | Betaproteobacteria | Nitrosomonadales | Nitrosomonadaceae | Transient-rare taxa |
| OTU 1809 | 0.00 | 0.00 | 0.00 | 0.00 | 0.02 | 0.00 | 0.00 | 0.00 | 0.00 | 0.00 | 0.00 | 0.00 | Proteobacteria | Betaproteobacteria | Burkholderiales | Burkholderiaceae | Transient-rare taxa |
| OTU 1810 | 0.00 | 0.01 | 0.00 | 0.00 | 0.00 | 0.00 | 0.00 | 0.00 | 0.01 | 0.00 | 0.00 | 0.00 | Proteobacteria | Betaproteobacteria | Rhodocyclales | Rhodocyclaceae | Transient-rare taxa |
| OTU 1811 | 0.00 | 0.00 | 0.00 | 0.00 | 0.00 | 0.01 | 0.00 | 0.00 | 0.01 | 0.00 | 0.00 | 0.00 | Bacteroidetes | Sphingobacteriia | Sphingobacteriales | Chitinophagaceae | Transient-rare taxa |
| OTU 1812 | 0.00 | 0.00 | 0.00 | 0.00 | 0.00 | 0.00 | 0.02 | 0.00 | 0.00 | 0.00 | 0.00 | 0.00 | Proteobacteria | Gammaproteobacteria | Thiotrichales | Thiotrichaceae | Transient-rare taxa |
| OTU 1813 | 0.00 | 0.00 | 0.00 | 0.00 | 0.00 | 0.00 | 0.00 | 0.00 | 0.00 | 0.00 | 0.02 | 0.00 | Proteobacteria | Deltaproteobacteria | Myxococcales | Nannocystineae | Transient-rare taxa |
| OTU 1814 | 0.00 | 0.00 | 0.00 | 0.00 | 0.00 | 0.00 | 0.02 | 0.00 | 0.00 | 0.00 | 0.00 | 0.00 | Proteobacteria | Gammaproteobacteria | Methylococcales | Methylococcaceae | Transient-rare taxa |
| OTU 1815 | 0.00 | 0.00 | 0.00 | 0.00 | 0.00 | 0.00 | 0.00 | 0.00 | 0.00 | 0.00 | 0.02 | 0.00 | Proteobacteria | Betaproteobacteria | Burkholderiales | Comamonadaceae | Transient-rare taxa |
| OTU 1816 | 0.01 | 0.00 | 0.00 | 0.00 | 0.00 | 0.00 | 0.01 | 0.00 | 0.00 | 0.00 | 0.00 | 0.00 | Proteobacteria | Betaproteobacteria | Rhodocyclales | Rhodocyclaceae | Transient-rare taxa |
| OTU 1817 | 0.00 | 0.00 | 0.00 | 0.00 | 0.00 | 0.00 | 0.00 | 0.01 | 0.00 | 0.00 | 0.01 | 0.00 | Proteobacteria | Alphaproteobacteria | Rhodobacterales | Rhodobacteraceae | Transient-rare taxa |
| OTU 1818 | 0.00 | 0.00 | 0.01 | 0.00 | 0.00 | 0.00 | 0.01 | 0.00 | 0.00 | 0.00 | 0.00 | 0.00 | Proteobacteria | Betaproteobacteria | Nitrosomonadales | Nitrosomonadaceae | Transient-rare taxa |
| OTU 1819 | 0.00 | 0.00 | 0.00 | 0.00 | 0.00 | 0.00 | 0.01 | 0.00 | 0.01 | 0.00 | 0.00 | 0.00 | Proteobacteria | Gammaproteobacteria | Legionellales | Legionellaceae | Transient-rare taxa |
| OTU 1820 | 0.00 | 0.00 | 0.00 | 0.00 | 0.00 | 0.00 | 0.00 | 0.00 | 0.00 | 0.00 | 0.02 | 0.00 | Proteobacteria | Deltaproteobacteria | Myxococcales | Nannocystineae | Transient-rare taxa |
| OTU 1821 | 0.00 | 0.00 | 0.00 | 0.01 | 0.00 | 0.00 | 0.01 | 0.00 | 0.00 | 0.00 | 0.00 | 0.00 | Proteobacteria | Gammaproteobacteria | Gammaproteobacteria_incertae_sedis | Thiohalobacter | Transient-rare taxa |
| OTU 1822 | 0.00 | 0.01 | 0.00 | 0.00 | 0.00 | 0.00 | 0.00 | 0.00 | 0.01 | 0.00 | 0.00 | 0.00 | Proteobacteria | Deltaproteobacteria | Myxococcales | Sorangiineae | Transient-rare taxa |
| OTU 1823 | 0.00 | 0.02 | 0.00 | 0.00 | 0.00 | 0.00 | 0.00 | 0.00 | 0.00 | 0.00 | 0.00 | 0.00 | Planctomycetes | Planctomycetia | Candidatus Brocadiales | Candidatus Brocadiaceae | Transient-rare taxa |
| OTU 1824 | 0.00 | 0.00 | 0.00 | 0.00 | 0.00 | 0.00 | 0.01 | 0.01 | 0.00 | 0.00 | 0.00 | 0.00 | Firmicutes | Clostridia | Clostridiales | Ruminococcaceae | Transient-rare taxa |
| OTU 1825 | 0.00 | 0.00 | 0.00 | 0.00 | 0.01 | 0.00 | 0.01 | 0.00 | 0.00 | 0.00 | 0.00 | 0.00 | Tenericutes | Mollicutes | Acholeplasmatales | Acholeplasmataceae | Transient-rare taxa |
| OTU 1826 | 0.00 | 0.00 | 0.00 | 0.00 | 0.01 | 0.00 | 0.01 | 0.00 | 0.00 | 0.00 | 0.00 | 0.00 | Proteobacteria | Gammaproteobacteria | Chromatiales | Ectothiorhodospiraceae | Transient-rare taxa |
| OTU 1827 | 0.00 | 0.00 | 0.00 | 0.00 | 0.00 | 0.00 | 0.01 | 0.00 | 0.00 | 0.00 | 0.01 | 0.00 | Proteobacteria | Betaproteobacteria | Burkholderiales | Comamonadaceae | Transient-rare taxa |
| OTU 1828 | 0.00 | 0.01 | 0.00 | 0.00 | 0.00 | 0.00 | 0.00 | 0.01 | 0.00 | 0.00 | 0.00 | 0.00 | Proteobacteria | Betaproteobacteria | Rhodocyclales | Rhodocyclaceae | Transient-rare taxa |
| OTU 1829 | 0.00 | 0.02 | 0.00 | 0.00 | 0.00 | 0.00 | 0.00 | 0.00 | 0.00 | 0.00 | 0.00 | 0.00 | Proteobacteria | Gammaproteobacteria | Chromatiales | Chromatiaceae | Transient-rare taxa |
| OTU 1830 | 0.00 | 0.02 | 0.00 | 0.00 | 0.00 | 0.00 | 0.00 | 0.00 | 0.00 | 0.00 | 0.00 | 0.00 | Proteobacteria | Betaproteobacteria | Rhodocyclales | Rhodocyclaceae | Transient-rare taxa |
| OTU 1831 | 0.01 | 0.01 | 0.00 | 0.00 | 0.00 | 0.00 | 0.00 | 0.00 | 0.00 | 0.00 | 0.00 | 0.00 | Proteobacteria | Betaproteobacteria | Burkholderiales | Comamonadaceae | Transient-rare taxa |
| OTU 1832 | 0.00 | 0.00 | 0.00 | 0.00 | 0.02 | 0.00 | 0.00 | 0.00 | 0.00 | 0.00 | 0.00 | 0.00 | Bacteroidetes | Sphingobacteriia | Sphingobacteriales | Saprospiraceae | Transient-rare taxa |
| OTU 1833 | 0.00 | 0.00 | 0.01 | 0.00 | 0.01 | 0.00 | 0.00 | 0.00 | 0.00 | 0.00 | 0.00 | 0.00 | Proteobacteria | Betaproteobacteria | Burkholderiales | Comamonadaceae | Transient-rare taxa |
| OTU 1834 | 0.00 | 0.00 | 0.00 | 0.00 | 0.00 | 0.00 | 0.00 | 0.00 | 0.02 | 0.00 | 0.00 | 0.00 | Proteobacteria | Deltaproteobacteria | Desulfuromonadales | Geobacteraceae | Transient-rare taxa |
| OTU 1835 | 0.00 | 0.00 | 0.00 | 0.00 | 0.00 | 0.00 | 0.00 | 0.02 | 0.00 | 0.00 | 0.00 | 0.00 | Planctomycetes | Phycisphaerae | Phycisphaerales | Phycisphaeraceae | Transient-rare taxa |
| OTU 1836 | 0.01 | 0.00 | 0.00 | 0.00 | 0.00 | 0.00 | 0.00 | 0.00 | 0.01 | 0.00 | 0.00 | 0.00 | Chloroflexi | Anaerolineae | Anaerolineales | Anaerolineaceae | Transient-rare taxa |
| OTU 1837 | 0.00 | 0.00 | 0.00 | 0.00 | 0.00 | 0.00 | 0.00 | 0.00 | 0.02 | 0.00 | 0.00 | 0.00 | Proteobacteria | Betaproteobacteria | Rhodocyclales | Rhodocyclaceae | Transient-rare taxa |
| OTU 1838 | 0.00 | 0.00 | 0.00 | 0.00 | 0.00 | 0.00 | 0.02 | 0.00 | 0.00 | 0.00 | 0.00 | 0.00 | Proteobacteria | Deltaproteobacteria | Myxococcales | Nannocystineae | Transient-rare taxa |

| | | | | | | | | | | | | | | | | | |
|---|---|---|---|---|---|---|---|---|---|---|---|---|---|---|---|---|---|
| OTU 1839 | 0.00 | 0.00 | 0.00 | 0.00 | 0.00 | 0.00 | 0.00 | 0.00 | 0.01 | 0.00 | 0.00 | 0.01 | Proteobacteria | Alphaproteobacteria | Sphingomonadales | Sphingomonadaceae | Transient-rare taxa |
| OTU 1840 | 0.00 | 0.00 | 0.00 | 0.00 | 0.00 | 0.00 | 0.00 | 0.00 | 0.01 | 0.01 | 0.00 | 0.00 | Proteobacteria | Alphaproteobacteria | Caulobacterales | Hyphomonadaceae | Transient-rare taxa |
| OTU 1841 | 0.01 | 0.00 | 0.00 | 0.00 | 0.00 | 0.00 | 0.00 | 0.00 | 0.01 | 0.00 | 0.00 | 0.00 | Planctomycetes | Planctomycetia | Planctomycetales | Planctomycetaceae | Transient-rare taxa |
| OTU 1842 | 0.00 | 0.00 | 0.00 | 0.00 | 0.00 | 0.00 | 0.00 | 0.00 | 0.02 | 0.00 | 0.00 | 0.00 | Proteobacteria | Betaproteobacteria | Rhodocyclales | Rhodocyclaceae | Transient-rare taxa |
| OTU 1843 | 0.00 | 0.01 | 0.00 | 0.00 | 0.00 | 0.00 | 0.00 | 0.00 | 0.01 | 0.00 | 0.00 | 0.00 | Bacteroidetes | Sphingobacteriia | Sphingobacteriales | Saprospiraceae | Transient-rare taxa |
| OTU 1844 | 0.00 | 0.00 | 0.00 | 0.00 | 0.00 | 0.00 | 0.00 | 0.00 | 0.02 | 0.00 | 0.00 | 0.00 | Proteobacteria | Betaproteobacteria | Burkholderiales | Burkholderiales_incertae_sedis | Transient-rare taxa |
| OTU 1845 | 0.01 | 0.00 | 0.00 | 0.00 | 0.00 | 0.00 | 0.00 | 0.00 | 0.01 | 0.00 | 0.00 | 0.00 | Proteobacteria | Betaproteobacteria | Rhodocyclales | Rhodocyclaceae | Transient-rare taxa |
| OTU 1846 | 0.00 | 0.00 | 0.00 | 0.00 | 0.00 | 0.00 | 0.00 | 0.00 | 0.01 | 0.01 | 0.00 | 0.00 | Verrucomicrobia | Verrucomicrobiae | Verrucomicrobiales | Verrucomicrobiaceae | Transient-rare taxa |
| OTU 1847 | 0.00 | 0.00 | 0.01 | 0.00 | 0.00 | 0.00 | 0.00 | 0.00 | 0.01 | 0.00 | 0.00 | 0.00 | Proteobacteria | Betaproteobacteria | Burkholderiales | Comamonadaceae | Transient-rare taxa |
| OTU 1848 | 0.00 | 0.00 | 0.00 | 0.02 | 0.00 | 0.00 | 0.00 | 0.00 | 0.00 | 0.00 | 0.00 | 0.00 | Proteobacteria | Epsilonproteobacteria | Campylobacterales | Helicobacteraceae | Transient-rare taxa |
| OTU 1849 | 0.00 | 0.00 | 0.00 | 0.02 | 0.00 | 0.00 | 0.00 | 0.00 | 0.00 | 0.00 | 0.00 | 0.00 | Bacteroidetes | Bacteroidia | Bacteroidales | Porphyromonadaceae | Transient-rare taxa |
| OTU 1850 | 0.01 | 0.01 | 0.00 | 0.00 | 0.00 | 0.00 | 0.00 | 0.00 | 0.00 | 0.00 | 0.00 | 0.00 | Proteobacteria | Betaproteobacteria | Rhodocyclales | Rhodocyclaceae | Transient-rare taxa |
| OTU 1851 | 0.00 | 0.01 | 0.00 | 0.01 | 0.00 | 0.00 | 0.00 | 0.00 | 0.00 | 0.00 | 0.00 | 0.00 | Firmicutes | Clostridia | Clostridiales | Gracilibacteraceae | Transient-rare taxa |
| OTU 1852 | 0.00 | 0.02 | 0.00 | 0.00 | 0.00 | 0.00 | 0.00 | 0.00 | 0.00 | 0.00 | 0.00 | 0.00 | Proteobacteria | Betaproteobacteria | Burkholderiales | Comamonadaceae | Transient-rare taxa |
| OTU 1853 | 0.00 | 0.00 | 0.00 | 0.00 | 0.02 | 0.00 | 0.00 | 0.00 | 0.00 | 0.00 | 0.00 | 0.00 | Proteobacteria | Deltaproteobacteria | Desulfobacterales | Desulfobacteraceae | Transient-rare taxa |
| OTU 1854 | 0.00 | 0.00 | 0.00 | 0.00 | 0.01 | 0.00 | 0.00 | 0.01 | 0.00 | 0.00 | 0.00 | 0.00 | Proteobacteria | Betaproteobacteria | Burkholderiales | Comamonadaceae | Transient-rare taxa |
| OTU 1855 | 0.01 | 0.01 | 0.00 | 0.00 | 0.00 | 0.00 | 0.00 | 0.00 | 0.00 | 0.00 | 0.00 | 0.00 | Proteobacteria | Gammaproteobacteria | Chromatiales | Chromatiaceae | Transient-rare taxa |
| OTU 1856 | 0.00 | 0.01 | 0.00 | 0.00 | 0.00 | 0.00 | 0.00 | 0.01 | 0.00 | 0.00 | 0.00 | 0.00 | Bacteroidetes | Flavobacteriia | Flavobacteriales | Flavobacteriaceae | Transient-rare taxa |
| OTU 1857 | 0.00 | 0.01 | 0.01 | 0.00 | 0.00 | 0.00 | 0.00 | 0.00 | 0.00 | 0.00 | 0.00 | 0.00 | Proteobacteria | Gammaproteobacteria | Gammaproteobacteria_incertae_sedis | Thiohalobacter | Transient-rare taxa |
| OTU 1858 | 0.00 | 0.00 | 0.00 | 0.00 | 0.00 | 0.00 | 0.00 | 0.02 | 0.00 | 0.00 | 0.00 | 0.00 | Bacteroidetes | Flavobacteriia | Flavobacteriales | Flavobacteriaceae | Transient-rare taxa |
| OTU 1859 | 0.00 | 0.02 | 0.00 | 0.00 | 0.00 | 0.00 | 0.00 | 0.00 | 0.00 | 0.00 | 0.00 | 0.00 | Proteobacteria | Alphaproteobacteria | Rhizobiales | Bradyrhizobiaceae | Transient-rare taxa |
| OTU 1860 | 0.00 | 0.01 | 0.00 | 0.00 | 0.00 | 0.00 | 0.00 | 0.01 | 0.00 | 0.00 | 0.00 | 0.00 | Proteobacteria | Betaproteobacteria | Rhodocyclales | Rhodocyclaceae | Transient-rare taxa |
| OTU 1861 | 0.00 | 0.00 | 0.00 | 0.00 | 0.02 | 0.00 | 0.00 | 0.00 | 0.00 | 0.00 | 0.00 | 0.00 | Bacteroidetes | Bacteroidia | Bacteroidales | Marinilabiliaceae | Transient-rare taxa |
| OTU 1862 | 0.00 | 0.02 | 0.00 | 0.00 | 0.00 | 0.00 | 0.00 | 0.00 | 0.00 | 0.00 | 0.00 | 0.00 | Proteobacteria | Gammaproteobacteria | Chromatiales | Chromatiaceae | Transient-rare taxa |
| OTU 1863 | 0.00 | 0.01 | 0.00 | 0.00 | 0.00 | 0.00 | 0.00 | 0.00 | 0.00 | 0.00 | 0.00 | 0.01 | Proteobacteria | Gammaproteobacteria | Xanthomonadales | Xanthomonadaceae | Transient-rare taxa |
| OTU 1864 | 0.00 | 0.02 | 0.00 | 0.00 | 0.00 | 0.00 | 0.00 | 0.00 | 0.00 | 0.00 | 0.00 | 0.00 | Proteobacteria | Alphaproteobacteria | Sphingomonadales | Sphingomonadaceae | Transient-rare taxa |
| OTU 1865 | 0.01 | 0.01 | 0.00 | 0.00 | 0.00 | 0.00 | 0.00 | 0.00 | 0.00 | 0.00 | 0.00 | 0.00 | Proteobacteria | Gammaproteobacteria | Xanthomonadales | Xanthomonadaceae | Transient-rare taxa |
| OTU 1866 | 0.00 | 0.01 | 0.00 | 0.00 | 0.00 | 0.00 | 0.00 | 0.00 | 0.00 | 0.00 | 0.00 | 0.01 | Proteobacteria | Betaproteobacteria | Rhodocyclales | Rhodocyclaceae | Transient-rare taxa |
| OTU 1867 | 0.00 | 0.00 | 0.00 | 0.00 | 0.00 | 0.00 | 0.00 | 0.02 | 0.00 | 0.00 | 0.00 | 0.00 | Bacteroidetes | Sphingobacteriia | Sphingobacteriales | Saprospiraceae | Transient-rare taxa |
| OTU 1868 | 0.00 | 0.01 | 0.00 | 0.00 | 0.00 | 0.00 | 0.00 | 0.01 | 0.00 | 0.00 | 0.00 | 0.00 | Proteobacteria | Deltaproteobacteria | Myxococcales | Sorangiineae | Transient-rare taxa |
| OTU 1869 | 0.00 | 0.02 | 0.00 | 0.00 | 0.00 | 0.00 | 0.00 | 0.00 | 0.00 | 0.00 | 0.00 | 0.00 | Proteobacteria | Deltaproteobacteria | Myxococcales | Sorangiineae | Transient-rare taxa |
| OTU 1870 | 0.00 | 0.00 | 0.00 | 0.00 | 0.00 | 0.00 | 0.00 | 0.00 | 0.02 | 0.00 | 0.00 | 0.00 | Proteobacteria | Betaproteobacteria | Rhodocyclales | Rhodocyclaceae | Transient-rare taxa |
| OTU 1871 | 0.00 | 0.00 | 0.00 | 0.00 | 0.00 | 0.00 | 0.00 | 0.01 | 0.01 | 0.00 | 0.00 | 0.00 | Planctomycetes | Planctomycetia | Planctomycetales | Planctomycetaceae | Transient-rare taxa |
| OTU 1872 | 0.00 | 0.00 | 0.00 | 0.00 | 0.00 | 0.00 | 0.00 | 0.00 | 0.02 | 0.00 | 0.00 | 0.00 | Proteobacteria | Betaproteobacteria | Rhodocyclales | Rhodocyclaceae | Transient-rare taxa |
| OTU 1873 | 0.00 | 0.00 | 0.00 | 0.00 | 0.00 | 0.00 | 0.00 | 0.00 | 0.02 | 0.00 | 0.00 | 0.00 | Bacteroidetes | Sphingobacteriia | Sphingobacteriales | Saprospiraceae | Transient-rare taxa |
| OTU 1874 | 0.00 | 0.00 | 0.00 | 0.00 | 0.00 | 0.00 | 0.00 | 0.00 | 0.01 | 0.01 | 0.00 | 0.00 | Proteobacteria | Alphaproteobacteria | Rhizobiales | Methylobacteriaceae | Transient-rare taxa |
| OTU 1875 | 0.00 | 0.00 | 0.00 | 0.00 | 0.02 | 0.00 | 0.00 | 0.00 | 0.00 | 0.00 | 0.00 | 0.00 | Proteobacteria | Deltaproteobacteria | Bdellovibrionales | Bdellovibrionaceae | Transient-rare taxa |
| OTU 1876 | 0.01 | 0.01 | 0.00 | 0.00 | 0.00 | 0.00 | 0.00 | 0.00 | 0.00 | 0.00 | 0.00 | 0.00 | Proteobacteria | Betaproteobacteria | Burkholderiales | Comamonadaceae | Transient-rare taxa |
| OTU 1877 | 0.01 | 0.01 | 0.00 | 0.00 | 0.00 | 0.00 | 0.00 | 0.00 | 0.00 | 0.00 | 0.00 | 0.00 | Bacteroidetes | Sphingobacteriia | Sphingobacteriales | Saprospiraceae | Transient-rare taxa |
| OTU 1878 | 0.00 | 0.00 | 0.00 | 0.02 | 0.00 | 0.00 | 0.00 | 0.00 | 0.00 | 0.00 | 0.00 | 0.00 | Bacteroidetes | Flavobacteriia | Flavobacteriales | Cryomorphaceae | Transient-rare taxa |
| OTU 1879 | 0.00 | 0.02 | 0.00 | 0.00 | 0.00 | 0.00 | 0.00 | 0.00 | 0.00 | 0.00 | 0.00 | 0.00 | Gemmatimonadetes | Gemmatimonadetes | Gemmatimonadales | Gemmatimonadaceae | Transient-rare taxa |
| OTU 1880 | 0.00 | 0.00 | 0.00 | 0.00 | 0.00 | 0.00 | 0.00 | 0.00 | 0.02 | 0.00 | 0.00 | 0.00 | Proteobacteria | Alphaproteobacteria | Rhizobiales | Beijerinckiaceae | Transient-rare taxa |
| OTU 1881 | 0.00 | 0.00 | 0.00 | 0.00 | 0.00 | 0.00 | 0.00 | 0.01 | 0.01 | 0.00 | 0.00 | 0.00 | Proteobacteria | Betaproteobacteria | Rhodocyclales | Rhodocyclaceae | Transient-rare taxa |
| OTU 1882 | 0.00 | 0.00 | 0.00 | 0.00 | 0.00 | 0.00 | 0.00 | 0.00 | 0.02 | 0.00 | 0.00 | 0.00 | Chloroflexi | Anaerolineae | Anaerolineales | Anaerolineaceae | Transient-rare taxa |
| OTU 1883 | 0.00 | 0.00 | 0.01 | 0.00 | 0.00 | 0.00 | 0.00 | 0.00 | 0.01 | 0.00 | 0.00 | 0.00 | Planctomycetes | Phycisphaerae | Phycisphaerales | Phycisphaeraceae | Transient-rare taxa |
| OTU 1884 | 0.00 | 0.00 | 0.00 | 0.02 | 0.00 | 0.00 | 0.00 | 0.00 | 0.00 | 0.00 | 0.00 | 0.00 | Proteobacteria | Gammaproteobacteria | Halioglobus | | Transient-rare taxa |
| OTU 1885 | 0.01 | 0.01 | 0.00 | 0.00 | 0.00 | 0.00 | 0.00 | 0.00 | 0.00 | 0.00 | 0.00 | 0.00 | Proteobacteria | Gammaproteobacteria | Xanthomonadales | Xanthomonadaceae | Transient-rare taxa |
| OTU 1886 | 0.00 | 0.00 | 0.00 | 0.00 | 0.00 | 0.00 | 0.00 | 0.00 | 0.02 | 0.00 | 0.00 | 0.00 | Proteobacteria | Gammaproteobacteria | Xanthomonadales | Xanthomonadaceae | Transient-rare taxa |
| OTU 1887 | 0.00 | 0.00 | 0.00 | 0.00 | 0.00 | 0.00 | 0.00 | 0.00 | 0.01 | 0.00 | 0.01 | 0.00 | Proteobacteria | Alphaproteobacteria | Rhodobacterales | Rhodobacteraceae | Transient-rare taxa |
| OTU 1888 | 0.00 | 0.01 | 0.00 | 0.00 | 0.00 | 0.00 | 0.00 | 0.00 | 0.00 | 0.01 | 0.00 | 0.00 | Proteobacteria | Betaproteobacteria | Rhodocyclales | Rhodocyclaceae | Transient-rare taxa |
| OTU 1889 | 0.00 | 0.00 | 0.00 | 0.01 | 0.00 | 0.00 | 0.00 | 0.00 | 0.00 | 0.00 | 0.01 | 0.00 | Proteobacteria | Alphaproteobacteria | Caulobacterales | Caulobacteraceae | Transient-rare taxa |
| OTU 1890 | 0.00 | 0.01 | 0.00 | 0.01 | 0.00 | 0.00 | 0.00 | 0.00 | 0.00 | 0.00 | 0.00 | 0.00 | Actinobacteria | Actinobacteria | Acidimicrobidae | Acidimicrobiales | Transient-rare taxa |
| OTU 1891 | 0.00 | 0.02 | 0.00 | 0.00 | 0.00 | 0.00 | 0.00 | 0.00 | 0.00 | 0.00 | 0.00 | 0.00 | Proteobacteria | Betaproteobacteria | Burkholderiales | Comamonadaceae | Transient-rare taxa |
| OTU 1892 | 0.00 | 0.01 | 0.00 | 0.01 | 0.00 | 0.00 | 0.00 | 0.00 | 0.00 | 0.00 | 0.00 | 0.00 | Actinobacteria | Actinobacteria | Actinobacteridae | Actinomycetales | Transient-rare taxa |
| OTU 1893 | 0.00 | 0.01 | 0.00 | 0.00 | 0.00 | 0.00 | 0.00 | 0.01 | 0.00 | 0.00 | 0.00 | 0.00 | Proteobacteria | Alphaproteobacteria | Rhodospirillales | Acetobacteraceae | Transient-rare taxa |
| OTU 1894 | 0.00 | 0.01 | 0.00 | 0.00 | 0.00 | 0.00 | 0.00 | 0.00 | 0.01 | 0.00 | 0.00 | 0.00 | Proteobacteria | Betaproteobacteria | Rhodocyclales | Rhodocyclaceae | Transient-rare taxa |
| OTU 1895 | 0.00 | 0.01 | 0.01 | 0.00 | 0.00 | 0.00 | 0.00 | 0.00 | 0.00 | 0.00 | 0.00 | 0.00 | Proteobacteria | Betaproteobacteria | Burkholderiales | Comamonadaceae | Transient-rare taxa |
| OTU 1896 | 0.00 | 0.01 | 0.00 | 0.00 | 0.00 | 0.00 | 0.00 | 0.01 | 0.00 | 0.00 | 0.00 | 0.00 | Chloroflexi | Anaerolineae | Anaerolineales | Anaerolineaceae | Transient-rare taxa |
| OTU 1897 | 0.00 | 0.01 | 0.00 | 0.00 | 0.00 | 0.00 | 0.00 | 0.00 | 0.00 | 0.00 | 0.00 | 0.01 | Actinobacteria | Actinobacteria | Actinobacteridae | Actinomycetales | Transient-rare taxa |
| OTU 1898 | 0.00 | 0.00 | 0.00 | 0.00 | 0.00 | 0.00 | 0.02 | 0.00 | 0.00 | 0.00 | 0.00 | 0.00 | Proteobacteria | Gammaproteobacteria | Thiotrichales | Thiotrichaceae | Transient-rare taxa |
| OTU 1899 | 0.01 | 0.01 | 0.00 | 0.00 | 0.00 | 0.00 | 0.00 | 0.00 | 0.00 | 0.00 | 0.00 | 0.00 | Planctomycetes | Planctomycetia | Planctomycetales | Planctomycetaceae | Transient-rare taxa |
| OTU 1900 | 0.00 | 0.02 | 0.00 | 0.00 | 0.00 | 0.00 | 0.00 | 0.00 | 0.00 | 0.00 | 0.00 | 0.00 | Proteobacteria | Deltaproteobacteria | Myxococcales | Nannocystineae | Transient-rare taxa |
| OTU 1901 | 0.01 | 0.01 | 0.00 | 0.00 | 0.00 | 0.00 | 0.00 | 0.00 | 0.00 | 0.00 | 0.00 | 0.00 | Proteobacteria | Gammaproteobacteria | Chromatiales | Chromatiaceae | Transient-rare taxa |
| OTU 1902 | 0.00 | 0.01 | 0.00 | 0.00 | 0.00 | 0.00 | 0.00 | 0.00 | 0.00 | 0.00 | 0.00 | 0.01 | Bacteroidetes | Sphingobacteriia | Sphingobacteriales | Chitinophagaceae | Transient-rare taxa |
| OTU 1903 | 0.00 | 0.01 | 0.00 | 0.00 | 0.00 | 0.00 | 0.00 | 0.00 | 0.01 | 0.00 | 0.00 | 0.00 | Proteobacteria | Deltaproteobacteria | Myxococcales | Sorangiineae | Transient-rare taxa |
| OTU 1904 | 0.00 | 0.00 | 0.00 | 0.00 | 0.01 | 0.00 | 0.01 | 0.00 | 0.00 | 0.00 | 0.00 | 0.00 | Bacteroidetes | Flavobacteriia | Flavobacteriales | Flavobacteriaceae | Transient-rare taxa |
| OTU 1905 | 0.00 | 0.00 | 0.00 | 0.00 | 0.00 | 0.01 | 0.01 | 0.00 | 0.00 | 0.00 | 0.00 | 0.00 | Proteobacteria | Deltaproteobacteria | Myxococcales | Nannocystineae | Transient-rare taxa |
| OTU 1906 | 0.00 | 0.00 | 0.00 | 0.00 | 0.00 | 0.00 | 0.02 | 0.00 | 0.00 | 0.00 | 0.00 | 0.00 | Proteobacteria | Alphaproteobacteria | Sphingomonadales | Sphingomonadaceae | Transient-rare taxa |
| OTU 1907 | 0.00 | 0.00 | 0.00 | 0.00 | 0.02 | 0.00 | 0.00 | 0.00 | 0.00 | 0.00 | 0.00 | 0.00 | Proteobacteria | Gammaproteobacteria | Methylococcales | Methylococcaceae | Transient-rare taxa |
| OTU 1908 | 0.00 | 0.00 | 0.00 | 0.00 | 0.01 | 0.01 | 0.00 | 0.00 | 0.00 | 0.00 | 0.00 | 0.00 | Bacteroidetes | Flavobacteriia | Flavobacteriales | Flavobacteriaceae | Transient-rare taxa |
| OTU 1909 | 0.00 | 0.00 | 0.00 | 0.00 | 0.02 | 0.00 | 0.00 | 0.00 | 0.00 | 0.00 | 0.00 | 0.00 | Proteobacteria | Gammaproteobacteria | Chromatiales | Chromatiaceae | Transient-rare taxa |
| OTU 1910 | 0.00 | 0.00 | 0.00 | 0.00 | 0.02 | 0.00 | 0.00 | 0.00 | 0.00 | 0.00 | 0.00 | 0.00 | Bacteroidetes | Cytophagia | Cytophagales | Flammeovirgaceae | Transient-rare taxa |
| OTU 1911 | 0.00 | 0.00 | 0.00 | 0.00 | 0.00 | 0.00 | 0.00 | 0.00 | 0.01 | 0.01 | 0.00 | 0.00 | Proteobacteria | Alphaproteobacteria | Rhizobiales | Bradyrhizobiaceae | Transient-rare taxa |
| OTU 1912 | 0.00 | 0.01 | 0.00 | 0.00 | 0.00 | 0.00 | 0.00 | 0.01 | 0.00 | 0.00 | 0.00 | 0.00 | Planctomycetes | Planctomycetia | Planctomycetales | Planctomycetaceae | Transient-rare taxa |
| OTU 1913 | 0.00 | 0.00 | 0.00 | 0.00 | 0.00 | 0.00 | 0.00 | 0.00 | 0.02 | 0.00 | 0.00 | 0.00 | Proteobacteria | Alphaproteobacteria | Caulobacterales | Caulobacteraceae | Transient-rare taxa |
| OTU 1914 | 0.00 | 0.01 | 0.00 | 0.00 | 0.00 | 0.00 | 0.00 | 0.00 | 0.01 | 0.00 | 0.00 | 0.00 | Planctomycetes | Planctomycetia | Candidatus Brocadiales | Candidatus Brocadiaceae | Transient-rare taxa |
| OTU 1915 | 0.00 | 0.02 | 0.00 | 0.00 | 0.00 | 0.00 | 0.00 | 0.00 | 0.00 | 0.00 | 0.00 | 0.00 | Parcubacteria | Parcubacteria_genera_incertae_sedis | | | Transient-rare taxa |
| OTU 1916 | 0.00 | 0.02 | 0.00 | 0.00 | 0.00 | 0.00 | 0.00 | 0.00 | 0.00 | 0.00 | 0.00 | 0.00 | Proteobacteria | Deltaproteobacteria | Myxococcales | Sorangiineae | Transient-rare taxa |
| OTU 1917 | 0.00 | 0.01 | 0.00 | 0.01 | 0.00 | 0.00 | 0.00 | 0.00 | 0.00 | 0.00 | 0.00 | 0.00 | Actinobacteria | Actinobacteria | Actinobacteridae | Actinomycetales | Transient-rare taxa |
| OTU 1918 | 0.00 | 0.01 | 0.00 | 0.00 | 0.00 | 0.00 | 0.00 | 0.00 | 0.01 | 0.00 | 0.00 | 0.00 | Proteobacteria | Deltaproteobacteria | Myxococcales | Nannocystineae | Transient-rare taxa |
| OTU 1919 | 0.01 | 0.01 | 0.00 | 0.00 | 0.00 | 0.00 | 0.00 | 0.00 | 0.00 | 0.00 | 0.00 | 0.00 | Proteobacteria | Betaproteobacteria | Rhodocyclales | Rhodocyclaceae | Transient-rare taxa |
| OTU 1920 | 0.00 | 0.00 | 0.00 | 0.02 | 0.00 | 0.00 | 0.00 | 0.00 | 0.00 | 0.00 | 0.00 | 0.00 | Fusobacteria | Fusobacteriia | Fusobacteriales | Fusobacteriaceae | Transient-rare taxa |
| OTU 1921 | 0.00 | 0.00 | 0.00 | 0.00 | 0.00 | 0.00 | 0.00 | 0.00 | 0.02 | 0.00 | 0.00 | 0.00 | Bacteroidetes | Sphingobacteriia | Sphingobacteriales | Saprospiraceae | Transient-rare taxa |
| OTU 1922 | 0.00 | 0.01 | 0.00 | 0.00 | 0.00 | 0.00 | 0.00 | 0.00 | 0.00 | 0.00 | 0.00 | 0.01 | Proteobacteria | Alphaproteobacteria | Rhodobacterales | Rhodobacteraceae | Transient-rare taxa |
| OTU 1923 | 0.00 | 0.02 | 0.00 | 0.00 | 0.00 | 0.00 | 0.00 | 0.00 | 0.00 | 0.00 | 0.00 | 0.00 | Proteobacteria | Gammaproteobacteria | Chromatiales | Chromatiaceae | Transient-rare taxa |
| OTU 1924 | 0.00 | 0.01 | 0.00 | 0.00 | 0.00 | 0.00 | 0.00 | 0.00 | 0.00 | 0.00 | 0.00 | 0.01 | Proteobacteria | Deltaproteobacteria | Syntrophobacterales | Syntrophaceae | Transient-rare taxa |
| OTU 1925 | 0.00 | 0.00 | 0.00 | 0.01 | 0.00 | 0.01 | 0.00 | 0.00 | 0.00 | 0.00 | 0.00 | 0.00 | Proteobacteria | Deltaproteobacteria | Myxococcales | Sorangiineae | Transient-rare taxa |
| OTU 1926 | 0.00 | 0.02 | 0.00 | 0.00 | 0.00 | 0.00 | 0.00 | 0.00 | 0.00 | 0.00 | 0.00 | 0.00 | Planctomycetes | Planctomycetia | Planctomycetales | Planctomycetaceae | Transient-rare taxa |
| OTU 1927 | 0.00 | 0.01 | 0.00 | 0.00 | 0.00 | 0.00 | 0.01 | 0.00 | 0.00 | 0.00 | 0.00 | 0.00 | Proteobacteria | Alphaproteobacteria | Rhodobacterales | Rhodobacteraceae | Transient-rare taxa |
| OTU 1928 | 0.00 | 0.00 | 0.00 | 0.00 | 0.00 | 0.02 | 0.00 | 0.00 | 0.00 | 0.00 | 0.00 | 0.00 | Proteobacteria | Alphaproteobacteria | Sphingomonadales | Erythrobacteraceae | Transient-rare taxa |
| OTU 1929 | 0.00 | 0.00 | 0.00 | 0.00 | 0.00 | 0.02 | 0.00 | 0.00 | 0.00 | 0.00 | 0.00 | 0.00 | Proteobacteria | Gammaproteobacteria | Oceanospirillales | Halomonadaceae | Transient-rare taxa |
| OTU 1930 | 0.00 | 0.00 | 0.00 | 0.00 | 0.00 | 0.00 | 0.02 | 0.00 | 0.00 | 0.00 | 0.00 | 0.00 | Proteobacteria | Betaproteobacteria | Rhodocyclales | Rhodocyclaceae | Transient-rare taxa |
| OTU 1931 | 0.00 | 0.00 | 0.00 | 0.00 | 0.00 | 0.00 | 0.02 | 0.00 | 0.00 | 0.00 | 0.00 | 0.00 | Planctomycetes | Planctomycetia | Planctomycetales | Planctomycetaceae | Transient-rare taxa |
| OTU 1932 | 0.00 | 0.00 | 0.00 | 0.00 | 0.01 | 0.01 | 0.00 | 0.00 | 0.00 | 0.00 | 0.00 | 0.00 | Bacteroidetes | Flavobacteriia | Flavobacteriales | Flavobacteriaceae | Transient-rare taxa |
| OTU 1933 | 0.00 | 0.00 | 0.00 | 0.00 | 0.00 | 0.02 | 0.00 | 0.00 | 0.00 | 0.00 | 0.00 | 0.00 | Proteobacteria | Deltaproteobacteria | Desulfobacterales | Desulfobacteraceae | Transient-rare taxa |
| OTU 1934 | 0.00 | 0.00 | 0.00 | 0.00 | 0.00 | 0.02 | 0.00 | 0.00 | 0.00 | 0.00 | 0.00 | 0.00 | Actinobacteria | Actinobacteria | Actinobacteridae | Actinomycetales | Transient-rare taxa |
| OTU 1935 | 0.00 | 0.01 | 0.00 | 0.00 | 0.00 | 0.00 | 0.00 | 0.00 | 0.00 | 0.00 | 0.01 | 0.00 | Proteobacteria | Deltaproteobacteria | Bdellovibrionales | Bacteriovoracaceae | Transient-rare taxa |
| OTU 1936 | 0.00 | 0.00 | 0.00 | 0.00 | 0.01 | 0.00 | 0.00 | 0.01 | 0.00 | 0.00 | 0.00 | 0.00 | Proteobacteria | Alphaproteobacteria | Rhizobiales | Hyphomicrobiaceae | Transient-rare taxa |
| OTU 1937 | 0.00 | 0.00 | 0.00 | 0.00 | 0.00 | 0.00 | 0.02 | 0.00 | 0.00 | 0.00 | 0.00 | 0.00 | Nitrospirae | Nitrospira | Nitrospirales | Nitrospiraceae | Transient-rare taxa |
| OTU 1938 | 0.00 | 0.00 | 0.00 | 0.00 | 0.02 | 0.00 | 0.00 | 0.00 | 0.00 | 0.00 | 0.00 | 0.00 | Bacteroidetes | Sphingobacteriia | Sphingobacteriales | Saprospiraceae | Transient-rare taxa |
| OTU 1939 | 0.00 | 0.00 | 0.00 | 0.00 | 0.02 | 0.00 | 0.00 | 0.00 | 0.00 | 0.00 | 0.00 | 0.00 | Spirochaetes | Spirochaetia | Spirochaetales | Spirochaetaceae | Transient-rare taxa |
| OTU 1940 | 0.00 | 0.00 | 0.00 | 0.00 | 0.00 | 0.00 | 0.00 | 0.01 | 0.01 | 0.00 | 0.00 | 0.00 | Bacteroidetes | Flavobacteriia | Flavobacteriales | Cryomorphaceae | Transient-rare taxa |
| OTU 1941 | 0.00 | 0.00 | 0.00 | 0.00 | 0.02 | 0.00 | 0.00 | 0.00 | 0.00 | 0.00 | 0.00 | 0.00 | Firmicutes | Clostridia | Clostridiales | Natranaerovirga | Transient-rare taxa |
| OTU 1942 | 0.00 | 0.00 | 0.01 | 0.00 | 0.00 | 0.01 | 0.00 | 0.00 | 0.00 | 0.00 | 0.00 | 0.00 | Firmicutes | Clostridia | Clostridiales | Ruminococcaceae | Transient-rare taxa |
| OTU 1943 | 0.00 | 0.00 | 0.00 | 0.00 | 0.02 | 0.00 | 0.00 | 0.00 | 0.00 | 0.00 | 0.00 | 0.00 | Proteobacteria | Alphaproteobacteria | Rhodobacterales | Rhodobacteraceae | Transient-rare taxa |
| OTU 1944 | 0.00 | 0.00 | 0.00 | 0.00 | 0.00 | 0.00 | 0.00 | 0.01 | 0.01 | 0.00 | 0.00 | 0.00 | Proteobacteria | Betaproteobacteria | Burkholderiales | Comamonadaceae | Transient-rare taxa |
| OTU 1945 | 0.00 | 0.01 | 0.00 | 0.00 | 0.00 | 0.00 | 0.00 | 0.00 | 0.01 | 0.00 | 0.00 | 0.00 | Proteobacteria | Betaproteobacteria | Rhodocyclales | Rhodocyclaceae | Transient-rare taxa |
| OTU 1946 | 0.01 | 0.01 | 0.00 | 0.00 | 0.00 | 0.00 | 0.00 | 0.00 | 0.00 | 0.00 | 0.00 | 0.00 | Proteobacteria | Gammaproteobacteria | Chromatiales | Chromatiaceae | Transient-rare taxa |
| OTU 1947 | 0.00 | 0.00 | 0.00 | 0.02 | 0.00 | 0.00 | 0.00 | 0.00 | 0.00 | 0.00 | 0.00 | 0.00 | Proteobacteria | Epsilonproteobacteria | Campylobacterales | Campylobacteraceae | Transient-rare taxa |
| OTU 1948 | 0.01 | 0.01 | 0.00 | 0.00 | 0.00 | 0.00 | 0.00 | 0.00 | 0.00 | 0.00 | 0.00 | 0.00 | Proteobacteria | Gammaproteobacteria | Chromatiales | Chromatiaceae | Transient-rare taxa |
| OTU 1949 | 0.00 | 0.01 | 0.00 | 0.00 | 0.00 | 0.00 | 0.00 | 0.00 | 0.01 | 0.00 | 0.00 | 0.00 | Proteobacteria | Betaproteobacteria | Burkholderiales | Comamonadaceae | Transient-rare taxa |
| OTU 1950 | 0.00 | 0.00 | 0.00 | 0.02 | 0.00 | 0.00 | 0.00 | 0.00 | 0.00 | 0.00 | 0.00 | 0.00 | Proteobacteria | Gammaproteobacteria | Chromatiales | Ectothiorhodospiraceae | Transient-rare taxa |
| OTU 1951 | 0.00 | 0.00 | 0.00 | 0.00 | 0.01 | 0.00 | 0.00 | 0.00 | 0.01 | 0.00 | 0.00 | 0.00 | Bacteroidetes | Sphingobacteriia | Sphingobacteriales | Saprospiraceae | Transient-rare taxa |
| OTU 1952 | 0.00 | 0.00 | 0.00 | 0.00 | 0.00 | 0.00 | 0.00 | 0.00 | 0.02 | 0.00 | 0.00 | 0.00 | Bacteroidetes | Sphingobacteriia | Sphingobacteriales | Cyclobacteriaceae | Transient-rare taxa |
| OTU 1953 | 0.00 | 0.02 | 0.00 | 0.00 | 0.00 | 0.00 | 0.00 | 0.00 | 0.00 | 0.00 | 0.00 | 0.00 | Proteobacteria | Betaproteobacteria | Rhodocyclales | Rhodocyclaceae | Transient-rare taxa |
| OTU 1954 | 0.00 | 0.02 | 0.00 | 0.00 | 0.00 | 0.00 | 0.00 | 0.00 | 0.00 | 0.00 | 0.00 | 0.00 | Proteobacteria | Deltaproteobacteria | Myxococcales | Nannocystineae | Transient-rare taxa |
| OTU 1955 | 0.00 | 0.00 | 0.01 | 0.00 | 0.00 | 0.00 | 0.00 | 0.00 | 0.01 | 0.00 | 0.00 | 0.00 | Actinobacteria | Actinobacteria | Rubrobacteridae | Solirubrobacterales | Transient-rare taxa |
| OTU 1956 | 0.00 | 0.00 | 0.00 | 0.00 | 0.00 | 0.00 | 0.00 | 0.00 | 0.02 | 0.00 | 0.00 | 0.00 | Acidobacteria | Acidobacteria_Gp4 | Gp4 | | Transient-rare taxa |
| OTU 1957 | 0.00 | 0.00 | 0.00 | 0.00 | 0.00 | 0.00 | 0.00 | 0.00 | 0.00 | 0.00 | 0.02 | 0.00 | Proteobacteria | Betaproteobacteria | Burkholderiales | Comamonadaceae | Transient-rare taxa |
| OTU 1958 | 0.00 | 0.00 | 0.00 | 0.00 | 0.00 | 0.02 | 0.00 | 0.00 | 0.00 | 0.00 | 0.00 | 0.00 | Proteobacteria | Alphaproteobacteria | Rhodobacterales | Rhodobacteraceae | Transient-rare taxa |
| OTU 1959 | 0.00 | 0.00 | 0.00 | 0.00 | 0.02 | 0.00 | 0.00 | 0.00 | 0.00 | 0.00 | 0.00 | 0.00 | Bacteroidetes | Sphingobacteriia | Sphingobacteriales | Cyclobacteriaceae | Transient-rare taxa |
| OTU 1960 | 0.00 | 0.00 | 0.00 | 0.00 | 0.01 | 0.00 | 0.01 | 0.00 | 0.00 | 0.00 | 0.00 | 0.00 | Proteobacteria | Gammaproteobacteria | Thiotrichales | Thiotrichaceae | Transient-rare taxa |
| OTU 1961 | 0.00 | 0.00 | 0.00 | 0.00 | 0.00 | 0.00 | 0.00 | 0.00 | 0.00 | 0.00 | 0.01 | 0.01 | Proteobacteria | Deltaproteobacteria | Myxococcales | Cystobacterineae | Transient-rare taxa |
| OTU 1962 | 0.00 | 0.00 | 0.01 | 0.00 | 0.00 | 0.00 | 0.00 | 0.01 | 0.00 | 0.00 | 0.00 | 0.00 | Proteobacteria | Betaproteobacteria | Burkholderiales | Oxalobacteraceae | Transient-rare taxa |
| OTU 1963 | 0.00 | 0.00 | 0.00 | 0.00 | 0.00 | 0.01 | 0.01 | 0.00 | 0.00 | 0.00 | 0.00 | 0.00 | Proteobacteria | Gammaproteobacteria | Chromatiales | Ectothiorhodospiraceae | Transient-rare taxa |
| OTU 1964 | 0.00 | 0.00 | 0.01 | 0.00 | 0.00 | 0.00 | 0.00 | 0.00 | 0.00 | 0.00 | 0.01 | 0.00 | Proteobacteria | Betaproteobacteria | Rhodocyclales | Rhodocyclaceae | Transient-rare taxa |
| OTU 1965 | 0.00 | 0.00 | 0.00 | 0.00 | 0.00 | 0.00 | 0.01 | 0.00 | 0.01 | 0.00 | 0.00 | 0.00 | Proteobacteria | Betaproteobacteria | Rhodocyclales | Rhodocyclaceae | Transient-rare taxa |
| OTU 1966 | 0.00 | 0.00 | 0.00 | 0.00 | 0.00 | 0.00 | 0.00 | 0.00 | 0.00 | 0.00 | 0.02 | 0.00 | Proteobacteria | Betaproteobacteria | Rhodocyclales | Rhodocyclaceae | Transient-rare taxa |
| OTU 1967 | 0.01 | 0.00 | 0.00 | 0.00 | 0.00 | 0.00 | 0.00 | 0.00 | 0.00 | 0.00 | 0.01 | 0.00 | Proteobacteria | Gammaproteobacteria | Xanthomonadales | Xanthomonadaceae | Transient-rare taxa |
| OTU 1968 | 0.00 | 0.00 | 0.00 | 0.00 | 0.00 | 0.00 | 0.00 | 0.01 | 0.00 | 0.00 | 0.01 | 0.00 | Chloroflexi | Anaerolineae | Anaerolineales | Anaerolineaceae | Transient-rare taxa |
| OTU 1969 | 0.00 | 0.00 | 0.00 | 0.00 | 0.00 | 0.00 | 0.00 | 0.02 | 0.00 | 0.00 | 0.00 | 0.00 | Proteobacteria | Alphaproteobacteria | Rhizobiales | Xanthobacteraceae | Transient-rare taxa |
| OTU 1970 | 0.00 | 0.00 | 0.00 | 0.00 | 0.00 | 0.00 | 0.00 | 0.02 | 0.00 | 0.00 | 0.00 | 0.00 | Proteobacteria | Deltaproteobacteria | Myxococcales | Nannocystineae | Transient-rare taxa |
| OTU 1971 | 0.02 | 0.00 | 0.00 | 0.00 | 0.00 | 0.00 | 0.00 | 0.00 | 0.00 | 0.00 | 0.00 | 0.00 | Proteobacteria | Alphaproteobacteria | Rhizobiales | Hyphomicrobiaceae | Transient-rare taxa |
| OTU 1972 | 0.00 | 0.00 | 0.00 | 0.00 | 0.02 | 0.00 | 0.00 | 0.00 | 0.00 | 0.00 | 0.00 | 0.00 | Actinobacteria | Actinobacteria | Actinobacteridae | Actinomycetales | Transient-rare taxa |
| OTU 1973 | 0.02 | 0.00 | 0.00 | 0.00 | 0.00 | 0.00 | 0.00 | 0.00 | 0.00 | 0.00 | 0.00 | 0.00 | Bacteroidetes | Sphingobacteriia | Sphingobacteriales | Saprospiraceae | Transient-rare taxa |
| OTU 1974 | 0.00 | 0.00 | 0.00 | 0.00 | 0.00 | 0.00 | 0.00 | 0.01 | 0.01 | 0.00 | 0.00 | 0.00 | Bacteroidetes | Sphingobacteriia | Sphingobacteriales | Saprospiraceae | Transient-rare taxa |
| OTU 1975 | 0.02 | 0.00 | 0.00 | 0.00 | 0.00 | 0.00 | 0.00 | 0.00 | 0.00 | 0.00 | 0.00 | 0.00 | Proteobacteria | Betaproteobacteria | Rhodocyclales | Rhodocyclaceae | Transient-rare taxa |
| OTU 1976 | 0.00 | 0.00 | 0.00 | 0.00 | 0.02 | 0.00 | 0.00 | 0.00 | 0.00 | 0.00 | 0.00 | 0.00 | Proteobacteria | Gammaproteobacteria | Xanthomonadales | Sinobacteraceae | Transient-rare taxa |
| OTU 1977 | 0.00 | 0.00 | 0.01 | 0.00 | 0.00 | 0.00 | 0.00 | 0.00 | 0.01 | 0.00 | 0.00 | 0.00 | Proteobacteria | Alphaproteobacteria | Rhodobacterales | Rhodobacteraceae | Transient-rare taxa |
| OTU 1978 | 0.00 | 0.00 | 0.00 | 0.00 | 0.00 | 0.00 | 0.00 | 0.01 | 0.01 | 0.00 | 0.00 | 0.00 | Proteobacteria | Epsilonproteobacteria | Campylobacterales | Helicobacteraceae | Transient-rare taxa |
| OTU 1979 | 0.00 | 0.00 | 0.00 | 0.02 | 0.00 | 0.00 | 0.00 | 0.00 | 0.00 | 0.00 | 0.00 | 0.00 | Proteobacteria | Deltaproteobacteria | Myxococcales | Nannocystineae | Transient-rare taxa |
| OTU 1980 | 0.00 | 0.00 | 0.00 | 0.00 | 0.00 | 0.00 | 0.00 | 0.01 | 0.01 | 0.00 | 0.00 | 0.00 | Bacteroidetes | Sphingobacteriia | Sphingobacteriales | Chitinophagaceae | Transient-rare taxa |

| | | | | | | | | | | | | | | | | | |
|---|---|---|---|---|---|---|---|---|---|---|---|---|---|---|---|---|---|
| OTU 1981 | 0.00 | 0.00 | 0.00 | 0.01 | 0.00 | 0.00 | 0.00 | 0.00 | 0.01 | 0.00 | 0.00 | 0.00 | Acidobacteria | Acidobacteria_Gp6 | Gp6 | | Transient-rare taxa |
| OTU 1982 | 0.00 | 0.00 | 0.00 | 0.00 | 0.00 | 0.00 | 0.00 | 0.00 | 0.02 | 0.00 | 0.00 | 0.00 | Proteobacteria | Gammaproteobacteria | Xanthomonadales | Xanthomonadaceae | Transient-rare taxa |
| OTU 1983 | 0.01 | 0.00 | 0.00 | 0.00 | 0.00 | 0.00 | 0.00 | 0.00 | 0.01 | 0.00 | 0.00 | 0.00 | Hydrogenedentes | Candidatus Hydrogenedens | | | Transient-rare taxa |
| OTU 1984 | 0.01 | 0.00 | 0.00 | 0.00 | 0.00 | 0.00 | 0.00 | 0.00 | 0.00 | 0.00 | 0.00 | 0.01 | Bacteroidetes | Flavobacteriia | Flavobacteriales | Flavobacteriaceae | Transient-rare taxa |
| OTU 1985 | 0.01 | 0.00 | 0.00 | 0.00 | 0.00 | 0.00 | 0.00 | 0.00 | 0.00 | 0.00 | 0.00 | 0.01 | Proteobacteria | Deltaproteobacteria | Myxococcales | Nannocystineae | Transient-rare taxa |
| OTU 1986 | 0.00 | 0.00 | 0.00 | 0.00 | 0.00 | 0.00 | 0.00 | 0.02 | 0.00 | 0.00 | 0.00 | 0.00 | Proteobacteria | Betaproteobacteria | Rhodocyclales | Rhodocyclaceae | Transient-rare taxa |
| OTU 1987 | 0.00 | 0.00 | 0.00 | 0.00 | 0.00 | 0.00 | 0.00 | 0.02 | 0.00 | 0.00 | 0.00 | 0.00 | Chloroflexi | Anaerolineae | Anaerolineales | Anaerolineaceae | Transient-rare taxa |
| OTU 1988 | 0.02 | 0.00 | 0.00 | 0.00 | 0.00 | 0.00 | 0.00 | 0.00 | 0.00 | 0.00 | 0.00 | 0.00 | Planctomycetes | Planctomycetia | Planctomycetales | Planctomycetaceae | Transient-rare taxa |
| OTU 1989 | 0.02 | 0.00 | 0.00 | 0.00 | 0.00 | 0.00 | 0.00 | 0.00 | 0.00 | 0.00 | 0.00 | 0.00 | Proteobacteria | Gammaproteobacteria | Oceanospirillales | Halomonadaceae | Transient-rare taxa |
| OTU 1990 | 0.02 | 0.00 | 0.00 | 0.00 | 0.00 | 0.00 | 0.00 | 0.00 | 0.00 | 0.00 | 0.00 | 0.00 | Proteobacteria | Betaproteobacteria | Rhodocyclales | Rhodocyclaceae | Transient-rare taxa |
| OTU 1991 | 0.00 | 0.00 | 0.00 | 0.00 | 0.02 | 0.00 | 0.00 | 0.00 | 0.00 | 0.00 | 0.00 | 0.00 | Actinobacteria | Actinobacteria | Actinobacteridae | Actinomycetales | Transient-rare taxa |
| OTU 1992 | 0.00 | 0.00 | 0.00 | 0.01 | 0.01 | 0.00 | 0.00 | 0.00 | 0.00 | 0.00 | 0.00 | 0.00 | Proteobacteria | Alphaproteobacteria | Sphingomonadales | Sphingomonadaceae | Transient-rare taxa |
| OTU 1993 | 0.00 | 0.00 | 0.00 | 0.00 | 0.02 | 0.00 | 0.00 | 0.00 | 0.00 | 0.00 | 0.00 | 0.00 | Proteobacteria | Alphaproteobacteria | Rhizobiales | Rhodobiaceae | Transient-rare taxa |
| OTU 1994 | 0.00 | 0.00 | 0.00 | 0.00 | 0.00 | 0.00 | 0.00 | 0.02 | 0.00 | 0.00 | 0.00 | 0.00 | Chloroflexi | Dehalococcoidetes | Dehalogenimonas | | Transient-rare taxa |
| OTU 1995 | 0.00 | 0.00 | 0.00 | 0.00 | 0.00 | 0.00 | 0.00 | 0.01 | 0.00 | 0.00 | 0.00 | 0.01 | Proteobacteria | Betaproteobacteria | Burkholderiales | Burkholderiales_incertae_sedis | Transient-rare taxa |
| OTU 1996 | 0.00 | 0.00 | 0.00 | 0.00 | 0.02 | 0.00 | 0.00 | 0.00 | 0.00 | 0.00 | 0.00 | 0.00 | Bacteroidetes | Sphingobacteriia | Sphingobacteriales | Saprospiraceae | Transient-rare taxa |
| OTU 1997 | 0.00 | 0.00 | 0.00 | 0.02 | 0.00 | 0.00 | 0.00 | 0.00 | 0.00 | 0.00 | 0.00 | 0.00 | Bacteroidetes | Sphingobacteriia | Sphingobacteriales | Chitinophagaceae | Transient-rare taxa |
| OTU 1998 | 0.01 | 0.00 | 0.00 | 0.00 | 0.00 | 0.00 | 0.00 | 0.00 | 0.00 | 0.00 | 0.01 | 0.00 | Firmicutes | Clostridia | Clostridiales | Ruminococcaceae | Transient-rare taxa |
| OTU 1999 | 0.02 | 0.00 | 0.00 | 0.00 | 0.00 | 0.00 | 0.00 | 0.00 | 0.00 | 0.00 | 0.00 | 0.00 | Proteobacteria | Alphaproteobacteria | Sphingomonadales | Sphingomonadaceae | Transient-rare taxa |
| OTU 2000 | 0.02 | 0.00 | 0.00 | 0.00 | 0.00 | 0.00 | 0.00 | 0.00 | 0.00 | 0.00 | 0.00 | 0.00 | Bacteroidetes | Cytophagia | Cytophagales | Cytophagaceae | Transient-rare taxa |
| OTU 2001 | 0.02 | 0.00 | 0.00 | 0.00 | 0.00 | 0.00 | 0.00 | 0.00 | 0.00 | 0.00 | 0.00 | 0.00 | Bacteroidetes | Sphingobacteriia | Sphingobacteriales | Saprospiraceae | Transient-rare taxa |
| OTU 2002 | 0.00 | 0.00 | 0.02 | 0.00 | 0.00 | 0.00 | 0.00 | 0.00 | 0.00 | 0.00 | 0.00 | 0.00 | Proteobacteria | Gammaproteobacteria | Halioglobus | | Transient-rare taxa |
| OTU 2003 | 0.02 | 0.00 | 0.00 | 0.00 | 0.00 | 0.00 | 0.00 | 0.00 | 0.00 | 0.00 | 0.00 | 0.00 | Bacteroidetes | Sphingobacteriia | Sphingobacteriales | Cyclobacteriaceae | Transient-rare taxa |
| OTU 2004 | 0.00 | 0.00 | 0.02 | 0.00 | 0.00 | 0.00 | 0.00 | 0.00 | 0.00 | 0.00 | 0.00 | 0.00 | Proteobacteria | Gammaproteobacteria | Alteromonadales | Pseudoalteromonadaceae | Transient-rare taxa |
| OTU 2005 | 0.00 | 0.00 | 0.02 | 0.00 | 0.00 | 0.00 | 0.00 | 0.00 | 0.00 | 0.00 | 0.00 | 0.00 | Bacteroidetes | Flavobacteriia | Flavobacteriales | Flavobacteriaceae | Transient-rare taxa |
| OTU 2006 | 0.00 | 0.00 | 0.02 | 0.00 | 0.00 | 0.00 | 0.00 | 0.00 | 0.00 | 0.00 | 0.00 | 0.00 | Actinobacteria | Actinobacteria | Acidimicrobidae | Acidimicrobiales | Transient-rare taxa |
| OTU 2007 | 0.01 | 0.00 | 0.00 | 0.00 | 0.00 | 0.00 | 0.00 | 0.00 | 0.00 | 0.00 | 0.00 | 0.01 | Bacteroidetes | Sphingobacteriia | Sphingobacteriales | Saprospiraceae | Transient-rare taxa |
| OTU 2008 | 0.02 | 0.00 | 0.00 | 0.00 | 0.00 | 0.00 | 0.00 | 0.00 | 0.00 | 0.00 | 0.00 | 0.00 | Proteobacteria | Betaproteobacteria | Rhodocyclales | Rhodocyclaceae | Transient-rare taxa |
| OTU 2009 | 0.02 | 0.00 | 0.00 | 0.00 | 0.00 | 0.00 | 0.00 | 0.00 | 0.00 | 0.00 | 0.00 | 0.00 | Bacteroidetes | Sphingobacteriia | Sphingobacteriales | Cyclobacteriaceae | Transient-rare taxa |
| OTU 2010 | 0.01 | 0.01 | 0.00 | 0.00 | 0.00 | 0.00 | 0.00 | 0.00 | 0.00 | 0.00 | 0.00 | 0.00 | Proteobacteria | Gammaproteobacteria | Chromatiales | Chromatiaceae | Transient-rare taxa |
| OTU 2011 | 0.01 | 0.00 | 0.00 | 0.00 | 0.00 | 0.00 | 0.00 | 0.01 | 0.00 | 0.00 | 0.00 | 0.00 | Proteobacteria | Epsilonproteobacteria | Campylobacterales | Campylobacteraceae | Transient-rare taxa |
| OTU 2012 | 0.00 | 0.00 | 0.00 | 0.02 | 0.00 | 0.00 | 0.00 | 0.00 | 0.00 | 0.00 | 0.00 | 0.00 | Proteobacteria | Alphaproteobacteria | Rhodobacterales | Rhodobacteraceae | Transient-rare taxa |
| OTU 2013 | 0.00 | 0.00 | 0.00 | 0.00 | 0.00 | 0.00 | 0.01 | 0.01 | 0.00 | 0.00 | 0.00 | 0.00 | Proteobacteria | Gammaproteobacteria | Pseudomonadales | Pseudomonadaceae | Transient-rare taxa |
| OTU 2014 | 0.00 | 0.00 | 0.00 | 0.02 | 0.00 | 0.00 | 0.00 | 0.00 | 0.00 | 0.00 | 0.00 | 0.00 | Proteobacteria | Gammaproteobacteria | Gammaproteobacteria_incertae_sedis | Methylohalomonas | Transient-rare taxa |
| OTU 2015 | 0.00 | 0.00 | 0.00 | 0.01 | 0.00 | 0.00 | 0.01 | 0.00 | 0.00 | 0.00 | 0.00 | 0.00 | Bacteroidetes | Cytophagia | Cytophagales | Cytophagaceae | Transient-rare taxa |
| OTU 2016 | 0.00 | 0.00 | 0.02 | 0.00 | 0.00 | 0.00 | 0.00 | 0.00 | 0.00 | 0.00 | 0.00 | 0.00 | Proteobacteria | Gammaproteobacteria | Alteromonadales | Maricurvus | Transient-rare taxa |
| OTU 2017 | 0.00 | 0.00 | 0.02 | 0.00 | 0.00 | 0.00 | 0.00 | 0.00 | 0.00 | 0.00 | 0.00 | 0.00 | Proteobacteria | Gammaproteobacteria | Chromatiales | Ectothiorhodospiraceae | Transient-rare taxa |
| OTU 2018 | 0.01 | 0.01 | 0.00 | 0.00 | 0.00 | 0.00 | 0.00 | 0.00 | 0.00 | 0.00 | 0.00 | 0.00 | Proteobacteria | Deltaproteobacteria | Myxococcales | Cystobacterineae | Transient-rare taxa |
| OTU 2019 | 0.00 | 0.02 | 0.00 | 0.00 | 0.00 | 0.00 | 0.00 | 0.00 | 0.00 | 0.00 | 0.00 | 0.00 | Proteobacteria | Betaproteobacteria | Rhodocyclales | Rhodocyclaceae | Transient-rare taxa |
| OTU 2020 | 0.00 | 0.00 | 0.00 | 0.02 | 0.00 | 0.00 | 0.00 | 0.00 | 0.00 | 0.00 | 0.00 | 0.00 | Bacteroidetes | Flavobacteriia | Flavobacteriales | Cryomorphaceae | Transient-rare taxa |
| OTU 2021 | 0.02 | 0.00 | 0.00 | 0.00 | 0.00 | 0.00 | 0.00 | 0.00 | 0.00 | 0.00 | 0.00 | 0.00 | Proteobacteria | Betaproteobacteria | Gallionellales | Gallionellaceae | Transient-rare taxa |
| OTU 2022 | 0.00 | 0.02 | 0.00 | 0.00 | 0.00 | 0.00 | 0.00 | 0.00 | 0.00 | 0.00 | 0.00 | 0.00 | Proteobacteria | Betaproteobacteria | Rhodocyclales | Rhodocyclaceae | Transient-rare taxa |
| OTU 2023 | 0.00 | 0.00 | 0.00 | 0.00 | 0.00 | 0.00 | 0.00 | 0.01 | 0.00 | 0.00 | 0.00 | 0.01 | Firmicutes | Clostridia | Clostridiales | Ruminococcaceae | Transient-rare taxa |
| OTU 2024 | 0.00 | 0.02 | 0.00 | 0.00 | 0.00 | 0.00 | 0.00 | 0.00 | 0.00 | 0.00 | 0.00 | 0.00 | Bacteroidetes | Cytophagia | Cytophagales | Flammeovirgaceae | Transient-rare taxa |
| OTU 2025 | 0.02 | 0.00 | 0.00 | 0.00 | 0.00 | 0.00 | 0.00 | 0.00 | 0.00 | 0.00 | 0.00 | 0.00 | Proteobacteria | Gammaproteobacteria | Xanthomonadales | Xanthomonadaceae | Transient-rare taxa |
| OTU 2026 | 0.02 | 0.00 | 0.00 | 0.00 | 0.00 | 0.00 | 0.00 | 0.00 | 0.00 | 0.00 | 0.00 | 0.00 | Proteobacteria | Deltaproteobacteria | Myxococcales | Sorangiineae | Transient-rare taxa |
| OTU 2027 | 0.00 | 0.01 | 0.00 | 0.00 | 0.00 | 0.00 | 0.00 | 0.01 | 0.00 | 0.00 | 0.00 | 0.00 | Proteobacteria | Deltaproteobacteria | Myxococcales | Nannocystineae | Transient-rare taxa |
| OTU 2028 | 0.01 | 0.00 | 0.00 | 0.00 | 0.00 | 0.00 | 0.00 | 0.00 | 0.00 | 0.01 | 0.00 | 0.00 | Actinobacteria | Actinobacteria | Actinobacteridae | Actinomycetales | Transient-rare taxa |
| OTU 2029 | 0.01 | 0.01 | 0.00 | 0.00 | 0.00 | 0.00 | 0.00 | 0.00 | 0.00 | 0.00 | 0.00 | 0.00 | Proteobacteria | Betaproteobacteria | Rhodocyclales | Rhodocyclaceae | Transient-rare taxa |
| OTU 2030 | 0.00 | 0.01 | 0.01 | 0.00 | 0.00 | 0.00 | 0.00 | 0.00 | 0.00 | 0.00 | 0.00 | 0.00 | Proteobacteria | Betaproteobacteria | Rhodocyclales | Rhodocyclaceae | Transient-rare taxa |
| OTU 2031 | 0.00 | 0.00 | 0.00 | 0.00 | 0.00 | 0.00 | 0.01 | 0.01 | 0.00 | 0.00 | 0.00 | 0.00 | Planctomycetes | Planctomycetia | Planctomycetales | Planctomycetaceae | Transient-rare taxa |
| OTU 2032 | 0.00 | 0.02 | 0.00 | 0.00 | 0.00 | 0.00 | 0.00 | 0.00 | 0.00 | 0.00 | 0.00 | 0.00 | Proteobacteria | Betaproteobacteria | Rhodocyclales | Rhodocyclaceae | Transient-rare taxa |
| OTU 2033 | 0.00 | 0.00 | 0.00 | 0.01 | 0.01 | 0.00 | 0.00 | 0.00 | 0.00 | 0.00 | 0.00 | 0.00 | Bacteroidetes | Flavobacteriia | Flavobacteriales | Cryomorphaceae | Transient-rare taxa |
| OTU 2034 | 0.00 | 0.00 | 0.02 | 0.00 | 0.00 | 0.00 | 0.00 | 0.00 | 0.00 | 0.00 | 0.00 | 0.00 | Bacteroidetes | Bacteroidia | Bacteroidales | Sunxiuqinia | Transient-rare taxa |
| OTU 2035 | 0.00 | 0.00 | 0.02 | 0.00 | 0.00 | 0.00 | 0.00 | 0.00 | 0.00 | 0.00 | 0.00 | 0.00 | Chloroflexi | Caldilineae | Caldilineales | Caldilineaceae | Transient-rare taxa |
| OTU 2036 | 0.00 | 0.01 | 0.00 | 0.01 | 0.00 | 0.00 | 0.00 | 0.00 | 0.00 | 0.00 | 0.00 | 0.00 | Planctomycetes | Planctomycetia | Planctomycetales | Planctomycetaceae | Transient-rare taxa |
| OTU 2037 | 0.00 | 0.00 | 0.02 | 0.00 | 0.00 | 0.00 | 0.00 | 0.00 | 0.00 | 0.00 | 0.00 | 0.00 | Proteobacteria | Gammaproteobacteria | Pseudomonadales | Moraxellaceae | Transient-rare taxa |
| OTU 2038 | 0.00 | 0.00 | 0.00 | 0.01 | 0.00 | 0.00 | 0.00 | 0.00 | 0.00 | 0.00 | 0.00 | 0.01 | Bacteroidetes | Flavobacteriia | Flavobacteriales | Cryomorphaceae | Transient-rare taxa |
| OTU 2039 | 0.01 | 0.00 | 0.00 | 0.00 | 0.00 | 0.00 | 0.00 | 0.00 | 0.00 | 0.00 | 0.00 | 0.01 | Bacteroidetes | Flavobacteriia | Flavobacteriales | Flavobacteriaceae | Transient-rare taxa |
| OTU 2040 | 0.00 | 0.01 | 0.00 | 0.00 | 0.00 | 0.00 | 0.00 | 0.00 | 0.00 | 0.00 | 0.00 | 0.01 | Bacteroidetes | Sphingobacteriia | Sphingobacteriales | Saprospiraceae | Transient-rare taxa |
| OTU 2041 | 0.00 | 0.00 | 0.00 | 0.00 | 0.00 | 0.00 | 0.00 | 0.00 | 0.00 | 0.00 | 0.00 | 0.02 | Proteobacteria | Alphaproteobacteria | Caulobacterales | Caulobacteraceae | Transient-rare taxa |
| OTU 2042 | 0.01 | 0.00 | 0.00 | 0.00 | 0.00 | 0.00 | 0.00 | 0.01 | 0.00 | 0.00 | 0.00 | 0.00 | Firmicutes | Negativicutes | Selenomonadales | Veillonellaceae | Transient-rare taxa |
| OTU 2043 | 0.02 | 0.00 | 0.00 | 0.00 | 0.00 | 0.00 | 0.00 | 0.00 | 0.00 | 0.00 | 0.00 | 0.00 | Planctomycetes | Planctomycetia | Planctomycetales | Planctomycetaceae | Transient-rare taxa |
| OTU 2044 | 0.02 | 0.00 | 0.00 | 0.00 | 0.00 | 0.00 | 0.00 | 0.00 | 0.00 | 0.00 | 0.00 | 0.00 | Bacteroidetes | Sphingobacteriia | Sphingobacteriales | Saprospiraceae | Transient-rare taxa |
| OTU 2045 | 0.00 | 0.00 | 0.00 | 0.00 | 0.00 | 0.00 | 0.00 | 0.01 | 0.01 | 0.00 | 0.00 | 0.00 | Proteobacteria | Alphaproteobacteria | Rhizobiales | Brucellaceae | Transient-rare taxa |
| OTU 2046 | 0.02 | 0.00 | 0.00 | 0.00 | 0.00 | 0.00 | 0.00 | 0.00 | 0.00 | 0.00 | 0.00 | 0.00 | Proteobacteria | Betaproteobacteria | Rhodocyclales | Rhodocyclaceae | Transient-rare taxa |
| OTU 2047 | 0.00 | 0.00 | 0.00 | 0.00 | 0.00 | 0.00 | 0.01 | 0.00 | 0.01 | 0.00 | 0.00 | 0.00 | Proteobacteria | Alphaproteobacteria | Sphingomonadales | Erythrobacteraceae | Transient-rare taxa |
| OTU 2048 | 0.00 | 0.00 | 0.00 | 0.00 | 0.00 | 0.02 | 0.00 | 0.00 | 0.00 | 0.00 | 0.00 | 0.00 | Bacteroidetes | Flavobacteriia | Flavobacteriales | Cryomorphaceae | Transient-rare taxa |
| OTU 2049 | 0.00 | 0.01 | 0.00 | 0.00 | 0.00 | 0.00 | 0.00 | 0.00 | 0.00 | 0.01 | 0.00 | 0.00 | Proteobacteria | Deltaproteobacteria | Desulfobacterales | Desulfobacteraceae | Transient-rare taxa |
| OTU 2050 | 0.01 | 0.01 | 0.00 | 0.00 | 0.00 | 0.00 | 0.00 | 0.00 | 0.00 | 0.00 | 0.00 | 0.00 | Proteobacteria | Gammaproteobacteria | Chromatiales | Chromatiaceae | Transient-rare taxa |
| OTU 2051 | 0.00 | 0.00 | 0.00 | 0.00 | 0.00 | 0.00 | 0.00 | 0.00 | 0.02 | 0.00 | 0.00 | 0.00 | Proteobacteria | Deltaproteobacteria | Myxococcales | Sorangiineae | Transient-rare taxa |
| OTU 2052 | 0.00 | 0.00 | 0.00 | 0.00 | 0.00 | 0.00 | 0.00 | 0.01 | 0.01 | 0.00 | 0.00 | 0.00 | Chloroflexi | Anaerolineae | Anaerolineales | Anaerolineaceae | Transient-rare taxa |
| OTU 2053 | 0.01 | 0.00 | 0.00 | 0.00 | 0.00 | 0.00 | 0.00 | 0.00 | 0.01 | 0.00 | 0.00 | 0.00 | Proteobacteria | Alphaproteobacteria | Rhodobacterales | Rhodobacteraceae | Transient-rare taxa |
| OTU 2054 | 0.00 | 0.00 | 0.00 | 0.00 | 0.00 | 0.00 | 0.00 | 0.00 | 0.00 | 0.00 | 0.00 | 0.02 | Proteobacteria | Gammaproteobacteria | Pseudomonadales | Moraxellaceae | Transient-rare taxa |
| OTU 2055 | 0.01 | 0.01 | 0.00 | 0.00 | 0.00 | 0.00 | 0.00 | 0.00 | 0.00 | 0.00 | 0.00 | 0.00 | Proteobacteria | Deltaproteobacteria | Myxococcales | Cystobacterineae | Transient-rare taxa |
| OTU 2056 | 0.02 | 0.00 | 0.00 | 0.00 | 0.00 | 0.00 | 0.00 | 0.00 | 0.00 | 0.00 | 0.00 | 0.00 | Proteobacteria | Alphaproteobacteria | Rhizobiales | Methylocystaceae | Transient-rare taxa |
| OTU 2057 | 0.01 | 0.00 | 0.00 | 0.00 | 0.00 | 0.00 | 0.00 | 0.00 | 0.01 | 0.00 | 0.00 | 0.00 | Proteobacteria | Betaproteobacteria | Burkholderiales | Burkholderiales_incertae_sedis | Transient-rare taxa |
| OTU 2058 | 0.00 | 0.00 | 0.00 | 0.00 | 0.00 | 0.00 | 0.00 | 0.00 | 0.01 | 0.01 | 0.00 | 0.00 | Proteobacteria | Betaproteobacteria | Rhodocyclales | Rhodocyclaceae | Transient-rare taxa |
| OTU 2059 | 0.00 | 0.00 | 0.00 | 0.00 | 0.00 | 0.00 | 0.00 | 0.00 | 0.01 | 0.01 | 0.00 | 0.00 | Proteobacteria | Deltaproteobacteria | Desulfobacterales | Desulfobacteraceae | Transient-rare taxa |
| OTU 2060 | 0.00 | 0.00 | 0.00 | 0.00 | 0.00 | 0.00 | 0.00 | 0.00 | 0.01 | 0.01 | 0.00 | 0.00 | Acidobacteria | Acidobacteria_Gp1 | Candidatus Koribacter | | Transient-rare taxa |
| OTU 2061 | 0.01 | 0.00 | 0.00 | 0.00 | 0.00 | 0.00 | 0.00 | 0.00 | 0.00 | 0.00 | 0.00 | 0.01 | Proteobacteria | Betaproteobacteria | Rhodocyclales | Rhodocyclaceae | Transient-rare taxa |
| OTU 2062 | 0.01 | 0.00 | 0.01 | 0.00 | 0.00 | 0.00 | 0.00 | 0.00 | 0.00 | 0.00 | 0.00 | 0.00 | Proteobacteria | Deltaproteobacteria | Myxococcales | Sorangiineae | Transient-rare taxa |
| OTU 2063 | 0.01 | 0.00 | 0.00 | 0.00 | 0.00 | 0.00 | 0.00 | 0.00 | 0.00 | 0.01 | 0.00 | 0.00 | Proteobacteria | Epsilonproteobacteria | Campylobacterales | Campylobacteraceae | Transient-rare taxa |
| OTU 2064 | 0.02 | 0.00 | 0.00 | 0.00 | 0.00 | 0.00 | 0.00 | 0.00 | 0.00 | 0.00 | 0.00 | 0.00 | Bacteroidetes | Cytophagia | Cytophagales | Flammeovirgaceae | Transient-rare taxa |
| OTU 2065 | 0.00 | 0.00 | 0.00 | 0.01 | 0.00 | 0.00 | 0.00 | 0.00 | 0.00 | 0.01 | 0.00 | 0.00 | Firmicutes | Clostridia | Clostridiales | Clostridiales_Incertae Sedis XI | Transient-rare taxa |
| OTU 2066 | 0.01 | 0.00 | 0.00 | 0.00 | 0.00 | 0.00 | 0.00 | 0.00 | 0.00 | 0.00 | 0.01 | 0.00 | Proteobacteria | Deltaproteobacteria | Myxococcales | Sorangiineae | Transient-rare taxa |
| OTU 2067 | 0.02 | 0.00 | 0.00 | 0.00 | 0.00 | 0.00 | 0.00 | 0.00 | 0.00 | 0.00 | 0.00 | 0.00 | Gemmatimonadetes | Gemmatimonadetes | Gemmatimonadales | Gemmatimonadaceae | Transient-rare taxa |
| OTU 2068 | 0.00 | 0.02 | 0.00 | 0.00 | 0.00 | 0.00 | 0.00 | 0.00 | 0.00 | 0.00 | 0.00 | 0.00 | Proteobacteria | Betaproteobacteria | Burkholderiales | Burkholderiales_incertae_sedis | Transient-rare taxa |
| OTU 2069 | 0.00 | 0.01 | 0.00 | 0.00 | 0.00 | 0.00 | 0.00 | 0.01 | 0.00 | 0.00 | 0.00 | 0.00 | Chloroflexi | Anaerolineae | Anaerolineales | Anaerolineaceae | Transient-rare taxa |
| OTU 2070 | 0.01 | 0.00 | 0.00 | 0.00 | 0.00 | 0.00 | 0.00 | 0.00 | 0.00 | 0.00 | 0.01 | 0.00 | Proteobacteria | Gammaproteobacteria | Xanthomonadales | Xanthomonadaceae | Transient-rare taxa |
| OTU 2071 | 0.00 | 0.01 | 0.00 | 0.00 | 0.00 | 0.00 | 0.01 | 0.00 | 0.00 | 0.00 | 0.00 | 0.00 | Proteobacteria | Betaproteobacteria | Burkholderiales | Burkholderiales_incertae_sedis | Transient-rare taxa |
| OTU 2072 | 0.01 | 0.01 | 0.00 | 0.00 | 0.00 | 0.00 | 0.00 | 0.00 | 0.00 | 0.00 | 0.00 | 0.00 | Proteobacteria | Gammaproteobacteria | Chromatiales | Chromatiaceae | Transient-rare taxa |
| OTU 2073 | 0.02 | 0.00 | 0.00 | 0.00 | 0.00 | 0.00 | 0.00 | 0.00 | 0.00 | 0.00 | 0.00 | 0.00 | Proteobacteria | Deltaproteobacteria | Desulfobacterales | Desulfobacteraceae | Transient-rare taxa |
| OTU 2074 | 0.01 | 0.00 | 0.00 | 0.00 | 0.01 | 0.00 | 0.00 | 0.00 | 0.00 | 0.00 | 0.00 | 0.00 | Bacteroidetes | Flavobacteriia | Flavobacteriales | Flavobacteriaceae | Transient-rare taxa |
| OTU 2075 | 0.00 | 0.00 | 0.00 | 0.00 | 0.00 | 0.00 | 0.00 | 0.00 | 0.00 | 0.02 | 0.00 | 0.00 | Thermodesulfobacteria | Thermodesulfobacteria | Thermodesulfobacteriales | Thermodesulfobacteriaceae | Transient-rare taxa |
| OTU 2076 | 0.00 | 0.00 | 0.00 | 0.00 | 0.00 | 0.00 | 0.00 | 0.01 | 0.01 | 0.00 | 0.00 | 0.00 | Proteobacteria | Alphaproteobacteria | Sphingomonadales | Sphingomonadaceae | Transient-rare taxa |
| OTU 2077 | 0.00 | 0.00 | 0.00 | 0.00 | 0.00 | 0.00 | 0.00 | 0.01 | 0.01 | 0.00 | 0.00 | 0.00 | Chloroflexi | Anaerolineae | Anaerolineales | Anaerolineaceae | Transient-rare taxa |
| OTU 2078 | 0.01 | 0.00 | 0.00 | 0.00 | 0.00 | 0.00 | 0.00 | 0.00 | 0.01 | 0.00 | 0.00 | 0.00 | Proteobacteria | Alphaproteobacteria | Sphingomonadales | Sphingomonadaceae | Transient-rare taxa |
| OTU 2079 | 0.00 | 0.01 | 0.00 | 0.00 | 0.00 | 0.01 | 0.00 | 0.00 | 0.00 | 0.00 | 0.00 | 0.00 | Planctomycetes | Planctomycetia | Planctomycetales | Planctomycetaceae | Transient-rare taxa |
| OTU 2080 | 0.00 | 0.00 | 0.00 | 0.00 | 0.00 | 0.00 | 0.00 | 0.00 | 0.00 | 0.00 | 0.00 | 0.02 | Proteobacteria | Alphaproteobacteria | Rhodobacterales | Rhodobacteraceae | Transient-rare taxa |
| OTU 2081 | 0.00 | 0.02 | 0.00 | 0.00 | 0.00 | 0.00 | 0.00 | 0.00 | 0.00 | 0.00 | 0.00 | 0.00 | Ignavibacteriae | Ignavibacteria | Ignavibacteriales | Ignavibacteriaceae | Transient-rare taxa |
| OTU 2082 | 0.00 | 0.02 | 0.00 | 0.00 | 0.00 | 0.00 | 0.00 | 0.00 | 0.00 | 0.00 | 0.00 | 0.00 | Proteobacteria | Gammaproteobacteria | Chromatiales | Ectothiorhodospiraceae | Transient-rare taxa |
| OTU 2083 | 0.01 | 0.00 | 0.00 | 0.00 | 0.01 | 0.00 | 0.00 | 0.00 | 0.00 | 0.00 | 0.00 | 0.00 | Proteobacteria | Betaproteobacteria | Rhodocyclales | Rhodocyclaceae | Transient-rare taxa |
| OTU 2084 | 0.01 | 0.00 | 0.00 | 0.00 | 0.00 | 0.00 | 0.00 | 0.00 | 0.00 | 0.00 | 0.00 | 0.01 | Planctomycetes | Planctomycetia | Planctomycetales | Planctomycetaceae | Transient-rare taxa |
| OTU 2085 | 0.01 | 0.00 | 0.00 | 0.00 | 0.00 | 0.00 | 0.00 | 0.00 | 0.01 | 0.00 | 0.00 | 0.00 | Firmicutes | Clostridia | Natranaerobiales | Natranaerobiaceae | Transient-rare taxa |
| OTU 2086 | 0.02 | 0.00 | 0.00 | 0.00 | 0.00 | 0.00 | 0.00 | 0.00 | 0.00 | 0.00 | 0.00 | 0.00 | Proteobacteria | Betaproteobacteria | Rhodocyclales | Rhodocyclaceae | Transient-rare taxa |
| OTU 2087 | 0.02 | 0.00 | 0.00 | 0.00 | 0.00 | 0.00 | 0.00 | 0.00 | 0.00 | 0.00 | 0.00 | 0.00 | Proteobacteria | Gammaproteobacteria | Xanthomonadales | Xanthomonadaceae | Transient-rare taxa |
| OTU 2088 | 0.02 | 0.00 | 0.00 | 0.00 | 0.00 | 0.00 | 0.00 | 0.00 | 0.00 | 0.00 | 0.00 | 0.00 | Proteobacteria | Deltaproteobacteria | Myxococcales | Sorangiineae | Transient-rare taxa |
| OTU 2089 | 0.00 | 0.00 | 0.00 | 0.00 | 0.00 | 0.00 | 0.01 | 0.00 | 0.00 | 0.01 | 0.00 | 0.00 | Proteobacteria | Betaproteobacteria | Burkholderiales | Burkholderiales_incertae_sedis | Transient-rare taxa |
| OTU 2090 | 0.00 | 0.00 | 0.00 | 0.00 | 0.00 | 0.00 | 0.00 | 0.00 | 0.00 | 0.02 | 0.00 | 0.00 | Proteobacteria | Alphaproteobacteria | Rhodobacterales | Rhodobacteraceae | Transient-rare taxa |
| OTU 2091 | 0.00 | 0.00 | 0.00 | 0.00 | 0.00 | 0.00 | 0.01 | 0.00 | 0.01 | 0.00 | 0.00 | 0.00 | Proteobacteria | Betaproteobacteria | Burkholderiales | Burkholderiaceae | Transient-rare taxa |
| OTU 2092 | 0.02 | 0.00 | 0.00 | 0.00 | 0.00 | 0.00 | 0.00 | 0.00 | 0.00 | 0.00 | 0.00 | 0.00 | Proteobacteria | Betaproteobacteria | Burkholderiales | Burkholderiales_incertae_sedis | Transient-rare taxa |
| OTU 2093 | 0.01 | 0.00 | 0.00 | 0.00 | 0.00 | 0.00 | 0.00 | 0.00 | 0.00 | 0.00 | 0.01 | 0.00 | Proteobacteria | Gammaproteobacteria | Xanthomonadales | Xanthomonadaceae | Transient-rare taxa |
| OTU 2094 | 0.00 | 0.00 | 0.00 | 0.00 | 0.00 | 0.01 | 0.00 | 0.00 | 0.00 | 0.01 | 0.00 | 0.00 | Bacteroidetes | Sphingobacteriia | Sphingobacteriales | Sphingobacteriaceae | Transient-rare taxa |
| OTU 2095 | 0.00 | 0.00 | 0.00 | 0.00 | 0.00 | 0.00 | 0.00 | 0.00 | 0.01 | 0.00 | 0.00 | 0.01 | Proteobacteria | Gammaproteobacteria | Pseudomonadales | Pseudomonadaceae | Transient-rare taxa |
| OTU 2096 | 0.01 | 0.00 | 0.00 | 0.00 | 0.00 | 0.00 | 0.00 | 0.00 | 0.00 | 0.00 | 0.00 | 0.01 | Proteobacteria | Betaproteobacteria | Rhodocyclales | Rhodocyclaceae | Transient-rare taxa |
| OTU 2097 | 0.01 | 0.01 | 0.00 | 0.00 | 0.00 | 0.00 | 0.00 | 0.00 | 0.00 | 0.00 | 0.00 | 0.00 | Proteobacteria | Gammaproteobacteria | Chromatiales | Chromatiaceae | Transient-rare taxa |
| OTU 2098 | 0.02 | 0.00 | 0.00 | 0.00 | 0.00 | 0.00 | 0.00 | 0.00 | 0.00 | 0.00 | 0.00 | 0.00 | Proteobacteria | Gammaproteobacteria | Chromatiales | Chromatiaceae | Transient-rare taxa |
| OTU 2099 | 0.02 | 0.00 | 0.00 | 0.00 | 0.00 | 0.00 | 0.00 | 0.00 | 0.00 | 0.00 | 0.00 | 0.00 | Proteobacteria | Gammaproteobacteria | Xanthomonadales | Xanthomonadaceae | Transient-rare taxa |
| OTU 2100 | 0.00 | 0.00 | 0.00 | 0.00 | 0.00 | 0.00 | 0.00 | 0.01 | 0.01 | 0.00 | 0.00 | 0.00 | Proteobacteria | Gammaproteobacteria | Xanthomonadales | Xanthomonadaceae | Transient-rare taxa |
| OTU 2101 | 0.02 | 0.00 | 0.00 | 0.00 | 0.00 | 0.00 | 0.00 | 0.00 | 0.00 | 0.00 | 0.00 | 0.00 | Proteobacteria | Betaproteobacteria | Burkholderiales | Burkholderiales_incertae_sedis | Transient-rare taxa |
| OTU 2102 | 0.00 | 0.00 | 0.00 | 0.00 | 0.00 | 0.00 | 0.00 | 0.01 | 0.00 | 0.01 | 0.00 | 0.00 | Proteobacteria | Alphaproteobacteria | Rhizobiales | Rhodobiaceae | Transient-rare taxa |
| OTU 2103 | 0.00 | 0.00 | 0.00 | 0.00 | 0.00 | 0.00 | 0.00 | 0.00 | 0.00 | 0.00 | 0.00 | 0.02 | Synergistetes | Synergistia | Synergistales | Synergistaceae | Transient-rare taxa |
| OTU 2104 | 0.00 | 0.00 | 0.00 | 0.00 | 0.00 | 0.00 | 0.00 | 0.02 | 0.00 | 0.00 | 0.00 | 0.00 | Bacteroidetes | Sphingobacteriia | Sphingobacteriales | Cyclobacteriaceae | Transient-rare taxa |
| OTU 2105 | 0.00 | 0.00 | 0.00 | 0.00 | 0.00 | 0.00 | 0.00 | 0.02 | 0.00 | 0.00 | 0.00 | 0.00 | Proteobacteria | Betaproteobacteria | Rhodocyclales | Rhodocyclaceae | Transient-rare taxa |
| OTU 2106 | 0.02 | 0.00 | 0.00 | 0.00 | 0.00 | 0.00 | 0.00 | 0.00 | 0.00 | 0.00 | 0.00 | 0.00 | Actinobacteria | Actinobacteria | Acidimicrobidae | Acidimicrobiales | Transient-rare taxa |
| OTU 2107 | 0.02 | 0.00 | 0.00 | 0.00 | 0.00 | 0.00 | 0.00 | 0.00 | 0.00 | 0.00 | 0.00 | 0.00 | Proteobacteria | Deltaproteobacteria | Desulfuromonadales | Desulfuromonadaceae | Transient-rare taxa |
| OTU 2108 | 0.00 | 0.00 | 0.00 | 0.00 | 0.00 | 0.00 | 0.00 | 0.00 | 0.00 | 0.00 | 0.00 | 0.02 | Bacteroidetes | Flavobacteriia | Flavobacteriales | Flavobacteriaceae | Transient-rare taxa |
| OTU 2109 | 0.01 | 0.00 | 0.00 | 0.00 | 0.00 | 0.00 | 0.00 | 0.00 | 0.00 | 0.00 | 0.01 | 0.00 | Proteobacteria | Deltaproteobacteria | Bdellovibrionales | Bdellovibrionaceae | Transient-rare taxa |
| OTU 2110 | 0.02 | 0.00 | 0.00 | 0.00 | 0.00 | 0.00 | 0.00 | 0.00 | 0.00 | 0.00 | 0.00 | 0.00 | Proteobacteria | Betaproteobacteria | Burkholderiales | Comamonadaceae | Transient-rare taxa |
| OTU 2111 | 0.01 | 0.01 | 0.00 | 0.00 | 0.00 | 0.00 | 0.00 | 0.00 | 0.00 | 0.00 | 0.00 | 0.00 | Proteobacteria | Betaproteobacteria | Rhodocyclales | Rhodocyclaceae | Transient-rare taxa |
| OTU 2112 | 0.02 | 0.00 | 0.00 | 0.00 | 0.00 | 0.00 | 0.00 | 0.00 | 0.00 | 0.00 | 0.00 | 0.00 | Parcubacteria | Parcubacteria_genera_incertae_sedis | | | Transient-rare taxa |
| OTU 2113 | 0.00 | 0.00 | 0.00 | 0.00 | 0.00 | 0.00 | 0.00 | 0.00 | 0.01 | 0.01 | 0.00 | 0.00 | Proteobacteria | Deltaproteobacteria | Bdellovibrionales | Bdellovibrionaceae | Transient-rare taxa |
| OTU 2114 | 0.00 | 0.00 | 0.01 | 0.00 | 0.00 | 0.00 | 0.00 | 0.00 | 0.01 | 0.00 | 0.00 | 0.00 | Proteobacteria | Gammaproteobacteria | Pseudomonadales | Moraxellaceae | Transient-rare taxa |
| OTU 2115 | 0.01 | 0.00 | 0.00 | 0.00 | 0.00 | 0.00 | 0.00 | 0.00 | 0.00 | 0.00 | 0.01 | 0.00 | Proteobacteria | Betaproteobacteria | Rhodocyclales | Rhodocyclaceae | Transient-rare taxa |
| OTU 2116 | 0.02 | 0.00 | 0.00 | 0.00 | 0.00 | 0.00 | 0.00 | 0.00 | 0.00 | 0.00 | 0.00 | 0.00 | Bacteroidetes | Sphingobacteriia | Sphingobacteriales | Cyclobacteriaceae | Transient-rare taxa |
| OTU 2117 | 0.00 | 0.00 | 0.00 | 0.01 | 0.00 | 0.00 | 0.00 | 0.00 | 0.00 | 0.01 | 0.00 | 0.00 | Armatimonadetes | Armatimonadetes_gp5 | | | Transient-rare taxa |
| OTU 2118 | 0.00 | 0.00 | 0.00 | 0.00 | 0.00 | 0.00 | 0.00 | 0.00 | 0.01 | 0.00 | 0.01 | 0.00 | Proteobacteria | Alphaproteobacteria | Sphingomonadales | Sphingomonadaceae | Transient-rare taxa |
| OTU 2119 | 0.02 | 0.00 | 0.00 | 0.00 | 0.00 | 0.00 | 0.00 | 0.00 | 0.00 | 0.00 | 0.00 | 0.00 | Bacteroidetes | Sphingobacteriia | Sphingobacteriales | Saprospiraceae | Transient-rare taxa |
| OTU 2120 | 0.00 | 0.00 | 0.00 | 0.00 | 0.00 | 0.00 | 0.00 | 0.01 | 0.01 | 0.00 | 0.00 | 0.00 | Proteobacteria | Alphaproteobacteria | Rhodobacterales | Rhodobacteraceae | Transient-rare taxa |
| OTU 2121 | 0.00 | 0.00 | 0.00 | 0.00 | 0.00 | 0.00 | 0.00 | 0.00 | 0.02 | 0.00 | 0.00 | 0.00 | Actinobacteria | Actinobacteria | Acidimicrobidae | Acidimicrobiales | Transient-rare taxa |
| OTU 2122 | 0.00 | 0.00 | 0.01 | 0.00 | 0.00 | 0.00 | 0.00 | 0.00 | 0.01 | 0.00 | 0.00 | 0.00 | Proteobacteria | Alphaproteobacteria | Sphingomonadales | Sphingomonadaceae | Transient-rare taxa |

| | | | | | | | | | | | | | | | | |
|---|---|---|---|---|---|---|---|---|---|---|---|---|---|---|---|---|
| OTU 2123 | 0.00 | 0.00 | 0.00 | 0.00 | 0.00 | 0.00 | 0.00 | 0.00 | 0.01 | 0.00 | 0.01 | 0.00 | Proteobacteria | Betaproteobacteria | Rhodocyclales | Rhodocyclaceae | Transient-rare taxa |
| OTU 2124 | 0.00 | 0.00 | 0.00 | 0.00 | 0.01 | 0.00 | 0.00 | 0.00 | 0.01 | 0.00 | 0.00 | 0.00 | Proteobacteria | Gammaproteobacteria | Xanthomonadales | Xanthomonadaceae | Transient-rare taxa |
| OTU 2125 | 0.01 | 0.01 | 0.00 | 0.00 | 0.00 | 0.00 | 0.00 | 0.00 | 0.00 | 0.00 | 0.00 | 0.00 | Proteobacteria | Gammaproteobacteria | Chromatiales | Chromatiaceae | Transient-rare taxa |
| OTU 2126 | 0.00 | 0.00 | 0.00 | 0.00 | 0.00 | 0.00 | 0.00 | 0.00 | 0.00 | 0.01 | 0.01 | 0.00 | Proteobacteria | Betaproteobacteria | Rhodocyclales | Rhodocyclaceae | Transient-rare taxa |
| OTU 2127 | 0.00 | 0.00 | 0.00 | 0.00 | 0.00 | 0.00 | 0.00 | 0.00 | 0.00 | 0.00 | 0.00 | 0.02 | Planctomycetes | Planctomycetia | Planctomycetales | Planctomycetaceae | Transient-rare taxa |
| OTU 2128 | 0.00 | 0.00 | 0.00 | 0.00 | 0.00 | 0.00 | 0.00 | 0.02 | 0.00 | 0.00 | 0.00 | 0.00 | Proteobacteria | Gammaproteobacteria | Thiotrichales | Thiotrichaceae | Transient-rare taxa |
| OTU 2129 | 0.00 | 0.00 | 0.00 | 0.00 | 0.00 | 0.00 | 0.00 | 0.02 | 0.00 | 0.00 | 0.00 | 0.00 | Proteobacteria | Deltaproteobacteria | Bdellovibrionales | Bacteriovoracaceae | Transient-rare taxa |
| OTU 2130 | 0.00 | 0.00 | 0.00 | 0.00 | 0.00 | 0.00 | 0.01 | 0.01 | 0.00 | 0.00 | 0.00 | 0.00 | Nitrospirae | Nitrospira | Nitrospirales | Nitrospiraceae | Transient-rare taxa |
| OTU 2131 | 0.01 | 0.00 | 0.00 | 0.00 | 0.00 | 0.00 | 0.00 | 0.00 | 0.00 | 0.00 | 0.01 | 0.00 | Proteobacteria | Gammaproteobacteria | Xanthomonadales | Xanthomonadaceae | Transient-rare taxa |
| OTU 2132 | 0.00 | 0.01 | 0.00 | 0.00 | 0.01 | 0.00 | 0.00 | 0.00 | 0.00 | 0.00 | 0.00 | 0.00 | Proteobacteria | Betaproteobacteria | Rhodocyclales | Rhodocyclaceae | Transient-rare taxa |
| OTU 2133 | 0.00 | 0.01 | 0.00 | 0.00 | 0.00 | 0.00 | 0.00 | 0.00 | 0.00 | 0.01 | 0.00 | 0.00 | Bacteroidetes | Sphingobacteriia | Sphingobacteriales | Cyclobacteriaceae | Transient-rare taxa |
| OTU 2134 | 0.00 | 0.01 | 0.00 | 0.00 | 0.00 | 0.00 | 0.00 | 0.00 | 0.01 | 0.00 | 0.00 | 0.00 | Proteobacteria | Betaproteobacteria | Nitrosomonadales | Nitrosomonadaceae | Transient-rare taxa |
| OTU 2135 | 0.00 | 0.02 | 0.00 | 0.00 | 0.00 | 0.00 | 0.00 | 0.00 | 0.00 | 0.00 | 0.00 | 0.00 | Planctomycetes | Planctomycetia | Planctomycetales | Planctomycetaceae | Transient-rare taxa |
| OTU 2136 | 0.00 | 0.00 | 0.01 | 0.00 | 0.00 | 0.00 | 0.00 | 0.00 | 0.01 | 0.00 | 0.00 | 0.00 | Proteobacteria | Deltaproteobacteria | Myxococcales | Sorangiineae | Transient-rare taxa |
| OTU 2137 | 0.00 | 0.00 | 0.00 | 0.00 | 0.00 | 0.00 | 0.00 | 0.00 | 0.02 | 0.00 | 0.00 | 0.00 | Proteobacteria | Betaproteobacteria | Rhodocyclales | Rhodocyclaceae | Transient-rare taxa |
| OTU 2138 | 0.00 | 0.00 | 0.00 | 0.00 | 0.00 | 0.00 | 0.00 | 0.00 | 0.00 | 0.00 | 0.01 | 0.01 | Proteobacteria | Betaproteobacteria | Rhodocyclales | Rhodocyclaceae | Transient-rare taxa |
| OTU 2139 | 0.00 | 0.00 | 0.00 | 0.00 | 0.00 | 0.00 | 0.00 | 0.00 | 0.00 | 0.00 | 0.00 | 0.02 | Proteobacteria | Betaproteobacteria | Rhodocyclales | Rhodocyclaceae | Transient-rare taxa |
| OTU 2140 | 0.00 | 0.00 | 0.00 | 0.00 | 0.00 | 0.00 | 0.00 | 0.00 | 0.00 | 0.00 | 0.02 | 0.00 | Proteobacteria | Betaproteobacteria | Burkholderiales | Comamonadaceae | Transient-rare taxa |
| OTU 2141 | 0.00 | 0.00 | 0.00 | 0.00 | 0.00 | 0.00 | 0.00 | 0.00 | 0.00 | 0.00 | 0.02 | 0.00 | Proteobacteria | Alphaproteobacteria | Rhodospirillales | Acetobacteraceae | Transient-rare taxa |
| OTU 2142 | 0.00 | 0.00 | 0.00 | 0.00 | 0.00 | 0.00 | 0.00 | 0.00 | 0.00 | 0.01 | 0.01 | 0.00 | Bacteroidetes | Sphingobacteriia | Sphingobacteriales | Saprospiraceae | Transient-rare taxa |
| OTU 2143 | 0.00 | 0.00 | 0.00 | 0.00 | 0.00 | 0.00 | 0.00 | 0.00 | 0.00 | 0.00 | 0.01 | 0.01 | Proteobacteria | Gammaproteobacteria | Xanthomonadales | Xanthomonadaceae | Transient-rare taxa |
| OTU 2144 | 0.00 | 0.00 | 0.00 | 0.00 | 0.00 | 0.00 | 0.00 | 0.00 | 0.00 | 0.00 | 0.00 | 0.02 | Bacteroidetes | Flavobacteriia | Flavobacteriales | Cryomorphaceae | Transient-rare taxa |
| OTU 2145 | 0.00 | 0.00 | 0.00 | 0.00 | 0.00 | 0.00 | 0.00 | 0.00 | 0.00 | 0.00 | 0.00 | 0.02 | Acidobacteria | Holophagae | Holophagales | Holophagaceae | Transient-rare taxa |
| OTU 2146 | 0.00 | 0.00 | 0.00 | 0.00 | 0.00 | 0.00 | 0.01 | 0.00 | 0.00 | 0.00 | 0.00 | 0.01 | Hydrogenedentes | Candidatus Hydrogenedens | | | Transient-rare taxa |
| OTU 2147 | 0.00 | 0.00 | 0.02 | 0.00 | 0.00 | 0.00 | 0.00 | 0.00 | 0.00 | 0.00 | 0.00 | 0.00 | Bacteroidetes | Flavobacteriia | Flavobacteriales | Cryomorphaceae | Transient-rare taxa |
| OTU 2148 | 0.00 | 0.00 | 0.00 | 0.00 | 0.00 | 0.00 | 0.00 | 0.00 | 0.00 | 0.00 | 0.02 | 0.00 | Firmicutes | Clostridia | Clostridiales | Ruminococcaceae | Transient-rare taxa |
| OTU 2149 | 0.00 | 0.01 | 0.00 | 0.00 | 0.00 | 0.00 | 0.00 | 0.00 | 0.00 | 0.00 | 0.00 | 0.01 | Actinobacteria | Actinobacteria | Actinobacteridae | Actinomycetales | Transient-rare taxa |
| OTU 2150 | 0.00 | 0.02 | 0.00 | 0.00 | 0.00 | 0.00 | 0.00 | 0.00 | 0.00 | 0.00 | 0.00 | 0.00 | Proteobacteria | Betaproteobacteria | Rhodocyclales | Rhodocyclaceae | Transient-rare taxa |
| OTU 2151 | 0.00 | 0.02 | 0.00 | 0.00 | 0.00 | 0.00 | 0.00 | 0.00 | 0.00 | 0.00 | 0.00 | 0.00 | Proteobacteria | Gammaproteobacteria | Chromatiales | Ectothiorhodospiraceae | Transient-rare taxa |
| OTU 2152 | 0.00 | 0.02 | 0.00 | 0.00 | 0.00 | 0.00 | 0.00 | 0.00 | 0.00 | 0.00 | 0.00 | 0.00 | Proteobacteria | Gammaproteobacteria | Xanthomonadales | Xanthomonadaceae | Transient-rare taxa |
| OTU 2153 | 0.00 | 0.00 | 0.00 | 0.02 | 0.00 | 0.00 | 0.00 | 0.00 | 0.00 | 0.00 | 0.00 | 0.00 | Proteobacteria | Gammaproteobacteria | Oceanospirillales | Oceanospirillaceae | Transient-rare taxa |
| OTU 2154 | 0.00 | 0.00 | 0.00 | 0.01 | 0.01 | 0.00 | 0.00 | 0.00 | 0.00 | 0.00 | 0.00 | 0.00 | Proteobacteria | Alphaproteobacteria | Rhodobacterales | Rhodobacteraceae | Transient-rare taxa |
| OTU 2155 | 0.00 | 0.00 | 0.01 | 0.00 | 0.00 | 0.00 | 0.00 | 0.00 | 0.00 | 0.00 | 0.01 | 0.00 | Firmicutes | Clostridia | Clostridiales | Peptostreptococcaceae | Transient-rare taxa |
| OTU 2156 | 0.00 | 0.00 | 0.01 | 0.00 | 0.00 | 0.00 | 0.01 | 0.00 | 0.00 | 0.00 | 0.00 | 0.00 | Bacteroidetes | Flavobacteriia | Flavobacteriales | Flavobacteriaceae | Transient-rare taxa |
| OTU 2157 | 0.00 | 0.00 | 0.00 | 0.00 | 0.00 | 0.00 | 0.00 | 0.00 | 0.00 | 0.00 | 0.01 | 0.01 | Proteobacteria | Betaproteobacteria | Rhodocyclales | Rhodocyclaceae | Transient-rare taxa |
| OTU 2158 | 0.00 | 0.00 | 0.00 | 0.00 | 0.00 | 0.00 | 0.00 | 0.00 | 0.00 | 0.00 | 0.01 | 0.01 | Proteobacteria | Gammaproteobacteria | Xanthomonadales | Xanthomonadaceae | Transient-rare taxa |
| OTU 2159 | 0.00 | 0.00 | 0.02 | 0.00 | 0.00 | 0.00 | 0.00 | 0.00 | 0.00 | 0.00 | 0.00 | 0.00 | Proteobacteria | Gammaproteobacteria | Thiotrichales | Piscirickettsiaceae | Transient-rare taxa |
| OTU 2160 | 0.00 | 0.00 | 0.00 | 0.00 | 0.00 | 0.00 | 0.00 | 0.00 | 0.00 | 0.01 | 0.00 | 0.01 | Proteobacteria | Deltaproteobacteria | Myxococcales | Cystobacterineae | Transient-rare taxa |
| OTU 2161 | 0.00 | 0.00 | 0.01 | 0.00 | 0.00 | 0.00 | 0.00 | 0.00 | 0.01 | 0.00 | 0.00 | 0.00 | Actinobacteria | Actinobacteria | Rubrobacteridae | Gaiellales | Transient-rare taxa |
| OTU 2162 | 0.00 | 0.01 | 0.01 | 0.00 | 0.00 | 0.00 | 0.00 | 0.00 | 0.00 | 0.00 | 0.00 | 0.00 | Proteobacteria | Gammaproteobacteria | Chromatiales | Halothiobacillaceae | Transient-rare taxa |
| OTU 2163 | 0.00 | 0.00 | 0.00 | 0.00 | 0.00 | 0.00 | 0.00 | 0.00 | 0.02 | 0.00 | 0.00 | 0.00 | Proteobacteria | Deltaproteobacteria | Bdellovibrionales | Bacteriovoracaceae | Transient-rare taxa |
| OTU 2164 | 0.00 | 0.00 | 0.00 | 0.00 | 0.00 | 0.00 | 0.00 | 0.00 | 0.02 | 0.00 | 0.00 | 0.00 | Firmicutes | Clostridia | Clostridiales | Ruminococcaceae | Transient-rare taxa |
| OTU 2165 | 0.00 | 0.00 | 0.00 | 0.00 | 0.00 | 0.00 | 0.00 | 0.00 | 0.00 | 0.01 | 0.00 | 0.01 | Bacteroidetes | Sphingobacteriia | Sphingobacteriales | Sphingobacteriaceae | Transient-rare taxa |
| OTU 2166 | 0.00 | 0.00 | 0.00 | 0.02 | 0.00 | 0.00 | 0.00 | 0.00 | 0.00 | 0.00 | 0.00 | 0.00 | Bacteroidetes | Flavobacteriia | Flavobacteriales | Cryomorphaceae | Transient-rare taxa |
| OTU 2167 | 0.00 | 0.00 | 0.00 | 0.00 | 0.00 | 0.00 | 0.00 | 0.00 | 0.02 | 0.00 | 0.00 | 0.00 | Proteobacteria | Betaproteobacteria | Burkholderiales | Comamonadaceae | Transient-rare taxa |
| OTU 2168 | 0.02 | 0.00 | 0.00 | 0.00 | 0.00 | 0.00 | 0.00 | 0.00 | 0.00 | 0.00 | 0.00 | 0.00 | Proteobacteria | Alphaproteobacteria | Rhodobacterales | Rhodobacteraceae | Transient-rare taxa |
| OTU 2169 | 0.00 | 0.00 | 0.02 | 0.00 | 0.00 | 0.00 | 0.00 | 0.00 | 0.00 | 0.00 | 0.00 | 0.00 | Proteobacteria | Gammaproteobacteria | Gammaproteobacteria_incertae_sedis | Simiduia | Transient-rare taxa |
| OTU 2170 | 0.01 | 0.00 | 0.00 | 0.00 | 0.00 | 0.00 | 0.00 | 0.00 | 0.00 | 0.00 | 0.01 | 0.00 | Acidobacteria | Acidobacteria_Gp4 | Gp4 | | Transient-rare taxa |
| OTU 2171 | 0.01 | 0.00 | 0.00 | 0.00 | 0.00 | 0.00 | 0.00 | 0.00 | 0.00 | 0.01 | 0.00 | 0.00 | Bacteroidetes | Sphingobacteriia | Sphingobacteriales | Chitinophagaceae | Transient-rare taxa |
| OTU 2172 | 0.00 | 0.00 | 0.01 | 0.00 | 0.00 | 0.00 | 0.00 | 0.01 | 0.00 | 0.00 | 0.00 | 0.00 | Proteobacteria | Gammaproteobacteria | Chromatiales | Ectothiorhodospiraceae | Transient-rare taxa |
| OTU 2173 | 0.01 | 0.00 | 0.00 | 0.00 | 0.00 | 0.00 | 0.00 | 0.00 | 0.00 | 0.00 | 0.01 | 0.00 | Proteobacteria | Gammaproteobacteria | Aeromonadales | Aeromonadaceae | Transient-rare taxa |
| OTU 2174 | 0.00 | 0.00 | 0.00 | 0.00 | 0.00 | 0.00 | 0.00 | 0.00 | 0.00 | 0.00 | 0.01 | 0.01 | Proteobacteria | Betaproteobacteria | Rhodocyclales | Rhodocyclaceae | Transient-rare taxa |
| OTU 2175 | 0.02 | 0.00 | 0.00 | 0.00 | 0.00 | 0.00 | 0.00 | 0.00 | 0.00 | 0.00 | 0.00 | 0.00 | Proteobacteria | Alphaproteobacteria | Rhodobacterales | Rhodobacteraceae | Transient-rare taxa |
| OTU 2176 | 0.02 | 0.00 | 0.00 | 0.00 | 0.00 | 0.00 | 0.00 | 0.00 | 0.00 | 0.00 | 0.00 | 0.00 | Proteobacteria | Betaproteobacteria | Rhodocyclales | Rhodocyclaceae | Transient-rare taxa |
| OTU 2177 | 0.01 | 0.00 | 0.00 | 0.00 | 0.00 | 0.00 | 0.00 | 0.00 | 0.00 | 0.01 | 0.00 | 0.00 | Proteobacteria | Deltaproteobacteria | Myxococcales | Nannocystineae | Transient-rare taxa |
| OTU 2178 | 0.02 | 0.00 | 0.00 | 0.00 | 0.00 | 0.00 | 0.00 | 0.00 | 0.00 | 0.00 | 0.00 | 0.00 | Proteobacteria | Betaproteobacteria | Rhodocyclales | Rhodocyclaceae | Transient-rare taxa |
| OTU 2179 | 0.01 | 0.00 | 0.00 | 0.00 | 0.00 | 0.00 | 0.00 | 0.01 | 0.00 | 0.00 | 0.00 | 0.00 | Proteobacteria | Betaproteobacteria | Rhodocyclales | Rhodocyclaceae | Transient-rare taxa |
| OTU 2180 | 0.01 | 0.00 | 0.00 | 0.00 | 0.00 | 0.00 | 0.00 | 0.01 | 0.00 | 0.00 | 0.00 | 0.00 | Proteobacteria | Betaproteobacteria | Burkholderiales | Comamonadaceae | Transient-rare taxa |
| OTU 2181 | 0.00 | 0.00 | 0.00 | 0.00 | 0.00 | 0.00 | 0.00 | 0.00 | 0.02 | 0.00 | 0.00 | 0.00 | Proteobacteria | Deltaproteobacteria | Myxococcales | Sorangiineae | Transient-rare taxa |
| OTU 2182 | 0.00 | 0.01 | 0.00 | 0.00 | 0.00 | 0.00 | 0.00 | 0.01 | 0.00 | 0.00 | 0.00 | 0.00 | Proteobacteria | Gammaproteobacteria | Chromatiales | Chromatiaceae | Transient-rare taxa |
| OTU 2183 | 0.01 | 0.01 | 0.00 | 0.00 | 0.00 | 0.00 | 0.00 | 0.00 | 0.00 | 0.00 | 0.00 | 0.00 | Proteobacteria | Gammaproteobacteria | Chromatiales | Chromatiaceae | Transient-rare taxa |
| OTU 2184 | 0.00 | 0.01 | 0.01 | 0.00 | 0.00 | 0.00 | 0.00 | 0.00 | 0.00 | 0.00 | 0.00 | 0.00 | Acidobacteria | Acidobacteria_Gp6 | Gp6 | | Transient-rare taxa |
| OTU 2185 | 0.00 | 0.01 | 0.00 | 0.00 | 0.00 | 0.00 | 0.00 | 0.00 | 0.00 | 0.01 | 0.00 | 0.00 | Proteobacteria | Betaproteobacteria | Ferrovales | Ferrovaceae | Transient-rare taxa |
| OTU 2186 | 0.00 | 0.01 | 0.00 | 0.00 | 0.01 | 0.00 | 0.00 | 0.00 | 0.00 | 0.00 | 0.00 | 0.00 | Bacteroidetes | Flavobacteriia | Flavobacteriales | Flavobacteriaceae | Transient-rare taxa |
| OTU 2187 | 0.00 | 0.00 | 0.01 | 0.00 | 0.00 | 0.00 | 0.00 | 0.00 | 0.01 | 0.00 | 0.00 | 0.00 | Proteobacteria | Deltaproteobacteria | Myxococcales | Nannocystineae | Transient-rare taxa |
| OTU 2188 | 0.00 | 0.01 | 0.00 | 0.00 | 0.00 | 0.00 | 0.00 | 0.00 | 0.00 | 0.00 | 0.01 | 0.00 | Proteobacteria | Betaproteobacteria | Rhodocyclales | Rhodocyclaceae | Transient-rare taxa |
| OTU 2189 | 0.00 | 0.00 | 0.00 | 0.00 | 0.00 | 0.00 | 0.00 | 0.00 | 0.02 | 0.00 | 0.00 | 0.00 | Bacteroidetes | Sphingobacteriia | Sphingobacteriales | Chitinophagaceae | Transient-rare taxa |
| OTU 2190 | 0.00 | 0.01 | 0.00 | 0.00 | 0.00 | 0.00 | 0.00 | 0.00 | 0.00 | 0.00 | 0.00 | 0.01 | Proteobacteria | Betaproteobacteria | Burkholderiales | Comamonadaceae | Transient-rare taxa |
| OTU 2191 | 0.00 | 0.00 | 0.02 | 0.00 | 0.00 | 0.00 | 0.00 | 0.00 | 0.00 | 0.00 | 0.00 | 0.00 | Planctomycetes | Planctomycetia | Planctomycetales | Planctomycetaceae | Transient-rare taxa |
| OTU 2192 | 0.00 | 0.00 | 0.02 | 0.00 | 0.00 | 0.00 | 0.00 | 0.00 | 0.00 | 0.00 | 0.00 | 0.00 | Proteobacteria | Betaproteobacteria | Burkholderiales | Comamonadaceae | Transient-rare taxa |
| OTU 2193 | 0.00 | 0.02 | 0.00 | 0.00 | 0.00 | 0.00 | 0.00 | 0.00 | 0.00 | 0.00 | 0.00 | 0.00 | Bacteroidetes | Sphingobacteriia | Sphingobacteriales | Chitinophagaceae | Transient-rare taxa |
| OTU 2194 | 0.00 | 0.00 | 0.00 | 0.00 | 0.00 | 0.00 | 0.00 | 0.00 | 0.00 | 0.01 | 0.00 | 0.01 | Proteobacteria | Gammaproteobacteria | Xanthomonadales | Xanthomonadaceae | Transient-rare taxa |
| OTU 2195 | 0.02 | 0.00 | 0.00 | 0.00 | 0.00 | 0.00 | 0.00 | 0.00 | 0.00 | 0.00 | 0.00 | 0.00 | Proteobacteria | Gammaproteobacteria | Chromatiales | Chromatiaceae | Transient-rare taxa |
| OTU 2196 | 0.01 | 0.00 | 0.00 | 0.00 | 0.00 | 0.00 | 0.00 | 0.00 | 0.00 | 0.00 | 0.01 | 0.00 | Proteobacteria | Gammaproteobacteria | Xanthomonadales | Xanthomonadaceae | Transient-rare taxa |
| OTU 2197 | 0.02 | 0.00 | 0.00 | 0.00 | 0.00 | 0.00 | 0.00 | 0.00 | 0.00 | 0.00 | 0.00 | 0.00 | Acidobacteria | Holophagae | Holophagales | Holophagaceae | Transient-rare taxa |
| OTU 2198 | 0.01 | 0.00 | 0.00 | 0.00 | 0.00 | 0.00 | 0.00 | 0.00 | 0.01 | 0.00 | 0.00 | 0.00 | Bacteroidetes | Sphingobacteriia | Sphingobacteriales | Saprospiraceae | Transient-rare taxa |
| OTU 2199 | 0.01 | 0.00 | 0.00 | 0.00 | 0.00 | 0.00 | 0.00 | 0.00 | 0.00 | 0.00 | 0.01 | 0.00 | Fusobacteria | Fusobacteriia | Fusobacteriales | Fusobacteriaceae | Transient-rare taxa |
| OTU 2200 | 0.00 | 0.00 | 0.00 | 0.00 | 0.00 | 0.00 | 0.00 | 0.00 | 0.00 | 0.00 | 0.00 | 0.02 | Proteobacteria | Gammaproteobacteria | Candidatus Carsonella | | Transient-rare taxa |
| OTU 2201 | 0.01 | 0.01 | 0.00 | 0.00 | 0.00 | 0.00 | 0.00 | 0.00 | 0.00 | 0.00 | 0.00 | 0.00 | Bacteroidetes | Sphingobacteriia | Sphingobacteriales | Saprospiraceae | Transient-rare taxa |
| OTU 2202 | 0.00 | 0.02 | 0.00 | 0.00 | 0.00 | 0.00 | 0.00 | 0.00 | 0.00 | 0.00 | 0.00 | 0.00 | Proteobacteria | Alphaproteobacteria | Caulobacterales | Hyphomonadaceae | Transient-rare taxa |
| OTU 2203 | 0.00 | 0.00 | 0.00 | 0.00 | 0.00 | 0.00 | 0.00 | 0.00 | 0.02 | 0.00 | 0.00 | 0.00 | Lentisphaerae | Oligosphaeria | Oligosphaerales | Oligosphaeraceae | Transient-rare taxa |
| OTU 2204 | 0.00 | 0.02 | 0.00 | 0.00 | 0.00 | 0.00 | 0.00 | 0.00 | 0.00 | 0.00 | 0.00 | 0.00 | Chloroflexi | Anaerolineae | Anaerolineales | Anaerolineaceae | Transient-rare taxa |
| OTU 2205 | 0.00 | 0.00 | 0.02 | 0.00 | 0.00 | 0.00 | 0.00 | 0.00 | 0.00 | 0.00 | 0.00 | 0.00 | Proteobacteria | Betaproteobacteria | Rhodocyclales | Rhodocyclaceae | Transient-rare taxa |
| OTU 2206 | 0.00 | 0.00 | 0.00 | 0.00 | 0.00 | 0.00 | 0.00 | 0.00 | 0.02 | 0.00 | 0.00 | 0.00 | Bacteroidetes | Sphingobacteriia | Sphingobacteriales | Chitinophagaceae | Transient-rare taxa |
| OTU 2207 | 0.00 | 0.00 | 0.00 | 0.00 | 0.00 | 0.00 | 0.00 | 0.00 | 0.01 | 0.00 | 0.00 | 0.01 | Proteobacteria | Betaproteobacteria | Rhodocyclales | Rhodocyclaceae | Transient-rare taxa |
| OTU 2208 | 0.00 | 0.00 | 0.00 | 0.00 | 0.00 | 0.00 | 0.00 | 0.00 | 0.01 | 0.01 | 0.00 | 0.00 | Chloroflexi | Anaerolineae | Anaerolineales | Anaerolineaceae | Transient-rare taxa |
| OTU 2209 | 0.00 | 0.00 | 0.02 | 0.00 | 0.00 | 0.00 | 0.00 | 0.00 | 0.00 | 0.00 | 0.00 | 0.00 | Bacteroidetes | Flavobacteriia | Flavobacteriales | Cryomorphaceae | Transient-rare taxa |
| OTU 2210 | 0.00 | 0.00 | 0.00 | 0.00 | 0.00 | 0.00 | 0.00 | 0.00 | 0.00 | 0.00 | 0.01 | 0.01 | Bacteroidetes | Sphingobacteriia | Sphingobacteriales | Chitinophagaceae | Transient-rare taxa |
| OTU 2211 | 0.00 | 0.00 | 0.00 | 0.00 | 0.00 | 0.00 | 0.00 | 0.01 | 0.00 | 0.00 | 0.01 | 0.00 | Proteobacteria | Betaproteobacteria | Rhodocyclales | Rhodocyclaceae | Transient-rare taxa |
| OTU 2212 | 0.00 | 0.00 | 0.00 | 0.00 | 0.00 | 0.00 | 0.00 | 0.00 | 0.00 | 0.00 | 0.02 | 0.00 | Proteobacteria | Gammaproteobacteria | Chromatiales | Ectothiorhodospiraceae | Transient-rare taxa |
| OTU 2213 | 0.00 | 0.00 | 0.00 | 0.00 | 0.00 | 0.00 | 0.00 | 0.00 | 0.00 | 0.00 | 0.02 | 0.00 | Proteobacteria | Deltaproteobacteria | Myxococcales | Nannocystineae | Transient-rare taxa |
| OTU 2214 | 0.00 | 0.00 | 0.00 | 0.01 | 0.00 | 0.00 | 0.00 | 0.00 | 0.00 | 0.00 | 0.01 | 0.00 | Proteobacteria | Gammaproteobacteria | Aeromonadales | Aeromonadaceae | Transient-rare taxa |
| OTU 2215 | 0.00 | 0.00 | 0.00 | 0.00 | 0.00 | 0.00 | 0.00 | 0.00 | 0.00 | 0.02 | 0.00 | 0.00 | Proteobacteria | Epsilonproteobacteria | Campylobacterales | Helicobacteraceae | Transient-rare taxa |
| OTU 2216 | 0.00 | 0.00 | 0.00 | 0.00 | 0.00 | 0.00 | 0.00 | 0.00 | 0.00 | 0.00 | 0.02 | 0.00 | Proteobacteria | Betaproteobacteria | Burkholderiales | Comamonadaceae | Transient-rare taxa |
| OTU 2217 | 0.00 | 0.00 | 0.00 | 0.00 | 0.01 | 0.00 | 0.00 | 0.00 | 0.00 | 0.00 | 0.00 | 0.01 | Proteobacteria | Betaproteobacteria | Ferrovales | Ferrovaceae | Transient-rare taxa |
| OTU 2218 | 0.00 | 0.00 | 0.00 | 0.00 | 0.00 | 0.00 | 0.00 | 0.00 | 0.00 | 0.00 | 0.02 | 0.00 | Proteobacteria | Deltaproteobacteria | Myxococcales | Nannocystineae | Transient-rare taxa |
| OTU 2219 | 0.00 | 0.00 | 0.00 | 0.00 | 0.00 | 0.00 | 0.00 | 0.00 | 0.00 | 0.02 | 0.00 | 0.00 | Bacteroidetes | Bacteroidia | Bacteroidales | Sunxiuqinia | Transient-rare taxa |
| OTU 2220 | 0.00 | 0.00 | 0.00 | 0.00 | 0.01 | 0.00 | 0.01 | 0.00 | 0.00 | 0.00 | 0.00 | 0.00 | Proteobacteria | Betaproteobacteria | Burkholderiales | Alcaligenaceae | Transient-rare taxa |
| OTU 2221 | 0.00 | 0.00 | 0.00 | 0.00 | 0.00 | 0.00 | 0.00 | 0.00 | 0.00 | 0.01 | 0.00 | 0.01 | Proteobacteria | Gammaproteobacteria | Methylococcales | Methylococcaceae | Transient-rare taxa |
| OTU 2222 | 0.00 | 0.01 | 0.00 | 0.00 | 0.01 | 0.00 | 0.00 | 0.00 | 0.00 | 0.00 | 0.00 | 0.00 | Bacteroidetes | Sphingobacteriia | Sphingobacteriales | Saprospiraceae | Transient-rare taxa |
| OTU 2223 | 0.00 | 0.00 | 0.00 | 0.00 | 0.02 | 0.00 | 0.00 | 0.00 | 0.00 | 0.00 | 0.00 | 0.00 | Bacteroidetes | Flavobacteriia | Flavobacteriales | Flavobacteriaceae | Transient-rare taxa |
| OTU 2224 | 0.00 | 0.00 | 0.00 | 0.00 | 0.00 | 0.00 | 0.00 | 0.00 | 0.00 | 0.00 | 0.02 | 0.00 | Proteobacteria | Betaproteobacteria | Burkholderiales | Burkholderiales_incertae_sedis | Transient-rare taxa |
| OTU 2225 | 0.00 | 0.00 | 0.00 | 0.00 | 0.00 | 0.00 | 0.00 | 0.02 | 0.00 | 0.00 | 0.00 | 0.00 | Proteobacteria | Gammaproteobacteria | Thiotrichales | Thiotrichaceae | Transient-rare taxa |
| OTU 2226 | 0.00 | 0.00 | 0.00 | 0.00 | 0.00 | 0.01 | 0.00 | 0.00 | 0.00 | 0.01 | 0.00 | 0.00 | Firmicutes | Clostridia | Clostridiales | Clostridiales_Incertae Sedis XIII | Transient-rare taxa |
| OTU 2227 | 0.00 | 0.00 | 0.00 | 0.00 | 0.01 | 0.01 | 0.00 | 0.00 | 0.00 | 0.00 | 0.00 | 0.00 | Proteobacteria | Alphaproteobacteria | Rhizobiales | Beijerinckiaceae | Transient-rare taxa |
| OTU 2228 | 0.00 | 0.00 | 0.00 | 0.00 | 0.00 | 0.00 | 0.00 | 0.00 | 0.00 | 0.02 | 0.00 | 0.00 | Proteobacteria | Deltaproteobacteria | Desulfobacterales | Desulfobacteraceae | Transient-rare taxa |
| OTU 2229 | 0.00 | 0.00 | 0.00 | 0.00 | 0.00 | 0.00 | 0.00 | 0.00 | 0.00 | 0.00 | 0.02 | 0.00 | Proteobacteria | Betaproteobacteria | Rhodocyclales | Rhodocyclaceae | Transient-rare taxa |
| OTU 2230 | 0.00 | 0.00 | 0.00 | 0.00 | 0.00 | 0.00 | 0.00 | 0.00 | 0.00 | 0.01 | 0.01 | 0.00 | Proteobacteria | Alphaproteobacteria | Rhodobacterales | Rhodobacteraceae | Transient-rare taxa |
| OTU 2231 | 0.00 | 0.01 | 0.00 | 0.00 | 0.00 | 0.00 | 0.00 | 0.00 | 0.00 | 0.00 | 0.01 | 0.00 | Proteobacteria | Betaproteobacteria | Burkholderiales | Comamonadaceae | Transient-rare taxa |
| OTU 2232 | 0.00 | 0.00 | 0.00 | 0.00 | 0.00 | 0.00 | 0.00 | 0.00 | 0.00 | 0.00 | 0.02 | 0.00 | Bacteroidetes | Sphingobacteriia | Sphingobacteriales | Saprospiraceae | Transient-rare taxa |
| OTU 2233 | 0.00 | 0.00 | 0.00 | 0.00 | 0.00 | 0.00 | 0.00 | 0.00 | 0.00 | 0.00 | 0.02 | 0.00 | Proteobacteria | Deltaproteobacteria | Myxococcales | Nannocystineae | Transient-rare taxa |
| OTU 2234 | 0.01 | 0.00 | 0.00 | 0.00 | 0.00 | 0.00 | 0.00 | 0.00 | 0.00 | 0.00 | 0.01 | 0.00 | Proteobacteria | Gammaproteobacteria | Pseudomonadales | Pseudomonadaceae | Transient-rare taxa |
| OTU 2235 | 0.00 | 0.00 | 0.00 | 0.00 | 0.00 | 0.00 | 0.00 | 0.00 | 0.00 | 0.00 | 0.02 | 0.00 | Proteobacteria | Deltaproteobacteria | Myxococcales | Nannocystineae | Transient-rare taxa |
| OTU 2236 | 0.00 | 0.00 | 0.00 | 0.00 | 0.00 | 0.00 | 0.00 | 0.00 | 0.00 | 0.00 | 0.02 | 0.00 | Proteobacteria | Betaproteobacteria | Burkholderiales | Comamonadaceae | Transient-rare taxa |
| OTU 2237 | 0.00 | 0.00 | 0.01 | 0.00 | 0.00 | 0.00 | 0.00 | 0.00 | 0.00 | 0.00 | 0.01 | 0.00 | Proteobacteria | Betaproteobacteria | Rhodocyclales | Rhodocyclaceae | Transient-rare taxa |
| OTU 2238 | 0.00 | 0.00 | 0.00 | 0.00 | 0.00 | 0.01 | 0.00 | 0.00 | 0.00 | 0.00 | 0.01 | 0.00 | Proteobacteria | Deltaproteobacteria | Desulfuromonadales | Geobacteraceae | Transient-rare taxa |
| OTU 2239 | 0.00 | 0.00 | 0.00 | 0.00 | 0.00 | 0.00 | 0.00 | 0.00 | 0.00 | 0.00 | 0.02 | 0.00 | Proteobacteria | Deltaproteobacteria | Myxococcales | Nannocystineae | Transient-rare taxa |
| OTU 2240 | 0.00 | 0.00 | 0.00 | 0.01 | 0.01 | 0.00 | 0.00 | 0.00 | 0.00 | 0.00 | 0.00 | 0.00 | Bacteroidetes | Flavobacteriia | Flavobacteriales | Flavobacteriaceae | Transient-rare taxa |
| OTU 2241 | 0.00 | 0.00 | 0.00 | 0.00 | 0.02 | 0.00 | 0.00 | 0.00 | 0.00 | 0.00 | 0.00 | 0.00 | Firmicutes | Erysipelotrichia | Erysipelotrichales | Erysipelotrichaceae | Transient-rare taxa |
| OTU 2242 | 0.00 | 0.00 | 0.00 | 0.00 | 0.00 | 0.00 | 0.00 | 0.02 | 0.00 | 0.00 | 0.00 | 0.00 | Proteobacteria | Betaproteobacteria | Gallionellales | Gallionellaceae | Transient-rare taxa |
| OTU 2243 | 0.00 | 0.00 | 0.01 | 0.00 | 0.00 | 0.00 | 0.00 | 0.01 | 0.00 | 0.00 | 0.00 | 0.00 | Actinobacteria | Actinobacteria | Actinobacteridae | Actinomycetales | Transient-rare taxa |
| OTU 2244 | 0.00 | 0.00 | 0.00 | 0.00 | 0.01 | 0.00 | 0.00 | 0.00 | 0.00 | 0.00 | 0.00 | 0.01 | Proteobacteria | Deltaproteobacteria | Myxococcales | Cystobacterineae | Transient-rare taxa |
| OTU 2245 | 0.00 | 0.00 | 0.00 | 0.00 | 0.00 | 0.00 | 0.00 | 0.00 | 0.00 | 0.00 | 0.01 | 0.01 | Proteobacteria | Betaproteobacteria | Burkholderiales | Burkholderiales_incertae_sedis | Transient-rare taxa |
| OTU 2246 | 0.00 | 0.00 | 0.00 | 0.00 | 0.00 | 0.00 | 0.00 | 0.00 | 0.00 | 0.00 | 0.02 | 0.00 | Bacteroidetes | Sphingobacteriia | Sphingobacteriales | Chitinophagaceae | Transient-rare taxa |
| OTU 2247 | 0.00 | 0.00 | 0.00 | 0.00 | 0.00 | 0.00 | 0.00 | 0.02 | 0.00 | 0.00 | 0.00 | 0.00 | Proteobacteria | Gammaproteobacteria | Chromatiales | Chromatiaceae | Transient-rare taxa |
| OTU 2248 | 0.00 | 0.00 | 0.00 | 0.00 | 0.00 | 0.00 | 0.00 | 0.00 | 0.00 | 0.00 | 0.02 | 0.00 | Proteobacteria | Betaproteobacteria | Burkholderiales | Comamonadaceae | Transient-rare taxa |
| OTU 2249 | 0.00 | 0.00 | 0.00 | 0.00 | 0.00 | 0.00 | 0.00 | 0.00 | 0.00 | 0.00 | 0.02 | 0.00 | Proteobacteria | Gammaproteobacteria | Xanthomonadales | Xanthomonadaceae | Transient-rare taxa |
| OTU 2250 | 0.00 | 0.00 | 0.00 | 0.00 | 0.02 | 0.00 | 0.00 | 0.00 | 0.00 | 0.00 | 0.00 | 0.00 | Firmicutes | Clostridia | Clostridiales | Ruminococcaceae | Transient-rare taxa |
| OTU 2251 | 0.00 | 0.00 | 0.00 | 0.00 | 0.00 | 0.00 | 0.00 | 0.00 | 0.00 | 0.00 | 0.02 | 0.00 | Proteobacteria | Alphaproteobacteria | Rhizobiales | Rhizobiales_incertae_sedis | Transient-rare taxa |
| OTU 2252 | 0.00 | 0.00 | 0.00 | 0.00 | 0.01 | 0.01 | 0.00 | 0.00 | 0.00 | 0.00 | 0.00 | 0.00 | Proteobacteria | Deltaproteobacteria | Myxococcales | Cystobacterineae | Transient-rare taxa |
| OTU 2253 | 0.00 | 0.00 | 0.00 | 0.00 | 0.00 | 0.00 | 0.00 | 0.02 | 0.00 | 0.00 | 0.00 | 0.00 | Proteobacteria | Deltaproteobacteria | Desulfobacterales | Desulfobulbaceae | Transient-rare taxa |
| OTU 2254 | 0.00 | 0.00 | 0.00 | 0.00 | 0.02 | 0.00 | 0.00 | 0.00 | 0.00 | 0.00 | 0.00 | 0.00 | Bacteroidetes | Flavobacteriia | Flavobacteriales | Flavobacteriaceae | Transient-rare taxa |
| OTU 2255 | 0.00 | 0.00 | 0.00 | 0.00 | 0.02 | 0.00 | 0.00 | 0.00 | 0.00 | 0.00 | 0.00 | 0.00 | Firmicutes | Erysipelotrichia | Erysipelotrichales | Erysipelotrichaceae | Transient-rare taxa |
| OTU 2256 | 0.00 | 0.00 | 0.00 | 0.00 | 0.00 | 0.00 | 0.01 | 0.01 | 0.00 | 0.00 | 0.00 | 0.00 | Proteobacteria | Betaproteobacteria | Burkholderiales | Burkholderiales_incertae_sedis | Transient-rare taxa |
| OTU 2257 | 0.00 | 0.00 | 0.00 | 0.00 | 0.02 | 0.00 | 0.00 | 0.00 | 0.00 | 0.00 | 0.00 | 0.00 | Proteobacteria | Alphaproteobacteria | Alphaproteobacteria_incertae_sedis | Geminicoccus | Transient-rare taxa |
| OTU 2258 | 0.00 | 0.00 | 0.00 | 0.00 | 0.01 | 0.00 | 0.01 | 0.00 | 0.00 | 0.00 | 0.00 | 0.00 | Proteobacteria | Deltaproteobacteria | Desulfuromonadales | Desulfuromonadaceae | Transient-rare taxa |
| OTU 2259 | 0.00 | 0.00 | 0.00 | 0.00 | 0.00 | 0.00 | 0.00 | 0.02 | 0.00 | 0.00 | 0.00 | 0.00 | Proteobacteria | Deltaproteobacteria | Myxococcales | Nannocystineae | Transient-rare taxa |
| OTU 2260 | 0.00 | 0.00 | 0.00 | 0.00 | 0.00 | 0.00 | 0.01 | 0.00 | 0.00 | 0.00 | 0.01 | 0.00 | Proteobacteria | Alphaproteobacteria | Rhodobacterales | Rhodobacteraceae | Transient-rare taxa |
| OTU 2261 | 0.00 | 0.00 | 0.00 | 0.00 | 0.00 | 0.00 | 0.00 | 0.00 | 0.00 | 0.00 | 0.02 | 0.00 | Proteobacteria | Betaproteobacteria | Burkholderiales | Burkholderiales_incertae_sedis | Transient-rare taxa |
| OTU 2262 | 0.00 | 0.00 | 0.00 | 0.00 | 0.00 | 0.00 | 0.00 | 0.00 | 0.00 | 0.00 | 0.02 | 0.00 | Proteobacteria | Alphaproteobacteria | Rhodobacterales | Rhodobacteraceae | Transient-rare taxa |
| OTU 2263 | 0.00 | 0.00 | 0.00 | 0.00 | 0.00 | 0.00 | 0.00 | 0.00 | 0.00 | 0.00 | 0.02 | 0.00 | Proteobacteria | Deltaproteobacteria | Myxococcales | Sorangiineae | Transient-rare taxa |
| OTU 2264 | 0.00 | 0.00 | 0.00 | 0.00 | 0.00 | 0.00 | 0.00 | 0.00 | 0.00 | 0.00 | 0.02 | 0.00 | Proteobacteria | Deltaproteobacteria | Myxococcales | Sorangiineae | Transient-rare taxa |

| | | | | | | | | | | | | | | | | | |
|---|---|---|---|---|---|---|---|---|---|---|---|---|---|---|---|---|---|
| OTU 2265 | 0.00 | 0.00 | 0.00 | 0.00 | 0.00 | 0.00 | 0.00 | 0.00 | 0.00 | 0.00 | 0.02 | 0.00 | *Bacteroidetes* | *Cytophagia* | *Cytophagales* | *Flammeovirgaceae* | Transient-rare taxa |
| OTU 2266 | 0.00 | 0.00 | 0.00 | 0.00 | 0.00 | 0.00 | 0.00 | 0.00 | 0.00 | 0.00 | 0.02 | 0.00 | *Proteobacteria* | *Deltaproteobacteria* | *Myxococcales* | *Sorangiineae* | Transient-rare taxa |
| OTU 2267 | 0.00 | 0.00 | 0.00 | 0.00 | 0.00 | 0.00 | 0.00 | 0.00 | 0.00 | 0.00 | 0.02 | 0.00 | *Proteobacteria* | *Betaproteobacteria* | *Burkholderiales* | *Comamonadaceae* | Transient-rare taxa |
| OTU 2268 | 0.00 | 0.00 | 0.00 | 0.00 | 0.00 | 0.00 | 0.00 | 0.00 | 0.01 | 0.00 | 0.01 | 0.00 | *Proteobacteria* | *Alphaproteobacteria* | *Rhizobiales* | *Hyphomicrobiaceae* | Transient-rare taxa |
| OTU 2269 | 0.00 | 0.00 | 0.00 | 0.00 | 0.01 | 0.00 | 0.00 | 0.00 | 0.00 | 0.00 | 0.01 | 0.00 | *Proteobacteria* | *Alphaproteobacteria* | *Alphaproteobacteria_incertae_sedis* | *Rhizomicrobium* | Transient-rare taxa |
| OTU 2270 | 0.00 | 0.00 | 0.00 | 0.00 | 0.00 | 0.00 | 0.00 | 0.00 | 0.00 | 0.00 | 0.02 | 0.00 | *Proteobacteria* | *Betaproteobacteria* | *Rhodocyclales* | *Rhodocyclaceae* | Transient-rare taxa |
| OTU 2271 | 0.00 | 0.00 | 0.00 | 0.00 | 0.00 | 0.00 | 0.00 | 0.01 | 0.01 | 0.00 | 0.00 | 0.00 | *Proteobacteria* | *Betaproteobacteria* | *Rhodocyclales* | *Rhodocyclaceae* | Transient-rare taxa |
| OTU 2272 | 0.00 | 0.00 | 0.00 | 0.00 | 0.00 | 0.00 | 0.00 | 0.00 | 0.00 | 0.00 | 0.02 | 0.00 | *Proteobacteria* | *Betaproteobacteria* | *Rhodocyclales* | *Rhodocyclaceae* | Transient-rare taxa |
| OTU 2273 | 0.00 | 0.00 | 0.00 | 0.00 | 0.01 | 0.00 | 0.00 | 0.00 | 0.00 | 0.00 | 0.01 | 0.00 | *Bacteroidetes* | *Flavobacteriia* | *Flavobacteriales* | *Flavobacteriaceae* | Transient-rare taxa |
| OTU 2274 | 0.00 | 0.00 | 0.01 | 0.00 | 0.00 | 0.00 | 0.00 | 0.01 | 0.00 | 0.00 | 0.00 | 0.00 | *Proteobacteria* | *Alphaproteobacteria* | *Rhodospirillales* | *Acetobacteraceae* | Transient-rare taxa |
| OTU 2275 | 0.00 | 0.00 | 0.00 | 0.00 | 0.00 | 0.00 | 0.00 | 0.00 | 0.00 | 0.00 | 0.02 | 0.00 | *Proteobacteria* | *Deltaproteobacteria* | *Myxococcales* | *Nannocystineae* | Transient-rare taxa |
| OTU 2276 | 0.00 | 0.00 | 0.00 | 0.00 | 0.00 | 0.00 | 0.00 | 0.01 | 0.00 | 0.00 | 0.01 | 0.00 | *Proteobacteria* | *Betaproteobacteria* | *Ferrovales* | *Ferrovaceae* | Transient-rare taxa |
| OTU 2277 | 0.00 | 0.00 | 0.00 | 0.00 | 0.00 | 0.00 | 0.00 | 0.00 | 0.00 | 0.02 | 0.00 | 0.00 | *Proteobacteria* | *Betaproteobacteria* | *Rhodocyclales* | *Rhodocyclaceae* | Transient-rare taxa |
| OTU 2278 | 0.02 | 0.00 | 0.00 | 0.00 | 0.00 | 0.00 | 0.00 | 0.00 | 0.00 | 0.00 | 0.00 | 0.00 | *Proteobacteria* | *Deltaproteobacteria* | *Myxococcales* | *Cystobacterineae* | Transient-rare taxa |
| OTU 2279 | 0.00 | 0.00 | 0.00 | 0.00 | 0.00 | 0.00 | 0.01 | 0.01 | 0.00 | 0.00 | 0.00 | 0.00 | *Planctomycetes* | *Planctomycetia* | *Planctomycetales* | *Planctomycetaceae* | Transient-rare taxa |
| OTU 2280 | 0.00 | 0.00 | 0.00 | 0.00 | 0.00 | 0.00 | 0.00 | 0.02 | 0.00 | 0.00 | 0.00 | 0.00 | *Armatimonadetes* | *Armatimonadetes_gp2* | | | Transient-rare taxa |
| OTU 2281 | 0.00 | 0.00 | 0.00 | 0.00 | 0.01 | 0.00 | 0.00 | 0.00 | 0.00 | 0.01 | 0.00 | 0.00 | *Firmicutes* | *Clostridia* | *Clostridiales* | *Lachnospiraceae* | Transient-rare taxa |
| OTU 2282 | 0.00 | 0.00 | 0.00 | 0.00 | 0.00 | 0.00 | 0.00 | 0.00 | 0.00 | 0.00 | 0.01 | 0.01 | *Proteobacteria* | *Alphaproteobacteria* | *Rhodobacterales* | *Rhodobacteraceae* | Transient-rare taxa |
| OTU 2283 | 0.01 | 0.00 | 0.00 | 0.00 | 0.00 | 0.00 | 0.00 | 0.00 | 0.00 | 0.00 | 0.01 | 0.00 | *Proteobacteria* | *Alphaproteobacteria* | *Sphingomonadales* | *Sphingomonadaceae* | Transient-rare taxa |
| OTU 2284 | 0.00 | 0.00 | 0.00 | 0.00 | 0.00 | 0.00 | 0.00 | 0.00 | 0.00 | 0.00 | 0.02 | 0.00 | *Planctomycetes* | *Planctomycetia* | *Planctomycetales* | *Planctomycetaceae* | Transient-rare taxa |
| OTU 2285 | 0.00 | 0.00 | 0.00 | 0.00 | 0.00 | 0.00 | 0.00 | 0.00 | 0.01 | 0.00 | 0.01 | 0.00 | *Proteobacteria* | *Alphaproteobacteria* | *Rhodobacterales* | *Rhodobacteraceae* | Transient-rare taxa |
| OTU 2286 | 0.00 | 0.00 | 0.00 | 0.00 | 0.00 | 0.00 | 0.00 | 0.00 | 0.00 | 0.00 | 0.02 | 0.00 | *Proteobacteria* | *Deltaproteobacteria* | *Myxococcales* | *Sorangiineae* | Transient-rare taxa |
| OTU 2287 | 0.00 | 0.00 | 0.00 | 0.00 | 0.00 | 0.00 | 0.00 | 0.00 | 0.00 | 0.00 | 0.02 | 0.00 | *Proteobacteria* | *Deltaproteobacteria* | *Myxococcales* | *Nannocystineae* | Transient-rare taxa |
| OTU 2288 | 0.00 | 0.00 | 0.00 | 0.00 | 0.00 | 0.00 | 0.00 | 0.00 | 0.00 | 0.00 | 0.02 | 0.00 | *Proteobacteria* | *Betaproteobacteria* | *Rhodocyclales* | *Rhodocyclaceae* | Transient-rare taxa |
| OTU 2289 | 0.00 | 0.01 | 0.00 | 0.00 | 0.00 | 0.00 | 0.00 | 0.00 | 0.00 | 0.00 | 0.01 | 0.00 | *Bacteroidetes* | *Sphingobacteriia* | *Sphingobacteriales* | *Chitinophagaceae* | Transient-rare taxa |
| OTU 2290 | 0.00 | 0.01 | 0.00 | 0.00 | 0.00 | 0.00 | 0.00 | 0.00 | 0.00 | 0.00 | 0.01 | 0.00 | *Proteobacteria* | *Betaproteobacteria* | *Burkholderiales* | *Comamonadaceae* | Transient-rare taxa |
| OTU 2291 | 0.00 | 0.00 | 0.00 | 0.00 | 0.00 | 0.00 | 0.00 | 0.00 | 0.00 | 0.02 | 0.00 | 0.00 | *Firmicutes* | *Clostridia* | *Clostridiales* | *Ruminococcaceae* | Transient-rare taxa |
| OTU 2292 | 0.01 | 0.00 | 0.00 | 0.00 | 0.00 | 0.00 | 0.00 | 0.00 | 0.00 | 0.00 | 0.00 | 0.01 | *Bacteroidetes* | *Sphingobacteriia* | *Sphingobacteriales* | *Chitinophagaceae* | Transient-rare taxa |
| OTU 2293 | 0.02 | 0.00 | 0.00 | 0.00 | 0.00 | 0.00 | 0.00 | 0.00 | 0.00 | 0.00 | 0.00 | 0.00 | *Proteobacteria* | *Betaproteobacteria* | *Burkholderiales* | *Burkholderiaceae* | Transient-rare taxa |
| OTU 2294 | 0.00 | 0.00 | 0.00 | 0.00 | 0.00 | 0.00 | 0.00 | 0.00 | 0.01 | 0.01 | 0.00 | 0.00 | *Proteobacteria* | *Alphaproteobacteria* | *Sphingomonadales* | *Sphingomonadaceae* | Transient-rare taxa |
| OTU 2295 | 0.00 | 0.00 | 0.00 | 0.00 | 0.00 | 0.00 | 0.00 | 0.00 | 0.00 | 0.00 | 0.02 | 0.00 | *Proteobacteria* | *Deltaproteobacteria* | *Bdellovibrionales* | *Bacteriovoracaceae* | Transient-rare taxa |
| OTU 2296 | 0.00 | 0.00 | 0.00 | 0.00 | 0.00 | 0.00 | 0.01 | 0.00 | 0.00 | 0.00 | 0.01 | 0.00 | *Proteobacteria* | *Betaproteobacteria* | *Rhodocyclales* | *Rhodocyclaceae* | Transient-rare taxa |
| OTU 2297 | 0.01 | 0.00 | 0.00 | 0.00 | 0.00 | 0.00 | 0.00 | 0.00 | 0.00 | 0.00 | 0.01 | 0.00 | *Proteobacteria* | *Deltaproteobacteria* | *Myxococcales* | *Cystobacterineae* | Transient-rare taxa |
| OTU 2298 | 0.00 | 0.00 | 0.00 | 0.00 | 0.00 | 0.00 | 0.00 | 0.00 | 0.00 | 0.00 | 0.02 | 0.00 | *Proteobacteria* | *Alphaproteobacteria* | *Rhodobacterales* | *Rhodobacteraceae* | Transient-rare taxa |
| OTU 2299 | 0.00 | 0.01 | 0.00 | 0.00 | 0.00 | 0.00 | 0.00 | 0.00 | 0.00 | 0.00 | 0.01 | 0.00 | *Proteobacteria* | *Betaproteobacteria* | *Rhodocyclales* | *Rhodocyclaceae* | Transient-rare taxa |
| OTU 2300 | 0.00 | 0.00 | 0.00 | 0.00 | 0.00 | 0.00 | 0.00 | 0.00 | 0.00 | 0.00 | 0.01 | 0.01 | *SR1* | *SR1_genera_incertae_sedis* | | | Transient-rare taxa |
| OTU 2301 | 0.00 | 0.00 | 0.00 | 0.00 | 0.00 | 0.00 | 0.00 | 0.00 | 0.00 | 0.01 | 0.01 | 0.00 | *Proteobacteria* | *Deltaproteobacteria* | *Myxococcales* | *Nannocystineae* | Transient-rare taxa |
| OTU 2302 | 0.00 | 0.00 | 0.00 | 0.00 | 0.00 | 0.00 | 0.00 | 0.00 | 0.00 | 0.00 | 0.02 | 0.00 | *Proteobacteria* | *Alphaproteobacteria* | *Rhizobiales* | *Rhodobiaceae* | Transient-rare taxa |
| OTU 2303 | 0.00 | 0.00 | 0.00 | 0.00 | 0.00 | 0.00 | 0.00 | 0.00 | 0.00 | 0.00 | 0.02 | 0.00 | *Proteobacteria* | *Deltaproteobacteria* | *Myxococcales* | *Nannocystineae* | Transient-rare taxa |
| OTU 2304 | 0.00 | 0.00 | 0.00 | 0.00 | 0.02 | 0.00 | 0.00 | 0.00 | 0.00 | 0.00 | 0.00 | 0.00 | *Bacteroidetes* | *Flavobacteriia* | *Flavobacteriales* | *Flavobacteriaceae* | Transient-rare taxa |
| OTU 2305 | 0.00 | 0.00 | 0.00 | 0.00 | 0.00 | 0.00 | 0.00 | 0.00 | 0.00 | 0.00 | 0.02 | 0.00 | *Bacteroidetes* | *Flavobacteriia* | *Flavobacteriales* | *Flavobacteriaceae* | Transient-rare taxa |
| OTU 2306 | 0.00 | 0.00 | 0.00 | 0.00 | 0.00 | 0.00 | 0.00 | 0.00 | 0.01 | 0.00 | 0.00 | 0.01 | *Gemmatimonadetes* | *Gemmatimonadetes* | *Gemmatimonadales* | *Gemmatimonadaceae* | Transient-rare taxa |
| OTU 2307 | 0.01 | 0.01 | 0.00 | 0.00 | 0.00 | 0.00 | 0.00 | 0.00 | 0.00 | 0.00 | 0.00 | 0.00 | *Proteobacteria* | *Gammaproteobacteria* | *Chromatiales* | *Ectothiorhodospiraceae* | Transient-rare taxa |
| OTU 2308 | 0.01 | 0.00 | 0.00 | 0.00 | 0.00 | 0.01 | 0.00 | 0.00 | 0.00 | 0.00 | 0.00 | 0.00 | *Proteobacteria* | *Betaproteobacteria* | *Rhodocyclales* | *Rhodocyclaceae* | Transient-rare taxa |
| OTU 2309 | 0.02 | 0.00 | 0.00 | 0.00 | 0.00 | 0.00 | 0.00 | 0.00 | 0.00 | 0.00 | 0.00 | 0.00 | *Bacteroidetes* | *Cytophagia* | *Cytophagales* | *Cytophagaceae* | Transient-rare taxa |
| OTU 2310 | 0.02 | 0.00 | 0.00 | 0.00 | 0.00 | 0.00 | 0.00 | 0.00 | 0.00 | 0.00 | 0.00 | 0.00 | *Bacteroidetes* | *Sphingobacteriia* | *Sphingobacteriales* | *Saprospiraceae* | Transient-rare taxa |
| OTU 2311 | 0.02 | 0.00 | 0.00 | 0.00 | 0.00 | 0.00 | 0.00 | 0.00 | 0.00 | 0.00 | 0.00 | 0.00 | *Proteobacteria* | *Betaproteobacteria* | *Nitrosomonadales* | *Nitrosomonadaceae* | Transient-rare taxa |
| OTU 2312 | 0.02 | 0.00 | 0.00 | 0.00 | 0.00 | 0.00 | 0.00 | 0.00 | 0.00 | 0.00 | 0.00 | 0.00 | *Proteobacteria* | *Gammaproteobacteria* | *Pseudomonadales* | *Moraxellaceae* | Transient-rare taxa |
| OTU 2313 | 0.00 | 0.00 | 0.00 | 0.00 | 0.02 | 0.00 | 0.00 | 0.00 | 0.00 | 0.00 | 0.00 | 0.00 | *Proteobacteria* | *Alphaproteobacteria* | *Kiloniellales* | *Kiloniellaceae* | Transient-rare taxa |
| OTU 2314 | 0.00 | 0.00 | 0.00 | 0.00 | 0.00 | 0.00 | 0.00 | 0.00 | 0.00 | 0.01 | 0.01 | 0.00 | *Proteobacteria* | *Betaproteobacteria* | *Rhodocyclales* | *Rhodocyclaceae* | Transient-rare taxa |
| OTU 2315 | 0.00 | 0.00 | 0.00 | 0.00 | 0.00 | 0.00 | 0.00 | 0.00 | 0.01 | 0.00 | 0.00 | 0.01 | *Proteobacteria* | *Deltaproteobacteria* | *Bdellovibrionales* | *Bacteriovoracaceae* | Transient-rare taxa |
| OTU 2316 | 0.00 | 0.00 | 0.00 | 0.00 | 0.00 | 0.00 | 0.00 | 0.00 | 0.00 | 0.00 | 0.00 | 0.02 | *Proteobacteria* | *Betaproteobacteria* | *Burkholderiales* | *Comamonadaceae* | Transient-rare taxa |
| OTU 2317 | 0.00 | 0.00 | 0.00 | 0.00 | 0.00 | 0.00 | 0.00 | 0.00 | 0.02 | 0.00 | 0.00 | 0.00 | *Proteobacteria* | *Betaproteobacteria* | *Nitrosomonadales* | *Nitrosomonadaceae* | Transient-rare taxa |
| OTU 2318 | 0.00 | 0.00 | 0.00 | 0.00 | 0.00 | 0.00 | 0.00 | 0.02 | 0.00 | 0.00 | 0.00 | 0.00 | *Proteobacteria* | *Betaproteobacteria* | *Rhodocyclales* | *Rhodocyclaceae* | Transient-rare taxa |
| OTU 2319 | 0.00 | 0.00 | 0.00 | 0.00 | 0.00 | 0.00 | 0.00 | 0.00 | 0.00 | 0.00 | 0.02 | 0.00 | *Bacteroidetes* | *Flavobacteriia* | *Flavobacteriales* | *Flavobacteriaceae* | Transient-rare taxa |
| OTU 2320 | 0.00 | 0.00 | 0.00 | 0.00 | 0.00 | 0.00 | 0.00 | 0.00 | 0.00 | 0.00 | 0.02 | 0.00 | *Proteobacteria* | *Betaproteobacteria* | *Rhodocyclales* | *Rhodocyclaceae* | Transient-rare taxa |
| OTU 2321 | 0.00 | 0.00 | 0.00 | 0.00 | 0.00 | 0.00 | 0.00 | 0.00 | 0.00 | 0.00 | 0.02 | 0.00 | *Proteobacteria* | *Gammaproteobacteria* | *Alteromonadales* | *Celerinatantimonadaceae* | Transient-rare taxa |
| OTU 2322 | 0.00 | 0.00 | 0.00 | 0.00 | 0.01 | 0.00 | 0.00 | 0.00 | 0.00 | 0.00 | 0.01 | 0.00 | *Proteobacteria* | *Betaproteobacteria* | *Burkholderiales* | *Comamonadaceae* | Transient-rare taxa |
| OTU 2323 | 0.00 | 0.00 | 0.00 | 0.00 | 0.00 | 0.00 | 0.00 | 0.00 | 0.00 | 0.00 | 0.02 | 0.00 | *Proteobacteria* | *Alphaproteobacteria* | *Sphingomonadales* | *Sphingomonadaceae* | Transient-rare taxa |
| OTU 2324 | 0.00 | 0.00 | 0.00 | 0.00 | 0.00 | 0.00 | 0.00 | 0.00 | 0.01 | 0.01 | 0.00 | 0.00 | *Proteobacteria* | *Gammaproteobacteria* | *Xanthomonadales* | *Xanthomonadaceae* | Transient-rare taxa |
| OTU 2325 | 0.00 | 0.00 | 0.00 | 0.00 | 0.00 | 0.00 | 0.00 | 0.00 | 0.02 | 0.00 | 0.00 | 0.00 | *Proteobacteria* | *Deltaproteobacteria* | *Myxococcales* | *Sorangiineae* | Transient-rare taxa |
| OTU 2326 | 0.00 | 0.00 | 0.00 | 0.00 | 0.00 | 0.01 | 0.00 | 0.00 | 0.01 | 0.00 | 0.00 | 0.00 | *Proteobacteria* | *Gammaproteobacteria* | *Alteromonadales* | *Alteromonadaceae* | Transient-rare taxa |
| OTU 2327 | 0.00 | 0.00 | 0.00 | 0.00 | 0.00 | 0.00 | 0.00 | 0.00 | 0.00 | 0.02 | 0.00 | 0.00 | *Actinobacteria* | *Actinobacteria* | *Rubrobacteridae* | *Solirubrobacterales* | Transient-rare taxa |
| OTU 2328 | 0.00 | 0.00 | 0.01 | 0.00 | 0.00 | 0.00 | 0.00 | 0.00 | 0.01 | 0.00 | 0.00 | 0.00 | *Proteobacteria* | *Alphaproteobacteria* | *Rhizobiales* | *Hyphomicrobiaceae* | Transient-rare taxa |
| OTU 2329 | 0.00 | 0.00 | 0.00 | 0.00 | 0.00 | 0.00 | 0.00 | 0.01 | 0.01 | 0.00 | 0.00 | 0.00 | *candidate division WPS-1* | *WPS-1_genera_incertae_sedis* | | | Transient-rare taxa |
| OTU 2330 | 0.00 | 0.00 | 0.00 | 0.00 | 0.00 | 0.00 | 0.00 | 0.00 | 0.01 | 0.01 | 0.00 | 0.00 | *Proteobacteria* | *Deltaproteobacteria* | *Desulfobacterales* | *Desulfobacteraceae* | Transient-rare taxa |
| OTU 2331 | 0.00 | 0.02 | 0.00 | 0.00 | 0.00 | 0.00 | 0.00 | 0.00 | 0.00 | 0.00 | 0.00 | 0.00 | *Proteobacteria* | *Betaproteobacteria* | *Burkholderiales* | *Comamonadaceae* | Transient-rare taxa |
| OTU 2332 | 0.00 | 0.00 | 0.00 | 0.01 | 0.00 | 0.00 | 0.01 | 0.00 | 0.00 | 0.00 | 0.00 | 0.00 | *Bacteroidetes* | *Sphingobacteriia* | *Sphingobacteriales* | *Cyclobacteriaceae* | Transient-rare taxa |
| OTU 2333 | 0.00 | 0.00 | 0.00 | 0.02 | 0.00 | 0.00 | 0.00 | 0.00 | 0.00 | 0.00 | 0.00 | 0.00 | *Proteobacteria* | *Alphaproteobacteria* | *Rhodospirillales* | *Rhodospirillaceae* | Transient-rare taxa |
| OTU 2334 | 0.00 | 0.00 | 0.00 | 0.02 | 0.00 | 0.00 | 0.00 | 0.00 | 0.00 | 0.00 | 0.00 | 0.00 | *Proteobacteria* | *Gammaproteobacteria* | *Pseudomonadales* | *Moraxellaceae* | Transient-rare taxa |
| OTU 2335 | 0.00 | 0.01 | 0.00 | 0.00 | 0.00 | 0.00 | 0.01 | 0.00 | 0.00 | 0.00 | 0.00 | 0.00 | *Proteobacteria* | *Betaproteobacteria* | *Rhodocyclales* | *Rhodocyclaceae* | Transient-rare taxa |
| OTU 2336 | 0.00 | 0.00 | 0.00 | 0.00 | 0.00 | 0.00 | 0.00 | 0.00 | 0.00 | 0.02 | 0.00 | 0.00 | *Bacteroidetes* | *Sphingobacteriia* | *Sphingobacteriales* | *Saprospiraceae* | Transient-rare taxa |
| OTU 2337 | 0.00 | 0.00 | 0.01 | 0.01 | 0.00 | 0.00 | 0.00 | 0.00 | 0.00 | 0.00 | 0.00 | 0.00 | *Proteobacteria* | *Alphaproteobacteria* | *Rhodobacterales* | *Rhodobacteraceae* | Transient-rare taxa |
| OTU 2338 | 0.00 | 0.00 | 0.00 | 0.02 | 0.00 | 0.00 | 0.00 | 0.00 | 0.00 | 0.00 | 0.00 | 0.00 | *Proteobacteria* | *Alphaproteobacteria* | *Rhodobacterales* | *Rhodobacteraceae* | Transient-rare taxa |
| OTU 2339 | 0.00 | 0.00 | 0.00 | 0.01 | 0.00 | 0.01 | 0.00 | 0.00 | 0.00 | 0.00 | 0.00 | 0.00 | *Chloroflexi* | *Anaerolineae* | *Anaerolineales* | *Anaerolineaceae* | Transient-rare taxa |
| OTU 2340 | 0.00 | 0.00 | 0.00 | 0.01 | 0.00 | 0.00 | 0.00 | 0.00 | 0.01 | 0.00 | 0.00 | 0.00 | *Bacteroidetes* | *Bacteroidia* | *Bacteroidales* | *Porphyromonadaceae* | Transient-rare taxa |
| OTU 2341 | 0.00 | 0.00 | 0.00 | 0.02 | 0.00 | 0.00 | 0.00 | 0.00 | 0.00 | 0.00 | 0.00 | 0.00 | *Proteobacteria* | *Gammaproteobacteria* | *Oceanospirillales* | *Oleiphilaceae* | Transient-rare taxa |
| OTU 2342 | 0.00 | 0.00 | 0.00 | 0.01 | 0.01 | 0.00 | 0.00 | 0.00 | 0.00 | 0.00 | 0.00 | 0.00 | *Firmicutes* | *Clostridia* | *Clostridiales* | *Clostridiaceae 3* | Transient-rare taxa |
| OTU 2343 | 0.00 | 0.00 | 0.00 | 0.01 | 0.01 | 0.00 | 0.00 | 0.00 | 0.00 | 0.00 | 0.00 | 0.00 | *Bacteroidetes* | *Flavobacteriia* | *Flavobacteriales* | *Cryomorphaceae* | Transient-rare taxa |
| OTU 2344 | 0.00 | 0.00 | 0.00 | 0.00 | 0.00 | 0.00 | 0.00 | 0.00 | 0.00 | 0.01 | 0.00 | 0.01 | *Proteobacteria* | *Betaproteobacteria* | *Rhodocyclales* | *Rhodocyclaceae* | Transient-rare taxa |
| OTU 2345 | 0.00 | 0.00 | 0.00 | 0.01 | 0.00 | 0.00 | 0.01 | 0.00 | 0.00 | 0.00 | 0.00 | 0.00 | *Proteobacteria* | *Gammaproteobacteria* | *Alteromonadales* | *Alteromonadaceae* | Transient-rare taxa |
| OTU 2346 | 0.00 | 0.00 | 0.00 | 0.00 | 0.00 | 0.00 | 0.00 | 0.00 | 0.00 | 0.02 | 0.00 | 0.00 | *Proteobacteria* | *Gammaproteobacteria* | *Chromatiales* | *Chromatiaceae* | Transient-rare taxa |
| OTU 2347 | 0.00 | 0.00 | 0.00 | 0.01 | 0.00 | 0.00 | 0.01 | 0.00 | 0.00 | 0.00 | 0.00 | 0.00 | *Proteobacteria* | *Betaproteobacteria* | *Rhodocyclales* | *Rhodocyclaceae* | Transient-rare taxa |
| OTU 2348 | 0.01 | 0.01 | 0.00 | 0.00 | 0.00 | 0.00 | 0.00 | 0.00 | 0.00 | 0.00 | 0.00 | 0.00 | *Actinobacteria* | *Actinobacteria* | *Acidimicrobidae* | *Acidimicrobiales* | Transient-rare taxa |
| OTU 2349 | 0.00 | 0.00 | 0.00 | 0.02 | 0.00 | 0.00 | 0.00 | 0.00 | 0.00 | 0.00 | 0.00 | 0.00 | *Bacteroidetes* | *Flavobacteriia* | *Flavobacteriales* | *Flavobacteriaceae* | Transient-rare taxa |
| OTU 2350 | 0.01 | 0.00 | 0.00 | 0.00 | 0.00 | 0.00 | 0.00 | 0.00 | 0.00 | 0.01 | 0.00 | 0.00 | *Proteobacteria* | *Gammaproteobacteria* | *Chromatiales* | *Ectothiorhodospiraceae* | Transient-rare taxa |
| OTU 2351 | 0.00 | 0.01 | 0.00 | 0.00 | 0.00 | 0.00 | 0.00 | 0.00 | 0.01 | 0.00 | 0.00 | 0.00 | *Proteobacteria* | *Gammaproteobacteria* | *Xanthomonadales* | *Xanthomonadaceae* | Transient-rare taxa |
| OTU 2352 | 0.00 | 0.00 | 0.01 | 0.01 | 0.00 | 0.00 | 0.00 | 0.00 | 0.00 | 0.00 | 0.00 | 0.00 | *Proteobacteria* | *Deltaproteobacteria* | *Desulfuromonadales* | *Geobacteraceae* | Transient-rare taxa |
| OTU 2353 | 0.00 | 0.01 | 0.00 | 0.00 | 0.00 | 0.00 | 0.00 | 0.00 | 0.01 | 0.00 | 0.00 | 0.00 | *Proteobacteria* | *Betaproteobacteria* | *Rhodocyclales* | *Rhodocyclaceae* | Transient-rare taxa |
| OTU 2354 | 0.00 | 0.00 | 0.00 | 0.00 | 0.00 | 0.00 | 0.00 | 0.00 | 0.00 | 0.02 | 0.00 | 0.00 | *Bacteroidetes* | *Flavobacteriia* | *Flavobacteriales* | *Flavobacteriaceae* | Transient-rare taxa |
| OTU 2355 | 0.00 | 0.00 | 0.00 | 0.00 | 0.00 | 0.00 | 0.00 | 0.00 | 0.00 | 0.00 | 0.02 | 0.00 | *Proteobacteria* | *Deltaproteobacteria* | *Myxococcales* | *Nannocystineae* | Transient-rare taxa |
| OTU 2356 | 0.00 | 0.01 | 0.00 | 0.00 | 0.00 | 0.00 | 0.01 | 0.00 | 0.00 | 0.00 | 0.00 | 0.00 | *Proteobacteria* | *Betaproteobacteria* | *Burkholderiales* | *Comamonadaceae* | Transient-rare taxa |
| OTU 2357 | 0.00 | 0.00 | 0.00 | 0.00 | 0.00 | 0.00 | 0.02 | 0.00 | 0.00 | 0.00 | 0.00 | 0.00 | *Proteobacteria* | *Betaproteobacteria* | *Rhodocyclales* | *Rhodocyclaceae* | Transient-rare taxa |
| OTU 2358 | 0.00 | 0.00 | 0.00 | 0.00 | 0.00 | 0.00 | 0.00 | 0.00 | 0.00 | 0.01 | 0.00 | 0.01 | *Bacteroidetes* | *Sphingobacteriia* | *Sphingobacteriales* | *Saprospiraceae* | Transient-rare taxa |
| OTU 2359 | 0.00 | 0.02 | 0.00 | 0.00 | 0.00 | 0.00 | 0.00 | 0.00 | 0.00 | 0.00 | 0.00 | 0.00 | *Proteobacteria* | *Betaproteobacteria* | *Rhodocyclales* | *Rhodocyclaceae* | Transient-rare taxa |
| OTU 2360 | 0.00 | 0.00 | 0.01 | 0.00 | 0.00 | 0.00 | 0.00 | 0.01 | 0.00 | 0.00 | 0.00 | 0.00 | *Proteobacteria* | *Deltaproteobacteria* | *Desulfovibrionales* | *Desulfovibrionaceae* | Transient-rare taxa |
| OTU 2361 | 0.00 | 0.00 | 0.00 | 0.00 | 0.00 | 0.00 | 0.00 | 0.02 | 0.00 | 0.00 | 0.00 | 0.00 | *Planctomycetes* | *Planctomycetia* | *Planctomycetales* | *Planctomycetaceae* | Transient-rare taxa |
| OTU 2362 | 0.00 | 0.00 | 0.00 | 0.00 | 0.00 | 0.00 | 0.00 | 0.00 | 0.01 | 0.01 | 0.00 | 0.00 | *Acidobacteria* | *Acidobacteria_Gp4* | *Gp4* | | Transient-rare taxa |
| OTU 2363 | 0.00 | 0.00 | 0.00 | 0.00 | 0.00 | 0.00 | 0.00 | 0.00 | 0.00 | 0.02 | 0.00 | 0.00 | *Proteobacteria* | *Deltaproteobacteria* | *Myxococcales* | *Sorangiineae* | Transient-rare taxa |
| OTU 2364 | 0.00 | 0.00 | 0.00 | 0.00 | 0.00 | 0.00 | 0.00 | 0.00 | 0.00 | 0.00 | 0.02 | 0.00 | *Proteobacteria* | *Betaproteobacteria* | *Rhodocyclales* | *Rhodocyclaceae* | Transient-rare taxa |
| OTU 2365 | 0.00 | 0.00 | 0.00 | 0.00 | 0.00 | 0.00 | 0.01 | 0.00 | 0.00 | 0.00 | 0.01 | 0.00 | *Proteobacteria* | *Betaproteobacteria* | *Rhodocyclales* | *Rhodocyclaceae* | Transient-rare taxa |
| OTU 2366 | 0.00 | 0.00 | 0.00 | 0.00 | 0.00 | 0.00 | 0.02 | 0.00 | 0.00 | 0.00 | 0.00 | 0.00 | *Nitrospirae* | *Nitrospira* | *Nitrospirales* | *Nitrospiraceae* | Transient-rare taxa |
| OTU 2367 | 0.00 | 0.00 | 0.00 | 0.00 | 0.00 | 0.01 | 0.01 | 0.00 | 0.00 | 0.00 | 0.00 | 0.00 | *Actinobacteria* | *Actinobacteria* | *Actinobacteridae* | *Actinomycetales* | Transient-rare taxa |
| OTU 2368 | 0.00 | 0.02 | 0.00 | 0.00 | 0.00 | 0.00 | 0.00 | 0.00 | 0.00 | 0.00 | 0.00 | 0.00 | *Proteobacteria* | *Betaproteobacteria* | *Burkholderiales* | *Comamonadaceae* | Transient-rare taxa |
| OTU 2369 | 0.00 | 0.01 | 0.01 | 0.00 | 0.00 | 0.00 | 0.00 | 0.00 | 0.00 | 0.00 | 0.00 | 0.00 | *Proteobacteria* | *Gammaproteobacteria* | *Oceanospirillales* | *Hahellaceae* | Transient-rare taxa |
| OTU 2370 | 0.00 | 0.00 | 0.00 | 0.00 | 0.00 | 0.00 | 0.00 | 0.00 | 0.00 | 0.02 | 0.00 | 0.00 | *Firmicutes* | *Clostridia* | *Clostridiales* | *Clostridiales_Incertae Sedis XII* | Transient-rare taxa |
| OTU 2371 | 0.00 | 0.01 | 0.00 | 0.00 | 0.00 | 0.00 | 0.00 | 0.00 | 0.00 | 0.00 | 0.00 | 0.01 | *Proteobacteria* | *Deltaproteobacteria* | *Myxococcales* | *Nannocystineae* | Transient-rare taxa |
| OTU 2372 | 0.00 | 0.00 | 0.00 | 0.02 | 0.00 | 0.00 | 0.00 | 0.00 | 0.00 | 0.00 | 0.00 | 0.00 | *Bacteroidetes* | *Flavobacteriia* | *Flavobacteriales* | *Cryomorphaceae* | Transient-rare taxa |
| OTU 2373 | 0.00 | 0.00 | 0.00 | 0.02 | 0.00 | 0.00 | 0.00 | 0.00 | 0.00 | 0.00 | 0.00 | 0.00 | *Bacteroidetes* | *Sphingobacteriia* | *Sphingobacteriales* | *Saprospiraceae* | Transient-rare taxa |
| OTU 2374 | 0.00 | 0.00 | 0.00 | 0.01 | 0.00 | 0.01 | 0.00 | 0.00 | 0.00 | 0.00 | 0.00 | 0.00 | *Proteobacteria* | *Alphaproteobacteria* | *Rhizobiales* | *Beijerinckiaceae* | Transient-rare taxa |
| OTU 2375 | 0.00 | 0.00 | 0.00 | 0.00 | 0.00 | 0.00 | 0.00 | 0.00 | 0.00 | 0.01 | 0.00 | 0.01 | *Proteobacteria* | *Deltaproteobacteria* | *Myxococcales* | *Cystobacterineae* | Transient-rare taxa |
| OTU 2376 | 0.00 | 0.00 | 0.00 | 0.00 | 0.00 | 0.01 | 0.00 | 0.00 | 0.00 | 0.00 | 0.00 | 0.01 | *Chloroflexi* | *Anaerolineae* | *Anaerolineales* | *Anaerolineaceae* | Transient-rare taxa |
| OTU 2377 | 0.00 | 0.01 | 0.00 | 0.00 | 0.00 | 0.00 | 0.00 | 0.00 | 0.00 | 0.00 | 0.00 | 0.01 | *Proteobacteria* | *Betaproteobacteria* | *Rhodocyclales* | *Rhodocyclaceae* | Transient-rare taxa |
| OTU 2378 | 0.00 | 0.00 | 0.00 | 0.00 | 0.00 | 0.00 | 0.00 | 0.01 | 0.00 | 0.00 | 0.00 | 0.01 | *Chloroflexi* | *Anaerolineae* | *Anaerolineales* | *Anaerolineaceae* | Transient-rare taxa |
| OTU 2379 | 0.00 | 0.00 | 0.00 | 0.00 | 0.00 | 0.00 | 0.02 | 0.00 | 0.00 | 0.00 | 0.00 | 0.00 | *Nitrospirae* | *Nitrospira* | *Nitrospirales* | *Nitrospiraceae* | Transient-rare taxa |
| OTU 2380 | 0.00 | 0.00 | 0.00 | 0.00 | 0.00 | 0.00 | 0.02 | 0.00 | 0.00 | 0.00 | 0.00 | 0.00 | *Planctomycetes* | *Planctomycetia* | *Planctomycetales* | *Planctomycetaceae* | Transient-rare taxa |
| OTU 2381 | 0.00 | 0.00 | 0.00 | 0.00 | 0.01 | 0.00 | 0.00 | 0.00 | 0.00 | 0.00 | 0.01 | 0.00 | *Proteobacteria* | *Alphaproteobacteria* | *Rhodobacterales* | *Rhodobacteraceae* | Transient-rare taxa |
| OTU 2382 | 0.00 | 0.00 | 0.00 | 0.00 | 0.00 | 0.00 | 0.00 | 0.00 | 0.00 | 0.00 | 0.02 | 0.00 | *Proteobacteria* | *Gammaproteobacteria* | *Pseudomonadales* | *Moraxellaceae* | Transient-rare taxa |
| OTU 2383 | 0.00 | 0.00 | 0.00 | 0.01 | 0.00 | 0.00 | 0.00 | 0.00 | 0.00 | 0.00 | 0.01 | 0.00 | *Proteobacteria* | *Betaproteobacteria* | *Rhodocyclales* | *Rhodocyclaceae* | Transient-rare taxa |
| OTU 2384 | 0.00 | 0.00 | 0.00 | 0.00 | 0.00 | 0.00 | 0.00 | 0.00 | 0.00 | 0.00 | 0.01 | 0.01 | *Proteobacteria* | *Deltaproteobacteria* | *Myxococcales* | *Sorangiineae* | Transient-rare taxa |
| OTU 2385 | 0.00 | 0.01 | 0.00 | 0.00 | 0.00 | 0.00 | 0.00 | 0.00 | 0.01 | 0.00 | 0.00 | 0.00 | *Proteobacteria* | *Betaproteobacteria* | *Burkholderiales* | *Comamonadaceae* | Transient-rare taxa |
| OTU 2386 | 0.00 | 0.01 | 0.00 | 0.00 | 0.00 | 0.00 | 0.00 | 0.00 | 0.00 | 0.00 | 0.01 | 0.00 | *Proteobacteria* | *Betaproteobacteria* | *Rhodocyclales* | *Rhodocyclaceae* | Transient-rare taxa |
| OTU 2387 | 0.00 | 0.00 | 0.01 | 0.00 | 0.00 | 0.00 | 0.00 | 0.01 | 0.00 | 0.00 | 0.00 | 0.00 | *Chloroflexi* | *Anaerolineae* | *Anaerolineales* | *Anaerolineaceae* | Transient-rare taxa |
| OTU 2388 | 0.00 | 0.00 | 0.00 | 0.00 | 0.00 | 0.00 | 0.00 | 0.01 | 0.00 | 0.01 | 0.00 | 0.00 | *Proteobacteria* | *Deltaproteobacteria* | *Desulfobacterales* | *Desulfobacteraceae* | Transient-rare taxa |
| OTU 2389 | 0.00 | 0.00 | 0.00 | 0.00 | 0.00 | 0.00 | 0.00 | 0.00 | 0.01 | 0.01 | 0.00 | 0.00 | *Proteobacteria* | *Deltaproteobacteria* | *Myxococcales* | *Sorangiineae* | Transient-rare taxa |
| OTU 2390 | 0.00 | 0.00 | 0.00 | 0.01 | 0.01 | 0.00 | 0.00 | 0.00 | 0.00 | 0.00 | 0.00 | 0.00 | *Proteobacteria* | *Epsilonproteobacteria* | *Campylobacterales* | *Helicobacteraceae* | Transient-rare taxa |
| OTU 2391 | 0.00 | 0.00 | 0.00 | 0.00 | 0.00 | 0.00 | 0.00 | 0.00 | 0.00 | 0.00 | 0.02 | 0.00 | *Firmicutes* | *Bacilli* | *Bacillales* | *Bacillaceae 2* | Transient-rare taxa |
| OTU 2392 | 0.00 | 0.00 | 0.00 | 0.00 | 0.00 | 0.00 | 0.00 | 0.00 | 0.00 | 0.00 | 0.01 | 0.01 | *Firmicutes* | *Bacilli* | *Bacillales* | *Bacillaceae 1* | Transient-rare taxa |
| OTU 2393 | 0.00 | 0.00 | 0.00 | 0.00 | 0.00 | 0.00 | 0.00 | 0.00 | 0.00 | 0.00 | 0.02 | 0.00 | *Proteobacteria* | *Alphaproteobacteria* | *Rhodobacterales* | *Rhodobacteraceae* | Transient-rare taxa |
| OTU 2394 | 0.00 | 0.00 | 0.00 | 0.02 | 0.00 | 0.00 | 0.00 | 0.00 | 0.00 | 0.00 | 0.00 | 0.00 | *Proteobacteria* | *Deltaproteobacteria* | *Myxococcales* | *Sorangiineae* | Transient-rare taxa |
| OTU 2395 | 0.01 | 0.01 | 0.00 | 0.00 | 0.00 | 0.00 | 0.00 | 0.00 | 0.00 | 0.00 | 0.00 | 0.00 | *Actinobacteria* | *Actinobacteria* | *Acidimicrobidae* | *Acidimicrobiales* | Transient-rare taxa |
| OTU 2396 | 0.00 | 0.00 | 0.00 | 0.00 | 0.00 | 0.00 | 0.02 | 0.00 | 0.00 | 0.00 | 0.00 | 0.00 | *Proteobacteria* | *Gammaproteobacteria* | *Gammaproteobacteria_incertae_sedis* | *Porticoccus* | Transient-rare taxa |
| OTU 2397 | 0.00 | 0.00 | 0.00 | 0.00 | 0.00 | 0.00 | 0.02 | 0.00 | 0.00 | 0.00 | 0.00 | 0.00 | *Proteobacteria* | *Gammaproteobacteria* | *Candidatus Carsonella* | | Transient-rare taxa |
| OTU 2398 | 0.00 | 0.00 | 0.00 | 0.01 | 0.01 | 0.00 | 0.00 | 0.00 | 0.00 | 0.00 | 0.00 | 0.00 | *Proteobacteria* | *Deltaproteobacteria* | *Desulfuromonadales* | *Geobacteraceae* | Transient-rare taxa |
| OTU 2399 | 0.01 | 0.00 | 0.00 | 0.00 | 0.00 | 0.00 | 0.00 | 0.00 | 0.00 | 0.00 | 0.00 | 0.01 | *Proteobacteria* | *Alphaproteobacteria* | *Caulobacterales* | *Hyphomonadaceae* | Transient-rare taxa |
| OTU 2400 | 0.00 | 0.00 | 0.00 | 0.00 | 0.00 | 0.00 | 0.02 | 0.00 | 0.00 | 0.00 | 0.00 | 0.00 | *Bacteroidetes* | *Flavobacteriia* | *Flavobacteriales* | *Flavobacteriaceae* | Transient-rare taxa |
| OTU 2401 | 0.00 | 0.00 | 0.00 | 0.00 | 0.00 | 0.00 | 0.00 | 0.00 | 0.00 | 0.00 | 0.00 | 0.02 | *Proteobacteria* | *Alphaproteobacteria* | *Rhodospirillales* | *Rhodospirillaceae* | Transient-rare taxa |
| OTU 2402 | 0.00 | 0.01 | 0.00 | 0.00 | 0.00 | 0.00 | 0.01 | 0.00 | 0.00 | 0.00 | 0.00 | 0.00 | *Proteobacteria* | *Betaproteobacteria* | *Rhodocyclales* | *Rhodocyclaceae* | Transient-rare taxa |
| OTU 2403 | 0.00 | 0.00 | 0.00 | 0.00 | 0.00 | 0.02 | 0.00 | 0.00 | 0.00 | 0.00 | 0.00 | 0.00 | *Proteobacteria* | *Gammaproteobacteria* | *Oceanospirillales* | *Hahellaceae* | Transient-rare taxa |
| OTU 2404 | 0.00 | 0.00 | 0.00 | 0.00 | 0.02 | 0.00 | 0.00 | 0.00 | 0.00 | 0.00 | 0.00 | 0.00 | *Bacteroidetes* | *Flavobacteriia* | *Flavobacteriales* | *Cryomorphaceae* | Transient-rare taxa |
| OTU 2405 | 0.00 | 0.00 | 0.00 | 0.00 | 0.00 | 0.00 | 0.02 | 0.00 | 0.00 | 0.00 | 0.00 | 0.00 | *Bacteroidetes* | *Cytophagia* | *Cytophagales* | *Flammeovirgaceae* | Transient-rare taxa |
| OTU 2406 | 0.00 | 0.00 | 0.00 | 0.00 | 0.00 | 0.00 | 0.01 | 0.00 | 0.00 | 0.00 | 0.00 | 0.01 | *Gemmatimonadetes* | *Gemmatimonadetes* | *Gemmatimonadales* | *Gemmatimonadaceae* | |

| | | | | | | | | | | | | | | | | |
|---|---|---|---|---|---|---|---|---|---|---|---|---|---|---|---|---|
| OTU 2407 | 0.00 | 0.01 | 0.00 | 0.00 | 0.00 | 0.00 | 0.00 | 0.00 | 0.00 | 0.00 | 0.00 | 0.01 | *Proteobacteria* | *Betaproteobacteria* | *Rhodocyclales* | *Rhodocyclaceae* | Transient-rare taxa |
| OTU 2408 | 0.01 | 0.00 | 0.00 | 0.00 | 0.00 | 0.00 | 0.00 | 0.00 | 0.00 | 0.00 | 0.00 | 0.01 | *Actinobacteria* | *Actinobacteria* | *Acidimicrobidae* | *Acidimicrobiales* | Transient-rare taxa |
| OTU 2409 | 0.00 | 0.00 | 0.00 | 0.00 | 0.00 | 0.00 | 0.00 | 0.00 | 0.00 | 0.00 | 0.00 | 0.02 | *Planctomycetes* | *Planctomycetia* | *Planctomycetales* | *Planctomycetaceae* | Transient-rare taxa |
| OTU 2410 | 0.00 | 0.00 | 0.00 | 0.01 | 0.01 | 0.00 | 0.00 | 0.00 | 0.00 | 0.00 | 0.00 | 0.00 | *Proteobacteria* | *Epsilonproteobacteria* | *Campylobacterales* | *Helicobacteraceae* | Transient-rare taxa |
| OTU 2411 | 0.00 | 0.00 | 0.00 | 0.01 | 0.00 | 0.00 | 0.01 | 0.00 | 0.00 | 0.00 | 0.00 | 0.00 | *Proteobacteria* | *Betaproteobacteria* | *Burkholderiales* | *Alcaligenaceae* | Transient-rare taxa |
| OTU 2412 | 0.00 | 0.02 | 0.00 | 0.00 | 0.00 | 0.00 | 0.00 | 0.00 | 0.00 | 0.00 | 0.00 | 0.00 | *Proteobacteria* | *Alphaproteobacteria* | *Rhizobiales* | *Bradyrhizobiaceae* | Transient-rare taxa |
| OTU 2413 | 0.00 | 0.01 | 0.00 | 0.00 | 0.00 | 0.00 | 0.00 | 0.00 | 0.01 | 0.00 | 0.00 | 0.00 | *Verrucomicrobia* | *Verrucomicrobiae* | *Verrucomicrobiales* | *Verrucomicrobiaceae* | Transient-rare taxa |
| OTU 2414 | 0.00 | 0.00 | 0.02 | 0.00 | 0.00 | 0.00 | 0.00 | 0.00 | 0.00 | 0.00 | 0.00 | 0.00 | *Proteobacteria* | *Gammaproteobacteria* | *Legionellales* | *Coxiellaceae* | Transient-rare taxa |
| OTU 2415 | 0.00 | 0.00 | 0.02 | 0.00 | 0.00 | 0.00 | 0.00 | 0.00 | 0.00 | 0.00 | 0.00 | 0.00 | *Proteobacteria* | *Betaproteobacteria* | *Hydrogenophilales* | *Hydrogenophilaceae* | Transient-rare taxa |
| OTU 2416 | 0.01 | 0.01 | 0.00 | 0.00 | 0.00 | 0.00 | 0.00 | 0.00 | 0.00 | 0.00 | 0.00 | 0.00 | *Proteobacteria* | *Alphaproteobacteria* | *Sphingomonadales* | *Sphingomonadaceae* | Transient-rare taxa |
| OTU 2417 | 0.00 | 0.00 | 0.00 | 0.00 | 0.00 | 0.01 | 0.01 | 0.00 | 0.00 | 0.00 | 0.00 | 0.00 | *Proteobacteria* | *Betaproteobacteria* | *Rhodocyclales* | *Rhodocyclaceae* | Transient-rare taxa |
| OTU 2418 | 0.00 | 0.00 | 0.00 | 0.00 | 0.00 | 0.00 | 0.02 | 0.00 | 0.00 | 0.00 | 0.00 | 0.00 | *Proteobacteria* | *Deltaproteobacteria* | *Myxococcales* | *Nannocystineae* | Transient-rare taxa |
| OTU 2419 | 0.00 | 0.00 | 0.00 | 0.00 | 0.02 | 0.00 | 0.00 | 0.00 | 0.00 | 0.00 | 0.00 | 0.00 | *Actinobacteria* | *Actinobacteria* | *Actinobacteridae* | *Actinomycetales* | Transient-rare taxa |
| OTU 2420 | 0.00 | 0.00 | 0.00 | 0.00 | 0.00 | 0.01 | 0.01 | 0.00 | 0.00 | 0.00 | 0.00 | 0.00 | *Bacteroidetes* | *Cytophagia* | *Cytophagales* | *Flammeovirgaceae* | Transient-rare taxa |
| OTU 2421 | 0.00 | 0.00 | 0.00 | 0.00 | 0.00 | 0.00 | 0.00 | 0.00 | 0.00 | 0.00 | 0.00 | 0.02 | *Proteobacteria* | *Alphaproteobacteria* | *Rickettsiales* | *Rickettsiaceae* | Transient-rare taxa |
| OTU 2422 | 0.00 | 0.00 | 0.00 | 0.00 | 0.00 | 0.00 | 0.02 | 0.00 | 0.00 | 0.00 | 0.00 | 0.00 | *Proteobacteria* | *Alphaproteobacteria* | *Rhodobacterales* | *Rhodobacteraceae* | Transient-rare taxa |
| OTU 2423 | 0.00 | 0.00 | 0.00 | 0.00 | 0.01 | 0.01 | 0.00 | 0.00 | 0.00 | 0.00 | 0.00 | 0.00 | *Firmicutes* | *Bacilli* | *Lactobacillales* | *Streptococcaceae* | Transient-rare taxa |
| OTU 2424 | 0.00 | 0.00 | 0.00 | 0.00 | 0.01 | 0.00 | 0.00 | 0.00 | 0.00 | 0.00 | 0.00 | 0.01 | *Proteobacteria* | *Gammaproteobacteria* | *Pseudomonadales* | *Pseudomonadaceae* | Transient-rare taxa |
| OTU 2425 | 0.00 | 0.00 | 0.00 | 0.01 | 0.00 | 0.01 | 0.00 | 0.00 | 0.00 | 0.00 | 0.00 | 0.00 | *Tenericutes* | *Mollicutes* | *Acholeplasmatales* | *Acholeplasmataceae* | Transient-rare taxa |
| OTU 2426 | 0.00 | 0.00 | 0.00 | 0.00 | 0.01 | 0.01 | 0.00 | 0.00 | 0.00 | 0.00 | 0.00 | 0.00 | *Chloroflexi* | *Thermomicrobia* | *Sphaerobacteridae* | *Sphaerobacterales* | Transient-rare taxa |
| OTU 2427 | 0.00 | 0.00 | 0.00 | 0.00 | 0.00 | 0.00 | 0.02 | 0.00 | 0.00 | 0.00 | 0.00 | 0.00 | *Planctomycetes* | *Planctomycetia* | *Planctomycetales* | *Planctomycetaceae* | Transient-rare taxa |
| OTU 2428 | 0.00 | 0.00 | 0.00 | 0.00 | 0.00 | 0.00 | 0.02 | 0.00 | 0.00 | 0.00 | 0.00 | 0.00 | *Proteobacteria* | *Alphaproteobacteria* | *Sphingomonadales* | *Sphingomonadaceae* | Transient-rare taxa |
| OTU 2429 | 0.00 | 0.00 | 0.00 | 0.00 | 0.01 | 0.01 | 0.00 | 0.00 | 0.00 | 0.00 | 0.00 | 0.00 | *Firmicutes* | *Clostridia* | *Clostridiales* | *Peptostreptococcaceae* | Transient-rare taxa |
| OTU 2430 | 0.00 | 0.00 | 0.00 | 0.00 | 0.00 | 0.02 | 0.00 | 0.00 | 0.00 | 0.00 | 0.00 | 0.00 | *Proteobacteria* | *Deltaproteobacteria* | *Myxococcales* | *Sorangiineae* | Transient-rare taxa |
| OTU 2431 | 0.00 | 0.00 | 0.00 | 0.00 | 0.00 | 0.00 | 0.02 | 0.00 | 0.00 | 0.00 | 0.00 | 0.00 | *Proteobacteria* | *Gammaproteobacteria* | *Alteromonadales* | *Alteromonadaceae* | Transient-rare taxa |
| OTU 2432 | 0.00 | 0.00 | 0.00 | 0.01 | 0.00 | 0.00 | 0.01 | 0.00 | 0.00 | 0.00 | 0.00 | 0.00 | *Proteobacteria* | *Gammaproteobacteria* | *Pseudomonadales* | *Pseudomonadaceae* | Transient-rare taxa |
| OTU 2433 | 0.00 | 0.00 | 0.00 | 0.00 | 0.01 | 0.01 | 0.00 | 0.00 | 0.00 | 0.00 | 0.00 | 0.00 | *Proteobacteria* | *Deltaproteobacteria* | *Bdellovibrionales* | *Bacteriovoracaceae* | Transient-rare taxa |
| OTU 2434 | 0.00 | 0.00 | 0.01 | 0.00 | 0.00 | 0.00 | 0.01 | 0.00 | 0.00 | 0.00 | 0.00 | 0.00 | *Proteobacteria* | *Gammaproteobacteria* | *Oceanospirillales* | *Hahellaceae* | Transient-rare taxa |
| OTU 2435 | 0.00 | 0.00 | 0.00 | 0.00 | 0.00 | 0.01 | 0.00 | 0.01 | 0.00 | 0.00 | 0.00 | 0.00 | *Proteobacteria* | *Betaproteobacteria* | *Rhodocyclales* | *Rhodocyclaceae* | Transient-rare taxa |
| OTU 2436 | 0.00 | 0.00 | 0.00 | 0.00 | 0.01 | 0.00 | 0.01 | 0.00 | 0.00 | 0.00 | 0.00 | 0.00 | *Bacteroidetes* | *Flavobacteriia* | *Flavobacteriales* | *Flavobacteriaceae* | Transient-rare taxa |
| OTU 2437 | 0.00 | 0.00 | 0.01 | 0.01 | 0.00 | 0.00 | 0.00 | 0.00 | 0.00 | 0.00 | 0.00 | 0.00 | *Proteobacteria* | *Alphaproteobacteria* | *Rhizobiales* | *Methylocystaceae* | Transient-rare taxa |
| OTU 2438 | 0.00 | 0.01 | 0.01 | 0.00 | 0.00 | 0.00 | 0.00 | 0.00 | 0.00 | 0.00 | 0.00 | 0.00 | *Bacteroidetes* | *Cytophagia* | *Cytophagales* | *Flammeovirgaceae* | Transient-rare taxa |
| OTU 2439 | 0.00 | 0.01 | 0.01 | 0.00 | 0.00 | 0.00 | 0.00 | 0.00 | 0.00 | 0.00 | 0.00 | 0.00 | *Proteobacteria* | *Betaproteobacteria* | *Hydrogenophilales* | *Hydrogenophilaceae* | Transient-rare taxa |
| OTU 2440 | 0.00 | 0.00 | 0.00 | 0.00 | 0.00 | 0.00 | 0.01 | 0.00 | 0.00 | 0.01 | 0.00 | 0.00 | *Proteobacteria* | *Gammaproteobacteria* | *Thiotrichales* | *Thiotrichaceae* | Transient-rare taxa |
| OTU 2441 | 0.00 | 0.01 | 0.01 | 0.00 | 0.00 | 0.00 | 0.00 | 0.00 | 0.00 | 0.00 | 0.00 | 0.00 | *Bacteroidetes* | *Sphingobacteriia* | *Sphingobacteriales* | *Chitinophagaceae* | Transient-rare taxa |
| OTU 2442 | 0.00 | 0.00 | 0.00 | 0.00 | 0.00 | 0.00 | 0.00 | 0.00 | 0.00 | 0.00 | 0.00 | 0.02 | *Proteobacteria* | *Deltaproteobacteria* | *Desulfobacterales* | *Desulfobacteraceae* | Transient-rare taxa |
| OTU 2443 | 0.00 | 0.00 | 0.01 | 0.00 | 0.01 | 0.00 | 0.00 | 0.00 | 0.00 | 0.00 | 0.00 | 0.00 | *Tenericutes* | *Mollicutes* | *Acholeplasmatales* | *Acholeplasmataceae* | Transient-rare taxa |
| OTU 2444 | 0.00 | 0.00 | 0.01 | 0.00 | 0.00 | 0.00 | 0.00 | 0.00 | 0.00 | 0.00 | 0.00 | 0.01 | *Bacteroidetes* | *Bacteroidia* | *Bacteroidales* | *Porphyromonadaceae* | Transient-rare taxa |
| OTU 2445 | 0.00 | 0.00 | 0.00 | 0.00 | 0.00 | 0.02 | 0.00 | 0.00 | 0.00 | 0.00 | 0.00 | 0.00 | *Proteobacteria* | *Betaproteobacteria* | *Burkholderiales* | *Burkholderiales_incertae_sedis* | Transient-rare taxa |
| OTU 2446 | 0.00 | 0.00 | 0.01 | 0.01 | 0.00 | 0.00 | 0.00 | 0.00 | 0.00 | 0.00 | 0.00 | 0.00 | *Proteobacteria* | *Gammaproteobacteria* | *Gammaproteobacteria_incertae_sedis* | *Eionea* | Transient-rare taxa |
| OTU 2447 | 0.00 | 0.00 | 0.00 | 0.00 | 0.00 | 0.02 | 0.00 | 0.00 | 0.00 | 0.00 | 0.00 | 0.00 | *Bacteroidetes* | *Sphingobacteriia* | *Sphingobacteriales* | *Chitinophagaceae* | Transient-rare taxa |
| OTU 2448 | 0.00 | 0.00 | 0.00 | 0.00 | 0.02 | 0.00 | 0.00 | 0.00 | 0.00 | 0.00 | 0.00 | 0.00 | *Spirochaetes* | *Spirochaetia* | *Spirochaetales* | *Spirochaetaceae* | Transient-rare taxa |
| OTU 2449 | 0.00 | 0.00 | 0.00 | 0.00 | 0.01 | 0.00 | 0.00 | 0.00 | 0.00 | 0.00 | 0.00 | 0.01 | *Actinobacteria* | *Actinobacteria* | *Coriobacteridae* | *Coriobacteriales* | Transient-rare taxa |
| OTU 2450 | 0.00 | 0.00 | 0.00 | 0.00 | 0.00 | 0.02 | 0.00 | 0.00 | 0.00 | 0.00 | 0.00 | 0.00 | *Proteobacteria* | *Deltaproteobacteria* | *Desulfobacterales* | *Desulfobacteraceae* | Transient-rare taxa |
| OTU 2451 | 0.00 | 0.00 | 0.00 | 0.00 | 0.00 | 0.02 | 0.00 | 0.00 | 0.00 | 0.00 | 0.00 | 0.00 | *Proteobacteria* | *Betaproteobacteria* | *Neisseriales* | *Neisseriaceae* | Transient-rare taxa |
| OTU 2452 | 0.00 | 0.00 | 0.02 | 0.00 | 0.00 | 0.00 | 0.00 | 0.00 | 0.00 | 0.00 | 0.00 | 0.00 | *Bacteroidetes* | *Flavobacteriia* | *Flavobacteriales* | *Cryomorphaceae* | Transient-rare taxa |
| OTU 2453 | 0.00 | 0.00 | 0.02 | 0.00 | 0.00 | 0.00 | 0.00 | 0.00 | 0.00 | 0.00 | 0.00 | 0.00 | *Bacteroidetes* | *Flavobacteriia* | *Flavobacteriales* | *Cryomorphaceae* | Transient-rare taxa |
| OTU 2454 | 0.00 | 0.00 | 0.01 | 0.00 | 0.00 | 0.01 | 0.00 | 0.00 | 0.00 | 0.00 | 0.00 | 0.00 | *Actinobacteria* | *Actinobacteria* | *Actinobacteridae* | *Actinomycetales* | Transient-rare taxa |
| OTU 2455 | 0.00 | 0.00 | 0.02 | 0.00 | 0.00 | 0.00 | 0.00 | 0.00 | 0.00 | 0.00 | 0.00 | 0.00 | *Proteobacteria* | *Betaproteobacteria* | *Rhodocyclales* | *Rhodocyclaceae* | Transient-rare taxa |
| OTU 2456 | 0.00 | 0.00 | 0.02 | 0.00 | 0.00 | 0.00 | 0.00 | 0.00 | 0.00 | 0.00 | 0.00 | 0.00 | *Proteobacteria* | *Betaproteobacteria* | *Rhodocyclales* | *Rhodocyclaceae* | Transient-rare taxa |
| OTU 2457 | 0.00 | 0.00 | 0.00 | 0.00 | 0.00 | 0.01 | 0.01 | 0.00 | 0.00 | 0.00 | 0.00 | 0.00 | *Proteobacteria* | *Deltaproteobacteria* | *Myxococcales* | *Sorangiineae* | Transient-rare taxa |
| OTU 2458 | 0.00 | 0.00 | 0.00 | 0.00 | 0.00 | 0.02 | 0.00 | 0.00 | 0.00 | 0.00 | 0.00 | 0.00 | *Bacteroidetes* | *Bacteroidia* | *Bacteroidales* | *Bacteroidaceae* | Transient-rare taxa |
| OTU 2459 | 0.00 | 0.00 | 0.00 | 0.00 | 0.00 | 0.01 | 0.01 | 0.00 | 0.00 | 0.00 | 0.00 | 0.00 | *Proteobacteria* | *Deltaproteobacteria* | *Myxococcales* | *Sorangiineae* | Transient-rare taxa |
| OTU 2460 | 0.00 | 0.00 | 0.00 | 0.01 | 0.00 | 0.00 | 0.01 | 0.00 | 0.00 | 0.00 | 0.00 | 0.00 | *Proteobacteria* | *Betaproteobacteria* | *Rhodocyclales* | *Rhodocyclaceae* | Transient-rare taxa |
| OTU 2461 | 0.00 | 0.00 | 0.00 | 0.00 | 0.00 | 0.02 | 0.00 | 0.00 | 0.00 | 0.00 | 0.00 | 0.00 | *Candidatus Saccharibacteria* | *Saccharibacteria_genera_incertae_sedis* | | | Transient-rare taxa |
| OTU 2462 | 0.00 | 0.00 | 0.01 | 0.00 | 0.01 | 0.00 | 0.00 | 0.00 | 0.00 | 0.00 | 0.00 | 0.00 | *Bacteroidetes* | *Cytophagia* | *Cytophagales* | *Cytophagaceae* | Transient-rare taxa |
| OTU 2463 | 0.00 | 0.00 | 0.00 | 0.00 | 0.02 | 0.00 | 0.00 | 0.00 | 0.00 | 0.00 | 0.00 | 0.00 | *Proteobacteria* | *Gammaproteobacteria* | *Alteromonadales* | *Alteromonadaceae* | Transient-rare taxa |
| OTU 2464 | 0.00 | 0.00 | 0.00 | 0.01 | 0.00 | 0.01 | 0.00 | 0.00 | 0.00 | 0.00 | 0.00 | 0.00 | *Proteobacteria* | *Betaproteobacteria* | *Rhodocyclales* | *Rhodocyclaceae* | Transient-rare taxa |
| OTU 2465 | 0.00 | 0.00 | 0.01 | 0.00 | 0.00 | 0.01 | 0.00 | 0.00 | 0.00 | 0.00 | 0.00 | 0.00 | *Bacteroidetes* | *Bacteroidia* | *Bacteroidales* | *Marinilabiliaceae* | Transient-rare taxa |
| OTU 2466 | 0.00 | 0.00 | 0.00 | 0.00 | 0.00 | 0.00 | 0.00 | 0.00 | 0.00 | 0.00 | 0.01 | 0.01 | *Verrucomicrobia* | *Opitutae* | *Opitutales* | *Opitutaceae* | Transient-rare taxa |
| OTU 2467 | 0.00 | 0.00 | 0.02 | 0.00 | 0.00 | 0.00 | 0.00 | 0.00 | 0.00 | 0.00 | 0.00 | 0.00 | *Firmicutes* | *Clostridia* | *Clostridiales* | *Clostridiales_Incertae Sedis XII* | Transient-rare taxa |
| OTU 2468 | 0.00 | 0.00 | 0.00 | 0.00 | 0.01 | 0.01 | 0.00 | 0.00 | 0.00 | 0.00 | 0.00 | 0.00 | *Bacteroidetes* | *Flavobacteriia* | *Flavobacteriales* | *Flavobacteriaceae* | Transient-rare taxa |
| OTU 2469 | 0.00 | 0.00 | 0.00 | 0.00 | 0.00 | 0.01 | 0.01 | 0.00 | 0.00 | 0.00 | 0.00 | 0.00 | *Proteobacteria* | *Epsilonproteobacteria* | *Campylobacterales* | *Helicobacteraceae* | Transient-rare taxa |
| OTU 2470 | 0.00 | 0.00 | 0.00 | 0.01 | 0.00 | 0.00 | 0.00 | 0.01 | 0.00 | 0.00 | 0.00 | 0.00 | *Proteobacteria* | *Betaproteobacteria* | *Rhodocyclales* | *Rhodocyclaceae* | Transient-rare taxa |
| OTU 2471 | 0.00 | 0.00 | 0.00 | 0.00 | 0.02 | 0.00 | 0.00 | 0.00 | 0.00 | 0.00 | 0.00 | 0.00 | *Bacteroidetes* | *Flavobacteriia* | *Flavobacteriales* | *Flavobacteriaceae* | Transient-rare taxa |
| OTU 2472 | 0.00 | 0.00 | 0.00 | 0.00 | 0.02 | 0.00 | 0.00 | 0.00 | 0.00 | 0.00 | 0.00 | 0.00 | *Bacteroidetes* | *Flavobacteriia* | *Flavobacteriales* | *Flavobacteriaceae* | Transient-rare taxa |
| OTU 2473 | 0.00 | 0.00 | 0.00 | 0.00 | 0.00 | 0.00 | 0.00 | 0.00 | 0.00 | 0.00 | 0.00 | 0.02 | *Planctomycetes* | *Planctomycetia* | *Planctomycetales* | *Planctomycetaceae* | Transient-rare taxa |
| OTU 2474 | 0.00 | 0.00 | 0.00 | 0.00 | 0.00 | 0.00 | 0.00 | 0.00 | 0.00 | 0.00 | 0.00 | 0.02 | *Bacteroidetes* | *Sphingobacteriia* | *Sphingobacteriales* | *Chitinophagaceae* | Transient-rare taxa |
| OTU 2475 | 0.00 | 0.00 | 0.00 | 0.01 | 0.00 | 0.00 | 0.01 | 0.00 | 0.00 | 0.00 | 0.00 | 0.00 | *Actinobacteria* | *Actinobacteria* | *Actinobacteridae* | *Actinomycetales* | Transient-rare taxa |
| OTU 2476 | 0.00 | 0.00 | 0.00 | 0.02 | 0.00 | 0.00 | 0.00 | 0.00 | 0.00 | 0.00 | 0.00 | 0.00 | *Bacteroidetes* | *Flavobacteriia* | *Flavobacteriales* | *Cryomorphaceae* | Transient-rare taxa |
| OTU 2477 | 0.00 | 0.00 | 0.00 | 0.00 | 0.00 | 0.02 | 0.00 | 0.00 | 0.00 | 0.00 | 0.00 | 0.00 | *Proteobacteria* | *Deltaproteobacteria* | *Desulfuromonadales* | *Geobacteraceae* | Transient-rare taxa |
| OTU 2478 | 0.00 | 0.00 | 0.00 | 0.00 | 0.00 | 0.02 | 0.00 | 0.00 | 0.00 | 0.00 | 0.00 | 0.00 | *Proteobacteria* | *Alphaproteobacteria* | *Rhodobacterales* | *Rhodobacteraceae* | Transient-rare taxa |
| OTU 2479 | 0.00 | 0.00 | 0.00 | 0.00 | 0.00 | 0.00 | 0.02 | 0.00 | 0.00 | 0.00 | 0.00 | 0.00 | *Proteobacteria* | *Betaproteobacteria* | *Burkholderiales* | *Burkholderiaceae* | Transient-rare taxa |
| OTU 2480 | 0.00 | 0.00 | 0.02 | 0.00 | 0.00 | 0.00 | 0.00 | 0.00 | 0.00 | 0.00 | 0.00 | 0.00 | *Proteobacteria* | *Gammaproteobacteria* | *Oceanospirillales* | *Hahellaceae* | Transient-rare taxa |
| OTU 2481 | 0.00 | 0.00 | 0.02 | 0.00 | 0.00 | 0.00 | 0.00 | 0.00 | 0.00 | 0.00 | 0.00 | 0.00 | *Proteobacteria* | *Alphaproteobacteria* | *Caulobacterales* | *Hyphomonadaceae* | Transient-rare taxa |
| OTU 2482 | 0.00 | 0.00 | 0.01 | 0.00 | 0.00 | 0.01 | 0.00 | 0.00 | 0.00 | 0.00 | 0.00 | 0.00 | *Firmicutes* | *Clostridia* | *Natranaerobiales* | *Natranaerobiaceae* | Transient-rare taxa |
| OTU 2483 | 0.00 | 0.00 | 0.00 | 0.00 | 0.00 | 0.01 | 0.00 | 0.01 | 0.00 | 0.00 | 0.00 | 0.00 | *Planctomycetes* | *Planctomycetia* | *Planctomycetales* | *Planctomycetaceae* | Transient-rare taxa |
| OTU 2484 | 0.00 | 0.00 | 0.00 | 0.00 | 0.00 | 0.00 | 0.00 | 0.00 | 0.02 | 0.00 | 0.00 | 0.00 | *Proteobacteria* | *Deltaproteobacteria* | *Bdellovibrionales* | *Bdellovibrionaceae* | Transient-rare taxa |
| OTU 2485 | 0.00 | 0.00 | 0.00 | 0.00 | 0.00 | 0.00 | 0.02 | 0.00 | 0.00 | 0.00 | 0.00 | 0.00 | *Proteobacteria* | *Alphaproteobacteria* | *Rhodospirillales* | *Rhodospirillaceae* | Transient-rare taxa |
| OTU 2486 | 0.00 | 0.00 | 0.00 | 0.00 | 0.00 | 0.02 | 0.00 | 0.00 | 0.00 | 0.00 | 0.00 | 0.00 | *Proteobacteria* | *Alphaproteobacteria* | *Sphingomonadales* | *Sphingomonadaceae* | Transient-rare taxa |
| OTU 2487 | 0.02 | 0.00 | 0.00 | 0.00 | 0.00 | 0.00 | 0.00 | 0.00 | 0.00 | 0.00 | 0.00 | 0.00 | *Proteobacteria* | *Gammaproteobacteria* | *Xanthomonadales* | *Xanthomonadaceae* | Transient-rare taxa |
| OTU 2488 | 0.01 | 0.00 | 0.01 | 0.00 | 0.00 | 0.00 | 0.00 | 0.00 | 0.00 | 0.00 | 0.00 | 0.00 | *Proteobacteria* | *Betaproteobacteria* | *Rhodocyclales* | *Rhodocyclaceae* | Transient-rare taxa |
| OTU 2489 | 0.00 | 0.00 | 0.02 | 0.00 | 0.00 | 0.00 | 0.00 | 0.00 | 0.00 | 0.00 | 0.00 | 0.00 | *Proteobacteria* | *Betaproteobacteria* | *Nitrosomonadales* | *Nitrosomonadaceae* | Transient-rare taxa |
| OTU 2490 | 0.02 | 0.00 | 0.00 | 0.00 | 0.00 | 0.00 | 0.00 | 0.00 | 0.00 | 0.00 | 0.00 | 0.00 | *Parcubacteria* | *Parcubacteria_genera_incertae_sedis* | | | Transient-rare taxa |
| OTU 2491 | 0.00 | 0.00 | 0.00 | 0.00 | 0.00 | 0.02 | 0.00 | 0.00 | 0.00 | 0.00 | 0.00 | 0.00 | *Bacteroidetes* | *Sphingobacteriia* | *Sphingobacteriales* | *Saprospiraceae* | Transient-rare taxa |
| OTU 2492 | 0.00 | 0.00 | 0.02 | 0.00 | 0.00 | 0.00 | 0.00 | 0.00 | 0.00 | 0.00 | 0.00 | 0.00 | *Nitrospirae* | *Nitrospira* | *Nitrospirales* | *Nitrospiraceae* | Transient-rare taxa |
| OTU 2493 | 0.00 | 0.00 | 0.00 | 0.00 | 0.00 | 0.00 | 0.00 | 0.00 | 0.02 | 0.00 | 0.00 | 0.00 | *Bacteroidetes* | *Cytophagia* | *Cytophagales* | *Cytophagaceae* | Transient-rare taxa |
| OTU 2494 | 0.00 | 0.00 | 0.01 | 0.00 | 0.00 | 0.00 | 0.00 | 0.00 | 0.00 | 0.00 | 0.00 | 0.01 | *Bacteroidetes* | *Bacteroidia* | *Bacteroidales* | *Porphyromonadaceae* | Transient-rare taxa |
| OTU 2495 | 0.02 | 0.00 | 0.00 | 0.00 | 0.00 | 0.00 | 0.00 | 0.00 | 0.00 | 0.00 | 0.00 | 0.00 | *Proteobacteria* | *Gammaproteobacteria* | *Chromatiales* | *Chromatiaceae* | Transient-rare taxa |
| OTU 2496 | 0.02 | 0.00 | 0.00 | 0.00 | 0.00 | 0.00 | 0.00 | 0.00 | 0.00 | 0.00 | 0.00 | 0.00 | *Proteobacteria* | *Betaproteobacteria* | *Burkholderiales* | *Comamonadaceae* | Transient-rare taxa |
| OTU 2497 | 0.00 | 0.00 | 0.00 | 0.00 | 0.00 | 0.02 | 0.00 | 0.00 | 0.00 | 0.00 | 0.00 | 0.00 | *Bacteroidetes* | *Cytophagia* | *Cytophagales* | *Flammeovirgaceae* | Transient-rare taxa |
| OTU 2498 | 0.02 | 0.00 | 0.00 | 0.00 | 0.00 | 0.00 | 0.00 | 0.00 | 0.00 | 0.00 | 0.00 | 0.00 | *Planctomycetes* | *Phycisphaerae* | *Phycisphaerales* | *Phycisphaeraceae* | Transient-rare taxa |
| OTU 2499 | 0.01 | 0.00 | 0.00 | 0.00 | 0.00 | 0.01 | 0.00 | 0.00 | 0.00 | 0.00 | 0.00 | 0.00 | *Proteobacteria* | *Alphaproteobacteria* | *Rhodobacterales* | *Rhodobacteraceae* | Transient-rare taxa |
| OTU 2500 | 0.00 | 0.00 | 0.00 | 0.00 | 0.00 | 0.01 | 0.01 | 0.00 | 0.00 | 0.00 | 0.00 | 0.00 | *Proteobacteria* | *Gammaproteobacteria* | *Chromatiales* | *Ectothiorhodospiraceae* | Transient-rare taxa |
| OTU 2501 | 0.00 | 0.00 | 0.00 | 0.00 | 0.00 | 0.00 | 0.01 | 0.00 | 0.00 | 0.01 | 0.00 | 0.00 | *Proteobacteria* | *Deltaproteobacteria* | *Myxococcales* | *Nannocystineae* | Transient-rare taxa |
| OTU 2502 | 0.01 | 0.00 | 0.01 | 0.00 | 0.00 | 0.00 | 0.00 | 0.00 | 0.00 | 0.00 | 0.00 | 0.00 | *Proteobacteria* | *Gammaproteobacteria* | *Chromatiales* | *Chromatiaceae* | Transient-rare taxa |
| OTU 2503 | 0.00 | 0.00 | 0.00 | 0.00 | 0.00 | 0.00 | 0.00 | 0.00 | 0.00 | 0.00 | 0.00 | 0.02 | *Proteobacteria* | *Betaproteobacteria* | *Burkholderiales* | *Burkholderiales_incertae_sedis* | Transient-rare taxa |
| OTU 2504 | 0.00 | 0.00 | 0.00 | 0.00 | 0.00 | 0.02 | 0.00 | 0.00 | 0.00 | 0.00 | 0.00 | 0.00 | *Bacteroidetes* | *Cytophagia* | *Cytophagales* | *Cytophagaceae* | Transient-rare taxa |
| OTU 2505 | 0.00 | 0.00 | 0.00 | 0.00 | 0.01 | 0.01 | 0.00 | 0.00 | 0.00 | 0.00 | 0.00 | 0.00 | *Proteobacteria* | *Gammaproteobacteria* | *Xanthomonadales* | *Xanthomonadaceae* | Transient-rare taxa |
| OTU 2506 | 0.00 | 0.00 | 0.01 | 0.00 | 0.00 | 0.00 | 0.01 | 0.00 | 0.00 | 0.00 | 0.00 | 0.00 | *Proteobacteria* | *Betaproteobacteria* | *Nitrosomonadales* | *Nitrosomonadaceae* | Transient-rare taxa |
| OTU 2507 | 0.00 | 0.00 | 0.00 | 0.00 | 0.00 | 0.02 | 0.00 | 0.00 | 0.00 | 0.00 | 0.00 | 0.00 | *Planctomycetes* | *Planctomycetia* | *Planctomycetales* | *Planctomycetaceae* | Transient-rare taxa |
| OTU 2508 | 0.00 | 0.00 | 0.00 | 0.00 | 0.00 | 0.00 | 0.00 | 0.00 | 0.00 | 0.00 | 0.00 | 0.02 | *Proteobacteria* | *Alphaproteobacteria* | *Sphingomonadales* | *Sphingomonadaceae* | Transient-rare taxa |
| OTU 2509 | 0.00 | 0.00 | 0.02 | 0.00 | 0.00 | 0.00 | 0.00 | 0.00 | 0.00 | 0.00 | 0.00 | 0.00 | *Proteobacteria* | *Epsilonproteobacteria* | *Nautiliales* | *Nautiliaceae* | Transient-rare taxa |
| OTU 2510 | 0.02 | 0.00 | 0.00 | 0.00 | 0.00 | 0.00 | 0.00 | 0.00 | 0.00 | 0.00 | 0.00 | 0.00 | *Proteobacteria* | *Deltaproteobacteria* | *Myxococcales* | *Nannocystineae* | Transient-rare taxa |
| OTU 2511 | 0.01 | 0.00 | 0.01 | 0.00 | 0.00 | 0.00 | 0.00 | 0.00 | 0.00 | 0.00 | 0.00 | 0.00 | *Proteobacteria* | *Alphaproteobacteria* | *Rhizobiales* | *Hyphomicrobiaceae* | Transient-rare taxa |
| OTU 2512 | 0.00 | 0.00 | 0.00 | 0.00 | 0.00 | 0.02 | 0.00 | 0.00 | 0.00 | 0.00 | 0.00 | 0.00 | *Proteobacteria* | *Gammaproteobacteria* | *Oceanospirillales* | *Oceanospirillaceae* | Transient-rare taxa |
| OTU 2513 | 0.00 | 0.00 | 0.00 | 0.00 | 0.00 | 0.02 | 0.00 | 0.00 | 0.00 | 0.00 | 0.00 | 0.00 | *Actinobacteria* | *Actinobacteria* | *Actinobacteridae* | *Actinomycetales* | Transient-rare taxa |
| OTU 2514 | 0.00 | 0.00 | 0.00 | 0.00 | 0.00 | 0.02 | 0.00 | 0.00 | 0.00 | 0.00 | 0.00 | 0.00 | *Bacteroidetes* | *Flavobacteriia* | *Flavobacteriales* | *Flavobacteriaceae* | Transient-rare taxa |
| OTU 2515 | 0.02 | 0.00 | 0.00 | 0.00 | 0.00 | 0.00 | 0.00 | 0.00 | 0.00 | 0.00 | 0.00 | 0.00 | *Planctomycetes* | *Planctomycetia* | *Candidatus Brocadiales* | *Candidatus Brocadiaceae* | Transient-rare taxa |
| OTU 2516 | 0.00 | 0.00 | 0.00 | 0.00 | 0.00 | 0.00 | 0.00 | 0.00 | 0.00 | 0.02 | 0.00 | 0.00 | *Firmicutes* | *Clostridia* | *Clostridiales* | *Clostridiales_Incertae Sedis XII* | Transient-rare taxa |
| OTU 2517 | 0.00 | 0.00 | 0.00 | 0.00 | 0.01 | 0.00 | 0.01 | 0.00 | 0.00 | 0.00 | 0.00 | 0.00 | *Proteobacteria* | *Alphaproteobacteria* | *Rhodobacterales* | *Rhodobacteraceae* | Transient-rare taxa |
| OTU 2518 | 0.00 | 0.00 | 0.00 | 0.00 | 0.00 | 0.02 | 0.00 | 0.00 | 0.00 | 0.00 | 0.00 | 0.00 | *Proteobacteria* | *Alphaproteobacteria* | *Rhizobiales* | *Rhizobiaceae* | Transient-rare taxa |
| OTU 2519 | 0.02 | 0.00 | 0.00 | 0.00 | 0.00 | 0.00 | 0.00 | 0.00 | 0.00 | 0.00 | 0.00 | 0.00 | *Proteobacteria* | *Betaproteobacteria* | *Rhodocyclales* | *Rhodocyclaceae* | Transient-rare taxa |
| OTU 2520 | 0.00 | 0.00 | 0.01 | 0.00 | 0.00 | 0.00 | 0.01 | 0.00 | 0.00 | 0.00 | 0.00 | 0.00 | *Bacteroidetes* | *Flavobacteriia* | *Flavobacteriales* | *Flavobacteriaceae* | Transient-rare taxa |
| OTU 2521 | 0.00 | 0.00 | 0.02 | 0.00 | 0.00 | 0.00 | 0.00 | 0.00 | 0.00 | 0.00 | 0.00 | 0.00 | *Firmicutes* | *Clostridia* | *Clostridiales* | *Lachnospiraceae* | Transient-rare taxa |
| OTU 2522 | 0.00 | 0.00 | 0.02 | 0.00 | 0.00 | 0.00 | 0.00 | 0.00 | 0.00 | 0.00 | 0.00 | 0.00 | *Bacteroidetes* | *Bacteroidia* | *Bacteroidales* | *Marinilabiliaceae* | Transient-rare taxa |
| OTU 2523 | 0.00 | 0.00 | 0.02 | 0.00 | 0.00 | 0.00 | 0.00 | 0.00 | 0.00 | 0.00 | 0.00 | 0.00 | *Proteobacteria* | *Gammaproteobacteria* | *Oceanospirillales* | *Oceanospirillaceae* | Transient-rare taxa |
| OTU 2524 | 0.00 | 0.00 | 0.00 | 0.00 | 0.00 | 0.00 | 0.00 | 0.00 | 0.01 | 0.00 | 0.00 | 0.01 | *Proteobacteria* | *Alphaproteobacteria* | *Rhodobacterales* | *Rhodobacteraceae* | Transient-rare taxa |
| OTU 2525 | 0.00 | 0.00 | 0.00 | 0.00 | 0.00 | 0.00 | 0.00 | 0.00 | 0.02 | 0.00 | 0.00 | 0.00 | *Proteobacteria* | *Alphaproteobacteria* | *Rhodospirillales* | *Rhodospirillaceae* | Transient-rare taxa |
| OTU 2526 | 0.01 | 0.00 | 0.00 | 0.00 | 0.00 | 0.00 | 0.00 | 0.00 | 0.01 | 0.00 | 0.00 | 0.00 | *Proteobacteria* | *Gammaproteobacteria* | *Xanthomonadales* | *Xanthomonadaceae* | Transient-rare taxa |
| OTU 2527 | 0.00 | 0.00 | 0.00 | 0.00 | 0.02 | 0.00 | 0.00 | 0.00 | 0.00 | 0.00 | 0.00 | 0.00 | *Chloroflexi* | *Anaerolineae* | *Anaerolineales* | *Anaerolineaceae* | Transient-rare taxa |
| OTU 2528 | 0.01 | 0.00 | 0.00 | 0.00 | 0.00 | 0.00 | 0.00 | 0.00 | 0.00 | 0.00 | 0.00 | 0.01 | *Chloroflexi* | *Anaerolineae* | *Anaerolineales* | *Anaerolineaceae* | Transient-rare taxa |
| OTU 2529 | 0.00 | 0.00 | 0.02 | 0.00 | 0.00 | 0.00 | 0.00 | 0.00 | 0.00 | 0.00 | 0.00 | 0.00 | *Bacteroidetes* | *Flavobacteriia* | *Flavobacteriales* | *Cryomorphaceae* | Transient-rare taxa |
| OTU 2530 | 0.00 | 0.00 | 0.01 | 0.00 | 0.00 | 0.00 | 0.00 | 0.01 | 0.00 | 0.00 | 0.00 | 0.00 | *Proteobacteria* | *Gammaproteobacteria* | *Thiotrichales* | *Thiotrichaceae* | Transient-rare taxa |
| OTU 2531 | 0.00 | 0.00 | 0.00 | 0.00 | 0.00 | 0.02 | 0.00 | 0.00 | 0.00 | 0.00 | 0.00 | 0.00 | *Proteobacteria* | *Deltaproteobacteria* | *Myxococcales* | *Cystobacterineae* | Transient-rare taxa |
| OTU 2532 | 0.00 | 0.00 | 0.00 | 0.00 | 0.00 | 0.01 | 0.00 | 0.00 | 0.01 | 0.00 | 0.00 | 0.00 | *Proteobacteria* | *Alphaproteobacteria* | *Rhodobacterales* | *Rhodobacteraceae* | Transient-rare taxa |
| OTU 2533 | 0.00 | 0.00 | 0.00 | 0.00 | 0.00 | 0.00 | 0.00 | 0.00 | 0.00 | 0.00 | 0.00 | 0.02 | *Proteobacteria* | *Gammaproteobacteria* | *Xanthomonadales* | *Xanthomonadaceae* | Transient-rare taxa |
| OTU 2534 | 0.00 | 0.00 | 0.00 | 0.00 | 0.00 | 0.00 | 0.00 | 0.02 | 0.00 | 0.00 | 0.00 | 0.00 | *Chloroflexi* | *Anaerolineae* | *Anaerolineales* | *Anaerolineaceae* | Transient-rare taxa |
| OTU 2535 | 0.00 | 0.00 | 0.00 | 0.00 | 0.02 | 0.00 | 0.00 | 0.00 | 0.00 | 0.00 | 0.00 | 0.00 | *Bacteroidetes* | *Flavobacteriia* | *Flavobacteriales* | *Flavobacteriaceae* | Transient-rare taxa |
| OTU 2536 | 0.00 | 0.00 | 0.00 | 0.00 | 0.00 | 0.00 | 0.00 | 0.00 | 0.00 | 0.00 | 0.02 | 0.00 | *Actinobacteria* | *Actinobacteria* | *Actinobacteridae* | *Actinomycetales* | Transient-rare taxa |
| OTU 2537 | 0.00 | 0.00 | 0.00 | 0.00 | 0.00 | 0.00 | 0.00 | 0.02 | 0.00 | 0.00 | 0.00 | 0.00 | *Proteobacteria* | *Epsilonproteobacteria* | *Campylobacterales* | *Campylobacteraceae* | Transient-rare taxa |
| OTU 2538 | 0.00 | 0.00 | 0.00 | 0.00 | 0.02 | 0.00 | 0.00 | 0.00 | 0.00 | 0.00 | 0.00 | 0.00 | *Cyanobacteria/Chloroplast* | *Chloroplast* | *Chloroplast* | *Cryptomonadaceae* | Transient-rare taxa |
| OTU 2539 | 0.00 | 0.02 | 0.00 | 0.00 | 0.00 | 0.00 | 0.00 | 0.00 | 0.00 | 0.00 | 0.00 | 0.00 | *Proteobacteria* | *Betaproteobacteria* | *Rhodocyclales* | *Rhodocyclaceae* | Transient-rare taxa |
| OTU 2540 | 0.00 | 0.00 | 0.00 | 0.00 | 0.00 | 0.00 | 0.00 | 0.00 | 0.00 | 0.00 | 0.02 | 0.00 | *Firmicutes* | *Bacilli* | *Bacillales* | *Bacillaceae 1* | Transient-rare taxa |
| OTU 2541 | 0.00 | 0.00 | 0.00 | 0.00 | 0.00 | 0.00 | 0.00 | 0.01 | 0.00 | 0.01 | 0.00 | 0.00 | *Proteobacteria* | *Betaproteobacteria* | *Rhodocyclales* | *Rhodocyclaceae* | Transient-rare taxa |
| OTU 2542 | 0.00 | 0.00 | 0.00 | 0.00 | 0.00 | 0.00 | 0.00 | 0.02 | 0.00 | 0.00 | 0.00 | 0.00 | *Proteobacteria* | *Gammaproteobacteria* | *Oceanospirillales* | *Oceanospirillaceae* | Transient-rare taxa |
| OTU 2543 | 0.00 | 0.00 | 0.02 | 0.00 | 0.00 | 0.00 | 0.00 | 0.00 | 0.00 | 0.00 | 0.00 | 0.00 | *Proteobacteria* | *Betaproteobacteria* | *Rhodocyclales* | *Rhodocyclaceae* | Transient-rare taxa |
| OTU 2544 | 0.00 | 0.00 | 0.00 | 0.00 | 0.00 | 0.00 | 0.00 | 0.02 | 0.00 | 0.00 | 0.00 | 0.00 | *Proteobacteria* | *Deltaproteobacteria* | *Myxococcales* | *Nannocystineae* | Transient-rare taxa |

a Transient-rare taxa were defiend as the OTUs

* OTUs belonging to the conditionally rare taxa (2%) were bolded.

[b] The values indicate average and range (minimum to maximum)

**Table S6.** Goodness of fit test for the dynamically changed taxa definition.

(A)

| Transient-rare taxa | Occurrence frequency | | | | |
|---|---|---|---|---|---|
| Log-series | 6 | 5 | 4 | 3 | 2 |
| Relative abundance 4.000 | 4155.3 | 2543.5 | 45.5 | 89.4 | 310.9 |
| 3.000 | 4155.3 | 2543.5 | 45.5 | 89.4 | 310.9 |
| 2.000 | 1905.3 | 2543.5 | 45.5 | 89.4 | 310.9 |
| 1.500 | 1905.3 | 2543.5 | 45.5 | 89.4 | 310.9 |
| 1.200 | 1905.3 | 2543.5 | 45.5 | 89.4 | 310.9 |
| 1.000 | 240.9 | 189.4 | 152.9 | 89.4 | 310.9 |
| 0.800 | 240.9 | 189.4 | 152.9 | 89.4 | 310.9 |
| 0.500 | 199.1 | 186.3 | 152.9 | 89.4 | 310.9 |
| 0.300 | 316.6 | 422.0 | 303.7 | 192.3 | 338.6 |
| 0.100 | 197.4 | 236.4 | 281.4 | 214.0 | 113.5 |
| 0.075 | 474.1 | 603.8 | 789.1 | 1794.7 | 726.2 |
| 0.050 | 419.3 | 472.6 | 569.3 | 1771.6 | 477.2 |

(B)

| Common taxa | Occurrence frequency | | | | |
|---|---|---|---|---|---|
| Log-series | 6 | 5 | 4 | 3 | 2 |
| Relative abundance 4.000 | 22.6 | 16.4 | 32.5 | 32.8 | 288.4 |
| 3.000 | 22.6 | 16.4 | 32.5 | 32.8 | 288.4 |
| 2.000 | 23.7 | 16.4 | 32.5 | 32.8 | 288.4 |
| 1.500 | 23.7 | 16.4 | 32.5 | 32.8 | 288.4 |
| 1.200 | 23.7 | 16.4 | 32.5 | 32.8 | 288.4 |
| 1.000 | 27.3 | 19.0 | 33.3 | 32.8 | 288.4 |
| 0.800 | 27.3 | 19.0 | 33.3 | 32.8 | 288.4 |
| 0.500 | 21.7 | 18.7 | 33.3 | 32.8 | 288.4 |
| 0.300 | 19.7 | 13.8 | 32.5 | 32.8 | 289.0 |
| 0.100 | 23.2 | 10.8 | 28.0 | 29.8 | 306.3 |
| 0.075 | 21.1 | 20.4 | 27.5 | 35.5 | 323.9 |
| 0.050 | 57.4 | 59.8 | 49.5 | 42.9 | 349.9 |

*Blue colored area represents log-normal fit, and red colored area represents log-series fit.

**Table S7.** Dynamic neutrality test for the transient-rare and the common taxa in various dataset.

| Transient-rare taxa | | # of non-neutral taxa | | | | |
|---|---|---|---|---|---|---|
| | | **Occurrence frequency** | | | | |
| | | 6 | 5 | 4 | 3 | 2 |
| **Relative abundance (%)** | 4.000 | 93.4 | 94.5 | 95.6 | 96.4 | 97.1 |
| | 3.000 | 93.4 | 94.5 | 95.6 | 96.4 | 97.1 |
| | 2.000 | 93.4 | 94.5 | 95.6 | 96.4 | 97.1 |
| | 1.500 | 93.4 | 94.5 | 95.6 | 96.4 | 97.1 |
| | 1.200 | 93.4 | 94.5 | 95.6 | 96.4 | 97.1 |
| | 1.000 | 93.5 | 94.6 | 95.6 | 96.4 | 97.1 |
| | 0.800 | 93.5 | 94.6 | 95.6 | 96.4 | 97.1 |
| | 0.500 | 93.7 | 94.7 | 95.6 | 96.4 | 97.1 |
| | 0.300 | 94.1 | 95.2 | 96.0 | 96.6 | 97.2 |
| | 0.100 | 95.9 | 97.0 | 97.5 | 97.5 | 97.6 |
| | 0.075 | 96.8 | 97.9 | 98.5 | 98.4 | 98.1 |
| | 0.050 | 97.9 | 98.6 | 99.3 | 99.2 | 99.1 |

| Common taxa | | # of non-neutral taxa | | | | |
|---|---|---|---|---|---|---|
| | | **Occurrence frequency** | | | | |
| | | 6 | 5 | 4 | 3 | 2 |
| **Relative abundance (%)** | 4.000 | 6.6 | 5.5 | 4.4 | 3.6 | 2.9 |
| | 3.000 | 6.6 | 5.5 | 4.4 | 3.6 | 2.9 |
| | 2.000 | 6.6 | 5.5 | 4.4 | 3.6 | 2.9 |
| | 1.500 | 6.6 | 5.5 | 4.4 | 3.6 | 2.9 |
| | 1.200 | 6.6 | 5.5 | 4.4 | 3.6 | 2.9 |
| | 1.000 | 6.5 | 5.4 | 4.4 | 3.6 | 2.9 |
| | 0.800 | 6.5 | 5.4 | 4.4 | 3.6 | 2.9 |
| | 0.500 | 6.3 | 5.3 | 4.4 | 3.6 | 2.9 |
| | 0.300 | 5.9 | 4.8 | 4.0 | 3.4 | 2.8 |
| | 0.100 | 4.1 | 3.0 | 2.5 | 2.5 | 2.4 |
| | 0.075 | 3.2 | 2.1 | 1.5 | 1.6 | 1.9 |
| | 0.050 | 2.1 | 1.4 | 0.7 | 0.8 | 0.9 |

**Table S8**. Goodness of fit test for the persistent-rare taxa.

| Persistent-rare taxa | | Occurrence frequency | | | | | | | | |
|---|---|---|---|---|---|---|---|---|---|---|
| | | 12 | 11 | 10 | 9 | 8 | 7 | 6 | 5 | 4 |
| Relative abundance (%) | 4.0 | 2051.6 | 3017.3 | 3400.8 | 4517.7 | 4258.6 | 4155.3 | 4155.3 | 2543.5 | 45.5 |
| | 3.0 | 2051.6 | 3017.3 | 3400.8 | 4517.7 | 4258.6 | 4155.3 | 4155.3 | 2543.5 | 45.5 |
| | 2.0 | 1818.8 | 1803.0 | 2683.2 | 1826.8 | 1888.8 | 1957.9 | 1905.3 | 2543.5 | 45.5 |
| | 1.5 | 1134.0 | 1803.0 | 1896.7 | 1826.8 | 1888.8 | 98.4 | 1905.3 | 2543.5 | 45.5 |
| | 1.2 | 1195.9 | 1677.2 | 2056.2 | 1968.2 | 1888.8 | 98.4 | 1905.3 | 2543.5 | 45.5 |
| | 1 | 76.4 | 105.7 | 139.1 | 127.5 | 113.4 | 260.4 | 240.9 | 189.4 | 152.9 |
| | 0.8 | 64.2 | 93.1 | 139.1 | 127.5 | 113.4 | 260.4 | 240.9 | 189.4 | 152.9 |
| | 0.5 | 203.4 | 233.2 | 244.9 | 255.4 | 266.9 | 221.4 | 199.1 | 186.3 | 152.9 |
| | 0.3 | 204.4 | 232.7 | 236.5 | 280.4 | 329.2 | 317.2 | 316.6 | 422.0 | 303.7 |
| | 0.1 | 125.8 | 125.8 | 125.9 | 134.0 | 141.8 | 168.4 | 197.4 | 236.4 | 281.4 |
| | 0.075 | 361.4 | 361.4 | 363.6 | 366.9 | 379.8 | 426.8 | 474.1 | 603.8 | 789.1 |
| | 0.05 | 387.4 | 387.4 | 387.4 | 387.4 | 388.8 | 407.5 | 419.3 | 472.6 | 569.3 |

*Blue colored area represents log-normal fit, and red colored area represents log-series fit.

**Fig. S1.**

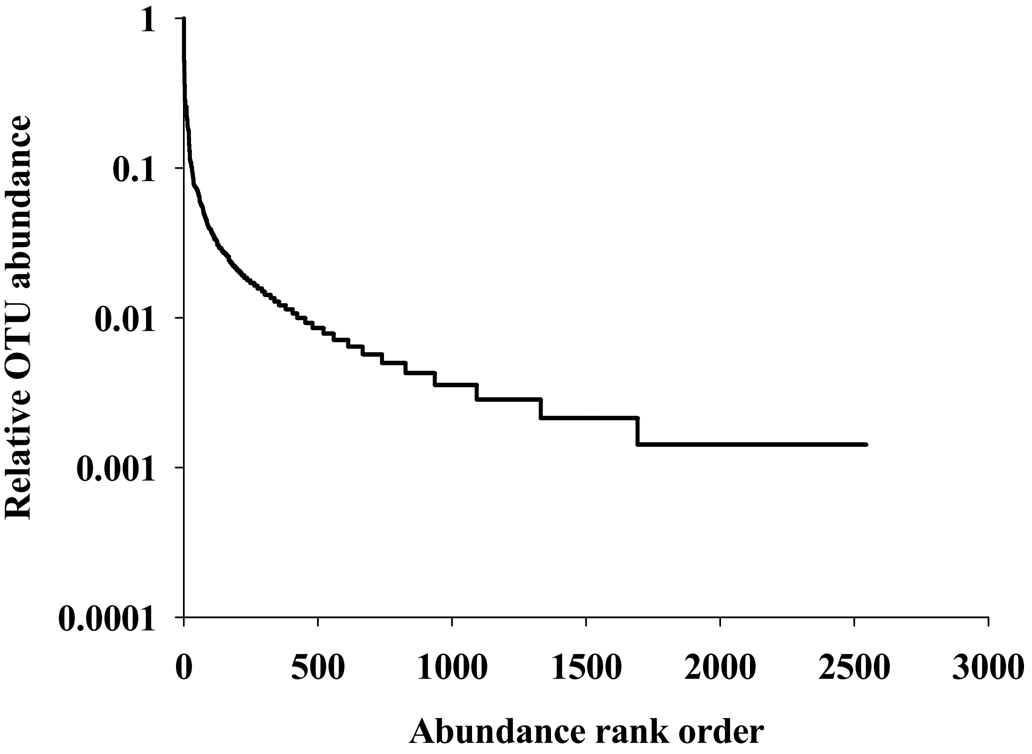

1
0.1
0.01
0.001
0.0001
Relative OTU abundance
0
500
1000
1500
2000
2500
3000
Abundance rank order

**Fig. S2.**

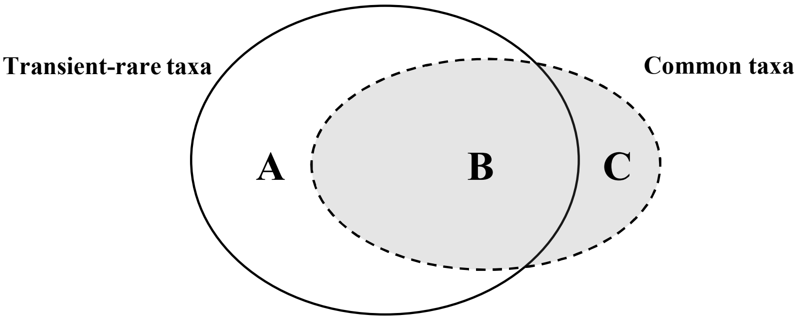


**A. 60 families**

| | |
|---|---|
| Acholeplasmatacea | Anaplasmataceae |
| Aquificaceae | Ardenticatenaceae |
| Bacillaceae 2 | Bacteriovoracaceae |
| Bacteroidaceae | Brucellaceae |
| Celerinatantimonadaceae | |
| Chlorobiaceae | Clostridiaceae 3 |
| Clostridiales_Incertae Sedis XI | |
| Clostridiales_Incertae Sedis XIII | |
| Cocleimonas | Coriobacteriales |
| Coxiellaceae | Cryptomonadaceae |
| Deferribacterales_incertae_sedis | |
| Desulfovibrionaceae | Desulfuromonadaceae |
| Eionea | Elioraea |
| Ferrovaceae | Fimbriimonadaceae |
| Flammeovirgaceae | Francisellaceae |
| Gemmatimonadaceae | |
| Gracilibacteraceae | Haloplasmataceae |
| Lachnospiraceae | Leptospiraceae |
| Maricurvus | Methylohalomonas |
| Natranaerobiaceae | Natranaerovirga |
| Nautiliaceae | Oleiphilaceae |
| Oligosphaeraceae | Oxalobacteraceae |
| Peptostreptococcaceae | |
| Piscirickettsiaceae | Prevotellaceae |
| Pseudoalteromonadaceae | |
| Psychromonadaceae | Puniceicoccaceae |
| Rhizomicrobium | Rhodobiaceae |
| Solirubrobacterales | Streptococcaceae |
| Sunxiuqinia | Synergistaceae |
| Syntrophaceae | Syntrophobacteraceae |
| Thermodesulfobacteriaceae | |
| Thiohalobacter | Umboniibacter |
| Veillonellaceae | Verrucomicrobiaceae |
| Victivallaceae | Xanthobacteraceae |

**B. 91 families**

| | |
|---|---|
| Acetobacteraceae | Acidimicrobiales |
| Actinomycetales | Aeromonadaceae |
| Alcaligenaceae | Alteromonadaceae |
| Anaerolineaceae | Bacillaceae 1 |
| Bdellovibrionaceae | Beijerinckiaceae |
| Bradyrhizobiaceae | Burkholderiaceae |
| Burkholderiales_incertae_sedis | |
| Caldilineaceae | Campylobacteraceae |
| Candidatus | Brocadiaceae |
| Carnobacteriaceae | Caulobacteraceae |
| Chitinophagaceae | Chromatiaceae |
| Clostridiaceae 1 | Clostridiales_Incertae Sedis XII |
| Comamonadaceae | Cryomorphaceae |
| Cyclobacteriaceae | Cystobacterineae |
| Cytophagaceae | Desulfobacteraceae |
| Desulfobulbaceae | Ectothiorhodospiraceae |
| Erysipelotrichaceae | Erythrobacteraceae |
| Flavobacteriaceae | Fusobacteriaceae |
| Gaiellales | Gallionellaceae |
| Geminicoccus | Geobacteraceae |
| Hahellaceae | Halomonadaceae |
| Halothiobacillaceae | Helicobacteraceae |
| Holophagaceae | Hydrogenophilaceae |
| Hyphomicrobiaceae | Hyphomonadaceae |
| Ignavibacteriaceae | Kiloniellaceae |
| Legionellaceae | Marinilabiaceae |
| Marinilabiliaceae | Mariprofundaceae |
| Methylobacteriaceae | Methylococcaceae |
| Methylocystaceae | Moraxellaceae |
| Nannocystineae | Neisseriaceae |
| Nitrosomonadaceae | Nitrospiraceae |
| Oceanospirillaceae | Opitutaceae |
| Phycisphaeraceae | Phyllobacteriaceae |
| Planctomycetaceae | Porphyromonadaceae |
| Porticoccus | Pseudomonadaceae |
| Rhizobiaceae | Rhizobiales_incertae_sedis |
| Rhodobacteraceae | Rhodocyclaceae |
| Rhodospirillaceae | Rickettsiaceae |
| Ruminococcaceae | Saprospiraceae |
| Sedimenticola | Simiduia |
| Sinobacteraceae | Sneathiellaceae |
| Sorangiineae | Sphaerobacterales |
| Sphingobacteriaceae | Sphingomonadaceae |
| Spirochaetaceae | Thermotogaceae |
| Thiohalophilus | Thiotrichaceae |
| Xanthomonadaceae | Unclassified_family |

**C. 11 families**

Congregibacter
Desulfarculaceae
Enterobacteriaceae
Flammeovirgaceae
Hydrogenothermaceae
Lentisphaeraceae
Procabacteriaceae
Thermomicrobiaceae
Thiohalomonas
Thioprofundum
Victivallaceae

Fig. S3.

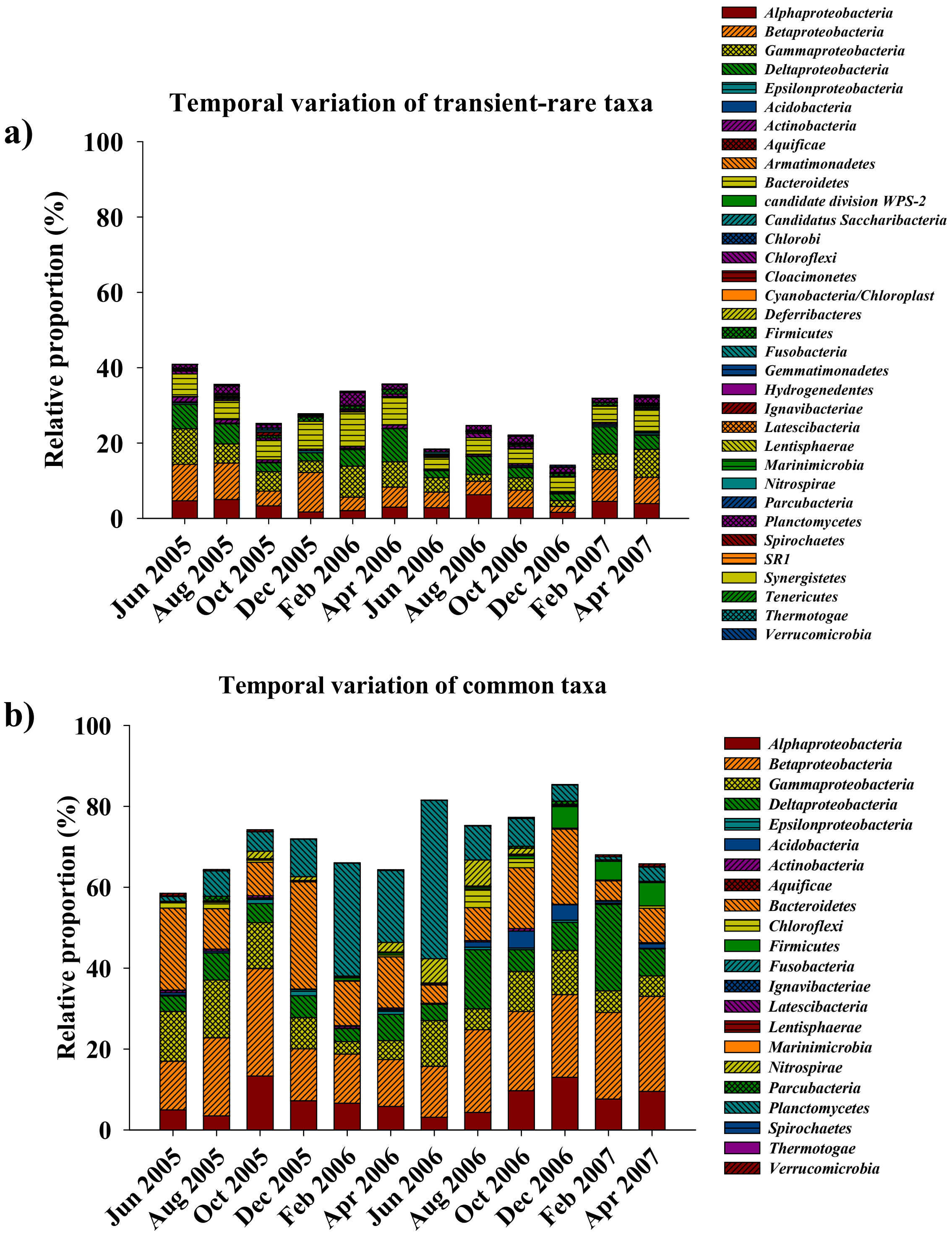
a)
Temporal variation of transient-rare taxa
Relative proportion (%)
0
20
40
60
80
100
Jun 2005
Aug 2005
Oct 2005
Dec 2005
Feb 2006
Apr 2006
Jun 2006
Aug 2006
Oct 2006
Dec 2006
Feb 2007
Apr 2007
Alphaproteobacteria
Betaproteobacteria
Gammaproteobacteria
Deltaproteobacteria
Epsilonproteobacteria
Acidobacteria
Actinobacteria
Aquificae
Armatimonadetes
Bacteroidetes
candidate division WPS-2
Candidatus Saccharibacteria
Chlorobi
Chloroflexi
Cloacimonetes
Cyanobacteria/Chloroplast
Deferribacteres
Firmicutes
Fusobacteria
Gemmatimonadetes
Hydrogenedentes
Ignavibacteriae
Latescibacteria
Lentisphaerae
Marinimicrobia
Nitrospirae
Parcubacteria
Planctomycetes
Spirochaetes
SR1
Synergistetes
Tenericutes
Thermotogae
Verrucomicrobia
b)
Temporal variation of common taxa
Relative proportion (%)
0
20
40
60
80
100
Jun 2005
Aug 2005
Oct 2005
Dec 2005
Feb 2006
Apr 2006
Jun 2006
Aug 2006
Oct 2006
Dec 2006
Feb 2007
Apr 2007
Alphaproteobacteria
Betaproteobacteria
Gammaproteobacteria
Deltaproteobacteria
Epsilonproteobacteria
Acidobacteria
Actinobacteria
Aquificae
Bacteroidetes
Chloroflexi
Firmicutes
Fusobacteria
Ignavibacteriae
Latescibacteria
Lentisphaerae
Marinimicrobia
Nitrospirae
Parcubacteria
Planctomycetes
Spirochaetes
Thermotogae
Verrucomicrobia
